# Amplicon sequencing with Oxford nanopore technologies as a diagnostic alternative for small ruminant lentiviruses in sheep

**DOI:** 10.1038/s41598-026-36989-y

**Published:** 2026-01-25

**Authors:** Magdalena Serrano, Carmen González, Rosa Roy, Almudena Fernández, Juan José Arranz, Jorge Hugo Calvo, Mª Ángeles Jiménez, Fernando Puente-Sánchez

**Affiliations:** 1 Departamento de Mejora Genética Animal, Instituto Nacional de Investigación y Tecnología Agraria y Alimentaria (INIA-CSIC), Ctra. de La Coruña km 7,5, 28040 Madrid, Spain; 2 Departamento de Biología, Facultad de Ciencias, Universidad Autónoma de Madrid, Cantoblanco, 28049 Madrid, Spain; 3 Departamento de Producción Animal, Facultad de Veterinaria, Universidad de León, Campus de Vegazana, 24007 Leon, Spain; 4 ARAID-Centro de Investigación y Tecnología Agroalimentaria de Aragón (CITA)-Instituto Agroalimentario de Aragón-IA2, Av. de Montañana 930, 50059 Zaragoza, Spain; 5 Department of Aquatic Sciences and Assessment, Swedish University for Agricultural Sciences (SLU), Lennart Hjelms väg 9, 756 51 Uppsala, Sweden

**Keywords:** SRLV diagnosis, PCR amplicons, ONT-Nanopore sequencing, Biological techniques, Biotechnology, Diseases, Microbiology, Molecular biology

## Abstract

In Europe, Maedi-Visna disease has high prevalence rates at the individual and flock levels, respectively, and is regarded as one of the most significant infectious disease in sheep. The lack of treatment or a commercial vaccine underscores the need for accurate and reliable diagnostic tools to support control programs. Conventional methods, including ELISA and qPCR, provide useful but incomplete information due to the genetic variability of small ruminant lentiviruses (SRLVs) and the heterogeneous host immune response. In this work, third-generation sequencing was assessed as a diagnostic strategy, focusing on Oxford Nanopore Technologies amplicon sequencing of different regions of the virus genome. DNA from whole blood, PBMCs, semen, and nasal mucosa of 44 rams previously tested for Maedi-Visna virus by ELISA was used to generate amplicons of the *gag*,* pol*, and p25 genes. Sequencing showed that blood DNA was the most reliable source for SRLVs detection by Nanopore, despite the low proportion of monocytes present in this medium. Compared with conventional approaches, Nanopore sequencing reduced the proportion of false negatives observed with ELISA (42%) and qPCR (77%). These results highlight Nanopore amplicon sequencing as a promising diagnostic alternative, combining epidemiological relevance with technological innovation to enhance SRLVs detection and strengthen control strategies for sustainable disease management.

**Supplementary Information:**

The online version contains supplementary material available at 10.1038/s41598-026-36989-y.

## Introduction

Maedi-Visna virus (MVV) and caprine arthritis-encephalitis virus (CAEV) are collectively referred to as small ruminant lentiviruses (SRLVs), due to their genetic, structural, and pathogenic similarities. These viruses cause persistent infections characterized by a slow progression before clinical signs emerge. Both MVV and CAEV belong to the family Retroviridae, genus *Lentivirus*. To date, five main SRLV genotypes (A–E) and more than 28 subtypes have been identified^1,2^. This high phylogenetic diversity results in substantial genetic and antigenic variability, which complicates both, serological and molecular diagnosis^3^.

Although most SRLV infections remain subclinical, a minority of animals develop progressive syndromes such as dyspnea, arthritis, or neurological symptoms, often leading to death. Maedi-Visna (MV) is a chronic, progressive, and currently incurable disease of sheep that leads to substantial economic losses in the dairy industry and acts as a barrier to animal trade worldwide^4^. Clinical manifestations include chronic mastitis, progressive wasting, arthritis, and neurological signs. Symptoms typically appear after prolonged incubation periods, extending from three to eight years. Economic losses stem from ~ 20% mortality, decreased fertility, reduced milk yield, and higher rates of mastitis, lamb mortality, and culling. The economic impact of MVV includes trade restrictions, early culling, and production losses. Infected dairy sheep may produce up to 3% less milk and exhibit an approximately 60% increase in somatic cell count (SCC)^5,6^. Subclinical increases in SCC have been associated with systemic lesions affecting up to 20% of infected animals^7^. In meat sheep, SRLV infection has been associated with smaller lamb crops and altered birth weights^5^. The long asymptomatic period of SRLV infections may compromise maternal condition and nutrient transfer, ultimately affecting fetal development.

Globally, the highest flock-level prevalence has been reported in Asia (66%), followed by Europe and North America (44.4%–48.6%), whereas Africa shows the lowest prevalence (7.7%)^4^. High MV prevalence in Europe is associated with intensive sheep farming. For instance, Greece (66%) and Spain (50%) exhibit the highest individual-level prevalence rates in Europe, both being major sheep-milk producing countries. Consequently, MVV infection is thus of major concern in Spain, representing one the most prevalent infectious disease in intensively managed dairy sheep.

Currently, no effective drugs or vaccines are available for the treatment or prevention of small ruminant lentivirus (SRLV) infections. Consequently, control programs largely rely on the identification and removal of infected animals. A complementary strategy involves the identification of genetically resistant individuals and the incorporation of this information into breeding programs to enhance flock-level resistance. Several sheep breeds—such as Texel, Border Leicester, Finnish Landrace, Biellese, Churra, and Assaf—have been reported as more susceptible to SRLV infection than others, including Rabouillet, Île de France, Suffolk, Columbia, Rambo, Polipay, Delle Langhe, Bergamasca, Rasa Navarra, and Rasa Aragonesa^8–10^. In addition, multiple host genetic loci have been associated with resistance or susceptibility, including CCR5^11^, DRB1^12^, DPPA2/DPPA4 and SYTL3^13^, TMEM154^8^, TLR9^14^, ZNF389^15^, and TRIM5α^16^. Assisted selection for gene variants conferring animals SRLV resistance can be a complementary tool in the fight against these diseases. However, in order to detect these variants and their effect on the resistance/susceptibility of animals to infection by SRLVs, it is essential to have precise and reliable phenotypes of the animals’ health status.

Therefore, regardless of the chosen strategy, reliable, cost-effective, and scalable diagnostic tools are crucial. Current diagnostic methods, however, are challenged by the extensive genetic and antigenic diversity of SRLVs. Their high mutation and recombination rates give rise to rapidly evolving viral populations, often existing as quasi-species within a single host^17^, with one variant usually dominating^18^. This variability complicates serological diagnosis, particularly with ELISA, the most widely used method, which nevertheless has several drawbacks, but also with qPCR strategies.

Most commercial ELISAs have not been validated against internationally recognized reference assays (e.g., radio immunoprecipitation or Western blot) as recommended by WOAH Terrestrial Manual, and only a minority meet these standards^19^. ELISA-based diagnosis is further limited by fluctuations in antibody titters, which may yield false negatives^19–21^. In addition, seroconversion may be delayed or even absent in infected animals^18^. Moreover, most ELISAs use antigens such as p28 (CAEV), p25 (MVV), or gp135, which are typically derived from the grow of a single viral isolate in cell cultures. Given the restricted cross-reactivity between SRLV groups, monostrain-based assays frequently miss infections^3,20^.

Molecular techniques such as PCR and in situ hybridization offer improved sensitivity and specificity over serological methods^22^, but they still face limitations^17,18^. Recent studies suggest that combining ELISA and PCR enhances diagnostic accuracy, reflecting both, the antigenic variability of the virus and differences in host immune responses^5,23^. Despite the available diagnostic tools, a true gold standard test has not yet been established.

Third generation sequencing techniques, such as Oxford Nanopore Technologies (ONT) long read sequencing, are a promising alternative to address the unique challenges of MV diagnosis. These platforms (e.g., MinION, GridION, PromethION) enable real-time sequencing by generating read data as DNA translocates through a nanopore, allowing for immediate downstream analysis and significantly reducing time to results^24,25^. Amplicon-based Nanopore sequencing has been successfully applied to detect several human and animal pathogens, including Zika virus^26^, salmonid RNA viruses^27^, human enteroviruses^28^, SARS-CoV-2^29,30^, Dengue virus^31^, Tilapia lake virus^32^, Toxoplasma gondii^33^, and Avian Influenza^34^. This approach not only facilitates rapid detection but also enables the genotyping of infecting strains, potentially enhancing control strategies for SRLV infection.

Considering the foregoing, our study aimed to develop a more precise and efficient diagnostic approach using ONT long read sequencing. Specifically, we focused on sequencing and characterizing SRLV PCR amplicons in sheep blood, nasal mucosa, and semen, and compared the results with those obtained using qPCR and ELISA. With this approach, we aim not only to diagnose the presence of the virus in animals more accurately, but also to detect the infecting genotype, since the sequencing of long fragments of the viral genome allows for a more precise characterisation of the strains detected.

## Results

**Fig. 1:**
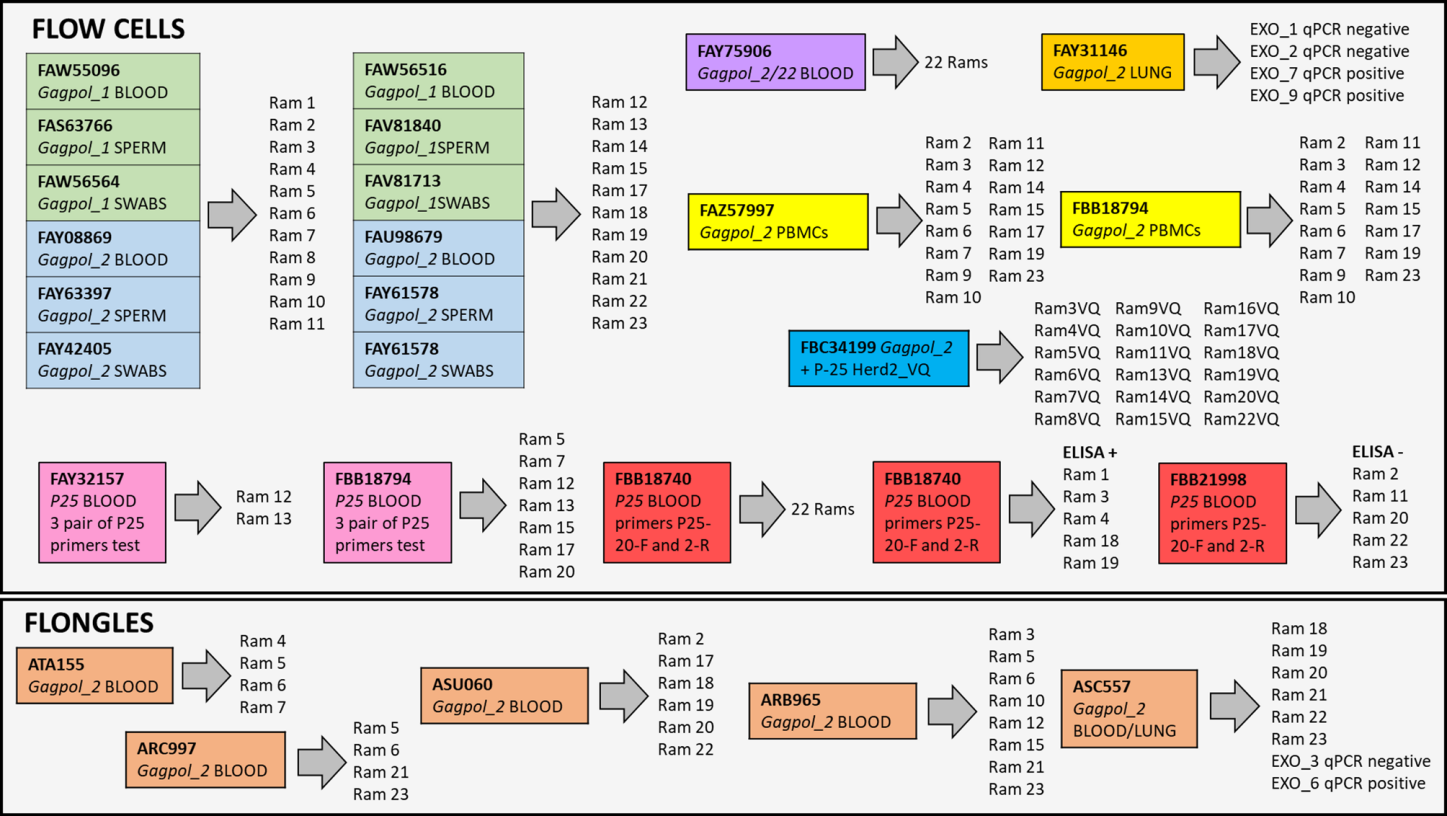
Experimental design and overview of Nanopore sequencing runs using R10.4.1 Flow Cells and Flongles. Amplicons targeting gag, pol, and p25 genes from blood, semen, nasal mucosa, and lung samples of 22 rams were sequenced. Each coloured box represents a sequencing run including the primer set used, the type of sample, and the IDs of the animals. Flow Cells are grouped by replicate and sample type, including special runs for PBMCs-enriched DNA (yellow), lung DNA from qPCR-tested animals (orange), and p25 validation (red). Flongle runs (bottom) include additional blood and lung samples.

An overview of all sequencing runs and sample types is illustrated in Fig. 1 to assist in monitoring the experimental development of the work.

### SRLV detection by qPCR

Of the 22 rams tested for SRLV using qPCR at the EXOPOL laboratory, only two animals (Rams 6 and 12) tested positive. Both showed high quantification cycle (Cq) values of 30 and 36, respectively. These two rams were also positive by ELISA test.

### Nanopore sequencing of blood samples

Table 1 summarize the Nanopore sequencing results of SRLV GAGPOL_1 and GAGPOL_2 amplicons obtained from blood samples of the 22 rams across different flow cells, and shows the quantitative results of sequencing, including number of passed reads (Q ≥ 10), number of mapped reads against SRLV genomes/genes, and number of mapped reads above quality thresholds. Also target genomes and genes covered by the sequences are summarized (see Flow Cells design in Fig. 1).

#### Flow cells GAGPOL_1 amplicons

Two flow cells (FAW55096 and FAW56516) were used for sequencing GAGPOL_1 amplicons, each including samples from 11 rams plus a negative control. Nanoplot metrics, sequencing, and mapping details are displayed in Supplemental Files S5 and S6.

Among the 10 ELISA-positive rams, 7 were also positive by Nanopore sequencing of GAGPOL_1 amplicons (Rams 3, 6, 8, 9, 12, 14, and 18), while 3 were negative (Rams 1, 4, 19). Of the 12 ELISA-negative rams, 5 were positive (Rams 2, 5, 7, 13, and 17) and 7 were negative (Rams 10, 11, 15, 20, 21, 22, and 23) by Nanopore sequencing.

Most reads passing quality thresholds mapped to the MVV genome HQ848062.1 -isolate 697-specifically within the *gag* gene (positions 291–1043, amplicon size ~ 752 bp, mean identity 90%). Notably, Ram 6, which yielded the highest number of SRLV aligned reads, also had a pair of reads mapping to the *pol* gene (positions 3709–4884, ~ 1,150 bp, mean identity 86%). Rams 5 and 13 showed a significant number of high quality reads (~ 737 bp) with 92–94% identity to the CAEV genome FJ195346.1 -isolate Ov496-, mapping to *gag* positions 330–1067. Additionally, Ram 5 yielded some high-quality reads (~ 700 bp, 91–93% identity) mapping to the Italian SRLV genome MH374288.1 (SRLV042) *gag* gene. Only Ram 6 yielded *pol* gene reads which meet mapping quality thresholds, highlighting limited amplification success of the nested PCR targeting this gene. Across all samples and flow cells, negative control samples didn’t yield any SRLV sequence.

In summary, GAGPOL_1 primers showed good performance in generating amplicons of the *gag* gene across most samples. However, amplification of the *pol* gene by nested PCR was inefficient, with reads detected only in one animal (Ram 6).

#### Flow cells: GAGPOL_2 amplicons

Two flow cells (FAY08869 and FAU98679) were used to sequence GAGPOL_2 amplicons from blood DNA of 11 rams each, along with a negative control. Nanoplot metrics, sequencing, and mapping details are displayed in Supplemental Files S5 and S6.

Among the 10 ELISA-positive rams, 9 were also positive and 1 negative (Ram 18) by Nanopore sequencing. Of the 12 ELISA-negative rams, 7 tested positive (Rams 5, 7, 11, 13, 15, 20, 22) and 5 were negative (Rams 2, 10, 17, 21, and 23). Notably, Rams 1, 4, 11, 15, 19, 20, and 22, which were negative using GAGPOL_1, tested positive with GAGPOL_2. Conversely, Rams 2, 17, and 18, which were GAGPOL_1 positive, tested negative using GAGPOL_2.

As observed with GAGPOL_1 amplicons, high-quality reads (~ 742 bp) from positive samples mapped to the *gag* gene (positions 291–1043) and/or the *pol* gene (positions 1795–2698) of the MVV reference genome HQ848062.1 with an 89% of mean identity. Seven samples had reads mapping only to *gag*; Ram 3 had reads mapping only to *pol*; and 5 Rams had reads mapping to both, *gag* and *pol* genes.

Rams 5 and 6 also had reads (~ 730 bp, 94–96% identity) mapping to the *gag* gene of the CAEV isolate Ov496 (FJ195346.1). Additionally, Rams 6, 9, and 12 showed reads (~ 900 bp) mapping (mean identity 85%) to the non-functional *pol* gene of AY454175.1, isolated from a Swiss goat. As in previous runs, no reads mapping to SRLV genomes appeared in the negative controls.

GAGPOL_2 primers showed superior performance compared to GAGPOL_1, generating amplicons for both *gag* and *pol* regions in a larger number of animals. Despite differences in sequencing time between Flow Cells GAGPOL_1 and GAGPOL_2, the number of reads obtained was very similar between them (Supplemental File S5). However, they showed different performances in mapping reads onto SRLV genomes. For most samples, Flow Cells GAGPOL_1 obtained a higher number of reads per barcode. This could be due, among other things, to the fact that the number of active pores in FAU98679 GAGPOL_2 was (681), which was much lower than in the rest of the Flow Cells (> 1000 pores). Nor can we rule out differences in viral DNA loaded into each of the libraries.

For the 10 ELISA positive animals, GAGPOL_1 and GAGPOL_2 sequencing runs detected SRLV reads for 7 and 9 samples, respectively, and for the 12 ELISA negative rams, the false negative rate detected were 41,6% and 58,3%, respectively. The combined result of the diagnosis using both strategies confirmed infection with SRLVs in all ELISA-positive animals, and detected 75% false negatives in the group of ELISA-negative animals.

**Table 1:** Results of nanopore sequencing of both GAGPOL_1 and GAGPOL_2 amplicons of DNA from blood samples of the 22 Rams used in this study. nPR, number of passed reads (Q ≥ 10); nHIT, number of reads successfully assigned to a SRVL genome; nRQT, number of reads above quality thresholds.

		FAW55096-FAW56516 GAGPOL_1					FAY08869-FAU98679 GAGPOL_2				
***Ram_ID***	***ELISA***	nPR	nHIT	nRQT	ID Genome	Gene	nPR	nHIT	nRQT	ID Genome	Gene
Ram1	+	143,080	4	0			218,587	113	60	HQ848062.1	*gag*
Ram3	+	109,504	31	14	HQ848062.1	*gag*	167,229	17	5	HQ848062.1	*pol*
Ram4	+	215,220	9	0			284,655	73	25	HQ848062.1	*gag*
Ram6	+	518,158	76,291	26,402	HQ848062.1	*gag/pol*↓	174,299	3,662	660	AY454175.1 FJ195346.1 HQ848062.1 JX469608.1	*pol gag gag/pol gag*
Ram8	+	116,845	1,640	451	HQ848062.1	*gag*	184,900	91	31	HQ848062.1	*gag/pol*↓
Ram9	+	81,394	244	71	HQ848062.1	*gag*	173,048	472	183	AY454175.1 HQ848062.1	*pol gag/pol*
Ram12	+	85,716	430	102	HQ848062.1	*gag*	121,571	113	41	AY454175.1 HQ848062.1	*gag gag*
Ram14	+	47,257	422	125	HQ848062.1	*gag*	89,835	5	2	HQ848062.1	*gag*
Ram18	+	71,295	93	25	HQ848062.1	*gag*	187,837	0	0		
Ram19	+	37,765	1	0			135,812	10	3	HQ848062.1	*gag*
Ram2	-	287,564	46	11	HQ848062.1	*gag*	360,573	13	0		
Ram5	-	226,324	3,734	1,648	FJ195346.1 HQ848062.1 MH374288.1	*gag gag gag*	176,566	553	217	FJ195346.1 HQ848062.1	*gag gag/pol*↓
Ram7	-	115,000	142	32	HQ848062.1	*gag*	231,919	14	11	HQ848062.1	*gag*
Ram10	-	78,289	10	0			174,606	1	0		
Ram11	-	93,006	6	0			174,963	57	24	HQ848062.1 MT993912.1	*gag/pol *↓ * pol*
Ram13	-	37,899	404	131	FJ195346.1 HQ848062.1	*gag gag*	131,787	101	42	HQ848062.1	*gag/pol*↓
Ram15	-	47,099	0	0			117,291	14	4	HQ848062.1	*gag*
Ram17	-	66,887	157	58	HQ848062.1	*gag*	182,607	1	0		
Ram20	-	81,649	0	0			113,858	51	36	HQ848062.1	*gag*
Ram21	-	32,934	0	0			133,936	0	0		
Ram22	-	28,259	0	0			103,620	44	8	HQ848062.1	*gag*
Ram23	-	46,968	1	0			116,864	2	0		

AY454175.1 = Small ruminant lentivirus isolate SNCR5560 gag protein gene.

FJ195346.1 = Caprine arthritis encephalitis virus Ov496, complete genome.

HQ848062.1 = Visna/maedi virus isolate 697, complete genome.

JX469608.1 = Small ruminant lentivirus isolate 2–97 g gag protein (gag) gene.

MH374288.1 = Small ruminant lentivirus isolate SRLV042, complete genome.

MT993918.1 = Small ruminant lentivirus isolate USMARC-199,916,193-r, complete genome.

↓ indicates a low number of *pol* gene sequences.

To test scalability, a third flow cell (FAY75906) was used to sequence GAGPOL_2 amplicons from all 22 rams and a negative control over a 95 h run, with library reloads at 24 h and 48 h. Nanoplot metrics, sequencing, and mapping details are displayed in Supplemental Files S5 and S6.

Among ELISA-positive rams, 8 were positive and 2 negative (Rams 3 and 18), and among ELISA-negative rams, 4 tested positive (Rams 5, 13, 15, 17) and 8 negative (Rams 2, 7, 10, 11, 20, 21, 22, 23). In this case the detection of false ELISA negative animals was lower than in previous runs, 33.3%.

The 12 Nanopore positive rams had variable number of high-quality reads (88–92% identity) mapping to the MVV isolate HQ848062.1. In all of them, reads mapped to the *gag* region (positions 291–1043; ~752 bp). Rams 4, 6, 9, and 13 also had reads mapping to the *pol* gene (positions 1795–2700; ~905 bp). Intriguingly, Ram 4 (ELISA positive), which in the previous runs GAGPOL_1 and GAGPOL_2 yielded few quality reads mapped to SRLVs, produced in this case180 reads (~ 720 bp, 95–97% identity) mapping to the *gag* gene of the isolate FJ195346.1. However, Ram 5 (ELISA negative), which consistently tested positive with a large number of quality reads mapped to SRLVs in Flows GAGPOL_1 and GAGPOL_2, yielded in this run only a small number of valid sequences (Supplemental File S6). As in previous runs, no reads mapping to SRLV genomes appeared in the negative controls.

Summarising the diagnosis of SRLVs across the five flow cells, taking into account the number of times that virus reads were detected in each sample, we can conclude that: (1) All ELISA-positive rams were confirmed as positives, except for ram 18, which showed an inconclusive result; (2) of the 12 ELISA-negative rams, 6 tested positive with Nanopore sequencing, 3 were confirmed as negative, and 3 (Rams 11, 20 and 22), showed an inconclusive result.

#### Flongle nanopore sequencing using GAGPOL_2 amplicons

To evaluate the diagnostic performance of Flongle ONT devices, a cost-effective and lower-throughput Nanopore sequencing platform (typically 50–100 active pores), sequencing experiments were conducted by using GAGPOL_2 amplicons from blood-derived DNA. Runs were performed with 4, 6, and 8 samples each (Fig. 1). A comprehensive summary of the sequencing metrics generated using NanoPlot, is provided in Supplementary File S5. In Supplementary File S6 sequencing results for GAGPOL_2 amplicons from blood samples of selected rams along with diagnostic outcomes, including the most reliable target genomes or genes identified, are shown.

The ARC997 Flongle, which included samples from rams 5, 6, 21, and 23, yielded 128,641 reads over 4 h and 23 min. Rams 5 and 6, previously diagnosed as positive using standard Flow Cells, showed reads mapping to the *gag* gene of the HQ848062.1 MVV genome with 89% of mean identity and read sizes around 752 bp. Ram 6 also had reads (~ 903 bp, mean identity 88%) mapping against the *pol* gene.

The ATA155 Flongle, containing samples from rams 4, 5, 6, and 7, yielded 104,338 reads over 5 h and 10 min Only Rams 4 and 6 were positive by Flongle sequencing. Ram 4 showed reads mapping (92–96% identity) to *gag* regions of both FJ195346.1 and HQ848062.1 genomes. Ram 6 showed reads mapping to both the *gag* and *pol* regions of HQ848062.1 genome, as well as a unique read (~ 896 bp, 85% identity) mapping to the *pol* gene MT993918.1 (isolate USMARC-199916193). Rams 5 and 7 tested negative in this run.

The ASU060 Flongle, which included samples from rams 2, 17, 18, 19, 20, and 22, all previously classified as positive by Flow Cell sequencing, produced 505,886 reads over 5 h and 13 min. Rams 2, 17, 18, and 19 tested negative. Ram 20 showed only three reads (~ 740 bp, 91% of identity) mapping to the *gag* region of HQ848062.1 MVV genome. Ram 22 showed two reads (~ 600 bp, 92% of identity) mapping to the *gag* gene of JN184353.1 (Small ruminant lentivirus isolate 166 gag protein complete cds).

The ARB965 Flongle, including samples from rams 3, 5, 6, 10, 12, 15, 21, and 23, yielded 62,425 reads over 4 h and 27 min. Rams 3, 10, 15, 21, and 23 were negative. Ram 5 had a single high-quality read (738 bp, 90% of identity) mapping to the *gag* gene of the HQ848062.1 genome. Ram 6 had 27 reads mapping to *gag* (~ 744 bp, 89% of identity) and *pol* (~ 900 bp, 88% of identity) of HQ848062.1. Finally, Ram 12 had 3 reads (~ 742 bp, 88% of identity) mapping to the *gag* gene of HQ848062.1 and a single read (877 bp, 84% of identity) to the *gag* gene of AY454175.1 (Small ruminant lentivirus isolate SNCR5560).

In all Flongle runs, control samples showed no reads mapping to SRLV genomes. Notably, Ram 6 consistently tested positive across all blood sample runs, both with Flow Cells and Flongles, indicating a high viral load in this animal.

### Nanopore sequencing of GAGPOL_2 amplicons from blood and lung samples using flow cells and flongles

To further assess the diagnostic performance of GAGPOL_2 amplicon sequencing, both standard Flow Cells and Flongle devices were employed to analyze DNA extracted from blood of our batch of rams and lung samples from EXOPOL lab. Lung samples were previously diagnosed by qPCR at EXOPOL lab using the EXOone Maedi Visna-CAEV oneMIX qPCR kit SRLV (Supplemental Files S1 and S2, and Fig. 1).

Flow Cell FAY31146 including four lung samples (2 qPCR negative: EXOlung_1 and EXOlung_2; and 2 qPCR positive: EXOlung_7 and EXOlung_9) produced 4.6 million reads over a 48-hour and 38-minute sequencing run, including a library reload at 24 h. Nanoplot metrics, sequencing, and mapping details are displayed in Supplemental Files S5 and S6.

Flongle ASC557 included 8 samples (6 blood samples from our Rams and 2 lung samples EXOlung_3 qPCR negative and EXOlung_6 qPCR positive). Details of the Nanopore runs and sequencing performance are shown in Supplementary Files S5 and S6.

In Flow Cell FAY31146, the two qPCR-negative lung samples (EXO_lung_1 and EXO_lung_2) each yielded three high-quality reads mapping to the *gag* gene (~ 752 bp, positions 292–1043, 90% of identity) of the AF479638.1 Ovine lentivirus (Portuguese strain P1OLV). In contrast, EXOPOL qPCR-positive lung samples (EXO_lung_7 and EXO_lung_9) yielded thousands of reads mapping to multiple SRLV genomes. Among these high amount of SRLV mapped reads, a selection of those with more than 91% of alignment identity yielded the following results:

- For the EXO_lung_7 sample, 760 reads (~ 748 bp, 91% of identity) mapped to the *gag* gene (positions 292–1040) of the P1OLV isolate AF479638.1. Over 1,560 reads with a mean alignment identity of 95%, mapped to the *gag* gene (positions 330–1067) of the Caprine arthritis encephalitis virus CAEV Ov496 genome (FJ195346.1). More than 200 reads mapped against the *gag* (~ 732 bp, positions 330–1076) and *pol* (~ 904 bp, positions 1824–2740) genes of the isolate SRLV001 (MG554402.1) with 92% of mean alignment identity, and 6,241 reads matched, with a mean identity of 93%, the *gag* (~ 732 bp, positions 330–1048) and *pol* (~ 895 bp, positions 1824–2721) genes of the isolate SRLV042 (MH374288.1).
- For the EXO_lung_9 more than 35,000 reads mapped with 95% of identity to the *gag* gene (723 bp, positions 330–1067) of CAEV Ov496 (FJ195346.1). Approximately 870 reads (91% of mean identity) matched the *gag* (737 bp, positions 342–1079) and *pol* (897 bp, positions 1843–2740) genes of SRLV001 (MG554402.1), and ~ 72,000 reads mapped (92% of mean identity) against the *gag* (730 bp, positions 330–1066) and *pol* (897 bp, positions 1824–2721) genes of the isolate SRLV042 (MH374288.1).

In Flongle ASC557, rams 18, 20, 21, 22 and 23 tested negative, while ram 19 tested positive but with a single high quality mapped read (~ 720 bp, 95% of identity) matching the *gag* gene (positions 344–1064) of the CAEV Ov496 (FJ195346.1). Notably, EXO_lung_3, previously classified as qPCR-negative (Cq = 0), tested positive with 120 high-quality reads (~ 900 bp, 90% of identity) mapping to the *pol* gene (positions 1824–2724) of SRLV042 (MH374288.1). EXO_lung_6, a qPCR-positive sample (Cq = 22.2), showed more than 5,500 high-quality reads mainly mapping (94% of mean alignment identity) to the *gag* (737 bp, positions 330–1067) and *pol* (904 bp, positions 1831–2735) genes of CAEV Ov496 (FJ195346.1), as well as four reads (~ 735 bp and 91% of identity) mapping to the *gag* gene (positions 331–1066) of SRLV042 (MH374288.1).

As in previous experiments, control samples showed no reads mapping to SRLV genomes.

Table 2 summarizes the results of the Flow Cell and Flongle Nanopore sequencing of GAGPOL_2 amplicons from blood and lung DNA samples along with the most reliable genome and gene alignments identified.

**Table 2:** Results of flow cell and flongle nanopore sequencing of GAGPOL_2 amplicons of DNA from blood and lung samples. qPCR test (Cq) from EXOPOL lab; nPR, number of nanopore passed reads (Q ≥ 10); nHIT, number of reads successfully assigned to a SRVL genome; nRQT, number of reads above quality thresholds; genome ID target genomes and genes.

		nPR	nHIT	nRQT	Genome ID	genes
Sample ID	qPCR test (Cq)	FAY31146 GAGPOL_2				
EXO_lung_1	0	331,994	16	3	AF479638.1	*gag*
EXO_lung_2	0	18,501	7	3	AF479638.1	*gag*
EXO_lung_7	23.7	365,460	293,827	232,910	AF479638.1 **FJ195346.1** MG554402.1 MH374288.1	*gag* ***gag*** *gag/pol* *gag/pol*
EXO_lung_9	22.9	493,147	323,748	149,065	**FJ195346.1** MG554402.1 MH374288.1	***gag*** *gag*,* pol* *gag*,* pol*
Unclassified		2,430,294	1,279,326			

Ram/Sample ID	qPCR test (Cq)	ASC557 GAGPOL_2				
Ram18	0	8,526	0	0		
Ram19	0	10,254	1	1	FJ195346.1	*gag*
Ram20	0	13,410	0	0		*-*
Ram21	0	19,520	0	0		*-*
Ram22	0	13,718	1	0		*-*
Ram23	0	29,307	1	0		*-*
EXO_lung_3	0	11,136	222	120	MH374288.1	*pol*
EXO_lung_6	22.2	13,431	7,223	5,687	**FJ195346.1** MH374288.1	***gag***,*** pol*** *gag*
Unclassified		132,295	7,014			

AF479638.1 Ovine lentivirus strain P1OLV, complete genome.

FJ195346.1 Caprine arthritis encephalitis virus Ov496, complete genome.

MG554402.1 Small ruminant lentivirus isolate SRLV001, complete genome.

MH374288.1 Small ruminant lentivirus isolate SRLV042, complete genome.

In lung DNA samples from EXOPOL, the primary distinction between qPCR-positive and qPCR-negative animals was the consistent detection of reads mapping to the Caprine arthritis encephalitis virus Ov496 genome (FJ195346.1) in the qPCR-positive samples which were absent in the qPCR-negative group. Conversely, reads mapping to P1OLV (AF479638.1) and SRLV042 (MH374288.1) were detected by Nanopore sequencing in both groups. This suggests that the qPCR TaqMan probe predominantly targets CAEV-related sequences, which appear to be detected primarily.

### Nanopore sequencing in isolates of peripheral blood mononuclear cells (PBMCs) samples

Two Flow Cells, FAZ57997 and FBB18794 (Fig. 1), each containing GAGPOL_2 PCR amplicons from 15 rams and one negative control sample, yielded approximately 0.7 and 0.4 million reads, respectively. Sequencing runs lasted 15 h for FAZ57997 and 24 h for FBB18794. A detailed summary of the Nanoplot metrics , the sequencing performance, and the mapping results are provided in Supplementary Files S5 and S6.

In the FAZ57997 Flow Cell, six rams (IDs 5, 6, 9, 12, 14, and 19) tested positive for SRLV in PBMCs-derived DNA samples, although they yielded very few high-quality reads in this run. Notably, all these rams were also found to be SRLV-positive in whole blood-based Nanopore sequencing. Rams 5, 6, 12 and 19 produced 13, 41, 1 and 1 quality reads, respectively, with an approximate length of 750 bp and a mean alignment identity of 90% matching to the *gag* gene (positions 291–1040) of the Visna/maedi virus isolate 697 (HQ848062.1). Additionally, Ram 9 had a single high-quality read (1,277 bp, 92% of identity) aligning to the *pol* gene (positions 935–1596) of SRLV isolate SNCR5560 (AY454175.1), and Ram 14, showed a single high-quality read (733 bp, 90% of identity) mapping to the *gag* gene (positions 210–705) of SRLV isolate 698 (JN184359.1).

In the FBB18794 Flow Cell, five rams (IDs 5, 6, 12, 14, and 19) tested positive in PBMCs-derived DNA samples, but again yielding very few high-quality reads. All five had been previously confirmed SRLV-positive by Nanopore sequencing of whole blood samples. Rams 5, 6, 14, and 19 produced 18, 25, 2 and 1 quality reads (722–843 bp, 91% of identity), respectively, mapping to the *gag* gene (positions 291–1042) of Visna/maedi virus isolate 697 (HQ848062.1). Additional reads in Rams 5 and 14 (735–889 bp, 90% of identity) mapped to the *gag* gene of the SRLV isolate 698 (JN184359.1). Ram 6 also had few reads (870 bp, 91% of identity) mapping to the *gag* gene of the Ovine lentivirus strain P1OLV genome (AF479638.1). A single high-quality read from Ram 12 (743 bp, 89% of identity) mapped to the *gag* gene of the SRLV isolate BL5761 (JN184372.1).

As observed in previous analyses, negative control samples showed no reads mapping to SRLV genomes. Overall, the detection of SRLV using GAGPOL_2 amplicons derived from DNA extracted from PBMCs was markedly less efficient compared to amplicons derived from whole blood DNA.

### Nanopore-based SRLV diagnosis in sperm samples using GAGPOL_1 and GAGPOL_2 amplicons

Two Flow Cells (FAS63766 and FAV81840), see Fig. 1, each loaded with GAGPOL_1 PCR amplicons from 11 rams plus a negative control, generated approximately 2.4 and 0.5 million reads, respectively, during 20-hour sequencing runs.

Two additional Flow Cells, FAY63397 and FAY61578 (Fig. 1), loaded with GAGPOL_2 PCR amplicons from the same 11 rams plus a control negative, produced approximately 3.1 and 1.7 million reads, respectively, in sequencing runs lasting 5 h.

A detailed summary of Nanoplot metrics, sequencing performance, and mapping results are provided in Supplementary FilesS5 and S6.

In both GAGPOL_1 and GAGPOL_2 amplicon datasets, only two rams -Ram 11 and Ram 15- tested positive for SRLV by Nanopore sequencing of sperm samples. Notably, these animals were seronegative by ELISA but had previously tested positive for SRLV in blood samples using GAGPOL_2 amplicon sequencing.

With both primer sets, most high-quality reads (∼745 bp, ≥ 95% alignment identity) from these two rams mapped to the *gag* gene (positions 330–1075) of the Caprine arthritis encephalitis virus Ov496 (FJ195346.1; Glaria et al., 2009). Additionally, in Ram 11, and exclusively with the GAGPOL_2 primer set, three high-quality reads (∼730 bp, 93% of identity) aligned to the *gag* gene (positions 330–1060) of the SRLV genome SRLV042 (MH374288.1), isolated from Italian sheep. Importantly, no mapped reads were detected in any of the negative control samples, confirming the specificity of the positive results.

### Nanopore-based SRLV diagnosis in nasal mucosa samples using GAGPOL_1 and GAGPOL_2 amplicons

Two Flow Cells (FAW56564 and FAW81713), see Fig. 1, each loaded with GAGPOL_1 PCR amplicons from 11 rams and one negative control, produced approximately 3.5 million and 0.5 million reads, respectively, during sequencing runs of ~ 20 h. Two additional Flow Cells, FAY42405 and FAY61578 (Fig. 1), each loaded with GAGPOL_2 PCR amplicons from the same 11 rams plus a negative control, yielded 2.4 million and 1.0 million reads, respectively, after 28.5 and 7.5 h of sequencing. Nanoplot metrics, sequencing performance, and mapping results are provided in Supplementary Files S5 and S6.

For the GAGPOL_1 amplicons, only Ram 6 tested positive for SRLV in nasal mucosa samples. This ram was also positive by ELISA and by Nanopore sequencing of whole blood. A total of 162 high-quality reads (~ 745 bp, 95–96% identity) aligned to the *gag* gene (positions 330–1075) of Caprine arthritis encephalitis virus Ov496 (FJ195346.1). Additionally, 134 high-quality reads (~ 753 bp, 88% identity) mapped to the *gag* gene (positions 291–1044) of Visna/maedi virus isolate 697 (HQ848062.1).

For the GAGPOL_2 amplicons, four rams (1, 4, 5, and 23) tested SRLV-positive in nasal mucosa samples. All, except Ram 23, had previously tested positive in blood samples by Nanopore sequencing. Rams 1 and 5 showed 5 and 1 high-quality reads (~ 749 bp, ~ 95%), respectively, mapping to the *gag* gene (positions 291–1040) of Visna/maedi virus isolate 697 (HQ848062.1). Rams 4 and 23 had 18 and 4 high-quality reads (~ 734 bp, ~ 96% identity), respectively, mapping to the *gag* gene (positions 330–1064) of Caprine arthritis encephalitis virus Ov496 (FJ195346.1).

No mapped reads against SRLV genomes were found in the negative control across any of the Flow Cells.

### Summary of nanopore sequencing-based SRLV diagnosis

Table 3 summarizes the SRLV diagnostic results obtained by Nanopore sequencing of DNA amplicons derived from different type of biological samples collected from the 22 rams included in this study. In addition to blood, PBMCs, sperm, and nasal mucosa, sequencing, results from EXOPOL lung samples were also included.

**Table 3:** Summary of SRLV diagnostic results in the 22 Rams based on nanopore sequencing of GAGPOL_1 and GAGPOL_2 amplicons from multiple biological sample types. The table includes results from ELISA (blood) and qPCR (EXOPOL blood/lung), alongside nanopore-based diagnoses (Flow cells and Flongles) using amplicons from blood, PBMCs, sperm, nasal mucosa, and EXOPOL lung tissue. “+” indicates a positive SRLV diagnosis; “–” indicates a negative result.

Ram ID	ELISA blood	qPCR EXOPOL blood/lung	ONT blood	ONT PBMCs	ONT sperm	ONT nasal mucosa	ONT lung
Ram_1	+	- Cq = 0	+	-	-	+	
Ram_3	+	- Cq = 0	+	-	-	-	
Ram_4	+	- Cq = 0	+	-	-	+	
Ram_6	+	+ Cq = 30.0	+	+	-	+	
Ram_8	+	- Cq = 0	+	-	-	-	
Ram_9	+	- Cq = 0	+	+	-	-	
Ram_12	+	+ Cq = 36.0	+	+	-	-	
Ram_14	+	- Cq = 0	+	+	-	-	
Ram_18	+	- Cq = 0	+	-	-	-	
Ram_19	+	- Cq = 0	+	+	-	-	
Ram_2	-	- Cq = 0	+	-	-	-	
Ram_5	-	- Cq = 0	+	+	-	+	
Ram_7	-	- Cq = 0	+	-	-	-	
Ram_10	-	- Cq = 0	-	-	-	-	
Ram_11	-	- Cq = 0	+	-	+	-	
Ram_13	-	- Cq = 0	+	-	-	-	
Ram_15	-	- Cq = 0	+	-	+	-	
Ram_17	-	- Cq = 0	+	-	-	-	
Ram_20	-	- Cq = 0	+	-	-	-	
Ram_21	-	- Cq = 0	-	-	-	-	
Ram_22	-	- Cq = 0	+	-	-	-	
Ram_23	-	- Cq = 0	-	-	-	+	
EXO_lung_6		+ Cq = 22.2					+
EXO_lung_7		+ Cq = 23.7					+
EXO_lung_9		+ Cq = 22.9					+
EXO_lung_1		- Cq = 0					+
EXO_lung_2		- Cq = 0					+
EXO_lung_3		- Cq = 0					+

**Fig. 2:**
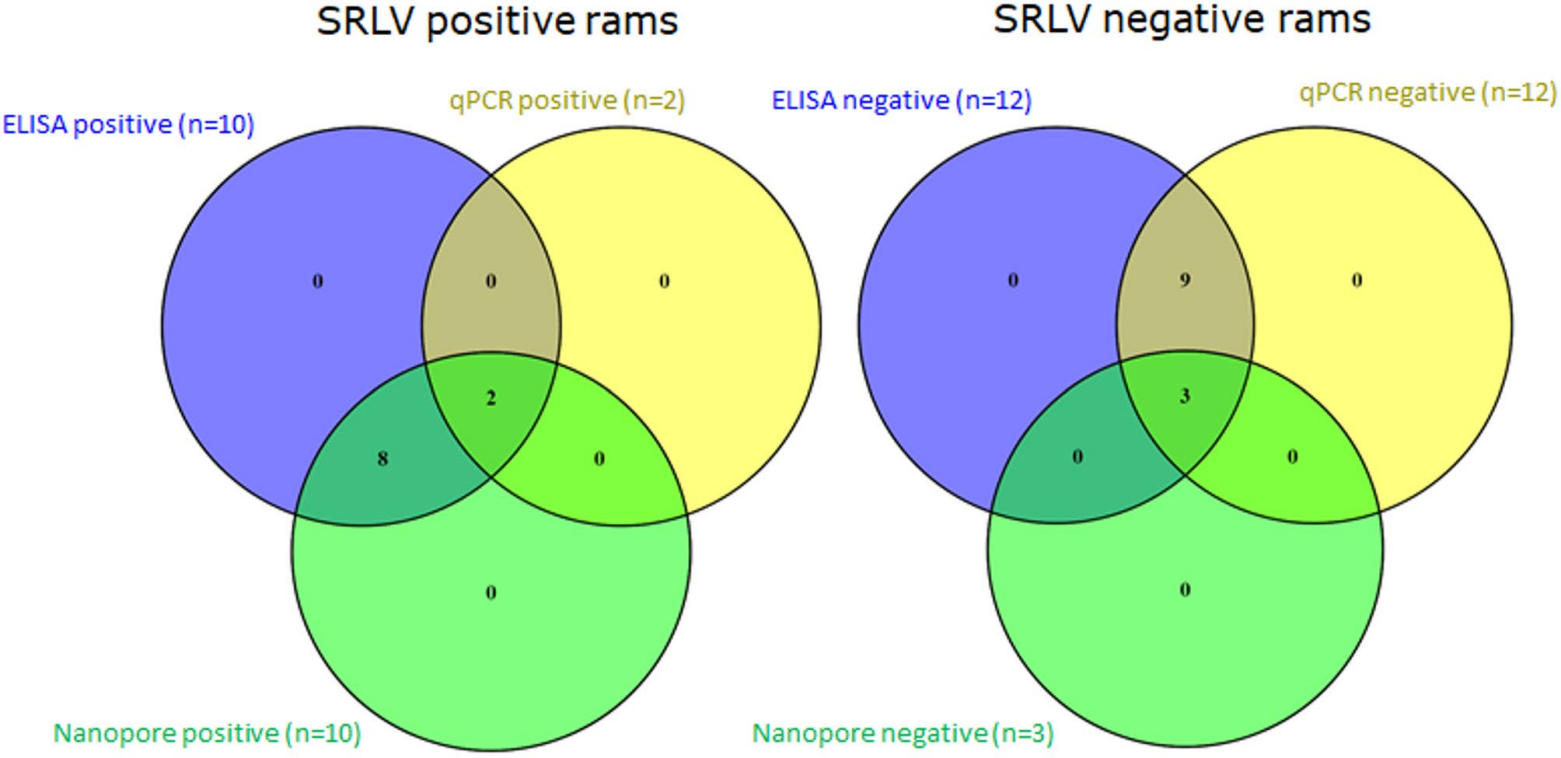
Comparison of SRLV diagnostic results in the 22 rams using ELISA (blood), qPCR (EXOPOL blood), and Nanopore sequencing (GAGPOL_1 and 2 amplicons from blood samples). The left Venn diagram shows the overlap among positive results obtained by each method. The right diagram shows the intersection of negative results. Notably, all ELISA-positive animals (*n* = 10) were also positive by Nanopore sequencing, while only 2 were confirmed by qPCR. Among the ELISA-negative animals (*n* = 12), only 3 were consistently negative across all three methods.

Figure 2 shows a Venn diagram summarising the results of SRLV diagnosis by Nanopore amplicon sequencing, ELISA and qPCR, from blood samples. As shown in this figure, ONT sequencing results were largely consistent with ELISA for positive animals. In contrast, qPCR detected only two ELISA-positive animals (Rams 6 and 12), both with borderline Cq values, highlighting the poor sensitivity of this method based in the EXOone kit for SRLV diagnosis.

Among the 12 ELISA-negative rams, only three (Rams 10, 21, and 23) were consistently negative in all Nanopore runs. Most others yielded positive results at least once, with GAGPOL_2 in 12-sample runs showing the best performance (7/12 positives; 58% false negatives). This false negative rate decreased to 33% when 22 samples were included in a single run. In contrast, ELISA and qPCR showed high concordance for negatives, but their agreement with Nanopore results was low.

### Nanopore-based SRLV detection in blood samples and variant calling of P-25 amplicons

Table 4 shows the alignment and mapping results of P-25 amplicon sequences obtained by Nanopore sequencing of DNA from blood samples across 5 Flow-Cells including the 22 rams under study. Libraries description and Nanoplot metrics are shown in Supplemental Files S3 and S5, respectively.

Consensus sequences for the *gag* protein genes more frequently detected in this group of rams were aligned to the corresponding reference sequence in order to identify alternative variants of ELISA positive and negative animals. Sequences were translated to protein to detect aminoacid substitutions. For the SRLV genome more frequently identified in this batch of samples, i.e., Visna/maedi virus isolate 697 complete genome (HQ848062.1), consensus sequences of the *gag* protein gene were also generated to compare variants.

In all ELISA positive animals, sequences mapping to 2–4 different *gag* genes were detected., confirming their positive status for the disease. However, in this case, sequences of the *gag* genes were found in only 6 of the 12 ELISA-negative rams (2, 5, 7, 13, 15, and 17). On the contrary, in ELISA negative rams 10, 11, 20, 21, 22 and 23, only sequences corresponding to the Jaagsiekte retrovirus (OQ150755.1; M80216.1; NC_001494.1/5) were identified.

**Table 4:** Summary of SRLV detection based on nanopore sequencing of P-25 amplicons in blood samples of the 22 Rams in this study.

Sample	ELISA	ONT *P*−25	Total reads	Mean quality	Mean length	Mean cove-rage	Total mapped reads	Median read length mapped reads	Mean coverage mapped reads	Mean accuracy mapped reads	Gene ID/nº sequences mapped	Gene name
Ram_1	+	+	280,228	17.0	922.8	7.0	15	1,275	95.05	87.50	JN184351.1/12 JN184353.1/3	72 gag 166 gag
Ram_3	+	+	114,795	17.8	937.8	3.8	12	1,280	96.60	85.80	JN184352.1/1 JN184353.1/7 JN184354.1/4	160 gag 166 gag 292 gag
Ram_4	+	+	558,023	17.2	870.9	7.1	59	1,281	92.90	87.60	JN184353.1/51	166 gag
Ram_6	+	+	950,129	18.3	735.2	13.1	250	1,277	92.60	87.50	JN184351.1/100 JN184352.1/4 JN184353.1/113 JN184354.1/4	72 gag 160 gag 166 gag 292 gag
Ram_8	+	+	109,058	18.1	1,000.1	23.5	79	1,275	91.05	87.10	JN184351.1/42 JN184353.1/33	72 gag 166 gag
Ram_9	+	+	223,692	18.2	933.1	11.2	84	1,276	95.50	87.30	JN184352.1/4 JN184353.1/67 JN184354.1/5	160 gag 166 gag 292 gag
Ram_12	+	+	309,216	15.6	1,100.7	15.2	160	1,275	93.40	86.40	JN184351.1/2 JN184352/42 JN184353.1/88 JN184354.1/9	72 gag 160 gag 166 gag 292 gag
Ram_14	+	+	280,073	18.3	877.5	7.0	15	1,274	93.00	87.60	JN184351.1/7 JN184353.1/8	72 gag 166 gag
Ram_18	+	+	589,681	17.3	848.4	7.1	34	1,278	96.40	86.00	JN184351.1/8 JN184352.1/17 JN184353.1/5	72 gag 160 gag 166 gag
Ram_19	+	+	450,028	17.4	949.6	2.6	33	1,274	94.20	87.00	JN184351.1/20 JN184353.1/9	72 gag 166 gag
Ram_2	-	+	631,585	17.3	862.0	2.4	13	1,283	95.80	86.00	JN184352.1/1 JN184353.1/4	160 gag 166 gag
Ram_5	-	+	258,903	16.8	1,000.5	6.8	117	1,278	93.00	87.50	JN184351.1/7 JN184352.1/82 JN184353.1/4 JN184354.1/12	72 gag 160 gag 166 gag 292 gag
Ram_7	-	+	178,002	17.0	920.4	18.4	75	965	67.25	89.05	JN184351.1/25 JN184353.1/49	72 gag 166 gag
Ram_10	-	-	52,539	17.6	994.2	0.4	1	1,136	37.90	87.90	OQ150755.1/1	Jaagsiekte
Ram_11	-	-	512,636	17.3	797.4	0.7	16	1,220	6.80	92.80	NC_001494.1/6 M80216.1/10	Jaagsiekte Jaagsiekte
Ram_13	-	+	464,139	16.4	871.4	20.1	98	1,276	95.70	87.20	JN184352.1/86 JN184353.1/1 JN184354.1/7	160 gag 166 gag 292 gag
Ram_15	-	+	284,392	17.1	1,033.1	3.7	46	445	31.30	90.60	JN184351.1/1 JN184353.1/40	72 gag 166 gag
Ram_17	-	+	369,537	17.4	821.7	4.3	9	1,288	96.30	86.60	JN184351.1/8 JN184353.1/1	72 gag 166 gag
Ram_20	-	-	876,960	17.3	778.9	1.2	26	1,111	8.70	91.60	NC_001494.1/12	Jaagsiekte
Ram_21	-	-	141,619	17.5	1,011.0	0.2	4	1,528	7.40	82.70	M80216.1/3	Jaagsiekte
Ram_22	-	-	499,610	17.3	997.6	0.7	7	2,344	14.60	92.80	NC_001494.1/5	Jaagsiekte
Ram_23	-	-	65,520	17.6	998.7	0.3	1	2,523	17.40	88.20	M80216.1/1	Jaagsiekte

**Fig. 3:**
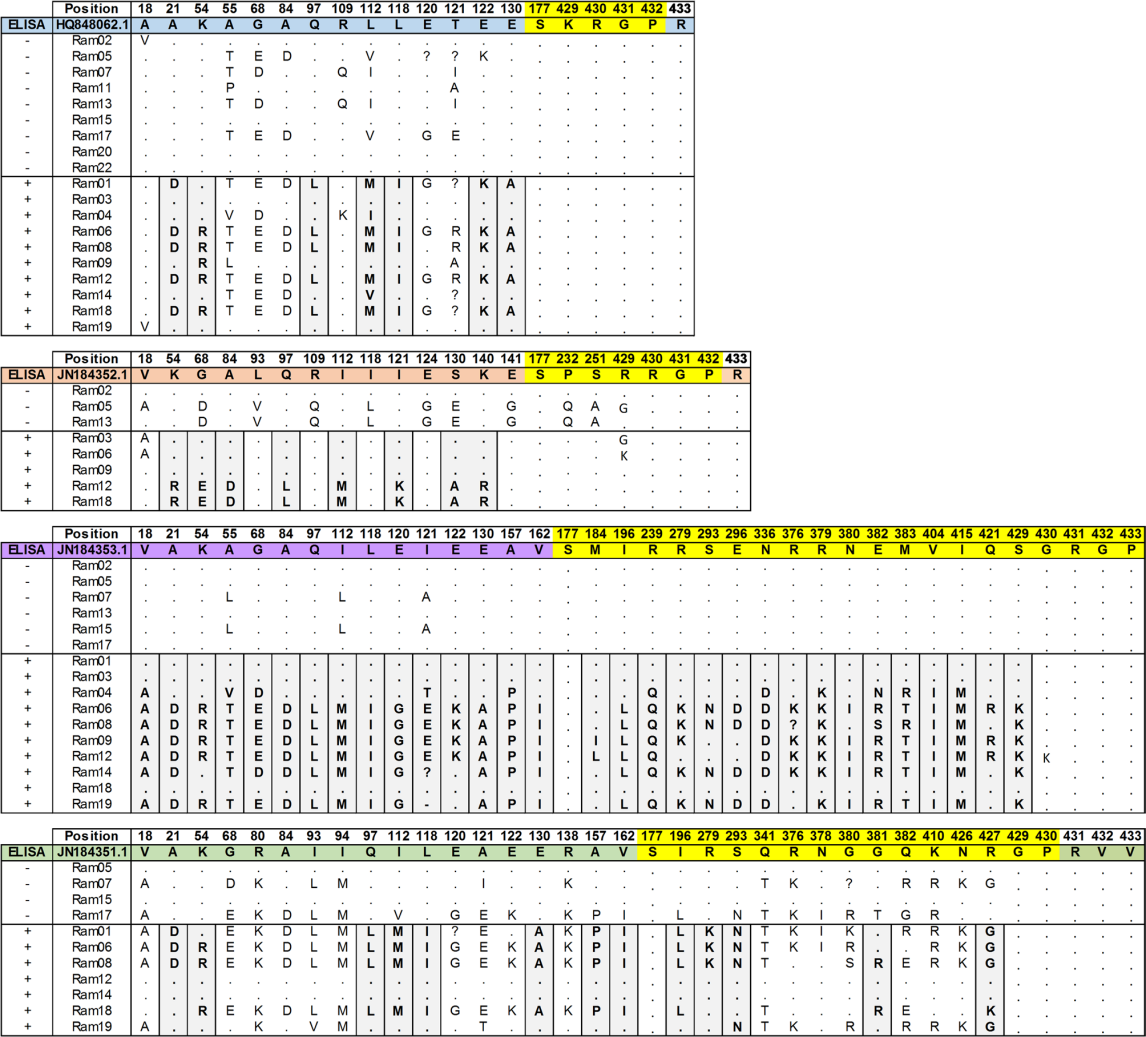
Changes in the amino acid sequences of the 22 rams under study compared to the protein sequences in the NCBI database (references HQ848062.1, JN184351.1, JN184352.1, and JN184353.1). Changes in the gag protein are indicated in blue, orange, purple and green, and those in the P25 protein are shown in yellow. ELISA-negative rams 10, 21, and 23 did not show sequences mapped against any SRLV genome/genes.

Most variants detected belong to *gag* genes HQ848062.1 (Visna/maedi virus isolate697,complete genome); JN184351.1 (Small ruminant lentivirus isolate 72 gag protein (gag) gene, complete cds); JN184352.1 (Small ruminant lentivirus isolate 160 gag protein (gag) gene, complete cds), JN184353.1 (Small ruminant lentivirus isolate 166 gag protein (gag) gene, complete cds) and JN184354.1 (Small ruminant lentivirus isolate 292 gag protein (gag) gene, complete cds), and are shown in Supplemental File S7. Figure 3 shows aminoacid changes common to the *gag* proteins detected in the 22 rams under study.

For the HQ848062.1 genome, no variants were detected in the *gag* gene fragment coding the P25 protein. However, the *gag* 166 protein, AEY84733.1, consisting of 443 aa and coded by JN184353.1 nucleotide sequence, showed the greatest number of amino acid variants between ELISA positive and negative animals in the P25 fragment. ELISA positive animals displayed 31 aminoacid substitutions compared with the reference protein sequence AEY84733.1, to which ELISA-negative animals are more similar to. In *gag* 72 (JN184351.1), although to a lesser extent, amino acid differences were also observed between ELISA positive and negative rams. For the *gag* 160 protein (JN184352.1), no clear pattern of differentiation between the two ELISA groups was observed.

If the peptide used in the ELISA test used for the diagnosis of these animals was similar to that detected for this protein in the *gag* gene JN184353.1 (gag 166), the negative diagnosis of rams 2, 5, 7, 13, 15 and 17 could partly justify the seronegativity of these animals. However, the scarcity of JN184353.1 sequences in some of the positive rams -such as Ram1 (3 sequences), Ram3 (7 sequences) and Ram18 (5 sequences)- and in most of the negative rams, does not allow definitive conclusions to be drawn.

A set of peptides from the *gag* gene designed by De Andrés and colleagues in 2013^20^ for ELISA-based diagnosis, was also compared with the P25 sequences obtained in our study. In Supplemental File S7 the mapping of these peptides against the sequences of the P25 of Rams under study is shown.

### Flow cell nanopore sequencing using GAGPOL_2 plus P-25 amplicons in blood samples from Rams of the Herd2_VQ

To assess the efficiency of the Nanopore sequencing diagnosis method using DNA isolated from blood samples and amplicons generated with GAGPOL_2 and P-25 primers in a different set of animals, a sequencing run was performed using a Flow Cell FBC34199 (Fig. 1). The run included 18 ELISA negative rams from Herd2_VQ plus a negative control (Supplemental File S3). Nanoplot metrics and results of SRLV Nanopore diagnosis in this batch of samples are shown in Supplemental Files S5 and S6. From the initial set of 18 ELISA negative rams, 8 tested positive and 10 tested negative with the Nanopore sequencing method (44.4% of false negatives). The SRLV genome most represented in this group of animals was the MVV isolate 697 (HQ848062.1). Also in one animal (Ram13_VQ) the Caprine arthritis encephalitis virus Ov496 (FJ195346.1) was detected.

### Phylogenetic analysis using SRLV consensus nanopore sequences

Phylogenetic analysis was conducted for the *gag* and *pol* genes (positions 290–3000) of the genome more frequently found in the group of Rams of this study, i.e., HQ848062.1. Alignment of the consensus sequences for these genes from the 19 Rams under study, plus 81 different *gag* and *pol* genes from various SRLV genomes downloaded from the NCBI database (Supplemental File S4), was performed using MAFFT. Subsequently, sequences were trimmed using Trimal software. Finally, a phylogenetic consensus tree was developed by the IQ-TREE2 software with 5,000 replicates.

**Fig. 4:**
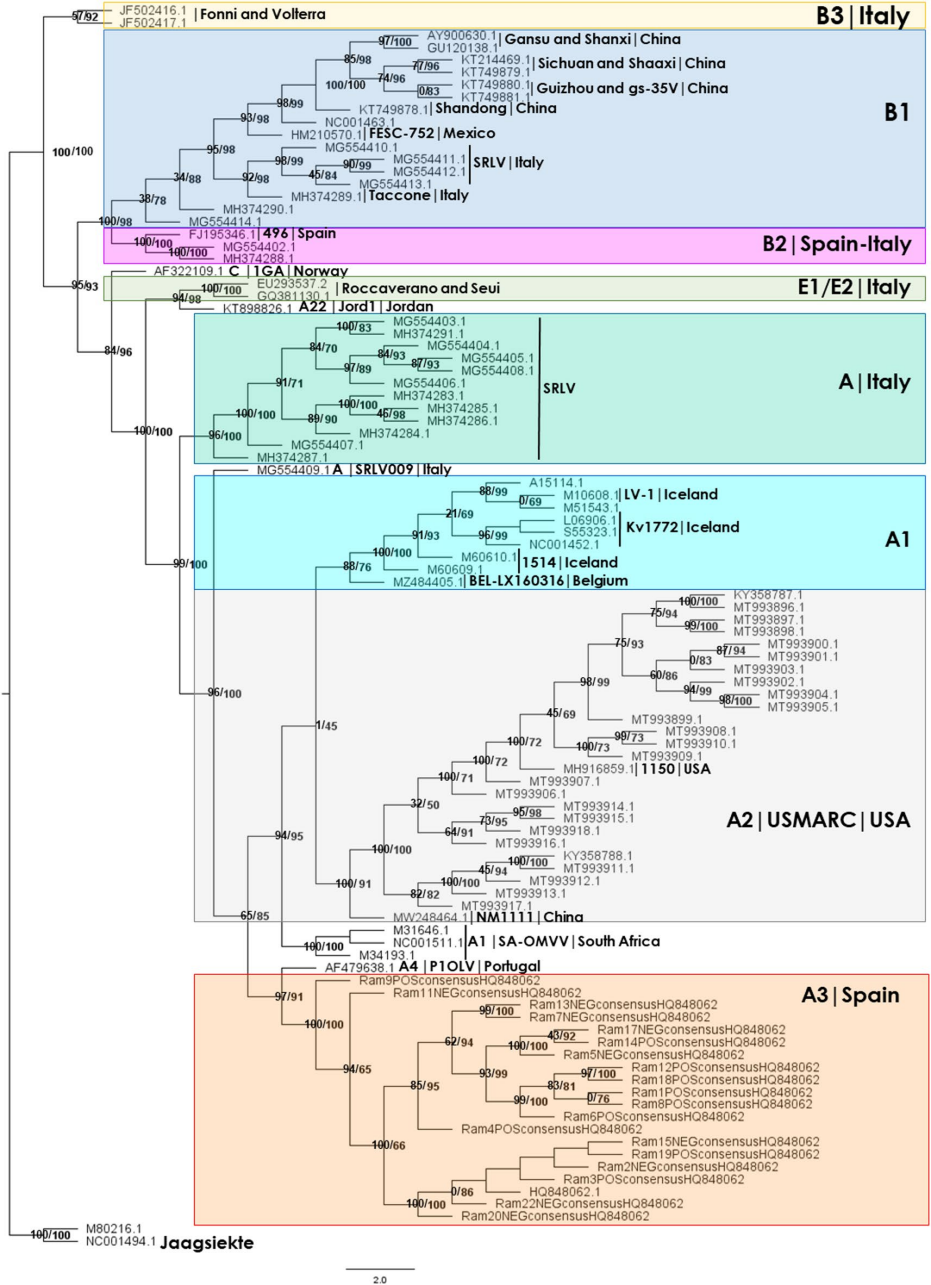
ML Phylogenetic Consensus Tree performed for the *gag* and *pol* genes consensus sequences of the 19 Rams in study mapped against the HQ848062.0 isolate 697 genome, plus the *gag* and *pol* genes of 81 SRLV genomes downloaded from NCBI (2,711 nucleotide sites). Bootstrap 5,000 replicates. Numbers in parentheses: SH-aLRT support (%)/ultrafast bootstrap support (%).

The best model found for these sequences based on the BIC value was GTR + F + R9 (BIC: 104536.735, + 95% confidence). The rate parameter (R) was, A-C: 3.4249, A-G: 8.5885, A-T: 1.2818, C-G: 1.8502, C-T: 13.1378, and G-T: 1.0000, and the state frequencies A: 0.3867, C: 0.1491, G: 0.2543, and T: 0.2100. Log-likelihood value of the consensus tree was − 51407.2925 (s.e. 876.6596). Figure 4 shows the Consensus tree. The tree was rooted in the Jaagsiekte retrovirus genomes. In Supplemental File S8 both, ML and Consensus Trees are shown.

Sequences of *gag-pol* genes of the rams under study belongs to the A3 group forming a branch alongside HQ848062.1. Five rams tested as ELISA positive were grouped in a branch with 100% bootstrap support (99.3% SH-aLRT). Some ELISA negative rams grouped with the HQ848062.1 (Visna/maedi virus isolate 697) whose *gag* and *pol* gene sequences are more similar. The phylogenetic tree agrees with that of Shi and co-workers^44^ based on the *gag* region of 79 SRLV genomes.

## Discussion

In this study, we evaluated ONT-based amplicon sequencing combined with a simplified bioinformatics pipeline as a rapid and accessible diagnostic tool for SRLV. We analyzed 22 Assaf rams from a commercial flock with a 39.5% SRLV prevalence as determined by the ID Screen^®^ MVV/CAEV Indirect ELISA. Samples were obtained from blood, PBMCs, semen, and nasal mucosa to assess the suitability of different biological matrices. Additional blood samples from 18 ELISA negative Assaf rams from another commercial farm were also analysed with the ONT-amplicon method.

Whole blood, while convenient, poses limitations because SRLV target cells -monocytes/macrophages- represent only 2–6% of leukocytes. Although SRLV presence in reproductive tissues and semen is well documented^45,46^, and transmission via embryo transfer has been described^47^, the nasal mucosa remains understudied. Despite intranasal infection being considered inefficient^48^, we included nasal samples to explore their diagnostic potential. The results of the diagnosis carried out with the different types of biological samples showed that lung samples from highly infected animals yielded a large amount of viral reads, while PBMCs samples showed no improvement over whole blood. This fact, coupled with the technical difficulty involved in isolating PBMCs, makes the use of this type of sample for the mass diagnosis of SRLVs unfeasible. In semen and nasal samples, SRLVs DNA was detected but at levels insufficient for reliable diagnosis.

Between the two PCR strategies tested, GAGPOL_2 (targeting *gag* and *pol* genes) clearly outperformed GAGPOL_1 (nested PCR targeting *pol*). The latter amplified *pol* viral sequences in only one animal, likely with a high viral load, suggesting that primer competition reduced its efficiency. With regard to the comparison of ONT diagnosis using only GAGPOL_2 primers versus the use of GAPOL_2 plus P-25 primers, the results do not differ in sequencing efficiency, but they do allow for greater refinement in terms of the infecting strain.

For blood samples, Nanopore sequencing showed a very high proportion of host and other organisms derived reads, indicating potential off-target amplification or background DNA. These non-specific amplicons frequently outcompeted viral targets, despite the use of the adaptive sampling strategy to enrich the sequencing of virus fragments. However, in the case of high infected lung samples, the proportion of mapped reads to SRLVs increases enormously, ∼73%. Flongles devices showed limited diagnostic value due to their low pore count (50–100), lack of library reloading, and inability to support the adaptive sampling approach, resulting in rapid pore depletion.

Our results suggest that the ELISA test used (ID Screen^®^ MVV/CAEV Indirect) underestimated SRLV prevalence, with approximately 42% false negatives in our experimental flock (as a result of several Nanopore runs) and 44.4% in Herd2_VQ, for which a single Nanopore run including 18 ELISA negative rams was performed. As for the comparison of the ONT-amplicon technique with diagnosis using qPCR (EXOone Maedi Visna – CAEV kit), the results indicated a false negative rate of 77% in data from our experimental flock.

Interestingly, ONT sequencing identified coinfections with CAEV-Ov496 (FJ195346.1) and MVV-isolate697 (HQ848062.1) in four rams (4, 5, 6, and 13), consistent with previous reports in Spanish sheep^49^. Such genotype-level resolution highlights an advantage of sequencing over conventional diagnostics.

The differences in the number of sequences mapped to SRLV genomes among Nanopore-positive animals may reflect varying infection levels. For example, the high number of SRLV reads observed in rams 5 and 6 could indicate a greater infection burden, whereas animals with fewer reads may carry a lower viral load. These observations suggest that Nanopore sequencing could provide added value, as it may not only detect the presence of SRLV infection but also offer insights into its relative extent.

Barcode stringency influenced diagnostic accuracy. We used a strict assignment score of 70 for both forward and reverse barcodes to minimize misclassification. Lowering this threshold (e.g., to the MinKNOW default of 60) would recover more unclassified reads but at the cost of increased false positives (Supplemental file S3). For diagnostic purposes, we recommend maintaining stringent thresholds.

Finally, based on sequencing duration and pore retention, we found that some Flow Cells maintained sufficient pores after ~ 24 h to support a second run. Thus, a practical strategy to optimize Flow Cell use and reduce costs, without compromising sensitivity, may be to perform two consecutive 14 to 16-sample runs plus a negative control per Flow Cell, rather than multiplexing ≥ 22 samples in a single run.

The low performance of Flongles for SRLV diagnosis was assessed, primarily due to their limited pore count (50–100) and lack of support for library reloading and adaptive sampling, leading to rapid pore depletion. Although Flongles are cost-effective, these limitations currently hinder their diagnostic utility.

With regard to the discrepancies between ELISA and Nanopore results, the lack of information on the exact peptide sequences used in the ELISA test hinders a comprehensive explanation. However, sequencing of the *gag*-encoded P25 protein revealed variants that differed between ELISA-positive and ELISA-negative animals. These sequence differences may contribute to ELISA detection failure in some cases. While not conclusive, since the ELISA test also incorporates TM and gp135 peptides that were not analysed in this work, our findings suggest that specific P25 variants could be associated with serological detection in some animals but failure in others.

A possible drawback when comparing the diagnostic results obtained by Nanopores and the previous ELISA tests is the time lapse between the two tests, which could lead to the spread of the disease among animals serodiagnosed as negative. However, given the age of the animals, between 2.5 and 3.8 years old, and their intensive management, we consider that the opportunity for them to become infected has been very high given the prevalence of the disease in the herd, and that therefore their health status should not have changed. Another weakness of the study is the small sample size used, a total of 40 animals (all male), which are also of the same breed and come from only two herds. We believe that the proposed method is robust, but that it should be confirmed with a larger number of animals and a representative collection of strains circulating in different regions and countries.

## Conclusions

Nanopore sequencing of PCR amplicons from SRLV genomic regions demonstrated high sensitivity for detecting these viruses in whole blood samples. Compared to conventional diagnostics such as ELISA and qPCR, which rely on highly specific short target sequences (15–20 amino acid peptides in ELISA and 20–25 nucleotide probes in qPCR), ONT sequencing appeared more robust and informative. Beyond distinguishing infected from uninfected animals, it enabled genotype-level characterization, offering insights into viral virulence and host resistance or susceptibility to specific strains.

The main limitations of ONT-based SRLV diagnosis in blood samples are the restricted availability of target cells, given the tropism of SRLVs for monocyte/macrophage lineages (a small fraction of leukocytes), and the co-amplification of host and non-viral DNA, which competes with viral DNA fragments during sequencing. These drawbacks could be mitigated by improved viral DNA extraction protocols and the design of multiple PCR primer sets targeting different viral genomic regions to enhance detection efficiency.

Taken together, our results highlight ONT amplicon sequencing as a powerful tool for SRLV diagnosis, capable of overcoming key limitations of current serological and molecular assays while providing valuable genotypic information relevant to disease control and breeding programs. Beyond enabling early detection and the discrimination of maternal antibody transfer from true infection, this approach may also offer insights into host resistance or susceptibility to lentiviral diseases, which could be leveraged in selective breeding programs to generate SRLV-resistant animals.

## Methods

### Ethical statement

The biological samples used in this study were collected by licensed veterinarians from the breeders’ association as part of routine health management and diagnostic procedures. No samples were collected specifically for research purposes, and no additional interventions, handling, or procedures were performed on the animals beyond normal veterinary practice. According to the European Directive 2010/63/EU and its national transposition in Spain (Real Decreto 53/2013), procedures carried out solely for veterinary clinical or diagnostic purposes, and which do not cause additional pain, suffering, distress, or lasting harm to the animals, are not considered scientific procedures and therefore do not require approval from an animal ethics committee.

### Animal sampling

Biological samples were collected from a sheep farm located in Castilla y León, Spain, in April 2023. All animals on the farm, born between 2015 and 2022, had been previously tested for MVV/CAEV infection using an indirect ELISA assay (ID Screen MVV/CAEV Indirect, Innovative Diagnostics, Grabels, France. https://www.innovative-diagnostics.com/produit/id-screen-mvv-caev-indirect). This ELISA detects antibodies against a panel of peptides derived from TM, gp135, and p25 proteins of SRLVs, allowing for the detection of genotypes A, B, and E with high specificity and sensitivity. The overall seroprevalence in the flock was 39.5%, with 1,480 animals testing positive out of a total of 3,750.

On April 18, 2023, blood, nasal mucosa, and semen samples were collected from 23 rams born in 2020 (one animal, Ram16, was discarded for future analyses since failure of semen sample collection). Based on the ELISA results obtained at November 2022, 10 of these rams were seropositive and 13 were seronegative. Rams ranged in age from 2.5 to 3.8 years. Although SRLVs are primarily known target monocytes in peripheral blood, additional sampling of semen and nasal mucosa was conducted to explore their potential utility for molecular diagnosis, and to investigate their possible roles in sexual and airborne transmission. A second blood sampling was performed on May 9, 2024, from eight of the initially sampled rams (4 ELISA positive and 4 ELISA negative), due to the discrepancies found in the Nanopore diagnosis of these animals by using different set of primers (see also Supplemental File S1).

Additionally, blood samples from 22 rams belonging to a different herd (Herd2_VQ), also located in Castilla y León (Spain), which were diagnosed with the same ELISA test in 2023 and 2024 (4 ELISA positive and 18 ELISA negative), were collected in July 2024 (Supplemental File S1).

### Sample collection and processing

Blood was drawn from the jugular vein using EDTA-coated Vacutainer tubes. Whole blood samples were aliquoted into cryovials and immediately frozen in liquid nitrogen. Semen was collected via electro-ejaculation, and all collection equipment was thoroughly disinfected between animals using povidone-iodine followed by ethanol to prevent cross-contamination. Nasal mucosa was sampled using sterile dual-swab tubes (BD Culture Swab, Becton Dickinson, Sparks, MD, USA) without the use of transport medium. Swabs were transported on dry ice and stored at − 80 °C until nucleic acid extraction. Semen collection failed for one ram (Ram 16), whose sample was excluded from downstream analyses.

### White blood cell isolation

Due to the successive culling of some animals included in the study, peripheral blood mononuclear cells (PBMCs) could only be isolated from 15 rams (7 ELISA-positive and 8 ELISA-negative). On September 9, 2024, 4 mL of whole blood were collected from these animals to compare the diagnostic performance of DNA extracted from whole blood versus isolated white blood cells (see Supplemental File S1).

PBMCs were isolated from EDTA-treated whole blood by density gradient centrifugation using Lymphoprep (Stemcell Technologies, Canada) in combination with Leucoseptubes (Greiner Bio-One, Germany), which contain a polyethylene porous barrier. Centrifugation was carried out using a swinging bucket rotor with the brake function disabled. The complete isolation protocol is provided in Supplemental File S2.

### DNA extraction

Genomic DNA was extracted from: 300 µL of whole blood, 300 µL of isolated PBMCs, and 100 µL of semen, using the ZymoBIOMICS DNA Miniprep Kit (Zymo Research Corporation, CA, USA), following the manufacturer’s protocol.

For nasal swab samples, the tips were aseptically cut and transferred to individual 1.5 mL microcentrifuge tubes. DNA was extracted using the MagMAX DNA Multi-Sample Ultra 2.0 Kit (Thermo Fisher Scientific, DE, USA), according to the manufacturer’s instructions. All DNA samples were eluted in 80 µL of elution buffer.

DNA concentration was measured using a Qubit 4 Fluorometer (Thermo Fisher Scientific), and purity ratios (A260/280 and A260/230) were assessed using a NanoDrop 2000 Spectrophotometer (Thermo Fisher Scientific, DE, USA).

### Additional samples

DNA samples from lung tissue of six adult sheep (three SRLV-positive and three SRLV-negative by qPCR) were also included for comparison. These samples were obtained from a certified diagnostic laboratory (EXOPOL, Spain, https://www.exopol.com/es/) using the EXOone Maedi Visna – CAEV qPCR kit, which targets the 5′LTR and *gag* regions of the viral genome using TaqMan probes^5^. Details of all samples used are provided in Supplemental File S1.

### SLRV detection by qPCR

DNA from the whole blood of the 22 rams was analyzed for the presence of SRLV proviral DNA using a commercial quantitative PCR assay. Testing was performed at a certified Spanish veterinary diagnostics laboratory (EXOPOL, Spain, https://www.exopol.com/es/) using the EXOone Maedi Visna – CAEV oneMIX qPCR Kit^5^, which targets the 5′LTR and *gag* regions of the SRLV genome using TaqMan probes. Further details of the qPCR protocol are available in Supplemental File S2.

### End-point PCR and amplicon design

#### Target gene selection: gag, pol, and p25 protein

For SRLV detection using Nanopore sequencing, amplicons were generated by end-point PCR targeting conserved regions of the *gag*, *pol* genes and p25 protein. The *gag* and *pol* genes were selected due to their relatively conserved sequences across SRLV genotypes, making them suitable for broad-range detection. In addition, p25 was targeted to assess the correspondence between molecular detection and serological status, since it encodes a major capsid protein recognized by commercial ELISA tests (ID Screen MVV/CAEV Indirect, Innovative Diagnostics).

To identify conserved motifs, the reference genome of the Visna/Maedi virus strain KV1772 (NCBI RefSeq: NC_001452.1) was aligned against over 100 *gag* and *pol* sequences from various SRLV isolates available in the NCBI database (Supplemental File S2). Alignments were performed using Clustal Omega v1.2.2^35^ and MEGA11^36^. For p25, variants were retrieved using the reference sequence DQ084338.1 and aligned similarly.

#### Primer design

Primer pairs were designed based on conserved regions identified through sequence alignments, prioritizing: amplicon sizes between ~ 750 bp and 3,000 bp; inclusion of degenerate bases to account for polymorphisms; high stability and minimal secondary structure (evaluated with NetPrimer, PREMIER Biosoft https://www.premierbiosoft.com/netprimer/); and compatibility with multiplexing and Nanopore-specific primer tailing.

Forward primers were designed with 5’ tails compatible with Oxford Nanopore library preparation protocols, allowing direct sequencing of PCR products. For *gag* amplification, we additionally employed a modified validated primer pair previously described by^37^: Forward: 5′-GGGACGCCTGAAGTAAGGTAAG-3′; Reverse (degenerate): 5′-CT**Y**CAAAATCCTCGGACACAAG-3′.

For p25, three degenerate primer pairs were designed based on alignment of known variants to ensure coverage across genotypes (Supplemental File S2). In this case, the ONT-specific tails were not added to the primers.

The size of the amplicons generated by each pair of primers used is shown in Supplementary File S2.

#### PCR conditions

PCR conditions and cycling parameters, including enzyme system, reaction volumes, and thermal profiles, are provided in detail in Supplemental File S2. Amplicons were visualized on agarose gels to confirm expected sizes before sequencing.

### SLRV detection by nanopore sequencing

#### Library Preparation and nanopore sequencing

Sequencing of PCR amplicons targeting the *gag* and *pol* genes of SRLV, obtained from blood, semen, and nasal mucosa samples of 22 rams, was performed using Oxford Nanopore Technologies (ONT) Nanopore long read sequencing on a GridION X5 Mk1 sequencer (GXB03398) with R10.4.1 flow cells (FLO-MIN114). For each biological sample type—blood, semen, and nasal mucosa—two sequencing replicates were conducted (a total of four sequencing runs), each including samples from 11 rams and one negative control. Replicates differed by the primer sets used: replicate 1 employed the GAGPOL_1 primers for *gag* (744 bp) and *pol* (1175 bp), while replicate 2 used GAGPOL_2 primers for *gag* (744 bp) and *pol* (903 bp) (see primer details in Supplemental File S2). A third sequencing run using GAGPOL_2 amplicons from the 22 blood samples was also conducted on a separate flow cell.

Additionally, blood samples were sequenced using Flongle R10.4.1 flow cells (FLO-FLG114) with varying numbers of samples per run. In this case, only GAGPOL_2 amplicons were used.

To assess whether isolating white blood cells improved diagnostic sensitivity, two additional sequencing runs were conducted using GAGPOL_2 amplicons from DNA extracted from PBMCs (lymphocytes and monocytes) of 15 rams (7 ELISA-positive, 8 ELISA-negative), using R10.4.1 flow cells.

To compare the Nanopore sequencing method with a certified qPCR diagnostic test (EXOone Maedi Visna – CAEV kit, EXOPOL Lab), amplicons from lung-derived DNA of four animals (2 qPCR-positive, 2 qPCR-negative) were sequenced in one flow cell run. A separate Flongle run included lung DNA from one qPCR-positive and one qPCR-negative animal, along with blood-derived amplicons from the rams. DNA extraction and qPCR of lung samples were performed by EXOPOL lab.

For GAGPOL_1 and GAGPOL_2 amplicons libraries were prepared using the Ligation Sequencing Kit SQK-LSK114 either with the Barcoding Expansion Kit EXP-PBC001 or EXP-PBC096, according to ONT protocols (estimated time: ~150 min). Details of the library preparation and sequencing protocols are provided in Supplemental File S3.

To evaluate the performance of the ELISA ID Screen MVV/CAEV Indirect test, which is based on peptides from gp135 and p25 proteins (exact peptide sequences not disclosed), five sequencing runs were conducted with R10.4.1 flow cells to sequence p25 amplicons generated from blood samples of the 22 rams. In the first two runs, three different primer pairs (Supplemental File S2) were tested to amplify the nucleotide coding region of the P25 protein of Visna/maedi virus isolate 697 (HQ848062.1)^38^. In the next three runs, amplicons of the P25 protein were sequenced with the primer pair with the best results obtained in the previous tests (HQ_P25_20F/HQ_P25_2R). In this case, libraries were performed using the Native Barcoding Kit SQK-NBD114.24 according to ONT recommendations (see Supplemental File S3 for full protocol).

#### Bioinformatic processing

Sequencing, basecalling, barcoding, and initial alignment were performed in real time using MinKNOW v24.11.8 (https://nanoporetech.com/software/devices/p2i/software/linux/history?version=24118) on the GridION device. Sequencing duration varied depending on pore availability and throughput.

For sequencing runs involving blood, white cell fractions, semen, and nasal mucosa, ONT’s Adaptive Sampling strategy was employed to enrich for target SRLV DNA sequences. This approach utilized a reference panel of 5,876 complete SRLV genomes and genomic regions from NCBI (listed in Supplemental File S4). Adaptive Sampling was not applied to Flongle runs or p25 gene sequencing, in accordance with ONT recommendations.

Raw fast5 reads were basecalled using the Guppy v6.4.6 (https://nanoporetech.com/software/other/guppy/history?version=6-4-6) super-accuracy model (Q ≥ 10), producing fastq files. Reads were demultiplexed with dual-barcode trimming (minimum barcode score = 70). Read alignment to the reference genome panel (Supplemental File S4) was performed using Minimap2 (https://github.com/lh3/minimap2)^39^. Reads were filtered using stringent quality and alignment criteria: mean read quality: Q ≥ 15 (97% base assignment precision); mapping quality: ≥ 30 (0.1% probability of error); alignment identity: ≥ 85%; alignment coverage: ≥ 85%; and minimum aligned length: 600 bp.

Read quality and sequencing performance metrics were assessed using NanoPlot v1.38.1 (https://github.com/wdecoster/NanoPlot)^40^ (see Supplemental File S5).

### Variant calling

Detection of variants within p25 amplicons was carried out using the ONT Epi2me Nextflow workflow wf-amplicon v1.1.3 (https://github.com/epi2me-labs/wf-amplicon), which implements the Medaka variant caller (2018- Oxford Nanopore Technologies Ltd). The following parameters were applied: min_length: 600; max_length: 3,000; min_read_qual: 15; min_mean_depth: 30; and primary_alignments_threshold: 0.7.

### Phylogenetic analysis

Consensus SRLV sequences of the *gag* and *pol* genes from the rams under study were generated from high-confidence reads (mean quality Q ≥ 15; mapping quality ≥ 30; alignment identity ≥ 85%; coverage ≥ 85%) using the wf_amplicon software. These sequences together with those of 81 SRLV genomes downloaded from the NCBI database were aligned using MAFFTv7.525 (https://mafft.cbrc.jp/alignment/software/)^41^. Alignments were trimmed with Trimalv1.4.1 (https://github.com/inab/trimal/releases)^42^. The phylogenetic tree was constructed by using IQ-TREE v2.1.3 (https://github.com/iqtree/iqtree2) with 5,000 replicates^43^.

## Supplementary Information

Below is the link to the electronic supplementary material.

Supplementary Material 1

Supplementary Material 2

Supplementary Material 3

Supplementary Material 4

Supplementary Material 5

Supplementary Material 6

Supplementary Material 7

Supplementary Material 8

## Data Availability

The datasets generated and analysed during the current study are available in the ENA European Nucleotide Archive repository, https://www.ebi.ac.uk/ena/browser/text-search? query=PRJEB98261.
