# Supplementary material for: Amplicon sequencing with Oxford nanopore technologies as a diagnostic alternative for small ruminant lentiviruses in sheep
Source: Sci Rep. 2026 Jan 25;16:6212. doi: 10.1038/s41598-026-36989-y (PMC12905341; doi:10.1038/s41598-026-36989-y)
Supplement: Supplementary file 2 — Supplementary Material 2 [file 41598_2026_36989_MOESM2_ESM.pdf]

EXOone Maedi Visna - CAEV oneMIX qPCR kit

SRLV_MIX

1. Intended use

EXOone Maedi Visna - CAEV oneMIX kit enables real-time PCR specific detection and/or quantification of Maedi Visna Virus / CAEV, based on fluorogenic hydrolysis probe chemistry. It is suitable for the analysis of lungs and nasopharyngeal secretions, milk and synovial fluids from Ovine, Caprine; and strains/vaccines samples by using a QuantStudio 5 Dx system from Applied Biosystems, CFX96™ Real-Time PCR System from Bio-Rad or another Real Time PCR thermocycler system.

This kit provides every component of the real-time PCR reaction mixed in one tube (buffers, enzyme, dNTPs, specific primers and probes for the pathogen). SRLV genome amplification will be detected through FAM channel at 530 nm. A specific synthetic positive control containing SRLV target sequence is provided for relative and absolute quantification.

2. Kit contents

Reference	Content	Presentation
SRLV oneMIX tube	Buffer, enzyme, dNTPs and primers/probes for pathogen detection	1 or 2 tubes[a] 750 µl each
RETR POSITIVE CONTROL	Lyophilized specific synthetic DNA[b]	1 tube
Water	Molecular grade water	1 tube (1ml)

[a] 1 tube for 50 reactions format and 2 tubes for 100 reactions format
[b] Reconstitute **RETR positive control** in 250 µl of molecular grade water (see 6.2)

Required but not provided in the kit

- qPCR plates/tubes and their corresponding film/caps.
- Disposable powder-less gloves.
- Set of pipettes (2 µl–1,000 µl).
- Sterile filter tips for micro-pipettes.
- Real Time PCR thermocycler.
- Desktop microcentrifuge for 1.5 ml microtubes.
- Sterile microtubes.
- Vortex mixer.

3. Storage

• EXOone Maedi Visna - CAEV oneMIX should be transported with dry ice and must be stored at ≤-18°C protected from light.

• SRLV oneMIX tube must be stored at ≤-18ºC.
Do not freeze-thaw the tube more than 5 times.

• Reconstituted Positive Control should be stored at ≤-18ºC (we recommend prepare aliquots). For quantification do not use a positive control frozen-thawed over 3 times.

• Reagents should not be used after the expiration date. See the Certificate of Analysis.

4. Warnings and precautions

• Intended use only in veterinary field with research purposes.

• Carefully read these instructions before starting the procedure.

• Clinical samples should be regarded as potentially infectious materials and should be prepared in a biosafety cabinet.

• This assay needs to be run according to Good Laboratory Practices.

• Do not use the kit after its expiration date.

• Set up at least two separate work areas:
1) Isolation of the nucleic acids.
2) Amplification/Detection area.

• Pipettes, vials and other working materials should not circulate between work areas.

• Use always sterile pipette tips with filters.

• Wear different coats and gloves in each area.

5. Sample collection, storage and transportation

• Collect samples into sterile tubes.

• Specimens should be extracted immediately or conveniently stored at ≤-18ºC.

• Transportation of clinical specimens must comply with local regulations for the transport of etiologic agents.

6. General procedures

6.1 Nucleic acid isolation

6.1.1 Samples preteatment

• MILK: individual or pooled specimens (maximum 10 samples) are required. Centrifuge pooled samples (1 ml/sample) at 3,500 x g for 10 min. or 1 ml of an individual sample for 10 min. at 13,000 x g. Collect 200 µl of the pellet avoiding milk fat and bring it to a DNAse/RNAse free microtube with 200 µl of PBS (or required volume of lysis buffer depending on your DNA extraction kit protocol). Homogenize at 6,000 rpm for 25 sec. in an automated tissue homogenizer.

• ORGANS, BIOPSIES AND TISSUES: Select a small fragment (approx. 25 mg) of tissue with individual sterile forceps and scissors for each case. Cut tissue into small pieces and put into a DNAse/RNAse free microtube with a 4 mm stainless steel bead and 200 µl of PBS (or required volume of lysis buffer depending on your DNA extraction kit protocol). Homogenize in 2 cycles of 3,000 rpm for 25 sec. in an automated tissue homogenizer.

• SWABS: Remove cells from swab with a rotary movement inside an microtube with 700 µl of PBS. Centrifuge at 13,000 x g for 5 min. Pellets can be pooled (max=5) if necessary. Pass the pellet to another microtube containing 200 µl of PBS (or required volume of lysis buffer depending on your DNA extraction kit protocol). Homogenize at 6,000 rpm for 25 sec. in an automated tissue homogenizer.

• STRAINS AND VACCINES: Centrifuge 1 ml of strain in liquid medium or vaccine sample at 13,000 x g for 10 min. Resuspend the pellet with 200 µl of PBS (or required volume of lysis buffer depending on your DNA extraction kit protocol). Homogenize at 6,000 rpm for 25 sec. in an automated tissue homogenizer.

• FLUSHINGS: Centrifuge pooled samples (up to 5 samples, 2 ml/sample) or 10 ml of single sample 3,500 x g for 10 min. Collect 200 µl of the pellet into a DNAse/RNAse free microtube with 200 µl of PBS (or required volume of lysis buffer depending on your DNA extraction kit protocol). Homogenize at 6,000 rpm for 25 sec. in an automated tissue homogenizer.

6.1.2 Extraction of nucleic acids

This qPCR kit is designed to work properly with all the extraction methods that yield high quality Nucleic Acids with minimal PCR inhibitors. Some recommended kits:

a) MANUAL NUCLEIC ACIDS EXTRACTION:
- QIAamp DNA Blood Mini Kit (QIAGEN, Inc.)
- DNeasy Blood & Tissue Kit (QIAGEN, Inc.)
- QIAamp Viral RNA Mini Kit (QIAGEN, Inc.)
- RNeasy PowerLyzer Tissue & Cells Kit (QIAGEN, Inc.)
- SpeedTools Total RNA extraction Kit (Biotools, S.A.)

b) AUTOMATED NUCLEIC ACIDS EXTRACTION:
- MagMAX CORETM Nucleic Acid Purification Kit (Thermo Fisher Scientific)
- MagNA Pure 24 Total NA Isolation Kit (Roche)

with their respectives automatic extraction robots (KingFisherTMFlex; MagNA Pure 24 System) following the manufacturer´s instructions.

6.2 Positive Control

Reconstitute the Positive Control (PC) as described below:

1. Spin briefly the PC tube and reconstitute with 250 µl of water.
2. To facilitate resuspension keep the tube for 10 min. at room temperature.
3. Vortex gently the tube before use.
4. Store reconstituted Positive Control at ≤-18ºC.

6.3 Qualitative and quantitative PCR

The kit can be used for quantitative or qualitative Real Time PCR. A specific **SRLV** positive control **(RETR)** with a determined number of copies (see CoA) is supplied with the kit.

For performance of quantitative Real Time PCR, a standard curve with ten-fold dilutions of positive control must be prepared as follows:

1. Pipette 450 µl of Molecular grade water into 5 tubes and label the tubes from 2 to 6.
2. Pipette 50 µl of **RETR** positive control into tube 2.
3. Vortex thoroughly.

4. Change pipette tip and pipette 50 µl from tube 2 into tube 3.

5. Vortex thoroughly.
6. Complete the following dilutions as indicated in steps 4 and 5.
7. Pipette 5 µl of each dilution into each well, according to your protocol plate set up.

Two or three replicates of each dilution are recommended in order to get a proper quantification analysis data. For quantification do not use a positive control frozen-thawed over 3 times. Standard curve is not needed to perform qualitative Real Time PCR.

6.4 PCR set-up

1. Thaw the oneMIX tube you need regarding the number of required reactions. Non used oneMIX tube must remain frozen.
2. Vortex the tube briefly.
3. Dispense 15 µl of oneMIX into each well/tube used for the assay.
4. Add 5 µl of nucleic acid sample or PC or negative control (water) in the corresponding wells/tube used for the assay.
5. Seal the wells/tubes with their corresponding film or caps.
6. Centrifuge the plate/tubes gently before inserting it into the thermocycler to prevent drops in the well pit walls.

6.5 Thermal profile

Cycles	Time	Action	Temperature
1 cycle	5 min	Enzyme activation	95ºC
42 cycles	15 sec	Denaturation	95ºC
	60 sec	Annealing	60ºC
		Data collection[c]	

Data collection: Fluorescence data should be collected during this step through FAM channel (SRLV>).

Thermal profiles of EXOone qPCR and RT-qPCR kits in oneMIX and multiplex formats are compatible and they can be run together using the RT-qPCR protocol. **Do not use ROX as passive reference.**

7. Data analysis and interpretation

SAMPLE[d] (FAM)	PC (FAM)	NC (FAM)	ASSAY RESULT
+	+	-	valid
-	+	-	valid
+ / -	-	-	not valid
+ / -	+	+	not valid

PC: Positive control<br>NC: Negative control<br>EC: Endogenous control	+ : positive<br>- : negative<br>+ / - : positive/negative

[d] a sample is considered as positive if **Cq ≤ 38**. For strains/microbiological isolates, a sample is considered positive if **Cq ≤ 30**.

8. Validation summary

a) A panel of 17 positive SLRV samples of different genotypes A (n=5), B (n=11) and E (n=1), resulted positive.

b) A total of 26 microorganisms including related pathogens and possible enviromental contaminants such as JSRV, *Mycoplasma ovipneumoniae* and *Pasteurella multocida* were evaluated as an specificity panel. All of them resulted negative.

c) A panel of 305 clinical cases from sheep (n=228) and goat (n=77) with respiratory signs and lentiviral infection suspicion were evaluated with this kit. Samples included lungs (n=150), bronchial alveolar lavage fluid (n=104), nasal swabs (n=24), articular fluid (n=10) and milk (n=17). Maedi Visna-CAEV was detected in 23% (71/305) of these samples, mainly in milk (65% of positives samples), articular fluid (30%) and lungs (26%).

- We studied the reportable range for this qPCR assay using a purified specific synthetic oligonucleotide (positive control).

- This qPCR kit can identify **SRLV** from clinical samples.

- Maximum quantification limit of **SRLV** ≥ 10^9 copies/reaction.
- Minimum quantification limit of **SRLV** = at least 100 copies/reaction.

White series cells isolation

Separation of lymphocytes and peripheral mononuclear cells from sheep whole blood (with EDTA anticoagulant) was performed by means of density gradient centrifugation with Lymphoprep™ medium (Stemcell Technologies., Canada) and Leucosep ™ tubes (Greiner bion-one, Germany) which incorporate a porous barrier of polyethylene. Centrifugation was done in a swinging bucket rotor and with switch off brake.

Preparation:

- Fill the 12 ml Leucosep™ tube (ref. 163289) with 3ml of separation medium Lymphoprep™ with screw-cap and centrifuge for 1 minute at 2200 rpm and at r.t. Lymphoprep™ is located below the porous barrier.
- The tube is ready for filling with dilution of 4ml of blood with 4ml Dulbecco's PBS with 2% FBS. Centrifuge 15 minutes at 2000 rpm and at r.t.
- Sequence of layers after centrifugation: plasma, lymphocytes, separation medium, porous barrier, separation medium and pellet of erythrocytes and granulocytes.
- Harvest the lymphocytes/PBMCs by means of pipette removing plasma.
- Wash lymphocyte/PBMCs with 10ml Dulbecco's PBS with 2% FBS and centrifuge for 10 minutes at 1100rpm at r.t.
- Repeat washing step twice with 5ml Dulbecco's PBS with 2% FBS and centrifuge for 10 minutes at 1100rpm at r.t.
- Harvest the pellet of lymphocyte/PBMCs in a 2ml eppendorf tube.

PRIMERS FOR ENT-TIME PCR

in blue 5' PBC tail

Primers
GAGPOL_1 primers
**PBC-NEIGAGXL-F Amplicon size ~ 744bp**
5’ ttt ctg ttg gtg ctg ata ttg cGG GAC GCC TGA AGT AAG GTA AG 3’
**PBC-NEIGAGXL-R**
5’ act tgc ctg tcg ctc tat ctt cCT YTC AAA ATC CTC GGA CAC AAG 3’
**MAPOLEX-F Amplicon size ~ 1402bp**
5’ CA TGA RGA RGG GAC MAA TCA RCA 3’
**MAPOLEX-R**
5’ CC CAV ART ACC TGT GTT GGY CC 3’
**PBC-MAPOLIN-F Amplicon size ~ 1175bp**
5’ ttt ctg ttg gtg ctg ata ttg cGG TGC CWG GAC AYA ARG GGA TTC 3’
**PBC-MAPOLIN-R**
5’ act tgc ctg tcg ctc tat ctt cGC CAC TCK CCT GGA TGY CCT CT 3’
GAGPOL_2 primers
**PBC-NEIGAGXL-F Amplicon size ~ 744bp**
5’ ttt ctg ttg gtg ctg ata ttg cGG GAC GCC TGA AGT AAG GTA AG 3’
**PBC-NEIGAGXL-R**
5’ act tgc ctg tcg ctc tat ctt cCT YTC AAA ATC CTC GGA CAC AAG 3’
**PBC-POLSEQ1-F Amplicon size ~ 903bp**
5’ ttt ctg ttg gtg ctg ata ttg cGT GTG STG CCG TCY G 3’
**PBC-POL900-R**
5’ act tgc ctg tcg ctc tat ctt cTR RTC ACT BCC TAT RTA RAT ATC ATC C 3’
P25 primmers
**HQ_P25_20-F Amplicon size ~ 1286bp**
5’ RRG GGA TAC CCC GAG CTS 3’
**HQ_P25_300-F Amplicon size ~ 967bp**
5’ RRG GGM TAC AAG AAR NAG AAG ARG 3’
**HQ_P25_6G_400-F Amplicon size ~ 863bp**
5’ GGG GGG AGA RRT KGG R 3’
**HQ_P25_5G_400-F Amplicon size ~ 863bp**
5’ GGG GG A GAR RTK GGR 3’
**HQ_P25_2-R**
5’ GCR GAC GGC ASC ACA C 3’

<u>End Time PCR performance for *GAG* and *POL* amplicons</u>

Optimization of multiplex-nested PCR (GAG_POL_1) and multiplex PCR (GAG_POL_2)

Primers optimization and PCR stock preparation including negative controls in the master mix area are a critical steps to sequencing library preparation. The PCR conditions and primers screening are based on the specificity and concentration of the PCR products determined by agarose gel electrophoresis and subsequent Nanopore sequencing test.

The primers used in multiplex PCR did not work equimolarly, probably due to different affinity of degenerated primers and the length of the 5' PBC tail primers (37bp to 45bp). To achieve efficient PCR amplification of certain regions adjusting the concentration of the primers and increasing the amount of DNA was necessary, taking into account that the coverage depends on the provirus abundance in the cells.

PCR performance

Multiplex-nested PCR master mix consisted on: Buffer 1x, Cl2Mg 1,5 mM, dNTPs 200µM, MAPOLEX primers 0.2 µM, PBC_MAPOLIN primers 0.3 µM, PBC_NEIGAGXL primers 0.25 µM 5U/µl Hotsplit Polymerase (Biotools, Spain) and 500-600ng per reaction in a total volume of 25 µl. PCR cycles: An initial denaturation of 6 min at 94°C, 34 cycles with denaturation of 30 seconds at 94ºC, annealing of 1 min at 61°C, extension of 1min and 30 seconds at 72°C, a final extension of 10 min at 72°C and hold at 4ºC.

Multiplex PCR master mix consisted on: Buffer 1x, Cl2Mg 2mM, dNTPs 200µM, PBC_POL primers (PBC-POLSEQ1 and PBC-POL900) 0.5 µM, PBC_NEIGAGXL primers 0.125 to 0.25 µM, 5U/µl Hotsplit Polymerase (Biotools, Spain) and 500-600ng per reaction in a total volume of 25 or 50 µl. PCR cycles: An initial denaturation of 6 min at 94°C, 34 cycles with denaturation of 45 seconds at 94ºC, annealing of 1 min at 62°C, extension of 1 min at 72°C, a final extension of 10 min at 72°C and hold at 4ºC.

First PCR clean up

Treatment with 2 µL of ExoSAP-IT Express reagent (Applied Biosystems) per 5 µL of PCR product at 37°C for 4 minutes followed by an incubation period at 80°C for 1 minute for enzyme inactivation. Protocol fine-tuning we changed the amount of PCR product to be treated depending on its concentration measured in Qubit™ fluorometer with Qubit™ ds DNA BR or HS Assay kits (Thermo Fisher Scientific, DE, USA).

SRLV genomes used to design *gag* and *pol* genes primers for the End Time PCR

- **NC_001452.1** Visna/Maedi virus strain kv1772, complete genome

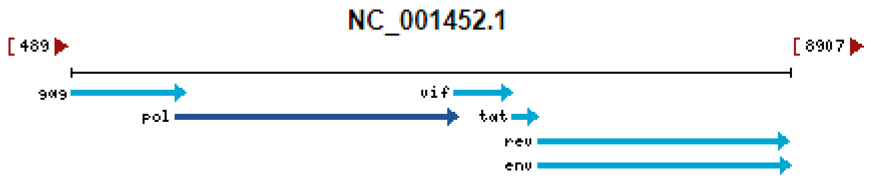

Braun MJ, Clements JE, Gonda MA. The visna virus genome: evidence for a hypervariable site in the env gene and sequence homology among lentivirus envelope proteins. J Virol. 1987 Dec;61(12):4046-54. doi: 10.1128/JVI.61.12.4046-4054.1987

GAG gene

>NC_001452.1 Visna/Maedi virus strain kv1772, complete genome

```
ATGGCGAAGCAAGGCTCAAAGGAGAAAAAGGGATACCCCGAGCTCAAGGAAGTAATTAAAGCAACTTGTA
AAATAAGGGTAGGGCCCGGGAAGGAGACCTTGACAGAAGGGAATTGTCTATGGGCATTAAAAACTATAGA
CTTTATATTTGAGGATTTAAAAACAGAGCCGTGGACGATTACAAAAATGTATACAGTATGGGATAGATTA
AAAGGGCTAACTCCGGAGGAAACAAGCAAAAGAGAATTCGCCTCCTTGCAAGCTACGTTGGCTTGCATAA
TGTGTAGTCAAATGGGCATGAAGCCCGAGACAGTGCAGGCAGCAAAGGGAATAATAAGTATGAAAGAAGG
ACTACACGAAAATAAGGAGGCCAAGGGGGAGAAGGTAGAGCAACTCTACCCCAACTTAGAGAAACATAGG
GAAGTTTATCCTATTGTGAATTTGCAAGCAGGAGGGAGAAGTTGGAAGGCGGTAGAGTCAGTAGTCTTCC
AGCAACTGCAAACAGTGGCAATGCAGCATGGACTTGTGTCCGAGGATTTTGAGAGGCAATTGGCATATTA
TGCTACTACCTGGACTAGTAAAGATATATTAGAAGTATTGGCTATGATGCCTGGGAATAGAGCACAGAAG
GAATTAATACAAGGAAAATTAAATGAAGAAGCAGAAAGGTGGGTAAGACAAAATCCACCCGGGCCGAATG
TCCTCACGGTGGATCAAATAATGGGAGTGGGACAAACCAATCAGCAGGCATCTCAAGCCAATATGGATCA
GGCAAGACAGATATGCCTGCAGTGGGTAATAACAGCGTTAAGATCAGTGAGGCATATGTCACATAGACCA
GGAAACCCTATGTTAGTGAAGCAGAAGAATACTGAGAGTTATGAAGACTTCATAGCTCGCCTACTAGAGG
CTATTGATGCGGAACCAGTGACGGACCCTATAAAAACATATTTAAAAGTAACATTGTCATATACAAATGC
TAGCACAGACTGTCAAAAGCAGATGGATAGGACATTGGGGACGAGGGTTCAACAAGCAACGGTAGAAGAA
AAAATGCAAGCATGTCGAGATGTGGGATCCGAAGGATTTAAGATGCAATTATTAGCACAAGCTTTGAGAC
CGCAAGGGAAGGCAGGACAAAAAGGGGTAAATCAAAAGTGTTATAATTGTGGGAAACCAGGACATCTCGC
AAGACAGTGTAGACAAGGAATAAT
```

```
>KY358788.1 Small ruminant lentivirus isolate USMARC-199906011-2,
complete genome
ATGGCGAAGCAAGGCTCAAAGGAGAAAAAGGGATACCCCGAGCTCAAAGAAGTAATTAAAGCA
ACATGTAAAATAAAAGTAGGGGCCGGGAAGGAGACCTTGACAGAAGGGAACTGTCTATGGGCATTAAAAA
CTGTAGACTTTATATTTGAGGATATAAAAACAGAACCGTGGACTCTTACAAAGATGTATACAGTATGGGG
AAGATTAAAGCAGTTAACTCCAGAGGAAACAAGTAAAAGAGAGTTTGCCTCCTTACAAGCTACAATGGCT
TGCATAATGTGTAGTCAAATGGGCATGAAGCCTGAGACAGTGCAGGCAGCACGGGGAATAATAAGTATGA
AAGAGGGGCTACGAGAAAATAAGGAGGACAAAGAAGGGGAGGTAGAGCAACTCTACCCAAATCTAGAGAA
GCACAAGGAAGTATACCCCATTGTGAATCTGCAAGCGGGGGGAAGGAGTTGGAAGGCAGTAGATTCTGTG
GTCTTCCAACAGCTGCAAAATGTAGCAATGCAGCATGGACTTGTGTCCGAGGATTTTGAGAGGCAAATAG
CATATTATGCCACCACGTGGACAAGTAAGGATATATTAGAAGTATTGGCTATGATGCCTGGAAACAGAGC
ACAGAAAGAATTAATACAAGGGAAATTAAATGAAGAAGCAGAAAGGTGGGTAAGACAGAATCCACCGGGG
CCAAATGTCCTTACAGTGGATCAAATCATGGGAGTGGGACAGACAAATCATCAGGCATCACAAGCCAATA
TGGATCAAGCAAGGCAAATATGTTTGCAATGGGTAATATCAGCATTAAGATCAGTAAGGCATATGTCCCA
TAGACCAGGAAACCCAATGTTAGTAAAGCAAAAGAACAGTGAAAGCTATGAAGATTTTATAGCCAGGCTA
TTAGAAGCCATAGATGCTGAGCCAGTAACAGACCCTATAAAGACATATCTGAAAGTAACTCTGTCATATA
CAAATGCAAGCACAGACTGTCAAAAACAAATGGACAGAGTATTAGGAACACGGGTACAACAAGCCTCAGT
AGAAGAAAAAATGCAAGCCTGTAGAGATGTAGGATCAGAAGGGTTCAAAATGCAATTATTAGCACAAGCT
```

TTAAGACCAGAAAGGAATTCAGGAAATCGGGGAACAGGACAAAAATGTTATAATTGTGGAAAACCGGGAC
ATTTGGCAAGACAATGCAGGCAAGGCATAATATGCCATCATTGTGGAAAAAGGGGACATATGCAAAAAGA
TTGTAGACAAAAGAAGAAAAATGATATAAAACAGCAGGGAAACAGCAGGAGGGGGCCACGTGTGGTGCCG
TCCGCGCCCCCTATGTTGTAA

>MT993911.1 Small ruminant lentivirus isolate USMARC-199906011-2, complete genome
ATGGCGAAGCAAGGCTCAAAGGAGAAAAAGGGATACCCCGAGCTCAAAGAAGTAATTAAA
GCAACATGTAAAATAAAAGTAGGGGCCGGGAAGGAGACCTTGACAGAAGGGAACTGTCTATGGGCATTAA
AAACTGTAGACTTTATATTTGAGGATATAAAAACAGAACCGTGGACTCTTACAAAGATGTATACAGTATG
GGGAAGATTAAAGCAGTTAACTCCAGAGGAAACAAGTAAAAGAGAGTTTGCCTCCTTACAAGCTACAATG
GCTTGCATAATGTGTAGTCAAATGGGCATGAAGCCTGAGACAGTGCAGGCAGCACGGGGAATAATAAGTA
TGAAAGAGGGGCTACGAGAAAATAAGGAGGACAAAGAAGGGGAGGTAGAGCAACTCTACCCAAATCTAGA
GAAGCACAAGGAAGTATACCCCATTGTGAATCTGCAAGCGGGGGGAAGGAGTTGGAAGGCAGTAGATTCT
GTGGTCTTCCAACAGCTGCAAAATGTAGCAATGCAGCATGGACTTGTGTCCGAGGATTTTGAGAGGCAAA
TAGCATATTATGCCACCACGTGGACAAGTAAGGATATATTAGAAGTATTGGCTATGATGCCTGGAAACAG
AGCACAGAAAGAATTAATACAAGGGAAATTAAATGAAGAAGCAGAAAGGTGGGTAAGACAGAATCCACCG
GGGCCAAATGTCCTTACAGTGGATCAAATCATGGGAGTGGGACAGACAAATCATCAGGCATCACAAGCCA
ATATGGATCAAGCAAGGCAAATATGTTTGCAATGGGTAATATCAGCATTAAGATCAGTAAGGCATATGTC
CCATAGACCAGGAAACCCAATGTTAGTAAAGCAAAAGAACAGTGAAAGCTATGAAGATTTTATAGCCAGG
CTATTAGAAGCCATAGATGCTGAGCCAGTAACAGACCCTATAAAGACATATCTGAAAGTAACTCTGTCAT
ATACAAATGCAAGCACAGACTGTCAAAAACAAATGGACAGAGTATTAGGAACACGGGTACAACAAGCCTC
AGTAGAAGAAAAAATGCAAGCCTGTAGAGATGTAGGATCAGAAGGGTTCAAAATGCAATTATTAGCACAA
GCTTTAAGACCAGAAAGGAATTCAGGAAATCGGGGAACAGGACAAAAATGTTATAATTGTGGAAAACCGG
GACATTTGGCAAGACAATGCAGGCAAGGCATAATATGCCATCATTGTGGAAAAAGGGGACATATGCAAAA
AGATTGTAGACAAAAGAAGAAAAATGATATAAAACAGCAGGGAAACAGCAGGAGGGGGCCACGTGTGGTG
CCGTCCGCGCCCCCTATGTTGTAA

>MT993912.1 Small ruminant lentivirus isolate USMARC-200106932-2, complete genome
ATGGCGAAGCAAGGCTCAAAGGAGAAAAAGGGATACCCCGAGCTCAAAGAAGTAATTAA
AGCAACATGTAAAATAAAAGTAGGGGCCGGGAAGGAGACCTTGACAGAAGGGAACTGTCTATGGGCATTG
AAAACTGTAGACTTTATATTTGAGGATATAAAAACAGAACCGTGGACTCTTACAAAGATGTATACAGTAT
GGGGAAGATTAAAGCAGTTAACTCCAGAGGAAACAAGTAAAAGAGAGTTTGCCTCCTTACAAGCTACAAT
GGCTTGCATAATGTGTAGTCAAATGGGCATGAAGCCTGAGACAGTGCAGGCAGCACAGGGAATAATAAGT
ATGAAAGACGGACTACGAGACAATAAGGAGGACAAGGAAGGGGAGGTAGAACAGCTCTACCCAAATCTAG
AGAAGCACAAGGAAGTATACCCCATTGTGAATCTGCAAGCGGGGGGAAGGAGTTGGAAGGCAGTAGATTC
TGTGGTCTTCCAACAGCTGCAAAATGTAGCGATGCAGCATGGACTTGTGTCCGAGGATTTTGAGAGGCAA
ATAGCATATTATGCCACCACGTGGACAAGTAAGGATATATTAGAAGTATTGGCTATGATGCCTGGGAACA
GAGCACAGAAAGAATTAATACAAGGGAAATTAAATGAAGAAGCAGAAAGGTGGGTAAGACAGAATCCACC
GGGGCCAAATGTCCTTACAGTGGATCAAATAATGGGAGTGGGACAGACAAATTATCAGGCATCACAAGCC
AATATGGATCAAGCAAGGCAAATATGTTTGCAATGGGTAATATCAGCATTAAGATCAGTAAGGCATATGT
CCCATAGACCAGGAAACCCAATGTTAGTAAAGCAAAAGAACAGTGAAAGCTATGAAGATTTTATAGCCAG
GCTATTAGAAGCCATAGATGCTGAGCCAGTAACAGACCCTATAAAGACATATCTGAAAGTAACTCTGTCA
TATACAAATGCAAGCACAGACTGTCAAAAACAAATGGACAGAGTATTAGGAACACGGGTACAACAAGCCT
CAGTAGAAGAAAAAATGCAAGCCTGTAGAGATGTAGGATCAGAAGGGTTCAAAATGCAATTATTAGCACA
AGCTTTAAGACCAGGAAGGAATTCAGGAAATCGGGGAGTAGGACAAAAATGTTATAATTGTGGAAAACCG
GGACACTTAGCACGACAGTGTCGCCAAGGCATAATATGCCATCATTGTGGAAAAAGGGGACATATGCAAA
AAGATTGTAGACAAAAGAAGAAAAATGATATAAAACAGCAGGGAAACAACAGGAGGGGGCCACGTGTGGT
GCCGTCCGCGCCCCCTATGTTGTAA

>MT993913.1 Small ruminant lentivirus isolate USMARC-200106929-2, complete genome
ATGGCGAAGCAAGGCTCAAAGGAGAAAAAGGGATACCCCGAGCTCAAAGAAGTAATT
AAAGCAACATGTAAAATAAAAGTAGGGGCCGGGAAGGAGACCTTGACAGAAGGGAACTGTCTATGGGCAT
TAAAAACTGTAGACTTTATATTTGAGGATATAAAAACAGAACCGTGGACTCTTACAAAGATGTATACAGT
ATGGGGAAGATTAAAGCAGTTAACTCCAGAGGAAACAAGTAAAAGAGAGTTTGCCTCCTTACAAGCTACA
ATGGCTTGCATAATGTGTAGTCAAATGGGCATGAAGCCTGAGACAGTGCAGGCAGCACGGGGAATAATAA
GTATGAAGGAAGGGCTACGAGAAAATAAGGAGGACAAAGAAGGGGAGGTAGAGCAACTCTACCCAAATCT
AGAGAAGCACAAGGAAGTATACCCCATTGTGAATCTGCAAGCGGGGGGAAGGAGTTGGAAGGCAGTAGAT
TCTGTGGTCTTCCAACAGCTGCAAAATGTAGCGATGCAGCATGGACTTGTGTCCGAGGATTTTGAGAGGC

AAATAGCATATTATGCCACCACGTGGACAAGCAAGGATATATTAGAAGTATTGGCTATGATGCCTGGAAA
CAGAGCACAGAAAGAATTAATACAAGGGAGATTAAATGAAGAGGCAGAAAGATGGGTGAGACAGAATCCG
CCGGGGCCAAATGTTCTTACAGTGGATCAAATCATGGGAGTGGGACAGACAAATCATCAGGCATCACAAG
CCAATATGGATCAAGCAAGGCAAATATGTTTGCAATGGGTAATATCAGCATTAAGGTCAGTAAGGCACAT
GTCACATAGACCAGGAAACCCAATGTTAGTAAAGCAAAAGAACAGTGAAAGCTATGAAGATTTTATAGCC
AGGCTATTAGAAGCCATAGATGCTGAACCAGTAACAGACCCTATAAAGACATATCTGAAAGTAACTTTGT
CATATACAAATGCAAGCACAGACTGTCAAAAACAAATGGACAGAGTATTAGGAACACGGGTACAACAAGC
CTCAGTAGAAGAAAAAATGCAAGCCTGTAGAGATGTAGGATCAGAAGGGTTCAAAATGCAATTATTAGCA
CAAGCTTTAAGACCAGAAAGGAATTCAGGAAATCGGGGAACAGGACAAAAATGTTATAATTGTGGGAAAC
CGGGACATTTGGCAAGACAATGCAGGCAAGGCATAATATGCCATCATTGTGGAAAAAGGGGACATATGCA
AAAAGATTGTAGACAAAAGAAGAAAAATGATATAAAACAGCAGGGAAACAGCAGGAGGGGGCCACGTGTG
GTGCCGTCCGCGCCCCCTATGTTGTAA

>MH916859.1 Small ruminant lentivirus isolate 1150, complete genome
ATGGCGAAGCAAGGCTCAAAGGAGAAAAAGGGATACCCCGAGCTCAAAGAAGTAATTAAAGCAACATGTA
AAATAAAAGTAGGGGCCGGGAAGGAGACCTTGACAGAAGGGAACTGTCTATGGGCATTAAAAACTGTAGA
CTTTATATTTGAGGATATAAAGACAGAACCGTGGACTCTTACAAAAATGTATACAGTATGGGGCAGATTA
AAACAGTTGACTCCAGAAGAGACAAGTAAAAGAGAGTTCGCCTCCTTGCAGGCTACAATGGCTTGCATAA
TGTGTAGTCAAATGGGCATGAAGCCGGAGACAGTGCAGGCAGCACAGGGAATAATAAGTATGAAGGAAGG
GCTACACGAGAATAAGGAGGACAAGGAGAAAAAGGTAGAGCAACTCTACCCGAACTTAGAGAAGCACAAG
GAAGTATACCCTATTGTGAACTTGCAAGCAGGGGGAAGGAGTTGGAAGGCAGTGGACTCAGTGGTCTTCC
AACAGCTGCAAAATGTAGCAATGCAGCATGGACTTGTGTCCGAGGACTTTGAAAGGCAAATGGCATATTA
TGCCACGACATGGACAAGCAAGGATATATTAGAAGTATTGGCCATGATGCCTGGGAACAGAGCTCAGAAA
GAATTAATACAAGGAAAATTAAATGAGGAAGCGGAGAGATGGGTGAGACAGAATCCGCCAGGGCCAAATG
TCCTTACAGTGGATCAAATCATGGGAGTAGGACAAACAAATCAGCAAGCATCGCAAGCCAACATGGATCA
AGCAAGACAGATATGCTTGCAATGGGTAATAACTGCATTGAGATCTGTAAGACATATGTCTCATAGACCA
GGGAATCCTATGCTAGTGAAGCAAAAGAACAATGAAAGTTATGAAGATTTTATAGCAAGACTATTAGAAG
CCATAGATGCCGAGCCAGTTACAGACCCTATAAAGACATATTTGAAAGTAACTCTGTCATATACAAATGC
TAGCACAGATTGTCAAAAACAAATGGACAGAGTATTAGGGACACGAGTACAGCAAGCAACAGTAGAAGAA
AAAATGCAGGCATGTAGAGATGTGGGATCAGAAGGGTTTAAAATGCAGTTGTTAGCGCAAGCATTAAGGC
CAGAGAAAAACTTGGGAAATAGAGGACAAGGGCAAAAATGTTATAATTGTGGAAAACCGGGACATTTGGC
AAGGCAGTGTAGGCAAGGCATCATATGCCATCATTGTGGAAAACGGGGGCATATGCAAAAAGATTGCCGG
CAAAAGAAGAGGCAAGAAATGAAGCAGCAGGGAAACAGCAGGAGGGGGCCACGTGTGGTGCCGTCCGCGC
CCCCTATGTTGTAA

>MT993917.1 Small ruminant lentivirus isolate USMARC-199916128-2, complete genome
ATGGCGAAGCA
AGGCTCAAAGGAGAAAAAGGGATACCCCGAGCTCAAAGAAGTAATTAAGGCAACATGTAAAATAAAAGTA
GGGGCCGGGAAGGAGACCTTGACAGAAGGGAACTGTCTATGGGCATTAAAAACTATAGACTTTATCTTTG
AGGATATAAAAACAGAGCCGTGGACTCTTACAAAAATGTATACAGTATGGGAAAGATTAAAACAGTTAAC
TCCAGAAGAGACAAGTAAGAGAGAGTTTGCCTCCTTACAAGCTACATTAGCTTGCATAATGTGTAGTCAA
ATGGGCATGAAGCCTGAGACAGTGCAGGCAGCACGGGGAATAATAAGTATGAAAGAAGGACTACGAGAAA
ATAAGGAGGGCAAAGAAAAGGAGGTAGAACAACTCTACCCAAATCTAGAGAAGCACAAGGAAGTATACCC
CATTGTGAATCTGCAAGCGGGGGGAAGGAGTTGGAAGGCAGTAGAGTCTGTGGTCTTCCAACAGCTGCAA
AATGTAGCAATGCAGCATGGACTTGTGTCCGAGGATTTTGAAAGGCAACTAGCATATTATGCCACTACAT
GGACAAGTAAGGATATATTAGAAGTATTGGCTATGATGCCTGGGAACAGAGCACAAAAAGAATTAATACA
AGGGAGATTAAATGAAGAAGCAGAAAGATGGGTGAGACAAAATCCGCCAGGGCCAAATGTCCTTACAGTG
GATCAAATCATGGGAGTAGGACAGACAAATCAGCAGGCATCACAAGCTAATATGGATCAAGCAAGACAAA
TATGTTTGCAATGGGTAATATCAGCATTAAGGTCGGTAAGACATATGTCGCACAGACCAGGAAACCCCAT
GTTAGTAAAACAAAAGAATAATGAAAGCTATGAAGACTTTATAGCAAGGTTGTTAGAAGCAATCGATGCA
GAACCAGTGACGGACCCCATCAAAACATATCTGAAAGTAACTCTGTCGTATACAAATGCAAGTACAGATT
GTCAGAAACAGATGGACAGAGTGTTAGGAACAAGAGTTCAACAAGCAACAGTGGAAGAAAAGATGCAAGC
CTGTAGAGATGTAGGATCAGAGGGATTTAAAATGCAATTATTAGCACAGGCTTTAAGACCAGAAAGGAAT
TCAGGAAATCGGGGAGCAGGACAAAAATGTTATAATTGTGGAAAACCGGGACATTTGGCAAGACAATGCA
GGCAAGGCATAATATGCCATCATTGTGGAAAAAGAGGACATATGCAAAAAGATTGTAGACAAAAGAAGAA
AAATGATATGAAACAGCAGGGAAACAACAGGAGGGGGCCACGTGTGGTGCCGTCCGCGCCCCCTATGTTG
TAA

>MT993910.1 Small ruminant lentivirus isolate USMARC-200312088-r, complete genome

ATGGCGAAGCAAGGCTCAAAGGAGAAAAAGGGATACCCCGAGCTCAAGGAAGTGAT
TAAAGCTACATGTAAAATAAGAGTAGGAGCCGGGAAGGAGACCTTGACAGAAGGGAACTGTCTATGGGCA
TTAAAGACTGTAGACTTTATATTTGAGGATATAAAGACAGAGCCGTGGACTCTTACAAAAATGTATACTG
TATGGGGCAGGCTAAAGCAGTTAACTCCGGAAGAGACAAGTAAAAGAGAGTTTGCCTCCTTGCAAGCTAC
AATGGCTTGCTTAATGTGCAGTCAAATGGGCATGAAGCCTGAGACAGTGCAGGCAGCACGGGGAATAATA
GGCATGAAGGAAGGACTGCAAGAGAATAAGGAGGAACAAGAAAAGAAGGTAGAGCAACTCTACCCAAATT
TAGAAAAGCATAAGGAAGTATACCCCATTGTGAATTTGCAAGCGGGGGGAAGGAGTTGGAAGGCAGTAGA
CTCAGTGGTCTTCCAACAGCTGCAAAATGTAGCGATGCAGCATGGACTTGTGTCCGAGGATTTTGAAAGG
CAAATAGCATATTATGCTACCACATGGACAAGTAAGGATATATTAGAAGTATTGGCCATGATGCCTGGAA
ACAGAGCACAGAAAGAATTAATACAAGGGAGATTAAATGAAGAAGCAGAAAGGTGGGTGAGACAGAATCC
GCCGGGGCCAAATGTCCTTACAGTGGATCAAATAATGGGAGTGGGACAAACAAATCACCAGGCATCACAA
GCTAATATGGATCAAGCAAGACAAATATGTTTGCAATGGGTGATATCAGCATTAAGGTCGGTAAGGCATA
TGTCGCACAGACCAGGAAACCCCATGTTAGTAAAACAAAAGAATAATGAAAGCTATGAAGACTTTATAGC
AAGGTTGTTAGAAGCAATTGATGCAGAACCAGTTACCGACCCTATAAAAACATATTTGAAAGTAACTCTG
TCATATACAAATGCTAGCACAGATTGTCAAAAACAAATGGACAGAGTATTAGGAACACGAGTACAGCAAG
CAACGGTAGAAGAAAAGATGCAAGCATGTAGAGATGTAGGATCAGAAGGATTTAAAATGCAGTTATTAGC
ACAAGCCTTGAGGCCAGAAAAAAGTTCAGGAAACAGAGGAGCAGGGCAAAAATGTTATAATTGTGGGAAA
CCGGGACATTTGGCAAGACAATGCAGGCAAGGAATAATATGCCATCATTGTGGAAAAAGAGGACACATGC
AAAAGGATTGTAGGAAAAAGAAAGGGGATATGAAGCAGCAGGGAAACAGCAGGAGGGGGCCACGTGTGGT
GCCGTCCGCGCCCCCTATGTTGTAA

>MT993915.1 Small ruminant lentivirus isolate USMARC-200335185-2, complete genome
ATGGCGAAGCAAGGCTCAAAGGAGAAAAAGGGATACCCCGAGCTCAAAGAAGTAAT
TAAAATGACGTGTAGAATAAGGGTAGGGGCCGGGAAGGAGACCTTGACAGAAGGGAACTGTCTATGGGCA
TTAAAAACTGTAGACTTTATCTTTGAGGATATAAAAACAGAGCCGTGGACTCTTACAAAAATGTATACAG
TATGGGAAAGATTAAAACAGTTAACTCCAGAAGAGACAAGTAAGAGAGAGTTTGCCTCCTTACAAGCTAC
ATTAGCTTGCATAATGTGTAGTCAAATGGGCATGAAGCCTGAGACAGTGCAGGCAGCACGGGGAATAATA
ACTATGAAAGAAGGACTACGAGAAAATAAGGAGGACAAAGAGAAAAAGGTAGAGCAACTCTACCCAAATC
TAGAGAAGCACAAGGAAGTATACCCCATTGTGAATTTGCAAGCGGGGGGAAGGAGTTGGAAGGCAGTAGA
TTCTGTGGTCTTCCAACAGCTGCAAAATGTAGCAATGCAGCATGGACTTGTGTCCGAGGATTTTGAGAGG
CAAGTAGCATATTATGCCACCACATGGACAAGTAAGGATATATTAGAAGTATTGGCCATGATGCCAGGAA
ATAGAGCTCAGAAAGAGTTAATACAAGGGAAATTAAATGAGGAAGCAGAAAGATGGGTAAGGCAGAATCC
CCCAGGGCCAAATGTCCTTACAGTGGATCAAATTATGGGGGTAGGACAAACAAATCAACAAGCATCGCAA
GCTAACATGGATCAAGCGAGGCAAATATGCTTGCAATGGGTAATAACAGCATTGAGATCGGTAAGACATA
TGTCCCATAGACCAGGAAATCCTATGTTAGTAAAGCAGAAGAATACAGAAAGCTATGAAGACTTTATAGC
AAGACTATTAGAGGCCATAGATGCAGAACCAGTGACGGACCCCATCAAAACATATTTGAAAGTAACTCTG
TCATATACAAATGCAAGTACAGATTGTCAGAAACAGATGGACAGAGTGTTAGGAACAAGAGTACAACAAG
CAACAGTAGAAGAAAAGATGCAAGCATGTAGAGATGTAGGATCAGAAGGATTTAAAATGCAGTTATTAGC
ACAAGCCTTGAGGCCAGAAAAAAGTTCAGGAAACAGAGGATCAGGACAAAAATGTTATAATTGTGGAAAA
CCGGGACACTTAGCACGACAGTGTCGACAAGGCATAATATGCCATCATTGTGGAAAAAGGGGACATATGC
AAAAAGATTGTAGACAGAAAAAGAAAAATGATATGAAACAGCAGGGAAACAGCAGGAGGGGGCCACGTGT
GGTGCCGTCTGCACCCCCTATGTTGTAA

>MT993909.1 Small ruminant lentivirus isolate USMARC-200312013-r, complete genome
ATGGCGAAGCAAGGCTCAAAGGAGAAAAAGGGATACCCCGAGCTCAAAGAAGTAATTA
AGGCAACATGTAAAATAAGAGTAGGGGCCGGGAAGGAGACCTTGACAGAAGGGAACTGTCTATGGGCATT
AAAGACTGTAGACTTTATATTTGAGGATATAAAGACAGAGCCGTGGACTCTTACAAAAATGTATACAGTA
TGGGGAAGATTAAAGCAGCTAACTCCAGAGGAAACAAGTAAAAGAGAGTTTGCCTCCTTGCAAGCTACAA
TGGCTTGCTTAATGTGCAGTCAAATGGGCATGAAGCCTGAGACAGTGCAGGCAGCACGGGGAATAATAGG
CATGAAGGAAGGACTGCAAGAGAATAAGGAGGAACAAGAAAAGAAGGTAGAGCAACTCTACCCAAATTTA
GAAAAGCATAAGGAAGTATACCCCATTGTGAATTTGCAAGCGGGGGGAAGGAGTTGGAAGGCAGTAGACT
CAGTGGTCTTCCAACAGCTGCAAAATGTAGCGATGCAGCATGGACTTGTGTCCGAGGATTTTGAAAGGCA
AATAGCATATTATGCTACCACATGGACCAGTAAGGATATTTTAGAAGTATTGGCCATGATGCCTGGGAAT
AGAGCACAGAAAGAATTAATACAAGGGAAATTGAATGAAGAAGCAGAAAGATGGGTGAGACAGAACCCGC
CAGGGCCAGGTGTCCTTACAGTGGATCAAATCATGGGAGTAGGACAAACAAATCATCAGGCATCACAAGC
TAATATGGATCAAGCAAGACAAATATGTTTGCAATGGGTAATATCAGCATTAAGATCAGTAAGGCATATG
TCACATAGGCCCGGAAACCCTATGTTAGTAAAGCAAAAGAATAATGAAAGCTATGAAGACTTTATAGCAA
GGTTGTTAGAAGCAATTGATGCAGAACCAGTTACCGACCCTATAAAGACATATCTAAAGTAACTCTGTC
ATATACAAATGCTAGCACAGATTGTCAAAAACAAATGGACAGAGTGATAGGAACAAGAGTACAACAAGCC

TCAGTAGAAGAGAAAATGCAAGCATGTAGAGATGTGGGATCAGAAGGGTTTAAGATGCAACTATTAGCGC
AGGCTTTAAGGCCAGAAAAAACCCCGGGAAATAAAGGACCAGGACAGAAATGTTATAATTGTGGAAAACC
GGGACATTTGGCAAGGCAGTGTAGACAAGGCATAATATGTCATCATTGTGGAAAAAGAGGGCATATGCAA
AAGGATTGCAGAAAAAAGAAAAGTGAGAACTTCAAGCAGCAGGGAAACAGCAGGAGGGGGCCACGTGTGG
TGCCGTCCGCGCCCCCTATGTTGTAA

>MT993905.1 Small ruminant lentivirus isolate USMARC-201373037-1,
complete genome
ATGGCGAAGCAAG
GCTCAAAGGAGAAAAAGGGATACCCCGAGCTCAAGGAAGTGATTAAAGCTACATGTAAAATAAAGGTAGG
GGCCGGGAAGGAGACCTTGACAGAAGGGAACTGTCTATGGGCATTAAAGACTGTAGACTTTATATTTGAG
GATATAAAGACAGAGCCGTGGACTCTTACAAAAATGTGTACTGTATGGGATAGGCTAAAGCAGTTAACTC
CGGAAGAGACAAGTAAAAGAGAGTTTGCCTCCTTGCAAGCTACAATGGCTTGCTTAATGTGCAGTCAAAT
GGGCATGAAGCCTGAGACAGTGCAGGCAGCACGGGGAATAATAGGCATGAAGGAAGGACTGCAAGAGAAT
AAGGAGGAACAAGAAAAGAAGGTAGAGCAACTCTACCCAAATTTAGAAAAGCATAAGGAAGTATACCCCA
TTGTGAATTTGCAAGCGGGGGGAAGGAGTTGGAAGGCAGTAGACTCAGTGGTCTTCCAACAGCTGCAAAA
TGTAGCGATGCAGCATGGACTTGTGTCCGAGGATTTTGAAAGGCAAATAGCATATTATGCCACTACATGG
ACCAGTAAGGATATTTTAGAAGTATTGGCCATGATGCCTGGGAATAGAGCACAGAAAGAATTAATACAAG
GGAAATTGAATGAAGAAGCAGAAAGATGGGTAAGACAGAATCCGCCAGGGCCAGGTGTCCTTACAGTGGA
TCAAATCATGGGAGTAGGACAAACAAATCATCAGGCATCACAAGCTAATATGGATCAAGCAAGGCAAGTA
TGCTTGCAATGGGTAATATCAGCATTAAGATCAGTAAGACATATGTCACATAGACCAGGAAACCCTATGC
TAGTAAAGCAGAAAAATAGTGAAAGTTATGAAGATTTCATCGCAAGACTGCTAGAGGCTATAGATGCTGA
GCCGGTAACAGACCCTATAAAAACATACTTGAAAGTGACTTTGTCATATACAAATGCTAGCACAGATTGT
CAAAAGCAAATGGACAGAGTGATAGGAACAAGAGTACAACAAGCCTCAGTAGAAGAGAAAATGCAAGCAT
GTAGAGATGTGGGATCAGAAGGGTTTAAGATGCAACTATTAGCGCAAGCTCTAAGGCCAGAAAAAACCCC
AGGAAATAAAGGATCAGGACAGAAATGTTATAATTGTGGAAAACCGGGACATTTGGCAAGGCAGTGTAGA
CAAGGCATAATATGTCATCATTGTGGAAAAAGAGGACATATGCAAAAGGATTGCAGAAAAAAGAAAAGTG
AGAACTTTAAGCAGCAGGGAAACAGCAGGAGGGGGCCACGTGTGGTGCCGTCCGCGCCCCCTATGTTGTA
A

>MT993918.1 Small ruminant lentivirus isolate USMARC-199916193-r,
complete genome
ATGGCGAAGCAAGGCTCAAAGGAGAAAAAGGGATACCCCGAGCTCAGAGAAGT
AATTAAGGCAACATGTAAAAAAGTAGGGGCCGGGAAGGAGACCTTGACAGAAGGGAACTGTCTATGGGCA
TTAAAAACTATAGACTTTATCTTTGAGGATATAAAAACAGAGCCGTGGACTCTTACAAAGATGTATACAG
TATGGGAAAAATTAAAACAGTTAACTCCAGAAGAGACAAGTAAGAGAGAGTTTGCCTCCTTACAAGCTAC
ATTAGCTTGCATAATGTGTAGTCAAATGGGCATGAAGCCTGAGACAGTGCAGGCAGCACGGGGAATAATA
AGTATGAAAGAAGGACTACGAGAAAATAAGGAGGACAAAGAAAAGGAGGTAGAACAACTCTACCCAAATC
TAGAGAAGCACAAGGAAGTATACCCCATTGTGAATCTGCAAGCGGGGGGAAGGAGTTGGAAGGCAGTAGA
ATCAGTAGTCTTTCAGCAGCTACAGACAGTGGCTATGCAGCATGGACTTGTGTCCGAGGATTTTGAAAGG
CAAATAGCATATTATGCCACTACATGGACAAGTAAAGATATATTGGAAGTATTGGCTATGATGCCAGGAA
ATAGAGCTCAGAAAGAGTTAATACAAGGGAAATTAAATGAGGAAGCAGAGAGATGGGTAAGACAGAATCC
CCCGGGGCCAAATGTCCTTACAGTGGATCAAATTATGGGGGTAGGACAAACAAATCAACAAGCCTCGCAA
GCTAATATGGATCAAGCGAGACAAATATGCTTGCAATGGGTGATAACAGCATTGAGATCAGTAAGACATA
TGTCCCATAGACCAGGGAACCCTATGCTAGTAAAGCAGAAGAATACAGAAAGTTATGAGGACTTTATAGC
AAGACTATTGGAAGCAATCGATGCAGAACCAGTGACGGACCCCATCAAAACATATCTGAAAGTAACTCTG
TCATATACAAATGCTAGCACAGATTGTCAGAAACAAATGGACAGAGTATTAGGAACGCGAGTACAGCAAG
CAACAGTAGAAGAAAAGATGCAAGCATGTAGAGATGTAGGATCAGAGGGATTTAAAATGCAATTATTAGC
ACAAGCCTTGAGGCCAGAAAAAAGTTCAGGAAACAGAGGATCAGGGCAAAAATGCTATAATTGTGGAAAA
CCGGGACATTTGGCAAGGCAGTGTAGACAAGGCATAATATGTCATCATTGTGGAAAAAGAGGACATATGC
AAAAGGATTGTAGAAAAAAGAAAAGTGAGAACTTCAAGCAGCAGGGAAACAGCAGGAGGGGGCCACGTGT
GGTGCCGTCCGCGCCCCCTATGTTGTAA

>MT993907.1 Small ruminant lentivirus isolate USMARC-200216049-r,
complete genome
ATGGCGAAGCAAGGCTCAAAGGAGAAAAAGGGATACCCCGAGCTCAAGGAAGTGATTAAA
GCAACATGTAAAATAAAAGTAGGGGCCGGGAAGGAGACCTTGACAGAAGGGAACTGTCTATGGGCATTAA
AGACTGTAGACTTTATATTTGAGGATCTAAAAACAGAGCCGTGGACTCTTACAAAAATGTATACTGTATG
GGGCAGGCTAAAGCAGTTAACTCCGGAAGAGACAAGTAAAAGAGAGTTTGCCTCCTTGCAAGCTACAATA
GCTTGCTTAATGTGCAGTCAAATGGGCATGAAGCCTGAGACAGTGCAGGCAGCACGGGGAATAATAAGTA
TGAAGGAAGGACTGCAAGAGAATAAGGAGGAACAAGAGAAGAAGGTAGAGCAACTCTACCCAAATTTAGA

AAAACATAAGGAAGTATACCCCATTGTGAATCTGCAAGCGGGGGGAAGGAGTTGGAAGGCAGTAGACTCA
GTGGTCTTCCAACAGCTGCAAAATGTAGCGATGCAGCATGGACTTGTGTCCGAGGATTTTGAGAGGCAAA
TAGCATATTATGCCACCACATGGACAAGTAAGGATATCTTAGAAGTATTGGCCATGATGCCAGGAAATAG
AGCTCAGAAAGAGTTAATACAAGGGAAATTAAACGAGGAAGCAGAAAGATGGGTAAGGCAAAATCCACCA
GGGCCAAATGTCCTTACAGTGGATCAAATCATGGGAGTAGGACAAACAAATCATCAGGCATCACAAGCTA
ATATGGATCAAGCAAGGCAAATATGCTTGCAATGGGTAATATCAGCATTAAGATCAGTAAGACATATGTC
ACATAGACCAGGAAACCCTATGCTGGTAAAGCAGAAAAATAGTGAAAGTTATGAAGATTTTATCGCAAGA
CTACTAGAGGCCATAGATGCTGAGCCGGTAACAGACCCTATAAAAACATATTTAAAAGTGACTCTGTCAT
ATACAAATGCTAGCACGGATTGTCAAAAACAAATGGACAGAGTGATAGGAACAAGAGTACAACAAGCCTC
AGTAGAAGAGAAAATGCAAGCATGTAGAGATGTGGGATCGGAAGGGTTTAAGATGCAACTATTGGCACAA
GCTCTAAGGCCAGAAAAAACCCCGGGAAATAGAGGATCAGGACAGAAATGTTATAATTGTGGAAAACCGG
GACACTTAGCAAGGCAGTGTAGACAAGGCATAATATGCCATCATTGTGGAAAAAGAGGACATATGCAAAA
GGATTGCAGAAAAAAGAAAAGTGAAAACTTTAAGCAGCAGGGAAACAGCAGGAGGGGGCCACGTGTGGTG
CCGTCCGCGCCCCCTATGTTGTAA

>MT993914.1 Small ruminant lentivirus isolate USMARC-200016283-2, complete genome
ATGGCGAAGCAAGGCTCAAAGGAGAAAAAGGGATACCCCGAGCTCAAAGAAGTAAT
TAAAATGACGTGTAAAATAAAAGTAGGGGCCGGGAAGGAGACCTTGACAGAAGGGAACTGTCTATGGGCA
TTAAAGACTGTAGACTTTATCTTTGAGGATATAAAAACAGAGCCGTGGACTCTTACAAAAATGTATACAG
TATGGGAAAGATTAAAACAGTTAACTCCAGAAGAGACAAGTAAGAGAGAGTTTGCCTCCTTACAAGCTAC
ATTAGCTTGCATAATGTGTAGTCAAATGGGCATGAAGCCTGAGACAGTGCAGGCAGCACGGGGAATAATA
AGTATGAAAGAAGGACTACGAGAAAATAAGGAGGACAAAGAAAAGGTAGAGCAACTCTACCCAAGTCTAG
AGAAGCATAAGGAAGTATACCCCATTGTGAATTTGCAAGCGGGGGGAAGGAGTTGGAAGGCAGTAGAATC
AGTGGTCTTCCAACAGCTGCAAAATGTAGCAATGCAGCATGGACTTGTGTCCGAGGATTTTGAAAGGCAG
CTAGCCTATTATGCTACTACCTGGACAAGTAAAGATATTTTAGAGGTATTGGCCATGATGCCAGGAAATA
GAGCTCAGAAAGAGTTAATACAAGGGAAATTAAACGAGGAAGCAGAAAGATGGGTAAGGCAAAATCCGCC
AGGGCCAAATGTCCTTACAGTGGATCAAATTATGGGGGTAGGACAAACAAATCAACAAGCATCGCAAGCT
AACATGGATCAAGCGAGACAAATATGCTTGCAATGGGTAATAACAGCATTGAGATCGGTAAGACATATGT
CCCATAGACCAGGGAATCCTATGCTAGTAAAGCAGAAGAATACAGAAAGTTATGAGGACTTCATAGCAAG
ACTATTGGAAGCAATCGATGCAGAACCAGTGACGGACCCCATCAAAACATATTTGAAAGTAACTCTGTCA
TATACAAATGCAAGTACAGATTGTCAGAAACAGATGGACAGAGTGTTAGGAACAAGAGTTCAACAAGCAA
CAGTGGAAGAAAAGATGCAAGCATGTAGAGATGTAGGATCAGAAGGATTTAAAATGCAATTATTAGCACA
AGCCTTGAGGCCAGAAAAAAGTTCAGGAAATCGAGGATCAGGGCAAAAATGTTATAATTGTGGAAAACCG
GGACACTTAGCACGGCAGTGTCGACAAGGCATAATATGCCATCATTGTGGAAAAAGGGGACATATGCAAA
AAGATTGTAGACAAAAGAAGAAAAATGATATGAAGCAGCAGGGAAACAGCAGGAGGGGGCCACGTGTGGT
GCCGTCCGCGCCCCCTATGTTGTAA

>MZ313872.1 Visna-maedi virus isolate XM-MDV30, complete genome
ATGGCGAAGCAAGGCTCAAAGGAGAAAAAGGGAT
ACCCCGAGCTCAAGGAAGTAATTAAAGCAACATGTAGAATAAAGGTAGGGGCCGGGAAGGAGACCTTGAC
AGAAGGGAACTGTCTATGGGCATTAAAAACTGTAGACTTTATATTTGCAGATATAAAGACAGAACCGTGG
ACTCTTACAAAGATGTATACTGTATGGGACAGATTAAGACAGTTGACTCCAGAAGAGACAAGTAAAAGAG
AGTTTGCCTCCTTGCAAGCTACATTGGCTTGCCTAATGTGTAGTCAAATGGGCATGAAGCCTGAGACAGT
GCAGGCAGCACGGGGAATAATAAGTATGAAGGAAGGACTACAAGAGAATAAGGAGGAAAAGAAGGTAGAA
CAACTCTACCCAAATTTAGAGAAGCATAAGGAAGTATACCCCATTGTGAATTTGCAAGCGGGGGGAAGGA
GTTGGAAGGCAGTAGACTCCGTAGTCTTCCAACAGCTGCAAAATGTAGCAATGCAGCATGGACTTGTGTC
CGAGGATTTTGAAAGGCAAATGGCATATTATGCCACCACATGGACAAGTAAAGATATATTAGAAGTATTG
GCCATGATGCCTGGAAATAGAGCTCAGAAAGAGTTAATACAAGGGAAATTAAATGAGGAAGCGGAGAGAT
GGGTAAGGCAGAATCCGCCAGGGCCAAACGTCCTTACAGTGGATCAAATTATGGGAGTAGGACAAACAAA
TCAACAGGCATCACAAGCTAACATGGATCAGGCAAGACAGATATGCTTGCAATGGGTAATAACCGCATTA
AGATCTGTAAGACATATGTCTCATAGGCCAGGAAATCCTATGCTGGTGAAGCAGAAAAATACAGAGAGTT
ATGAAGACTTCATAGCCAGACTGTTGGAAGCAATCGATGCAGAACCAGTCGCAGATCCTATAAAAACATA
CTTGAAAGTAACTCTGTCATATACAAATGCTAGCACAGATTGTCAAAAACAAATGGACAGAGTGTTAGGA
ACTAGAGTCCAACAAGCAACAGTAGAAGAAAAGATGCAAGCCTGTAGAGACGTAGGATCAGAAGGGTTTA
AAATGCAACTATTAGCGCAGGCTTTAAGACCAGAAAAGAGTGCAGGAAATCGGGGAGCAGGACAAAAATG
TTATAATTGTGGAAAACAGGGACATTTAGCAAGGCAGTGTAGGCAAGGCATAATATGCCATCATTGTGGA
AAAAGAGGACATATGCAAAAAGATTGCAGGAAAAAGAAAAGAGAGAACATGAAGCAGCAGGGAAACAGCA
GGAGGGGGCCACGTGTGGTGCCGTCCGCGCCCCCTATGCTGTAA

>MZ484405.1 Small ruminant lentivirus isolate BEL-LX160316, complete genome
ATGGCGAAGCAAGGCTCGAAGGAGAAAAAGGGATACCCTGAGCTCAAGGA
TGTCATTAAATTGACATGTAAGATAAAGATAGGGGCCGGGAAGGAGACCTTGACAGAAGGGAATTGTCTA
TGGGCATTAAAAACCCTAGACTTTATATTTGAGGATATAAGCTCAGAACCGTGGACTCTAACAAAGATGT
ACACAGTATGGGGAAGGTTAAAACAGTTAACTCCAGAGGAGTCAAGTAAGAGAGAATTTGCCTCGTTGCA
GGCCACAATGGCTTGTTTGATGTGTAGTCAAATGGGCATGAAGCCCGAGACAGTGCAGGCAGCAAGGGGA
ATAATGAGTATGAAAGAAGGACTACACGAGAATAAGGAAGAAAAGGAGAAAAAGGTAGAACAACTCTACC
CAAACCTAGAGAGGCATAAAGAAGTATATCCTATTGTGAATTTACAAGCAGGAGGGAGGAGCTGGAAGGC
GGTAGATTCGGTAGTCTTCCAACAACTGCAGAATGTGGCAATGCAGCATGGGCTTGTGTCCGAGGATTTT
GAGAGACAAATGGCATACTATGCCACTACGTGGACAAGTAAAGATATTTTAGAAGTGCTGGCCATGATGC
CTGGAAACAGAGCCCAGAAAGAGTTGATACAAGGAAAATTAAATGATGAAGCAGAGAGATGGGTGAGACA
GAATCCACCGGGCCCAAATGTCCTTACAATGGATCAAATTATGGGAGTAGGACAAACAAATCAGCAGGCC
TCGCAGGCCAATATGGATCAGGCAAGACAAATATGTCTGCAGTGGGTAATATCTGCCTTAAGATCAGTCA
GGCATATGGCACATAGACCAGGAAATCCTATGCTAGTGAAGCAAAAAACTAATGAGAGTTATGAGGAATT
CATAGCGAGGCTGCTAGAGGCAATTGATGCAGAGCCAGTCACTGATCCAATAAAGACATATTTGAAAGTA
ACGTTGTCTTATACAAATGCTAGTACTGACTGTCAAAAACAAATGGATAGGGTACTGGGTACTAGGGTCC
AACAGGCATCAGTGGAAGAAAAAATGCAGGCATGTCGGGATGTAGGATCCGAAGGATTTAAAATGCAGCT
ATTAGCACAAGCTTTAAGGCCACAGAGGAGAGAAGGGAAGGCAGGGGCAAATCAGAAGTGCTATAATTGT
GGAAAGCCAGGACATCTAGCACGGCAATGTAGACAAGGAATAATATGTCATAATTGTGGGAAACGAGGAC
ATGTGCAAAGAGATTGCCGACTGAAGAAAAAGAATAACATAGGACAGCAGGGAAACAGCAGGGGGGGGAC
CACGTGTGGTGCCGTCCGCGCCCCCCTATGTTGTAACAGAAGCACCACCCAGAATAGAAGTAAGGATAGG
AACAGAGTGGAAAAGGTTATTAGTAGACACAGGGGCTGA

>MT993916.1 Small ruminant lentivirus isolate USMARC-200177363-2, complete genome
ATGGCGAAGCAAGGCTCAAAGGAGAAAAAGGGATACCCCGAGCTCAAAGAAGTAAT
TAAAATGACATGTAAAATAAAAGTAGGGGCCGGGAAGGAGACCTTGACAGAAGGGAACTGTCTATGGGCA
TTAAAAACTGTAGACTTTATCTTTGAGGATATAAAAACAGAGCCGTGGACTCTTACAAAAATGTATACAG
TATGGGAAAGATTAAAACAGTTAACTCCAGAAGAGACAAGTAAGAGAGAGTTTGCCTCCTTACAAGCTAC
ATTAGCTTGCATAATGTGTAGTCAAATGGGCATGAAGCCTGAGACAGTGCAGGCAGCACGGGGAATAATA
AGTATGAAAGAAGGACTACGAGAAAATAAGGAGGACAAAGAAAAGGAGGTAGAACAACTCTACCCAAATC
TAGAGAAGCACAGGGAAGTATACCCCATTGTGAATCTGCAAGCGGGGGGAAGGAGTTGGAAGGCAGTAGA
TTCTGTGGTCTTCCAACAGCTGCAAAATGTAGCAATGCAGCATGGACTTGTGTCCGAGGATTTTGAAAGG
CAAATAGCATATTATGCTACTACCTGGACAAGTAAAGATATATTGGAAGTATTGGCCATGATGCCAGGAA
ATAGAGCTCAGAAAGAGTTAATACAAGGGAAATTAAATGAGGAAGCAGAGAGATGGGTAAGGCAAAATCC
GCCAGGGCCAAATGTCCTTACAGTGGATCAAATTATGGGGGTAGGACAAACAAATCACCAAGCATCGCAA
GGTAACATGGATCAAGCGAGACAAATATGTTTGCAATGGGTAATATCAGCATTGAGATCGGTAAGACATA
TGTCCCATAGACCAGGGAATCCTATGCTAGTAAAGCAGAAGAATACAGAAAGTTATGAGGACTTCATAGC
AAGACTATTGGAAGCAATCGATGCAGAACCAGTTACCGACCCTATAAAAACATATTTGAAAGTAACTCTG
TCATATACAAATGCAAGTACAGATTGTCAGAAACAAATGGACAGAGTGTTAGGAACAAGAGTCCAACAAG
CAACAGTAGAAGAAAAGATGCAAGCCTGTAGAGATGTAGGATCAGAGGGATTTAAAATGCAATTATTAGC
ACAGGCTTTAAGACCAGAAAGGAATTCAGGAAATCGGGGAGCAGGACAAAAATGTTATAATTGTGGAAAA
CCGGGACATTTGGCAAGACAATGCAGGCAAGGCATAATATGCCATCATTGTGGAAAAAGGGGACATATGC
AAAAAGATTGTAGACAAAAGAAGAAAAATGATATGAAACAGCAGGGAAACAACAGGAGGGGGCCACGTGT
GGTGCCGTCCGCGCCCCCTATGTTGTAA

>MT993908.1 Small ruminant lentivirus isolate USMARC-200212120-r, complete genome
ATGGCGAAGCAAGGCTCAAAAGAGAAAAAGGGATACCCCGAGCTCAA
AGAAGTAATTAAAGCAACATGTAAAATAAGAGTAGGGGCCGGGAAGGAGACCTTGACAGAAGGGAACTGT
CTATGGGCATTAAAGACTGTAGACTTTATATTTGAGGATATAAAGACAGAGCCGTGGACTCTTACAAAGA
TGTATACAGTATGGGGAAGATTAAAGCAGCTAACTCCAGAGGAAACAAGTAAAAGAGAGTTTGCCTCCTT
GCAAGCTACAATGGCTTGCTTAATGTGCAGTCAAATGGGCATGAAGCCTGAGACAGTGCAGGCAGCACGG
GGAATAATAGGCATGAAGGAAGGACTGCAAGAGAATAAGGAGGAACAAGAAAAGAAGGTAGAGCAACTCT
ACCCAAATTTAGAAAAGCATAAGGAAGTATACCCCATTGTGAATTTGCAAGCGGGGGGAAGGAGTTGGAA
GGCAGTAGATTCTGTGGTCTTCCAACAGCTGCAAAATGTAGCGATGCAGCATGGACTTGTGTCCGAGGAT
TTTGAAAGGCAAATAGCATATTATGCCACCACATGGACCAGTAAGGATATTTTAGAAGTATTGGCCATGA
TGCCTGGGAATAGAGCACAGAAAGAATTAATACAAGGGAAATTGAATGAAGAAGCAGAAAGATGGGTGAG
ACAGAATCCGCCGGGGCCAAATGTCCTTACAGTGGATCAAATCATGGGAGTAGGACAAACAAATCATCAG
GCATCACAAGCTAATATGGATCAAGCAAGACAAATATGTTTGCAATGGGTAATATCAGCATTAAGGTCGG

TAAGGCATATGTCGCACAGACCAGGAAACCCCATGTTAGTAAAACAAAAGAATAATGAAAGCTATGAAGA
TTTTATAGCAAGGTTGTTAGAAGCAATTGATGCAGAACCAGTTACCGACCCTATAAAAACATATTTGAAA
GTAACTCTGTCATATACAAATGCTAGCACGGATTGTCAAAAACAAATGGACAGAGTGATAGGAACAAGAG
TACAACAAGCCTCAGTAGAAGAGAAAATGCAAGCATGTAGAGATGTGGGATCAGAAGGGTTTAAGATGCA
ACTATTAGCACAGGCTCTAAGGCCAGAAAAAACCCCGGGAAATAAAGGACCAGGACAGAAATGTTATAAT
TGTGGAAAACCGGGACATTTGGCAAGGCAGTGTAGACAAGGCATAATATGTCATCATTGTGGAAAAAGAG
GACATATGCAAAAGGATTGCAGAAAAAAGAAAAGTGAGAACTTCAAGCAGCAGGGAAACAGCAGGAGGGG
GCCACGTGTGGTGCCGTCCGCGCCCCCTATGTTGTAA

>NC_001511.1 Ovine lentivirus, complete genome
ATGGCGACGCAAGGCTCAAAGGAGAAGAAGGGATACCCTGA
GCTCAAAGAGGTCATTAAGACAACATGTAAAATAAAGGTGGGGCCCGGGAAGGAGACCTTGACAGAAGGG
AACTGTCTATGGGCCTTAAAAACCCTAGACTTTATATTTGAGGATATAAAAACGGAGCCGTGGACTCTTA
CAAAAATGTATACAGTCTGGGAAAAATTAAAGCAAGTAACTCCAGAAGAAACAAGTAAAAGAGAGTTTGC
CTCCTTACAGGCCACATTGGCTTGTATAATGTGTAGTCAAATGGGCATGAGGCCCGAGACAGTGCAGGCA
GCCAGGGGAATAATAAGTATGAAAGAAGGGCTACACGAAAAACAGGAGGATAAAGAAAAGAAGGTAGAAC
AACTCTACCCAAACTTGGAAAAACACAGAGAAGTGTATCCTATTGTAAATTTGCAGGCTGGAGGGAGAAG
TTGGAAAGCGGTAGAGTCAGTGACATTCCAGCAGCTGCAAACAGTAGCAATGCAGCATGGACTTGTGTCC
GAGGATTTTGAAAGACAATTAGCATATTATGCCACTACATGGACAAGCAAAGATATATTAGAAGTACTAG
CCATGATGCCTGGAAATAGGGCGCAAAAAGAGTTAATACAGGGGAAATTAAATGAGGAAGCAGAAAGGTG
GGTAAGGCAGAATCCCCCAGGGCCAAATGTCCTTACTGTGGATCAGATTATGGGAGTCGGACAAACAAAT
CAACAGGCATCGCAAGCTAATATGGATCAGGCAAGACAGCTTTGCTTGCAGTGGGTAATAACAGCCTTGA
GATCAGTAAGACATATGTCACACAGACCAGGAAATCCTATGCTGATAAAACAAAAGAATAGTGAAAGTTA
TGAAGATTTTATAGCAAGATTGCTAGAAGCAATTGATACAGAACCCGTCACGGATCCTATAAAAACATAT
TTAAAAGTAACTCTGTCGTTCACAAATGCTAGCACAGATTGTCAAAAACAAATGGACAGAGTGTTAGGGA
CAAGAGTCCAACAGGCATCAGTAGAAGAAAAAATGCAAGCATGTCGGGACGTAGGATCAGAAGGATTTAA
AATGCAGCTGTTAGCACAGGCATTGAGACCGCCACGGAAGGAAGGAAAACAGGGAGTACAAAAATGCTAT
TACTGCGGGAAACCAGGACATCTCGCAAGGCAATGCAGACAAGGAATAATATGTCATCATTGTGGGAAAA
GAGGACATATGCAAAAAGATTGCAGACAAAAGAAAGGTAATCCAACATCACAGCAGGGAAACAGCAGGAG
GGGGCCACGTGTGGTGCCGTCCGCGCCCCCTATGTTGTAA

>GQ255401.1 Ovine progressive pneumonia virus isolate 76.4 gag protein (gag) gene, partial cds
GAGAAAAAGGGATACCCCGAGCTCAAGGAAGTGATTAAGGCAACATGTAAAATAAAAGTAGGGGCCGGGA
AGGAGACCTTGACAGAAGGGAACTGTCTATGGGCATTAAAAACCGTAGACTTTATATTTGAGGATATCAA
AACGGAGCCGTGGACTCTTACAAAGATGTATACAGTATGGGAAAGATTAAGGCAGTTAACTCCAGAAGAG
ACAAGTAAAAGAGAGTTTGCCTCCTTGCAAGCTACAATGGCTTGCCTAATGTGTAGTCAAATGGGCATGA
AGCCCGAGACGGTGCAGGCAGCACAGGGAATAATAGGTATAAAAGAAGGACTACGAGAAAATAAGGAGGA
CAAAGAGAAAAAGGTAGAGCAACTCTACCCAAATTTAGAGAAGCACAAGGAAGTATACCCCATTGTGAAT
TTACAAGCGGGGGGAAGGAGCTGGAAGGCAGTAGATTCTGTGGTCTTCCAACAGCTGCAAAATGTAGCAA
TGCAGCATGGACTTGTGTCCGAGGATTTTGAAAGGCAAATAGCATATTATGCCACCACATGGACAAGTAA
GGATATATTAGAAGTATTGGCCATGATGCCTGGGAACAGAGCACAGAAAGAATTAATACAAGGGAGATTA
AATGAAGAAGCAGAAAGGTGGGTAAGACAGAATCCGCCGGGGCCAAATGTCCTTACAGTGGATCAAATCA
TGGGAGTAGGACAAACAAATCACCAGGCGTCACAAGCTAATATGGATCAAGCGAGACAGATATGTTTGCA
ATGGGTAATATCAGCATTAAGATCAGTAAGGCATATGTCACATAGACCAGGGAACCCTATGTTAGTAAAA
CAAAAGAATAGTGAAAGCTATGAAGATTTTATAGCAAGACTATTAGAGGCCATAGATGCTGAACCAGTGA
CAGATCCTATAAAAACATATTTGAAAGTAACTCTGTCATATACAAATGCAAGCACAGATTGTCAAAAACA
AATGGACAGAGTATTAGGAACACGAGTACAGCAAGCAACAGTGGAAGAAAAAATGCAAGCATGTAGAGAT
GTGGGATCAGAAGGGTTCAAGATGCAGTTATTGGCGCAGGCATTAAGGCCAGAGAAAGGAGGAAATAAAG
GATCAGGACAAAAATGCTATAATTGTGGAAAACCAGGACATTTGGCAAGGCAGTGTAGGCAAGGCATAAT
ATGCCATCATTGTGGAAAAAGGGGACATATGCAAAAGGATTGTAGGAAAAAGAAAGGGGATATGAAGCAG
CAGGGAAACAGCAGGAGGGGGCCA

>GQ255399.1 Ovine progressive pneumonia virus isolate 76.3 gag protein (gag) gene, partial cds
GAGAAAAAGGGATACCCCGAGCTCAAGGAAGTGATTAAGGCAACATGTAAAATAAAAGTAGGGGCCGGGA
AGGAGACCTTGACAGAAGGGAACTGTCTATGGGCATTAAAAACCGTAGACTTTATATTTGAGGATATCAA
AACGGAGCCGTGGACTCTTACAAAGATGTATACAGTATGGGAAAGATTAAGGCAGTTAACTCCAGAAGAG
ACAAGTAAAAGAGAGTTTGCCTCCTTGCAAGCTACAATGGCTTGCCTAATGTGTAGTCAAATGGGCATGA
AGCCCGAGACGGTGCAGGCAGCACAGGGAATAATAGGTATAAAAGAAGGACTACGAGAAAATAAGGAGGA
CAAAGAGAAAAAGGTAGAGCAACTCTACCCAAATTTAGAGAAGCACAAGGAAGTATACCCCATTGTGAAT

TTACAAGCGGGGGGAAGGAGCTGGAAGGCAGTAGATTCTGTGGTCTTCCAACAGCTGCAAAATGTAGCAA
TGCAGCATGGACTTGTGTCCGAGGATTTTGAAAGGCAAATAGCATATTATGCCACCACATGGACAAGTAA
GGATATATTAGAAGTATTGGCCATGATGCCTGGGAACAGAGCACAGAAAGAATTAATACAAGGGAGATTA
AATGAAGAAGCAGAAAGGTGGGTAAGACAGAATCCGCCGGGGCCAAATGTCCTTACAGTGGATCAAATCA
TGGGAGTAGGACAAACAAATCACCAGGCGTCACAAGCTAATATGGATCAAGCGAGACAGATATGTTTGCA
ATGGGTAATATCAGCATTAAGATCAGTAAGGCATATGTCACATAGACCAGGGAACCCTATGTTAGTAAAA
CAAAAGAATAGTGAAAGCTATGAAGATTTTATAGCAAGACTATTAGAGGCCATAGATGCTGAACCAGTGA
CAGATCCTATAAAAACATATTTGAAAGTAACTCTGTCATATACAAATGCAAGCACAGATTGTCAAAAACA
AATGGACAGAGTATTAGGAACACGAGTACAGCAAGCAACAGTGGAAGAAAAAATGCAAGCATGTAGAGAT
GTGGGATCAGAAGGGTTCAAGATGCAGTTATTGGCGCAGGCATTAAGGCCAGAGAAAGGAGGAAATAAAG
GATCAGGACAAAAATGCTATAATTGTGGAAAACCAGGACATTTGGCAAGGCAGTGTAGGCAAGGCATAAT
ATGCCATCATTGTGGAAAAAGGGGACATATGCAAAAGGATTGTAGGAAAAAGAAAGGGGATATAAAGCAG
CAGGGAAACAGCAGGAGGGGGCCA

>M34193.1 Ovine lentivirus, complete genome
ATGGCGACGCAAGGCTCAAAGGAGAAGAAGGGATACCCTGA
GCTCAAAGAGGTCATTAAGACAACATGTAAAATAAAGGTGGGGCCCGGGAAGGAGACCTTGACAGAAGGG
AACTGTCTATGGGCCTTAAAAACCCTAGACTTTATATTTGAGGATATAAAAACGGAGCCGTGGACTCTTA
CAAAAATGTATACAGTCTGGGAAAAATTAAAGCAAGTAACTCCAGAAGAAACAAGTAAAAGAGAGTTTGC
CTCCTTACAGGCCACATTGGCTTGTATAATGTGTAGTCAAATGGGCATGAGGCCCGAGACAGTGCAGGCA
GCCAGGGGAATAATAAGTATGAAAGAAGGGCTACACGAAAAACAGGAGGATAAAGAAAAGAAGGTAGAAC
AACTCTACCCAAACTTGGAAAAACACAGAGAAGTGTATCCTATTGTAAATTTGCAGGCTGGAGGGAGAAG
TTGGAAAGCGGTAGAGTCAGTGACATTCCAGCAGCTGCAAACAGTAGCAATGCAGCATGGACTTGTGTCC
GAGGATTTTGAAAGACAATTAGCATATTATGCCACTACATGGACAAGCAAAGATATATTAGAAGTACTAG
CCATGATGCCTGGAAATAGGGCGCAAAAAGAGTTAATACAGGGGAAATTAAATGAGGAAGCAGAAAGGTG
GGTAAGGCAGAATCCCCCAGGGCCAAATGTCCTTACTGTGGATCAGATTATGGGAGTCGGACAAACAAAT
CAACAGGCATCGCAAGCTAATATGGATCAGGCAAGACAGCTTTGCTTGCAGTGGGTAATAACAGCCTTGA
GATCAGTAAGACATATGTCACACAGACCAGGAAATCCTATGCTGATAAAACAAAAGAATAGTGAAAGTTA
TGAAGATTTTATAGCAAGATTGCTAGAAGCAATTGATACAGAACCCGTCACGGATCCTATAAAAACATAT
TTAAAAGTAACTCTGTCGTTCACAAATGCTAGCACAGATTGTCAAAAACAAATGGACAGAGTGTTAGGGA
CAAGAGTCCAACAGGCATCAGTAGAAGAAAAAATGCAAGCATGTCGGGACGTAGGATCAGAAGGATTTAA
AATGCAGCTGTTAGCACAGGCATTGAGACCGCCACGGAAGGAAGGAAAACAGGGAGTACAAAAATGCTAT
TACTGCGGGAAACCAGGACATCTCGCAAGGCAATGCAGACAAGGAATAATATGTCATCATTGTGGGAAAA
GAGGACATATGCAAAAAGATTGCAGACAAAAGAAAGGTAATCCAACATCACAGCAGGGAAACAGCAGGAG
GGGGCCACGTGTGGTGCCGTCCGCGCCCCCTATGTTGTAA

>GQ255398.1 Ovine progressive pneumonia virus isolate 76.2 gag protein (gag) gene, partial cds
GAGGAAAAGGGATACCCCGAGCTCAAGGAAGTGATTAAGGCAACATGTAAAATAAAAGTAGGGGCCGGGA
AGGAGACCTTGACAGAAGGGAACTGTCTATGGGCATTAAAAACCGTAGACTTTATATTTGAGGATATCAA
AACGGAGCCGTGGACTCTTACAAAGATGTATACAGTATGGGAAAGATTAAGGCAGTTAACTCCAGAAGAG
ACAAGTAAAAGAGAGTTTGCCTCCTTGCAAGCTACAATGGCTTGCCTAATGTGTAGTCAAATGGGCATGA
AGCCCGAGACGGTGCAGGCAGCACAGGGAATAATAGGTATAAAAGAAGGACTACGAGAAAATAAGGAGGA
CAAAGAGAAAAAGGTAGAGCAACTCTACCCAAATTTAGAGAAGCACAAGGAAGTATACCCCATTGTGAAT
TTACAAGCGGGGGGAAGGAGCTGGAAGGCAGTAGATTCTGTGGTCTTCCAACAGCTGCAAAATGTAGCAA
TGCAGCATGGACTTGTGTCCGAGGATTTTGAAAGGCAAATAGCATATTATGCCACCACATGGACAAGTAA
GGATATATTAGAAGTATTGGCCATGATGCCTGGGAACAGAGCACAGAAAGAATTAATACAAGGGAGATTA
AATGAAGAAGCAGAAAGGTGGGTAAGACAGAATCCGCCGGGGCCAAATGTCCTTACAGTGGATCAAATCA
TGGGAGTAGGACAAACAAATCACCAGGCGTCACAAGCTAATATGGATCAAGCGAGACAGATATGTTTGCA
ATGGGTAATATCAGCATTAAGATCAGTAAGGCATATGTCACATAGACCAGGGAACCCTATGTTAGTAAAA
CAAAAGAATAGTGAAAGCTATGAAGATTTTATAGCAAGACTATTAGAGGCCATAGATGCTGAACCAGTGA
CAGATCCTATAAAAACATATTTGAAAGTAACTCTGTCATATACAAATGCAAGCACAGATTGTCAAAAACA
AATGGACAGAGTATTAGGAACACGAGTACAGCAAGCAACAGTGGAAGAAAAAATGCAAGCATGTAGAGAT
GTGGGATCAGAAGGGTTCAAGATGCAGTTATTGGCGCAGGCATTAAGGCCAGAGAAAGGAGGAAATAAAG
GATCAGGACAAAAATGCTATAATTGTGGAAAACCAGGACATTTGGCAAGGCAGTGTAGGCAAGGCATAAT
ATGCCATCATTGTGGAAAAAGGGGACATATGCAAAAGGATTGTAGGAAAAAGAAAGGGGATATAAAGCAG
CAGGGAAACAGCAGGAGGGGGCCA

>KY358787.1 Small ruminant lentivirus isolate USMARC-200303013-1, complete genome
ATGGCGAAGCAAGGCTCAAAGGAGAAAAAGGGATACCCCGAGCTCA

AGGAAGTGATTAAAGCTACATGTAAAATAAAGGTAGGGGCCGGGAAGGAGACCTTGACAGAAGGGAACTG
TCTATGGGCATTAAAGACTGTAGACTTTATATTTGAGGATATAAAGACAGAGCCGTGGACTCTTACAAAA
ATGTATACTGTATGGGACAGGCTAAAGCAGTTAACTCCGGAAGAGACAAGTAAAAGAGAGTTTGCCTCCT
TGCAAGCTACAATGGCTTGCTTAATGTGCAGTCAAATGGGCATGAAGCCTGAGACAGTGCAGGCAGCACA
GGGAATAATAGGTATGAAGGAAGGACTGCAAGAGAAAAAGGAGGAACAAGAAAAGAAGGTAGAGCAACTC
TACCCAAATTTAGAAAAGCATAAGGAAGTATACCCCATTGTGAATCTGCAAGCGGGGGGAAGGAGCTGGA
AGGCAGTAGACTCAGTGGTCTTCCAACAGCTGCAAAATGTAGCAATGCAGCATGGACTTGTGTCCGAGGA
TTTTGAAAGGCAAATAGCATATTATGCCACCACATGGACCAGTAAGGATATTTTAGAAGTATTGGCCATG
ATGCCTGGGAATAGAGCACAGAAAGAATTAATACAAGGGAAATTGAATGAAGAAGCAGAAAGATGGGTAA
GACAGAACCCGCCCGGGCCAGGTGTCCTTACAGTGGATCAAATCATGGGAGTAGGACAAACAAATCATCA
GGCATCACAAGCTAATATGGATCAAGCAAGGCAAGTATGCTTGCAATGGGTAATATCAGCATTAAGATCA
GTAAGACATATGTCACATAGACCAGGAAACCCTATGCTAGTAAAGCAGAAAAATAGTGAAAGTTATGAAG
ATTTCATCGCAAGACTACTAGAGGCCATAGATGCTGAGCCGGTAACAGACCCTATAAAAACATACTTGAA
AGTGACTCTGTCATATACAAATGCTAGCACGGATTGTCAAAAACAAATGGACAGAGTGATAGGAACAAGA
GTACAACAAGCCTCAGTAGAAGAGAAAATGCAAGCATGTAGAGATGTGGGATCAGAAGGGTTTAAGATGC
AACTATTAGCGCAGGCTCTAAGGCCAGAAAAAACCCCGGGAAATAGAGGACCAGGACAGAAATGTTATAA
TTGTGGAAAACCGGGACATTTGGCAAGGCAGTGTAGACAAGGCATAATATGTCATCATTGTGGAAAAAGA
GGACATATGCAAAAGGATTGCAGAAAAAAGAAAAGTGAGAACTTCAAGCAGCAGGGAAACAGCAGGAGGG
GGCCACGTGTGGTGCCGTCCGCGCCCCCTATGTTGTAA

>MZ313871.1 Visna-maedi virus isolate CMV-1, complete genome
ATGGCGAAGCAAGGCTCAAAGGAGAAAAAGGGAT
ACCCCGAGCTCAAGGAAGTAATTAAAGCAACATGTAAAATAAAGGTAGGGGCCGGGAAGGAGACCTTGAC
AGAAGGGAACTGTCTATGGGCATTAAAAACTGTAGACTTTATATTTGCAGATATAAAGACAGAACCGTGG
ACTCTTACAAAGATGTATACTGTATGGGACAGATTAAGGCAGTTGACTCCAGAAGAGACAAGTAAAAGAG
AGTTTGCCTCCTTGCAAGCTACATTGGCTTGCCTAATGTGTAGTCAAATGGGCATGAAGCCTGAGACAGT
GCAGGCAGCACGGGGAATAATAAGTATGAAGGAAGGACTACAAGAGAATAAGGAGGAAAAGAAGGTAGAA
CAACTCTACCCAAATTTAGAGAAGCATAAGGAAGTATACCCCATTGTGAATTTGCAAGCGGGGGGAAGGA
GTTGGAAGGCAGTAGACTCCGTAGTCTTCCAACAGCTGCAAAATGTAGCAATGCAGCATGGACTTGTGTC
CGAGGATTTTGAAAGGCAAATGGCATATTATGCCACCACATGGACAAGTAAAGATATATTAGAAGTATTG
GCCATGATGCCTGGAAATAGAGCTCAGAAAGAGTTAATACAAGGGAAATTAAATGAGGAAGCGGAGAGAT
GGATAAGGCAGAATCCCCCAGGGCCAAACGTCCTTACAGTGGATCACATTATGGGAGTAGGACAAACAAA
TCAACAGGCATCACAAGCTAACATGGATCAGGCAAGACAGATATGCTTGCAATGGGTAATAACCGCATTA
AGATCTGTAAGACATATGTCTCATAGGCCAGGAAATCCTATGCTGGTGAAGCAGAAAAATACAGAGAGTT
ATGAAGACTTCATAGCCAGACTGTTGGAAGCAATCGATGCAGAACCAGTCGCAGATCCTATAAAAACATA
CTTGAAAGTAACTCTGTCATATACAAATGCTAGCACAGATTGTCAAAAACAAATGGACAGAGTGTTAGGA
ACTAGAGTCCAACAAGCAACAGTAGAAGAAAAGATGCAAGCCTGTAGAGACGTAGGATCAGAAGGGTTTA
AAATGCAACTATTAGCGCAGGCTTTAAGACCAGAAAAGAGTGCAGGAAATCGGGGAGCAGGACAAAAATG
TTATAATTGTGGAAAACAGGGACATTTAGCAAGGCAGTGTAGGCAAGGCATAATATGCCATCATTGTGGA
AAAAGAGGACATATGCAAAAAGATTGCAGGAAAAAGAAAAGCGAGAACATGAAGCAGCAGGGAAACAGCA
GGAGGGGGCCACGTGTGGTGCCGTCCGCGCCCCCTATGCTGTAA

>GQ255402.1 Ovine progressive pneumonia virus isolate 76.5 gag protein (gag) gene, partial cds
GAGAAAAAGGGATACCCCGAGCTCAAGGAAGTGATTAAGGCAACATGTAAAATAAAAGTAGGGGCCGGGA
AGGAGACCTTGACAGAAGGGAACTGTCTATGGGCATTAAAAACCGTAGACTTTATATTTGAGGATATCAA
AACGGAGCCGTGGACTCTTACAAAGATGTATACAGTATGGGAAAGATTAAGGCAGTTAACTCCAGAAGAG
ACAAGTAAAAGAGAGTTTGCCTCCTTGCAAGCTACAATGGCTTGCCTAACGTGTAGTCAAATGGGCATGA
AGCCCGAGACGGTGCAGGCAGCACAGGGAATAATAGGTATAAAAGAAGGACTACGAGAAAATAAGGAGGA
CAAAGAGAAAAAGGTAGAGCAACTCTACCCAAATTTAGAGAAGCACAAGGAAGTATACCCCATTGTGAAT
TTACAAGCGGGGGGAAGGAGCTGGAAGGCAGTAGATTCTGTGGTCTTCCAACAGCTGCAAAATGTAGCAA
TGCAGCATGGACTTGTGTCCGAGGATTTTGAAAGGCAGATAGCATATTATGCCACCACATGGACAAGTAA
GGATATATTAGAAGTATTGGCCATGATGCCTGGGAACAGAGCACAGAAAGAATTAATACAAGGGAGATTA
AATGAAGAAGCAGAAAGGTGGGTAAGACAGAATCCGCCGGGGCCAAATGTCCTTACAGTGGATCAAATCA
TGGGAGTAGGACAAACAAATCACCAGGCGTCACAAGCTAATATGGATCAAGCGAGACAGATATGTTTGCA
ATGGGTAATATCAGCATTAAGATCAGTAAGGCATATGTCACATAGACCAGGGAACCCTATGTTAGTAAAA
CAAAAGAATAGTGAAAGCTATGAAGATTTTATAGCAAGACTATTAGAGGCCATAGATGCTGAACCAGTGA
CAGATCCTATAAAAACATATTTGAAAGTAACTCTGTCATATACAAATGCAAGCACAGATTGTCAAAAACA
AATGGACAGAGTATTAGGAACACGAGTACAGCAAGCAACAGTGGAAGAAAAAATGCAAGCATGTAGAGAT
GTGGGATCAGAAGGGTTCAAGATGCAGTTATTGGCGCAGGCATTAAGGCCAGAGAAAGGAGGAAATAAAG
GATCAGGACAAAAATGCTATAATTGTGGAAAACCAGGACATTTGGCAAGGCAGTGTAGGCAAGGCATAAT

ATGCCATCATTGTGGAAAAAGGGGACATATGCAAAAGGATTGTAGGAAAAAGAAAGGGGATATAAAGCAG
CAGGGAAACAGCAGGAGGGGGCCA

>GQ255400.1 Ovine progressive pneumonia virus isolate 76.4_(20AUG) gag protein (gag) gene, partial cds
GAGAAAAGGGGATACCCCGAGCTCAAGGAAGTAATTAAGGCAACATGTAAAATAAAAGTAGGGGCCGGGA
AGGAGACCTTGACAGAAGGGAACTGTCTATGGGCATTAAAAACCGTAGACTTTATATTTGAGGATATCAA
AACGGAGCCGTGGACTCTTACAAAGATGTATACAGTATGGGAAAGATTAAGGCAGTTAACTCCAGAAGAG
ACAAGTAAAAGAGAGTTTGCCTCCTTGCAAGCTACAATGGCTTGCCTAATGTGTAGTCAAATGGGCATGA
AGCCCGAGACGGTGCAGGCAGCACAGGGAATAATAGGTATAAAAGAAGGACTACGAGAAAATAAGGAGGA
CAAAGAGAAAAAGGTAGAGCAACTCTACCCAAATTTAGAGAAGCACAAGGAAGTATACCCCATTGTGAAT
TTACAAGCGGGGGGAAGGAGCTGGAAGGCAGTAGATTCTGTGGTCTTCCAACAGCTGCAAAATGTAGCAA
TGCAGCATGGACTTGTGTCCGAGGATTTTGAAAGGCAAATAGCATATTATGCCACCACATGGACAAGTAA
GGATATATTAGAAGTATTGGCCATGATGCCTGGGAACAGAGCACAGAAAGAATTATTACAAGGGAGATTA
ATTGAAGAAGCAGAAAGGTGGGTAAGACAGAATCCGCCGGGGCCAAATGTCCTTACAGTGGATCAAATCA
TGGGAGTAGGACAAACAAATCACCAGGCGTCACAAGCTAATATGGATCAAGCGAGACAGATATGTTTGCA
ATGGGTAATATCAGCATTAAGATCAGTAAGGCATATGTCACATAGACCAGGGAACCCTATGTTAGTAAAA
CAAAAGAATAGTGAAAGCTATGAAGATTTTATAGCAAGACTATTAGAGGCCATAGATGCTGAACCAGTGA
CAGATCCTATAAAAACATATTTGAAAGTAACTCTGTCATATACAAATGCAAGCACAGATTGTCAAAAACA
AATGGACAGAGTATTAGGAACACGAGTACAGCAAGCAACAGTGGAAGAAAAAATGCAAGCATGTAGAGAT
GTGGGATCAGAAGGGTTCAAGATGCAGTTATTGGCGCAGGCATTAAGGCCAGAGAAAGGAGGAAATAAAG
GATCAGGACAAAAATGCTATAATTGTGGAAAACCAGGACATTTGGCAAGGCAGTGTAGGCAAGGCATAAT
ATGCCATCATTGTGGAAAAAGGGGACATATGCAAAAGGATTGTAGGAAAAAAAAAGGGGATATAAAGCAG
CAGGGAAACAGCAGGAGGGGGCCA

>GQ255396.1 Ovine progressive pneumonia virus isolate 76.1 gag protein (gag) gene, partial cds
GAGAAAAAGGGATACCCCGAGCTCAAGGAAGTGATTAAGGCAACATGTAAAATAAAAGTAGGGGCCGGGA
AGGAGACCTTGACAGAAGGGAACTGTCTATGGGCATTAAAAACCGTAGACTTTATATTTGAGGATATCAA
AACGGAGCCGTGGACTCTTACAAAGATGTATACAGTATGGGAAAGATTAAGGCAGTTAACTCCAGAAGAG
ACAAGTAAAAGAGAGTTTGCCTCCTTGCAAGCTACAATGGCTTGCCTAATGTGTAGTCAAATGGGCATGA
AGCCCGAGACGGTGCAGGCAGCACAGGGAATAATAGGTATAAAAGAAGGACTACGAGAAAATAAGGAGGA
CAAAGAGAAAAAGGTAAAGCAACTCTACCCAAATTTAGAGAAGCACAAGGAAGTATACCCCATTGTGAAT
TTACAAGCGGGGGGAAGGAGCTGGAAGGCAGTAGATTCTGTGGCCTTCCAACAGCTGCAAAATGTAGCAA
TGCAGCATGGACTTGTGTCCGAGGATTTTGAAAGGCAAATAGCATATTATGCCACCACATGGACAAGTAA
GGATATATTAGAAGTATTGGCCATGATGCCTGGGAACAGAGCACAGAAAGAATTAATACAAGGGAGATTA
AATGAAGAAGCAGAAAGGTGGGTAAGACAGAATCCGCCGGGGCCAAATGTCCTTACAGTGGATCAAATCA
TGGGAGTAGGACAAACAAATCACCAGGCGTCACAAGCTAATATGGATCAAGCGAGACAGATATGTTTGCA
ATGGGTAATATCAGCATTAAGATCAGTAAGGCATATGTCACATAGACCAGGGAACCCTATGTTAGTAAAA
CAAAAGAATAGTGAAAGCTATGAAGATTTTATAGCAAGACTATTAGAGGCCATAGATGCTGAACCAGTGA
CAGATCCTATAAAAACATATTTGAAAGTAACTCTGTCATATACAAATGCAAGCACAGATTGTCAAAAACA
AATGGACAGAGTATTAGGAACACGAGTACAGCAAGCAACAGTGGAAGAAAAAATGCAAGCATGTAGAGAT
GTGGGATCAGAAGGGTTCAAGATGCAGTTATTGGCGCAGGCATTAAGGCCAGAGAAAGGAGGAAATAAAG
GATCAGGACAAAAATGCTATAATTGTGGAAAACCAGGACATTTGGCAAGGCAGTGTAGGCAAGGCATAAT
ATGCCATCATTGTGGAAAAAGGGGACATATGCAAAAGGATTGTAGGAAAAAGAAAGGGGATATAAAGCAG
CAGGGAAACAGCAGGAGGGGGCCA

>GQ255395.1 Ovine progressive pneumonia virus isolate 76.1_(20AUG) gag protein (gag) gene, partial cds
GAGAAAAAGGGATACCCCGAGCTCAAGGAAGTGATTAAGGCAACATGTAAAATAAAAGTAGGGGCCGGGA
AGGAGACCTTGACAGAAGGGAACTGTCTATGGGCATTAAAAACCGTAGACTTTATATTTGAGGATATCAA
AACGGAGCCGTGGACTCTTACAAAGATGTATACAGTATGGGAAAGATTAAGGCAGTTAACTCCAGAAGAG
ACAAGTAAAAGAGAGTTTGCCTCCTTGCAAGCTACAATGGCTTGCCTAATGTGTAGTCAAATGGGCATGA
AGCCCGAGACGGTGCAGGCAGCACAGGGAATAATAGGTATAAAAGAAGGACTACGAGAAAATAAGGAGGA
CAAAGAGAAAAAGGTAGAGCAACTCTACCCAAATTTAGAGAAGCACAAGGAAGTATACCCCATTGTGAAT
TTACAAGCGGGGGGAAGGAGCTGGAAGGCAGTAGATTCTGTGGTCTTCCAACAGCTGCAAAATGTAGCAA
TGCAGCATGGACTTGTGTCCGAGGATTTTGAAAGGCAAATAGCATATTATGCCACCACATGGACAAGTAA
GGATATATTAGAAGTATTGGCCATGATGCCTGGGAACAGAGCACAGAAAGAATTAATACCAGGGAGATTA
AATGAAGAAGCAGAAAGGTGGGTAAGACAGAATCCGCCGGGGCCAAATGTCCTTACAGTGGATCAAATCA
TGGGAGTAGGACAAACGAATCACCAGGCGTCACAAGCTAATATGGATCAAGCAAGACAGATATGTTTGCA
ATGGGTAATATCAGCATTAAGATCAGTAAGGCATATGTCACATAGACCAAGGAACCCTATGTTAGTAAAA

CAAAAGAATAGTGAAAGCTATGAAGATTTTATAGCAAGACTATTAGAGGCCATAGATGCTGAACCAGTGA
CAGATCCTATAAAAACATATTTGAAAGTAACTCTGTCATATACAAATGCAAGCACAGATTGTCAAAAACA
AATGGACAGAGTATTAGGAACACGAGTACAGCAAGCAACAGTGGAAGAAAAAATGCAAGCATGTAGAGAT
GTGGGATCAGAAGGGTTCAAGATGCAGTTATTGGCGCAGGCATTAAGGCCAGAGAAAGGAGGAAATAAAG
GATCAGGACAAAAATGCTATAATTGTGGAGAACCAGGACATTTGGCAAGGCAGTGTAGGCAAGGCATAAT
ATGCCATCATTGTGGAAAAAGGGGACATATGCAAAAGGATTGTAGGAAAAAGAAAGGGGATATAAAGCAG
CAGGGAAACAGCAGGAGGGGGCCA

>KR011757.1 Visna/maedi virus isolate Sichuan gag protein (gag) gene, complete cds
ATGGCGAAGCAAGGCTCAAAGGAGAAAAAGGGATACCCCGAGCTCAAGGAAGTAATTAAAGCAACATGTA
AAATAAAGGTAGGGGCCGGGAAGGAGACCTTGACAGAAGGGAACTGTTTATGGGCACTAAAAACTGTAGA
CTTTATATTTGAAGATATAAAGACAGAACCGTGGACTCTTACAAAGATGTATACTGTATGGGACAGATTA
AGACAGTTGACTCCAGAAGAGACAAGTAAAAGAGAGTTTGCCTCCTTGCAAGCTACATTGGCTTGCCTAA
TGTGTAGTCAAATGGGCATGAAGCCTGAGACAGTGCAGGCAGCACGGGGAATAATAAGTATGAAGGAAGG
ACTACAAGAGAATAAGGAGGAAAAAAAGGTAGAACAACTCTACCCAAATCTAGAGAAGCACAAGGAAGTA
TACCCCATTGTGAATTTGCAAGCGGGGGGAAGGAGTTGGAAGGCAGTAGACTCCGTAGTCTTCCAACAGC
TGCAAAATGTAGCAATGCAGCATGGACTTGTGTCCGAGGATTTTGAAAGGCAAATGGCATATTATGCCAC
CACATGGACAAGTAAAGATATATTAGAAGTATTGGCCATGATGCCTGGAAATAGAGCTCAGAAAGAGTTA
ATACAAGGGAAATTAAATGAGGAAGTGGAGAGATGGGTAAGGCAGAATCCCCCAGGGCCAAACGTCCTTA
CAGTGGATCACATTATGGGAGTAGGACAAACAAATCAACAGGCATCACAAGCTAACATGGATCAGGCAAG
ACAGATATGCTTGCAATGGGTAATAACCGCATTAAGATCTGTAAGACATATGTCTCATAGGCCAGGAAAT
CCTATGCTGGTGAAGCAGAAAAATACAGAGAGTTATGAAGACTTCATAGCCAGACTGTTGGAAGCAATCG
ATGCAGAACCAGTCGCAGATCCTATAAAAACATACTTGAAAGTAACTCTGTCATATACAAATGCTAGCAC
AGATTGTCAAAAACAAATGGACAGAGTGTTAGGAACTAGAGTCCAACAAGCAACAGTAGAAGAAAAGATG
CAAGCCTGTAGAGACGTAGGATCAGAAGGGTTTAAAATGCAACTATTAGCGCAGGCTTTAAGACCAGAAA
AGAGTGCAGGAAATCGGGGAGCAGGACAAAAATGTTATAATTGTGGAAAACCAGGACATTTAGCAAGGCA
GTGTAGGCAAGGCATAATATGCCATCATTGTGGAAAAAGAGGACATATGCAAAAAGATTGTAGGAAAAAG
AAAAGTGAGAACATGAAGCAGCAGGGAAACAGCAGGAGGGGGCCACGTGTGGTGCCGTCCGCGCCCCCTA
TGTTGTAA

>MT993900.1 Small ruminant lentivirus isolate USMARC-200050064-r, complete genome
ATGGCGAAGCAAGGCTCAAAGGAGAAAAAGGGATACCCCGAGCTCAA
GGAAGTGATTAAAGCTACATGTAAAATAAAGGTAGGGGCCGGGAAGGAGACCTTGACAGAAGGGAACTGT
CTATGGGCATTAAAGACTGTAGACTTTATATTTGAGGATATAAAGACAGAGCCGTGGACTCTTACAAAAA
TGTATACTGTATGGGACAGGCTAAAGCAGTTAACTCCGGAAGAGACAAGTAAAAGAGAGTTTGCCTCCTT
GCAAGCTACAATGGCTTGCTTAATGTGCAGTCAAATGGGCATGAAGCCTGAGACAGTGCAGGCAGCACGG
GGAATAATAGGCATGAAGGAAGGACTGCAAGAGAATAAGGAGGAACAAGAAAAGAAGGTAGAGCAACTCT
ACCCAAATTTAGAAAAGCATAAGGAAGTATACCCCATTGTGAATTTGCAAGCGGGGGGAAGGAGCTGGAA
GGCAGTAGACTCAGTGGTCTTCCAACAGCTGCAAAATGTAGCAATGCAGCATGGACTTGTGTCCGAGGAT
TTTGAAAGGCAAATAGCATATTATGCCACCACATGGACCAGTAAGGATATTTTAGAAGTATTGGCCATGA
TGCCTGGGAATAGAGCACAGAAAGAATTAATACAAGGGAAATTGAATGAAGAAGCAGAAAGATGGGTGAG
ACAGAACCCGCCAGGGCCAGGTGTCCTTACAGTGGATCAAATCATGGGAGTAGGACAAACAAATCATCAG
GCATCACAAGCTAATATGGATCAAGCAAGGCAAGTATGCTTGCAATGGGTAATATCAGCATTAAGATCAG
TAAGACATATGTCACATAGACCAGGAAACCCTATGCTAGTAAAGCAGAAAAATAGTGAAAGTTATGAAGA
GTTCATCGCAAGACTGCTAGAGGCCATAGATGCTGAGCCGGTAACAGACCCTATAAAAACATACTTGAAA
GTGACTCTGTCATATACAAATGCTAGCACGGATTGTCAAAAACAAATGGACAGAGTGATAGGAACAAGAG
TACAACAAGCCTCAGTAGAAGAGAAAATGCAAGCATGTAGAGATGTGGGATCAGAAGGGTTTAAGATGCA
ACTATTAGCGCAGGCTCTAAGGCCAGAAAAAACCCCAGGAAATAAAGGACCAGGACAGAAATGTTATAAT
TGTGGAAAACCGGGACATTTGGCAAGGCAGTGTAGACAAGGCATAATATGTCATCATTGTGGAAAAAGAG
GACATATGCAAAAGGATTGCAGAAAAAAGAAAAGTGAGAACTTCAAGCAGCAGGGAAACAGCAGGAGGGG
GCCACGTGTGGTGCCGTCCGCGCCCCCTATGTTGTAA

>JN184351.1 Small ruminant lentivirus isolate 72 gag protein (gag) gene, complete cds
ATGGCGAAGCAAGGCTCAAGGGAGAAAAAGGGATACCCCGAGCTCAAGGAGGTTATAAAAGCGACATGTA
AAATAAAAATTGGGCCAGGGAAGGAGACCTTGACAGAAGGGAATTGTCTATGGGCTCTAAAAACTGTAGA
CTTTATATTTGAGGATTTAAAAACAGAGCCATGGACGTTAACAAAAATGTATACAGTATGGGGAAGATTA
AAAAAGTTAACTCCAGAGGAGACAAGCAGGAGAGAATTTGCTTCGTTGCAAGCCACGATGGCTTGTATAA
TATGTAGTCAAATGGGAATGAAGCCGGAAACAGTGCAGGCAGCTAGAGGAATAATAAGTATGAAAGAAGG

GCTACAAGAAGCAGAGGAGGAAAAAGAGAAAAAAGTAGAGCAGCTTTATCCAAACTTAGAGAGACATAGG
GAGGTATATCCTATAGTAAATCTGCAAGCAGGGGGAAGGAGTTGGAAGGCTGTAGATTCAGTAGTCTTCC
AGCAGCTGCAAACGGTTGCAATGCAGCATGGACTTGTGTCCGAGGATTTTGAGAGGCAAATGGCATATTA
TGCTACCACTTGGACTAGTAAAGATATATTAGAAGTCCTAGCCATGATGCCTGGGAACAGAGCACAAAAA
GAGTTGATCCAAGGGAAGTTAAATGAGGAAGCAGAAAGATGGGTAAGGCAAAATCCACCGGGACCAAATG
TCCTCACAGTGGATCAGATTATGGGAGTAGGACAAACAAATCAGCAGGCATCTCAAGCGAATATGGATCA
GGCAAGACAAATTTGCCTGCAGTGGGTTATAAATGCCCTGAGGTCAGTAAGGCATATGTCACATAGACCA
GGAAACCCTATGCTGGTAAAGCAAAAGAACAATGAGAGTTATGAAGATTTTATAGCAAGACTGTTGGAAG
CAATTGATGCAGAACCAGTAACAGACCCTATAAAAACATATTTGAAGGTAACACTGTCATATACAAATGC
TAGTACAGATTGTCAGAAACAAATGGACAGAGTATTAGGCCAGAGGGTCCAACAGGCAACAGTAGAAGAA
AAAATGCAAGCTTGCCGCGATGTAGGGTCGGAAGGATTTAAAATGCAGTTGCTAGCACAAGCCTTGCGAC
CCGAGAGAAAAAATCAAGGGGGACAACAGAAATGCTATAATTGTGGAAAACCAGGACATCTTGCAAGGCA
GTGTAGGCAAGGAATAATATGTCATCATTGTGGTAAGAAGGGACATATGCAAAAAGACTGCAGACAGAAA
AAACAGCAGGGAAACAACAGGAGGGGGCCACGTGTGGTGCCGTCCGCGCCCCCTATGCTGTAA

>HQ848062.1 Visna/maedi virus isolate 697, complete genome
ATGGCGAAGCAAGGCTCAAAGGAGAAAAAGGGATACCCCGAGCTCAAAGAGGCTATAAAAGCA
ACATGTAAAATAAAGATTGGGCCAGGGAAGGAGACCTTGACAGAAGGGAATTGTCTATGGGCTTTAAAAA
CTGTAGACTTCATATTTGAAGACTTAAAAGCAGAGCCATGGACACTAACAAAAATGTACACAGTATGGGG
GAGATTAAAAAAGTTAACTCCAGAGGAAACAAGCAAAAGGGAATTTGCTTCATTGCAAGCCACAATGGCA
TGTTTAATGTGTAGCCAAATGGGGATGAAGCCAGAGACAGTACAGGCAGCTAGGGGAATACTGAGTATGA
AGGAAGGGCTACAAGAAACAGAAGAAGAAAAAGAGAAAAAGGTAGAACAGCTCTACCCAAACTTAGAGAG
GCACAGAGAAGCGTACCCTATAGTAAATTTACAAGCAGGGGGAAGAAGCTGGAAGGCTGTAGATTCAGTA
GTCTTTCAGCAGCTGCAAACAGTGGCAATGCAGCATGGACTTGTGTCCGAGGATTTTGAAAGGCAATTAG
CATATTATGCTACTACTTGGACTAGTAAAGATATATTAGAAGTATTAGCTATGATGCCTGGAAACAGGGC
GCAAAAAGAATTAATCCAAGGGAAATTAAATGAAGAGGCAGAGAGATGGGTGAGGCAGAATCCACCAGGG
CCGAATGTCCTCACCGTGGATCAAATTATGGGAGTGGGACAAACAAATCAGCAAGCGTCCCAGGCTAATA
TGGATCAAGCAAGACAGATCTGCCTGCAATGGGTAATAAACGCCTTGAGATCGGTGAGACATATGTCACA
TAGGCCAGGAAATCCCATGTTAGTAAAGCAAAAGAACAATGAGAGTTATGAAGATTTTATAGCAAGACTG
TTGGAAGCAATTGATGCAGAGCCAGTCGCAGATCCTATAAAGACATATTTAAAAGTGACGCTGTCATATA
CAAATGCTAGTACAGATTGTCAGAAACAAATGGACAGGGTATTAGGCACTAGGGTTCAACAAGCAACAGT
AGAAGAAAAAATGCAGGCATGCCGAGACGTGGGATCGGAAGGATTTAAAATGCAGTTATTAGCACAAGCT
TTGAGGCCAGAAAGGAAGGCAAGAAATCAGGAGATTGGGCAAAAATGTTATAATTGTGGGAAGCCAGGAC
ATCTTGCGAGGCAGTGTAGACAGGGAATAATATGTCATCATTGTGGAAAAAGGGGACATATGCAAAAAGA
TTGCAGACAGAAGAAACAACAGGGAAACACCAAGAGGGGGCCACGTGTGGTGCCGTCCGCGCCCCCTATG
TTGTAA

>MT993906.1 Small ruminant lentivirus isolate USMARC-200117502-r, complete genome
ATGGCGAAGCAAGGCTCAAAGGAGAAAAAGGGATACCCCGAGCTCAAGGAAGTGATTAAAGC
TACATGTAAAATAAAGGTAGGGGCCGGGAAGGAGACCTTGACAGAAGGGAACTGTCTATGGGCATTAAAG
ACTGTAGACTTTATATTTGAGGATATAAAGACAGAGCCGTGGACTCTTACAAAAATGTATACTGTATGGG
ACAGGCTAAAGCAGTTAACTCCGGAAGAGACAAGTAAAAGAGAGTTTGCCTCCTTGCAAGCTACAATGGC
TTGCTTAATGTGCAGTCAAATGGGCATGAAGCCTGAGACAGTGCAGGCAGCACGGGGAATAATAGGCATG
AAGGAAGGACTGCAAGAGAATAAGGAGGAACAAGAAAAGAAGGTAGAGCAACTCTACCCAAATTTAGAAA
AGCATAAGGAAGTATACCCCATTGTGAATTTGCAAGCGGGGGGAAGGAGCTGGAAGGCAGTAGACTCAGT
GGTCTTCCAACAGCTGCAAAATGTAGCGATGCAGCATGGACTTGTGTCCGAGGATTTTGAAAGGCAAATA
GCATATTATGCCACCACATGGACCAGTAAGGATATTTTAGAAGTATTGGCCATGATGCCTGGGAATAGAG
CACAGAAAGAATTAATACAAGGGAAATTGAATGAAGAAGCAGAAAGATGGGTGAGACAGAACCCGCCAGG
GCCAGGTGTCCTTACAGTGGATCAAATCATGGGAGTAGGACAAACAAATCATCAGGCATCACAAGCTAAT
ATGGATCAAGCAAGGCAAGTATGTTTGCAATGGGTAATATCAGCATTAAGATCAGTAAGACATATGTCAC
ATAGACCAGGAAACCCTATGCTAGTAAAGCAGAAAAATAGTGAAAGTTATGAAGATTTCATCGCAAGACT
GCTAGAGGCCATAGATGCTGAGCCGGTAACAGACCCTATAAAAACATATTTGAAAGTAACTCTGTCATAT
ACAAATGCTAGCACGGATTGTCAAAAACAAATGGACAGAGTGATAGGAACAAGAGTACAACAAGCCTCAG
TAGAAGAGAAAATGCAAGCATGTAGAGATGTGGGATCAGAAGGGTTTAAGATGCAACTATTAGCGCAGGC
TCTAAGGCCAGAAAAAACCCCGGGAAATAGAGGATCAGGACAGAAATGTTATAATTGTGGAAAACCGGGA
CATTTGGCAAGGCAGTGTAGACAAGGTATAATATGTCATCATTGTGGAAAAAGAGGACATATGCAAAAAG
ATTGTAGAAAAAAGAAAAGTGAGAACTTCAAGCAGCAGGGAAACAGCAGGAGGGGGCCACGTGTGGTGCC
GTCCGCGCCCCCTATGTTGTAA

>GQ255425.1 Ovine progressive pneumonia virus isolate 101.3 gag protein (gag) gene, partial cds
GAGAAAAAGGGATACCCCGAGCTCAAGGAAGTGATTAAGGCAACATGTAAAATAAGAGTAGGGGCCGGGA
AGGAGACCTTGACAGAAGGGAACTGTCTATGGGCATTAAAGACTGTAGACTTTATATTTGAGGATATAAA
AACAGAGCCGTGGACTCTTACAAAGATGTATACAGTATGGGGAAGATTAAAGCAGTTAACTCCAGAAGAG
ACAAGTAAAAGAGAGTTTGCCTCCTTACAAGCTACAATGGCTTGCATCATGTGTAGTCAAATGGGCATGA
AGCCTGAGACAGTGCAGGCAGCACAGGGAATAATAAGTATGAAAGAAGGACTACGAGAAAATAAGGAGGA
CAAAGAAAAGGAGGTAGAGCAACTCTACCCCAATCTAGAGAAGCACAGAGAAGTGTACCCCATTGTGAAT
TTACAAGCGGGGGGAAGGAGTTGGAAGGCAGTAGATTCTGTGGTCTTCCAACAGCTGCAAAATGTAGCAA
TGCAGCATGGACTTGTGTCCGAGGATTTTGAAAGGCAAATAGCATATTATGCCACCACATGGACAAGTAA
GGACATATTAGAAGTGTTGGCCATGATGCCTGGAAACAGAGCACAAAAAGAATTAATACAGGGGAGATTA
AATGAAGAAGCAGAAAGGTGGGTGAGACAGAATCCGCCGGGGCCAAATGTCCTTACAGTGGATCAAATCA
TGGGAGTAGGACAAACAAATCACCAGGCATCACAAGCTAATATGGATCAAGCAAGGCAAATATGTTTGCA
ATGGGTAATATCAGCCTTAAGGTCAGTAAGGCATATGTCACATAGACCAGGAAACCCAATGCTAGTAAAG
CAAAAGAATAGTGAAAGCTATGAAGATTTTATAGCAAGACTATTAGAGGCCATAGATGCTGAGCCAGTGA
CAGATCCTATAAAAACATATTTGAAAGTAACTCTGTCATATACAAATGCAAGCACAGACTGTCAAAAACA
AATGGACAGAGTATTAGGAACACGGGTACAGCAAGCGTCAGTAGAAGAAAAAATGCAAGCATGTAGAGAT
GTGGGATCGGAAGGGTTCAAGATGCAGTTGTTGGCGCAGGCATTAAGGCCAGAAAAAAATCCAGGAAATA
AAGGATTAGGACAAAAATGCTATAATTGTGGAAAACCAGGACATTTGGCAAGACAATGTAGGCAAGGCAT
AATATGCCATCATTGTGGAAAAAGAGGACATATGCAAAAGGATTGTAGAAAAAAGAAAAGTGATACAAGG
CAGCAGGGAAACAGCAGGAGGGGGCCA

>JN184358.1 Small ruminant lentivirus isolate 368 gag protein (gag) gene, complete cds
ATGGCGAAGCAAGGCTCAAAGGAGAAAAAGGGATACCCCGAGCTCAAGGAGGCGATAAAAGCAACATGCA
AAATAAAAATTGGGCCAGGGAAGGAGACCTTGACGGAAGGGAATTGTCTGTGGGCCTTAAAAACTGTAGA
CTTCATATTTGAAGATATAAAAACAGAGCCATGGACAATAACAAAAATGTATACTGTGTGGGGAAGATTG
AAAAAGTTAACTCCAGAGGAAACAAGCAAGAGGGAATTTGCTTCGTTGCAGGCCACAATGGCTTGTTTGA
TGTGTAGCCAAATGGGTATGAAACCCGAGACAGTACAGGCAGCAAGGGGAATAATAAGTATGAAAGAAGG
GCTACAAGAAATAGAAGAAGACAAAGAGAAAAAGGTGGAGCAACTCTACCCAAACTTAGAGAAACATAGA
GAAGTATACCCTATTGTGAATTTGCAAGCAGGAGGAAGAAGTTGGAAGGCGGTAGAGTCAGTAACCTTCC
AGCAGCTGCAAAATGTAGCCATGCAGCATGGACTAGTGTCCGAGGATTTTGAGAGGCAGTTAGCATATTA
TGCTACTACTTGGACAAGTAAGGATATTCTAGAAGTACTGGCCATGATGCCTGGGAATAGAGCGCAGAAA
GAGTTAATACAGGGAAAGTTAAATGAAGAGGCAGAAAGATGGGTGAGACAGAATCCACCCGGCCCAAATG
TCCTAACAGTGGATCAAATCATGGGAGTAGGACAAACAAATCAACAGGCATCCCAGGCTAATATGGATCA
AGCAAGGCAGATATGCTTGCAGTGGGTAATAACAGCATTAAGATCAGTTAGGCATATGTCACACAGGCCA
GGGAATCCTATGCTGGTAAAGCAAAAGAACACAGAAAGTTATGAAGAATTCATAGCAAGATTGCTCGAGG
CAATTGATGCAGAGCCAGTGACAGAGCCTATAAAAACATATTTAAAAGTGACTCTATCGTACACAAATGC
TAGTACAGATTGTCAGAAACAAATGGACAGAGTCTTGGGAACTAGGGTGCAGCAAGCAACAGTAGAAGAG
AAGATGCAAGCATGCAGAGATGTAGGATCAGAAGGATTCAAGATGCAACTATTAGCACAAGCTTTACGAC
CTCCAAGAAAAGGAAGAAATGAAGGTCCAGGTCAAAAATGTTATAATTGTGGGAAAATGGGACATCTTGC
AAGACAATGCAGACAAGGGATAATATGCCATCTATGTGGGAAAAGAGGACATATGAAAAAAGACTGTCGA
CAAAAGAAAAAATGATGGCATGCCAACACAGGGAAACAACAGGAGGGGGCCACGTGTGGTGCCGTCCGCGC
CCCCTATGCTGTAA

>GQ255397.1 Ovine progressive pneumonia virus isolate 76.2_(20AUG) gag protein (gag) gene, partial cds
GAGAAAAAGGGATACCCCGAGCTCAAGGAAGTGATTAAGGCAACATGTAAAATAAAAATAGGGGCCGGGA
AGGAGACCTTGACAGAAGGGAACTGTCTATGGGCATTAAAAACCGTAGATTTTATATTTGAGGATATCAA
AACGGAGCCGTGGACTCTTACAAAGATGTATACAGTATGGGAAAGATTAAGGCAGTTAACTCCAGAAGAG
ACGAGTAAAAAAGAGTTTGCCTCCTTGCAAGCTACAATGGCTTGCCTAATGTGTAGTCAAATGGGCATGA
AGCCCGAGACGGTGCAGGCAGCACAGGGAATAATAGGTATAAAAGAAGGACTACGAGAAAATAAGGAGGA
CAAAGAGAAAAAGGTAGAGCAACTCTACCCAAATTTAGAGAAGCACAAGGAAGTATACCCCATTGTGAAT
TTACAAGCGGGGGGAAGGAGCTGGAAGGCAGTAGATTCTGTGGCCTTCCAACAGCTGCAAAATGTAGCAA
TGCAGCATGGACTTGTGTCCGAGGATTTTGAAAGGCAAATAGCATATTATGCCACCACATGGACAAGTAA
GGATATATTAGAAGTATTGGCCATGATGCCTGGGAACAGAGCACAGAAAGAATTAATACAAGGGAGATTA
AATGAAGAAGCAGAAAGGTGGGTAAGACAGAATCCGCCGGGGCCAAATGTCCTTACAGTGGATCAAATCA
TGGGAGTAGGACAAACAAATCACCAGGCGTCACAAGCTAATATGGATCAAGCGAGACAGATATGTTTGCA
ATGGGTAATATCAGCATTAAGATCAGTAAGGCATATGTCACATAGACCAGGGAACCCTATGTTAGTAAAA
CAAAAGAATAGTGAAAGCTATGAAGATTTTATAGCAAGACTATTAGAGGCCATAGATGCTGAACCAGTGA
CAGATCCTATAAAAACATATTTGAAAGTAACTCTGTCATATACAAATGCAAGCACAGATTGTCAAAAACA

AATGGACAGAGTATTAGGAACACGAGTACAGCAAGCAACAGTGGAAGAAAAAATGCAAGCATGTAGAGAT
GTGGGATCAGAAGGGTTCAAGATGCAGTTATTGGCGCAGGCATTAAGGCCAGAGAAAGGAGGAAATAAAG
GATCAGGACAAAAATGCTATAATTGTGGAAAACCAGGACATTTGGCAAGGCAGTGTAGGCAAGGCATAAT
ATGCCATCATTGTGGAAAAAGGGGACATATGCAAAAGGATTGTAGGAAAAAGAAAGGGGATATAAAGCAG
CAGGGAAACAGCAGGAGGGGGCCA

>MT993904.1 Small ruminant lentivirus isolate USMARC-200023230-1, complete genome
ATGGCGAAGCAAGGCTCAAAGGAGAAAAAGGGATACCCCGAGCTCAAGGAAGTGAT
TAAAGCTACATGTAAAATAAAGGTAGGGGCCGGGAAGGAGACCTTGACAGAAGGGAACTGTCTATGGGCA
TTAAAGACTGTAGACTTTATATTTGAGGATATAAAGACAGAGCCGTGGACTCTTACAAAAATGTATACTG
TATGGGATAGGCTAAAGCAGTTAACTCCAGAAGAGACAAGTAAAAGAGAGTTTGCCTCCTTGCAAGCTAC
AATGGCTTGCTTAATGTGCAGTCAAATGGGCATGAAGCCTGAGACAGTGCAGGCAGCACGGGGAATAATA
GGCATGAAGGAAGGACTGCAAGAGAATAAGGAGGAACAAGAAAAGAAGGTAGAGCAACTCTACCCAAATT
TAGAAAAGCATAAGGAAGTATACCCCATTGTGAATTTGCAAGCGGGGGGAAGGAGCTGGAAGGCAGTAGA
CTCAGTGGTCTTCCAACAGCTGCAAAATGTAGCGATGCAGCATGGACTTGTGTCCGAGGATTTTGAAAGG
CAAATAGCATATTATGCCACCACATGGACCAGTAAGGATATTTTAGAAGTATTGGCCATGATGCCTGGGA
ATAGAGCACAGAAAGAATTAATACAAGGGAAATTGAATGAAGAAGCAGAAAGATGGGTGAGACAGAACCC
GCCAGGGCCAGGTATCCTTACAGTGGATCAAATCATGGGAGTAGGACAAACAAATCATCAGGCATCACAA
GCTAATATGGATCAAGCAAGGCAAGTATGCTTGCAATGGGTAATATCAGCATTAAGATCAGTAAGACATA
TGTCACATAGACCAGGAAACCCTATGCTAGTAAAGCAGAAAAATAGTGAAAGTTATGAAGATTTCATCGC
AAGACTGCTAGAGGCCATAGATGCTGAGCCGGTAACAGACCCTATAAAAACATACTTGAAAGTGACTTTG
TCATATACAAATGCTAGCACGGATTGTCAAAAACAAATGGACAGAGTGATAGGAACAAGAGTACAACAAG
CCTCAGTAGAAGAGAAAATGCAAGCATGTAGAGATGTGGGATCAGAAGGGTTTAAGATGCAACTATTAGC
GCAGGCTCTAAGGCCAGAAAAAAACCCCGGGAAATAGAGGACCAGGACAGAAATGTTATAATTGTGGAAAA
CCGGGACATTTGGCAAGGCAGTGTAGACAAGGCATAATATGTCATCATTGTGGAAAAAGAGGACATATGC
AAAAGGATTGCAGAAAAAAGAAAAGTGAGAACTTCAAGCAGCAGGGAAACAGCAGGAGGGGGCCACGTGT
GGTGCCGTCCGCGCCCCCTATGTTGTAA

>MT993903.1 Small ruminant lentivirus isolate USMARC-199835918-1, complete genome
ATGGCGAAGCAAGGCTCAAAGGAGAAAAAGGGATACCCCGAGCTCAAGGAAGTGATT
AAAGCTACATGTAAAATAAAGGTAGGGGCCGGGAAGGAGACCTTGACAGAAGGGAACTGTCTATGGGCAT
TAAAGACTGTAGACTTTATATTTGAGGATATAAAGACAGAGCCGTGGACTCTTACAAAAATGTGTACTGT
ATGGGGCAGGCTAAAGCAGTTAACTCCGGAAGAGACAAGTAAAAGAGAGTTTGCCTCCTTGCAAGCTACA
ATGGCTTGCTTAATGTGCAGTCAAATGGGCATGAAGCCTGAGACAGTGCAGGCAGCACAGGGAATAATAA
GCATGAAGGAAGGACTGCAAGAGAATAAGGAGGAACAAGAAAAGAAGGTAGAGCAACTCTACCCAAATTT
AGAGAAGCATAAGGAAGTATACCCCATTGTGAATTTGCAAGCGGGGGGAAGGAGCTGGAAGGCAGTAGAC
TCAGTGGTCTTCCAACAGCTGCAAAATGTAGCGATGCAGCATGGACTTGTGTCCGAGGATTTTGAAAGGC
AAATAGCATATTATGCCACCACATGGACCAGTAAGGATATTTTAGAAGTATTGGCCATGATGCCTGGGAA
TAGAGCACAGAAAGAATTAATACAAGGGAAATTGAATGAAGAAGCAGAAAGATGGGTGAGACAGAACCCG
CCAGGGCCAGGTGTCCTTACAGTGGATCAAATCATGGGAGTAGGACAAACAAATCATCAGGCATCACAAG
CTAATATGGATCAAGCAAGGCAAGTATGCTTGCAATGGGTAATATCAGCATTAAGATCAGTAAGACATAT
GTCACATAGACCAGGAAACCCTATGCTAGTAAAGCAGAAAAATAGTGAAAGTTATGAAGATTTCATCGCA
AGACTGCTAGAGGCCATAGATGCTGAGCCGGTAACAGACCCTATAAAAACATACTTGAAAATGACTCTGT
CTTATACAAATGCTAGCACGGATTGTCAAAAACAAATGGACAGAGTGATAGGAACAAGAGTACAACAAGC
CTCAGTAGAAGAGAAAATGCAAGCATGTAGAGATGTGGGATCAGAAGGGTTTAAGATGCAACTATTAGCG
CAGGCTCTAAGGCCAGAAAAAAACCCCGGGAAATAGAGGAACAGGACAGAAATGTTATAATTGTGGAAAAC
CGGGACATTTGGCAAGGCAGTGTAGACAAGGCATAATATGTCATCATTGTGGAAAAAGAGGACATATGCA
AAAGGATTGCAGAAAAAAGAAAAGTGAGAACTTCAAGCAGCAGGGAAACAGCAGGAGGGGGCCACGTGTG
GTGCCGTCCGCGCCCCCTATGTTGTAA

>MT993901.1 Small ruminant lentivirus isolate USMARC-200323455-1, complete genome
ATGGCGAAGCAAGGCTCAAAGGAGAAAAAGGGATACCCCGAGCTCAAGGAAGTGA
TTAAAGCTACATGTAAAATAAAGGTAGGGGCCGGGAAGGAGACCTTGACAGAAGGGAACTGTCTATGGGC
ATTAAAGACTGTAGACTTTATATTTGAGGATATAAAGACAGAGCCGTGGACTCTTACAAAAATGTATACT
GTATGGAACAGGCTAAAGCAGTTAACTCCGGAAGAGACAAGTAAAAGAGAGTTTGCCTCCTTGCAAGCTA
CAATGGCTTGCTTAATGTGCAGTCAAATGGGCATGAAGCCTGAGACAGTGCAGGCAGCACGGGGAATAAT
AGGCATGAAGGAAGGACTGCAAGAGAATAAGGAGGAACAAGAAAAGAAGGTAGAGCAACTCTACCCAAAT
TTAGAAAAGCATAAGGAAGTATACCCCATTGTGAATTTGCAAGCGGGGGGAAGGAGCTGGAAGGCAGTAG

ACTCAGTGGTCTTCCAACAGCTGCAAAATGTAGCAATGCAGCATGGACTTGTGTCCGAGGATTTTGAAAG
GCAAATAGCATATTATGCCACCACATGGACCAGTAAGGATATTTTAGAAGTATTGGCCATGATGCCTGGG
AATAGAGCACAGAAAGAATTAATACAAGGGAAATTGAATGAAGAAGCAGAAAGATGGGTGAGACAGAACC
CGCCAGGGCCAGGTGTCCTTACAGTGGATCAAATCATGGGAGTAGGACAAACAAATCATCAGGCATCACA
AGCTAATATGGATCAAGCAAGGCAAGTATGCTTGCAATGGGTAATATCAGCATTGAGATCAGTAAGACAT
ATGTCACATAGACCAGGAAACCCTATGCTAGTAAAGCAGAAAAATAGTGAAAGTTATGAAGATTTCATCG
CAAGACTGCTAGAGGCCATAGATGCGGAGCCGGTAACAGACCCTATAAAAACATACTTGAAAATGACTCT
GTCATATACAAATGCTAGCACGGATTGTCAAAAACAAATGGACAGAGTGATAGGAACAAGAGTACAACAA
GCCTCAGTAGAAGAGAAAATGCAAGCATGTAGAGATGTGGGATCAGAAGGGTTTAAGATGCAACTATTAG
CGCAGGCTCTAAGGCCAGAAAAAACCCCGGGAAATAAAGGACCAGGACAGAAATGTTATAATTGTGGAAA
ACCGGGACATTTGGCAAGGCAGTGTAGACAAGGCATAATATGTCATCATTGTGGAAAAAGAGGACATATG
CAAAAGGATTGCAGAAAAAAGAAAAGTGAGAACTTCAAGCAGCAGGGAAACAGCAGGAGGGGGCCACGTG
TGGTGCCGTCCGCGCCCCCTATGTTGTAA

>MT993899.1 Small ruminant lentivirus isolate USMARC-200103515-1,
complete genome
ATGGCGAAGCAAG
GCTCAAAGGAGAAAAAGGGATACCCCGAGCTCAAGGAAGTGATTAAAGCTACATGTAAAATAAAGGTAGG
GGCCGGGAAGGAGACCTTGACAGAAGGGAACTGTCTATGGGCATTAAAGACTGTAGACTTTATATTTGAG
GATATAAAGACAGAGCCGTGGACTCTTACAAAAATGTATACTGTATGGGACAGGCTAAAGCAGTTAACTC
CGGAAGAGACAAGTAAAAGAGAGTTTGCCTCCTTGCAAGCTACAATGGCTTGCTTAATGTGCAGTCAAAT
GGGCATGAAGCCTGAGACAGTGCAGGCAGCACGGGGAATAATAGGCATGAAGGAAGGACTGCAAGAGAAT
AAGGAGGAACAAGAAAAGAAGGTAGAGCAACTCTACCCAAATTTAGAAAAGCATAAGGAAGTATACCCCA
TTGTGAATTTGCAAGCGGGGGGAAGGAGCTGGAAGGCAGTAGACTCAGTGGTCTTCCAACAGCTGCAAAA
TGTAGCGATGCAGCATGGACTTGTGTCCGAGGATTTTGAAAGGCAAATAGCATATTATGCCACCACATGG
ACCAGTAAGGATATTTTAGAAGTATTGGCCATGATGCCTGGGAATAGAGCACAGAAAGAATTAATACAAG
GGAAATTGAATGAAGAAGCAGAAAGATGGGTGAGACAGAACCCGCCAGGGCCAGGTGTCCTTACAGTGGA
TCAAATCATGGGAGTAGGACAAACAAATCATCAGGCATCACAAGCTAATATGGATCAAGCAAGGCAAGTA
TGCTTGCAATGGGTAATATCAGCATTAAGATCAGTAAGACATATGTCACATAGACCAGGAAACCCTATGC
TGGTAAAGCAGAAAAATAGTGAAAGTTATGAAGATTTCATTGCAAGACTACTAGAGGCCATAGATGCTGA
GCCGGTAACAGACCCTATAAAAACATACTTGAAAGTGACTCTGTCATATACAAATGCTAGCACGGATTGT
CAAAAACAAATGGACAGAGTGATAGGAACAAGAGTACAACAAGCCTCAGTAGAAGAGAAAATGCAAGCAT
GTAGAGATGTGGGATCAGAAGGGTTTAAGATGCAACTATTAGCGCAGGCTCTAAGGCCAGAAAAAACCCC
GGGAAATAGAGGACCAGGACAGAAATGTTATAATTGTGGAAAACCGGGACATTTGGCAAGGCAGTGTAGA
CAAGGCATAATATGTCATCATTGTGGAAAAAGAGGACATATGCAAAAGGATTGCAGAAAAAAGAAAAGTG
AGAACTTCAAGCAGCAGGGAAACAGCAGGAGGGGGCCACGTGTGGTGCCGTCCGCGCCCCCTATGTTGTA
A

>GQ255427.1 Ovine progressive pneumonia virus isolate 101.5 gag
protein (gag) gene, partial cds
GAGAAAAAGGGATACCCCGAGCTCAAGGAAGTGATTAAGGCAACATGTAAAATAAGAGTAGGGGCCGGGA
AGGAGACCTTGACAGAAGGGAACTGTCTATGGGCATTAAAGACTGTAGACTTTATATTTGAGGATATAAA
AACAGAGCCGTGGACTCTTACAAAGATGTATACAGTATGGGGAAGATTAAAGCAGTTAACTCCAGAAGAG
ACAAGTAAAAGAGAGTTTGCCTCCTTACAAGCTACAATGGCTTGCATCATGTGTAGTCAAATGGGCATGA
AGCCTGAGACAGTGCAGGCAGCACAGGGAATAATAGGTATGAAAGAAGGACTACGAGAAAATAAGGAGGA
CAAAGAAAAGGAGGTAGAGCAACTCTACCCCAATCTAGAGAAGCACAGAGAAGTGTACCCCATTGTGAAT
TTACAAGCGGGGGGAAAGAGTTGGAAGGCAGTAGATTCTGTGGTCTTCCAACAGCTGCAAAATGTAGCAA
TGCAGCATGGACTTGTGTCCGAGGATTTTGAAAGGCAAATAGCATATTATGCCACCACATGGACAAGTAA
GGACATATTAGAAGTGTTGGCCATGATGCCTGGAACCAGAGCACAAAAAGAATTAATACAGGGGAAATTA
AATGAAGAAGCAGAAAGGTGGGTGAGACAGAATCCGCCGGGGCCAAATGTCCTTACAGTGGATCAAATCA
TGGGAGTAGGACAAACAAATCACCAGGCATCACAAGCTAATATGGATCAAGCAAGGCAAATATGTTTGCA
ATGGGTAATATCAGCCTTAAGGTCAGTAAGGCATATGTCACATAGACCAGGAAACCCAATGCTAGTAAAG
CAAAAGAATAGTGAAAGCTATGAAGATTTTATAGCAAGACTATTAGAGGCCATAGATGCTGAGCCAGTGA
CAGATCCTATAAAAACATATTTGAAAGTAACTCTGTCATATACAAATGCAAGCACAGACTGTCAAAAACA
AATGGACAGAGTATTAGGAACACGGGTACAGCAAGCGTCAGTAGAAGAAAAAATGCAAGCATATAGAGAT
GTGGGATCGGAAGGGTTCAAGATGCAGTTGTTGGCGCAGGCATTAAGGCCAGAAAAAAATCCAGGAAATA
AAGGATTAGGACAAAAATGCTATAATTGTGGAAAACCAGGACATTTGGCAAGACAATGTAGGCAAGGCAT
AATATGCCATCATTGTGGAAAAAGAGGACATATGCAAAAGGATTGTAGAAAAAAGAAAAGTGATACAAGG
CAGCAGGGAAACAGCAGGAGGGGGCCA

>MT993902.1 Small ruminant lentivirus isolate USMARC-200103342-1, complete genome
ATGGCGAAGCAAGGCTCAAAGGAGAAAAAGGGATACCCCGAGCTCAAGGAAGTG
ATTAAAGCTACATGTAAAATAAAGGTAGGGGCCGGGAAGGAGACCTTGACAGAAGGGAACTGTCTATGGG
CATTAAAGACTGTAGACTTTATATTTGAGGATATAAAGACAGAGCCGTGGACTCTTACAAAAATGTATAC
TGTATGGGACAGGCTAAAGCAGTTAACTCCGGAAGAGACAAGTAAAAGAGAGTTTGCCTCCTTGCAAGCT
ACAATGGCTTGCTTAATGTGCAGTCAAATGGGCATGAAGCCTGAGACAGTGCAGGCAGCACGGGGAATAA
TAGGCATGAAGGAAGGACTGCAAGAGAATAAGGAGGAACAAGAAAAGAAGGTAGAGCAACTCTACCCAAA
TTTAGAAAAGCATAAGGAAGTATACCCCATTGTGAATCTGCAAGCGGGGGGAAGGAGCTGGAAGGCAGTA
GACTCAGTGGTCTTCCAACAGCTGCAAAATGTAGCGATGCAGCATGGACTTGTGTCCGAGGATTTTGAAA
GGCAAATAGCATATTATGCCACCACATGGACCAGTAAGGATATTTTAGAAGTATTGGCCATGATGCCTGG
GAATAGAGCACAGAAAGAATTAATACAAGGGAAATTGAATGAAGAAGCAGAAAGATGGGTGAGACAGAAC
CCGCCAGGGCCAGGTGTCCTTACAGTGGATCAAATCATGGGAGTAGGACAAACAAATCATCAGGCATCAC
AAGCTAATATGGATCAAGCAAGGCAAGTATGCTTGCAATGGGTAATATCAGCATTAAGATCAGTAAGACA
TATGTCACATAGACCAGGAAACCCTATGCTAGTAAAGCAGAAAAATAGTGAAAGTTATGAAGATTTCATC
GCAAGACTGCTAGAGGCCATAGATGCTGAGCCGGTAACAGACCCTATAAAAACATACTTGAAAGTGACTC
TGTCATATACAAATGCTAGCACGGATTGTCAAAAACAAATGGACAGAGTGATAGGAACAAGAGTACAACA
AGCCTCAGTAGAAGAGAAAATGCAAGCATGTAGAGATGTGGGATCAGAAGGGTTTAAGATGCAACTATTA
GCGCAGGCTCTAAGGCCAGAAAAAACCCCGGGGAATAGAGGACCAGGACAGAAATGTTATAATTGTGGAA
AACCGGGACATTTGGCAAGGCAGTGTAGACAAGGCATAATATGTCATCATTGTGGAAAAAGAGGACATAT
GCAAAAGGATTGCAGAAAAAAGAAAAGTGAGAACTTCAAGCAGCAGGGAAACAGCAGGAGGGGGCCACGT
GTGGTGCCGTCCGCGCCCCCTATGTTGTAA

>MT993898.1 Small ruminant lentivirus isolate USMARC-200303332-1, complete genome
ATGGCGAA
GCAAGGCTCAAAGGAGAAAAAGGGATACCCCGAGCTCAAGGAAGTGATTAAAGCTACATGTAAAATAAAG
GTAGGGGCCGGGAAGGAGACCTTGACAGAAGGGAACTGTCTATGGGCATTAAAGACTGTAGACTTTATAT
TTGAGGATATAAAGACAGAGCCGTGGACTCTTACAAAAATGTATACTGTATGGGACAGGCTAAAGCAGTT
AACTCCGGAAGAGACAAGTAAAAGAGAGTTTGCCTCCTTGCAAGCTACAATGGCTTGCTTAATGTGCAGT
CAAATGGGCATGAAGCCTGAGACAGTGCAGGCAGCACGGGGAATAATAGGCATGAAGGAAGGACTGCAAG
AGAATAAGGAGGAACAAGAAAAGAAGGTAGAGCAACTCTACCCAAATCTAGAAAAGCATAAGGAAGTATA
CCCCATTGTGAATTTGCAAGCGGGGGGAAGAAGCTGGAAGGCAGTAGACTCAGTAGTCTTCCAACAGCTG
CAAAATGTAGCGATGCAGCATGGACTTGTGTCCGAGGATTTTGAAAGGCAAATAGCATATTATGCCACCA
CATGGACCAGTAAGGATATTTTAGAAGTATTAGCCATGATGCCTGGGAATAGAGCACAGAAAGAATTAAT
ACAAGGGAAATTGAATGAAGAAGCAGAAAGATGGGTAAGACAGAACCCGCCGGGGCCAGGTGTCCTTACA
GTGGATCAAATCATGGGAGTAGGACAAACAAATCATCAGGCATCACAAGCTAATATGGATCAAGCAAGGC
AAGTATGCTTGCAATGGGTAATATCAGCATTAAGATCAGTAAGACATATGGCACATAGACCAGGAAACCC
TATGCTAGTAAAGCAGAAAAATAGTGAAAGTTATGAAGATTTCATCGCAAGACTACTAGAGGCCATAGAT
GCTGAGCCGGTAACAGACCCTATAAAAACATACTTGAAAATGACTCTGTCATATACAAATGCTAGCACGG
ATTGTCAAAAACAAATGGACAGAGTAATAGGAACAAGAGTACAACAAGCCTCAGTAGAAGAGAAAATGCA
AGCATGTAGAGATGTGGGATCAGAAGGGTTTAAGATGCAACTATTAGCGCAGGCTCTAAGGCCAGAAAAA
ACCCCGGGAAATAGAGGACCAGGACAGAAATGTTATAATTGTGGAAAACTGGGACATTTGGCAAGGCAGT
GTAGACAAGGCATAATATGTCATCATTGTGGAAAAAGAGGACATATGCAAAAGGATTGCAGAAAAAAGAA
AAGTGAGAACTTCAAGCAGCAGGGAAACAGCAGGAGGGGGCCACGTGTGGTGCCGTCCGCGCCCCCTATG
TTGTAA

>MT993897.1 Small ruminant lentivirus isolate USMARC-200303038-1, complete genome
ATGGCGAAGCAAGGCTCAAAGGAGAAAAAGGGATACCCCGAGCTCAAGGAAGTGATT
AAAGCTACATGTAAAATAAAGGTAGGGGCCGGGAAGGAGACCTTGACAGAAGGGAACTGTCTATGGGCAT
TAAAGACTGTAGACTTTATATTTGAGGATATAAAGACAGAGCCGTGGACTCTTACAAAAATGTATACTGT
ATGGGACAGGCTAAAGCAGTTAACTCCGGAAGAGACAAGTAAAAGAGAGTTTGCCTCCTTACAAGCTACA
ATGGCTTGCTTAATGTGCAGTCAAATGGGCATGAAGCCTGAGACAGTGCAGGCAGCACGGGGAATAATAG
GCATGAAGGAAGGACTGCAAGAGAATAAGGAGGAACAAGAAAAGAAGGTAGAGCAACTCTACCCAAATCT
AGAAAAGCATAAGGAAGTATACCCCATTGTGAATTTGCAAGCGGGGGGAAGAAGCTGGAAGGCAGTAGAC
TCAGTAGTCTTCCAACAGCTGCAAAATGTAGCGATGCAGCATGGACTTGTGTCCGAGGATTTTGAAAGGC
AAATAGCATATTATGCCACCACATGGACCAGTAAGGATATTTTAGAAGTATTGGCCATGATGCCTGGGAA
TAGAGCACAGAAAGAATTAATACAAGGGAAATTGAATGAAGAAGCAGAAAGATGGGTAAGACAGAACCCG
CCGGGGCCAGGTGTCCTTACAGTGGATCAAATCATGGGAGTAGGACAAACAAATCATCAGGCATCACAAG
CTAATATGGATCAAGCAAGGCAAGTATGCTTGCAATGGGTAATATCAGCATTAAGATCAGTAAGACATAT

GTCACATAGACCAGGAAACCCTATGCTAGTAAAACAGAAAAATAGTGAAAGTTATGAAGATTTCATCGCA
AGACTACTAGAGGCCATAGATGCTGAGCCGGTAACAGACCCTATAAAAACATACTTGAAAATGACTCTGT
CATATACAAATGCTAGCACGGATTGTCAAAAACAAATGGACAGAGTAATAGGAACAAGAGTACAACAAGC
CTCAGTAGAAGAGAAAATGCAAGCATGTAGAGATGTGGGATCAGAAGGGTTTAAGATGCAACTATTAGCG
CAGGCTCTAAGGCCAGAAAAAAACCCCGGGAAATAGAGGACCAGGACAGAAATGTTATAATTGTGGAAAAC
TGGGACATTTGGCAAGGCAGTGTAGACAAGGCATAATATGTCATCATTGTGGAAAAAGAGGACATATGCA
AAAGGATTGCAGAAAAAAGAAAAGTGAGAACTTCAAGCAGCAGGGAAACAGCAGGAGGGGGCCACGTGTG
GTGCCGTCCGCGCCCCCTATGTTGTAA

>GQ255431.1 Ovine progressive pneumonia virus isolate 10.9 gag protein (gag) gene, partial cds
GAGAAAAAGGGATACCCCGAGCTCAAGGAGGTGATTAAAGCAACATGTAAAATAAAAGTAGGGGCCGGGA
AGGAGACCTTGACAGAAGGGAACTGTCTATGGGCACTAAAGACTATAGACTTTATATTTGAAGATATAAA
AACGGAACCGTGGACTCTTACAAAGATGTATACTGTATGGGGTAGATTACAGCAGTTAACTCCAGAAGAG
ACAAGTAAAAGAGAGTTTGCCTCCTTGCAAGCTACAATGGCTTGTATAATGTGTAGTCAACTGGGCATGC
AGCCTGAGACAGTGCAGGCAGCACGGGGAATAATAAGTATGAAAGACGGACTGCACGGAAACAAGGAGGA
CAAAGAGAAGGAGGTAGAGCAACTCTACCCAAATTTAGAGAAACACAAGGAAGTATACCCTATTGTGAAT
TTGCAAGCAGGGGGAAGGAGTTGGAAGGCAGTAGACTCAGTGGTCTTCCAACAGCTGCAAAATGTAGCAA
TGCAGCATGGACTTGTGTCCGAGGATTTTGAAAGGCAAGTGGCATATTATGCCACTACATGGACAAGCAA
GGATATATTAGAGGTATTGGCCATGATGCCTGGGACCAGAGCACAGAAAGAATTAATACAAGGAAAATTA
AATGAAGAAGCGGAAAGGTGGGTGAGACAAAATCCGCCAGGACCAAATGTCCTCACAGTGGATCAGATCA
TGGGAGTGGGACAGACAAACCATCAGGCATCACAAGCCAACATGGATCAAGCAAGACAAATATGCTTGCA
ATGGGTAATATCAGCATTAAGGTCAGTAAGGCATATGTCACATAGACCAGGGAATCCTATGTTAGTAAAA
CAGAAGAACAATGAAAGCCATGAAGATTTTATAGCAAGACTATTAGAGGCCATAGATGCTGAGCCAGTGA
CAGATCCTATAAAAACATATTTGAAAGTAACTCTGTCATATACGAATGCAAGCACCGATTGTCGAAAACA
AATGGACAGAGTAATAGGAACTCGAGTACAGCAAGCCACAGTAGAAGAAAAAATGCAAGCATGTAGAGAT
GTGGGATCAGAAGGGTTTAAGATGCAGTTATTGGCACAAGCATTAAGGCCAGAGAAAAATCCAGGAAATA
GAGGAGCAGGACAAAAATGCTATAATTGTGGAAAACTAGGACATTTGGCAAGGCAGTGTAGGCAAGGCAT
AATATGCCATCATTGTGGAAAAAGAGGACATATGCAGAGGGATTGCAGAAGAAAGAAAAGTGATAACGTC
AAGCAGCAGGGAAACAGCAGGAGGGGGCCA

>GQ255424.1 Ovine progressive pneumonia virus isolate 101.2 gag protein (gag) gene, partial cds
GAGAAAAAGGGATACCCCGAGCTCAAGGAAGTGATTAAGGCAACATGTAAAATAAGAGTAGGGGCCGGGA
AGGAGACCTTGACAGAAGGGAACTGTCTATGGGCATTAAAGACTGTAGACTTTATATTTGAGGATATAAA
AACAGAGCCGTGGACTCTTACAAAGATGTATACAGTATGGGGAAGATTAAAGCAGTTAACTCCAGAAGAG
ACAAGTAAAAGAGAGTTTGCCTCCTTACAAGCTACAATGGCTTGCATAATGTGTAGTCAAATGGGCATGA
AGCCTGAGACAGTGCAGGCAGCACAGGGAATAATAGGTATGAAAGAAGGACTACGAGAAAATAAGGAGGA
CAAAGAAAAGGAGGTAGAGCAACTCTACCCCAATCTAGAGAAGCACAGAGAAGTGTACCCCATTGTGAAT
TTACAAGCGGGGGGAAGGAGTTGGAAGGCAGTAGATTCTGTGGTCTTCCAACAGCTGCAAAATGTAGCAA
TGCAGCATGGACTTGTGTCCGAGGATTTTGAAAGGCAAATAGCATATTATGCCACCACATGGACAAGTAA
GGACATATTAGAAGTGTTGGCCATGATGCCTGGAACCAGAGCACAAAAAGAATTAATACAGGGGAGATTA
AATGAAGAAGCAGAAAGGTGGGTGAGACAGAATCCGCCGGGGCCAAATGTCCTTACAGTGGATCAAGTCA
TGGGAGTAGGACAAACAAATCACCAGGCATCACAAGCTAATATGGATCAAGCAAGGCAAATATGTTTGCA
ATGGGTAATATCAGCCTTAAGGTCAGTAAGGCATATGTCACATAGACCAGGAAACCCAATGCTAGTAAAG
CAAAAGAATAGTGAAAGCTATGAAGATTTTATAGCAAGACTATTAGAGGCCATAGATGCTGAGCCAGTGA
CAGATCCTATAAAAACATATTTGAAAGTAACTCTGTCATATACAAATGCAAGCACAGACTGTCAAAAACA
AATGGACAGAGTATTAGGAACACGGGTACAGCAAGCGTCAGTAGAAGAAAAAATGCAAGCCTGTAGAGAT
GTGGGATCGGAAGGGTTCAAGATGCAGTTGTTGGCGCAGGCATTAAGGCCAGAAAAAAATCCAGGAAATA
AAGGATTAGGACAAAAATGCTATAATTGTGGAAAACCAGGACATTTGGCAAGACAATGTAGGCAAGGCAT
AATATGCCATCATTGTGGAAAAAGAGGACATATGCAAAAGGATTGTAGAAAAAAGAAAAGTGATACAAGG
CAGCAGGGAAACAGCAGGAGGGGGCCA

>GQ255423.1 Ovine progressive pneumonia virus isolate 101.1 gag protein (gag) gene, partial cds
GAGAAAAAGGGATACCCCGAGCTCAAGGAAGTGATTAAGGCAACATGTAAAATAAGAGTAGGGGCCGGGA
AGGAGACCTTGACAGAAGGGAACTGTCTATGGGCATTAAAGACTGTAGACTTTATATTTGAGGATATAAA
AACAGAGCCGTGGACTCTTACAAAGATGTATACAGTATGGGGAAGATTAAAGCAGTTAACTCCAGAAGAG
ACAAGTAAAAGAGAGTTTGCCTCCTTACAAGCTACAATGGCTTGCATAATGTGTAGTCAAATGGGCATGA
AGCCTGAGACAGTGCAGGCAGCACAGGGAATAATAGGTATGAAAGAAGGACTACGAGAAAATAAGGAGGA
CAAAGAAAAGGAGGTAGAGCAACTCTACCCCAATCTAGAGAAGCACAGAGAAGTGTACCCCATTGTGAAT

TTACAAGCGGGGGGAAGGAGTTGGAAGGCAGTAGATTCTGTGGTCTTCCAACAGCTGCAAAATGTAGCAA
TGCAGCATGGACTTGTGTCCGAGGATTTTGAAAGGCAAATAGCATATTATGCCACCACATGGACAAGTAA
GGACATATTAGAAGTGTTGGCCATGATGCCTGGAACCAGAGCACAAAAAGAATTAATACAGGGGAGATTA
AATGAAGAAGCAGAAAGGTGGGTGAGACAGAATCCGCCGGGGCCAAATGTCCTTACAGTGGATCAAATCA
TGGGAGTAGGACAAACAAATCACCAGGCATCACAAGCTAATATGGATCAAGCAAGGCAAATATGTTTGCA
ATGGGTAATATCAGCCTTAAGGTCAGTAAGGCATATGTCACATAGACCAGGAAACCCAATGCTAGTAAAG
CAAAAGAATAGTGAAAGCTATGAAGATTTTATAGCAAGACTATTAGAGGCCATAGATGCTGAGCCAGTGA
CAGATCCTATAAAAACATATTTGAAAGTAACTCTGTCATATACAAATGCAAGCACAGACTGTCAAAAACA
AATGGACAGAGTATTAGGAACACGGGTACAGCAAGCGTCAGTAGAAGAAAAAATGCAAGCCTGTAGAGAT
GTGGGATCGGAAGGGTTCAAGATGCAGTTGTTGGCGCAGGCATTAAGGCCAGAAAAAAAATCCAGGAAATA
AAGGATTAGGACAAAAATGCTATAATTGTGAAAAACCAGGACATTTGGCAAGACAATGTAGGCAAGGCAT
AATATGCCATCATTGTGGAAAAAGAGGACATATGCAAAAGGATTGTAGAAAAAAGAAAAGTGATACAAGG
CAGCAGGGAAACAGCAGGAGGGGGCCA

>GQ255422.1 Ovine progressive pneumonia virus isolate 99.9 gag protein (gag) gene, partial cds
GAGAAAAAGGGATACCCCGAGCTCAAGGAGGTGATTAAAGCAACATGTAAAATAAAAGTAGGGGCCGGGA
AGGAGACCTTGACAGAAGGGAACTGTCTATGGGCATTAAAGACTATAGACTTTATATTTGAGGATATAAA
AACGGAACCGTGGACTCTTACAAAGATGTATACTGTATGGGGTAGATTAAAGCAGTTAACTCCAGAAGAG
ACAAGTAAAAGAGAGTTTGCCTCCTTGCAAGCTACAATGGCTTGTATGATGTGTAGTCAGATGGGCATGA
AGCCTGAGACAGTGCAGGCAGCACGGGGAATAATAGAGATGAAAGAAGGACTGCACAGAAACAAGGAGGA
CAAAGAGAAGGAGGTGGAGCAACTCTACCCAAATCTAGAAAAACACAAGGAGGTATACCCTATTGTGAAT
TTGCAAGCAGGGGGAAGGAGTTGGAAGGCAGTAGACTCAGTGGTCTTCCAACAGCTGCAAAATGTAGCAA
TGCAGCATGGACTTGTGTCCGAGGATTTTGAAAGGCAAGTGGCATATTATGCCACTACATGGACAAGTAA
GGATATATTAGAGGTATTGGCCATGATGCCTGGGAACAGAGCACAGAAAGAATTAATACAAGGAAAATTA
AATGAAGAAGCGGAAAGGTGGGTGAGACAAAATCCGCCAGGGCCAAATGTCCTCACAGTGGATCAGATCA
TGGGAGTAGGACAGACAAATCATCAGGCATCACAAGCCAACATGGATCAAGCAAGGCAAATATGCTTGCA
ATGGGTAATATCAGCGTTACGGTCAGTAAGACATATGTCCCATAGACCAGGGAACCCAATGTTAATAAAG
CAGAAGAATAGTGAAAGCTATGAAGATTTTATAGCAAGACTATTAGAGGCCATAGATGCTGAGCCCGTGA
CAGATCCTATAAAAACATATTTGAAAGTAACTCTGTCATATACAAATGCAAGCACAGACTGTCAAAAACA
AATGGACAGAGTATTAGGAACACGAGTACAGCAAGCATCAGTGGAAGAAAAAATGCAAGCATGTAGAGAT
GTGGGATCAGAAGGGTTCAAGATGCAGCTATTGGCACAAGCATTAAGGCCAGAGAAAAATCCAGGAAATA
GAGGAGCAGGACAAAAATGCTATAATTGTGGAAAACTAGGACATTTGGCAAGGCAGTGTAGGCAAGGCAT
AATATGCCATCATTGTGGAAAAAGAGGACATATGCAGAGGGATTGCAGAAAAAAGAAAAGTGATAACATC
AAGCAGCAGGGAAACAGCAGGAGGGGGCCA

>AY101611.1 Visna virus strain 85/34 from USA Gag polyprotein (gag), pol polyprotein (pol), virion infectivity protein (vif), and transcriptional activator (tat) mRNAs, complete cds; and envelope glycoprotein (env) mRNA, partial cds
ATGGCGAAGCAAGGCTCAAAGGAGAAAAAGGGATACCC
CGAGCTTAAGGAAGTAATTAGAGCAACCTGCAAAATAAAGGTAGGGGCCGGGAAGGAGACCTTGACAGAA
GGGAACTGTCTATGGGCATTAAAAACTGTAGACTTTATATTTGAAGATATAAAGACAGAACCGTGGACTC
TTACAAAGATGTATACTGTATGGGACAGACTAAGGCAGTTGACTCCAGAAGAGACAAGTAAAAGAGAGTT
TGCCTCCTTGCAAGCTACAATGGCTTGCCTAATGTGTAGTCAAATGGGCATGAAGCCCGAGACAGTGCAG
GCAGCACGGGGAATAATAAGTATGAAGGAAGGACTACAAGAAAATAAGGAGGAAAAGAAGGTAGAACAAC
TCTACCCAAATTTAGAGAAGCACAAGGAAGTATTCCCCATTGTGAATTTGCAAGCGGGGGGTAGGAGTTG
GAAGGCAGTAGAGTCAGTAGTCTTCCAACAGCTGCAAAATGTAGCAATGCAGCATGGACTTGTGTCCGAG
GATTTTGAAAGGCAAATGGCATATTATGCCACCACATGGACAAGTAAAGATATTTTGGAAGTATTGGCCA
TGATGCCTGGAAATAGAGCTCAGAAAGAGTTAATACAAGGGAAATTAAATGAAGAAGCGGAGAGATGGGT
AAGGCAGAATCCCCCAGGGCCAAATGTCCTTACAGTGGACCAAATTATGGGAGTAGGACAAACAAATCAA
CAGGCATCACAAGCTAACATGGATCAGGCAAGACAAATATGCTTGCAATGGGTAATAACAGCATTGAGAT
CTGTAAGACATATGTCTCATAGGCCAGGAAATCCTATGCTAGTAAAGCAGAAAAATACAGAGAGTTATGA
AGACTTCATAGCGAGACTGTTGGAAGCAATCGATGCAGAACCAGTCACAGATCCTATAAAAACATACTTG
AAAGTAACTCTGTCATATACAAATGCTAGCGCAGATTGTCAAAAACAAATGGACAGAGTGTTAGGAACTA
GAGTCCAGCAAGCAACAGTGGAAGAAAAGATGCAAGCATGTAGAGATGTGGGATCAGAAGGATTTAAAAT
GCAGCTATTAGCACAAGCTTTAAGGCCAGAGAAAAACCCGGGGAATAGGGGACCAGGACAAAAATGTTAT
AATTGTGGAAAACCGGGACATTTAGCGAGGCAGTGCCGGCAAGGGATAATATGCCATCATTGTGGAAAGA
GGGGGCATATGCAAAAAGATTGCCGGCAAAAGAAGAACAAGGATATGAAGCAGCAGGGAAACAGCAGGAG
GGGGCCACGTGTGGTGCCGTCCGCGCCCCCTATGTTGTAA

>EU010123.1 Small ruminant lentivirus isolate It-017.16g03 gag protein gene, complete cds
ATGGCGAAGCAAGGCTCAAGAGAGGGGAAAAAGGGATACCCCGAGCTCAAAGAGGCAATTAAGGCCACAT
GTAAAATAAGGGTAGGAGCTGGGAAGGAGACCTTGACAGAAGGGAACTGTCTATGGGCATTAAAGACCAT
AGGCTTTATCTTTGAAGATATAGCCGCAGAGCCATGGACGCTCACAAAAATGTATGCTGTATGGGGGAGA
TTAAAGCAGCTAACTCCAGAAGAAACAAGTAAGAGAGAGTTTGCCTCTTTGCAAGCAACATTAGCTTGTG
TAATGTGTAGTCAAATGGGGATGAAGCCCGAGACAGTGCAGGCAGCAAGAGGGATAATAGGCATGAAAGA
GGGACTACAGGAGAATAAAGAGGCAGCAGAGAAGAAGGTAGAGCAACTCTACCCAAATGTAGAGAAACAT
AATGAGGTGTACCCTATTGTGAATTTGCAAGCAGGGGGGAGAGCATGGAAGGCTGTAGAGTCAGTAGTCT
TCCAGCAATTGCAGACAGTAGCCATGCAACACGGGCTAGTGTCTGAAGATTTCGAGAGGCAGTTGGCATA
TTATGCTACTACCTGGACTAGTAAGGATATATTGGAGGTACTGGCCATGATGCCTGGGAATAGAGCACAG
AAGGAATTAATACAAGGAAAATTGAATGAAGAAGCAGAAAGATGGGTAAGGCAAAATCCTCCGGGACAGA
ATGTCCTAACGGTGGATCAAATTATGGGAGTAGGACAAACAAATCAGCAGGCAGCCCAGGCTAATATGGA
TCAAGCAAGGCAGATATGTTTGCAATGGGTAATAACAGCATTAAGATCAGTAAGGCATATGTCGCATAGA
CCAGGAAACCCTATGCTAGTCAAGCAGAAGAATAATGAGAGCTATGAAGATTTCATAGCTAGACTATTAG
AAGCAATTGATGCAGAACCTGTTACTGAGCCTATAAAAACATATCTAAAGGTGACTCTGTCGTACACAAA
TGCGAGTTCAGATTGTCAAAAGCAAATGGACAGAGTGTTGGGAAGTAGGGTACAACAGGCAACGGTAGAA
GAGAAAATGCAAGCCTGTCGGGACGTAGGATCAGAAGGATTTAGAATGCAGCTGTTGGCACAAGCTTTAA
GGCCTGAGAAGAGGCCAGGAAACCCAGGGGAAAAGCAAAAGTGCTATAATTGTGGAAAACCAGGACATTT
AGCAAGACAATGCAGACAAGGAATAATATGCCATTATTGTGGAAAAAGGGGACATCTACAAAAGGATTGC
AGACAAAAGAAAAACAATAGCACAAAACAGCAGGGAAACGGGAGGCGGGGGATACGTGTGCTGCCG

>GQ255429.1 Ovine progressive pneumonia virus isolate 10.3 gag protein (gag) gene, partial cds
GAGAAAAAGGGATACCCCGAGCTCAAGGAAGTAATTAAGGCAACATGTAAAATAAAAGTAGGAGCCGGGA
AGGAGACCTTGACGGAGGGAAACTGTCTATGGGCATTAAAAACTGTAGACTTTATATTTGAGGATATAAA
AACAGAACCGTGGACTCTTACAAAGATGTATACAGTATGGGGAAGATTAAAGCAGTTAACTCCAGAAGAG
ACAAGTAAAAGAGAGTTTGCCTCCTTACAAGCTACAATGGCTTGCATAATGTGTAGTCAAATGGGCATGA
AGCCTGAGACAGTGCAGGCAGCACAGGGAATAATAGGTATGAAAGAAGGACTACGCGAAAATAAGGAGGA
CAAAGAAAAGGAGGTAGAGCAACTCTACCCAAATCTAGAGAAGCACAGAGAAGTATACCCCATTGTGAAT
TTACAAGCGGGGGGAAGAAGTTGGAAGGCAGTAGATTCTGTGGTCTTCCAACAGCTGCAAAATGTAGCAA
TGCAGCATGGACTTGTGTCCGAGGATTTTGAAAGGCAACTAGCATATTATGCCACCACATGGACAAGTAA
GGATATATTAGAAGTATTGGCCATGATGCCTGGGAACAGGGCACAGAAAGAATTAATACAAGGGAAATTA
AATGAAGAAGCAGAAAGGTGGGTAAGACAGAATCCGCCGGGGCCAAATGTCCTTACAGTGGATCAGATCA
TGGGAGTAGGACAGACAAATCACCAGGCATCACAAGCTAATATGGATCAAGCAAGACAGATATGTTTACA
ATGGGTAATATCAGCATTAAGGTCAGTAAGACATATGTCACATAGACCAGGGAACCCAATGTTAATAAAG
CAGAAGAATAGTGAAAGCTATGAAGATTTTATAGCAAGACTATTAGAGGCCATAGATGCTGAGCCAGTGA
CAGATCCTATAAAAACATATTTAAAAGTAACTCTGTCATATACGAATGCAAGCACAGACTGTCAAAAACA
AATGGACAGAGTATTAGGAACACGAGTACAACAAGCATCAGTGGAAGAAAAAATGCAAGCATGTAGAGAT
GTGGGGTCAGAAGGGTTCAAGATGCAGTTGTTGGCGCAAGCATTAAGGTCAGAGAAAAATCCAGGAAATA
AAGGATCAGGACAAAAATGCTATAATTGTGGGAAACCAGGGCATTTGGCAAGGCAATGCAGGCAAGGCAT
AATATGCCATCATTGTGGAAAAAGAGGACATATGCAGAGGGATTGCCGAAAAAAGAAAAGTGATAACATC
AAGCAGCAGGGAAACAGCAGGAGGGGGCCA

>GQ255420.1 Ovine progressive pneumonia virus isolate 99.6 gag protein (gag) gene, partial cds
GAGAAAAAGGGATACCCCGAGCTCAAAGAGGTGATTAAAGCAACATGTAAAATAAAAGTAGGGGCCGGGA
AGGAGACCTTGACAGAAGGGAACTGTCTATGGGCATTAAAGACTATAGACTTTATATTTGAGGATATAAA
AACGGAACCGTGGACTCTTACAAAGATGTATACTGTATGGGGTAGATTAAAGCAGTTAACTCCAGAAGAG
ACAAGTAAAAGAGAGTTTGCCTCCTTGCAAGCTACAATGGCTTGTATGATGTGTAGTCAGATGGGCATGA
AGCCTGAGACAGTGCAGGCAGCACGGGGAATAATAGAGATGAAAGAAGGACTGCACGGAAACAAGGAGGA
CAAAGAGAAGGAGGTAGAGCAACTCTACCCAAATCTAGAAAAACACAAGGAGGTATACCCTATTGTGAAT
TTGCAAGCAGGGGGAAGGAGTTGGAAGGCAGTAGACTCAGTGGTCTTCCAACAGCTGCAAAATGTAGCAA
TGCAGCATGGACTTGTGTCCGAGGATTTTGAAAGGCAAGTGGCATATTATGCCACTACATGGACAAGTAA
GGATATATTAGAGGTATTGGCCATGATGCCTGGGAACAGAGCACAGAAAGAATTAATACAAGGAAAATTA
AATGAAGAAGCGGAAAGGTGGGTGAGACAAAATCCGCCAGGGCCAAATGTCCTCACAGTGGATCAGATCA
TGGGAGTAGGACAGACAAATCATCAGGCATCACAAGCCAACATGGATCAAGCAAGGCAAATATGCTCGCA
ATGGGTAATATCAGCATTACGGTCAGTAAGACATATGTCCCATAGACCAGGGAACCCAATGTTAATAAAG
CAGAAGAATAGTGAAAGCTATGAAGATTTTATAGCAAGACTATTAGAGGCCATAGATGCTGAGCCCGTGA
CAGATCCTATAAAAACATATTTGAAAGTAACTCTGTCATATACAAATGCAAGCACAGACTGTCAAAAACA
AATGGACAGAGTATTAGGAACACGAGTACAGCAAGCATCAGTGGAAGAAAAAATGCAAGCATGTAGAGAT

GTGGGATCAGAAGGGTTCAAGATGCAGCTATTGGCACAAGCATTAAGGCCAGAGAAAAATCCAGGAAATA
GAGGAGCAGGACAAAAATGCTATAATTGTGGAAAACTAGGACATTTGGCAAGGCAGTGTAGGCAAGGCAT
AATATGCCATCATTGTGGAAAAAGAGGACATATGCAGAGGGATTGCAGAAAAAAGAAAAGTGATAACATC
AAGCAGCAGGGAAACAGCAGGAGGGGGCCA

>GQ255383.1 Ovine progressive pneumonia virus isolate 18.2 gag protein (gag) gene, partial cds
GAGAAAAAGGGATACCCCGAGCTCAAGGAGGTGATTAAAGCAACATGTAAAATAAAAGTAGGGGCCGGGA
AGGAGACCTTGACAGAAGGGAACTGTCTATGGGCATTAAAGACTGTAGACTTTATATTTGAGGATATAAA
AACGGAACCGTGGACTCTTACAAAGATGTATACAGTATGGGGGAGATTAAAGCAGCTAACTCCAGAAGAG
ACAAGTAAAAGGGAGTTTGCCTCCTTGCAAGCTACAATGGCTTGTATGATGTGTAGTCAGATGGGCATGA
AGCCTGAGACAGTGCAGGCAGCACGGGGAATAATAGAGATGAAAGAAGGACTGCACGGAAACAAGGAGGA
CAAAGAGAAGGAGGTAGAGCAACTCTACCCAAATCTAGAGAAGCACAAGGAGGTATACCCTATTGTGAAT
TTGCAAGCAGGGGGAAGGAGTTGGAAGGCAGTAGATTCTGTGGTCTTCCAACAGCTGCAAAATGTAGCAA
TGCAGCATGGACTTGTGTCCGAGGATTTTGAAAGGCAAGTGGCATATTATGCCACTACATGGACAAGTAA
GGATATATTAGAGGTATTGGCCATGATGCCTGGGAACAGAGCACAGAAAGAATTAATACAAGGAAAATTA
AATGAAGAAGCGGAAAGGTGGGTGAGACAAAATCCGCCAGGGCCAAATATCCTCACAGTGGATCAGATCA
TGGGAGTAGGACAGACAAATCATCAGGCATCACAAGCCAACATGGATCAAGCAAGGCAAATATGCTTGCA
ATGGGTAATATCAGCATTAAGGTCAGTAAGGCATATGTCACATAGACCAGGGAATCCTATGCTAGTAAAG
CAGAAGAACAGTGAAAGCTATGAAGATTTTATAGCAAGACTATTAGAAGCAATTGATGCAGAACCAGTAA
CAGATCCTATAAAAACATATTTGAAAGTAACTCTGTCATATACGAATGCAAGCACAGATTGTCAAAAACA
AATGGACAGAGTAATAGGAACTCGAGTACAGCAAGCCACAGTAGAAGAAAAAATGCAAGCATGTAGAGAT
GTGGGATCGGAAGGGTTCAAAATGCAGTTATTGGCACAAGCATTAAGGCCAGAGAAAAATCCAGGAAATA
GAGGAGCAGGACAAAAATGCTATAATTGTGGAAAACTAGGACATTTGGCAAGGCAGTGTAGGCAAGGCAT
AATATGCCATCATTGTGGAAAAAGAGGACATATGCAGAGGGATTGCAGAAAAAAGAAAAGTGATAACATC
AAGCTGCAGGGAAACAGCAGGAGGGGGCCA

>S51392.1 gag...rev [maedi-visna-like virus EV1, Genomic RNA Complete, 6 genes, 9203 nt]
AGTAAGGTAAGAGAGACACCTACTGGGAAAGTAGGGA
ATAACCCTTCGACGAAAGAAAAGGCGCTGCTTGGCGACAGGAGGAGGGCTCGCGACCCTGTAATAGGAGA
AGCAGGGGCGAGCTCTGGTCCTGGACCTGAGGAGGGCAAGTGCAGCGCTGGTAAGGAAACCGCCGTGGTG
AGTCTAGATAGAGACATGGCGAAGCAAGGCTCAAGAGAGAAAAAGGGATACCCCGAGCTCAAAGAGGTAA
TAAGGAAAACATGTAGGATAAGAGTAGGCCCAAGGGAAGGAGAACCCTTGACAGAAGGGAACTGTCTATG
GGCATTAAAAACTGTAGACTTTATATTTGAAGATTTAAAAGGAGAGCCGTGGACCATTACAAAAATGTAT
ACAGTATGGGATAGATTAAAACAGTTAACTCCAGAAGAGACAAGTAAAAGAGAATTTGCCTCCTTGCAGG
CCACAATGGCTTGCCTTATGTGTAGTCAGCTGGGTATGAAACCCGAGACAGTGCAAGCAGCAAGGGGAAT
AATGCATATGAAAGAAGGACTACAGGAGAATAAGGAGGAAAAGGAGAAAAAGGTAGAACAACTCTACCCT
AATTTAGAGAAACATAGAGAAGTGTACCCTATTGTAAATCTGCAAGCAGGGGGGAGAAGTTGGAAGGCGG
TAGATTCAGTAGTCTTCCAGCAATTGCAAACTGTGGCTATGCAGCATGGCCTTGTGTCCGAGGATTTTGA
AAGACAGCTGGCATATTATGCTACTACATGGACAAGCAAGGATATATTAGAAGTATTGGCCATGATGCCT
GGGAACAGAGCACAGAAAGAGCTGATTCAGGGAAAATTAAATGAAGAAGCAGAAAGATGGGTAAGGCAGA
ATCCGCCAGGGCCAAATGTCCTCACGGTGGATCAAATCATGGGAGTAGGACAAACAAATCAACAGGCATC
ACAGGCTAATATGGATCAACGAAGGGAACTGTGCTTGCAGTGGGTCATAACAGCCTTGAGAGCGGTAAGG
CATATGTCGCATAGGCCAGGTAACCCAATGCTGGTAAAGCAGAAGAATACTGAGAGTTATGAAGATTTCA
TAGCGAGGTTGCTGGAAGCAATTGATGCAGAACCAGTCACCGACCCTATAAAGACATATTTCAAAAGTGA
CTCTGCATACACGAATGCTAGTACAGATTGTCAAAAGCAAATGGACAGAGTCTTGGGAAATAGGGTCCAA
CAGGCATCAGTAGAAGAGAGATGCAAGCATGTAGAGATGTAGGATCAGAAGGGTTTAAAATGCAACTGTT
AGCACAGGCCTTGAGACCTCCACGAAAAGGAGGCAACGTAGGGTCAAGTCAAAAATGTTATAATTGTGGG
AAAACAAGGACATCTTGCAAGACAATGCAGGCAAGGGATAATTTGCCATCAGTGTGGAAGAGAGGACATG
TGAGAAAAGACTGTCGGCAAAAGAACAAGGATAACATACAGCTGCAGGGAAACAACAGGAGGGGGCCACG
TGTGGTGCCGTCCGCGCCTCCTATGTTG

>GQ255430.1 Ovine progressive pneumonia virus isolate 10.7 gag protein (gag) gene, partial cds
GAGAAAAAGGGATACCCCGAGCTCAAGGAAGTAATTAAGGCAACATGTAAAATAAAAGTAGGAGCCGGGA
AGGAGACCTTGACGGAGGGAAACTGTCTATGGGCATTAAAAACTGTAGACTTTATATTTGAGGATATAAA
AACAGAACCGTGGACTCTTACAAAGATGTATACAGTATGGGGAAGATTAAAGCAGTTAACTCCAGAAGAG
ACAAGTAAAAGAGAGTTTGCCTCCTTACAAGCTACAATGGCTTGCATAATGTGTAGTCAAATGGGCATGA
AGCCTGAGACAGTGCAGGCAGCACAGGGAATAATAGGTATGAAAGAAGGACTACGCGAAAATAAGGAGGA
CAAAGAAAAGGAGGTAGAGCAACTCTACCCAAATCTAGAGAAGCACAGAGAAGTATACCCCATTGTGAAT

TTACAAGCGGGGGGAAGAAGTTGGAAGGCAGTAGATTCTGTGGTCTTCCAACAGCTGCAAAATGTAGCAA
TGCAGCATGGACTTGTGTCCGAGGATTTTGAAAGGCAACTAGCATATTATGCCACCACATGGACAAGTAA
GGATATATTAGAAGTATTGGCCATGATGCCTGGGAACAGGGCACAGAAAGAATTAATACAAGGGAAATTA
AATGAAGAAGCAGAAAGGTGGGTAAGACAGGATCCGCCGGGGCCAAATGTCCTTACAGTGGATCAGATCA
TGGGAGTAGGACAGACAAATCACCAGGCATCACAAGCTAATATGGATCAAGCAAGACAGATATGTTTACA
ATGGGTAATATCAGCATTAAGGTCAGTAAGACATATGTCACATAGACCAGGGAACCCAATGTTAATAAAG
CAGAAGAATAGTGAAAGCTATGAAGATTTTATAGCAAGACTATTAGAGGCCATAGATGCTGAGCCAGTGA
CAGATCCTATAAAAACATATTTAAAAGTAACTCTGTCATATACGAATGCAAGCACAGACTGTCAAAAACA
AATGGACAGAGTATTAGGAACACGAGTACAACAAGCATCAGTGGAAGAAAAAATGCAAGCATGTAGAGAT
GTGGGGTCAGAAGGGTTCAAGATGCAGTTGTTGGCGCAAGCATTAAGGTCAGAGAAAAATCCAGGAAATA
AAGGATCAGGACAAAAATGCTATAATTGTGGGAAACCAGGGCATTTGGCAAGGCAATGCAGGCAAGGCAT
AATATGCCATCATTGTGGAAAAAGAGGACATATGCAGAGGGATTGCCGAAAAAAGAAAAGTGATAACATC
AAGCAGCAGGGAAACAGCAGGAGGGGGCCA

>GQ255421.1 Ovine progressive pneumonia virus isolate 99.8 gag protein (gag) gene, partial cds
GAGAAAAAGGGATACCCCGAGCTCAAGGAGGTGATTAAAGCAACATGTAAAATAAAAGTAGGGGCCGGGA
AGGAGACCTTGACAGAAGGGAACTGTCTATGGGCATTAAAGACTATAGACTTTATATTTGAGGATATAAA
AACGGAACCGTGGACTCTTACAAAGATGTATACTGTATGGGGTAGATTAAAGCAGTTAACTCCAGAAGAG
ACAAGTAAAAGAGAGTTTGCCTCCTTGCAAGCTACAATGGCTTGTATGATGTGTAGTCAGATGGGCATGA
AGCCTGAGACAGTGCAGGCAGCACGGGGAATAATAGAGATGAAAGAAGGACTGCACAGAAACAAGGAGGA
CAAAGAGAAGGAGGTGGAGCAACTCTACCCAAATCTAGAAAAACACAAGGAGGTATACCCTATTGTGAAT
TTGCAAGCAGGGGGGAAGGAGTTGGAAGGCAGTAGACTCAGTGGTCTTCCAACAGCTGCAAAATGTAGCAA
TGCAGCATGGACTTGTGTCCGAGGATTTTGAAAGGCAAGTGGCATATTATGCCACTACATGGACAAGTAA
GGATATATTAGAGGTATTGGCCATGATGCCTGGGAACAGAGCACAGAAAGAATTAATACAAGGAAAATTA
AATGAAGAAGCGGAAAGGTGGGTGAGACAAAATCCGCCAGGGCCAAATGTCCTCACAGTGGATCAGATCA
TGGGAGTAGGACAGACAAATCATCGGGCATCACAAGCCAACATGGATCAAGCAAGGCAAATATGCTTGCA
ATGGGTAATATCAGCATTACGGTCAGTAAGACATATGTCCCATAGACCAGGGAACCCAATGTTAATAAAG
CAGAAGAATAGTGAAAGCTATGAAGATTTTATAGCAAGACTATTAGAGGCCATAGATGCTGAGCCCGTGA
CAGATCCTATAAAAACATATTTGAAAGTAACTCTGTCATATACAAATGCAAGCACAGACTGTCAAAAACA
AATGGACAGAGTATTAGGAACACGAGTACAGCAAGCATCAGTGGAAGAAAAAATGCAAGCATGTAGAGAT
GTGGGATCAGAAGGGTTCAAGATGCAGCTATTGGCACAAGCATTAAGGCCAGAGAAAAATCCAGGAAATA
GAGGAGCAGGACAAAAATGCTATAATTGTGGAAAACTAGGACATTTGGCAAGGCAGTGTAGGCAAGGCAT
AATATGCCATCATTGTGGAAAAAGAGGACATATGCAGAGGGATTGCAGAAAAAAGAAAAGTGATAACATC
AAGCAGCAGGGAAACAGCAGGAGGGGGCCA

>GQ255419.1 Ovine progressive pneumonia virus isolate 99.3 gag protein (gag) gene, partial cds
GAGAAAAAGGGATACCCCGAGCTCAAGGAGGTGATTAAAGCAACATGTAAAATAAAAGTAGGGGCCGGGA
AGGAGACCTTGACAGAAGGGAACTGTCTATGGGCATTAAAGACTATAGACTTTATATTTGAGGATATAAA
AACGGAACCGTGGACTCTTACAAAGATGTATACTGTATGGGGTAGATTAAAGCAGTTAACTCCAGAAGAG
ACAAGTAAAAGAGAGTTTGCCTCCTTGCAAGCTACAATGGCTTGTATGATGTGTAGTCAGATGGGCATGA
AGCCTGAGACAGTGCAGGCAGCACGGGGAATAATAGAGATGAAAGAAGGACTGCACAGAAACAAGGAGGA
CAAAGAGAAGGAGGTGGAGCAACTCTACCCAAATCTAGAAAAACACAAGGAGGTATACCCTATTGTGAAT
TTGCAAGCAGGGGGGAAGGAGTTGGAAGGCAGTAGACTCAGTGGTCTTCCAACAGCTGCAAAATGTAGCAA
TGCAGCATGGACTTGTGTCCGAGGATTTTGAAAGGCAAGTGGCATATTATGCCACTACATGGACAAGTAA
GGATATATTAGAGGTATTGGCCATGATGCCTGGGAACAGAGCACAGAAAGAATTAATACAAGGAAAATTA
AATGAAGGAGCGGAAAGGTGGGTGAGACAAAATCCGCCAGGGCCAAATGTCCTCACAGTGGATCAGATCA
TGGGAGTAGGACAGACAAATCATCAGGCATCACAAGCCAACATGGATCAAGCAAGGCAAATATGCTTGCA
ATGGGTAATATCAGCATTACGGTCAGTAAGACATATGTCCCATAGACCAGGGAACCCAATGTTAATAAAG
CAGAAGAATAGTGAAAGCTATGAAGATTTTATAGCAAGACTATTAGAGGCCATAGATGCTGAGCCCGTGA
CAGATCCTATAAAAACATATTTGAAAGTAACTCTGTCATATACAAATGCAAGCACAGACTGTCAAAAACA
AATGGACAGAGTATTAGGAACACGAGTACAGCAAGCATCAGTGGAAGAAAAAATGCAAGCATGTAGAGAT
GTGGGATCAGAAGGGTTCAAGATGCAGCTATTGGCACAAGCATTAAGGCCAGAGAAAAATCCAGGAAATA
GAGGAGCAGGACAAAAATGCTATAATTGTGGAAAACTAGGACATTTGGCAAGGCAGTGTAGGCAAGGCAT
AATATGCCATCATTGTGGAAAAAGAGGACATATGCAGAGGGATTGCAGAAAAAAGAAAAGTGATAACATC
AAGCAGCAGGGAAACAGCAGGAGGGGGCCA

>GQ255394.1 Ovine progressive pneumonia virus isolate 68.7 gag protein (gag) gene, partial cds
GAGAAAAAGGGATACCCCGAGCTCAAGGAGGTGATTAAAGCAACATGTAAAATAAAAGTAGGGGCCGGGA

AGGAGACCTTGACAGAAGGGAACTGTCTATGGGCATTAAAGACTGTAGACTTTATATTTGAGGATATAAA
AACGGAACCGTGGACTCTTACAAAGATGTATACTGTATGGGGTAGATTAAAGCAGTTAACTCCAGAAGAG
ACAAGTAAAAGAGAGTTTGCCTCCTTGCAAGCTACAATGGCTTGTATGATGTGTAGTCAGATGGGCATGA
AGCCTGAGACAGTGCAGGCAGCACGGGGAATAATAGAGATGAAAGAAGGACTGCACGGAAACAAGGAGGA
CAAAGAGAAGGAGGTGGAGCAACTCTACCCAAATCTAGAAAAACATAAGGAGGTACACCCTATTGTGAAT
TTGCAAGCAGGGGGAAGGAGTTGGAAGGCAGTAGACTCAGTGGTCTTCCAACAGCTGCAAAATGTAGCAA
TGCAGCATGGACTTGTGTCCGAGGATTTTGAAAGGCAAGTGGCATATTATGCCACTACATGGACAAGTAA
GGATATATTAGAGGTATTGGCCATGATGCCTGGGAACAGAGCACAGAAAGATTTAATACAAGGAAAATTA
AATGAAGAAGCGGAAAGGTGGGTGAGACAAAATCCGCCAGGGCCAAATGCCCTCACAGTGGATCAGATCA
TGGGAGTAGGACAGACAAATCATCAGGCATCACAAGCCAACATGGATCAAGCAAGGCAAATATGCTTGCA
ATGGGTAATATCAGCATTAAGGTCAGTAAGACATATGTCCCATAGACCAGGGAACCCAATGTTAGTAAAG
CAGAAGAATAGTGAAAGCTATGAAGATTTTATAGCAAGACTATTAGAGGCCATAGATGCTGAGCCCGTGA
CAGATCCTATAAAAACATATTTGAAAGTAACTTTGTCATATACAAATGCAAGCACAGACTGTCAAAAACA
AATGGACAGAGTATTAGGAACACGAGTACAGCAAGCATCAGTGGAAGAAAAAATGCAAGCATGTAGAGAT
GTGGGATCAGAAGGGTTCAAGATGCAGCTATTGGCACAAGCATTAAGGCCAGAGAAAAATCCAGGGACTA
GAGGAGCAGGACAAAAATGCTATAATTGTGGAAAACTAGGACATTTGGCAAGGCAATGTAGGCAAGGCAT
AATATGCCATCATTGTGGAAAAAGAGGACATATGCAGAGGGATTGCAGAAAAAAGAAAAGTGATAACATC
AAGCAGCAGGGAAACAGCAGGAGGGGGCCA

>JN184353.1 Small ruminant lentivirus isolate 166 gag protein (gag) gene, complete cds
ATGGCGAAGCAAGGCTCAAGGGAGAAAAAGGGATACCCCGAGCTCAAGGAGGTTATAAAAGCAACATGCA
AAATAAGAATTGGGCCAGGGAAGGAGACCTTGACAGAAGGGAATTGTCTGTGGGCCTTGAAAACTATAGA
CTTCATATTTGAAGATTTAAAGGCAGAACCATGGACAATAACAAAAATGTATACAGTATGGGGAAGATTA
AAAAAGTTAACTCCAGAGGAAACAAGCAAAAGGGAATTTGCTTCATTGCAGGCCACCATGGCTTGTTTAA
TGTGTAGCCAAATGGGAATGAAACCAGAGACAGTACAGGCAGCTAGGGGAATAATAAGTATGAAGGAAGG
GCTACAAGAGATAGAAGGAGAAAAGGAGAAAAAGGTAGAACAGCTCTACCCAAACTTAGAAAAGCACAAG
GAAGTGTATCCTATAGTAAATTTACAAGCAGGGGGGAGAAGTTGGAAGGCTGTAGATTCAGTAGTCTTCC
AACAGCTACAAACAGTTGCAATGCAGCATGGACTTGTGTCCGAGGATTTTGAGAGGCAAATGGCATATTA
TGCTACTACTTGGACTAGTAAAGATATATTAGAGGTACTGGCCATGATGCCGGGGAACAGGGCACAAAAA
GAGCTAATCCAAGGGAAATTAAATGAAGAAGCAGAGAGATGGGTAAGACAAAATCCACCTGGACCAAATG
TCCTTACAGTGGATCGGATTATGGGAGTAGGACAAACAAATCAGCAAGCATCCCAGGCCAATATGGATCA
AGCAAGACAAATATGCCTACAATGGGTTATAAATGCCTTGAGGTCTGTAAGGCACATGTCACATAGACCA
GGAAATCCCATGCTGGTGAAGCAAAAGAACAATGAGAGCTATGAGGAGTTTATAGCAAGGTTGTTGGAAG
CAATTGATGCAGAACCAGTAACAGACCCTATAAAAACATATTTGAAGGTAACGCTGTCATATACAAACGC
TAGTACAGATTGTCAAAAACAAATGAATAGGGTATTGGGCACTAGGGTCCAACAAGCAACAGTAGAAGAA
AAAATGCAAGCTTGTCGAGATGTCGGCTCAGAGGGATTTAAAATGCAGTTGTTAGCACAAGCTCTAAGAC
CAGAGAGAGAGACAAGAAATCAGGAGATGGGGCAAAAATGTTATAATTGTGGGAAGCCAGGACATCTTGC
GAGACAGTGTAGACAAGGAGTAATATGTCATCATTGTGGAAAACGGGGACATATACAAAAAGACTGCAGA
CAGAAAAGAAAACAGCAGGGAAACAGTGGGAGGGGGCCACGTGTGGTGCCGTCCGCGCCCCCTATGTTGT
AA

>GQ255418.1 Ovine progressive pneumonia virus isolate 99.2 gag protein (gag) gene, partial cds
GAGAAAAAGGGATACCCCGAGCTCAAGGAGGTGATTAAAGCAACATGTAAAATAAAAGTAGGGGCCGGGA
AGGAGACCTTGACAGAAGGGAACTGTCTATGGGCATTAAAGACTATAGACTTTATATTTGAGGATATAAA
AACGGAACCGTGGACTCTTACAAAGATGTATACTGTATGGGGTAGATTAAAGCAGTTAACTCCAGAAGAG
ACAAGTAAAAGAGAGTTTGCCTCCTTGCAAGCTACAATGGCTTGTATGATGTGTAGTCAGATGGGCATGA
AGCCTGAGACAGTGCAGGCAGCACGGGGAATAATAGAGATGAAAGAAGGACTGCACAGAAACGAGGAGGA
CAAAGAGAAGGAGGTGGAGCAACTCTACCCAAATCTAGAAAAACACAAGGAGGTATACCCTATTGTGAAT
TTGCACGCAGGGGGAAGGAGTTGGAAGGCAGTAGACTCAGTGGTCTTCCAACAGCTGCAAAATGTAGCAA
TGCAGCATGGACTTGTGTCCGAGGATTTTGAAAGGCAAGTGGCATATTATGCCACTACATGGACAAGTAA
GGATATATTAGAGGTATTGGCCATGATGCCTGGGAACAGAGCACAGAAAGAATTAATACAAGGAAAATTA
AATGAAGAAGCGGAAAGGTGGGTGAGACAAAATCCGCCAGGGCCAAATGTCCTCACAGTGGATCAGATCA
TGGGAGTAGGACAGACAAATCATCAGGCATCACAAGCCAACATGGATCAAGCAAGGCAAATATGCTTGCA
ATGGGTAATATCAGCATTACGGTCAGTAAGACATATGTCCCATAGACCAGGGAACCCAATGTTAATAAAG
CAGAAGAATAGTGAAAGCTATGAAGATTTTATAGCAAGACTATTAGAGGCCATAGATGCTGAGCCCGTGA
CAGATCCTATAAAAACATATTTGAAAGTAACTCTGTCATATACAAATGCAAGCACAGACTGTCAAAAACA
AATGGACAGAGTATTAGGAACACGAGTACAGCAAGCATCAGTGGAAGAAAAAATGCAAGCATGTAGAGAT
GTGGGATCAGAAGGGTTCAAGATGCAGCTATTGGCACAAGCATTAAGGCCAGAGAAAAATCCAGGAAATA
GAGGAGCAGGACAAAAATGCTATAATTGTGGAAAACTAGGACATTTGGCAAGGCAGTGTAGGCAAGGCAT

AATATGCCATCATTGTGGAAAAAGAGGACATATGCAGAGGGATTGCAGAAAAAAGAAAAGTGATAACATC
AAGCAGCAGGGAAACAGCAGGAGGGGGCCA

>GQ255417.1 Ovine progressive pneumonia virus isolate 99.1 gag protein (gag) gene, partial cds
GAGAAAAAGGGATACCCCGAGCTCAAGGAGGTGATTAAAGCAACATGTAAAATAAAAGTAGGGGCCGGGA
AGGAGACCTTGACAGAAGGGAACTGTCTATGGGCATTAAAGACTATAGACTTTATATTTGAAGATATAAA
AACGGAACCGTGGACTCTTACAAAGATGTATACTGTATGGGGTAGATTAAAGCAGTTAACTCCAGAAGAG
ACAAGTAAAAGAGAGTTTGCCTCCTTGCAAGCTACAATGGCTTGTATGATGTGTAGTCAGATGGGCATGA
AGCCTGAGACAGTGCAGGCAGCACGGGGAATAATAGAGATGAAAGAAGGACTGCACAGAAACGAGGAGGA
CAAAGAGAAGGAGGTGGAGCAACTCTACCCAAATCTAGAAAAACATAAGGAGGTATACCCTATTGTGAAT
TTGCAAGCAGGGGGAAGGAGTTGGAAGGCAGTAGACTCAGTGGTCTTCCAACAGCTGCAAAATGTAGCAA
TGCAGCATGGACTTGTGTCCGAGGATTTTGAAAGGCAAGTGGCATATTATGCCACTACATGGACAAGTAA
GGATATATTAGAGGTATTGGCCATGATGCCTGGGAACAGAGCACAGAAAGAATTATTACAAGGAAAATTA
AATGAAGAAGCGGAAAGGTGGGTGAGACAAAATCCGCCAGGGCCAAATGTCCTCACAGTGGATCAGATCA
TGGGAGTAGGACAGACAAATCATCAGGCATCACAAGCCAACATGGATCAAGCAAGGCAAATATGCTTGCA
ATGGGTAATATCAGCATTACGGTCAGTAAGACATATGTCCCATAGACCAGGGAACCCAATGTTAATAAAG
CAGAAGAATAGTGAAAGCTATGAAGATTTTATAGCAAGACTATTAGAGGCCATAGATGCTGAGCCCGTGA
CAGATCCTATAAAAACATATTTGAAAGTAACTCTGTCATATACAAATGCAAGCACAGACTGTCAAAAACA
AATGGACAGAGTATTAGGAACACGAGTACAGCAAGCATCAGTGGAAGAAAAAATGCAAGCATGTAGAGAT
GTGGGATCAGAAGGGTTCAAGATGCAGCTATTGGCACAAGCATTAAGGCCAGAGAAAAATCCAGGAAATA
GAGGAGCAGGACAAAAATGCTATAATTGTGGAAAACTAGGACATTTGGCAAGGCAGTGTAGGCAAGGCAT
AATATGCCATCATTGTGGAAAAAGAGGACATATGCAGAGGGATTGCAGAAAAAAGAAAAGTGACAACATC
AAGCAGCAGGGAAACAGCAGGAGGGGGCCA

>GQ255411.1 Ovine progressive pneumonia virus isolate 89.1_(20AUG) gag protein (gag) gene, partial cds
GAGAAAAAGGGATACCCCGAGCTCAAGGAGGTGATTAAAGCAACATGTAAAATAAAAGTAGGGGCCGGGA
AGGAGACCTTGACGGAAGGGAACTGTCTATGGGCATTAAAGACTATAGACTTTATATTTGAGGATATAAA
AACAGAACCGTGGACTCTTACAAAGATGTATACTGTATGGGGAAGATTAAAGCAGTTAACTCCAGAAGAG
ACAAGTAAAAGAGAGTTTGCCTCCTTGCAAGCTACAATGGCTTGTATGATGTGTAGTCAGATGGGCATGA
AGCCTGAGATAGTGCAGGCAGCACGGGGAATAATAGAGATGAAAGAAGGACTGCACGGAAACAAGGAGGA
CAAAGAGAAGGAGGTAGAGCAACTCTACCCAAATCTAGAAAAACATAAGGAGGTATACCCTATTGTGAAT
TTGCAAGCAGGGGGAAGGAGTTGGAAGGCAGTAGATTCTGTGGTCTTCCAACAGCTGCAAAATGTAGCAA
TGCAGCATGGACTTGTGTCCGAGGATTTTGAAAGGCAAGTAGCATATTATGCCACTACATGGACAAGTAA
GGATATATTAGAGGTATTGGCCATGATGCCTGGGAACAGAGCACAGAAAGAATTAATACAGGGGAGATTA
AATGAAGAAGCAGAAAGGTGGGTAAGACAGAATCCACCGGGGCCAAATGTCCTTACAGTGGATCAAATCA
TGGGAGTGGGACAAACAAATCATCAGGCATCACAAGCCAACATGGATCAAGCAAGGCAAATATGCTTGCA
ATGGGTAATATCAGCATTAAGGTCAGTAAGGCATATGTCCCATAAACCAGGGAATCCTATGCTAGTAAAG
CAGAAGAACAGTGAAAGCTATGAAGATTTTATAGCAAGACTATTAGAGGCCATAGATGCTGAGCCAGTGA
CAGATCCTATAAAAACATATTTGAAAGTAACTCTGTCATATACAAATGCAAGCACAGATTGTCAAAAACA
AATGGACAGAGTATTAGGAGCTCGAGTACAGCAAGCCTCAGTAGAAGAAAAAATGCAAGCATGTAGAGAT
GTGGGATCAGAAGGGTTCAAGATGCAGCTATTGGCGCAAGCATTAAGGCCAGAGAAAAATCCAGGAAATA
GAGGAGCAGGACAAAAATGCTATAATTGTGGAAAACTAGGACATTTGGCAAGGCAGTGTAGGCAAGGCAT
AATATGCCATCATTGTGGAAAGAGAGGACATATGCAGAGGGATTGCAGAAAAAAGAAAAGTGATAACATC
AAGCAGCAGGGAAACAGCAGGAGGGGGCCA

>GQ255391.1 Ovine progressive pneumonia virus isolate 68.2 gag protein (gag) gene, partial cds
GAGAAAAAGGGATACCCCGAGCTCAAGGAGGTGATTAAAGCAACATGTAAAATAAAAGTAGGGGCCGGGA
AGGAGACCTTGACAGAAGGGAACTGTCTATGGGCATTAAAGACTGTAGACTTTATATTTGAGGATATAAA
AACGGAACCGTGGACTCTTACAAAGATGTATACAGTATGGGGGAGATTAAAGCAGCTAACTCCAGAAGAG
ACAAGTAAAAGGGAGTTTGCCTCCTTGCAAGCTACAATGGCTTGTATGATGTGTAGTCAGATGGGCATGA
AGCCTGAGACAGTGCAGGCAGCACGGGGAATAATAGAGATGAAAGAAGGACTGCACGGAAACAAGGAGGA
CAAAGAGAAGGAGGTAGAGCAACTCTACCCAAATCTAGAGAAGCACAAGGAGGTATACCCTATTGTGAAT
TTGCAAGCAGGGGGAAGGAGTTGGAAGGCAGTAGATTCTGTGGTCTTCCAACAGCTGCAAAATGTAGCAA
TGCAGCATGGACTTGTGTCCGAGGATTTTGAAAGGCAAGTGGCATATTATGCCACTACATGGACAAGTAA
GGATATATTAGAGGTATTGGCCATGATGCCTGGGAACAGAGCACAGAAAGAATTAATACAAGGAAAATTA
AATGAAGAAGCGGAAAGGTGGGTGAGACAAAATCCGCCAGGGCCAAATGCCCTCACAGTGGATCAGATCA
TGGGAGTAGGACAGACAAATCATCAGGCATCACAAGCCAACATGGATCAAGCAAGGCAAATATGCTTGCA
ATGGGTAATATCAGCATTACGGTCAGTAAGACATATGTCCCATAGACCTGGGAACCCAATGTTAGTAAAG

CAGAAGAATAGTGAAAGCTATGAAGATTTTATAGCAAGACTATTAGAGGCCATAGATGCTGAGCCCGTGA
CAGATCCTATAAAAACATATTTGAAAGTAACTCTGTCATATACAAATGCAAGCACAGACTGTCAAAAACA
AATGGACAGAGTATTAGGAACACGAGTACAGCAAGCATCAGTGGAAGAAAAAATGCAAGCATGTAGAGAT
GTGGGATCAGAAGGGTTCAAGATGCAGCTATTGGCACAAGCATTAAGGCCAGAGAAAAATCCAGGAAATA
GAGGAGCAGGACAAAAATGCTATAATTGTGGAAAACTAGGACATTTGGCAAGGCAGTGCAGGCAAGGCAT
AATATGCCATCATTGTGGAAAAAGAGGACATATGCAGAGGGATTGCAGAAAAAAGAAAAGTGATAACATC
AAGCAGCAGGGAAACAGCAGGAGGGGGCCA

>GQ255387.1 Ovine progressive pneumonia virus isolate 27.3 gag protein (gag) gene, partial cds
GAGAAAAAGGGATACCCCGAGCTCAAGGAGGTGATTAAAGCAACATGTAAAATAAAAGTAGGGGCCGGGA
AGGAGACCTTGACAGAAGGGAACTGTCTATGGGCATTAAAAACTGTAGACTTTATATTTGAGGATATAAA
AACAGAACCGTGGACTCTTACAAAGATGTATACTGTATGGGGTAGATTAAAGCAGTTAACTCCAGAAGAG
ACAAGTAAAAGAGAGTTTGCCTCCTTGCAAGCTACAATGGCTTGTATGATGTGTAGTCAGATGGGCATGA
AGCCTGAGACAGTGCAGGCAGCACGGGGAATAATAGAGATGAAAGAAGGACTGCACGGAAACAAGGAGGA
CAAAGAAAAGGAGATAGAGCAACTCTACCCCAATCTAGAGAAGCACAGAGAAGTGTACCCCATTGTGAAT
TTACAAGCGGGGGGAAGGAGTTGGAAGGCAGTAGATTCTGTGGTCTTCCAACAGCTGCAAAATGTGGCAA
TGCAGCATGGACTTGTGTCCGAGGATTTTGAAAGGCAAATAGCATATTGTGCCACCACATGGACAAGTAA
GGATATCTTAGAAGTATTGGCCATGATGCCTGGGAACAGAGCACAGAAAGAATTAATACAAGGAAAATTA
AATGAAGAAGCGGAAAGGTGGGTGAGACAAAATCCGCCAGGGCCAAATGTCCTCACAGTGGATCAGATCA
TGGGAGTAGGACAGACAAATCACCAGGCATCACAAGCCAACATGGATCAAGCAAGGCAAATATGCTTGCA
ATGGGTAATATCAGCATTAAGATCAGTAAGGCATATGTCACATAGACCAGGGAATCCTATGCTAGTAAAG
CAGAAGAACAGTGAAAGCTATGAAGATTTTATAGCAAGACTATTAGAAGCAATTGATGCAGAGCCAGTAA
CAGATCCTATAAAAACATATTTAAAAGTAACTCTGTCATATACAAATGCAAGCACAGACTGTCAAAAACA
AATGGACAGAGTATTAGGAACACGAGTACAGCAAGCATCAGTGGAAGAAAAAATGCAAGCATGTAGAGAT
GTGGGATCAGAAGGGTTCAAGATGCAGCTATTGGCGCAAGCATTAAGGCCAGAGAAAAATCCAGGAAATA
GAGGAGCAGGACAAAAATGCTATAATTGCGGAAAACTAGGACATTTGGCAAGACAATGTAGGCAAGGCAC
AATATGCCATCATTGTGGAAAAAGAGGACATATGCAGAGGGATTGCAGAAAAAAGAAAAGTGATAACATC
AAGCAGCAGGGAAACAGCAGGAGGGGGCCA

>GQ255381.1 Ovine progressive pneumonia virus isolate 18.1_(20AUG) gag protein (gag) gene, partial cds
GAGAAAAAGGGATACCCCGAGCTCAAGGAAGTAATTAAGGCAACATGTAAAATAAAAGTAGGGGCCGGGA
AGGAGACCTTGACAGAAGGGAACTGTCTATGGGCACTAAAGACTATAGACTTTATATTTGAGGATATAAA
AACGGAACCATGGACTCTTACAAAGATGTATACTGTATGGGGTAGATTAAAGCAGTTAACTCCGGAAGAG
ACAAGTAGAAGAGAGTTTGCCTCCTTGCAAGCTACCATGGCTTGTATGATGTGTAGTCAAATGGGCATGA
AGCCTGAGACAGTGCAGGCAGCACGGGGAATACTAGAGATGAAAGAAGGACTGCACGGAAACAAGGAGGA
TAAAGAGAAGGAGGTAGAGCAACTCTACCCAAATCTAGAAAAACACAAGGAGGTATACCCTATTGTAAAT
TTGCAAGCAGGGGGAAGGAGTTGGAAGGCAGTAGACTCAGTGGTCTTCCAACAGCTGCAAAATGTAGCAA
TGCAGCATGGACTTGTGTCCGAGGATTTTGAAAGGCAAGTAGCATATTATGCCACTACATGGACAAGTAA
GGATATATTAGAGGTATTGGCCATGATGCCTGGGAACAGAGCACAGAAAGAATTAATACAAGGAAAATTA
AATGAAGAAGCGGAAAGGTGGGTGAGACAAAATCCGCCAGGGCCAAATATCCTCACAGTGGATCAGATCA
TGGGAGTAGGACAGACAAATCATCAGGCATCACAAGCCAACATGGATCAAGCAAGGCAAATATGCTTGCA
ATGGGTAATATCAGCATTAAGGTCAGTAAGGCATATGTCACATAGACCAGGGAATCCTATGCTAGTAAAG
CAGAAGAACAGTGAAAGCTATGAAGATTTTATAGCAAGACTATTAGAAGCAATTGATGCAGAACCAGTAA
CAGATCCTATAAAAACATATTTGAAAGTAACTCTGTCATATACGAATGCAAGCACAGATTGTCAAAAACA
AATGGACAGAGTAATAGGAACTCGAGTACAGCAAGCCACAGTAGAAGAAAAAATGCAAGCATGTAGAGAT
GTGGGATCGGAAGGGTTCAAAATGCAGTTATTGGCACAAGCATTAAGGCCAGAGAAAAATCCAGGAAATA
GAGGAGCAGGACAAAAATGCTATAATTGTGGAAAACTAGGACATTTGGCAAGGCAGTGTAGGCAAGGCAT
AATATGCCATCATTGTGGAAAAAGAGGACATATGCAGAGGGATTGCAGAAAAAAGAAAAGTGATAACATC
AAGCTGCAGGGAAACAGCAGGAGGGGGCCA

>GQ255389.1 Ovine progressive pneumonia virus isolate 27.9 gag protein (gag) gene, partial cds
GAGAAAAAGGGATACCCCGAGCTCAAGGAGGTGATTAAAGCAACATGTAAAATAAAAGTAGGGGCCGGGA
AGGAGACCTTGACAGAAGGGAACTGTCTATGGGCATTAAAAACTGTAGACTTTATATTTGAGGATATAAA
AACGGAACCGTGGACTCTTACAAAGATGTATACTGTATGGGGTAGATTAAAGCAGTTAACTCCAGAAGAG
ACAAGTAAAAGAGAGTTTGCCTCCTTGCAAGCTACAATGGCTTGTATGATGTGTAGTCAGATGGGCATGA
AGCCTGAGACAGTGCAGGCAGCACGGGGAATAATAGAGATGAAAGAAGGACTGCACGGAAACAAGGAGGA
CAAAGAAAAGGAGATAGAGCAACTCTACCCCAATCTAGAGAAGCACAGAGAAGTGTACCCCATTGTGAAT
TTACAAGCGGGGGGAAGGAGTTGGAAGGCAGTAGATTCTGTGGTCTTCCAACAGCTGCAAAATGTGGCAA

TGCAGCATGGACTTGTGTCCGAGGATTTTGAAAGGCAAATAGCATATTATGCCACCACATGGACAAGTAA
GGATATCTTAGAAGTATTGGCCATGATGCCTGGGAACAGAGCACAGAAAGAATTAATACAAGGAAAATTA
AATGAAGAAGCGGAAAGGTGGGTGAGACAAAATCCGCCAGGGCCAAATGTCCTCACAGTGGATCAGATCA
TGGGAGTAGGACAGACAAATCACCAGGCATCACAAGCCAACATGGATCAAGCAAGGCAAATATGCTTGCA
ATGGGTAATATCAGCATTAAGATCAGTAAGGCATATGTCACATAGACCAGGGAATCCTATGCTAGTAAAG
CAGAAGAACAGTGAAAGCTATGAAGATTTTATAGCAAGACTATTAGAAGCAATTGATGCAGAACCAGTAA
CAGATCCTATAAAAACATATTTAAAAGTAACTCTGTCATATACAAATGCAAGCACAGACTGTCAAAAACA
AATGGACAGAGTATTAGGAACACGAGTACAGCAAGNATCAGTGGAAGAAAAAATGCAAGCATGTAGAGAT
GTGGGATCAAAAGGGTTCAAGATGCAGCTATTGGCGCAAGCATTAAGGCCAGAGAAAAATCCAGGAAATA
GAGGAGCAGGACAAAAATGCTATAATTGCGGAAAACTAGGACATTTGGCAAGACAATGTAGGCAAGGCAC
AATATGCCATCATTGTGGAAAAAGAGGACATATGCAGAGGGATTGCAGAAAAAAGAAAAGTGATAACATC
AAGCAGCAGGGAAACAGCAGGAGGGGGCCA

>GQ255388.1 Ovine progressive pneumonia virus isolate 27.7 gag protein (gag) gene, partial cds
GAGAAAAAGGGATACCCCGAGCTCAAGGAGGTGATTAGAGCAACATGTAAAATAAAAGTAGGGGCCGGGA
AGGAGACCTTGACAGAAGGGAACTGTCTATGGGCATTAAAAACTGTAGACTTTATATTTGAGGATATAAA
AACGGAACCGTGGACTCTTACAAAGATGTATACTGTATGGGGTAGATTAAAGCAGTTAACTCCAGAAGAG
ACAAGTAAAAGAGAGTTTGCCTCCTTGCAAGCTACAATGGCTTGTATGATGTGTAGTCAGATGGGCATGA
AGCCTGAGACAGTGCAGGCAGCACGGGGAATAATAGAGATGAAAGAAGGACTGCACGGAAACAAGGAGGA
CAAAGAAAAGGAGATAGAGCAACTCTACCCCAATCTAGAGAAGCACAGAGAAGTGTACCCCATTGTGAAT
TTACAAGCGGGGGGAAGGAGTTGGAAGGCAGTAGATTCTGTGGTCTTCCAACAGCTGCAAAATGTGGCAA
TGCAGCATGGACTTGTGTCCGAGGATTTTGAAAGGCAAATAGCATATTATGCCACCACATGGACAAGTAA
GGATATCTTAGAAGTATTGGCCATGATGCCTGGGAACAGAGCACAGAAAGAATTAATACAAGGNAAATTA
AATGAAGAAGCGGAAAGGTGGGTGAGACAAAATCCGCCAGGGCCAAATGTCCTCACAGTGGATCAGATCA
TGGGAGTAGGACAGACAAATCACCAGGCATCACAAGCCAACATGGATCAAGCAAGGCAAATATGCTTGCA
ATGGGTAATATCAGCATTAAGATCAGTAAGGCATATGTCACATAGACCAGGGAATCCTATGCTAGTAAAG
CAGAAGAACAGTGAAAGCTATGAAGATTTTATAGCAAGACTATTAGAAGCAATTGATGCAGAACCAGTAA
CAGATCCTATAAAAACATATTTAAAAGTAACTCTGTCATATACAAATGCAAGCACAGACTGTCAAAAACA
AATGGACAGAGTATTAGGAACACGAGTACAGCAAGCATCAGTGGAAGAAAAAATGCAAGCATGTAGAGAT
GTGGGATCAGAAGGGTTCAAGATGCAGCTATTGGCGCAAGCATTAAGGCCAGAGAAAAATCCAGGAAATA
GAGGAGCAGGACAAAAATGCTATAATTGCGGAAAACTAGGACATTTGGCAAGACAATGTAGGCAAGGCAC
AATATGCCATCATTGTGGGAAAAGAGGACATATGCAGAGGGATTGCAGAAAAAAGAAAAGTGATAACATC
AAGCAGCAGGGAAACAGCAGGAGGGGGCCA

>GQ255405.1 Ovine progressive pneumonia virus isolate 83.3_(20AUG) gag protein (gag) gene, partial cds
GAGAAAAAGGGATACCCCGAGCTCAAGGAGGTGATTAAAGCAACATGTAAAATAAAAGTAGGGGCCGGGA
AGGAGACCTTGACAGAAGGGAACTGTCTATGGGCATTAAAAACTGTAGACTTTATATTTGAGGATATAAA
AGCGGAACCGTGGACTCTTACAAAGATGTATACTGTATGGGGTAGATTAAAGCAGTTAACTCCAGAAGAG
ACAAGTAAAAGAGAGTTTGCCTCCTTGCAAGCTACAATGGCTTGTATGATGTGTAGTCAGATGGGCATGA
AGCCTGAGACAGTGCAGGCAGCACGGGGAATAATAGAGATGAAAGAAGGACTGCACGGAAACAAGGAGGA
CAAAGAAAAGGAGATAGAGCAACTCTACCCCAATCTAGAGAAGCACAGAGAAGTGTACCCCATTGTGAAT
TTACAAGCGGGGGGAAGGAGTTGGAAGGCAGTAGATTCTGTGGTCTTCCAACAGCTGCAAAATGTGGCAA
TGCAGCATGGACTTGTGTCCGAGGATTTTGAAAGGCAAATAGCATATTATGCCACCACATGGACAAGTAA
GGATATCTTAGAAGTATTGGCCATGATGCCTGGGAACAGAGCACAGAAAGAATTAATACAAGGAAAATTA
AATGAAGAAGCGGAAAGGTGGGTGAGACAAAATCCGCCAGGGCCAAATGTCCTCACAGTGGATCAGATCA
TGGGAGTAGGACAGACAAATCACCAGGCATCACAAGCCAACATGGATCAAGCAAGGCAAATATGCTTGCA
ATGGGTAATATCAGCATTAAGGTCAGTAAGGCATATGTCACATAGACCAGGGAATCCTATGCTAGTAAAG
CAGAAGAACAGTGAAAGCTATGAAGATTTTATAGCAAGACTATTAGAAGCAATTGATGCAGAACCAGTAA
CAGATCCTATAAAAACATATTTGAAAGTAACTCTGTCATATACAAATGCAAGCACAGACTGTCAAAAACA
AATGGACAGAGTATTAGGAACACGAGTACAGCAAGCATCAGTGGAAGAAAAAATGCAAGCATGTAGAGAT
GTGGGATCAGAAGGGTTTAAGATGCAGCTATTGGCGCAAGCATTAAGGCCAGAGAAAAATCCAGGAAATA
GAGGAGCAGGACAAAAATGCTATAATTGCGGAAAACTAGGACATTTGGCAAGGCAATGTAGGCAAGGCAT
AATATGCCATCATTGTGGAAAAAGAGGACATATGCAGAGGGATTGCAGAAAAAAGAAAAGTGATAACATC
AAGCAGCAGGGAAACAGCAGGAGGGGGTCA

>GQ255392.1 Ovine progressive pneumonia virus isolate 68.3 gag protein (gag) gene, partial cds
GAGAAAAAGGGATACCCCGAGCTCAAGGAGGTGATTAAAGCAACATGTAAAATAAAAGTAGGGGCCGGGA
AGGAGACCTTGACAGAAGGGAACTGTCTATGGGCATTAAAGACTGTAGACTTTATATTTGAGGATATAAA

AACGGAACCGTGGACTCTTACAAAGATGTATACAGTATGGGGGAGATTAAAGCAGTTAACTCCAGAAGAG
ACAAGTAAAAGAGAGTTTGCCTCCTTGCAAGCTACAATGGCTTGTATGATGTGTAGTCAGATGGGCATGA
AGCCTGAGACAGTGCAGGCAGCACGGGGAATAATAGAGATGAAAGAAGGACTGCACGGAAACAAGGAGGA
CAAAGAGAAGGAGGTGGAGCAACTCTACCCAAATCTAGAAAAACATAAGGAGGTATACCCTATTGTGAAT
TTGCAAGCAGGGGGAAGGAGTTGGAAGGCAGTAGACTCAGTGGTCTTCCAACAGCTGCAAAATGTAGCAA
TGCAGCATGGACTTGTGTCCGAGGATTTTGAAAGGCAAGTGGCATATTATGCCACTACATGGACAAGTAA
GGATATATTAGAGGTATTGGCCATGATGCCTGGGAACAGAGCACAGAAAGAATTAATACAAGGAAAATTA
AATGAAGAAGCGGAAAGGTGGGTGAGACAAAATCCCCCAGGGCCAAATGCCCTCACAGTGGATCAGATCA
TGGGAGTAGGACAGACAAATCATCAGGCATCACAAGCCAACATGGATCAAACAAGGCAAATATGCTTGCA
ATGGGTAATATCAGCATTACGGTCAGTAAGACATATGTCCCATAGACCAGGGAACCCAATGTTAGTAAAG
CAGAAGAATAGTGAAAGCTATGAAGATTTTATAGCAAGACTATTAGAGGCCATAGATGCTGAGCCCGTGA
CAGATCCTATAAAAACATATTTGAAAGTAACTCTGTCATATACAAATGCAAGCACAGACTGTCAAAAACA
AATGGACAGAGTATTAGGAACACGAGTACAGCAAGCATCAGTGGAAGAAAAAATGCAAGCATGTAGAGAT
GTAGGATCAGAAGGGTTCAAGATGCAGTTATTGGCGCAAGCATTAAGGCCAGAGAAAAATCCAGGGACTA
GAGGAGCAGGACAAAAATGCTATAATTGTGGAAAACTAGGACATTTGGCAAGGCAATGTAGGCAAGGCAT
AATATGCCATCATTGTGGAAAAAGAGGACATATGCAGAGGGATTGCAGAAAAAAGAAAAGTGATAACATC
AAGCAGCAGGGAAACAGCAGGAGGGGGCCA

>GQ255386.1 Ovine progressive pneumonia virus isolate 27.1_(June) gag protein (gag) gene, partial cds
GAGAAAAAGGGATACCCCGAGCTCAAGGAGGTGATTAAAGCAACATGTAAAATAAAAGTAGGGGCCGGGA
AGGAGACCTTGACAGAAGGGAACTGTCTATGGGCATTAAAAACTGTAGACTTTATATTTGAGGATATAAA
AACGGAACCGTGGACTCTTACAAAGATGTATACTGTATGGGGTAGATTAAAGCAGTTAACTCCAGAAGAG
ACAAGTAAAAGAGAGTTTGCCTCCTTGCAAGCTACAATGGCTTGTATGATGTGTAGTCAGATGGGCATGA
AGCCTGAGACAGTGCAGGCAGCACGGGGAATAATAGAGATGAAAGAAGGACTGCACGGAAACAAGGAGGA
CAAAGAAAAGGAGATAGAGCAACTCTACCCCAATCTAGAGAAGCACAGAGAAGTGTACCCCATTGTGAAT
TTACAAGCGGGGGGAAGGAGTTGGAAGGCAGTAGATTCTGTGGTCTTCCAACAGCTGCAAAATGTGGCAA
TGCAGCATGGACTTGTGTCCGAGGATTTTGAAAGGCAAATAGCATATTATGCCACCACATGGACAAGTAA
GGATATCTTAGAAGTATTGGCCATGATGCCTGGGAACAGAGCACAGAAAAAATTAATACAGGGAAAATTA
AATGAAGAAGCGGAAAGGTGGGTGAGACAAAATCCGCCAGGGCCAAATGTCCTCACAGTGGATCAGATCA
TGGGAGTAGGACAGACAAATCACCAGGCATCACAAGCCAACATGGATCAAGCAAGGCAAATATGCTTGCA
ATGGGTAATATCAGCATTAAGATCAGTAAGGCATATGTCACATAGACCAGGGAATCCTATGCTAGTAAAG
CAGAAGAACAGTGAAAGCTATGAAGATTTTATAGCAAGACTATTAGAAGCAATTGATGCAGAACCAGTAA
CAGATCCTATAAAAACATATTTAAAAGTAACTCTGTCATATACAAATGCAAGCACAGACTGTCAAAAACA
AATGGACAGAGTATTAGGAACACGAGTACAGCAAGCATCAGTGGAAGAAAAAATGCAAGCATGTAGAGAT
GTGGGATCAGAAGGGTTCAAGATGCAGCTATTGGCGCAAGCATTAAGGCCAGAGAAAAATCCAGGAAATA
GAGGAGCAGGACAAAAATGCTATAATTGCGGAAAACTAGGACATTTGGCAAGACAATGTAGGCAAGGCAC
AATATGCCATCATTGTGGGAAAAGAGGACATATGCAGAGGGATTGCAGAAAAAAGAAAAGTGATAACATC
AAGCAGCAGGGAAACAGCAGGAGGGGGCCA

>GQ255415.1 Ovine progressive pneumonia virus isolate 89.3 gag protein (gag) gene, partial cds
GAGAAAAAGGGATACCCCGAGCTCAAGGAGGTGATTAAAGCAACATGTAAAATAAAAGTAGGGGCCGGGA
AGGAGACCTTGACGGAAGGGAACTGTCTATGGGCATTAAAGACTATAGACTTTATATTTGAGGATATAAA
AACAGAACCGTGGACTCTTACAAAGATGTATACTGTATGGGGAAGATTAAAGCAGTTAACTCCAGAAGAG
ACAAGTAAAAGAGAGTTTGCCTCCCTGCAAGCTACAATGGCTTGCATAATGTGTAGTCAGATGGGCATGA
AGCCTGAGACAGTGCAGGCAGCACGGGGAATAATAGAGATGAAAGAAGGACTGCACGGAAACAAGGAGGA
CAAAGAGAAGGAGGTAGAGCAACTCTACCCAAATCTAGAAAAACATAAGGAGGTATACCCCATTGTGAAT
TTACAAGCAGGGGGAAGAAGTTGGAAGGCAGTAGATTCTGTGGTCTTCCAACAGCTGCAAAATGTAGCAA
TGCAGCATGGACTTGTGTCCGAGGATTTTGAAAGGCAAGTAGCATATTATGCCACTACATGGACAAGTAA
GGATATATTAGAGGTATTGGCCATGATGCCTGGGAACAGAGCACAGAAAGAATTAATACAGGGGAGATTA
AATGAAGAAGCGGAAAGGTGGGTGAGACAGAATCCACCGGGGCCAAATGTCCTCACAGTGGATCAGATCA
TGGGAGTAGGACAGACAAATCATCAGGCATCACAAGCCAACATGGATCAAGCAAGACAGATATGTTTGCA
ATGGGTAATATCAGCATTAAGGTCAGTAAGGCATATGTCCCATAAACCAGGGAATCCTATGCTAGTAAAG
CAGAAGAACAGTGAAAGCTATGAAGATTTTATAGCAAGACTATTAGAGGCCATAGATGCTGAGCCAGTAA
CAGATCCTATAAAAACATATTTGAAAGTAACTCTGTCCTATACAAATGCAAGCACAGACTGTCAAAAACA
AATGGACAGAGTATTAGGAACTCGAGTACAGCAAGCCTCAGTAGAAGAAAAAATGCAAGCATGTAGAGAT
GTGGGATCAGAAGGGTTCAAGATGCAGCTATTGGCGCAAGCATTAAGGCCGGAGAAAAATCCAGGAAATA
GAGGAGCAGGACAAAAATGCTATAATTGTGGAAAACCAGGACATTTAGCAAGGCAATGTAGGCAAGGCAT
AACATGCCATCATTGTGGAAAAAGAGGACATATGCAAAAGGATTGTAGAAAAAAGAAAAGTGATATGAAG
CAGCAGGGAAACAGCAGGAGGGGGCCA

>GQ255414.1 Ovine progressive pneumonia virus isolate 89.2 gag protein (gag) gene, partial cds
GAGAAAAAGGGATACCCCGAGCTCAAGGAGGTGATTAAAGCAACATGTAAAATAAAAGTAGGGGCCGGGA
AGGAGACCTTGACGGAAGGGAACTGTCTATGGGCATTAAAGACTATAGACTTTATATTTGAGGATATAAA
AACAGAACCGTGGACTCTTACAAAGATGTATACAGTATGGGAAAGGTTAAAGCAGTTAACTCCAGAAGAG
ACAAGTAAAAGAGAGTTTGCCTCCTTACAAGCTACAATAGCTTGCATAATGTGTAGTCAAATGGGCATGA
AGCCTGAGACAGTGCAGGCAGCACGGGGAATAATAAGTATGAAAGAAGGACTACGAGAAAATAAGGAGGA
CAAAGAAAAGGAGGTAGAGCAACTCTACCCCAATCTAGAGAAGCACAGAGAAGTGTACCCCATTGTGAAT
TTACAAGCAGGGGGAAGAAGTTGGAAGGCAGTAGATTCTGTGGTCTTCCAACAGCTGCAAAATGTAGCAA
TGCAGCATGGACTTGTGTCCGAGGATTTTGAAAGGCAAATAGCATATTATGCCACTACATGGACAAGTAA
GGATATATTAGAGGTATTGGCCATGATGCCTGGGAACAGAGCACAGAAAGAATTAATACAGGGGAGATTA
AATGAAGAAGCGGAAAGGTGGGTGAGACAAAATCCGCCAGGGCCAAATGTCCTCACAGTGGATCAGATCA
TGGGAGTAAGACAGACAAATCATCAGGCCTCACAAGCCAACATGGATCAAGCAAGGCAAATATGCTTGCA
ATGGGTAATATCAGCATTAAGGTCAGTAAGGCATATGTCCCATAAACCAGGGAATCCTATGCTAGTAAAG
CAGAAGAACAGTGAAAGCTATGAAGATTTTATAGCAAGACTATTAGAGGCCATAGATGCTGAGCCAGTAA
CAGATCCTATAAAAACATATTTGAAAGTAACTCTGTCATATACAAATGCAAGCACAGATTGTCAGAAACA
AATGGACAGAGTATTAGGAACTCGAGTACAGCAAGCCTCAGTAGAAGAAAAAATGCAAGCATGTAGAGAT
GTGGGATCAGAAGGGTTCAAGATGCAGCTATTGGCGCAAGCATTAAGGCCGGAGAAAAATCCAGGAAATA
GAGGAGCAGGACAAAAATGCTATAATTGTGGAAAACCAGGACATTTAGCAAGGCAATGTAGGCAAGGCAT
AACATGCCATCATTGTGGAAAAAGAGGACATATGCAAAAGGATTGTAGAAAAAAGAAAAGTGATATGAAG
CAGCAGGGAAACAGCAGGAGGGGGCCA

>GQ255412.1 Ovine progressive pneumonia virus isolate 89.1 gag protein (gag) gene, partial cds
GAGAAAAAGGGATACCCCGAGCTCAAGGAGGTGATTAAAGCAACATGTAAAATAAAAGTAGGGGCCGGGA
AGGAGACCTTGACGGAAGGGAACTGTCTATGGGCATTAAAGACTATAGATTTTATATTTGAGGATATAAA
AGCAGAACCGTGGACTCTTACAAAGATGTATACTGTATGGGGAAGATTAAAGCAGTTAACTCCAGAAGAG
ACAAGTAAAAGAGAGTTTGCCTCCTTGCAAGCTACAATGGCTTGTATGATGTGTAGTCAAATGGGCATGA
AGCCTGAGACAGTGCAGGCAGCACGGGGAATAATAAGTATGAAAGAAGGACTACGAGAAAATAAGGAGGA
CAAAGAAAAGGAGGTAGAGCAACTCTACCCCAATCTAGAGAAGCACAGAGAAGTGTACCCCATTGTGAAT
TTACAAGCAGGGGGAAGAAGTTGGAAGGCAGTAGATTCTGTGGTCTTCCAACAGCTGCAAAATGTGGCGA
TGCAGCATGGACTGGTGTCCGAGGATTTTGAAAGGCAAATAGCATATTATGCCACCACATGGACAAGTAA
GGATATATTAGAAGTATTGGCCATGATGCCTGGGACCAGAGCACAGAAAGAATTAATACAGGGGAGATTA
AATGAAGAAGCGGAAAGGTGGGTGAGACAAAATCCGCCAGGGCCAAATGTCCTCACAGTGGATCAGATCA
TGGGAGTAGGACAGACAAATCATCAGGCATCACAAGCCAACATGGATCAAGCAAGGCAAATATGCTTGCA
ATGGGTAATATCAGCATTAAGGTCAGTAAGGCATATGTCACATAAACCAGGAAACCCAATGTTAGTAAAG
CAGAAGAATAGCGAAAGCTATGAAGATTTTATAGCAAGATTATTAGAGGCCATAGATGCTGAGCCAGTGA
CAGATCCTATAAAAACATATTTGAAAGTAACTCTGTCATATACAAATGCAAGCACAGATTGTCAGAAACA
AATGGACAGAGTATTAGGACAACGAGTACAGCAAGCATCAGTAGAAGAAAAAATGCAAGCATGTAGAGAT
GTAGGATCAGAAGGGTTCAAGATGCAGCTATTGGCGCAAGCATTAAGGCCGGAGAAAAATCCAGGAAATA
GAGGAGCAGGACAAAAATGCTATAATTGTGGAAAACCAGGACATTTAGCAAGGCAATGTAGGCAAGGCAT
AACATGCCATCATTGTGGAAAAAGAGGACATATGCAAAAGGATTGTAGAAAAAAGAAAAGTAATATGAAG
CAGCAGGGAAACAGCAGGAGGGGGCCA

>MW248464.1 Visna-maedi virus strain NM1111, complete genome
ATGGCGAAGCAAGGCTCGAGAGAGAAAAAGGGATACCCCGAGCTCAAAGAGGTCATTAA
GATGACGTGTAAAATAAAAGTAGGGGCCGGGAAGGAGACCTTGACAGAAGGGAATTGTTTATGGGCATTA
AAGACCATAGACTTTATCTTTGAAGATATAACAACAGAGCCGTGGACTCTTACAAAAATGTATACAGTAT
GGGAAAGGTTAAAAAAGTTGACTCCTGAAGACACAAGTAAAAGAGAGTTTGCTTCCTTGCAAGCTACATT
GGCTTGCATAATGTGTAGTCAAATGGGTATGAAGCCCGAGACAGTGCAGGCAGCAAAGGGAATTATAAGT
ATGAAAGAAGGACTACAAGGAAGCAAGGAGAAACAGGAGTCGCAGGTAGAGCAGCTCTATCCGAATTTAG
AAAAACATAAGGAAGTGTATCCTATTGTAAATCTGCAAGCAGGAGGAAGAAGTTGGAAGGCAGTAGAAGC
AGTGGTCTTCCAGCAACTACAAACAGTAGCAATGCAGCATGGACTTGTGTCCGAGGACTTTGAGAGACAG
TTAGCATATAATGCGACTACCTGGACTAGTAAAGATATCTTAGAAGTATTGGCCATGATGCCCGGGAATA
GAGCACAGAAAGAGTTAATACAGGGAAAATTAAATGAGGAAGCAGAGAGATGGGTAAGGCAAAATCCTCC
GGGTCCAAATGTCCTTACAGTGGATCAAATTATGGGAGTAGGACAAACAAATCAACAGGCATCACAAGCT
AATATGGATCAAGCAAGACAGATATGCTTACAATGGGTAATATCTGCATTAAGATCAGTGAGACATATGT
CTCATAGGCCTGGAAATCCTATGCTAGTAAAGCAAAAGAATACAGAGAGCTATGAAGAGTTCATAGCTAG
ATTGTTAGAGGCAATTGATGCAGAGCCAGTCACAGATCCCATAAAGACTTATCTAAAGGTAACTCTGTCA
TACACAAATGCAAGCACAGATTGTCAGAAACAGATGGACAGAGTGTTAGGGACAAGAGTTCAGCAGGCAA
CAGTAGAAGAAAAAATGCAAGCATGTAGAGATGTGGGCTCAGAAGGATTTAAAATGCAGTTGTTAGCACA

GGCCTTACGGCCTGACAGAAGCTTTAATAATCAAAGACAAGGCCAAAAATGTTATAATTGTGGAAAACCG
GGACATTTGGCTAGACAATGCCGACAAGGCATAATATGCCATCATTGTGGAAAAAGGGGGCACATGCAGA
AAGACTGTCGGCAAAAGAAACAAAAAGATATAAAGCAGCAGGGAAACAGCAGGAGGGGGCCACGTGTGGT
GCCGTCCGCACCCCCTATGTTATAA

>GQ255410.1 Ovine progressive pneumonia virus isolate 83.7 gag protein (gag) gene, partial cds
GAGAAAAAGGGATACCCCGAGCTCAAGGAGGTGATTAAAGCAACATGTAAAATAACAGTAGGGGCCGGGA
AGGAGACCTTGACAGAAGGGAACTGTCTATGGGCATTAAAAACTGTAGACTTTATATTTGAGGATATAAA
AACGGAACCGTGGACTCTTACAAAGATGTATACTGTATGGGGTAGATTAAAGCAGTTAACTCCAGAAGAG
ACAAGTAAAAGAGAGTTTGCCTCCTTGCAAGCTACAATGGCTTGTATGATGTGTAGTCAGATGGGCATGA
AGCCTGAGACAGTGCAGGCAGCACGGGGAATAATAGAGATGAAAGAAGGACTGCACGGAAACAAGGAGGA
CAAAGAAAAGGAGATAGAGCAACTCTACCCCAATCTAGAGAAGCACAGAGAAGTGTACCCCATTGTGAAT
TTACAAGCGGGGGGAAGGAGTTGGAAGGCAGTAGATTCTGTGGTCTTCCAACAGCTGCAAAATGTGGCAA
TGCAGCATGGACTTGTGTCCGAGGATTTTGAAAGGCAAATAGCATATTATGCCACCACATGGACAAGTAA
GGATATCTTAGAAGTATTGGCCATGATGCCTGGGAACAGAGCACAGAAAGAATTAATACAAGGAAAATTA
ATTGAAGAAGCGGAAAGGTGGGTGAGACAAAATCCGCCAGGGCCAAATGTCCTCACAGTGGATCAGATCA
TGGGAGTAGGACAGACAAATCACCAGGCATCACAAGCCAACATGGATCAAGCAAGGCAAATATGCTTGCA
ATGGGTAATATCAGCATTAAGGTCAGTAAGGCATATGTCACATAGACCAGGGAATCCTATGCTAGTAAAG
CAGAAGAACAGTGAAAGCTATGAAGATTTTATAGCAAGACTATTAGAAGCAATTGATGCAGAACCAGTAA
CAGATCCTATAAAAACATATTTGAAAGTAACTCTGTCATATACAAATGCAAGCACAGACTGTCAAAAACA
AATGGACAGAGTATTAGGAACACGAGTACAGCAAGCATCAGTGGAAGAAAAAATGCAAGCATGTAGAGAT
GTGGGATCAGAAGGGTTCAAGATGCAGCTATTGGCGCAAGCATTAAGGCCAGAGAAAAATCCAGGAAATA
GAGGAGCAGGACAAAAATGCTATAATTGCGGAAAACTAGGACATTTGGCAAGGCAATGTAGGCAAGGCAT
AATATGCCATCATTGTGGAAAAAGAGGACATATGCAGAGGGATTGCAGAAAAAAGAAAGGTGATAACATC
AAGCAGCAGGGAAACAGCAGGAGGGGGCCA

>GQ255409.1 Ovine progressive pneumonia virus isolate 83.6 gag protein (gag) gene, partial cds
GAGAAAAAGGGATACCCCGAGCTCAAGGAGGTGATTAAAGCAACATGTAAAATAAAAGTAGGGGCCGGGA
AGGAGACCTTGACAGAAGGGAACTGTCTATGGGCATTAAAAACTGTAGACTTTATATTTGAGGATATAAA
AACGGACCCGTGGACTCTTACAAAGATGTATACTGTATGGGGTAGATTAAAGCAGTTAACTCCAGAAGAG
ACAAGTAAAAGAGAGTTTGCCTCCTTGCAAGCTACAATGGCTTGTATGATGTGTAGTCAGATGGGCATGA
AGCCTGAGACAGTGCAGGCAGCACGGGGAATAATAGAGATGAAAGAAGGACTGCACGGAAACAAGGAGGA
CAAAGAAAAGGAGATAGAGCAACTCTACCCCAATCTAGAGAAGCACAGAGAAGTGTACCCCATTGTGAAT
TTACAAGCGGGGGGAAGGAGTTGGAAGGCAGTAGATTCTGTGGTCTTCCAACAGCTGCAAAATGTGGCAA
TGCAGCATGGACTTGTGTCCGAGGATTTTGAAAGGCAAATAGCATATTATGCCACCACATGGACAAGTAA
GGATATCTTAGAAGTATTGGCCGTGATGCCTGGGAACAGAGCACAGAAAGAATTAATACAAGGAAAATTA
AATGAAGAAGCGGAAAGGTGGGTGAGACAAAATCCGCCAGGGCCAAATGTCCTCACAGTGGATCAGATCA
TGGGAGTAGGACAGACAAATCACCAGGCATCACAAGCCAACATGGATCAAGCAAGGCAAATATGCTTGCA
ATGGGTAATATCAGCATTAAGGTCAGTAAGGCATATGTCACATAGACCAGGGAATCCTATGCTAGTAAAG
CAGAAGAACAGTGAAAGCTATGAAGATTTTATAGCAAGACTATTAGAAGCAATTGATGCAGAACCAGTAA
CAGATCCTATAAAAACATATTTGAAAGTAACTCTGTCATATACAAATGCAAGCACAGACTGTCAAAAACA
AATGGACAGAGTATTAGGAACACGAGTACAGCAAGCATCAGTGGAAGAAAAAATGCAAGCATGTAGAGAT
GTGGGATCAGAAGGGTTCAAGATGCAGCTATTGGCGCAAGCATTAAGGCCAGAGAAAAATCCAGGAAATA
GAGGAGCAGGACAAAAATGCTATAATTGCGGAAAACTAGGACATTTGGCAAGGCAATGTAGGCAAGGCAT
AATATGCCATCATTGTGGAAAAAGAGGACATATGCAGAGGGATTGCAGAAAAAAGAAAAGTGATAACATC
AAGCAGCAGGGAAACAGCAGGAGGGGGCCA

>GQ255406.1 Ovine progressive pneumonia virus isolate 83.3 gag protein (gag) gene, partial cds
GAGAAAAAGGGATACCCCGAGCTCAAGGAGGTGATTAAAGCAACATGTAAAATAAAAGTAGGGGCCGGGA
AGGAGACCTTGACAGAAGGGAACTGTCTATGGGCATTAAAAACTGTAGACTTTATATTTGAGGATATAAA
AACGGAACCGTGGACTCTTACAAAGATGTATACTGTATGGGGTAGATTAAAGCAGTTAACTCCAGAAGAG
ACAAGTAAAAGAGAGTTTGCCTCCTTGCAAGCTACAATGGCTTGTATGATGTGTAGTCAGATGGGCATGA
AGCCTGAGACAGTGCAGGCAGCACGGGGAATAATAGAGATGAAAGAAGGACTGCACGGAAACAAGGAGGA
CAAAGAAAAGGAGATAGAGCAACTCTACCCCAATCTAGAGAAGCACAGAGAAGTGTACCCCATTGTGAAT
TTACAAGCGGGGGGAAGGAGTTGGAAGGCAGTAGATTCTGTGGTCTTCCAACAGCTGCAAAATGTGGCAA
TGCAGCATGGACTTGTGTCCGAGGATTTTGAAAGGCAAATAGCATATTATGCCACCACATGGACAAGTAA
GGATATCTTAGAAGTATTGGCCATGATGCCTGGGAACAGAGCACAGAAAGAATTAATACAAGGAAAATTA
AATGAAGAAGCGGAAAGGTGGGTGAGACAAAATCCGCCAGGGCCAAATGTCCTCACAGTGGATCAGATCA

TGGGAGTAGGACAGACAAATCACCAGGCATCACAAGCCAACATGGATCAAGCAAGGCAAATATGCTTGCA
ATGGGTAATATCAGCATTAAGGTCAGTAAGGCATATGTCACATAGACCAGGGAATCCTATGCTAGTAAAG
CAGAAGAACAGTGAAAGCTATGAAGATTTTATAGCAAGACTATTAGAAGCAATTGATGCAGAACCAGTAA
CAGATCCTATAAAAACATATTTGAAAGTAACTCTGTCATATACAAATGCAAGCACAGACTGTCAAAAACA
AATGGACAGAGTATTAGGAACACGAGTACAGCAAGCATCAGTGGAAGAAAAAATGCAAGCATGTAGAGAT
GTGGGATCAGAAGGGTTCAAGATGCAGCTATTGGCGCAAGCATTAAGGCCAGAGAAAAATCCAGGAAATA
GAGGAGCAGGACAAAAATGCTATAATTGCGGAAAACTAGGACATTTGGCAAGGCAATATAGGCAAGGCAT
AATATGCCATCATTGTGGAAAAAAAGGACATATGCAGAGGGATTGCAGAAAAAAGAAAAGTGATAACATC
AAGCAGCAGGGAAACAGCAGGAGGGGGCCA

>GQ255435.1 Ovine progressive pneumonia virus isolate WLC-1.4 gag protein (gag) gene, partial cds
GAGAAAAAGGGATACCCCGAGCTCAAGGAAGTAATTAAAGCAACATGCAGAATAAAGGTAGGGGCCGGGA
AGGAGACCTTGACAGAAGGGAACTGTTTATGGGCATTAAAAACTGTAGACTTTATATTTGAGGATATAAA
GACAGAACCGTGGACCCTTACAAAGATGTATACTGTATGGGACAGATTAAGGCAGTTGACTCCAGAAGAG
ACAAGTAAAAGAGAGTTTGCCTCCTTGCAAGCTACATTGGCTTGCCTAATGTGTAGTCAAATGGGCATGA
AGCCTGAGACAGTGCAGGCAGCACGGGGAATAATAAGTATGAAGGGAGGACTACAAGAGAATAAGGAGGA
AAAGAAGGTAGAACAACTCTACCCAAATTTAGAGAAGCATAAGGAAGTATACCCCATTGTGAATTTGCAA
GCGGGGGGAAGGAGTTGGAAGGCAGTAGACTCCGTGGTCTTCCAACAGCTGCAAAATGTAGCAATGCAGC
ATGGACTTGTGTCCGAGGATTTTGAAAGGCAAATGGCATATTATGCCACCACATGGACAAGTAAGGATAT
CCTAGAAGTATTGGCCATGATGCCTGGAAATAGAGCTCAGAAAGAGTTAATACAAGGGAAATTAAATGAG
GAAGCGGAGAGATGGGTAAGGCAGAATCCCCCGGGGCCAAATGTCCTTACAGTGGATCAAATTATGGGAG
TAGGACAAACAAATCAACAGGCATCACAAGCTAACATGGATCAAGCAAGACAGATATGCTTGCAATGGGT
AATAACAGCATTAAGATCTGTAAGACATATGTCTCATAGGCCAGGAAATCCTATGCTGGTGAAGCAGAAA
AATACAGAGAGTTATGAAGACTTCATAGCAAGACTGTTAGAAGCAATCGATGCAGAACCAGTTATGGACC
CCATCAAAACGTACTTGAAAGTAACTCTGTCATATACAAATGCTAGCACAGATTGTCAAAAGCAAATGGA
CAGAGTGTTAGGAACCAGAGTCCAACATGCAACAGTAGAAGAAAAGATGCAAGCCTGTAGAGATGTAGGA
TCAGAAGGATTTAAGATGCAGTTATTGGCCCAAGCGTTAAGGCCAGAAAAAAACCCGGGAAATAGGGGAC
CAGGGCAAAAATGTTATAATTGTGGAAAACCAGGACATTTAGCAAGGCAGTGCCGCCAAGGCATAATATG
CCATCATTGTGGAAAAAGAGGACATATGCAAAAAGATTGTAGGAAAAAGAAAAGTGAGAACATGAAGCAG
CAGGGAAACAGCAGGAGGGGGCCA

>GQ255434.1 Ovine progressive pneumonia virus isolate WLC-1.3 gag protein (gag) gene, partial cds
GAGAAAAAGGGATACCCCGAGCTCAAGGAAGTAATTAAAGCAACATGCAGAATAAAGGTAGGGGCCGGGA
AGGAGACCTTGACAGAAGGGAACTGTTTATGGGCATTAAAAACTGTAGACTTTATATTTGAGGATATAAA
GACAGAACCGTGGACTCTTACAAAGATGTATACTGTATGGGACAGATTAAGGCAGTTGACTCCAGAAGAG
ACAAGTAAAAGAGAGTTTGCCTCCTTGCAAGCTACATTGGCTTGCCTAATGTGTAGTCAAATGGGCATGA
AGCCTGAGACAGTGCAGGCAGCACGGGGAATAATAAGTATGAAGGGAGGACTACAAGAGAATAAGGAGGA
AAAGAAGGTAGAACAACTCTACCCAAATTTAGAGAAGCATAAGGAAGTATACCCCATTGTGAATTTGCAA
GCGGGGGGAAGGAGTTGGAAGGCAGTAGACTCCGTGGTCTTCCAACAGCTGCAAAATGTAGCAATGCAGC
ATGGACTTGTGTCCGAGGATTTTGAAAGGCAAATGGCATATTATGCCACCACATGGACAAGTAAGGATAT
CTTAGAAGTATTGGCCATGATGCCTGGAAATAGAGCTCAGAAAGAGTTAATACAAGGGAAATTAAATGAG
GAAGCGGTGAGATGGGTAAGGCAGAATCCCCCGGGGCCAAATGTCCTTACAGTGGATCAAATTATGGGAG
TAGGACAAACAAATCAACAGGCATCACAAGCTAACATGGATCAAGCAAGACAGATATGCTTGCAATGGGT
AATAACAGCATTAAGATCTGTAAGACATATGTCTCATAGGCCAGGAAATCCTATGCTGGTGAAGCAGAAA
AATACAGAGAGTTATGAAGACTTCATAGCAAGACTGTTGGAAGCAATCGATGCAGAACCAGTTATGGACC
CCATCAAAACGTACTTGAAAGTAACTCTGTCATATACAAATGCTAGCACAGATTGTCAAAAGCAAATGGA
CAGAGTGTTAGGAACCAGAGTCCAACATGCAACAGTAGAAGAAAAGATGCAAGCCTGTAGAGATGTAGGA
TCAGAAGGATTTAAGATGCAGTTATTGGCCCAAGCGTTGAGGCCAGAAAAAAACCCGGGAAATAGGGGAC
CAGGGCAAAAATGTTATAATTGTGGAAAACCAGGACATTTAGCAAGGCAGTGCCGCCAAGGCATAATATG
CCATCATTGTGGAAAAAGAGGACATATGCAAAAAGATTGTAGGAAAAAGAAAAGTGAGAACATGAAGCAG
CAGGGAAACAGCAGGAGGGGGCCA

>GQ255433.1 Ovine progressive pneumonia virus isolate WLC-1.1 gag protein (gag) gene, partial cds
GAGAAAAAGGGATACCCCGAGCTCAAGGAAGTAATTAAAGCAACATGCAGAATAAAGGTAGGGGCCGGGA
AGGAGACCTTGACAGAAGGGAACTGTTTATGGGCATTAAAAACTGTAGACTTTATATTTGAGGATATAAA
GACAGAACCGTGGACTCTTACAAAGATGTATACTGTATGGGACAGATTAAGGCAGTTGACTCCAGAAGAG
ACAAGTAAAAGAGAGTTTGCCTCCTTGCAAGCTACATTGGCTTGCCTAATGTGTAGTCAAATGGGCATGA
AGCCTGAGACAGTGCAGGCAGCACGGGGAATAATAAGTATGAAGGGAGGACTACAAGAGAATAAGGAGGA

AAAGAAGGTAGAACAACTCTACCCGAATTTAGAGAAGCATAAGGAAGTATACCCCATTGTGAATTTGCAA
GCGGGGGGAAGGAGTTGGAAGGCAGTAGACTCCGTGGTCTTCCAACAGCTGCAAAATGTAGCAATGCAGC
ATGGACTTGTGTCCGAGGATTTTGAAAGGCAAATGGCATATTATGCCACCACATGGACAAGTAAGGATAT
CTTAGAAGTATTGGCCATGATGCCTGGAAATAGAGCTCAGAAAGAGTTAATACAAGGGAAATTAAATGAG
GAAGCGGAGAGATGGGTAAGGCAGAATCCCCCGGGGCCAAATGTCCTTACAGTGGATCAAATTATGGGAG
TAGGACAAACAAATCAACAGGCATCACAAGCTAACATGGATCAGGCAAGACAGATATGCTTGCAATGGGT
AATAACAGCATTAAGATCTGTAAGACATATGTCTCATAGGCCAGGAAATCCTATGCTGGTGAAGCAGAAA
AATACAGAGAGTTATGAAGACTTCATAGCAAGACTGTTGGAAGCAATCGATGCAGAACCAGTTATGGACC
CCATCAAAACGTACTTGAAAATAACTCTGTCATATACAAATGCTAGCACAGATTGTCAAAAGCAAATGGA
CAGAGTGTTAGGAACCAGAGTCCAACATGCAACAGTAGAAGAAAAGATGCAAGCCTGTAGAGATGTAGGA
TCAGAAGGATTTAAGATGCAGTTATTGGCCCAAGCGTTAAGGCCAGAAAAAAACCCGGGAAATAGGGGAC
CAGGGCAAAAATGTTATAATTGTGGAAAACCAGGACATTTAGCAAGGCAGTGCCGCCAAGGCATAATATG
CCATCATTGTGGAAAAAGAGGACATATGCAAAAAGATTGTAGGAAAAAGAAAAGTGAGAACATGAAGCAG
CAGGGAAACAGCAGGAGGGGGCCA

>GQ255413.1 Ovine progressive pneumonia virus isolate 89.2_(20AUG) gag protein (gag) gene, partial cds
GAGAAAAAGGGATACCCCGAGCTCAAGGAGGTGATTAAGGCAACATGTAAAATAAAAGTAGGGGCCGGGA
AGGAGACCTTGACGGAAGGGAACTGTCTATGGGCATTAAAGACTATAGACTTTATATTTGAGGATATAAA
AACAGAACCGTGGACTCTTACAAAGATGTATACTGTATGGGGAAGATTAAAGCAGTTAACTCCAGAAGAG
ACAAGTAAAAGAGAGTTTGCCTCCTTGCAAGCTACAATGGCTTGTATGATGTGTAGTCAGATGGGCATGA
AGCCTGAGACAGTGCAGGCAGCACGGGGAATAATAGAGATGAAAGAAGGACTGCACGGAAACAAGGAGGA
CAAAGAGAAGGAGGTAGAGCAACTCTACCCAAATCTAGAAAAACATAAGGAGGTATACCCTATTGTGAAT
TTGCAAGCAGGGGGGAAGGAGTTGGAAGGCAGTAGATTCTGTGGTCTTCCAACAGCTGCAAAATGTAGCAA
TGCAGCATGGACTTGTGTCCGAGGATTTTGAAAGGCAAATAGCATATTATGCCACTACATGGACAAGTAA
GGATATATTAGAGGTATTGGCCATGATGCCTGGGAACAGAGCACAGAAAGAATTAATACAGGGGAGATTA
AATGAAGAAGCGGAAAGGTGGGTGAGACAGAATCCACCGGGGCCAAATGTCCTCACAGTGGATCAGATCA
TGGGAGTAGGACAGACAAATCATCAGGCATCACAAGCCAACATGGATCAAGCAAGGCAAATATGCTTGCA
ATGGGTAATATCAGCATTAAGGTCAGTAAGGCATATGTCCCATAAACCAGGGAATCCTATGCTAGTAAAG
CAGAAGAACAGTGAAAGCTATGAAGATTTTATAGCAAGACTATTAGAGGCCATAGATGCTGAGCCAGTGA
CAGATCCTATAAAAACATATTTGAAAGTAACTCTGTCATATACAAATGCAAGCACAGATTGTCAAAAACA
AATGGACAGAGTATTAGGAACTCGAGTACAGCAAGCCTCAGTAGAAGAAAAAATGCAAGCATGTAGAGAT
GTGGGATCAGAAGGGTTCAAGATGCAGCTATTGGCGCAAGCATTAAGGCCAGAGAAAAATCCAGGAAATA
GAGGAGCAGGACAAAAATGCTATAATTGTGGAAAACTAGGACATTTGGCAAGGCAGTGTAGGCAAGGCAT
AATATGCCATCATTGTGGAAAGAGAGGACATATGCAGAGGGATTGCAGAAAAAAGAAAAGTGATAACATC
AAGCAGCAGGGAAACAGCAGGAGGGGGCCA

>GQ255408.1 Ovine progressive pneumonia virus isolate 83.5 gag protein (gag) gene, partial cds
GAGAAAAAGGGATACCCCGAGCTCAAGGAGGTGATTAAAGCAACATGTAAAATAAAAGTAGGGGCCGGGA
AGGAGACCTTGACAGAAGGGAACTGTCTATGGGCATTAAAAACTGTAGACTTTATATTTGAGGATATAAA
AGCGGAACCGTGGACTCTTACAAAGATGTATACTGTATGGGGTAGATTAAAGCAGTTAACTCCAGAAGAG
ACGAGTAAAAGAGAGTTTGCCTCCTTGCAAGCTACAATGGCTTGTATGATGTGTAGTCAGATGGGCATGA
AGCCTGAGACAGTGCAGGCAGCACGGGGAATAATAGAGATGAAAGAAGGACTGCACGGAAACAAGGAGGA
CAAAGAAAAGGAGATAGAGCAACTCTACCCCAATCTAGAGAAGCACAGAGAAGTGTACCCCATTGTGAAT
TTACAAGCGGGGGGAAGGAGTTGGAAGGCAGTAGATTCTGTGGTCTTCCAACAGCTGCAAAATGTGGCAA
TGCAGCATGGACTTGTGTCCGAGGATTTTGAAAGGCAAATAGCATATTATGCCACCACATGGACAAGTAA
GGATATCTTAGAAGTATTGGCCATGATGCCTGGGAACAGAGCACAGAAAGAATTAATACAAGGAAAATTA
AATGAAGAAGCGGAAAGGTGGGTGAGACAAAATCCGCCAGGGCCAAATGTCCTCACAGTGGATCAGATCA
TGGGAGTAGGACAGACAAATCACCAGGCATCACAAGCCAACATGGATCAAGCAAGGCAAATATGCTTGCA
ATGGGTAATATCAGCATTAAGGTCAGTAAGGCATATGTCACATAGACCAGGGAATCCTATGCTAGTAAAG
CAGAAGAACAGTGAAAGCTATGAGGATTTTATAGCAAGACTATTAGAAGCAATTGATGCAGAACCAGTAA
CAGATCCTATAAAAACATATTTGAAAGTAACTCTGTCATATACAAATGCAAGCACAGACTGTCAAAAACA
AATGGACAGAGTATTAGGAACACGAGTACAGCAAGCATCAGTGGAAGAAAAAATGCAAGCATGTAGAGAT
GTGGGATCAGAAGGGTTCAAGATGCAGCTATTAGCGCAAGCATTAAGGCCAGAGAAAAATCCAGGAAATA
GAGGAGCAGGACAAAAATGCTATAATTGCGGAAAACTAGGACATTTGGCAAGGCAATGTAGGCAAGGCAT
AATATGCCATCATTGTGGAAAAAGAGGACATATGCAGAGGGATTGCAGAAAAAAGAAAAGTGATAACATC
AAGCAGCAGGGAAACAGCAGGAGGGGGTCA

>GQ255407.1 Ovine progressive pneumonia virus isolate 83.4 gag protein (gag) gene, partial cds

GAGAAAAAGGGATACCCCGAGCTCAAGGAGGTGATTAAAGCAACATGTAAAATAAAAGTAGGGGCCGGGA
AGGAGACCTTGACAGAAGGGAACTGTCTATGGGCATTAAAAACTGTAGACTTTATATTTGAGGATATAAA
AACGGACCCGTGGACTCTTACAAAGATGTATACTGTATGGGGTAGATTAAAGCAGTTAACTCCAGAAGAG
ACAAGTAAAAGAGAGTTTGCCTCCTTGCAAGCTACAATGGCTTGTATGATGTGTAGTCGGATGGGCATGA
AGCCTGAGACAGTGCAGGCAGCACGGGGAATAATAGAGATGAAAGAAGGACTGCACGGAAACAAGGAGGA
CAAAGAAAAGGAGATAGAGCAACTCTACCCCAATCTAGAGAAGCACAGAGAAGTGTACCCCATTGTGAAT
TTACAAGCGGGGGGAAGGAGTTGGAAGGCAGTAGATTCTGTGGTCTTCCAACAGCTGCAAAATGTGGCAA
TGCAGCATGGACTTGTGTCCGAGGATTTTGAAAGGCAAATAGCATATTATGCCACCACATGGACAAGTAA
GGATATCTTAGAAGTATTGGCCATGATGCCTGGGAACAGAGCACAGAAAGAATTAATACAAGGAAAATTA
AATGAAGAAGCGGAAAGGTGGGTGAGACAAATTCCGCCAGGGCCAAATGTCCTCACAGTGGATCAGATCA
TGGGAGTAGGACAGACAAATCACCAGGCATCACAAGCCAACATGGATCAAGCAAGGCAAATATGCTTGCA
ATGGGTAATATCAGCATTAAGGTCAGTAAGGCATATGTCACATAGACCAGGGAATCCTATGCTAGTAAAG
CAGAAGAACAGTGAAAGCTATGAAGATTTTATAGCAAGACTATTAGAAGCAATTGATGCAGAACCAGTAA
CAGATCCTATAAAAACATATTTGAAAGTAACTCTGTCATATACAAATGCAAGCACAGACTGTCAAAAACA
AATGGACAGAGTATTAGGAACACGAGTACAGCAAGCATCAGTGGAAGAAAAAATGCAAGCATGTAGAGAT
GTGGGATCAGAAGGGTTCAAGATGCAGCTATTGGCGCAAGCATTAAGGCCAGAGAAAAATCCAGGAAATA
GAGGAGCAGGACAAAAATGCTATAATTGCGGAAAACTAGGACATTTGGCAAGGCAATGTAGGCAAGGCAT
AATATGCCATCATTGTGGAAAAAGAGGACATATGCAGAGGGATTGCAGAAAAAAGAAAAGTGATAACATC
AAGCAGCAGGGAAACAGCAGGAGGGGGCCA

>GQ255385.1 Ovine progressive pneumonia virus isolate 18.4 gag protein (gag) gene, partial cds
GAGAAAAAGGGATACCCCGAGCTCAAGGAGGTGATTAAAGCAACATGTAAAATAAAAGTAGGGGCCGGGA
AGGAGACCTTGACAGAAGGGAACTGTCTATGGGCACTAAAGACTATAGACTTTATATTTGAGGATATAAA
AACGGAACCATGGACTCTTACAAAGATGTATACTGTATGGGGTAGATTAAAGCAGTTAACTCCGGAAGAG
ACAAGTAGAAGAGAGTTTGCCTCCTTGCAAGCTACCATGGCTTGTATGATGTGTAGTCAAATGGGCATGA
AGCCTGAGACAGTGCAGGCAGCACGGGGAATACTAGAGATGAAAGAAGGACTGCACGGAAACAAGGAGGA
TAAAGAGAAGGAGGTAGAGCAACTCTACCCAAATCTAGAAAAACACAAGGAGGTATACCCTATTGTAAAT
TTGCAAGCAGGGGGAAGGAGTTGGAAGGCAGTAGACTCAGTGGTCTTCCAACAGCTGCAAAATGTAGCAA
TGCAGCATGGACTTGTGTCCGAGGATTTTGAAAGGCAAGTAGCATATTATGCCACTACATGGACAAGTAA
GGATATATTAGAGGTATTGGCCATGATGCCTGGGAACAGAGCACAGAAAGAATTAATACAAGGAAAATTA
AATGAAGAAGCGGAAAGGTGGGTGAGACAAAATCCGCCAGGGCCAAATATCCTCACAGTGGATCAGATCA
TGGGAGTAGGACAGACAAATCATCAGGCATCACAAGCCAACATGGATCAAGCAAGGCAAATATGCTTGCA
ATGGGTAATATCAGCATTAAGGTCAGTAAGGCATATGTCACATAGACCAGGGAATCCTATGCTAGTAAAG
CAGAAGAACAGTGAAAGCTATGAAGATTTTATAGCAAGACTATTAGAAGCAATTGATGCAGAACCAGTAA
CAGATCCTATAAAAACATATTTGAAAGTAACTCTGTCATATACGAATGCAAGCACAGATTGTCAAAAACA
AATGGACAGAGTAATAGGAACTCGAGTACAGCAAGCCACAGTAGAAGAAAAAATGCAGGCATGTAGAGAT
GCGGGATCGGAAGGGTTCAAAATGCAGTTATTGGCACAAGCATTAAGGCCAGAGAAAAATCCAGGAAATA
GAGGAGCAGGACAAAAATGCTATAATTGTGGAAAACTAGGACATTTGGCAAGGCAGTGTAGGCAAGGCAT
AATATGCCATCATTGTGGAAAAAGAGGACATATGCAGAGGGATTGCAGAAAAAAGAAAAGTGATAACATC
AAGCTGCAGGGAAACAGCAGGAGGGGGCCA

>GQ255384.1 Ovine progressive pneumonia virus isolate 18.3 gag protein (gag) gene, partial cds
GAGAAAAAGGGATACCCCGAGCTCAAGGAGGTGATTAAAGCAACATGTAAAATAAAAGTAGGGGCCGGGA
AGGAGACCTTGACAGAAGGGAACCGTCTATGGGCACTAAAGACTATAGACTTTATATTTGAGGATATAAA
AACGGAACCATGGACTCTTACAAAGATGTATACTGTATGGGGTAGATTGAAGCAGTTAACTCCGGAAGAG
ACAAGTAGAAGAGAGTTTGCCTCCTTGCAAGCTACCATGGCTTGTATGATGTGTAGTCAAATGGGCATGA
AGCCTGAGACAGTGCAGGCAGCACGGGGAATACTAGAGATGAAAGAAGGACTGCACGGAAACAAGGAGGA
TAAAGAGAAGGAGGTAGAGCAACTCTACCCAAATCTAGAAAAACACAAGGAGGTATACCCTATTGTAAAT
TTGCAAGCAGGGGGAAGGAGTTGGAAGGCAGTAGACTCAGTGGTCTTCCAACAGCTGCAAAATGTAGCAA
TGCAGCATGGACTTGTGTCCGAGGATTTTGAAAGGCAAGTAGCATATTATGCCACTACATGGACAAGTAA
GGATATATTAGAGGTATTGGCCATGATGCCTGGGAACAGAGCACAGAAAGAATTAATACAAGGAAAATTA
AATGAAGAAGCGGAAAGGTGGGTGAGACAAAATCCGCCAGGGCCAAATATCCTCACAGTGGATCAGATCA
TGGGAGTAGGACAGACAAATCATCAGGCATCACAAGCCAACATGGATCAAGCAAGGCAAATATGCTTGCA
ATGGGTAATATCAGCATTAAGGTCAGTAAGGCATATGTCACATAGACCAGGGAATCCTATGCTAGTAAAG
CAGAAGAACAGTGAAAGCTATGAAGATTTTATAGCAAGACTATTAGAAGCAATTGATGCAGAACCAGTAA
CAGATCCTATAAAAACATATTTGAAAGTAACTCTGTCATATACGAATGCAAGCACAGATTGTCAAAAACA
AATGGACAGAGTAATAGGAACTCGAGTACAGCAAGCCACAGTAGAAGAAAAAATGCAAGCATGTAGAGAT
GTGGGATCGGAAGGGTTCAAAATGCAGTTATTGGCACAAGCATTAAGGCCAGAGAAAAATCCAGGAAATA
GAGGAGCAGGACAAAAATGCTATAATTGTGGAAAACTAGGACATTTGGCAAGGCAGTGTAGGCAAGGCAT

AATATGCCATCATTGTGGAAAAAGAGGACATATGCAGAGGGATTGCAGAAAAAAGAAAAGTGATAACATC
AAGCTGCAGGGAAACAGCAGGAGGGGGCCA

>GQ255436.1 Ovine progressive pneumonia virus isolate WLC-1.8 gag protein (gag) gene, partial cds

GAGAAAAAGGGATACCCCGAGCTCAAGGAAGTAATTAAAGCAACATGCAGAATAAAGGTAGGGGCCGGGA
AGGAGACCTTGACAGAAGGGAACTGTTTATGGGCATTAAAAACTGTAGACTTTATATTTGAGGATATAAA
GACAGAACCGTGGACTCTTACAAAGATGTATACTGTATGGGACAGATTAAGGCAGTTGACTCCAGAAGAG
ACAAGTAAAAGAGAGTTTGCCTCCTTGCAAGCTACATTGGCTTGCCTAATGTGTAGTCAAATGGGCATGA
AGCCTGAGACAGTGCAGGCAGCACGGGGAATAATAAGTATGAAGGGAGGACTACAAGAGAATAAGGAGGA
AAAGAAGGTAGAACAACTCTACCCAAATTTAGAGAAGCATAAGGAAGTATACCCCATTGTGAATTTGCAA
GCGGGGGGAAGGAGTTGGAAGGCAGTAGACTCCGTGGTCTTCCAACAGCTGCAAAATGTAGCAATGCAGC
ATGGACTTGTGTCCGAGGATTTTGAAAGGCAAATGGCATATTATGCCACCACATGGACAAGTAAGGATAT
CTTAGAAGTATTGGCCATGATGCCTGGAAATAGAGCTCAGAAAGAGTTAATACAAGGGAAATTAAATGAG
GAAGCGGAGAGATGGGTAAGGCAGAATCCCCCGGGGCCAAATGTCCTTACAGTGGATCAAATTATGGGAG
TAGGACAAACAAATCAACAGGCATCACAAGCTAACATGGATCAAGCAAGACAGATATGCTTGCAATGGGT
AATAACAGCATTAAGATCTGTAAGACATATGTCTCATAGGCCAGGAAATCCTATGCTGGTGAAGCAGAAA
AATACAGAGAGTTATGAAGACTTCATAGCAAGACTGTTGGAAGCAATCGATGCAGAACCAGTTATGGACC
CCATCAAAACGTACTTGAAAGTAACTCTGTCATATACAAATGCTAGCACAGATTGTCAAAAGCAAATGGA
CAGAGTGTTAGGAACCAGAGTCCAACATGCAACAGTAGAAGAAAAGATGCAAGCCTGTAGAGATGTAGGA
TCAAAAGGATTTAAGATGCAGTTATTGGCCCAAGCGTTAAGGCCAGAAAAAAACCCGGGAAATAGGGGAC
CAGGGCAAAAATGTTATAATTGTGGAAAACCAGGACATTTAGCAAGGCAGTGCCGCCAAGGCATAATATG
CCATCATTGTGGAAAAAGAGGACATATGCAAAAAGATTGTAGGAAAAAGAAAAGTGAGAACATGAAGCAG
CAGGGAAACAGCAGGAGGGGGCCA

>GQ255404.1 Ovine progressive pneumonia virus isolate 83.2 gag protein (gag) gene, partial cds

GAGAAAAAGGGATGCCCCGAGCTCAAGGAGGTGATTAAAGCAACATGTAAAATAAAAGTAGGGGCCGGGA
AGGAGACCTTGACAGAAGGGAACTGTCTATGGGCATTAAAAACTGTAGACTTTATATTTGAGGATATAAA
AACGGACCCGTGGACTCTTACAAAGATGTATACTGTATGGGGTAGATTAAAGCAGTTAACTCCAGAAGAG
ACAAGTAAAAGAGAGTTTGCCTCCTTGCAAGCTACAATGGCTTGTATGATGTGTAGTCAGATGGGCATGA
AGCCTGAGACAGTGCAGGCAGCACGGGGAATAATAGAGATGAAAGAAGGACTGCACGGAAACAAGGAGGA
CAAAGAAAAGGAGATAGAGCAACTCTACCCCAATCTAGAGAAGCGCAGAGAAGTGTACCCCATTGTGAAT
TTACAAGCGGGGGGAAGGAGTTGGAAGGCAGTAGATTCTGTGGTCTTCCAACAGCTGCAAAATGTGGCAA
TGCAGCATGGACTTGTGTCCGAGGATTTTGAAAGGCAAATAGCATATTATGCCACCACATGGACAAGTAA
GGATATCTTAGAAGTATTGGCCATGATGCCTGGGAACAGAGCACAGAAAGAATTAATACAAGGAAAATTA
AATGAAGAAGCGGAAGGGTGGGTGAGACAAAATCCGCCAGGGCCAAATGTCCTCACAGTGGATCAGATCA
TGGGAGTAGGACAGACAAATCACCAGGCATCACAAGCCAACATGGATCAAGCAAGGCAAATATGCTTGCA
ATGGGTAATATCAGCATTAAGGTCAGTAAGGCATATGTCACATAGACCAGGGAATCCTATGCTAGTAAAG
CAGAAGAACAGTGAAAGCTATGAAGATTTTATAGCAAGACTATTAGAAGCAATTGATGCAGAACCAGTAA
CAGATCCTATAAAAACATATTTGAAAGTAACTCTGTCATATACAAATGCAAGCACAGACTGTCAAAAACA
AATGGACAGAGTATTAGGAACACGAGTACAGCAAGCATCAGTGGAAGAAAAAATGCAAGCATGTAGAGAT
GTGGGATCAGAAGGGTTCAAGATGCAGCTATTGGCGCAAGCATTAAGGCCAGAGAAAAATCCAGGAAATA
GAGGAGCAGGACAAAAATGCTATAATTGCGGAAAACTAGGACATTTGGCAAGGCAATGTAGGCAAGGCAT
AATATGCCATCATTGTGGAAAAAGAGGACATATGCAGAGGGATTGCAGAAAAAAGAAAAGTGATAACATC
AAGCAGCAGGGAAACAGCAGGAGGGGGCCA

>GQ255403.1 Ovine progressive pneumonia virus isolate 83.1 gag protein (gag) gene, partial cds

GAGAAAAAGGGATACCCCGAGCTCAAGGAGGTGATTAAAGCAACATGTAAAATAAAAGTAGGGGCCGGGA
AGGAGACCTTGACAGAAGGGAACTGTCTATGGGCATTAAAAACTGTAGACTTTATATTTGAGGATATAAA
AAAGGAACCGTGGACTCTTACAAAGATGTATACTGTATGGGGTAGATTAAAGCAGTTAACTCCAGAAGAG
ACAAGTAAAAGAGAGTTTGCCTCCTTGCAAGCTACAATGGCTTGTATGATGTGTAGTCAGATGGGCATGA
AGCCTGAGACAGTGCAGGCAGCACGGGGAATAATAGAGATGAAAGAAGGACTGCACGGAAACAAGGAGGA
CAAAGAAAAGGGGATAGAGCAACTCTACCCCAATCTAGAGAAGCACAGAGAAGTGTACCCCATTGTGAAT
TTACAAGCGGGGGGAAGGAGTTGGAAGGCAGTAGATTCTGTGGTCTTCCAACAGCTGCAAAATGTGGCAA
TGCAGCATGGACTTGTGTCCGAGGATTTTGAAAGGCAAATAGCATATTATGCCACCACATGGACAAGTAA
GGATATCTTAGAAGTATTGGCCATGATGCCTGGGACCAGAGCACAGAAAGAATTAATACAAGGAAAATTA
AATGAAGAAGCGGAAAGGTGGGTGAGACAAAATCCGCCAGGGCCAAATGTCCTCACAGTGGATCAGATCA
TGGGAGTAGGACAGACAAATCACCAGGCATCACAAGCCAACATGGATCAAGCAAGGCAAATATGCTTGCA
ATGGGTAATATCAGCATTAAGGTCAGTAAGGCATATGTCACATAGACCAGGGAATCCTATGCTAGTAAAG

CAGAAGAACAGTGAAAGCTATGAAGATTTTATAGCAAGACTATTAGAAGCAATTGATGCAGAACCAGTAA
CAGATCCTATAAAAACATATTTGAAAGTAACTCTGTCATATACAAATGCAAGCACAGACTGTCAAAAACA
AATGGACAGAGTATTAGGAACACGAGTACAGCAAGCATCAGTGGAAGAAAAAATGCAAGCATGTAGAGAT
GTGGGATCAGAAGGGTTCAAGATGCAGCTATTGGCGCAAGCATTAAGGCCAGAGAAAAATCCAGGAAATA
GAGGAGCAGGACAAAAATGCTATAATTGCGGAAAACTAGGACATTTGGCAAGGCAATGTAGGCAAGGCAT
AATATGCCATCATTGTGGAAAAAGAGGACATATGCAGAGGGATTGCAGAAAAAAGAGAAGTGATAACATC
AAGCAGCGGGGAAACAGCAGGAGGGGGCCA

>GQ255393.1 Ovine progressive pneumonia virus isolate 68.4 gag protein (gag) gene, partial cds
GAGAAAAAGGGATACCCCGAGCTCAAGGACGTAATTAAGGCAACATGTAAAATAAAAGTAGGGGCCGGGA
AGGAGACCTTGACAGAAGGGAACTGTCTATGGGCATTAAAGACTGTAGACTTTATATTTGAGGATATAAA
AACGGAACCGTGGACTCTTGCAAAGATGTATACAGTATGGGGGAGATTAAAGCAGCTAACTCCAGAAGAG
ACAAGTAAAAGAGAGTTTGCCTCCTTGCAAGCTACTATGGCTTGCCTCATGTGTAGTCAAATGGGCATGA
AGCCTGAGACAGTGCAGGCAGCACGGGGAATAATAGAGATGAAAGAAGGACTGCGCGGAAACAAGGAGGA
CAAAGAGAAGGAGGTAGAGCAACTCTACCCAAATCTAGAGAAGCACAAGGAGGTATACCCTATTGTGAAT
TTGCAAGCAGGGGGGAAGGAGTTGGAAGGCAGTAGATTCTGTGGTCTTCCAACAGCTGCAAAATGTAGCGA
TGCAGCATGGACTTGTGTCCGAGGATTTTGAAAGGCAAATAGCGTATTATGCCACCACATGGACAAGTAA
GGATATATTAGAAGTATTGGCCATGATGCCTGGGAACAGAGCACAGAAAGAATTATACAGGGGAGAATTA
AATGAAGAAGCGGAAAGGTGGGTAAGACAAAATCCACAGGGGCCAAATGTCCTCACAGTGGATCAAATTA
TGGGAGTAGGACAGACAAATCATCAGGCATCACAAGCCAACATGGATCAAGCAAGACAGATATGTTTGCA
ATGGGTAATATCAGCATTAAGGTCAGTAAGGCATATGTCACATAGACCAGGAAACCCAATGTTAATAAAA
CAAAAGAATAGTGAAAGCTATGAAGATTTTATAGCAAGACTATTAGAGGCCATAGACGCTGAGCCAGTGA
CAGATCCTATAAAAACGTATTTGAAAGTAACTCTGTCATATACGAATGCAAGCACAGATTGTCAAAAACA
AATGGACAGAGTATTGGGGACACGAGTACAGCAAGCATCAGTGGAAGAAAAAATGCAAGCATGTAGAGAT
GTAGGATCAGAAGGGTTCAAGATGCAGTTATTGGCGCAAGCATTAAGGCCAGAGAAAAATCCAGGGACTA
GAGGAGCAGGACAAAAATGCTATAATTGTGGAAAACTAGGACATTTGGCAAGGCAATGTAGGCAAGGCAT
AATATGCCATCATTGTGGAAAAAGAGGACATATGCAAAAGGATTGTAGGAAAAAGAAAAGTGATATGAAG
CAGCAGGGAAACAGCAGGAGGGGGCCA

***POL* gene**

>NC_001452.1 Visna/Maedi virus strain kv1772, complete genome

**ATGCCATCATTGTGGAAAAAGAGGACATATGCAAAAGGACTGCCGGCAGAAGAAACAGCAGGGAAACAAC
AGGAGGGGGCCACGTGTGGTGCCGTCCGCGCCCCCTATGTTGTAACAGAAGCACCACCCAAAATAGAAAT
AAAAGTAGGAACAAGGTGGAAAAAATTATTAGTAGATACAGGGGCAGATAAAACTATAGTAACATCCCAT
GATATGTCAGGGATACCAAAGGGAAGGATAATATTACAGGGCATAGGGGGAATAATAGAAGGAGAAAAAT
GGGAACAAGTACACTTGCAATATAAAGATAAAATAATCAGAGGTACCATAGTGGTGTTAGCTACGAGTCC
GGTAGAAGTATTAGGAAGAGATAATATGAGAGAATTGGGAATAGGATTAATTATGGCAAATTTAGAAGAA
AAGAAAATTCCCAGTACAAGAGTAAGATTAAAAGAGGGATGTAAGGGACCCCACATAGCGCAATGGCCTT
TGACGCAAGAAAAAATTAGAGGGATTAAAAGAAATAGTAGACAGATTAGAGAAGGAAGGGAAAGTAGGAAG
AGCGCCCCCACACTGGACTTGTAATACCCCTATATTTTGTATTAAGAAGAAATCAGGAAAATGGAGGATG
TTAATAGATTTTAGAGAATTAAATAAGCAAACAGAAGATTTAGCAGAAGCACAGTTAGGGTTACCGCATC
CAGGAGGATTACAGAGAAAGAAACATGTAACAATATTAGATATAGGAGATGCATATTTTACAATACCATT
ATATGAGCCATATAGACAATATACATGCTTTACCATGTTAAGTCCAAATAATTTAGGACCATGTGTAAGA
TATTATTGGAAAGTGTTACCACAAGGATGGAAATTAAGTCCTGCAGTGTATCAATTTACAATGCAAAAAA
TATTAAGAGGATGGATAGAAGAACACCCTATGATACAATTTGGAATATACATGGATGATATCTATATAGG
GAGTGATTTAGGACTAGAAGAGCACAGGGGTATCGTGAACGAACTAGCATCATATATAGCGCAATATGGA
TTCATGCTGCCTGAAGATAAGAGGCAAGAAGGATACCCGGCTAAATGGCTTGGATTTGAATTGCATCCGG
AGAAATGGAAATTTCAAAAACATACGCTCCCAGAGATTACAGAAGGACCCATAACCTTGAATAAACTACA
GAAATTAGTAGGAGATTTAGTTTGGAGACAATCCCTAATAGGAAAAAGCATCCCAAATATCTTAAAATTA
ATGGAAGGAGATAGGGCTTTACAAAGTGAAAGATACATAGAGAGTATACATGTAAGAGAATGGGAAGCCT
GTAGACAAAAGCTGAAGGAAATGGAAGGAAATTATTATGATGAAGAGAAGGATATCTATGGGCAACTAGA
TTGGGGAAATAAAGCAATAGAATACATAGTATTTCAAGAAAAAGGAAAACCTTTATGGGTAAATGTAGTA
CATAGTATTAAGAATTTGAGTCAAGCCCAACAAATTATCAAAGCAGCACAAAAACTGACACAAGAAGTAA
TAATAAGAACAGGCAAGATACCCTGGATTTTGTTGCCGGGAAGGGAAGAAGATTGGATATTAGAGTTACA
AATGGGAAACATAAATTGGATGCCATCATTTTGGTCATGTTATAAAGGCTCGGTGAGGTGGAAAAAGAGG
AATGTAATAGCGGAAGTAGTCCCAGGACCAACATATTATACCGATGGAGGAAAGAAAAATGGGCGGGGAA
GCCTGGGGTATATTACCTCCACAGGTGAAAAGTTTAGAATACATGAAGAAGGGACAAATCAGCAGTTAGA
ATTGAGGGCCATAGAAGAGGCATGTAAACAGGGACCAGAAAAAATGAATATAGTAACAGATAGCAGGTAT**

GCATATGAATTTATGTTGCGGAACTGGACGAAGAAGTAATAAGAAACCCTATACAAGCGAGAATCATGG
AATTAGTGCATAATAAAGAAAAAATAGGGGTACATTGGGTGCCTGGACACAAGGGGATTCCTCAAAATGA
AGAAATAGATAGGTATATCTCAGAAATATTTTTAGCAAAAGAGGGAAGAGGGATTTTACAAAAAAGGGCA
GAAGATGCTGGATATGACTTAATATGTCCACAGGAGATAAGCATTCCGGCGGGGCAAGTGAAAAGGATAG
CAATTGACTTGAAAATAAATTTGAAAAAGGACCAGTGGGCCATGATAGGGACCAAAAGCAGTTTTGCAAA
TAAGGGAGTATTCGTACAAGGAGGTATCATAGATTCGGGATATCAAGGAACAATACAAGTAGTGATATAT
AATAGTAATAATAAAGAAGTAGTAATACCACAGGGAAGAAAATTTGCACAGTTGATCCTCATGCCTCTAA
TACATGAAGAGTTGGAGCCATGGGGAGAAACAAGAAAAACAGAAAGAGGGGAACAAGGATTTGGATCAAC
AGGGATGTATTGGATAGAAAATATTCCCCTAGCAGAAGAAGAGCATAACAAATGGCATCAAGATGCTGTG
TCCTTGCATTTAGAATTTGGGATTCCCAGGACAGCTGCAGAAGATATAGTTCAACAATGTGATGTGTGTC
AAGAAAATAAAATGCCTAGTACATTAAGAGGCAGTAATAAAAGGGGCATAGACCATTGGCAAGTAGACTA
TACCCACTATGAAGACAAGATAATATTGGTCTGGGTAGAAACAAATTCAGGACTAATCTATGCAGAGAGG
GTAAAAGGAGAAACAGGACAAGAATTTAGGGTGCAAACTATGAAATGGTATGCGATGTTTGCCCCGAAAT
CATTGCAGTCTGATAACGGACCAGCATTTGTAGCAGAATCTACTCAGCTCTTAATGAAATATTTGGGCAT
AGAACATACTACAGGGATCCCCTGGAACCCACAATCTCAAGCATTAGTAGAGAGAACACATCAGACGTTA
AAGAATACATTAGAAAAACTTATACCTATGTTTAACGCGTTTGAATCAGCCCTCGCAGGGACCCTCATTA
CTCTAAATATAAAAAGAAAGGGTGGGCTAGGGACAAGCCCTATGGATATATTTATATTTAATAAGGAACA
ACAAAGAATACAGCAACAAAGTAAATCAAAACAAGAAAAAATTCGATTTTGTTATTACAGAACAAGAAAA
AGAGGGCATCC

>M10608.1 Visna/Maedi virus, complete genome
ATGCCATCATTGTGGAAAAAGAGGACATATGCAAAAGGACTGCCGGCAGAAGAAACAGCAGGGAAACAAC
AGGAGGGGGCCACGTGTGGTGCCGTCCGCGCCCCCTATGTTGTAACAGAAGCACCACCCAAAATAGAAAT
AAAAGTAGGAACAAGGTGGAAAAAATTATTAGTAGATACGGGGGCAGATAAAACTATAGTAACATCCCAT
GATATGTCAGGGATACCAAAGGGAAGGATAATATTACAGGGCATAGGGGGAATAATAGAAGGAGAAAAAT
GGGAACAAGTACACTTGCAATATAAAGATAAAATGATCAAAGGTACCATAGTGGTGTTAGCTACGAGTCC
GGTAGAAGTATTAGGAAGAGATAATATGAGAGAATTGGGAATAGGATTAATTATGGCAAATTTAGAAGAA
AAGAAAATTCCCAGTACAAGAGTAAGATTAAAAGAGGGATGTAAGGGACCCCACATAGCGCAATGGCCTT
TGACGCAAGAAAAATTAGAGGGATTAAAAGAAATAGTAGACAGATTAGAGAAGGAAGGGAAAGTAGGAAG
AGCGCCCCCACACTGGACTTGTAATACCCCTATATTTGTATTAAGAAGAAATCAGGAAAATGGAGGATG
TTAATAGATTTTAGAGAATTAAATAAGCAAACAGAAGATTTAGCAGAAGCACAGTTAGGGTTACCTCATC
CAGGAGGATTACAGAGAAAGAAACATGTAACAATATTAGATATAGGAGATGCATATTTTACAATACCATT
ATATGAGCCATATAGACAATATACATGCTTTACCATGTTAAGTCCAAATAATTTAGGACCATGTGTAAGA
TATTATTGGAAAGTGTTACCACAAGGATGGAAATTAAGTCCTGCAGTGTATCAATTTACAATGCAAAAAA
TATTAAGAGGATGGATAGAAGAACACCCTATGATACAATTTGGAATATACATGGATGATATCTATATAGG
GAGTGATTTAGGACTAGAAGAGCACAGGGGTATCGTGAACGAACTAGCATCATATATAGCGCAATATGGA
TTCATGCTGCCTGAAGATAAGAGGCAAGAAGGATACCCGGCGAAATGGCTTGGATTTGAATTGCATCCGG
AGAAATGGAAATTTCAAAAACATACGCTCCCAGAGATTACAGAAGGACCCATAACCTTGAATAAACTACA
GAAATTAGTAGGAGATTTAGTTTGGAGACAATCCCTAATAGGAAAAAGCATCCCAAATATCTTAAAATTA
ATGGAAGGAGATAGGGCTTTACAAAGTGAAAGATACATAGAGAGTATACATGTAAGAGAATGGGAAGCCT
GTAGACAAAAGCTGAAGGAAATGGAAGGAAATTATTATGATGAAGAGAAGGATATCTATGGGCAACTAGA
TTGGGGAAATAAAGCAATAGAATACATAGTATTTCAAGAAAAAGGAAAACCTTTATGGGTAAATGTAGTA
CATAGTATTAAGAATTTGAGTCAAGCCCAACAAATTATCAAAGCAGCACAAAAACTGACACAAGAAGTAA
TAATAAGAACAGGCAAGATACCCTGGATTTTGTTGCCGGGAAGGGAAGAAGATTGGATATTAGAGTTACA
AATGGGAAACATAAATTGGATGCCATCATTTTGGTCATGTTATAAAGGCTCGGTGAGGTGGAAAAAGAGG
AATGTAATAGCGGAACTAGTCCCAGGACCAACATATTATACCGATGGAGGAAAGAAAAATGGCGGGGAA
GCCTGGGGTATATTGCCTCCACAGGTGAAAAGTTTAGAATACATGAAGAAGGGACAAATCAGCAGTTAGA
ATTGAGGGCCATAGAAGAGGCATGTAAACAGGGACCAGAAAAAATGAATATAGTAACAGATAGCAGGTAT
GCATATGAATTTATGTTGCGGAACTGGGACGAAGAAGTAATAAGAAACCCTATACAAGCGAGAATTATGG
AATTAGTGCATAATAAAGAAAAAATAGGGGTACATTGGGTGCCTGGACACAAGGGGATTCCTCAAAATGA
AGAAATAGATAGGTATATCTCAGAAATATTTTTAGCAAAAGAGGGAAGAGGGATTTTACAAAAAAGGGCA
GAAGATGCTGGATATGACTTAATATGTCCACAGGAGATAAGCATTCCGGCGGGACAAGTGAAAAGGATAG
CAATTGACTTGAAAATAAATTTGAAAAAGGACCAGTGGGCCATGATAGGGACCAAAAGCAGTTTTGCAAA
TAAGGGAGTATTCGTACAAGGAGGTATCATAGATTCGGGATATCAAGGAACAATACAAGTAGTAATATAT
AATAGTAATAATAAAGAAGTAGTAATACCACAGGGAAGAAAATTTGCACAGTTGATCCTCATGCCTCTAA
TACATGAAGAGTTGGAGCCATGGGGAGAAACAAGAAAAACAGAAAGAGGGGAACAAGGATTTGGATCAAC
AGGGATGTATTGGATAGAAAATATTCCCCTAGCAGAAGAAGAGCATAACAAATGGCATCAAGATGCTGTG
TCCTTGCATTTAGAATTTGGGATTCCCAGGACAGCTGCAGAAGATATAGTTCAACAATGTGATGTGTGTC
AAGAAAATAAAATGCCTAGTACATTAAGAGGCAGTAATAAAAGGGGCATAGACCATTGGCAAGTAGACTA
TACCCACTATGAAGACAAGATAATATTGGTCTGGGTAGAAACAAATTCAGGACTAATCTATGCAGAGAGG
GTAAAAGGAGAAACAGGACAAGAATTTAGGGTGCAAACTATGAAATGGTATGCGATGTTTGCCCCGAAAT

CATTGCAGTCTGATAACGGACCAGCATTTGTAGCAGAATCTACTCAGCTCTTAATGAAATATTTGGGCAT
AGAACATACTACAGGGATCCCCTGGAACCCACAATCTCAAGCATTAGTAGAGAGAACACATCAGACGTTA
AAGAATACATTAGAAAAACTTATACCTATGTTTAATGCGTTTGAATCAGCCCTCGCAGGGACCCTCATTA
CTCTAAATATAAAAAGAAAGGGTGGGCTAGGGACAAGCCCTATGGATATATTTATATTTAATAAGGAACA
ACAAAGAATACAGCAACAAAGTAAATCAAAACAAGAAAAAATTCGATTTTGTTATTACAGAACAAGAAAA
AGAGGGCATCC

>M51543.1 Visna/Maedi virus Icelandic strains LV1-1 and 1514, complete genome
ATGCCATCATTGTGGAAAAAGAGGACATATGCAAAAGGACTGCCGGCA
GAAGAAACAGCAGGGAAACAACAGGAGGGGGCCACGTGTGGTGCCGTCCGCGCCCCCTATGTTGTAACAG
AAGCACCACCCAAAATAGAAATAAAAGTAGGAACAAGGTGGAAAAAATTATTAGTAGATACGGGGGCAGA
TAAAACTATAGTAACATCCCATGATATGTCAGGGATACCAAAGGGAAGGATAATATTACAGGGCATAGGG
GGAATAATAGAAGGAGAAAAATGGGAACAAGTACACTTGCAATATAAAGATAAAATGATCAAAGGTACCA
TAGTGGTGTTAGCTACGAGTCCGGTAGAAGTATTAGGAAGAGATAATATGAGAGAATTGGGAATAGGATT
AATTATGGCAAATTTAGAAGAAAAGAAAATTCCCAGTACAAGAGTAAGATTAAAAGAGGGATGTAAGGGA
CCCCACATAGCGCAATGGCCTTTGACGCAAGAAAAATTAGAGGGATTAAAAGAAATAGTAGACAGATTAG
AGAAGGAAGGGAAAGTAGGAAGAGCGCCCCCACACTGGACTTGTAATACCCCTATATTTTGTATTAAGAA
GAAATCAGGAAAATGGAGGATGTTAATAGATTTTAGAGAATTAAATAAGCAAACAGAAGATTTAGCAGAA
GCACAGTTAGGGTTACCTCATCCAGGAGGATTACAGAGAAAGAAACATGTAACAATATTAGATATAGGAG
ATGCATATTTTACAATACCATTATATGAGCCATATAGACAATATACATGCTTTACCATGTTAAGTCCAAA
TAATTTAGGACCATGTGTAAGATATTATTGGAAAGTGTTACCACAAGGATGGAAATTAAGTCCTGCAGTG
TATCAATTTACAATGCAAAAAATATTAAGAGGATGGATAGAAGAACACCCTATGATACAATTTGGAATAT
ACATGGATGATATCTATATAGGGAGTGATTTAGGACTAGAAGAGCACAGGGGTATCGTGAACGAACTAGC
ATCATATATAGCGCAATATGGATTCATGCTGCCTGAAGATAAGAGGCAAGAAGGATACCCGGCGAAATGG
CTTGGATTTGAATTGCATCCGGAGAAATGGAAATTTCAAAAACATACGCTCCCAGAGATTACAGAAGGAC
CCATAACCTTGAATAAACTACAGAAATTAGTAGGAGATTTAGTTTGGAGACAATCCCTAATAGGAAAAAG
CATCCCAAATATCTTAAAATTAATGGAAGGAGATAGGGCTTTACAAAGTGAAAGATACATAGAGAGTATA
CATGTAAGAGAATGGGAAGCCTGTAGACAAAAGCTGAAGGAAATGGAAGGAAATTATTATGATGAAGAGA
AGGATATCTATGGGCAACTAGATTGGGGAAATAAAGCAATAGAATACATAGTATTTCAAGAAAAAGGAAA
ACCTTTATGGGTAAATGTAGTACATAGTATTAAGAATTTGAGTCAAGCCCAACAAATTATCAAAGCAGCA
CAAAAACTGACACAAGAAGTAATAATAAGAACAGGCAAGATACCCTGGATTTTGTTGCCGGGAAGGGAAG
AAGATTGGATATTAGAGTTACAAATGGGAAACATAAATTGGATGCCATCATTTTGGTCATGTTATAAAGG
CTCGGTGAGGTGGAAAAAGAGGAATGTAATAGCGGAACTAGTCCCAGGACCAACATATTATACCGATGGA
GGAAAGAAAAATGGGCGGGGAAGCCTGGGGTATATTGCCTCCACAGGTGAAAAGTTTAGAATACATGAAG
AAGGGACAAATCAGCAGTTAGAATTGAGGGCCATAGAAGAGGCATGTAAACAGGGACCAGAAAAAATGAA
TATAGTAACAGATAGCAGGTATGCATATGAATTTATGTTGCGGAACTGGGACGAAGAAGTAATAAGAAAC
CCTATACAAGCGAGAATTATGGAATTAGTGCATAATAAAGAAAAAATAGGGGTACATTGGGTGCCTGGAC
ACAAGGGGATTCCTCAAAATGAAGAAATAGATAGGTATATCTCAGAAATATTTTTAGCAAAAGAGGGAAG
AGGGATTTTACAAAAAAGGGCAGAAGATGCTGGATATGACTTAATATGTCCACAGGAGATAAGCATTCCG
GCGGGACAAGTGAAAAGGATAGCAATTGACTTGAAAATAAATTTGAAAAAGGACCAGTGGGCCATGATAG
GGACCAAAAGCAGTTTTGCAAATAAGGGAGTATTCGTACAAGGAGGTATCATAGATTCGGGATATCAAGG
AACAATACAAGTAGTAATATATAATAGTAATAATAAAGAAGTAGTAATACCACAGGGAAGAAAATTTGCA
CAGTTGATCCTCATGCCTCTAATACATGAAGAGTTGGAGCCATGGGGAGAAACAAGAAAAACAGAAAGAG
GGGAACAAGGATTTGGATCAACAGGGATGTATTGGATAGAAAATATTCCCCTAGCAGAAGAAGAGCATAA
CAAATGGCATCAAGATGCTGTGTCCTTGCATTTAGAATTTGGGATTCCCAGGACAGCTGCAGAAGATATA
GTTCAACAATGTGATGTGTGTCAAGAAAATAAAATGCCTAGTACATTAAGAGGCAGTAATAAAAGGGGCA
TAGACCATTGGCAAGTAGACTATACCCACTATGAAGACAAGATAATATTGGTCTGGGTAGAAACAAATTC
AGGACTAATCTATGCAGAGAGGGTAAAAGGAGAAACAGGACAAGAATTTAGGGTGCAAACTATGAAATGG
TATGCGATGTTTGCCCCGAAATCATTGCAGTCTGATAACGGACCAGCATTTGTAGCAGAATCTACTCAGC
TCTTAATGAAATATTTGGGCATAGAACATACTACAGGGATCCCCTGGAACCCACAATCTCAAGCATTAGT
AGAGAGAACACATCAGACGTTAAAGAATACATTAGAAAAACTTATACCTATGTTTAATGCGTTTGAATCA
GCCCTCGCAGGGACCCTCATTACTCTAAATATAAAAAGAAAGGGTGGGCTAGGGACAAGCCCTATGGATA
TATTTATATTTAATAAGGAACAACAAAGAATACAGCAACAAAGTAAATCAAAACAAGAAAAAATTCGATT
TTGTTATTACAGAACAAGAAAAAGAGGGCATCC

>M60610.1 Visna virus Icelandic strain 1514, complete genome
ATGCCATCATTGTGGAAAAAGAGGACATATGCAAAAGGACTGCCGGC
AGAAGAAACAGCAGGGAAACAACAGGAGGGGGCCACGTGTGGTGCCGTCCGCGCCCCCTATGTTGTAACA
GAAGCACCACCCAAAATAGAAATAAAAGTAGGAACAAGGTGGAAAAAATTATTAGTAGATACGGGGGCAG
ATAAAACTATAGTAACATCCCATGATATGTCAGGGATACCAAAGGGAAGGATAATATTACAGGGCATAGG
GGGAATAATAGAAGGAGAAAAATGGGAACAAGTACACTTGCAATATAAAGATAAAATGATCAAAGGTACC

ATAGTGGTGTTAGCTACGAGTCCGGTAGAAGTATTAGGAAGAGATAATATGAGAGAATTAGGAATAGGAT
TAATTATGGCAAATTTAGAAGAAAAGAAAATTCCCAGTACAAGAGTAAGATTAAAAGAGGGATGTAAGGG
ACCCCACATAGCGCAATGGCCTTTGACGCAAGAAAAATTAGAGGGATTAAAAGAAATAGTAGACAGATTA
GAGAAGGAAGGGAAAGTAGGAAGAGCGCCCCCACACTGGACTTGTAATACCCCTATATTTTGTATTAAGA
AGAAATCAGGAAAATGGAGGATGTTAATAGATTTTAGAGAATTAAATAAGCAAACAGAAGATTTAGCAGA
AGCACAGTTAGGGTTACCGCATCCAGGAGGATTACAGAGAAAGAAACATGTAACAATATTAGATATAGGA
GATGCATATTTTACAATACCATTATATGAGCCATATAGACAATATACATGCTTTACCATGTTAAGTCCAA
ATAATTTAGGACCATGTGTAAGATATTATTGGAAAGTGTTACCACAAGGATGGAAATTAAGTCCTGCAGT
GTATCAATTTACAATGCAAAAAATATTAAGAGGATGGATAGAAGAACACCCTATGATACAATTTGGAATA
TACATGGATGATATCTATATAGGGAGTGATTTAGGACTAGAAGAGCACAGGGGTATCGTGAACGAACTAG
CATCATATATAGCGCAATATGGATTCATGCTGCCTGAAGATAAGCGGCAAGAAGGATACCCGGCTAAATG
GCTTGGATTTGAATTGCATCCGGAGAAATGGAAATTTCAAAAACATACGCTCCCAGAGATTACAGAAGGA
CCCATAACCTTGAATAAACTACAGAAATTAGTAGGAGATCTAGTTTGGAGACAATCCCTAATAGGAAAAA
GCATCCCAAATATCTTAAAATTAATGGAAGGAGATAGGGCTTTACAAAGTGAAAGATACATAGAGAGTAT
ACATGTAAGAGAATGGGAAGCCTGTAGACAAAAGCTGAAGGAAATGGAAGGAAATTATTATGATGAAGAG
AAGGATATCTATGGGCAACTAGATTGGGGAAATAAAGCAATAGAATACATAGTATTTCAAGAAAAAGGAA
AACCTTTATGGGTAAATGTAGTACATAGTATTAAGAATTTGAGTCAAGCCCAACAAATTATCAAAGCAGC
ACAAAAACTGACACAAGAAGTAATAATAAGAACAGGCAAGATACCCTGGATTTTGTTGCCGGGAAGGGAA
GAAGATTGGATATTAGAGTTACAAATGGGAAACATAAATTGGATGCCATCATTTTGGTCATGTTATAAAG
GCTCGGTGAGGTGGAAAAAGAGGAATGTAATAGCGGAAGTAGTCCCAGGACCAACATATTATACCGATGG
AGGAAAGAAAAATGGGCGGGGAAGCCTGGGGTATATTGCCTCCACAGGTGAAAAGTTTAGAATACATGAA
GAAGGGACAAATCAGCAGTTAGAATTGAGGGCCATAGAAGAGGCATGTAAACAGGGACCAGAAAAAATGA
ATATAGTAACAGATAGCAGGTATGCATATGAATTTATGTTGCGGAACTGGGACGAAGAAGTAATAAGAAA
CCCTATACAAGCGAGAATTATGGAATTAGTGCATAATAAAGAAAAAATAGGGGTACATTGGGTGCCTGGA
CACAAGGGGATTCCTCAAAATGAAGAAATAGATAGGTATATCTCAGAAATATTTTTAGCAAAAGAGGGAA
GAGGGATTTTACAAAAAAGGGCAGAAGATGCTGGATATGACTTAATATGTCCACAGGAGATAAGCATTCC
GGCGGGACAAGTGAAAAGGATAGCAATTGACTTGAAAATAAATTTGAAAAAGGACCAGTGGGCCATGATA
GGGACCAAAAGCAGTTTTGCAAATAAGGGAGTATTCGTACAAGGAGGTATCATAGATTCGGGATATCAAG
GAACAATACAAGTAGTAATATATAATAGTAATAATAAAGAAGTAGTAATACCACAGGGAAGAAAATTTGC
ACAGTTGATCCTCATGCCTCTAATACATGAAGAGTTGAAGCCATGGGGAGAAACAAGAAAAACAGAAAGA
GGGGAACAAGGATTTGGATCAACAGGGATGTATTGGATAGAAAATATTCCCCTAGCAGAAGAAGAGCATA
ACAAATGGCATCAAGATGCTGTGTCCTTGCATTTAGAATTTGGGATTCCCAGGACAGCTGCAGAAGATAT
AGTTCAACAATGTGATGTGTGTCAAGAAAATAAAATGCCTAGTACATTAAGAGGCAGTAATAAAAGGGGC
ATAGACCATTGGCAAGTAGACTATACCCACTATGAAGACAAGATAATATTGGTCTGGGTAGAAACAAATT
CAGGACTAATCTATGCAGAAAGGGTAAAAGGAGAAACAGGACAAGAATTTAGGGTGCAAACTATGAAATG
GTATGCGATGTTTGCCCCGAAATCATTGCAGTCTGATAACGGACCAGCATTTGTAGCAGAATCTACTCAG
CTCTTAATGAAATATTTGGGCATAGAACATACTACAGGGATCCCCTGGAACCCACAATCTCAAGCATTAG
TAGAGAGAACACATCAGACGTTAAAGAATACATTAGAAAAACTTATACCTATGTTTAACGCGTTTGAATC
AGCCCTCGCAGGGACCCTCATTACTCTAAATATAAAAAGAAAGGGTGGGCTAGGGACAAGCCCTATGGAT
ATATTTATATTTAATAAGGAACAACAAAGAATACAGCAACAAAGTAAATCAAAACAAGAAAAAATTCGAT
TTTGTTATTACAGAACAAGAAAAAGAGGGCATCC

>M60609.1 Visna virus Icelandic strain 1514, complete genome
ATGCCATCATTGTGGAAAAAGAGGACATATGCAAAAGGACTGCCGGC
AGAAGAAACAGCAGGGAAACAACAGGAGGGGGCCACGTGTGGTGCCGTCCGCGCCCCCTATGTTGTAACA
GAAGCACCACCCAAAATAGAAATAAAAGTAGGAACAAGGTGGAAAAAATTATTAGTAGATACGGGGGCAG
ATAAAACTATAGTAACATCCCATGATATGTCAGGGATACCAAAGGGAAGGATAATATTACAGGGCATAGG
GGGAATAATAGAAGGAGAAAAATGGGAACAAGTACACTTGCAATATAAAGATAAAATAATCAGAGGTACC
ATAGTGGTGTTAGCTACGAGTCCGGTAGAAGTATTAGGAAGAGATAATATGAGCGAATTGGGAATAGGAT
TAATTATGGCAAATTTAGAAGAAAAGAAAATTCCCATTACAGAAGTAAGATTAAAAGAGGGATGTAAGGG
ACCCCACATAGCGCAATGGCCTTTGACGCAAGAAAAATTAGAGGGATTAAAAGAAATAGTAGACAGATTA
GAGAAGGAAGGGAAAGTAGGAAGAGCGCCCCCACACTGGACTTGTAATACCCCTATATTTTGTATTAAGA
AGAAATCAGGAAAATGGAGGATGTTAATAGATTTTAGAGAGTTAAATAAGCAAACAGAAGATTTAGCAGA
AGCACAGTTAGGGTTACCGCATCCAGGAGGATTACAGAGAAAGAAACATGTAACAATATTAGATATAGGA
GATGCATATTTTACAATACCATTATATGAGCCATATAGACAATATACATGCTTTACCATGTTAAGTCCAA
ATAATTTAGGACCATGTGTAAGATATTATTGGAAAGTGTTACCACAAGGATGGAAATTAAGTCCTTCAGT
GTATCAATTTACAATGCAAAAAATATTAAGAGGATGGATAGAAGAACACCCTATGATACAATTTGGAATA
TACATGGATGATATCTATATAGGGAGTGATTTAGGATTAGAAGAGCACAGGGGTATCGTGAACGAACTAG
CATCATATATAGCGCAATATGGATTTATGCTGCCTGAAGATAAGAGGCAAGAAGGATACCCGGCTAAATG
GCTTGGATTTGAATTACATCCGGAGAAATGGAAATTTCAAAAACATACGCTCCCAGAGATTACAGAAGGA
CCCATAACCTTGAATAAACTACAGAAATTAGTAGGAGATTTAGTTTGGAGACAATCCCTAATAGGAAAAA

GCATCCCAAATATCTTAAAATTAATGGAAGGAGATAGGGCTTTACAAAGTGAAAGATACATAGAGAGTAT
ACATGTAAGAGAATGGGAAGCCTGTAGACAAAAGCTGAAGGAAATGGAAGGAAATTATTATGATGAAGAG
AAGGATATCTATGGGCAACTAGATTGGGGAAATAAAGCAATAGAATACATAGTATTTCAAGAAAAAGGAA
AACCTTTATGGGTAAATGTAGTACATAGTATTAAGAATTTGAGTCAAGCCCAACAAATTATCAAAGCAGC
ACAAAAACTGACACAAGAAGTAATAATAAGAACAGGCAAGATACCCTGGATTTTGTTGCCGGGAAGGGAA
GAAGATTGGATATTAGAGTTACAAATGGGAAACATAAATTGGATGCCATCATTTTGGTCATGTTATAAAG
GCTCGGTGAGGTGGAAAAAGAGGAATGTAATAGCGGAAGTAGTCTCAGGACCAACATATTATACCGATGG
AGGAAAGAAAAATGGGCGGGGAAGCCTGGGGTATATTGCCTCCACAGGTGAAAAGTTTAGAATATATGAA
GAAGGGACAAATCAGCAGTTAGAATTGAGGGCCATAGAAGAGGCATGTAAACAGGGACCAGAAAAAATGA
ATATAGTAACAGATAGCAGGTATGCATATGAATTTATGTTGCGGAACTGGGACGAAGAAGTAATAAGAAA
CCCTATACAAGCGAGAATCATGGAATTAATGCATAATAAAGAAAAAATAGGGGTACATTGGGTGCCTGGA
CACAAGGGGATTCCTCAAAATGAAGAAATAGATAGGTATATCTCAGAAATATTTTTAGCAAAAGAGGGAA
GAGGGATTTTACAAAAAAGGGCAGAAGATGCTGGATATGACTTAATATGTCCACAGGAGATAAGCATTCC
GGCGGGACAAGTGAAAAGGATAGCAATTGACTTGAAAATAAATTTGAAAAAGGACCAATGGGCCATGATA
GGGACCAAAAGCAGTTTTGCAAATAAGGGAGTATTTGTACAAGGAGGTATCATAGATTCGGGATATCAAG
GAACAATACAAGTAGTAATATATAATAGTAATAATAAAGAAGTAGTAATACCACAGGGAAGAAAATTTGC
ACAGTTGATCCTCATGCCTCTAATACATGAAGAGTTGGAGCCATGGGGAGAAACAAGAAAAACAGAAAGA
GGGGAACAGGGATTTGGATCAACAGGGATGTATTGGATAGAAAATATTCCCCTAGCAGAAGAAGAGCATA
ACAAATGGCATCAAGATGCTGTGTCCTTGCATTTAGAATTTGGGATTCCCAGGACAGCTGCAGAAGATAT
AGTTCAACAATGTGATGTGTGTCAAGAAAATAAAATGCCTAGTACATTAAGAGGCAGTAATAAAAGGGGC
ATAGACCATTGGCAAGTAGACTATACCCACTATGAAGACAAGATAATATTGGTCTGGGTAGAAACAAATT
CAGGACTAATCTATGCAGAAAGGGTAAAAGGAGAAACAGGACAAGAATTTAGGGTGCAAACTATGAAATG
GTATGCGATGTTTGCCCCGAAATCATTGCAGTCTGATAACGGACCAGCATTTGTAGCAGAATCTACTCAG
CTCTTAATGAAATATTTGGGCATAGAACATACTACAGGGATCCCCTGGAACCCACAATCTCAAGCATTAG
TAGAGAGAACACATCAGACGTTAAAGAATACATTAGAAAAACTTATACCTATGTTTAACGCGTTTGAATC
AGCCCTCGCAGGGACCCTCATTACTCTAAATATAAAAAGAAAGGGTGGGCTAGGGACAAGCCCTATGGAT
ATATTTATATTTAATAAGGAACAACAAAGAATACAGCAACAAAGTAAATCAAAACAAGAAAAAATTCGAT
TTTGTTATTACAGAACAAGAAAAAGAGGGCATCC

>NC_001511.1 Ovine lentivirus, complete genome
TCCAACATCACAGCAGGGAAACAGCAGGAG
GGGGCCACGTGTGGTGCCGTCCGCGCCCCCTATGTTGTAACAGAAGCACCACCAAAGATAGATATAAAGG
TAGGGACAAATTGGAAAAAGGTATTAGTAGATACTGGGGCAGATAGAACTATAGTAAGATATCATGACAA
TTCGGGAATACCCACAGGGAGAATAAAACTACAAGGGATAGGAGGAATAATAGAAGGAGAAAAAATGGGAT
AAAGTAGTAATACAATATAAAGAAAAAAGAATAGAAGGGACAATAGTGGTACTGCCCAGTAGTCCAGTAG
AGGTATTAGGAAGGGATAATATGGCAAAATTAGACATAGGAATAATTATGGCAAATTTAGAAGAAAAGAA
AATTCCCATTACACAGGTAAAATTAAAAGAAGGCTGTAAGGGTCCTCATATAGCACAATGGCCTTTAACT
CAAGAAAAATTAGAAGGCTTAAAAGAAATAGTGGACAAGTTAGAAAAGGAAGGAAAGGTAGGTAGAGCGC
CGCCACATTGGACATGTAATACTCCTATATTTTGCATCAAGAAAAAATCAGGGAAATGGAGAATGTTAAT
AGATTTTAGGGAATTAAATAAGCAAACAGAAGATTTGGCGGAGGCACAATTAGGTTTGCCGCATCCAGGG
GGATTACAGAAAAAGAAGCATGTAACAATACTTGACATAGGGGATGCATATTTTACAATACCATTGTATG
AGCCATATAGACCATATACATGTTTTACCATGTTAAGTCCAAATAATTTGGGACCATGTACACGGTATTA
TTGGAAAGTACTACCACAAGGATGGAAGTTGAGTCCCTCAGTGTATCAATTTACAATGCAAGAAATATTA
AGGGATTGGATAGCGAAACATCCTATGATACAATTTGGAATATACATGGATGATATTTATATAGGGAGTG
ATTTGGATATAATGAAACACAGAGAGATAGTAGAAGAACTAGCTAGCTATATTGCCCAGTATGGATTTAT
GTTACCAGAAGAAAAGAGACAAGAAGGGTATCCAGCAAAGTGGCTTGGATTTGAATTGCACCCAGAGAAA
TGGAGATTTCAGAAACATACACTTCCAGAAATAAAGGAAGGGACCATAACATTAAATAAATTACAAAAAT
TAGTAGGAGATTTAGTCTGGAGACAATCATTAATAGGAAAAAGCATACCTAATATACTAAAGTTAATGGA
AGGGGATAGGGCGCTCCAAAGTGAAAGAAGGATAGAGCTCAGACATGTAAAGGAATGGGAGGAATGTAGA
AGAAAATTAGCAGAAATGGAAGGAAATTACTATGATGAAGAGAAGGATGTATATGGACAAATAGATTGGG
GAGATAAGGCAATAGAGTACATAGTGTTTCAAGAGAGAGGAAAACCTTTATGGGTAAATGTAGTACATAA
TATTAAAAACCTCAGTCAATCACAGCAAATTATTAAAGCAGCACAAAAACTTACGCAAGAGGTAATAATA
AGGATAGGAAAAATACCATGGATACTATTACCAGGAAAGGAGGAAGATTGGATCTTAGAGTTGCAAATAG
GAAATATAACATGGATGCCCTCATTTTGGTCGTGTTATAGGGGGTCAATAAGGTGGAAAAAGAGAAATGT
AATAACAGAAGTAGTAGAAGGCCCGACATATTATACAGATGGAGGTAAGAAGAATGGAAAAGGAAGTCTA
GGATTCATTGCCTCTACAGGCGTTAAATTTAGAAAACACGAAGAGGGAACAAATCAACAATTAGAATTAA
GAGCAATAGAAGAGGCGTGCAAACAGGGACCAGAGAAAATGAATATAGTAACAGATAGCAGATATGCATA
TGAATTTATGAGAAGAAATTGGGATGAAGAAGTTATAAAGAACCCAATACAGGCTAGAATTATGAAATTA
GTGCATGATAAGGAACAGATAGGGGTACATTGGGTACCTGGACACAAAGGAATTCCTCAAAATGAAGAAA
TAGATAAATATATTTCAGAAATATTTTTAGCAAGAGAAGGAAGCGGAATTCTTCCAAAAAGAGCGGAAGA
TGCAGGGTATGATCTCATATGTCCGCAGGAAGTGTGTATTCCAGCGGGGCAAGTAAGAAAAATTCCAATT

AATCTAAGAATAAACTTAAAGGAGGATCAGTGGGCCATGGTAGGGACGAAAAGTAGTTTTGCAAGCAAGG
GAGTATTTGTACAAGGGGGAATAATAGATTCAGGATATCAAGGCATTATACAGGTAGTAGTATATAACAG
CAATGACAAGGAAGTCATTATCCCACAAGGGAGAAAATTTGCACAATTAATTCTCATGCCTTTAATACAT
GAAGACCTAGAAGCTTGGGGGGAAACTAGAAGGACAGAAAGAGGAAACCAAGGATTTGGATCAACGGGAG
CATATTGGATTGAAAATATTCCCCTAGCAGAGGAAGATCACAGTAAATGGCATCAAGATGCTGGGTCATT
ACACTTAGACTTTGGGATACCCCGAACTGCAGCTGAGGATATTGTACAACAATGTGAAGTATGTCAAGAA
AATAAAATGCCCAGCACAATAAGGGGAAGCAATAGGAGAGGAATAGATCATTGGCAAGTAGACTATACAC
ATTATGAGGACAAGATAATATTAGTATGGGTAGAAACAAATTCAGGATTAATATATGCAGAAAGAGTAAA
AGGGGAAACAGGACAAGAATTTAGAATCATGACTATAAGGTGGTATGGTCTGTTTGCCCCAAAGTCATTG
CAGTCTGACAATGGACCAGCATTTGTAGCAGAGCCAACACAGCTACTAATGAAATATTTGGGGATAACAC
ACACAACAGGGATACCATGGAATCCCCAATCGCAAGCACTAGTAGAAAGAACTCATCAAACTCTAAAAAA
TACAATAGAAAAATTTGTTTCTATGTTTGCCTCATTTGATTCAGCAATAGCCGCAGCATTAATCACACTA
AATATAAAAAGAAAGGGTGGGCTAGGGACAAGCCCTATGGATATATTCATATTTAATAAGGAACAGCAAA
GAATACAACAGCAATCTACAAGAAATCAATCAAAATTTCGATTTTGTTATTACAGAGTCAGGAAAAGAGG
ACACCCAGGCGAGTGGCTGGGACCAACACAGGTACTCTGGGAAGGGGAAGGAGCAATCGTAATAAAAGAT
AAAAATCTAGAAAAGTATTTAGTCATAGCAAAAAAGGATGTTAAGTTCATACCGCAACCAAAAGAAATAC
AAACAGAATAA

>M34193.1 Ovine lentivirus, complete genome
TCCAACATCACAGCAGGGAAACAGCAGGAG
GGGGCCACGTGTGGTGCCGTCCGCGCCCCCTATGTTGTAACAGAAGCACCACCAAAGATAGATATAAAGG
TAGGGACAAATTGGAAAAAGGTATTAGTAGATACTGGGGCAGATAGAACTATAGTAAGATATCATGACAA
TTCGGGAATACCCACAGGGAGAATAAAACTACAAGGGATAGGAGGAATAATAGAAGGAGAAAAATGGGAT
AAAGTAGTAATACAATATAAAGAAAAAAGAATAGAAGGGACAATAGTGGTACTGCCCAGTAGTCCAGTAG
AGGTATTAGGAAGGGATAATATGGCAAAATTAGACATAGGAATAATTATGGCAAATTTAGAAGAAAAGAA
AATTCCCATTACACAGGTAAAATTAAAAGAAGGCTGTAAGGGTCCTCATATAGCACAATGGCCTTTAACT
CAAGAAAAATTAGAAGGCTTAAAAGAAATAGTGGACAAGTTAGAAAAGGAAGGAAAGGTAGGTAGAGCGC
CGCCACATTGGACATGTAATACTCCTATATTTTGCATCAAGAAAAAATCAGGGAAATGGAGAATGTTAAT
AGATTTTAGGGAATTAAATAAGCAAACAGAAGATTTGGCGGAGGCACAATTAGGTTTGCCGCATCCAGGG
GGATTACAGAAAAAGAAGCATGTAACAATACTTGACATAGGGGATGCATATTTTACAATACCATTGTATG
AGCCATATAGACCATATACATGTTTTACCATGTTAAGTCCAAATAATTTGGGACCATGTACACGGTATTA
TTGGAAAGTACTACCACAAGGATGGAAGTTGAGTCCCTCAGTGTATCAATTTACAATGCAAGAAATATTA
AGGGATTGGATAGCGAAACATCCTATGATACAATTTGGAATATACATGGATGATATTTATATAGGGAGTG
ATTTGGATATAATGAAACACAGAGAGATAGTAGAAGAACTAGCTAGCTATATTGCCCAGTATGGATTTAT
GTTACCAGAAGAAAAGAGACAAGAAGGGTATCCAGCAAAGTGGCTTGGATTTGAATTGCACCCAGAGAAA
TGGAGATTTCAGAAACATACACTTCCAGAAATAAAGGAAGGGACCATAACATTAAATAAATTACAAAAAT
TAGTAGGAGATTTAGTCTGGAGACAATCATTAATAGGAAAAAGCATACCTAATATACTAAAGTTAATGGA
AGGGGATAGGGCGCTCCAAAGTGAAAGAAGGATAGAGCTCAGACATGTAAAGGAATGGGAGGAATGTAGA
AGAAAATTAGCAGAAATGGAAGGAAATTACTATGATGAAGAGAAGGATGTATATGGACAAATAGATTGGG
GAGATAAGGCAATAGAGTACATAGTGTTTCAAGAGAGAGGAAAACCTTTATGGGTAAATGTAGTACATAA
TATTAAAAACCTCAGTCAATCACAGCAAATTATTAAAGCAGCACAAAAACTTACGCAAGAGGTAATAATA
AGGATAGGAAAAATACCATGGATACTATTACCAGGAAAGGAGGAAGATTGGATCTTAGAGTTGCAAATAG
GAAATATAACATGGATGCCCTCATTTTGGTCGTGTTATAGGGGGTCAATAAGGTGGAAAAAGAGAAATGT
AATAACAGAAGTAGTAGAAGGCCCGACATATTATACAGATGGAGGTAAGAAGAATGGAAAAGGAAGTCTA
GGATTCATTGCCTCTACAGGCGTTAAATTTAGAAAACACGAAGAGGGAACAAATCAACAATTAGAATTAA
GAGCAATAGAAGAGGCGTGCAAACAGGGACCAGAGAAAATGAATATAGTAACAGATAGCAGATATGCATA
TGAATTTATGAGAAGAAATTGGGATGAAGAAGTTATAAAGAACCCAATACAGGCTAGAATTATGAAATTA
GTGCATGATAAGGAACAGATAGGGGTACATTGGGTACCTGGACACAAAGGAATTCCTCAAAATGAAGAAA
TAGATAAATATATTTCAGAAATATTTTTAGCAAGAGAAGGAAGCGGAATTCTTCCAAAAAGAGCGGAAGA
TGCAGGGTATGATCTCATATGTCCGCAGGAAGTGTGTATTCCAGCGGGGCAAGTAAGAAAAATTCCAATT
AATCTAAGAATAAACTTAAAGGAGGATCAGTGGGCCATGGTAGGGACGAAAAGTAGTTTTGCAAGCAAGG
GAGTATTTGTACAAGGGGGAATAATAGATTCAGGATATCAAGGCATTATACAGGTAGTAGTATATAACAG
CAATGACAAGGAAGTCATTATCCCACAAGGGAGAAAATTTGCACAATTAATTCTCATGCCTTTAATACAT
GAAGACCTAGAAGCTTGGGGGGAAACTAGAAGGACAGAAAGAGGAAACCAAGGATTTGGATCAACGGGAG
CATATTGGATTGAAAATATTCCCCTAGCAGAGGAAGATCACAGTAAATGGCATCAAGATGCTGGGTCATT
ACACTTAGACTTTGGGATACCCCGAACTGCAGCTGAGGATATTGTACAACAATGTGAAGTATGTCAAGAA
AATAAAATGCCCAGCACAATAAGGGGAAGCAATAGGAGAGGAATAGATCATTGGCAAGTAGACTATACAC
ATTATGAGGACAAGATAATATTAGTATGGGTAGAAACAAATTCAGGATTAATATATGCAGAAAGAGTAAA
AGGGGAAACAGGACAAGAATTTAGAATCATGACTATAAGGTGGTATGGTCTGTTTGCCCCAAAGTCATTG
CAGTCTGACAATGGACCAGCATTTGTAGCAGAGCCAACACAGCTACTAATGAAATATTTGGGGATAACAC
ACACAACAGGGATACCATGGAATCCCCAATCGCAAGCACTAGTAGAAAGAACTCATCAAACTCTAAAAAA

TACAATAGAAAAATTTGTTTCTATGTTTGCCTCATTTGATTCAGCAATAGCCGCAGCATTAATCACACTA
AATATAAAAAGAAAGGGTGGGCTAGGGACAAGCCCTATGGATATATTCATATTTAATAAGGAACAGCAAA
GAATACAACAGCAATCTACAAGAAATCAATCAAAATTTCGATTTTGTTATTACAGAGTCAGGAAAAGAGG
ACACCCAGGCGAGTGGCTGGGACCAACACAGGTACTCTGGGAAGGGGAAGGAGCAATCGTAATAAAAGAT
AAAAATCTAGAAAAGTATTTAGTCATAGCAAAAAAGGATGTTAAGTTCATACCGCAACCAAAAGAAATAC
AAACAGAATAA

>MT993912.1 Small ruminant lentivirus isolate USMARC-200106932-2,
complete genome
GGGAAACAACAGGAGGGGGCCACGTGTGGT
GCCGTCCGCGCCCCCTATGTTGTAACAGAAGCACCACCAAGAATAGAAGCAAGGGTAGGAACAACTTGGA
AAGAATTATTAGTAGATACAGGAGCAGATAGAACTATAGTGAGAAAACATGATAGTACAGGGATACCAAG
GGGAAGAATAAAGCTTCAAGGGATAGGAGGGATTATAGAAGGAGAAAAATGGGATCAAGTACAAATACAA
TATAAAGGAAAAATAATAAGAGGAACAATAGTAGTGCTACCGACAAGTCCGGTAGAAGTGTTAGGGAGAG
ATAATATGGGAGAGTTAGGCATCGGGTTAATTATGGCCAATTTAGAAGAAAAGAAAATCCCTATTACACA
GGTAAAATTAAAGGAGGGATGTAAGGGACCCCATATAGCACAGTGGCCTTTAACTCAAGAAAAATTGGAA
GGGTTAAAAGAAATAGTAAATAGATTAGAAAAAGAAGGAAAGCTAGGCAGAGCACCACCGCATTGGACAT
GTAACACCCCTATTTTTTGTATTAAGAAAAAATCAGGAAAATGGAGAATGTTAATAGACTTTAGAGAATT
AAATAAGCAAACGGAAGATCTAGCGGAAGCACAGTTGGGGCTACCACACCCAGGGGGATTACAAAAGAAG
AAACATGTAACAATATTAGATATAGGAGATGCGTACTTCACTATCCCATTGTATGAACCATATCGGCAAT
ATACGTGCTTTACGCTTTTAAGCCCTAATAATCTGGGACCATGTGAAAGGTATTATTGGAAAGTGTTGCC
ACAAGGCTGGAAATTAAGTCCGTCAGTATATCAGTTTACAATGCAAAAAATATTAAGAGACTGGATAAAG
GAACATCCTATGATACAATTTGGAATATACATGGATGATATTTATATAGGAAGTGATTTAGACATAGGGG
AGCACAGAAAGATTGTAGAAAACTTGGCTGGATATATCGCACAATATGGATTTATGCTGCCTGAAGAGAA
AAGGCAAGAAGGATATCCAGCTCAATGGCTTGGATTTGAGTTACATCCAGATAAATGGAGATTTCAAAAA
CATGCTTTGCCAGATATCCAGGAAGGGTCAATTACTTTAAACAAGTTGCAAAAATTAGTAGGGGATCTGG
TGTGGAGGCAATCATTGATAGGAAAAAGCATTCCAAACATATTAAAATTGATGGAAGGAGATAGGGCATT
ACAAAGTGAAAGGCGAGTAGAACAAATTCATGTACAGGAATGGGAAAATTGTAAGAGAAAATTAAAAGAA
ATGGAAGGAAATTATTATAATGAAGAAAGGGACATCTATGGACAAATAGATTGGGGAGATAAAGCAATTG
AATATATAGTATTCCAAGAGAAAGGGAAACCTTTGTGGGTAAATGTAGTCCATAATATTAAAAACCTAAG
CCAACCACAGCAAATTATTAAAGCAGCACAAAAACTAACCCAAGAGGTGATAATAAGAACAGGAAAAATA
CCATGGATACTGCTACCAGGAAAGGAAGAGGACTGGATATTAGAATTACAAATAGGGAATATAACATGGA
TGCCTTCATTCTGGTCATGCTATAGAGGGTCAGTAAGGTGGAAAAGGAGAAATATAGTAACAGAAGTAGT
AGAAGGACCAACATACTATACAGATGGAGGTAAGAAAAATGGGATTGGAAATTTAGGATATATTGCCTCG
ACAGGAGAAAAATATAGAAAACATGAAGAAGGAACAAATCAACAGTTAGAATTGAGAGCAATAGAAGAGG
CATGTAAACAGGGACCAAGCAAGATGAATATAGTAACTGATAGTAGGTACGCATATGAATTTATGCTAAG
AAATTGGGATGAAGAAGTAATAAGAAACCCTATACAGGCCAGAATTATGAGAATAATTCATGGAAAAGAA
AAGGTGGGAGTGCACTGGGTGCCAGGACATAAAGGGATTCCTCAGAATGAGGAGATAGATAAATATATTT
CAGAAATATTTCTAGCAAAAGAAGGAGAAGGGATTCTCCCTAAAAGAGAAGAAGATGCAGGGTATGATTT
AATCTGTCCTCAGGAAATACATATCCCGGCGGGAGAGGTGAAAAAGATTCCAATAGATTTAAGAATAAAT
TTGAAAGGAAATCAATGGGCCATGATAGCAACAAAAAGTAGTTTTGCAAGTAAGGGAGTCTTTGTACAAG
GAGGGATAATAGATTCAGGATATCAAGGAACAATACAAGTAGTAATATACAATAGCAATAAGGTAGAGGT
AATAATACCACAAGGAAGAAAGTTTGCACAATTGATCCTTATGCCTTTGATACATGAAGAATTGGAGCCA
TGGGGAATGGCAAGAAGAACTGAAAGAGGGAATGAAGGGTTTGGTTCAACAGGAGCCTATTGGATAGAAA
ATATCCCTAAAGCAGAAGAGGATCATGATAAATGGCATCAAGATGCAACTTCATTGCACTTAGAATTTGG
GATCCCTAAATCAGCGGCTGAAGATATAGTACAACAGTGTGAAGTGTGTCAAGAAAATAAAATGCCAAGT
ACCCTCAGAGGAGGCAATAAAAGAGGAATAGACCATTGGCAAGTAGATTACACTCATTATGAAGATAAAA
TATTATTAGTATGGGTAGAAACAAATTCAGGGTTAATCTATGCAGAAAAGGTAAAAGGAGAAACAGGCCA
AGAATTCAGAATACATGTTATGAAATGGTATGCCATGTTTTCTCCAAAATCATTGCAGTCCGATAATGGA
CCGGCCTTTGTAGCAGAGCCAACACAGCTCTTAATGAAATATCTAGGGGTAGAACACAGTACAGGGGTTC
CGTGGAACCCACAATCTCAAGCCTTGGTGGAAAGAACCCATCAAACGTTGAAACATACCCTAGAAAAATT
TATTCCCATGTTTGTAGCATTTGAATCTGCTCTTGCGGCCTCGCTCATAAATCTAAATATAAAAAGAAAG
GGTGGGCTAGGGACAAGCCCTATGGATATATTCATATTTAATAAAGAACAACAAAGAATACAGCAACAAT
TTCAAATAAATCAAGAAAAAATTCGGTTTTGTTATTACAGGATCAGGAAAAGAGGACATCCAGGCGAGTG
GCAAGGGCCAACACAGGTATTGTGGGAAGGGGAAGGTGCAATAGTGGTAAAAGATAAACATACAGAACGA
TACTTGGTAGTAACTAATAAAGATGTCAGGTTTGTCCCACCACCAAAAGAAATACAAAAGAAATAA

>MT993916.1 Small ruminant lentivirus isolate USMARC-200177363-2,
complete genome
GGGAAACAACAGGAGGGGGCCACGTGT
GGTGCCGTCCGCGCCCCCTATGTTGTAACAGAAGCACCACCTAGAATAGAAGCAAGAGTAGGGACAACTT

GGAAGGAATTATTAGTAGATACAGGAGCAGATAGAACTATAGTGAGAAAACATGATAATACAGGGATTCC
AAAGGGAAGAATAAAACTACAAGGAATAGGAGGAATTATAGAAGGGGAAAAATGGGATCAAGTACAAATA
CAATATAAAGAAAAAATAACAAGAGGAACAATAGTGGTACTGCCGACAAGTCCGGTGGAAGTGTTAGGGA
GAGATAATATGGCAGAATTAGGCATAGGGTTAATTATGGCCAATTTAGAAGAAAAGAAAATTCCCGTAAC
ACAGGTAAAATTAAAAGAGGGGTGTAAAGGACCTCATATAGCTCAGTGGCCTTTAACTCAAGAAAAATTG
GAAGGGTTAAAAGAAATAGTAAACAGATTAGAAAAGGAGGGAAAGTTAGGCAGGGCACCACCTCATTGGA
CATGTAACACCCCTATCTTCTGTATTAAGAAAAAATCAGGGAAATGGAGAATGTTAATAGACTTTAGAGA
ATTAAATAAGCAAACGGAAGACTTAGCGGAAGCACAGTTAGGACTACCACATCCAGGAGGATTACAGAAA
AAGAAGCATGTAACAATATTAGATATAGGGGATGCGTACTTCACTATTCCATTGTATGAACCATATCGGC
AATATACGTGCTTTACGCTTCTAAGCCCTAATAATCTGGGACCATGTGAAAGGTATTATTGGAAAGTGTT
GCCACAGGGCTGGAAATTGAGTCCGTCGGTATATCAGTTTACAATGCAAACAATATTAAGAGAATGGATA
AAGGAACACCCTATGATACAATTTGGAATATATATGGATGATATTTATATAGGAAGTGATTTAGAATTAA
GGGAACACAGAGAGATTGTAGAAAACTTGGCTGGATATATTGCACAATATGGATTTATGTTGCCTGAAGA
GAAGAGGCAAGAAGGGTATCCAGCTCAATGGCTTGGATTTGAGTTACATCCAGACAAATGGAGATTTCAA
AAACATGCTTTGCCAGACATCAAGGAAGGGTCAATTACTTTAAACAAGTTGCAAAAATTAGTAGGAGATT
TGGTATGGCGACAATCCTTGATAGGAAAAAGTATTCCAAACATATTAAAGTTAATGGAGGGCGATAGGGC
ACTCCAAAGTGAAAGACAAATAGAAAAAATTCATGTGCAGGAATGGGAAACATGTAAGAAAAAATTAGCA
GAGATGGAAGGAAATTATTATGATGAAGAAAAGGACATCTATGGACAAATAGATTGGGGAAATAAAGCAA
TTGAATATATAGTGTTTCAGGAAAAAGGGAAACCTTTATGGGTGAATGTAGTGCATAGCATTAAAAATCT
AAGTCAACCACAACAAATTATTAAAGCAGCCCAGAAGCTGACGCAAGAAGTAATCATTAGAACAGGGAAG
ATACCATGGGTACTACTACCAGGGAAGGAAGAAGATTGGATATTAGAATTACAAATAGGGAATATAACGT
GGATGCCTTCCTTCTGGTCATGCTATAGAGGGTCAGTAAGGTGGAAAAGGAGAAATGTAGTAACAGAAGT
AGTGGAAGGACCAACATATTATACAGATGGAGGCAAGAAAAATGGCCTTGGAAACTTAGGATATATTGCC
TCGACAGGAGAAAAATATAGACTACATGAAGAAGGGACAAATCAACAGTTAGAATTGAGAGCAATAGAAG
AGGCATGTAAGAGGGGACCAAGTAAGATGAATATAGTAACTGATAGTAGGTACGCATATGAATTTATGCT
AAGGAACTGGGATGAAGAAGTAATAAGAAACCCTATACAGGCCAGAATCATGAAAATAATTCATGAGAAA
GAAAGAGTGGGAGTGCATTGGGTGCCAGGACATAGAGGCATCCCTCAGAATGAAGAAATAGATAAATATA
TATCAGAAGTATTTTTAGCAAAACAAGGGGAAGGGATTGTCCAGAAAAGAGCAGAAGACGCTGGATATGA
CTTAATATGTCCGCAGGAAGTAGTCATCCCACCTGGAGAAGTAAGAAAGGTTCCAATAGATTTAAGAATA
AATTTGAAAGAAAATCAATGGGCCATGATAGCAACAAAAAGTAGTTTTGCAAGTAAGGGAGTCTTTATAC
AGGGAGGGATAATAGATTCAGGATATCAAGGAACCATACAAGTAGTAATATACAACAGCAATAAGGTAGA
AGTAATAATACCACAAGGAAGAAAGTTTGCGCAATTAATTCTTATGCCTTTGATACATGAAGAATTAGAA
CCATGGGGAACGGCAAGAAGAACTGAGAGAGGGAATGAAGGATTTGGTTCAACAGGAGCCTATTGGATAG
AAAATATCCCTAAAGCAGAAGAGGATCATGATAAATGGCATCAAGATGCAACTTCATTGCACTTAGAATT
TGGGATCCCTAAGTCAGCGGCTGAAGATATAGTACAACAGTGTGAAGTGTGCCAAGAAAATAAGATGCCA
AGTACCCTCAGAGGAGGAAATAAAAGAGGAATAGACCATTGGCAAGTAGACTATACTCATTATGAAGATA
AAATATTATTAGTATGGGTAGAAACAAATTCAGGATTAATTTATGCAGAAAAGGTAAAAGGAGAAACAGG
CCAAGAATTCAGAATACATGTTATGAAATGGTATGCCATGTTTTATCCAAAATCATTGCAGTCTGATAAT
GGACCTGCGTTCGTAGCAGAGCCAACACAGCTCTTAATGAAATATCTAGGGGTAGAACATACTACAGGGG
TTCCGTGGAATCCACAATCTCAAGCCTTGGTTGAAAGAACCCATCAAACGTTGAAACATACCCTAGAAAA
ATTTATTCCTATGTTTGTAGCATTTGAATCTGCTCTTGCGGCCTCGCTCATAACTCTAAATATAAAAAGA
AAGGGTGGGCTAGGGACAAGCCCTATGGATATATTCATATTTAATAAAGAACAGCAAAGAATACAGCAAC
AATTTCAAATAAATCACGAAAAAATTCGGTTTTGTTATTACAGGATCAGGAAAAGAGGACATCCAGGCGA
TTGGCAAGGGCCTACGCAGGTATTATGGGAAGGGGAAGGTGCAATAGTAGTAAAAGATAAGCATACAGAA
CGATACTTGGTAATAACTAATAAGGATGTCAGGTTTGTCCCACAACCAAAAGAAATACAAAAGAAATAA

>MT993907.1 Small ruminant lentivirus isolate USMARC-200216049-r, complete genome
GGGAAACAGCAGGAGGGGGCCACGTGTGGTG
CCGTCCGCGCCCCCTATGTTGTAACAGAAGCACCACCGAAAATAGAAGCAAGAGTGGGGACAACTTGGAA
GGAATTATTAGTGGATACAGGAGCAGATAGAACTATAGTAAGACAACATGATAGCACAGGAATACCAAAG
GGAAGAATAAAGCTTCAAGGAATAGGAGGGATTATAGAAGGGGAAAAATGGGATCAAGTACAAATACAAT
ATAAAGAAAAAATAATGAAAGGAACAATAGTAGTACTGCCAACAAGTCCAGTAGAAGTGTTAGGGAGAGA
CAATATGGGAAAATTGGGTATAGAATTAATTATGGCCAATTTAGAAGAAAAGAAAATTCCCATAACACAG
GTAAAATTAAAGGAGGGGTGTAAGGGACCTCATATAGCTCAGTGGCCTTTAACTCAAGAAAAATTGGAAG
GGTTAAAAGAAATAGTAAACAGATTAGAAAAAGAAGGAAAGCTAGGCAGAGCACCACCTCATTGGACATG
TAACACCCCTATCTTCTGTATTAAGAAAAAATCAGGGAAATGGAGAATGTTAATAGACTTCAGAGAATTA
AATAAACAAACGGAAGATTTAGCGGAAGCACAGTTAGGGCTACCACATCCAGGAGGATTACAGAAGAAGA
AGCATGTAACAATATTAGATATAGGGGATGCGTACTTCACTATTCCATTGTATGAACCATATCGGCAATA
TACGTGCTTTACGCTTCTAAGCCCTAATAATTTGGGACCATGTGTTAGATATTATTGGAAAGTGTTGCCT
CAAGGATGGAAGTTAAGCCCGTCGGTATATCAGTTTACAATGCAGAAAATATTAAAAGATTGGATAGAGG

AACACCCTATGATACAATTTGGGATATATATGGATGATATTTATATAGGAAGTGATCTAGAAATAACAGA
GCATAGGAAAATAGTAGAAGAATTAGCAAAGTATATAGCGCAGTTTGGCTTTATGTTGCCTGAAGATAAA
AGGCAAGAAGGGTATCCAGCCAAGTGGCTAGGATTTGAGTTACATCCTGACAAATGGAGGTTTCAAAAAC
ATACATTGGCAGAGTTAAAAGAAGGACCGATCACATTAAATAAATTGCAAAAATTAGTAGGGGACTTAGT
CTGGAGACAATCATTAATAGGAAAAAGTATTCCAAACATATTAAAGTTAATGGAGGGAGATAGGGCACTC
CAAAGTGAAAGGCAAATAGAAAAAGTTCATGTACAGGAGTGGGAAACCTGTAAGAAAAAGTTAGAAGAAA
TGGAAGGAAATTATTATAATGAAGAAAAGGACATTTATGGGCAAATAGATTGGGGAAATAAAGCAATTGA
ATATATAGTGTTTCAGGAAAAAGGGAAACCTTTATGGGTGAATGTAGTGCATAGCATTAAAAATCTAAGT
CAACCACAACAAATTATTAAAGCAGCCCAGAAGCTGACGCAAGAAGTAATCATTAGAACAGGGAAGATAC
CATGGGTACTACTGCCAGGGAAGGAAGAAGATTGGATATTAGAATTACAAATAGGGAATATAACGTGGAT
GCCTTCCTTCTGGTCATGCTATAGAGGGTCAGTAAGGTGGAAAAGGAGAAATGTAGTAACAGAAGTAGTG
GAAGGACCAACATATTATACCGATGGAGGGAAGAAAAATGGCCTTGGAAATCTAGGCTACATTGCATCGA
CAGGAGAAAAATATAGACTACATGAAGAAGGCACAAATCAACAGTTAGAATTGAGAGCAATAGAAGAGGC
ATGTAAACAGGGACCAAGCAAGATGAATATAGTAACTGATAGTAGGTACGCATATGAATTTATGCTAAGG
AACTGGGATGAAGAAGTAATAAGAAACCCTATACAGGCCAGAATCATGAAAATAATTCATGAGAAAGAAA
GAGTGGGAGTGCATTGGGTACCAGGACATAAAGGCATCCCTCAAAATGAAGAAGTAGATAAATATATATC
AGAGGTATTTTTAGCAAAACAAGGGGAAGGGATTGTCCAGAAAAGAGCAGAGGACGCTGGATATGATTTA
ATATGTCCTCAGGAAGTAATCATCCCACCTGGAGAAGTAAGAAAGGTTCCAATAGATTTAAGAATAAATT
TGAAAGAAAATCAATGGGCCATGATAGCAACAAAAAGTAGTTTTGCAAGTAAGGGGGTATTTGTTCAAGG
AGGAATAATAGATTCAGGATATCAAGGAACAATACAAGTAGTAATATACAATAGTAATGATGTGGAAGTG
GTAATTCCCCAGGGAAGGAAGTTTGCACAGTTAATTCTTATGCCTTTGATACATGAAGAATTAGAACCAT
GGGGAATGGCAAGAAGAACTGAGAGAGGGAATGAAGGATTTGGTTCAACAGGAGCCTATTGGATAGAAAA
CATCCCTAAAGCAGAAGAGGATCATGATAAGTGGCATCAAGATGCAAATTCATTGCACTTAGAATTTGGG
ATCCCTAAATCAGCTGCTGAAGATATAGTACAACAATGTGAGGTGTGTCAAGAAAATAAGATGCCAAGTA
CCCTCAGAGGGGGAAATAAAAGAGGAATAGATCATTGGCAGGTAGATTATACACATCATGAAGATAAAAT
ATTATTAGTATGGGTAGAAACAAATTCAGGATTAATTTATGCAGAAAAGGTAAAAGGAGAAACAGGCCAA
GAATTCAGAATACATGTTATGAAATGGTATGCCATGTTTCATCCAAAATCATTGCAGTCCGATAATGGAC
CAGCTTTTGTAGCAGAGCCAACACAGCTCTTAATGAAATATCTGGGGGTGGAACACACTACGGGGGTGCC
ATGGAACCCACAATCTCAAGCCTTAGTGGAAAGAACTCATCAAACGTTAAAACATACCCTGGAAAAATTT
ATCCCCATGTTTGCAGCATTCGAATCTGCTCTTGCGGCCGCGCTCATAACTCTAAATATAAAAAGAAAGG
GTGGGCTAGGGACAAGCCCTATGGATATATTTATATTTAATAAAGAACAACAAAGAATACAGCAACAATT
TCAAGTAAATCACGAAAAAATTCGATTTTGTTATTACAGGATCAGGAAAAAAGGACATCCAGGCGAGTGG
CAGGGGCCAACACAGGTATTGTGGGAAGGGGAAGGCGCAATAGTAGTAAAAGATAAACCCACAGAAAGAT
ATTTAGTAGTAACCAACAAGGATGTCAGGTTCATCCCACCACCGAAAGGAATACAAAAAGAATAG

>MT993914.1 Small ruminant lentivirus isolate USMARC-200016283-2, complete genome
GGGAAACAGCAGGAGGGGGCCACGTGTGGT
GCCGTCCGCGCCCCCTATGTTGTAACAGAAGCACCACCTAGAATAGAAGCAAGAGTAGGGACAACTTGGA
AAGAGTTGTTGGTGGATACAGGAGCAGATAGAACAATAATAAGAAAACATGATAATACAGGGATACCAAA
GGGAAGAATAAAATTACAAGGAATAGGAGGGATTATAGAAGGAGAAAAATGGGATCAAGTACAGATACAA
TATAAAGGAAAAATAATAAGGGGAACAATAGTAGTCCTATCGACAAGTCCAGTAGAAGTATTAGGGAGAG
ATAATATGGGAAAATTGGGCATAGGATTAATTATGGCCAATTTAGAAGAAAAGAAAATTCCCATTACACA
GGTAAAATTAAAGGAGGGGTGTAAGGGACCTCATATAGCACAGTGGCCTTTGACGCAAGAGAAATTGGAA
GGATTAAAAGAAATAGTAGACAGATTAGAAAAAGAAGGAAAGTTAGGCAGAGCACCACCACATTGGACAT
GTAACACCCCTATTTTTTGTATTAAGAAAAAATCAGGGAAATGGAGAATGTTAATAGACTTCAGAGAATT
AAATAAACAAACGGAAGATTTAGCGGAAGCACAGTTAGGGCTACCACACCCAGGAGGCTTACAGAAGAAG
AAGCATGTCACAATATTAGATATAGGGGATGCGTACTTCACTATTCCATTGTATGAACCATATCGGCAAT
ATACGTGCTTTACGCTTCTAAGCCCTAATAATCTGGGACCATGTGAAAGGTATTATTGGAAAGTGTTGCC
ACAAGGCTGGAAATTGAGTCCGTCGGTATATCAGTTTACAATGCAAAAAATATTAAGAGACTGGATAAAG
GAACATCCTATGATACAATTTGGAATATATATGGATGATATTTATATAGGAAGTGATTTAGAACTAAAGG
AACACAGAGAGATTGTAGAAAACTTGGCTGTATATATTGCACAATATGGATTTATGCTGCCGGAAGAAAA
GAGGCAAGAAGGGTATCCAGCTCAATGGCTTGGATTTGAGTTACATCCAGATAAATGGAGATTTCAAAAA
CATGCTTTGCCAGACATCAAGGAAGGGTCAATTACTTTAAATAAGTTGCAAAAATTAGTAGGGGATCTGG
TGTGGCGACAATCATTGATAGGAAAAAGTATTCCAAACATATTAAAGTTAATGGAGGGCGACAGGGCATT
ACAAAGTGAGAGACAAATAGAAAAAATTCATGTACAGGAATGGGAAATATGTAAGAGAAAATTAAAAGAA
ATGGAAGGAAATTATTATAATGAAGAGAGGGACGTCTATGGACAAATAGATTGGGGAAATAAAGCAATTG
AATATATAGTGTTTCAGGAGAAAGGGAAACCTTTATGGGTGAATGTAGTGCATAGCATTAAAAATCTAAG
TCAGCCACAACAAATTATTAAAGCAGCCCAGAAGCTGACGCAAGAAGTAATCATTAGAACAGGGAAGATA
CCATGGGTACTACTGCCAGGGAAGGAAGAAGATTGGATATTAGAATTACAAATAGGGAATATAACGTGGA
TGCCTTCCTTCTGGTCATGCTATAGAGGGTCAGTAAGGTGGAAAAGGAGAAATGTAGTAACAGAAGTAGT

GGAAGGACCAACATATTATACAGATGGAGGTAAGAAAAATGGCCTTGGAAACCTAGGCTACATTGCCTCG
ACAGGAGAAAAATATAGACTACATGAAGAAGGGACAAATCAACAGTTAGAGTTGAGAGCAATAGAAGAGG
CATGTAAGAGGGGACCAAGCAAGATGAATATAGTAACTGATAGTAGGTACGCATATGAATTTATGCTAAG
GAACTGGGATGAAGAAGTAATAAGAAACCCTATACAGGCCAGAATCATGAAAATAATTCATGGGAAAGAA
AGAGTGGGAGTACATTGGGTACCAGGACATAGAGGGATCCCTCAGAATGAGGAAATAGATAAATATATAT
CAGAAATATTTTTAGCAAAGCAAGGGGAAGGGATTGTCCAGAAAAGAGCAGAGGACGCTGGATATGACTT
AATATGTCCGCAGGAAGTAGTCATCCCACCTGGAGAAGTGAGAAAGGTTCCAATAGATTTAAGAATAAAT
TTGAAAGAAAATCAATGGGCCATGATAGCAACAAAAAGTAGTTTTGCAAGTAAGGGAGTCTTTGTACAAG
GAGGGATAATAGATTCAGGATATCAAGGAACTATACAAGTAGTAATATACAACAGCAATAAGGTAGAGGT
AATAATACCACAAGGAAGAAAGTTTGCACAATTAATTCTTATGCCTTTGGTACATGAAGAATTAGAACCA
TGGGGAACGGCAAGAAGAACTGAGAGAGGGAATGAAGGATTTGGTTCAACAGGAGCCTATTGGATAGAAA
ATATCCCTAAAGCAGAAGAGGATCATGATAAATGGCATCAAGATGCAACTTCATTGCACTTAGAATTTGG
GATCCCTAAATCAGCGGCTGAAGATATAGTACAACAGTGTGAAGTGTGCCAAGAAAATAAAATGCCAAGT
ACCCTTAGAGGGGGAAATAAAAGAGGAATAGACCATTGGCAAGTAGATTATACTCATTATGAAGATAAAA
TACTATTAGTATGGGTAGAAACAAATTCAGGATTAATTTATGCAGAAAAAGTAAAAGGAGAAACAGGCCA
AGAATTCAGAATACATGTTATGAAATGGTATGCCATGTTTTATCCAAAATCATTGCAGTCCGATAATGGA
CCAGCTTTTGTAGCAGAGCCAACACAGCTCTTAATGAAATATCTAGGGATAGAACATACAACAGGGGTTC
CGTGGAACCCACAATCTCAAGCCTTGGTTGAGAGAACCCATCAAACGTTGAAACATACCCTAGAGAAACT
TATCCCCATGTTTGTAGCATTTGAATCTGCTCTTGCCGCCTCGCTCATAACTCTAAATATAAAAAGAAAG
GGTGGGCTAGGGACAAGCCCTATGGATATATTCATATTTAATAAAGAACAACAAAGAATACAGCAACAAT
TTCAAGTAAATCATGAAAAAATTCGGTTTTGTTATTACAGGAGCAGGAAAAGAGGACATCCAGGCGAGTG
GCAGGGGCCTACACAGGTATTATGGGAAGGGGAAGGTGCAATAGTAGTAAAAGATAAGCAGACAGAACGA
TACTTGGTAATAACTAATAAGGATGTCAAGTTTGTCCCACAACCAAAAGAAATACAAAAGAAATAA

>MT993906.1 Small ruminant lentivirus isolate USMARC-200117502-r,
complete genome
GGGAAACAGCAGGAGGGGGCCACGTGTGGTGCC
GTCCGCGCCCCCTATGTTGTAACAGAAGCACCACCGAAAATAGAAGCAAGAGTGGGGACAACTTGGAAGG
AATTATTAGTGGATACAGGAGCAGATAGAACTATAGTAAGAAAACATGATAGCACAGGAATACCAAAGGG
AAGAATAAAGCTTCAAGGAATAGGAGGAATTATAGAAGGGGAAAAATGGGATCAAGTACAAATACAATAT
AAAGAAAAAAATAATAAAAGGAACAATAGTAGTACTGCCAACAAGTCCAGTGGAAGTGTTAGGGAGAGATA
ATATGGGAAAATTGGGCATAGGATTAATTATGGCCAATTTAGAAGAAAAGAAAATTCCTATTACACAGGT
AAAATTAAAAGAGGGGTGTAAGGGACCCCATATAGCACAGTGGCCTTTGACGCAAGAGAAATTGGAAGGA
TTAAAAGAAATAGTAAACAGATTAGAAAAGGAGGGAAAGTTAGGCAGGGCACCACCTCATTGGACATGTA
ACACCCCTATTTTTTGCATTAAGAAAAAATCAGGGAAATGGAGAATGTTAATAGACTTCAGAGAATTAAA
TAAACAAACGGAAGACTTAGCGGAAGCACAGTTAGGGCTACCACACCCAGGAGGATTACAGAAGAAGAAG
CATGTAACAATATTAGATATAGGAGATGCGTACTTCACTATTCCATTGTATGAACCATATCGGCAATATA
CGTGCTTTACGCTTCTAAGCCCTAATAATCTGGGACCATGTGAAAGGTATTATTGGAAAGTGTTGCCACA
GGGCTGGAAATTGAGTCCGTCGGTATATCAGTTTACAATGCAAAAAATATTAAGAGACTGGATAAAGGAA
CATCCTATGATACAATTTGGAATATATATGGATGATATTTATATAGGAAGTGATTTAGAACTAAGGGAAC
ACAGAGAGATTGTAGAAAACTTGGCTGTATATATTGCACAATATGGATTTATGCTGCCTGAAGAAAAGAG
ACAAGAAGGGTATCCAGCTCAATGGCTTGGATTTGAGTTACATCCAGACAAATGGAGATTTCAAAAACAT
GCTTTGCCAGAGATCAAGGAAGGATCAATTACTTTAAACAAGTTGCAAAAATTAGTAGGAGATCTGGTGT
GGCGACAATCATTGATAGGAAAAAGTATTCCAAACATATTAAAGTTAATGGAGGGCGACAGGGCACTCCA
AAGTGAAAGACAAATAGAAAAGATTCATGTACAGGAATGGGAAATATGTAAGAGAAAATTAGTAGAGATG
GAAGGAAATTATTATGATGCAGAAAAGGACATCTATGGACAAATAGATTGGGGAAATAAAGCAATTGAAT
ATATAGTGTTTCAGGAAAAAGGGAAACCTTTATGGGTGAATGTAGTGCATAGCATTAAAAATCTAAGTCA
ACCACAACAAATTATTAAAGCAGCCCAGAAGCTGACGCAAGAAGTAATCATTAGAACAGGGAAGATACCA
TGGGTACTACTGCCAGGGAAGGAAGAAGATTGGATATTAGAATTACAAATAGGGAATATAACGTGGATGC
CTTCCTTCTGGTCATGCTATAGAGGGTCAGTAAGGTGGAAAAGGAGAAATGTAGTAACGGAAGTAGTGGA
AGGACCAACATATTATACAGATGGAGGTAAGAAAAATGGCCTTGGAAACTTAGGCTACATTGCCTCGACA
GGAGAAAAATATAGATTACATGAAGAAGGCACAAATCAACAGTTAGAATTGAGAGCAATAGAAGAGGCAT
GTAAAAGGGGACCAAGCAAGATGAATATAGTAACTGATAGTAGGTACGCATATGAATTTATGCTAAGGAA
CTGGGATGAAGAAGTAATAAGAAACCCTATACAGGCCAGAATCATGAAAATAGTTCATGGGAAAGAAAGA
GTGGGAGTACATTGGGTACCAGGACATAAAGGCATCCCTCAAAATGAAGAAGTAGATAAGTATATATCAG
AAGTATTTTTAGCAAAACAAGGGGAAGGGATTGTCCAGAAAAGAGCAGAAGACGCTGGATATGACTTAAT
ATGTCCGCAGGAAGTAGTCATCCCACCTGGAGAAGTGAGAAAGGTTCCAATAGATTTAAGAATAAATTTG
AAAGAAAATCAATGGGCCATGATAGCAACAAAAAGTAGTTTTGCAAGTAAGGGAGTCTTTATACAAGGAG
GGATAATAGATTCAGGATATCAAGGAACTATACAAGTAGTAATATACAACAGCAATAAGGTAGAGGTAAT
AATACCACAAGGAAGAAAGTTTGCACAATTAATTCTTATGCCTTTGATACATGAAGAATTAGAACCATGG
GGAACGGCAAGAAGAACTGAGAGAGGGAATGAAGGATTTGGTTCAACAGGAGCCTATTGGATAGAAAATA

TCCCTAAAGCAGAAGAGGATCATGATAAATGGCATCAAGATGCAACTTCATTGCACTTAGAGTTTGGGAT
CCCTAAATCAGCAGCTGAAGATATAGTACAGCAGTGTGAAGTGTGCCAAGAAAATAAGATGCCAAGTACC
CTCAGAGGGGGAAATAAAAGAGGAATAGACCACTGGCAAGTAGATTATACTCATTATGAAGAAAAAATAT
TATTAGTATGGGTAGAAACAAATTCAGGATTAATTTATGCAGAAAAGGTAAAAGGAGAAACAGGCCAAGA
ATTCAGAATACATGTTATGAAATGGTATGCCATGTTTTATCCAAAATCATTGCAGTCCGATAATGGACCA
GCCTTTGTAGCAGAGCCAACACAGCTCTTGATGAAATATCTAGGGGTGGAACATACTACAGGGGTTCCGT
GGAACCCACAATCTCAAGCCTTGGTTGAAAGAACCCATCAAACGTTGAAACATACCCTAGAGAAACTTAT
CCCCATGTTTGTAGCATTTGAATCTGCTCTTGCGGCCTCGCTCGTAACTCTAAATATAAAAAGAAAGGGT
GGGCTAGGGACAAGCCCTATGGATATATTCATATTTAATAAAGAACAACAAAGAATACAGCAACAATTTC
AAATAAATCACGAAAAAATTCGGTTTTGTTATTACAGGATCAGGAAAAGAGGACATCCAGGCGAGTGGCA
GGGGCCTACACAGGTATTATGGGAAGGGGAAGGTGCAATAGTAGTAAAAGATAAGCATACAGAACGATAC
TTGGTAATAACTAATAAGGATGTCAAGTTTGTCCCACAACCAAAAGAAATACAAAAGAAATAA

>MT993915.1 Small ruminant lentivirus isolate USMARC-200335185-2, complete genome
GGGAAACAGCAGGAGGGGGCCACGTGT
GGTGCCGTCTGCACCCCCTATGTTGTAACAGAAGCACCACCTAGAATAGAAGCAAGAGTAGGGACAACTT
GGAAAGAGTTGTTGGTGGATACAGGAGCAGATAGAACTATAGTGAGAAAACATGATAATACAGGGATACC
AAAGGGAAGAATAAAGCTTCAAGGAATAGGAGGAATTATAGAAGGAGAAAAATGGGATCAAGTACAGATA
CAATATAAAGAAAAAATGATAAGAGGAACAATAGTAGTTCTACCGACAAGTCCGGTAGAAGTGTTAGGGA
GAGATAATATGGGAAAATTGGGCATAGGATTAATTATGGCCAATTTAGAAGAAAAGAAAATTCCTATTAC
ACAGGTAAAATTAAAAGAGGGGTGTAAGGGACCCCATATAGCACAGTGGCCTTTGACGCAAGAAAAATTG
GAAGGATTAAAAGAAATAGTAGACAGATTAGAAAAGGAGGGAAAGTTAGGCAGGGCACCACCACATTGGA
CATGTAACACCCCTATTTTTTGCATTAAGAAAAAATCAGGGAAATGGAGAATGTTAATAGACTTCAGAGA
ATTAAATAAACAAACGGAAGACTTAGCGGAGGCACAGTTAGGGCTACCACACCCAGGAGGATTACAGAAG
AAGAAGCATGTAACAATATTAGATATAGGAGATGCGTACTTCACTATTCCATTGTATGAACCATATCGGC
AATATACGTGCTTTACGCTTCTAAGCCCTAATAATCTGGGACCATGTGAAAGGTATTATTGGAAAGTGTT
GCCACAGGGCTGGAAATTGAGTCCGTCGGTATATCAGTTTACAATGCAAAAAATATTAAGAGACTGGATA
AAGGAACATCCTATGATACAATTTGGAATATATATGGATGATATTTATATAGGAAGTGATTTAGAACTAA
GGGAACACAGAGAGATTGTAGAAAACTTGGCTGTATATATTGCACAATATGGATTTATGCTGCCTGAAGA
AAAGAGACAAGAAGGGTATCCAGCTCAATGGCTTGGATTTGAGTTACATCCAGATAAATGGAGATTTCAA
AAACATGCTTTGCCAGAGATCAAGGAAGGATCAATTACTTTAAACAAGTTGCAAAAATTAGTAGGAGATC
TGGTGTGGCGACAATCATTGATAGGAAAAAGTATTCCAAACATATTAAAGTTAATGGAGGGCGACAGGGC
ACTCCAAAGTGAAAGACAAATAGAAAAAATTCATGTACAGGAATGGGAAATATGTAAGAGAAAATTAGTA
GAGATGGAAGGAAATTATTATGATGCAGAAAAGGACATCTATGGACAAATAGATTGGGGAAATAAAGCAA
TTGAATATATAGTGTTTCAGGAAAAAGGGAAACCTTTATGGGTGAATGTAGTGCATAGCATTAAAAATCT
AAGTCAACCACAACAAATTATTAAAGCAGCCCAGAAGTTGACGCAAGAAGTAATCATTAGAACAGGGAAG
ATACCATGGGTACTACTGCCAGGGAAGGAAGAAGATTGGATATTAGAATTACAAATAGGGAATATAACGT
GGATGCCTTCCTTCTGGTCATGCTATAGAGGGTCAGTAAGGTGGAAAAGGAGAAATGTAGTAACAGAAGT
AGTGGAAGGACCAACATATTATACAGATGGAGGTAAGAAAAATGGCCTTGGAAACTTAGGCTACATTGCC
TCGACAGGAGAAAAATATAGATTACATGAAGAAGGCACAAATCAACAGTTAGAATTGAGAGCAATAGAAG
AGGCATGTAAAAGGGGACCAAGCAAGATGAATATAGTAACTGATAGTAGGTACGCATATGAATTTATGCT
AAGGAACTGGGATGAAGAAGTAATAAGAAACCCTATACAGGCCAGAATCATGAAAATAATTCATGGGAAA
GAAAGAGTGGGAGTACATTGGGTACCAGGACATAAAGGCATCCCTCAAAATGAAGAAGTAGATAAATATA
TATCAGAAGTATTTTTAGCAAAACAAGGGGAAGGGATTGTCCAGAAAAGAGCAGAAGACGCTGGATATGA
CTTAATATGTCCGCAGGAAGTAGTCATCCCACCTGGAGAAGTAAGAAAGGTTCCAATAGATTTAAGAATA
AATTTGAAAGAAAATCAATGGGCCATGATAGCAACAAAAAGTAGTTTTGCAAGCAAAGGAGTCTTTATAC
AAGGAGGGATAATAGATTCAGGATATCAAGGAACTATACAAGTAGTAATATACAACAGCAATAAGGTAGA
AGTAATAATACCACAAGGAAGAAAGTTTGCACAATTAATTCTTATGCCTTTGATACATGAAGAATTAGAA
CCATGGGGAACGGCAAGAAGAACTGAGAGAGGGAATGAAGGATTTGGTTCAACAGGAGCCTATTGGATAG
AAAATATCCCTAAAGCAGAAGAGGATCATGATAAATGGCATCAAGATGCAACTTCATTGCACTTAGAATT
TGGGATCCCTAAATCAGCAGCTGAAGATATAGTACAGCAGTGTGAAGTGTGCAAAGAAAATAAGATGCCA
AGTACCCTCAGAGGGGGAAATAAAAGAGGAATAGACCACTGGCAAGTAGATTATACTCATTATGAAGATA
AGATATTATTAGTATGGGTAGAAACAAATTCAGGATTAATTTATGCAGAAAAGGTAAAAGGAGAAACAGG
CCAAGAATTCAGAATACATGTTATGAAATGGTATGCCATGTTTTATCCAAAATCATTGCAGTCCGATAAT
GGACCAGCCTTTGTAGCAGAGCCAACACAGCTCTTGATGAAATATTTAGGGGTAGAACATACTACAGGGG
TTCCGTGGAACCCACAATCTCAAGCCTTGGTTGAAAGAACCCATCAAACGTTGAAACATACCCTAGAGAA
ACTTATCCCCATGTTTGTAGCATTTGAATCTGCTCTTGCGGCCTCGCTCGTAACTCTAAATATAAAAAGA
AAGGGTGGGCTAGGGACAAGCCCTATGGATATATTCATATTTAATAAAGAACAACAAAGAATACAGCAAC
AATTTCAAGTAAATCACGAAAAAATTCGGTTTTGTTATTACAGGATCAGGAAAAGAGGACATCCAGGCGA
GTGGCAGGGGCCTACACAGGTATTATGGGAAGGGGAAGGTGCAATAGTAGTAAAAGATAAGCATACAGAA

CGATATTTGGTAATAACTAATAAGGATGTCAAGTTTGTCCCACAACCAAAAGAAATACAAAAGAGATAA

>MT993913.1 Small ruminant lentivirus isolate USMARC-200106929-2, complete genome
GGGAAACAGCAGGAGGGGGCCACGTGTG
GTGCCGTCCGCGCCCCCTATGTTGTAACAGAAGCACCACCGAGAATAGAAGCAAGAGTAGGAACAACTTG
GAAGGAATTATTAATAGATACAGGAGCAGATAGAACTATAGTGAGAAAACATGATAGTACAGGGATACCA
AGGGGAAGAATAAAGCTTCAAGGGATAGGAGGGATTATAGAAGGAGAAAAATGGGATCAAGTACAAATAC
AATATAAAGGAAAAATAATAAGAGGAACAATAGTAGTGCTACCGACAAGTCCAGTAGAAGTGTTAGGGAG
AGATAATATGGGAGAGTTAGGCATCGGGTTAATTATGGCCAATTTAGAAGAAAAGAAAATCCCTATTACA
CAGGTAAAATTAAAGGAGGGATGTAAGGGACCCCATATAGCACAGTGGCCTTTAACTCAAGAAAAAATTGG
AAGGGTTAAAAGAAATAGTAAATAGATTAGAAAAAGAAGGAAAGCTAGGCAGAGCACCACCGCATTGGAC
ATGTAACACCCCTATTTTTTGTATTAAGAAAAAAATCAGGAAAATGGAGAATGTTAATAGACTTTAGAGAA
TTAAATAAGCAAACGGAAGATTTAGCGGAAGCACAGTTGGGGCTACCACACCCAGGAGGATTACAAAAGA
AGAAACATGTAACAATATTAGATATAGGAGATGCGTACTTCACTATTCCATTGTATGAACCATATCGGCA
ATATACGTGCTTTACGCTTTTAAGCCCTAATAATCTGGGACCATGTGAAAGGTATTATTGGAAAGTGTTG
CCACAAGGCTGGAAATTAAGTCCGTCAGTGTATCAGTTTACAATGCAAAAAATATTAAGAGACTGGATAA
AGGAACATCCTATGATACAATTTGGAATATATATGGATGATATTTATATAGGAAGTGATTTAGACATAGG
GGAGCATAGAAAGATTGTAGAAAACTTGGCTGGATATATCGCACAATATGGATTTATGCTGCCTGAAGAG
AAAAGGCAAGAAGGATATCCAGCTCAATGGCTTGGATTTGAGTTACATCCAGATAAATGGAGATTTCAAA
AACATGCTTTGCCAGATATCCAGGAAGGGTCAATTACTTTAAACAAGTTGCAAAAGTTAGTAGGGGATCT
GGTGTGGAGACAATCATTGATAGGAAAAAGCATTCCAAACATATTAAAATTGATGGAAGGAGATAGGGCA
TTACAAAGTGAAAGGCGAGTAGAACAAATTCATGTACAGGAATGGGAAAATTGTAAGAGAAAATTAAAAG
AAATGGAAGGAAATTATTATAATGAAGAGAGGGACATCTATGGACAAATAGAGTGGGGAGATAAAGCAAT
TGAATATATAGTATTCCAAGAGAAAGGGAAACCTTTGTGGGTAAATGTAGTCCATAATATTAAAAACCTA
AGCCAACCACAGCAAATTATTAAAGCAGCACAAAAACTAACCCAAGAGGTGATAATAAGAACAGGAAAAA
TACCATGGATACTGCTACCAGGAAAGGAAGAGGACTGGATATTAGAATTACAAATAGGGAATATAACATG
GATGCCTTCATTCTGGTCATGCTATAGAGGGTCGGTAAGGTGGAAAAGGAGAAATATAGTAACAGAAGTA
GTAGAAGGACCAACATACTATACTGATGGAGGAAAGAAAAATGGGATTGGGAATCTAGGATATATTGCCT
CGACAGGAGAAAAATATAGAAAACATGAGGAAGGGACAAACCAACAATTAGAATTGAGAGCAATAGAAGA
GGCATGTAAACAGGGGCCAAGCAAAATGAATATAGTAACTGATAGTAGGTACGCATATGAATTTATGCTA
AGAAACTGGGATGAAGAAGTAATAAGAAACCCTATACAGGCCAGAATTATGAAAATAATTCATGGAAAAG
AAAAGGTGGGAGTGCATTGGGTACCAGGACATAAAGGGATTCCTCAGAATGAGGAGATAGATAAATATAT
TTCAGAAATATTTCTAGCAAAAGAAGGAGAAGGGATTCTCCCTAAAAGAGAAGAAGATGCAGGGTATGAT
TTAATCTGTCCTCAGGAGATACATATCCCGGCGGGACAGGTGAGAAAAATTCCTATAGATCTAAGGTTGA
ATTTAAAGAAGAATCAATGGGCCATGATAGCAACAAAAAGTAGTTTTGCAAGTAAGGGAGTCTTTGTACA
AGGAGGGATAATAGATTCAGGATATCAAGGAACAATACAAGTAGTAATATACAACAGCAATAAGGTAGAG
GTAATAATACCACAAGGAAGAAAGTTTGCACAATTGATCCTTATGCCTTTGATACATGAAGAATTGGAGC
CATGGGGAATGGCAAGAAGAACTGAAAGAGGGAATGAAGGGTTTGGTTCAACAGGAGCCTATTGGATAGA
AAATATCCCTAAAGCGGAAGAGGATCATGATAAATGGCATCAAGATGCAACTTCATTGCACTTAGAATTT
GGGATCCCTAAATCAGCGGCTGAAGATATAGTACAACAGTGTGAAGTGTGTCAAGAAAATAAAATGCCAA
GTACCCTCAGAGGAGGAAATAAAAGAGGAATAGATCATTGGCAAGTAGATTACACACATTATGAGGATAA
AATATTATTAGTATGGGTAGAAACAAATTCAGGGTTAATCTATGCAGAAAAGGTAAAAGGAGAAACAGGC
CAAGAATTCAGAATACATGTTATGAAATGGTATGCCATGTTTTCTCCAAAATCATTGCAGTCCGATAATG
GACCGGCCTTTGTAGCGGAGCCAACACAGCTCTTAATGAAATATCTAGGGGTAGAACACAGTACAGGGGT
TCCGTGGAACCCACAATCTCAAGCCTTGGTGGAAAGAACCCATCAAACGTTGAAACATATCCTAGAAAAA
TTTATTCCCATGTTTGTAGCATTTGAATCTGCTCTTGCGGCCTCGCTCATAAATCTAAATATAAAAAGAA
AGGGTGGGCTAGGGACAAGCCCTATGGATATATTCATATTTAATAAAGAACAACAAAGAATACAGCAACA
ATTTCAAGTAAATCAAGAAAAAAATTCGGTTTTGTTATTACAGGATCAGGAAAAGAGGACATCCAGGCGAG
TGGCAAGGGCCTACACAGGTATTGTGGGAAGGGGAAGGTGCAATAGTGGTAAAAGATAAACATACAGAAC
GATACTTGGTAGTAACTAATAAAGATGTCAGGTTTGTCCCACCACCAAAAGAAATACAAAAGAAATAA

>MT993910.1 Small ruminant lentivirus isolate USMARC-200312088-r, complete genome
GGGAAACAGCAGGAGGGGGCCACGTGTGGT
GCCGTCCGCGCCCCCTATGTTGTAACAGATGCACCACCGAGAATAGAAGCAAGAGTAGGGACAACTTGGA
AAGAATTATTAGTAGATACGGGAGCAGATAGAACTATAGTGAGAAAACATGATAATTCAGGGATACCAAG
GGGAAGAATAAAACTACAAGGAATAGGAGGGATTATAGAAGGGGAAAAATGGGATCAAGTACAGATACAA
TATAAAGAGAAAATAATAAGAGGAACAATAGTAGTACTACCAACAAGTCCGGTAGAAGTGTTAGGGAGAG
ATAATATGGGAGAATTGGGCATAGGATTAATTATGGCCAATTTAGAAGAAAAGAAAATTCCCATAACACA
GGTAAAATTAAAAGAGGGATGTAAGGGACCCCATATAGCACAGTGGCCTCTGACTCAAGAAAAGTTGGAA

GGGCTGAAAGAAATTGTAGATAGATTAGAAAAAGAAGGAAAACTAGGAAGGGCGCCACCTCATTGGACAT
GCAATACTCCAATATTTTGCATTAAGAAAAAATCAGGGAAATGGAGAATGTTAATAGATTTTAGGGAATT
AAACAAGCAAACAGAGGATTTAGCAGAGGCTCAGCTAGGGTTACCGCATCCGGGGGGATTGAAGAAGAAG
AAAAATGTAACAGTCCTCGATATTGGGGACGCGTATTTTACAATCCCATTATATGAACCTTATAGACAGT
ATACATGCTTTACTCTGCTAAGTCCCAATAATTTGGGACCATGTGTTAGATACTATTGGAAAGTATTGCC
TCAAGGATGGAAGTTAAGCCCGTCCGTATATCAGTTTACAATGCAGAAAATATTAAGAGATTGGATAGAG
GCACATCCTCTGATACAGTTTGGGATATATATGGATGATATTTATATAGGAAGTGATCTAGAGATAAAAG
AGCATAGAGAGATAGTGGATGAATTAGCAAATTATATAGCACAATTTGGCTTCATGTTGCCTGAAGATAA
GAGACAGGAAGGGTATCCAGCCAAGTGGCTAGGATTTGAATTACACCCTGACAAATGGAAGTTTCAAAAA
CATACATTAGCAGAGCTGAAAGAAGGACCAATCACATTAAATAAATTACAAAAATTAGTAGGGGACTTAG
TATGGCGGCAATCGTTGATAGGAAAAAGTATTCCAAGTATATTAAAGTTAATGGAGGGAGATAGGGCACT
CCAAAGTGAAAGACAAATAGAAAAAATTCATGTACAGGAATGGGAAACATGTAAGAAAAAATTAGTAGAA
ACAGAAGGAAATTATTATGATGAAGAAAAGGATATTTATGGACAAGTAGATTGGGGAAATAAAGCAATTG
AATATATGGTATTTCAGGAAAAGGGGAAACCTTTGTGGGTAAATGTAGTCCATAACATAAAGAATTTGAG
TCAACCACAGCAAATTATTAAAGCAGCCCAGAAGCTGACACAAGAAGTAATCATTAGAACAGGAAAGATA
CCATGGATACTACTGCCAGGGAAGGAAGAAGATTGGATTTTAGAAATGCAAATAGGGAATATAACGTGGA
TGCCTTCATTTTGGTCATGTTATAGAGGATCAGTGAGATGGAAAAGGAGAAATATAGTAGCAGAAGTAGT
AGAAGGGCCAACATATTATACAGATGGAGGAAAGAAAAATGGGGTTGGAAATTTAGGCTACATTGCCTCA
ACAGGGGAAAAATACAGGATACATGAGGAAGGGACCAATCAGCAGTTAGAACTAAGGGCAATAGAAGAGG
CATGTAAACAGGGACCAAGCAAGATGAATATAGTAACTGACAGCAGATATGCATATGAATTTATGCTAAG
GAATTGGGATGAAGAAGTAATAAGAAACCCTATACAGGCCAGAATTATGAAAATAATTCATGGAAAAGAA
AAAGTGGGAGTACATTGGGTTCCAGGACATAAAGGAATTCCTCAGAATGAGGAAATAGATAAATATATTT
CAGAAATATTTCTAGCAAAAGAAGGAGGAGGGATTCTCCCTAAAAGAGAAGAAGATGCAGGGTATGATTT
AATTTGTCCTCAGGAGGTACATATTCCGGCGGGACAAGTAAGAAAAATCCCCGTAGACTTAAGATTAAAT
TTACAAGAAAAGCAATGGGCCATGATAGGGACAAAGAGTAGCTTTGCCAGCAAGGGGGTATTTGTTCAAG
GAGGAATAATAGATTCAGGGTATCAGGGAACAATACAAGTAGTAATATACAATAGTAATGATGTGGAAGT
GGTAATTCCCCAGGGAAGGAAGTTTGCACAATTAATTCTTATGCCTTTGATACATGAAGAATTGGAACCA
TGGGGAACGGCAAGAAGAACTGAGAGAGGGAATGAAGGATTTGGGTCAACAGGAGCCTATTGGATAGAAA
ATATCCCTAGAGCAGAAGAGGATCATGATAAGTGGCATCAAGATGCAAATTCATTGCACTTAGAATTTGG
AATTCCTAAATCAGCTGCTGAAGATATAGTGCAACAATGTGAAGTGTGTCAAGAAAATAAAATGCCAAGT
ACCCTCAGAGGAGGAAATAAAAGAGGAATAGATCATTGGCAAGTGGATTATACACATTATGAGGATAAAA
TATTGTTAGTATGGGTAGAAACAAATTCAGGATTAATCTATGCAGAAAAGGTGAAAGGAGAAACAGGTCA
AGAATTCAGAATACATGCTATGAAATGGTATGCCATGTTTAATCCAAAATCAGTGCAGTCCGATAATGGA
CCGGCTTTTGTAGCGGAGCCAACACAGCTCTTAATGAAATATCTAGGAGTAGAACACACTACAGGGGTTC
CGTGGAACCCACAATCTCAAGCCTTAGTAGAAAGAACTCATCAAACGTTAAAACATACCCTGGAAAAATT
TATCCCCATGTTTGCAGCATTTGAATCTGCTCTTGCGGCAACCCTGATAGCACTAAATATAAAAAGAAAG
GGTGGGCTAGGGACAAGCCCTATGGATATATATATATTTAATAAGGAACAACAAAGAATACAGCAACAGT
CTCAATTAAATCAATCAAAAATTCGATTTTGTTATTACAGGATCAGGAAGAAAGGACATCCAGGCGAGTG
GCAAGGGCCAACTCAGGTATTGTGGGAAGGGGAAGGTGCAATAGTAGTAAGAGATAAATCCACAGAAAGA
TATTTCGTAGTAACAAACAAGGATGTCAAATTCATTCCACCGCCAAAAGAAATACAAAAGGAATAA

>MT993917.1 Small ruminant lentivirus isolate USMARC-199916128-2, complete genome
GGGAAACAACAGGAGGGGGCCACGTGTGGTGCCGTCCGCGCCCCCTATGTTG
TAACAGAAGCACCACCTAGAATAGAAGCAAGAGTAGGGACAACTTGGAAAGAATTGTTGGTGGATACAGG
AGCAGATAGAACAATAATAAGAAAACATGATAGTACAGGGATACCAAGGGGAAGAATAAAATTACAAGGA
ATAGGAGGGATTATAGAAGGAGAAAAATGGGAGCAGGTACAAATACAATATAAAGGAAAAATAATAAGGG
GAACTATAGTAGTCCTACCGACAAGTCCAGTAGAAGTGTTAGGTAGAGATAATATGGGAAAATTGGGCAT
AGGATTAATTATGGCCAATTTAGAAGAAAAGAAAATTCCTATTACACAGGTAAAATTAAAGGAGGGGTGT
AAGGGACCCCATATAGCACAGTGGCCTTTGACGCAAGAGAAATTGGAAGGATTAAAAGAAATAGTAAACA
GATTAGAAAAAGAAGGAAAGTTAGGCAGAGCACCACCTCATTGGACATGTAACACCCCTATTTTTTGCAT
TAAGAAAAAATCAGGGAAATGGAGAATGTTAATAGATTTTAGAGAATTAAATAAGCAAACGGAAGATTTA
GCAGAAGCACAGTTAGGGCTACCACATCCAGGAGGATTACAGAAAAAGAAGCATGTAACAATATTAGATA
TAGGAGATGCATACTTCACTATTCCATTGTATGAACCATATCGGCAATATACATGCTTTACGCTTCTAAG
CCCTAATAATCTGGGACCATGTGAAAGGTATTATTGGAAAGTGTTGCCACAAGGCTGGAAATTGAGTCCG
TCGGTATATCAGTTTACAATGCAAAAAATATTAAGAGACTGGATAAGGGAACACCCTATGATACAATTTG
GAATATATATGGATGATATTTATATAGGAAGTGATTTAGAACTAAGGGAACACAGAGAGATTGTAGAAAA
CTTGGCTGTATATATTGCACAATATGGATTTATGCTGCCGGAAGAAAAGAGGCAAGAAGGGTATCCAGCT
CAATGGCTTGGATTTGAGTTACATCCAGACAAATGGAGATTTCAAAAACATGCTTTGCCAGACATCAAGG
AAGGGTCAATTACTTTAAATAAGTTGCAAAAATTGGTAGGGGAGCTGGTATGGAGGCAATCCTTGATAGG
AAAAGTATTCCAAACATATTAAAATTAATGGAAGGAGATAGGGCGTTACAGAGTGAAAGGCGAGTAGAA

CAAATTCATGTACTTGAATGGGAAAATTGTAAGAAAAAATTAAGAGAAATGGAAGGAAATTATTATAATA
AAGAGAGGGATATTTATGGACAAATAGATTGGGGAAATAAAGCAATTGAATATATAGTGTTTCAGGAAAA
AGGGAAACCTTTATGGGTGAATGTAGTGCATAGCATTAAAAATCTAAGTCAGCCACAACAAATTATTAAA
GCAGCCCAGAAGATGACGCAAGAAGTGATCATTAGAACAGGGAAGATACCATGGATACTACTACCAGGGA
AGGAAGAAGATTGGATATTAGAATTACAAATAGGGAATATAACGTGGATGCCTTCCTTCTGGTCATGCTA
TAGAGGGTCAGTAAGGTGGAAAAGGAGAAATATAGTAACAGAAGTAGTAGAGGGACCAACGTACTATACA
GATGGAGGTAAGAAAAATGGGATTGGAAATTTAGGATATATTGCCTCGACAGGAGAGAAATATAGACTAC
ATGAGGAAGGGACAAATCAACAGCTAGAATTGAGAGCAATAGAAGAGGCATGTAGAAGGGGACCAAGTAA
GATGAATATAGTAACTGATAGTAGGTACGCATATGAATTTATGATAAGGAACTGGGATGAAGAAGTAATA
AGAAACCCTATACAGGCCAGAATCATGAAAATAATTCATGGGAAAGAAAGAGTGGGAGTGCATTGGGTAC
CAGGACATAGAGGGATCCCTCAGAATGAGGAAATAGATAAATATATATCAGAAATATTTTTAGCAAGACA
AGGGGAAGGGATTGTCCAGAAAAGAGCAGAGGACGCTGGATATGACTTAATATGTCCGCAAGAAGTAGTC
ATCCCACCTGGAGAAGTAAGAAAGGTTCCAATAGATTTAAGAATAAATTTGAAAGAAAATCAATGGGCCA
TGATAGCAACAAAAAGTAGTTTTGCAAGTAAGGGAGTCTTTGTACAAGGAGGGATAATAGATTCAGGATA
TCAAGGAACTATACAAGTAGTAATATACAACAGCAATAAGGTAGAGGTAATAATACCACAAGGAAGAAAG
TTTGCACAATTAATTCTTATGCCTTTGGTACATGAAGAATTAGAACCATGGGGAACGGCAAGAAGAACTG
AGAGAGGGAATGAAGGATTTGGATCAACAGGAGCCTATTGGATAGAAAATATCCCTAAGGCGGAAGAGGA
TCATGGTAAATGGCATCAAGATGCAACTTCATTGCACTTAGAATTTGGGATCCCTAAATCAGCGGCTGAA
GATATAGTACAACAGTGTGAAGTGTGCCAAGAAAATAAAATGCCAAGTACCCTCAGAGGAGGAAATAAAA
GAGGAATAGACCATTGGCAAGTAGATTATACTCATTATGAAGATAAAATATTATTAGTATGGGTAGAAAC
CAATTCAGGATTAATTTATGCAGAAAAGGTAAAAGGAGAAACAGGTCAAGAATTCAGAATACATGTTATG
AAATGGTATGCCATGTTTTATCCAAAATCATTACAGTCCGATAATGGACCTGCTTTTGTAGCAGAGCCAA
CACAGCTCTTGATGAAATATCTGGGGGTGGAACATACAACAGGGGTTCCGTGGAACCCGCAATCTCAAGC
CTTGGTTGAAAGAACCCATCAAACGTTGAAACATACCCTAGAGAAACTTATCCCCATGTTTGTAGCATTT
GAATCTGCTCTTGCGGCCTCGCTCATAACTCTAAATATAAAAAGAAAGGGTGGGCTAGGGACAAGCCCTA
TGGATATATTCATATTTAATAAAGAGCAACAAAGAATACAGCAACAATTTCAAGTAAATCAAGAAAAAAT
TCGGTTTTGTTATTACAGAATCAGGAAAAGAGGACATCCAAGCGAGTGGAAAGGACCAACACAGGTATTG
TGGGAAGGAGAAGGGGCAATAGTAGTAAAAGATAAGCAGACAGAACGATACTTGGTAGTAACTAATAAGG
ATGTCAGGTTTGTCCCATCACCAAAAGAAATACAAAAGAAATAA

>KY358788.1 Small ruminant lentivirus isolate USMARC-199906011-2, complete genome
GGGAAACAGCAGGAGGGGGCCACGTGTGGTGCCG
TCCGCGCCCCCTATGTTGTAACAGAAGCACCACCGAGAATAGAAGCAAGAGTAGGAACAACTTGGAAGGA
ATTATTAATAGATACAGGAGCAGATAGAACTATAGTGAGAAAACATGATAGTACAGGGATACCAAGGGGA
AGAATAAAGCTTCAAGGGATAGGAGGGATTATAGAAGGAGAAAAATGGGATCAAGTACAAATACAATATA
AAGGAAAAATAATAAGAGGAACAATAGTGGTATTACCGACAAGTCCAGTAGAAGTGTTAGGGAGAGATAA
TATGGGAGAGTTAGGCATCGGGTTAATTATGGCCAATTTAGAAGAAAAGAAAATCCCTATTACACAGGTA
AAATTAAAGGAGGGATGTAAGGGACCCCATATAGCACAGTGGCCTTTAACTCAAGAAAAATTGGAAGGGT
TAAAAGAAATAGTAAATAGATTAGAAAAAGAAGGAAAGCTAGGCAGAGCACCACCGCATTGGACATGTAA
CACCCCTATTTTTTGTATTAAGAAAAAATCAGGAAAATGGAGAATGTTAATAGACTTTAGAGAATTAAAT
AAGCAAACGGAAGATCTAGCGGAAGCACAGTTGGGGCTACCACACCCAGGGGGATTACAAAAGAAGAAAC
ATGTAACAATATTAGATATAGGAGATGCGTACTTTACTATTCCATTGTATGAACCATATCGGCAATATAC
GTGCTTTACGCTTTTAAGCCCTAATAATCTGGGACCATGTGAAAGGTATTATTGGAAAGTGTTGCCACAA
GGCTGGAAATTAAGTCCGTCAGTGTATCAGTTTACAATGCAAAAAATATTAAAAGACTGGATAAAGGAAC
ATCCTATGATACAGTTTGGGATATATATGGATGATATCTATATAGGAAGTGATCTAGAGATAAAAGAGCA
TAGAGGAATAGTGGAAGAGTTAGCAAGTTATATAGCACAATTTGGCTTCATGTTGCCTGAAGATAAAAGA
CAGGAAGGGTATCCAGCCAAATGGCTAGGATTCGAGCTACACCCTGATAAATGGAGGTTTCAAAAGCATA
CCTTAGCAGAGTTAAAAGAAGGACCAATTACATTAAATAAGTTACAAAAATTAGTAGGGGAGCTGGTATG
GCGGCAATCATTGATAGGAAAAAGTATTCCAAACATATTAAAATTAATGGAAGGGGATAGGGCATTACAG
AGTGAAAGACGAGTAGAACAAATTCATGTACGTGAATGGGAAAATTGTAAGAAAAAATTAGAAGAAATGG
AAGGAAATTATTATAACGAAGAGAGGGATATTTATGGACAAATAGATTGGGGAAATAAAGCAATTGAATA
TATAGTATTCCAAGAGAAAGGGAAACCTTTGTGGGTAAATGTAGTCCATAATATTAAAAACCTAAGCCAA
CCACAGCAAATTATTAAAGCAGCACAAAAGCTAACCCAAGAGGTGATAATAAGAACAGGAAAAATACCAT
GGATCCTGTTACCAGGAAAGGAAGAGGACTGGATATTAGAATTACAAATAGGGAATATAACATGGATGCC
TTCATTCTGGTCATGCTATAGAGGGTCAGTAAGGTGGAAAAGGAGAAATATAGTAACAGAAGTAGTAGAA
GGACCAACATACTATACTGATGGAGGAAAGAAAAATGGGATTGGGAATCTAGGATATATTGCCTCGACAG
GAGAAAAATATAGAAAACATGAAGAAGGAACAAATCAACAATTAGAACTACGAGCAATAGAAGAGGCATG
TAAACAGGGGCCAAGCAAGATGAATATAGTAACTGATAGTAGGTACGCATATGAATTTATGCTAAGAAAT
TGGGATGAAGAAGTAATAAGAAACCCTATACAGGCCAGAATTATGAGAATAATTCATGGGAAAGAAAGAG
TGGGAGTGCATTGGGTACCAGGACATAAAGGGATCCCTCAGAATGAGGAAATAGATAAATATATTTCAGA

AGTATTTTTAGCAAAAGAAGGAGAAGGGATTCTCCAGAAAAGAGCAGAAGATGCGGGATATGATTTAATC
TGTCCTCAGGAGATACATATCCCGGCGGGACAGGTGAGAAAAATTCCTATAGATCTAAGGTTGAATTTAA
AGAAGAATCAATGGGCCATGATAGCAACAAAAAGTAGTTTTGCAAGTAAGGGAGTCTTTGTACAAGGAGG
GATAATAGATTCAGGATATCAAGGAACAATACAAGTAGTAATATACAACAGCAATAAGGTAGAGGTAATA
ATACCACAAGGAAGAAAGTTTGCACAATTGATCCTTATGCCTTTAATACATGAAGAATTGGAGCCATGGG
GAATGGCAAGAAGAACTGAAAGAGGAAATGAAGGGTTTGGTTCAACAGGAGCCTATTGGATAGAAAATAT
CCCTAAAGCGGAAGAGGATCATGATAAATGGCATCAAGATGCAACTTCATTGCACTTAGAATTTGGGATC
CCTAAATCAGCGGCTGAAGATATAGTACAACAGTGTGAAGTGTGTCAAGAAAATAAAATGCCAAGTACCC
TCAGAGGAGGAAATAAAAGAGGAATAGATCATTGGCAAGTGGACTACACACATTATGAGGATAAAATATT
ATTAGTATGGGTAGAAACAAATTCAGGGTTAATCTATGCAGAAAAGGTAAAAGGAGAAACAGGCCAAGAA
TTCAGAATACATGTTATGAAATGGTATGCCATGTTTTCTCCAAAATCATTGCAGTCCGATAATGGACCGG
CCTTTGTAGCGGAGCCAACACAGCTCTTAATGAAATATCTGGGAGTAGAACATACTACAGGGGTTCCGTG
GAATCCACAATCACAAGCTTTAGTGGAAAGAACCCATCAAACGCTGAAACATCTCCTAGAAAAATTTATT
CCCATGTTTGTAGCATTTGAATCTGCTCTTGCGGCCTCGCTCATAAATCTAAATATAAAAAGAAAGGGTG
GGCTAGGGACAAGCCCTATGGATATATTCATATTTAATAAAGAACAACAAAGAATACAGCAACAATTTCA
AGTAAATCAAGAAAAAAATTCGGTTTTGTTATTACAGGATCAGGAAAAGAGGACATCCAGGCGAGTGGCAA
GGGCCAACACAGGTATTGTGGGAAGGGGAAGGAGCAATAGTGGTAAAAGATAAGCATACAGAACGATACT
TGGTAGTAACTAATAAGGATGTCAAGTTTGTCCCACCACCAAAAGAAATACAAAAGAAATAA

>MT993911.1 Small ruminant lentivirus isolate USMARC-199906011-2,
complete genome
GGGAAACAGCAGGAGGGGGCCACGTGTGGTG
CCGTCCGCGCCCCCTATGTTGTAACAGAAGCACCACCGAGAATAGAAGCAAGAGTAGGAACAACTTGGAA
GGAATTATTAATAGATACAGGAGCAGATAGAACTATAGTGAGAAAACATGATAGTACAGGGATACCAAGG
GGAAGAATAAAGCTTCAAGGGATAGGAGGGATTATAGAAGGAGAAAAATGGGATCAAGTACAAATACAAT
ATAAAGGAAAAATAATAAGAGGAACAATAGTGGTATTACCGACAAGTCCAGTAGAAGTGTTAGGGAGAGA
TAATATGGGAGAGTTAGGCATCGGGTTAATTATGGCCAATTTAGAAGAAAAGAAAATCCCTATTACACAG
GTAAAATTAAAGGAGGGATGTAAGGGACCCCATATAGCACAGTGGCCTTTAACTCAAGAAAAATTGGAAG
GGTTAAAAGAAATAGTAAATAGATTAGAAAAAGAAGGAAAGCTAGGCAGAGCACCACCGCATTGGACATG
TAACACCCCTATTTTTTGTATTAAGAAAAAATCAGGAAAATGGAGAATGTTAATAGACTTTAGAGAATTA
AATAAGCAAACGGAAGATCTAGCGGAAGCACAGTTGGGGCTACCACACCCAGGGGGATTACAAAAGAAGA
AACATGTAACAATATTAGATATAGGAGATGCGTACTTTACTATTCCATTGTATGAACCATATCGGCAATA
TACGTGCTTTACGCTTTTAAGCCCTAATAATCTGGGACCATGTGAAAGGTATTATTGGAAAGTGTTGCCA
CAAGGCTGGAAATTAAGTCCGTCAGTGTATCAGTTTACAATGCAAAAAATATTAAAAGACTGGATAAAGG
AACATCCTATGATACAGTTTGGGATATATATGGATGATATCTATATAGGAAGTGATCTAGAGATAAAAGA
GCATAGAGGAATAGTGGAAGAGTTAGCAAGTTATATAGCACAATTTGGCTTCATGTTGCCTGAAGATAAA
AGACAGGAAGGGTATCCAGCCAAATGGCTAGGATTCGAGCTACACCCTGATAAATGGAGGTTTCAAAAGC
ATACCTTAGCAGAGTTAAAAGAAGGACCAATTACATTAAATAAGTTACAAAAATTAGTAGGGGAGCTGGT
ATGGCGGCAATCATTGATAGGAAAAAGTATTCCAAACATATTAAAATTAATGGAAGGGGATAGGGCATTA
CAGAGTGAAAGACGAGTAGAACAAATTCATGTACGTGAATGGGAAAATTGTAAGAAAAAATTAGAAGAAA
TGGAAGGAAATTATTATAACGAAGAGAGGGATATTTATGGACAAATAGATTGGGGAAATAAAGCAATTGA
ATATATAGTATTCCAAGAAAAAGGAAACCTTTGTGGGTAAATGTAGTCCATAATATTAAAAACCTAAGC
CAACCACAGCAAATTATTAAAGCAGCACAAAAACTAACCCAAGAGGTGATAATAAGAACAGGAAAAATAC
CATGGATACTGTTACCAGGAAAGGAAGAGGACTGGATATTAGAATTACAAATAGGGAATATAACATGGAT
GCCTTCATTCTGGTCATGCTATAGAGGGTCAGTAAGGTGGAAAAGGAGAAATATAGTAACAGAAGTAGTA
GAAGGACCAACATACTATACTGATGGAGGAAAGAAAAATGGGATTGGGAATCTAGGATATATTGCCTCGA
CAGGAGAAAAATATAGAAAACATGAAGAAGGAACAAATCAACAATTAGAACTACGAGCAATAGAAGAGGC
ATGTAAACAGGGGCCAAGCAAGATGAATATAGTAACTGATAGTAGGTACGCATATGAATTTATGCTAAGA
AATTGGGATGAAGAAGTAATAAGAAACCCTATACAGGCCAGAATTATGAGAATAATTCATGGGAAAGAAA
GAGTGGGAGTGCATTGGGTACCAGGACATAAAGGGATCCCTCAGAATGAGGAAATAGATAAATATATATC
AGAAGTATTTTTAGCAAGAGAAGGAGAAGGGATTCTCCAGAAAAGAGCAGAAGATGCGGGATATGATTTA
ATCTGTCCTCAGGAGATACATATCCCGGCGGGACAGGTGAGAAAAATTCCTATAGATCTAAGGTTGAATT
TAAAGAAGAATCAATGGGCCATGATAGCAACAAAAAGTAGTTTTGCAAGTAAGGGAGTCTTTGTACAAGG
AGGGATAATAGATTCAGGATATCAAGGAACAATACAAGTAGTAATATACAACAGCAATAAGGTAGAGGTA
ATAATACCACAAGGAAGAAAGTTTGCACAATTGATCCTTATGCCTTTAATACATGAAGAATTGGAGCCAT
GGGGAATGGCAAGAAGAACTGAAAGAGGAAATGAAGGGTTTGGTTCAACAGGAGCCTATTGGATAGAAAA
TATCCCTAAAGCGGAAGAGGATCATGATAAATGGCATCAAGATGCAACTTCATTGCACTTAGAATTTGGG
ATCCCTAAATCAGCGGCTGAAGATATAGTACAACAGTGTGAAGTGTGTCAAGAAAATAAAATGCCAAGTA
CCCTCAGAGGAGGAAATAAAAGAGGAATAGATCATTGGCAAGTGGACTACACACATTATGAGGATAAAAT
ATTATTAGTATGGGTAGAAACAAATTCAGGGTTAATCTATGCAGAAAAGGTAAAAGGAGAAACAGGCCAA
GAATTCAGAATACATGTTATGAAATGGTATGCCATGTTTTCTCCAAAATCATTGCAGTCCGATAATGGAC

CGGCCTTTGTAGCGGAGCCAACACAGCTCTTAATGAAATATCTGGGAGTACAACATACTACAGGGGTTCC
GTGGAATCCACAATCACAAGCTTTAGTGGAAAGAACCCATCAAACGCTGAAACATCTCCTAGAAAAATTT
ATTCCCATGTTTGTAGCATTTGAATCTGCTCTTGCGGCCTCGCTCATAAATCTAAATATAAAAAGAAAGG
GTGGGCTAGGGACAAGCCCTATGGATATATTCATATTTAATAAAGAACAACAAAGAATACAGCAACAATT
TCAAGTAAATCAAGAAAAAATTCGGTTTTGTTATTACAGGATCAGGAAAAGAGGACATCCAGGCGAGTGG
CAAGGGCCAACACAGGTATTGTGGGAAGGGGAAGGAGCAATAGTGGTAAAAGATAAGCATACAGAACGAT
ACTTGGTAGTAACTAATAAGGATGTCAAGTTTGTCCCACCACCAAAAGAAATACAAAAGAAATAA

>EU528031.1 Synthetic construct polymerase (pol) gene, complete cds
ATGCATACAGCTGCAGGGAAACAACAGGAGGGGGCCACGTGTGGTGCCGTCCGCGCCTCCTATGTTGTAA
CAGAAGCACCACCAAAGGCAGAAATAAAGGTAGGGACAACATGGAGAATGTTATTAGTAGACACCGGAGC
AGATAGGACAATAGTAAGATATCATGATAATTCGGGAATACCAAAAGGAAGAATAAAATTGCAGGGTATA
GGGGGAATTATAGAAGGAGAAAAATGGGACAAAGTGGCGTTACAGTATAAAGAAAAAAGAATCTTGGGTA
CCATAGTAGTACTGCCTAGCAGTCCAGTGGAGGTATTAGGAAGGGATAATATGGGAGAATTAGGAATAGG
ACTAATTATGGCAAATCTGGAAGAAAGGAAAATCCCTATTACCAAGGTAAGCCTAAAAGAAGGCTGCAAG
GGACCTCATATAGCGCAGTGGCCTTTGACTCAAGAAAAATTAGAAGGATTGAAAGAAATAGTGGAAAGAT
TAGAAAAAGAAGGGAAATTAGGTAGAGCACCTCCACATTGGACATGTAGCACTCCTATATTTTGCATCAA
AAAGAAATCAGGAAAATGGAGAATGTTAATAGACTTCAGGGAATTGAATAAACAAACAGAAGATTTGGCA
GAGGCACAGTTGGGATTACCACATCCGGGAGGATTGCAGAAAAAGAAACATGTAACAGTATTGGATATAG
GAGATGCATATTTTACAATACCATTATATGAACCTTATAGACAGTATACATGTTTTACCATGCTGAGTCC
CAATAATTTGGGACCTTGTGTAAGGTATTATTGGAAAGTGTTGCCACAAGGATGGAAATTGAGTCCCTCA
GTGTATCAATTTACAATGCAGGAGATATTGAAAGATTGGATAAGGGAACACCCTATGGTGCAATTTGGGA
TATATATGGATGATATATATATAGGCAGTGATTTAGAAATGGGGGAACACAGAAGAATAGTAGAAGAACT
TGCCAGTTATATTGCCCAATATGGGTTTATGCTGCCGGAAGAGAAGAGGCAAGAAGGGTATCCAGCAAAT
TGGCTTGGATTTGAACTACATCCAGAGAGATGGAAGTTTCAAAAACATAAGCTTCCAGATATGGAAGAAG
GACCAATAACGTTAAATAAATTGCAGAAATTAGTAGGAGAGTTAGTTTGGAGGCAATCATTGATAGGGAA
AAGTATACCAAATATACTGAAATTGATGGAAGGAGATAGAGCGTTACAAAGTGTAAGGAATGTAGAGAAA
ATACATATAGAAGAATGGGAAGGATGTAAAAGAAAACTAGAAGAAATGGAAGGGAATTATTATAATGCAG
AAAGGGACGTTTATGGACAAGTAGACTGGGGAAACAAAGCAATAGAATATATAGTGTTCCAAGAAAAAGG
GAAACCATTATGGGTGAATGTAGTACATAGCATTAAGAATTTGAGCCAAGCACAGCAGATCATTAAAGCG
GCACAAAAGCTTACACAAGAAGTAATAATAAGAACAGGAAAAATACCATGGATACTACTGCCAGGAAAAG
AGGAGGACTGGATCTTGGAACTGCAGGTGGGAAATATCACGTGGATGCCATCATTTTGGTCATGTTATAG
GGGATCAGTAAGATGGAAAAAGAGAAATGTAGTAACAGAAGTAGTAGAGGGGCCAACATATTATACAGAT
GGAGGGAAGAAAAATGGAGAGGGAAGCTTAGGATATATTGCTTCCACCGGGGAAAAATGTAGAATGCATG
AGAAAGGGACAAATCAACAACTAGAATTAAGGGCAATTGAGGAAGCATGTAAACAGGGACCAAGCAAAAT
GAATATAGTAACAGATAGTAGATATGCATTTGAGTTCATGATAAGGAACTGGGATGAAGAAGTTATAAAG
AATCCAATACAGGCACGAATCATGAAGTTGATACATAGTAAGGAGAAGGTAGGGATACATTGGGTGCCAG
GCCATAAAGGGATTCCTCAAAATGAAGAAATAGATAAATATATTTCAGAAGTATTTTTAGCAAAAGAAGG
AAATGGGATAGTAAAGAAAAGAGCAGAGGATGCTGGGTATGATTTGATATGCCCACAAGAGGTAAGTATC
CCAGCAGGACAAGTAAAGAAGATTCCAATTGATTTAAGAATAAATTTAAGAAAAAATCAATGGGCTATGA
TAGGGACAAAAAGCAGTTTTGCAAGTAAGGGAGTATTTGTACAAGGAGGAATAGTAGATTCAGGATATCA
GGGAATCATACAAGTAGTAATTTATAACAGCAATGACGAAGAGGTCATTATACCCCAGGGGAGGAAATTT
GCACAGTTAATTCTCATGCCGCTGATACATGAGGAATTAGAGCCATGGGGGGAAACAAGAAAAACAGAAA
GAGGAAATCAGGGATTTGGATCAACAGGAGCATATTGGATTGAAAATATTCCCCTGGCAGAGGAAGAGCA
TAGTAAATGGCATCAAGATGCTCAATCATTGCATCTAGAATTTGACATACCAAGAACAGCGGCTGAAGAT
ATAGTGCAACAATGTGAAATATGTCAAGAAAATAAAATGCCTAGTACAATGAGAGGAAGTAACAAGAGGG
GAATAGATCATTGGCAGGTGGATTACACTCATTTTGAAGATAAGATATTACTAGTATGGGTAGAAACAAA
TTCGGGATTAATTTATGCAGAAAGGGTGAAAGGGGAGACAGGACAAGAATTTAGAGTAACAACTATGAAG
TGGTATGCTCTGTTTAGGCCAAAATCACTGCAATCTGATAATGGACCAGCATTTATAGCAGAAGCAACAC
AATTGTTAATGACATATTTAGGTGTAGAACACACAACAGGGATACCTTGGAATCCACAGTCTCAAGCGCT
AGTGGAAAGGGCTCATCAGACTTTAAAGAAAACAATTGAAAAACTTGTTCCTATGTTCTCTGCATTTGAA
TCAGCTGTCGCAGCTGCATTAATAGCTCTAAATATAAAAAGAAAGGGTGGGCTAGGGACAAGCCCTATGG
ATATATTCATATTTAATAAGGAACAGCAAAGAATACAACAACAGTATAAATTAAATCAAGAAAAAATTCG
ATTTTGTTATTACAGAATCAGAAAAAGAGGACACCCAGGCGACTGGCTGGGACCGTCTCAGGTACTCTGG
GAAGGGGAAGGAGCAATAGTCGTAAAAGATAGAACTCTAGATAAGTATTTAGTAATAGCTAACAAAGATG
TTAAATTCATACCGCAACCAAAAGAAATACAAAAAGAGCAAAAATAG

>MT993918.1 Small ruminant lentivirus isolate USMARC-199916193-r, complete genome
GGGAAACAGCAGGAGGGGGCCACGTGT
GGTGCCGTCCGCGCCCCCTATGTTGTAACAGAAGCACCACCGAGAATAGAAGCCAGGGTAGGGAAAACTT

GGAAGGAATTATTAGTAGATACAGGAGCAGATAGAACTATAGTGAGAAAACATGATAATACAGGGATACC
AAAAGGAAGAATAAAGCTTCAAGGAATAGGAGGAATTATAGAAGGAGAAAAATGGGATCAAGTACAGATA
CAATATAAAGAAAAAATAATAAGGGGAACAATAGTAGTCCTACCGACAAGCCCAGTAGAAGTGTTAGGTA
GAGATAATATGGGAAAATTGGGCATAGGGTTAATTATGGCCAATTTAGAAGAAAAGAAAATTCCTATTAC
ACAGGTAAAATTAAAAGAGGGGTGTAAGGGACCTCATATAGCTCAGTGGCCTTTAACTCAAGAAAAATTG
GAAGGGTTGAAAGAAATAGTAAACAGATTAGAAAAAGAAGGAAAGTTAGGCAGAGCACCACCACATTGGA
CATGTAACACCCCTATTTTTTGCATTAAGAAAAAATCAGGGAAATGGAGAATGTTAATAGATTTTAGAGA
ACTAAATAAGCAAACGGAAGACTTAGCGGAAGCACAATTAGGGCTACCACATCCAGGAGGATTACAGAAA
AAGAAGCATGTAACAATATTAGATATAGGAGATGCATACTTCACTATTCCATTGTATGAACCATATCGGC
AATATACATGCTTTACGCTTCTAAGCCCTAATAATCTGGGACCATGTGAAAGGTATTATTGGAAAGTGTT
GCCACAAGGCTGGAAATTGAGTCCGTCGGTATATCAGTTCACAATGCAAAAAATATTAAGAGACTGGATA
AAGGAACACCCTATGATACAATTTGGAATATATATGGATGATATTTATATAGGAAGTGATTTAGAACTAA
GGGAACACAGAGAGATTGTAGAAAACTTGGCTGTATATATTGCACAATATGGATTTATGCTGCCGGAAGA
AAAGAGGCAAGAAGGGTATCCAGCTCAATGGCTTGGATTTGAGTTACATCCAGATAAATGGAGATTTCAA
AAACATGCTTTGCCAGATATCAAGGAAGGGTCAATTACTTTAAACAAGTTGCAAAAATTAGTAGGAGATC
TGGTGTGGCGACAATCATTGATAGGAAAAAGTATTCCGAATATATTAAAGTTAATGGAGGGCGACAGGGC
ACTCCAAAGTGAGAGACAAATAGAAAAAATTCATGTACAGGAATGGGAAATATGTAAGAGAAAATTAATA
GAGATGGAAGGAAATTATTATGATGAAGAAAAGGACATCTATGGACAAATAGATTGGGGAAATAAAGCAA
TTGAATATATAGTGTTTCAGGAAAAAGGGAAACCTTTATGGGTGAATGTAGTGCATAGCATTAAAAATCT
AAGTCAGCCACAACAAATTATTAAAGCAGCCCAGAAGCTAACACAAGAAGTAATCATTAGAACAGGGAAG
ATACCATGGGTACTACTGCCAGGGAAGGAAGAAGATTGGATATTAGAATTACAAATAGGGAATATAACGT
GGATGCCTTCCTTCTGGTCATGCTATAGAGGGTCAGTAAGGTGGAAAAGGAGAAATATAGTAACAGAAGT
AGTAGAGGGACCAACGTACTATACAGATGGAGGTAAGAAAAATGGGATTGGAAATTTAGGATATATTGCC
TCGACAGGAGAGAAATATAGATTACATGAGGAAGGGACAAATCAACAGCTAGAATTGAGAGCAATAGAAG
AGGCATGTAGAAGGGGACCAAGTAAGATGAATATAGTAACTGATAGTAGGTACGCATATGAATTTATGCT
AAGGAACTGGGATGAAGAAGTAATAAGAAACCCTATACAGGCCAGAATCATGAAAATAATCCATGAGAAA
GAAAAAGTGGGAGTGCATTGGGTACCAGGACATAGAGGGATCCCTCAGAATGAGGAAATAGATAAATATA
TATCAGAAGTATTTTTAGCAAAACAAGGGGAAGGGATTGTCCAGAAAAGAGCAGAGGACGCTGGATATGA
CTTAATATGTCCTCAGGAAGTAGTCATCCCACCTGGAGAAGTAAGAAAGGTTCCAATAGATTTAAGAATA
AATTTGAAAGAAAATCAATGGGCCATGATAGCAACAAAAAGTAGTTTTGCAAGTAAGGGAGTCTTTGTAC
AAGGAGGGATAATAGATTCAGGATATCAAGGAACCATACAAGTAGTAATATACAACAGCAATAAGGTAGA
AGTAATAATACCACAAGGAAGAAAGTTTGCGCAATTAATTCTTATGCCTTTGATACATGAAAAATTAGAA
CCATGGGGAACGGCAAGAAGAACTGAGAGAGGGGATGAAGGATTTGGTTCAACAGGAGCCTATTGGATAG
AAAATATCCCTAAGGCGGAAGAGGATCATGATAAATGGCATCAAGATGCAACTTCATTGCACTTAGAGTT
TGGGATCCCTAAATCAGCGGCTGAAGATATAGTACAACAGTGTGAAGTGTGCCAAGAAAATAAGATGCCA
AGTACCCTCAGAGGGGGAAATAAAAGAGGAATAGACCACTGGCAAGTAGATTATACTCATTATGAAGATA
AAATATTATTAGTATGGGTAGAAACAAATTCAGGATTAATTTATGCAGAAAAGGTAAAAGGGGAAACAGG
CCAAGAATTCCGAATACATGTTATGAAATGGTATGCCATGTTTTATCCAAAATCATTGCAGTCTGATAAT
GGACCTGCGTTCGTAGCAGAGCCAACACAGCTCTTAATGAAATATCTAGGAGTACAACATACTACAGGGG
TTCCGTGGAATCCACAATCTCAAGCTTTGGTTGAAAGAACCCATCAAACGTTGAAACATACCCTAGAGAA
ACTTATCCCCATGTTTGTAGCATTTGAATCTGCTCTTGCGGCCTCGCTCATAACTCTAAATATAAAAAGA
AAGGGTGGGCTAGGGACAAGCCCTATGGATATATTCATATTTAATAAAGAACAACAAAGAATACAGCAAC
AATTTCAAGTAAATCACGAAAAAATTCGGTTTTGTTATTACAGGATCAGGAAAAGAGGACATCCAGGTGA
CTGGCAGGGGCCTACCCAGGTATTATGGGAAGGGGAAGGTGCAATAGTAGTAAAAGATAAGCATACAGAA
CGATACTTGGTAATAACTAATAAGGATGTCAGGTTTGTCCCACCACCAAAAGAAATACAAAAGAAATAA

>MT993908.1 Small ruminant lentivirus isolate USMARC-200212120-r, complete genome
GGGAAACAGCAGGAGGGG
GCCACGTGTGGTGCCGTCCGCGCCCCCTATGTTGTAACAGATGCACCACCGAGAATAGAAGCAAGAGTGG
GGACAACTTGGAAGGAATTATTAGTGGATACAGGAGCAGATAGAACTATAGTGAGAAAACATGATAATTC
AGGGATACCAAGGGGAAGAATAAAACTACAAGGAATAGGAGGAATTATAGAAGGGGAAAAATGGGATCAA
GTACAAATACAATATAAAGAAAAAATAATAAAAGGAACAATAGTAGTACTGCCAACAAGTCCAGTAGAAG
TGTTAGGGAGAGACAATATGGGAAAATTGGGGATAGAATTAATTATGGCCAATTTAGAAGAAAAGAAAAT
TCCCATAACGCAGGTAAAATTAAAGGAGGGATGTAAGGGACCCCATATAGCACAGTGGCCTTTAACTCAA
GAAAAGTTGGAAGGGCTGAAAGAAATTATAGATAGATTAGAAAAAGAAGGAAAACTAGGAAGAGCACCAC
CTCATTGGACATGCAATACTCCAATATTTTGCATTAAGAAAAAATCAGGGAAATGGAGAATGTTAATAGA
TTTTAGGGAATTAAACAAGCAAACAGAGGATTTAGCAGAGGCTCAGCTAGGGTTACCGCATCCGGGGGGA
TTGAAGAAGAAGAAAAATGTAACAGTCCTCGATATTGGGGACGCGTATTTTACAATCCCATTATATGAAC
CTTATAGACAGTATACATGCTTTACTCTGCTAAGTCCCAATAATTTGGGACCATGTGTTAGATATTATTG
GAAAGTGTTGCCTCAAGGATGGAAGTTAAGCCCGTCGGTATATCAGTTTACAATGCAGAAAATATTAAAA

GATTGGATAGAGGAACACCCTATGATACAGTTTGGGATATATATGGATGATATTTATATAGGAAGTGATC
TAGAGATAAAAGAGCATAGAAAGATAGTGGATGAATTAGCAAATTATATAGCACAATTTGGCTTCATGTT
GCCTGAAGATAAGAGACAGGAAGGGTATCCAGCCAAGTGGCTAGGATTTGAATTACACCCTGACAAATGG
AAGTTTCAAAAACATACATTAGCAGAGCTAAAAGAAGGACCGATCACATTAAATAAATTGCAAAAATTAG
TAGGGGACTTAGTATGGCGGCAATCATTAATAGGAAAAGGTATTCCAAATATATTAAAGTTAATGGAGGG
AGATAGGGCACTCCAAAGTGAAAGGCAAATAGAAAAAGTTCATGTACAGGAATGGGAAACATGTAAGAAA
AAATTAGAAGAAATGGAAGGAAGTTATTATAATGAAGAAAAGGACATTTATGGGCAAATAGATTGGGGAA
ATAAGGCAATTGAGTATATAGTGTTTCAGGAAAAGGGGAAACCTTTGTGGGTAAATGTAGTCCATAACAT
AAAGAATTTGAGTCAACCACAGCAAATTATTAAAGCAGCACAGAAATTAACACAAGAGGTGATAATAAGA
ACAGGAAAAATACCATGGATCCTGTTACCGGGAAAAGAAGAGGACTGGATTTTAGAAATGCAAATAGGGA
ATATAACGTGGATGCCTTCATTTTGGTCATGTTATAGAGGATCAGTGAGATGGAAAAGGAGAAATATAGT
AGCAGAAGTAGTAGAAGGGCCAACATATTATACCGATGGAGGGAAGAAAAATGGGGTTGGAAATTTAGGC
TACATTGCCTCAACAGGGGAAAAATACAGGATACATGAGGAAGGGACCAATCAGCAGTTAGAACTAAGGG
CAATAGAAGAGGCATGTAAACAGGGACCAAGCAAAATGAATATAGTAACTGACAGCAGATATGCATATGA
ATTTATGCTAAGAAATTGGGATGAAGAAGTAATAAGAAACCCTATACAGGCCAGAATTATGAAAATAATT
CATGAGAAAGAAAAAGTGGGAGTGCATTGGGTACCAGGACATAAAGGGATTCCTCAGAATGAGGAAATAG
ATAAATATATTTCAGAAATATTTCTAGCAAAGGAAGGAGAAGGGATTCTCCCTAAAAGAGAAGAAGATGC
AGGGTATGATTTAATTTGTCCTCAGGAGGTACATATTCCGGCGGGACAAGTAAGAAAAATCCCCGTAGAC
TTAAGATTAAATTTACAAGAGAAGCAATGGGCCATGATAGGGACAAAGAGTAGCTTTGCCAGCAAGGGGG
TATTTGTTCAAGGAGGAATAATAGATTCAGGGTATCAGGGAACAATACAAGTAGTAATATACAATAGTAA
TGATGTGGAAGTGGTAATTCCCCAGGGAAGGAAGTTTGCACAGTTAATTCTGATGCCCTTGATACATGAA
GAATTAGAATCTTGGGGAGAGACAAGGAAAACAGAAAGGGGAAAACAAGGATTTGGATCTACGGGGGCTT
ATTGGATAGAGAATATTCCTAAAGCAGAAGAGGATCATGATAAGTGGCATCAAGATGCAAATTCATTGCA
CTTAGAATTTGGGATTCCTAAATCAGCTGCTGAAGATATAGTGCAACAATGTGAAGTGTGCCAAGAAAAT
AAAATGCCAAGTACCCTCAGAGGAGGAAATAAAAGAGGAATAGATCATTGGCAAGTGGATTACACACATT
ATGAGGATAAAATATTGTTAGTATGGGTAGAAACAAATTCAGGATTAATCTATGCAGAAAGGGTGAAAGG
AGAAACAGGTCAAGAATTCAGAATACATGCTATGAAATGGTATGCCATGTTTAATCCAAAATCATTGCAG
TCCGATAATGGACCGGCTTTTGTAGCGGAGCCAACACAGCTCTTAATGAAATATTTAGGGGTAGAGCACA
CTACAGGAGTTCCATGGAACCCACAATCTCAAGCCTTAGTAGAAAGAACTCATCAAACGTTAAAACATAC
CCTGGAAAAATTTATCCCCATGTTTGCAGCATTTGAATCTGCTCTTGCAGCGGCCCTAATAGCACTAAAT
ATAAAAAGAAAGGGTGGGCTAGGGACAAGCCCTATGGATATATTTATATTTAATAAGGAACAGCAAAGAA
CACAACAACAATATAAGTTAAATCAGTCAAAAATTCGATTTTGTTATTACAGGATCAGGAAAAAAGGACA
TCCAGGCGAGTGGCAAGGGCCAACACAGGTATTGTGGGAAGGGGAAGGTGCAATAGTAGTAAAAGATAAA
CCCACAGAAAGATATTTGGTAGTAACCAACAAGGATGTCAGGTTCATCCCACCACCAAAAGAAATACAAA
AAGAATAA

>MH916859.1 Small ruminant lentivirus isolate 1150, complete genome
GGGAAACAGCAGGAGGGGGCCACGTGTGGTGCCGTCCGCGC
CCCCTATGTTGTAACAGAAGCACCACCCAGAATAGAAGCAAGAGTAGGGACAACTTGGAAGGAATTGTTA
GTGGATACAGGAGCAGATAGAACTATAGTAAGAAAACATGATAATACAGGGATACCAAAGGGAAGAATAA
AGCTTCAAGGGATAGGAGGAATTATAGAAGGGGAAAAATGGGATCAAGTACAAATACAATATAAAGAAAA
AATAATAAGAGGAACGATCGTGGTACTGCCGACAAGTCCAGTAGAAGTATTAGGGAGAGATAATATGGGA
GAATTGGGCATAGGATTGATTATGGCCAATTTAGAAGAAAAGAAAATTCCAATAACACAGGTAAAATTAA
AAGAGGGGTGTACAGGACCTCATATAGCTCAGTGGCCTTTAACTCAAGAAAAATTAGAAGGTCTAAAAGA
GATTGTAGATAGGTTAGAGAAAGAAGGGAAGCTAGGAAGAGCACCACCACATTGGACATGCAATACGCCA
ATATTTTGCATTAAGAAGAAATCAGGGAAATGGAGAATGTTAATAGATTTTAGGGAATTAAACAAGCAAA
CAGAGGATTTGGCAGAGGCACAGCTAGGACTACCGCATCCAGGGGGGTTGAAGAAGAAGAAAAATGTGAC
AGTTCTGGATATTGGGGATGCGTATTTTACAATACCATTATATGAACCTTATAGGCAATATACATGCTTT
ACTCTGCTAAGTCCAAATAATTTAGGGCCATGTGTTAGGTATTATTGGAAAGTGTTGCCTCAAGGATGGA
AGTTAAGCCCGTCGGTATATCAATTTACAATGCAGAAAATATTAGAAGATTGGATAAAGGAACATCCTAT
GATACAGTTTGGGATATATATGGATGATATTTATATAGGAAGTGATCTGGAGATAACAGAACATAGGAAA
ATAGTGGAGGAATTAGCAAATTATATAGTACAGTTTGGCTTTATGTTGCCTGAAGATAAAAGACAGGAAG
GCTATCCAGCCAAGTGGCTAGGATTTGAGCTCCATCCTGACAAATGGAGGTTTCAAAAACATACATTGAC
AGAGATAAAAGAAGGGCCAATTACATTAAATAAGTTGCAAAAATTAGTAGGAGATCTGGTATGGAGACAA
TCATTAATAGGAAAAAGTATTCCAAACATATTAAAATTAATGGAGGGAGATAGGGCACTCCAAAGTGAAA
GACAAATAGAAAAAAATTCATGTACAGGAATGGGAAACATGTAAGAGGAAATTAGAAGAAATGGAAGGAAA
TTATTATGATGAAGAAAAGGACATCTATGGACAAATAGATTGGGGAAATAAAGCAATTGAATATATAGTG
TTTCAGGAGAAAGGGAAACCTTTATGGGTAAATGTAGTCCATAATATTAAAAACTTAAGCCAACCACAGC
AAATTATTAAGGCAGCACAGAAATTAACACAAGAAGTGATAATTAGAACAGGGAAAATACCATGGATACT
ATTACCAGGAAAAGAAGAAGATTGGATGTTAGAATTACAAGTAGGGAATATAACGTGGATGCCATCATTT
TGGTCGTGCTATAGAGGGTCAGTGAGATGGAAAAAGAGAAATGTAGTAGCAGAAGTAGTGGAAGGACCAA

CATATTATACCGATGGAGGGAAGAAAAATGGGATTGGAAATTTAGGCTACATTGCCTCAACAGGGGAAAA
ATATCGGCAACATGAGGAAGGGACTAATCAACAATTAGAATTGAGAGCAATAGAAGAGGCATGTAAGCGA
GGACCAAGCAAAATGAATATAGTAACTGATAGTAAGTATGCATATGAATTTATGTTAAGGAATTGGGATG
AAGAAGTAATAAAAAACCCTATACAGGCCAGAATCATGAAGATAATTCATGGGAAAGAAGAGGTGGGAGT
GCATTGGGTACCAGGACACAAAGGAATTCCTCAAAATGAAGAAATAGATAGATATATCTCAGAAGTATTC
TTAGCAAAAGAAGGAAAAGGAATTCTTCCCAAAAGGGAAGAAGATGCAGGGTATGATTTAATCTGTCCTC
AAGAAGTAAGCATCCCAGCAGGACAAGTAAAAAGAATTCCTATTGATCTAAGATTAAATTTAAAAAGGAA
TCAGTGGGCAATGATAGGGACAAAAAGCAGCTTTGCAAGCAAGGGAGTATTTGTTCAAGGAGGAATAATA
GACTCGGGATATCAAGGCATAGTACAGGTGGTAGTATACAATAGTAATGAAGTGGAAGTAGTAATACCCT
CGGGAAGAAAATTTGCACAATTAATTCTAATGCCCTTAATGCATGAGGAATTAGAAACATGGGGAAAAAC
AAGAAAAACAGAAAGAGGAAAGCAGGGATTCGGGTCTACGGGGGCCTATTGGGTTGAGAATATCCCTAAA
GCAGAGGAAGATCATGAGAAATGGCATCAAGATGCAAATTCATTGCACTTAGAATTTGGGATTCCTAGAT
CAGCGGCTGAAGATATCGTACAACAATGTGAAGTATGTCAGGAAAATAAAATGCCAAGTACCCTCAGAGG
AGGAAATAAAAGAGGAATAGACCATTGGCAAGTAGATTACACTCATTATGAAGATAAAATATTATTAGTA
TGGATAGAAACAAATTCAGGATTAATATATGCAGAAAAGGTAAAAGGGGAAACAGGCCAAGAATTTAGAA
TACATGTTATGAAATGGTATGCCATGTTTTATCCAAAATCATTGCAGTCCGATAACGGACCTGCCTTTGT
GGCAGAGCCAACACAGCTCTTAATGAAATATCTAGGGATAGAGCATACTACAGGGGTCCCGTGGAACCCA
CAATCTCAAGCCTTGGTGGAAAGAAGCCATCAAACATTAAAACATGTTTTAGAAAAATTTATTCCCATGT
TTGTAGCATTTGAATCTGCTCTTGCTGCTGCCCTAATAGCACTAAATATAAAAAGAAAGGGTGGGCTAGG
GACAAGCCCTATGGATATATTCATATTTAATAAAGAACAGCATAGAATACAGCAACAAGATAAATTAAAT
CAATCAAAAATTCGATTTTGTTATTACAGGATCAGGAAAAAAGGACATCCAGGCGAGTGGCAAGGGCCAA
CACAGGTATTGTGGGAAGGAGAAGGGGCAGTAGTGGTAAAAGATAAACACACAGAAAGATATTTGGTAGT
AACTAACAAGGATGTCAGGCTTATCCCCCCACCAAAAGAAATACAAAAAGGATAG

>MZ313871.1 Visna-maedi virus isolate CMV-1, complete genome
ATGCCATCATTGTGGA
AAAAGAGGACATATGCAAAAAGATTGCAGGAAAAAGAAAAGCGAGAACATGAAGCAGCAGGGAAACAGCA
GGAGGGGGCCACGTGTGGTGCCGTCCGCGCCCCCTATGCTGTAACAGAAGCACCACCAATAATAAGAATA
AAAGTAGGGACAAATTGGAAAGAGGTATTAGTAGACACAGGAGCAGACAGGACTATAGTTAGAAAACATG
ATAATTCAGGGGTACCAAAGGGAAGAATAACATTACAAGGAATAGGAGGGATTATAGAAGGGGAAAAATG
GGATCAAGTACAAATACAATATAAAGAAAAAACAATAAGAGGAACAATAGTAGTACTGCCGACAAGTCCG
GTAGAAGTATTAGGGAGAGATAATATGGCAAAATTGGGCATAGGATTAATTATGGCCAATTTAGAAGAAA
AGAAAATTCCCATAACACAGGTAAAATTGAAGGAGGGATGTAAGGGACCCCATATAGCACAGTGGCCTTT
GACTCAAGAAAAATTGGAAGGATTAAAGGAAATAGTAAACAGATTAGAAAAAGAGGGAAAGCTAGGCAGA
GCCCCACCGCATTGGACATGTAACACCCCTATCTTTTGTATTAAGAAAAAGTCGGGGAAATGGAGAATGT
TAATAGATTTTAGGGAGTTAAATAAGCAAACAGAGGATTTGGCAGAAGCACAATTAGGGCTACCGCATCC
GGGGGGATTAAAAAGAAAGAAAAATGTAACAGTCCTCGATATTGGGGATGCGTATTTTACAATACCTTTA
TATGAACCTTATAGACAATATACATGTTTTACTCTGCTAAGTCCAAATAATTTGGGACCATGTGTTAGAT
ACTATTGGAAGGTGCTGCCGCAAGGATGGAAGTTGAGTCCGTCAGTATATCAGTTTACAATGCAGAAGAT
ATTAAAAGATTGGATAGAGGCACATCCTATGATACAGTTTGGAATATATATGGATGATATTTATATAGGA
AGTGATCTAGAGATAAAAAAGCATAGAGAAATAGTAAATGAATTAGCAAGTTATATAGCACAATTTGGCT
TCATGTTGCCTGAAGATAAAAGACAGGAAGGGTATCCAGCCAAGTGGCTAGGATTTGAGCTACACCCTGA
CAAATGGAGGTTTCAAAAACATACATTAGCAGAACTAAAAGAGGGAACAATCACATTAAATAAGTTGCAG
AAGTTAGTAGGGGATTTGGTCTGGCGGCAATCATTGATAGGAAAAAGTATTCCAAATATATTAAAGTTAA
TGGAGGGAGATAGGGCACTCCAAAGTGAAAGGCAAATAGAAAAAAATTCATGTACAAGAATGGGAAACATG
TAGGAAAAAAATTAGCAGAAATGGAAGGAAATTATTATGATGAAGAGAAAGACATTTATGGACAGATAGAT
TGGGGAAATAAAGCAATTGAGTATATAGTGTTTCAAGAGAAAGGGAAACCTTTATGGGTGAATGTAGTCC
ATAATATTAAAAACTTAAGTCAACCACAGCAAATTATTAAAGCAGCCCAGAAATTGACACAGGAAGTGAT
AATAAGAACAGGAAAAATACCATGGATATTATTACCAGGAAAAGAAGAGGATTGGATCTTAGAATTACAA
ATAGGGAATATAACGTGGATGCCATCATTTTGGTCATGTTATAGAGGAGCAGTGAGGTGGAAAAAGAGAA
ATATAGTAACAGAAGTAGTAGAAGGACCAACATACTATACAGATGGAGGGAAGAAAAATGGAATAGGAAA
TTTAGGCTACATTGCCTCGACAGGAGAAAAATATAGGATACATGAAGAAGGGACTAATCAGCAATTAGAA
CTACGGGCAATAGAGGAAGCATGTAAACGGGGACCAAGCAAAATGAATATAGTAACGGATAGCAAGTACG
CATATGAATTTATGCTAAGAAATTGGGATGAAGAGGTAGTAAAAAATCCTATACAGGCCAGAATTATGAA
AATAATTCATGGAAAGGAAAAAGTAGGAGTGCATTGGGTACCAGGACATAGAGGGATCCCTCAAAATGAG
GAAATAGATAAATATATTTCAGAAATATTCCTAGCAAGAGAAGGAGAAGGGATTCTCCCTAAAAGAGAAG
AAGATGCAGGATATGATTTAATTTGTCCTCAAGAGATACATATCCCGGCAGGCCAAGTAAGAAAAATTCC
CATAGATCTAAGGTTAAATTTAAAAAAGAATCAATGGACCATGATAGGAACAAAAAGCAGTTTTGCAAGC
AAGGGAGTATTTGTTCAGGGAGGAATAATAGACTCTGGGTATCAAGGAACAATACAGGTAGTAGTATACA
ATAGCAATGAAATAGAAGTAGTAATACCCCAAGGGAGGAAATTTGCGCAATTAATTCTAATGCCATTGAT
ACATGAAGAATTGGAACATTGGGGGAAAACGAGAAAAACAGAAAGGGGAAAGCAAGGATTTGGATCCACG

GGGGCATATTGGATAGAAAATATCCCTAAAGCAGAAGAGGACCATAATAAATGGCATCAGGATGCAAATT
CATTGCACTTAGACTTTGGGATTCCTAAATCAGCTGCTGAAGATATAGTACAACAATGTGAAGTGTGTCA
GGAAAATAAAATGCCAAGCACCCTCAGAGGAGGAAATAAAAGGGGAATAGACCATTGGCAAGTGGACTAC
ACACATTATGAAGATAAAATATTATTAGTATGGGTAGAAACAAATTCAGGATTAGTTTATGCAGAAAAGG
TAAAAGGAGAAACAGGCCAAGAATTCAGAATACAGGTTATGAAATGGTATGCTATGTTTAATCCAAAATC
ATTGCAGTCTGATAACGGACCTGCCTTTGTAGCGGAGCCAACCCAGCTCTTAATGAAATATCTAGGGATA
GAACACACTACGGGAGTTCCCTGGAACCCACAATCTCAAGCCTTGGTTGAAAGAACCCATCAAACGTTGA
AACATACCTTAGAAAAATTCATCCCCATGTTTGTAGCATTTGACTCTGCTCTTGCAGCCGCACTCATAGC
TCTAAATATAAAAAGAAAGGGTGGGCTAGGGACAAGCCCTATGGATATATTTATATTTAATAAAGAACAA
CAAAGAATACAGCAACAGTCTCAAGTAAATCAATCAAAAATTCGATTTTGTTATTACAGGATCAGGAAAA
AAGGACATCCAGGAGACTGGCAGGGGCCCACACAGGTATTGTGGGAAGGGGAAGGTGCAATAGTGGTAAA
AGATAAATTCACAGAAAGATATTTTGTGGTAACTAACAAGGATGTCAGGTTCATCCCACCACCAAAAGAA
ATACAAAAGGAATAA

>MT993902.1 Small ruminant lentivirus isolate USMARC-200103342-1, complete genome
GGGAAACAGCAGGAGGGGGCCACGT
GTGGTGCCGTCCGCGCCCCCTATGTTGTAACAGAAGCACCACCGAAAATAGAAGCAAGAGTGGGGACAAC
TTGGAAGGAATTATTAGTGGATACAGGAGCAGATAGAACTATAGTAAGAAAACATGATAGCACAGGAATA
CCAAAGGGAAGAATAAAGCTTCAAGGAATAGGAGGAATTATAGAAGGGGAAAAATGGGATCAAGTACAAA
TACAATATAAAGAAAAAATAATAAAAGGAACAATAGTAGTACTGCCCACAAGTCCAGTAGAAGTGTTAGG
GAGAGACAATATGGGAAAATTGGGTATAGAATTAATTATGGCCAATTTAGAGGAAAAGAAAATTCCCATA
ACACAGGTAAAATTGAAAGAGGGATGTAAGGGACCTCAGGTAGCACAGTGGCCTTTAACTCAAGAAAAGT
TGGAAGGGCTGAAAGAAATTATAGATAGATTAGAAAAAGAAGGAAAACTAGGAAGAGCACCACCTCATTG
GACATGCAATACTCCAATATTTTGCATTAGGAAAAAATCAGGGAAATGGAGAATGTTAATAGATTTTAGG
GAATTAAACAAGCAAACAGAGGATTTAGCAGAGGCTCAGCTAGGGTTACCGCATCCGGGGGGATTAAAGA
AGAAGAAAAATGTAACAATCCTCGATATTGGGGACGCATATTTTACAATCCCATTATATGAACCTTATAG
ACAGTATACATGCTTTACTCTGCTAAGTCCCAATAATTTGGGACCATGTGTTAGATATTATTGGAAAGTG
TTGCCTCAAGGATGGAAGTTAAGCCCGTCGGTATATCAGTTTACAATGCAGAAAATATTAAAAGATTGGA
TAGAGGAACACCCTATGATACAGTTTGGGATATATATGGATGATATTTATATAGGAAGTGATCTAGAAAT
AACAGAGCATAGGAAAATAGTAGAAGGATTAGCAAATTATATAGCACAGTTTGGCTTTATGGTGCCTGAA
GATAAAAGGCAAGAAGGGTATCCAGCCAAGTGGCTAGGATTTGAGTTACATCCTGACAAATGGAGGTTTC
AAAAACATACATTAGCAGAGCTAAAAGAAGGACCGATCACATTAAATAAATTGCAAAAATTAGTAGGGGA
CTTAGTCTGGAGACAATCATTAATAGGAAAAAGTATTCCAAATATATTAAAGTTAATGGAGGGAGATAGG
GCACTCCAAAGTGAAAGGCAAATAGAAAAAGTTCATGTACAGGAGTGGGAAACATGTAAGAAAAAAATTAG
AAGAAATGGAAGGAAGTTATTATAATGAAGAAAAGGACATTTATGGGCAAATAGATTGGGGAAATAAGGC
AATTGAGTATATAGTGTTTCAGGAGAAGGGGAAACCTTTGTGGGTAAATGTGGTCCATAATATTAAAAAC
TTAAGTCAACCACAACAAATTATTAAGGCAGCACAGAAGTTAACACAAGAAGTGATAATAAGAACAGGGA
AAATACCATGGATTTTATTACCAGGAAAAGAAGAAGATTGGATTTTGGAATTGCAAATAGGGAATATCAC
ATGGATGCCTTCATTTTGGTCATGCTACAGGGGATCAGTAAGGTGGAAAAGGAGAAATGTAGTAACGGAA
ATAGTAGAAGGACCAACATATTATACCGATGGAGGGAAGAAAAATGGGGTTGGAAATTTAGGCTACATTG
CCTCAACAGGGGAAAAATACAGGAGACATGAGGAAGGGACCAATCAGCAGTTAGAACTAAGGGCAATAGA
AGAGGCATGTAAACAGGGACCAAGCAAAATGAATATAGTAACTGACAGCAGATATGCATATGAATTTATG
CTAAGGAATTGGGATGAAGAAGTAATAAGAAACCCTATACAGGCCAGAATTATGAAAATAATTCATGGAA
AAGAAAAGGTGGGAGTACATTGGGTTCCAGGACATAAAGGAATCCCTCAAAATGAGGAGGTAGATAAATA
TATTGCAGAAATATTTCTAGCAAAAGAAGGAGAAGGGATTCTCCCAAAAAGGGAGGAAGATGCAGGGTAT
GATTTAATTTGTCCTCAGGAGGTACATATTCCGGCGGGACAAGTAAGAAAAATCCCCGTAGACTTAAGAT
TAAATTTAAAAGAGAAGCAATGGGCCATGATAGGGACAAAGAGTAGCTTTGCCAGCAAGGGGGTATTTGT
TCAAGGAGGAATAATAGATTCAGGGTATCAGGGAACAATACAAGTAGTAATATACAATAGTAATGATGTG
GAAGTGGTAATTCCCCAGGGAAGGAAGTTTGCACAGTTAATTCTGATGCCCTTGATACATGAAGAATTAG
AATCTTGGGGAGAAACAAGGAAAACAGAAAGGGGAAAACAAGGATTTGGATCTACGGGGGCTTATTGGAT
AGAGAATATTCCTAAAGCAGAAGAGGATCATGATAAGTGGCATCAAGATGCAAATTCATTGCACTTGGAA
TTTGGGATCCCTAGATCAGCTGCTGAAGATATAGTACAACAATGTGAGGTGTGTCAAGAAAATAAGATGC
CAAGTACCATCAGAGGAGGCAATAAAAGAGGAATAGATCATTGGCAGGTAGATTATACACATCATGAAGA
GAAAATATTATTAGTATGGGTAGAAACAAATTCAGGATTGATTTATGCAGAACGGGTAAAAGGGGAAACA
GGCCAAGAATTCAGAGTACACGTTATGAAATGGTATGCCATGTTTCATCCAAAATCATTGCAGTCTGATA
ACGGACCTGCCTTTGTAGCAGAGCCAACGCAGCTCTTAATGAAATATCTAGGAATAGAGCACACTACAGG
AGTTCCATGGAACCCACAATCTCAAGCCTTAGTAGAAAGAACTCATCAAACGTTAAAACATACCCTAGAA
AAATTTATCCCCATATTTGCAGCATTCGAATCTGCCCTTGCCGCAGCCCTAATAGCACTTAATATAAAAA
GAAAGGGTGGGCTAGGGACAAGCCCTATGGATATATTTATATTTAATAAGGAACAGCAAAGAACACAACA
ACAATATAATTTAAATCAGTCAAAAATTCGATTTTGTTATTACAGGATCAGGAAAAAAGGACATCCAGGC

GAGTGGCAAGGGCCAACACAGGTATTGTGGGAAGGGGAAGGTGCAATAGTAGTAAAAGATAAACCCACAG
AAAGATATTTGGTAGTAACCAACAAGGATGTCAGGTTCATCCCACCACCAAAAGAAATACAAAAAGAATA
A

>HQ848062.1 Visna/maedi virus isolate 697, complete genome
ATGTCATCATTGTGGAAAAAGGGGACATATGCAAAAAGA
TTGCAGACAGAAGAAACAACAGGGAAACACCAAGAGGGGGCCACGTGTGGTGCCGTCCGCGCCCCCTATG
TTGTAACAAAAGCACCACCAAGAATAAAAGTAAGAGTAGGGACACAATGGAAGGAATTGTTAGTAGATAC
AGGAGCCGATAGGACAATAGTAAAAAATCATGATAATTCAGGAAGACCAAGAGGAAGAATAACGTTACAA
GGAATAGGAGGGATTATAGAAGGGGAAAAATGGGATCAAGTAGACATACAATATAAAGAAAAAATCATAA
GAGGGCAAATAGTGGTGTTACCTACTAGCCCAGTAGAAGTACTGGGAAGGGATAATATGTTAGAGTTAGG
AATTGGATTAATTATGGCAAATTTGGAAGAGAAGAAAATTCCTATTACTGAGGTAAAATTAAAGGAAGGA
TGTAAAGGGCCACATATAGCACAATGGCCGTTAACACAGGAAAAATTAGAAGGATTAACAGAAATAGTAG
ATAGGCTAGAGAAGGAAGGGAAGTTAGGGAGAGCACCGCCACATTGGACATGTAATACCCCCATATTTTG
CATCAAAAAGAAGTCGGGGAAATGGCGGATGCTAATAGATTTCAGGGAGTTAAATAAGCAAACAGAAGAC
TTGGCAGAAGCACAATTAGGGTTACCGCATCCGGGGGGATTACAGAGGAAAAAGAATGTAACAATATTAG
ATATAGGGGATGCGTATTTTACAATACCATTGTATGAACCATATAGAAAATACACATGTTTTACTTTACT
AAGTCCTAATAATTTGGGACCATGTGTAAGATATTATTGGAAGGTATTACCACAGGGGTGGAAATTAAGT
CCATCGGTGTATCAGTTCACAATGCAAGAAATACTAAGAAACTGGATAACAAGACATCCTGAGATACAAT
TTGGAATTTACATGGATGATATCTATATAGGGAGTGACCTGGAATTAAGAGAGCATAGAGGCGTAGTAGA
GGAATTAGCTAACTATATTGCGCAATATGGCTTTATGTTACCCGAAGAGAAAAGACAGGAAGGGTATCCA
GCGAAATGGCTTGGATTTGAATTACATCCGGAGAAATGGAAATTTCAGAAACATACACTTCCGGAAATTA
CAGAAGGACCCATAACCTTAAATAAATTGCAAAAATTAGTAGGGGATTTAGTATGGAGGCAATCATTAAT
AGGAAAAAGTATTCCCCATATACTAAAATTAATGGAAGGAGATAGAGCTTTGCAAAGTGAAAGAGTTATA
GAGACAATACATGTAAAAGAATGGGAACAATGCAAGAAGAAATTAGCAGAAATGGAAGGACATTATTATA
ATGAAGAAAAGGATATTTATGGACAAGTAGACTGGGGAGACAGAGCAATAGAATACATAGTATTCCAAGA
AAAAGGAAAACCTCTATGGGTTAATGTAGTACACAGCATTAAGAACTTGAGTCAAGCTCAACAAATTATT
AAAGCGGCTCAAAAGCTTACACAAGAAGTAATTGTTAGGACAGGGAAAATACCGTGGATATTATTACCTG
GAAAAGAAGAGGATTGGATATTAGAATTACAAATAGGAAATATAACCTGGATGCCATCATTTTGGTCGTG
TTATAGAGGATCAGTAAGATGGAAGAGAAGGAATGTAGTAACAGAGGTAGTGGAAGGACCAACATATTAT
ACAGATGGAGGCAAGAAAAATGGACTAGGAAGTTTAGGGTATATAGCCTCAACAGGAGATAAGTATAGAA
TACATGAAAAAGGGACAAATCAGCAATTAGAGTTGAGAGCAATAGAGGAAGCATGTAAGCACGGGCCAAC
GAGGATGAATATAGTAACAGATAGCAGATATGCATATGAATTTATGATAAGAAATTGGGATGAAGAAGTT
ACAAAAAATCCGATTCAAGCAAGAATTATGAAATTAATTCATAACAAAGAAAAGGTAGGGATACATTGGG
TACCGGGGCATAAAGGAATCCCTCAGAATGAGGAAATAGATAGATATATTTCAGAGATATTTTTGGCAAA
GGAAGGGGAAGGGATTCTAAGGAAAAGACAAGAAGATGCGGGATATGACCTAATCTGTCCTCAAGAAACA
ATTATCCCAGCAAGGGAGGTGAGAAAGATCCCCATAGATCTACGGCTGAATTTAAAAAGGAATCAATGGG
CTATGATAGGGACAAAAAGCAGTTTTGCTAGCAAAGGAGTATTTGTCCAAGGAGGAATAATAGATTCAGG
ATATCAAGGAACAATACAAGTAATAGTACACAACAGTAATAATATAGATGTGGTAATACCACAAGGGAGA
AAATTTGCACAATTAATTTTAATGCCACTAATACATGAAGAGTTAGAGCACTGGGGGCAAACCAGGAAAA
CAGAGAGAGGAGAGAAAGGATTTGGGTCCACTGGGGCCTATTGGATAGAGAATATCCCTAAAGCAGAAGA
AGACCATGCTAAGTGGCATCAAGATGCTAACTCAATGCACCTGGAGTATGGGATTCCCAGAACAGCTGCG
GAAGATATAGTACGTCAGTGTGAAGTGTGTCAAGAGAATAAAATGCCTAGCACTCTTAGAGGAGGAAATA
AGAGGGGAATAAATCATTGGCAAGTGGATTACACTCATTATGAGGACAAAATAATATTGGTATGGGTAGA
AACAAATTCAGGGTTAATTTACGCAGAAAAAGTAAAAGGAGAAACAGGACAAGAATTCAGAGTACAAGCG
ATGAGATGGTACTCACTGTTTACTCCAACATCAGTGCAATCAGATAATGGACCTGCATTCATAGCAGAAC
CAACACAGCTCTTAATGAAATATTTGGGGGTAGAACATACAACAGGAATTCCGTGGAATCCACAATCTCA
AGCATTAGTAGAAAGAGCACACCAAACATTAAAACATATGTTAGAAAAGTTTATTCCCATGTTCGCTGCA
TTTGAATCTGCTCTGGCCGCTGCCCTAATAGCACTAAATATAAAAAGAAAGGGTGGGCTAGGGACAAGCC
CTATGGATATCTTTATATATAATAAAGAACAGCAAAGAATACAACAGCAAACTGTATTAAATCACTCAAA
AATTCGATTTTGCTATTACAGGATCAGGAAAAAAGGACATCCAGGCGAGTGGCTTGGACCAACACAGGTA
CTGTGGGAAGGAGAAGGGGCTATAGTAGTAAAGGATAAAGAAACAGAACGGTATTTAGTAATAGCAAGAA
AAGATGTTAAATTCGTCCCGGAGCCAAAGCAGATCAAAACAAGAGAGGCATAA

>S51392.1 gag...rev [maedi-visna-like virus EV1, Genomic RNA Complete,
6 genes, 9203 nt]
CATACAGCTGCAGGGAAACAACAGGAGGGGGCCACG
TGTGGTGCCGTCCGCGCCTCCTATGTTGTAACAGAAGCACCACCAAAGGCAGAAATAAAGGTAGGGACAA
CATGGAGAATGTTATTAGTAGACACCGGAGCAGATAGGACAATAGTAAGATATCATGATAATTCGGGAAT
ACCAAAAGGAAGAATAAAATTGCAGGGTATAGGGGGAATTATAGAAGGAGAAAAATGGGACAAAGTGGCG
TTACAGTATAAAGAAAAAAGAATCTTGGGTACAATAGTAGTACTGCCTAGCAGTCCAGTGGAGGTATTAG

GAAGGGATAATAGGGAGAATTAGGAATAGGACTAATTATGGCAAATCTGGAAGAAAAGAAAAATTCCTAT
TACCAAGGTAAGCCTAAAAGAAGGCTGCAAAGGACCTCATATAGCGCAGTGGCCTTTGACTCAAGAAAAA
TTAGAAGGACTAAAAGAAATAGTGGAAAGATTAGAAAAAGAAGGGAAATTAGGTAGAGCACCTCCACATT
GGACATGCAACACTCCTATATTTTGTATTAAAAAGAAATCAGGAAAATGGAGAATGTTAATAGACTTCAG
GGAATTGAATAAACAAACAGAAGATCTGGCAGAGGCACAGTTGGGATTACCACATCCGGGAGGATTGCAG
AAAAAGAAACATGTAACAGTATTGGATATAGGAGATGCATATTTTACAATACCATTATATGAACCTTATA
GACCGTATACATGTTTTACCATGCTGAGTCCCAATAATTTGGGACCTTGTGTAAGGTATTATTGGAAAGT
GTTGCCACAAGGATGGAAATTGAGTCCCTCAGTGTATCAATTTACCATGCAGGAGATATTAAGAGATTGG
ATAAGGGAACACCCTATGGTGCAATTTGGGATATATATGGATGATATCTATATAGGCAGTGATTTAGAAA
TGGGGGAACACAGAAGAATAGTAGAAGAACTTGCCAGTTATATTGCCCAATATGGGTTTATGCTGCCGGA
AGAAAAGAGGCAAGAAGGGTATCCAGCAAATTGGCTTGGATTTGAACTACATCCAGAGAGATGGAAGTTT
CAAAAACATAAGCTTGCAAATATGGAAGAAGGACCAATAAGGTTAAATAAATTGCAGAAATTAGTAGGAG
AATTAGTTTGGAGGCAATCATTGATAGGGAAAAGTATACCAAATATACTGAAATTGATGGAAGGAGATAG
AGCGTTACAAAGTGTAAGGAATGTAGAGACAATACATAAAGAAGAATGGGAAAGATGTAGAAGAAAACTA
GAAGAAATGGAAGGGAATTATTATAATGCAGAAAGGGACGTTTATGGACAAGTAGATTGGGGAAATAAAG
CAATAGAATATATAGTGTTCCAAGAAAAAGGGAAACCATTATGGGTCAATGTGGTACATGCAATTAAAAA
TCTGAGTCAAGCACAGCAAATCATTAAAGCGGCACAAAAACTTACACAGGAAGTAATAATAAGAACTGGG
AAAATACCATGGATACTATTGCCAGTAAAAGAGGAGGATTGGATTTTAGAACTGCAGGTGGGAAATATCA
CGTGGATGCCATCATTTTGGTCATGTTATAGAGGGTCAGTACGATGGAAGAGAAGAAATGTGGTCACAGA
AGTAGTAGAAGGACCAACATATTATACTGATGGTGGCAAGAAGAATGGAATAGGGAGTTTAGGGTATATT
GCTTCCACCGGAGAAAAATATAGAATACATGAAAATGGGACAAATCAGCAATTAGAATTAAGGGCAATTG
AGGAAGCATGTAAACAGGGACCAAGCAAAATGAATATAGTAACAGATAGTAGATATGCATTTGAGTTCAT
GATAAGGAACTGGGATGAAGAAGTTATAAAGAATCCAATACAGGCACGAATCATGAAGTTGATACATAGT
AAGGAGAAGGTAGGGATACATTGGGTGCCAGGCCATAAAGGGATTCCTCAAAATGAAGAAATAGATAAAT
ATATTTCAGAAGTATTTTTAGCAAAAGAAGGAAATGGGATAGTAAAGAAAAGAGCAGAGGATGCTGGGTA
TGATTTGATATGCCCACAAGAGGTAAGTATCCCAGCAGGACAAGTAAAGAAGATTCCAATTGATTTAAGA
ATAAATTTAAGAAAAAATCAATGGGCTATGATAGGGACAAAAAGCAGTTTTGCAAGTAAGGGAGTATTTG
TACAAGGAGGAATAGTAGATTCAGGATATCAGGGAATCATACAAGTAGTAATTTATAACAGCAATGACGA
AGAGGTCATTATACCCCAGGGGAGGAAATTTGCACAGTTAATTCTCATGCCGCTGATACATGAGGAATTA
GAGCCATGGGGGGAAACAAGAAAAACAGAAAGGGGAAATCAGGGATTTGGATCAACAGGAGCATATTGGA
TTGAAAATATTCCCTTGGCGGAAGAAGATCATAGTAAATGGCATCAAGATGCTCAATCATTGCATCTAGA
ATTTGACATACCAAGAACAGCGGCTCAAGATATAGTGCAACAATGTGAAATATGTCAAGAAAATAAAATG
CCTAATACAATGAGAGGAAGTAACAAGAGGGGAATAGATCATTGGCAAGTGGATTACACTCATTTTGAAG
ATAAGATATTACTAGTATGGGTAGAAACAAATTCGGGATTAATTTATGCAGAAAGGGTGAAAGGGGAGAC
AGGACAAGAATTTAGAGTAACAACTATGAGGTGGTATGCTCTGTTCGCCCCAAAATCATTGCAGTCAGAT
TATGGGCCAGCATTTATAGCAGAAGCAACACAATTGTTAATGACATATCTAGGTGTAGAACACACAACCG
GCATACCTTGGAATCCACAGTCTCAAGCTCTCGTAGAAAGAGCTCATCAGACTTTAAAGAAAACAATTGA
AAAACTTGTTCCTATGTTCTCTGCATTTGAATCACGTGTCGCAGCTGCATTAATAGCTCTAAATATAAAA
AGAAAGGGTGGGCGAGGGACAAGCCCTATGGATATATTCATATTTAATAAGGAACAGCAAAGAATACAAC
AACAGTATAAATTAAATCAAGAAAAAATTCGATTTTGTTATTCAGAGATCAGAAAAAGAGGACACCCGGG
AGACTGGCTGGGACCGTCTCAGGTACTCTGGGAAGGGGAAGGAGCTAAGATCGTAAAAGATAGAACTCTA
GATAAGTATTTAGTAATAGCTAATAAAGATGTTAAATTCATACCGCAGCCAAAAGAAATACAAAAAGAGC
AAAAA
>KY358787.1 Small ruminant lentivirus isolate USMARC-200303013-1,
complete genome
GGGAAACAGCAGGAGGG
GGCCACGTGTGGTGCCGTCCGCGCCCCCTATGTTGTAACAGAAGCACCACCGAAAATAGAAGCAAGAGTG
GGGACAGCTTGGAAGGAATTATTAGTGGATACAGGAGCAGATAGAACTATAGTAAGAAAACATGATAGCA
CAGGAATACCAAAGGGAAGAATAAAGCTTCAAGGAATAGGAGGAATTATAGAAGGGGAAAAATGGGATCA
AGTACAAATACAATATAAAGAAAAAATAATAAAAGGGACAATAGTAGTACTGCCAACAAGTCCAGTAGAA
GTGTTAGGGAGAGACAATATGGGAAAATTGGGTATAGAATTAATTATGGCCAATTTAGAAGAAAAGAAAA
TTCCCATAACGCAGGTAAAATTGAAAGAGGGATGTAAGGGACCTCAGGTAGCACAGTGGCCTTTAACTCA
AGAAAAGTTGGAAGGGCTGAAAGAAATTATAGATAGATTAGAAAAAGAAGGAAAACTAGGAAGAGCACCA
CCGCATTGGACATGCAATACACCAATATTTTGCATTAGGAAAAAGTCAGGGAAATGGAGAATGTTAATAG
ATTTTAGGGAATTAAACAAGCAAACAGAGGATTTAGCAGAGGCTCAGTTAGGGTTACCGCATCCGGGGGG
ATTGAAGAAGAAGAAAAATGTAACAGTCCTCGATATTGGGGACGCGTATTTTACAATCCCATTATATGAA
CCTTATAGACAGTATACATGCTTTACTCTGCTAAGTCCCAATAATTTGGGACCATGTGTTAGATATTATT
GGAAAGTGTTGCCTCAAGGATGGAAGTTAAGCCCGTCGGTATATCAGTTTACAATGCAGAAAATATTAAA
AGATTGGATAGAGGAACACCCTATGATACAGTTTGGGATATATATGGATGATATTTATATAGGAAGTGAT
CTAGAAATAACAGAGCATAGGAAAATAGTAGAAGAATTAGCAAATTATATAGCACAGTTTGGCTTTATGG
TGCCTGAAGATAAAAGGCAAGAAGGGTATCCAGCCAAGTGGCTAGGATTTGAGTTACATCCTGACAAATG

GAGGTTTCAAAAACATACATTAGCAGAGCTAAAAGAAGGACCGATCACATTAAATAAATTGCAAAAATTA
GTAGGGGACTTAGTCTGGAGACAATCATTAATAGGAAAAGGTATTCCAAATATATTAAAGTTAATGGAGG
GAGATAGGGCACTCCAAAGTGAAAGGCAAATAGAAAAAGTTCATGTACAGGAGTGGGAAACGTGTAAGAA
AAAATTAGAAGAAATGGAAGGAAGTTATTATAATGAAGAAAAGGACATTTATGGGCAAATAGATTGGGGA
AATAAAGCAATTGAGTATATAGTGTTTCAGGAGAAGGGGAAACCTTTGTGGGTAAATGTGGTCCATAATA
TTAAAAACTTAAGTCAACCACAACAAATTATTAAGGCAGCACAGAAGTTAACACAGGAAGTGATAATAAG
AACAGGGAAAATACCATGGATTTTATTGCCAGGAAAAGAAGAAGATTGGATTTTGGAATTGCAAATAGGG
AATATCACATGGATGCCTTCATTTTGGTCATGCTACAGGGGATCAGTAAGGTGGAAAAGGAGAAATGTAG
TAACAGAAATAGTAGAAGGACCAACATATTATACCGATGGAGGGAAGAAAAATGGGGTTGGAAATTTAGG
CTACATTGCCTCAACAGGGGAAAAATACAGGATACATGAGGAAGGGACCAATCAGCAGTTAGAACTAAGG
GCAATAGAAGAGGCATGTAAACAGGGACCAAGCAAAATGAATATAGTAACTGACAGCAGATATGCATATG
AATTTATGCTAAGGAATTGGGATGAAGAAGTAATAAAAAACCCTATACAGGCCAGAATTATGAAAATAAT
TCATGGAAAAGAAAAGGTGGGAGTACATTGGGTTCCAGGACATAAAGGAATTCCTCAAAATGAGGAGGTA
GATAAATATATTGCAGAAATATTTCTAGCAAAAGAAGGAGAAGGGATTCTCCCAAAAAGGGAGGAAGATG
CAGGGTATGATTTAATTTGTCCTCAGGAGGTACATATTCCGGCGGGACAAATAAGAAAAATCCCTGTAGA
CTTGAGATTAAATTTAAAAGAGACGCAATGGGCCATGATAGGGACAAAGAGTAGCTTTGCCAGCAAGGGG
GTATTTGTTCAAGGAGGAATAATAGATTCAGGGTATCAGGGAACAATACAAGTAATAATATACAATAGTA
ATGATGTGGAAGTGGTAATTCCCCAGGGAAGGAAGTTTGCACAGTTAATTCTGATGCCCTTGATACATGA
AGAATTAGAATCTTGGGGAGAAACAAGGAAAACAGAAAGGGGAAAACAAGGATTTGGATCTACGGGGGCT
TATTGGATAGAGAATATTCCTAAAGCAGAAGAGGATCATGATAAGTGGCATCAAGATGCAAATTCATTGC
ACTTAGAATTTGGGATCCCTAGATCAGCTGCTGAAGATATAGTACAACAATGTGAGGTGTGTCAAGAAAA
TAAGATGCCAAGTACCATCAGAGGTGGGAATAAAAGAGGAATAGATCATTGGCAGGTAGATTATACACAT
CATGAAGATAAAATATTATTGGTATGGGTAGAAACAAATTCAGGATTAATTTATGCAGAAAGGGTAAAAG
GGGAAACAGGCCAAGAATTCAGAGTACACGTTATGAAATGGTATGCCATATTTCATCCAAAATCATTGCA
GTCTGATAACGGACCTGCCTTTGTAGCAGAGCCAACGCAGCTCTTAATGAAATATCTAGGAATAGAACAC
ACTACAGGAGTTCCATGGAACCCACAATCTCAAGCCTTAGTGGAAAGAACTCATCAAACGTTAAAACATA
CCCTGGAAAAATTTTTCCCCATATTTGCAGCATTCGAATCTGCCCTTGCCGCGGCCCTAATAGCACTTAA
TATAAAAAGAAAGGGTGGGCTAGGGACAAGCCCTATGGATATATTTATATTTAATAAGGAACAGCAAAGA
ACACAACAACAATATAATTTAAATCAGTCAAAAATGCGATTTTGTTATTACAGGATCAGGAAAAAAGGAC
ATCCAGGCGAGTGGCAAGGGCCAACACAGGTATTGTGGGAAGGGGAAGGTGCAATAGTAGTAAAAGATAA
ACCCACAGAAAGATATGTGGTAGTAACCAACAAGGATGTCAGGTTCGTCCCACCACCAAAAGAAATACAA
AAAGAATAA

>MT993903.1 Small ruminant lentivirus isolate USMARC-199835918-1, complete genome
GGGAAACAGCAGGAGGGGGCCACGTGTG
GTGCCGTCCGCGCCCCCTATGTTGTAACAGAAGCACCACCGAAAATAGAAGCAAGAGTGGGAACAACTTG
GAAGGAATTATTAGTGGATACAGGAGCAGATAGAACTATAGTAAGAAAACATGATAGCACAGGAATACCA
AAGGGAAGAATAAAGCTTCAAGGAATAGGAGGAATTATAGAAGGGGAAAAATGGATCAAGTACAAATAC
AATATAAAGAAAAAATAATAAAAGGAACAATAGTAGTACTGCCAACAAGTCCAGTAGAAGTGTTAGGGAG
AGACAATATGGGAAAATTGGGTATAGAATTAATTATGGCCAATTTAGAAGAAAAGAAAATTCCCATAACG
CAGGTAAAATTGAAAGAGGGATGTAAGGGACCTCAGGTAGCACAGTGGCCTTTAACTCAAGAAAAGTTGG
AAGGGCTAAAAGAAATTATAGATAGATTAGAAAAAGAAGGAAAACTAGGAAGAGCACCACCTCATTGGAC
ATGCAATACTCCAATATTTTGCATTAGGAAAAAATCAGGGAAATGGAGAATGTTAATAGATTTTAGGGAA
TTAAACAAGCAAACAGAGGATTTAGCAGAGGCTCAGCTAGGGTTACCGCATCCGGGGGGATTGAGGAAGA
AGAAAAATGTAACAATCCTCGATATTGGGGACGCGTATTTTACAATCCCATTATATGAACCTTATAGACA
GTATACATGCTTTACTCTGCTAAGTCCCAATAATTTGGGACCATGTGTTAGATATTATTGGAAAGTGTTG
CCTCAAGGATGGAAGTTAAGCCCGTCGGTATATCAGTTTACAATGCAGAAAATATTAAAGGATTGGATAG
AGGAACACCCTATGATACAGTTTGGGATATATATGGATGATATTTATATAGGAAGTGATCTAGAAATAAC
AGAGCATAGGAAAATAGTAGAAGGATTAGCAAATTATATAGCACAGTTTGGCTTTATGGTGCCTGAAGAT
AAAAGGCAAGAAGGGTATCCAGCCAAGTGGCTAGGATTTGAGTTACATCCTGACAAATGGAGGTTTCAAA
AACATACATTAGCGGAGCTAAAAGAAGGACCGATCACATTAAATAAATTGCAAAAATTAGTAGGGGACTT
AGTCTGGAGACAATCATTAATAGGAAAAGGTATTCCAAATATATTAAAGTTAATGGAGGGAGATAGGGCA
CTCCAAAGTGAAAGGCAAATAGAAAAAGTTCATGTACAGGAGTGGGAAACATGTAAGAAAAAATTAGAAG
AAATGGAAGGAAGTTATTATAATGAAGAAAAGGACATTTATGGGCAAATAGATTGGGGAAATAAGGCAAT
TGAGTATATAGTGTTTCAGGAGAAGGGGAAACCTTTGTGGGTAAATGTGGTCCATAATATTAAAAACTTA
AGTCAACCACAGCAAATTATTAAGGCAGCACAGAAGTTAACACAGGAAGTGATAATAAGACTAGGGAAAA
TACCATGGATTTTATTACCAGGAAAAGAAGAAGATTGGATTTTGGAATTGCAAATAGGGAATATCACATG
GATGCCTTCATTTTGGTCATGCTACAGGGGATCAGTAAGGTGGAAAAGGAGAAATGTAGTAACGGAAATA
GTAGAGGGACCAACATATTATACCGATGGAGGGAAGAAAAATGGGGTTGGAAATTTAGGCTACATTGCCT
CAACAGGGGAAAAATACAGGATACATGAGGAAGGGACCAATCAGCAGTTAGAACTAAGGGCAATAGAAGA

GGCATGTAAACAGGGACCAAGCAAAATGAATATAGTAACTGACAGCAGATATGCATATGAATTTATGCTA
AGGAATTGGGATGAAGAAGTAATAAGAAACCCTATACAGGCCAGAATTATGAAAATAATTCATGGAAAAG
AAAAGGTGGGAGTACATTGGGTTCCAGGACATAAAGGAATTCCTCAAAATGAGGAGGTAGATAAATATAT
TGCAGAAATATTTCTAGCAAAAGAAGGAGAAGGGATTCTCCCAAAAAGGGAGGAAGATGCAGGGTATGAT
TTAATTTGTCCTCAGGAGGTACATATTCCGGCGGGACAAGTAAGAAAAATCCCCGTAGACTTAAGATTAA
ATTTACAAGAGAAGCAATGGGCCATGATAGGGACAAAGAGTAGCTTTGCCAGCAAGGGGGTATTTGTTCA
AGGAGGAATAATAGATTCAGGGTATCAGGGAACAATACAAGTAGTAATATACAATAGTAATGATGTGGAA
GTGGTAATTCCCCAGGGAAGGAAGTTTGCACAGTTAATTCTGATGCCCTTGATACATGAAGAATTAGAAT
CTTGGGGAGAAACAAGGAAAACAGAAAGGGGAAAACAAGGATTTGGATCTACGGGGGCTTATTGGATAGA
GAATATTCCTAAAGCAGAAGAGGATCATGATAAGTGGCATCAAGATGCAAATTCATTGCACTTAGAATTT
GGGATCCCTAGATCAGCTGCTGAAGATATAGTACAACAATGTGAGGTGTGTCAAGAAAATAAGATGCCAA
GTACCATCAGAGGAGGGAATAAAAGAGGAATAGATCATTGGCAGGTAGATTATACACATCATGAAGAGAA
AATATTATTAGTATGGGTAGAAACAAATTCAGGATTGATTTATGCAGAACGGGTAAAAGGGGAAACAGGC
CAAGAATTCAGAGTACACGTTATGAAATGGTATGCCATGTTTCATCCAAAATCATTGCAGTCTGATAACG
GACCTGCTTTTGTAGCAGAGCCAACGCAGCTCTTAATGAAATATCTAGGAATAGAGCACACTACAGGAGT
TCCATGGAACCCACAATCTCAAGCCTTAGTAGAAAGAACTCATCAAACGTTAAAACATACCTTGGAAAAA
TTTATCCCCATATTTGCAGCATTCGAATCTGCCCTTGCCGCGGCCCTAATAGCACTTAATATAAAAAGAA
AGGGTGGGCTAGGGACAAGCCCTATGGATATATTTATATTTAATAAGGAACAGCAAAGAACACAACAACA
ATATAATTTAAATCAGTCAAAAATTCGATTTTGTTATTACAGGATCAGAAAAAAAGGACATCCAGGTGAG
TGGCAAGGGCCAACACAGGTATTGTGGGAAGGGGAAGGTGCAATAGTAGTAAAAGATAAACCCACAGAAA
GATATTTGGTAGTAACCAACAAGGATGTCAGGTTCATCCCACCACCAAAAGAAATACAAAAAGAATAA

>MT993899.1 Small ruminant lentivirus isolate USMARC-200103515-1,
complete genome
GGGAAACAGCAGGAGGGGGCCACGTGTGGTGCCGTCCGCGCCCCCTATGTTGTA
ACAGAAGCACCACCGAAAATAGAAGCAAGAGTGGGGACAACTTGGAAGGAATTACTAGTGGATACAGGAG
CAGATAGAACTATAGTAAGAAAACATGATAGCACAGGAATACCAAAGGGAAGAATAAAGCTTCAAGGAAT
AGGAGGAATTATAGAAGGGGAAAAATGGGATCAAGTACAAATACAATATAAAGAAAAAATAATAAAAGGA
ACAGTAGTAGTACTGCCAACAAGTCCAGTAGAAGTGTTAGGGAGAGACAATATGGGAAAATTGGGTATAG
AATTAATTATGGCCAATTTAGAAGAAAAGAAAATTCCCATAACGCAGGTAAAATTGAAAGAGGGATGTAA
GGGACCTCAGGTAGCACAGTGGCCTTTAACTCAAGAAAAGTTAGAAGGGCTGAAAGAAATTATAGATAGA
TTAGAAAAAGAAGGAAAACTAGGAAGAGCACCACCTCATTGGACATGCAATACTCCAATATTTTGCATTA
GGAAAAAATCAGGGAAATGGAGAATGTTAATAGATTTTAGGGAATTAAACAAGCAAACAGAGGATTTAGC
AGAGGCTCAGCTAGGGTTACCGCATCCGGGGGGATTGAAGAAGAAGAAAAATGTAACAGTCCTCGATATT
GGGGACGCGTATTTTACAATCCCATTATATGAACCTTATAGACAGTATACATGCTTTACTCTGCTAAGTC
CCAATAATTTGGGACCATGTGTTAGATATTATTGGAAAGTGTTGCCTCAAGGATGGAAGTTAAGCCCGTC
GGTATATCAGTTTACAATGCAGAAAATATTAAAAGATTGGATAGAGGAACACCCTATGATACAGTTTGGG
ATATATATGGATGATATTTATATAGGAAGTGATCTAGAAATAACAGAGCATAGGAAAATAGTAGAAGAAT
TAGCAAATTATATAGCCCAGTTTGGCTTTATGTTGCCTGAGGATAAAAGGCAAGAAGGGTATCCAGCCAA
GTGGCTAGGATTTGAGTTACATCCTGACAAATGGAGGTTTCAAAAACATACATTAGCAGAGCTAAAAGAA
GGACCAATCACATTAAATAAATTGCAAAAATTAGTAGGGGACTTAGTCTGGAGACAATCATTAATAGGAA
AAAGTATTCCAAATATATTAAAGTTAATGGAGGGAGATAGGGCACTCCAAAGTGAAAGGCAAATAGAAAA
AGTTCATGTACAGGAGTGGGAAACATGTAAGAAAAAATTAGAAGAAATGGAAGGAAGTTATTATAATGAA
GAAAAGGACATTTATGGGCAAATAGATTGGGGAAATAAGGCAATTGAGTATATAGTGTTTCAGGAGAAGG
GGAAACCTTTGTGGGTAAATGTGGTCCATAATATTAAAAACTTAAGTCAACCACAACAAATTATTAAGGC
AGCACAGAAGTTAACGCAGGAAGTGATAATAAGAACAGGGAAAATACCATGGATTTTATTACCAGGAAAA
GAAGAAGATTGGATTTTGGAATTGCAAATAGGGAATATCACATGGATGCCTTCATTTTGGTCATGCTACA
GGGGATCAGTAAGGTGGAAAAGGAGAAATGTAGTAACGGAAATAGTAGAAGGACCAACATATTATACCGA
TGGAGGGAAGAAAAATGGGGTTGGAAATTTAGGCTACATTGCCTCAACAGGGGAAAAATACAGGATACAT
GAGGAAGGGACCAATCAGCAGTTAGAACTAAGGGCAATAGAAGAGGCATGTAAACAGGGACCAAGCAAAA
TGAATATAGTAACTGACAGCAGATATGCATATGAATTTATGCTAAGGAATTGGGATGAAGAAGTAATAAG
AAACCCTATACAGGCCAGAATTATGAAAATAATTCATGGAAAAGAAAAGGTGGGAGTACATTGGGTTCCA
GGACATAAAGGAATTCCTCAAAATGAGGAGGTAGATAAATATATTGCAGAAATATTTCTAGCAAAAGAAG
GAGAAGGGATTCTCCCAAAAAGAGAGGAAGATGCAGGGTATGATTTAATTTGTCCTCAGGAGGTACATAT
TCCGGCGGGACAAGTAAGAAAAATCCCCGTAGACTTAAGATTAAATTTAAAAGAGAATCAATGGGCCATG
ATAGGGACAAAGAGTAGCTTTGCCAGCAAGGGGGTATTTGTTCAAGGAGGAATAATAGATTCAGGATATC
AGGGAACAATACAAGTAGTAATATACAATAGTAATGATGTGGAAGTGGTAATTCCCCAGGGAAGGAAGTT
TGCACAGTTAATTCTGATGCCCTTGATACATGAAGAATTAGAAGCTTGGGGAGAAACAAGGAAAACAGAA
AGGGGAAAACAAGGATTTGGATCTACGGGGGCTTATTGGATAGAGAATATTCCTAAAGCAGAAGAGGATC
ATGATAAGTGGCATCAAGATGCAAATTCATTGCACTTAGAATTTGGGATCCCTAGATCAGCTGCTGAAGA
TATAGTACAACAATGTGAGGTGTGTCAAGAAAATAAGATGCCGAGTACCATCAGAGGAGGGAATAAAAGA

GGAATAGATCATTGGCAGGTAGATTATACACATCATGAAGATAAAATATTATTAGTATGGGTAGAAACAA
ATTCAGGATTGATTTATGCAGAACGGGTAAAAGGGGAAACAGGCCAAGAATTCAGAGTACACGTTATGAA
ATGGTATGCCATGTTTCATCCAAAATCATTGCAGTCTGATAACGGACCTGCCTTTGTAGCAGAGCCCACG
CAGCTCTTAATGAAATATCTAGGAATAGAGCACACTACAGGAGTTCCATGGAACCCACAATCTCAAGCCT
TAGTAGAAAGAACTCATCAAACGTTAAAACATACCCTGGAAAAATTTATCCCCATATTTGCAGCATTCGA
ATCTGCCCTTGCCGCGGCCTTAATAGCACTTAATATAAAAAGAAAGGGTGGGCTAGGGACAAGCCCTATG
GATATATTTATATTTAATAAGGAACAGCAAAGAACACAACAACAATATAATTTAAAGCAGTCAAAAATTC
GATTTTGTTATTACAGGATCAGGAAAAAAGGACATCCAGGCGAGTGGCAAGGGCCAACACAGGTATTGTG
GGAAGGGGAAGGTGCAATAGTAGTAAAAGATAAACCCACAGAAAGATATTTGGTAGTAACCAACAAGGAT
GTCAGGTTCATCCCACCACCAAAAGAAATACAAAAAGAATAA

>MT993897.1 Small ruminant lentivirus isolate USMARC-200303038-1, complete genome
GGGAAACAGCAGGAGGGGGCCACGTGTG
GTGCCGTCCGCGCCCCCTATGTTGTAACAGAAGCACCACCGAAAATAGAAGCAAGAGTGGGGACAACTTG
GAAGGAATTATTAGTGGATACAGGAGCAGATAGAACTATAGTAAGAAAACATGATAGCACAGGAATACCA
AAGGGAAGAATAAAGCTTCAAGGAATAGGAGGAATTATAGAAGGGGAAAAATGGGATCAAGTACAAATAC
AATATAAAGAAAAAATAATAAAAGGAACAATAGTAGTACTGCCAACAAGTCCAGTAGAAGTGTTAGGGAG
AGACAATATGGGAAAATTGGGTATAGAATTAATTATGGCCAATTTAGAAGAAAAGAAAATTCCCATAACG
CAGGTAAAATTGAAAGAGGGATGTAAGGGACCTCAGGTAGCACAGTGGCCTTTAACTCAAGAAAAGTTGG
AAGGGCTAAAAGAAATTATAGATAGATTAGAAAAAGAAGGAAAACTAGGAAGAGCACCACCTCATTGGAC
ATGCAATACTCCAATATTTTGCATTAGGAAAAAATCAGGGAAATGGAGAATGTTAATAGATTTTAGGGAA
TTAAACAAGCAAACAGAGGATTTAGCAGAGGCTCAGCTAGGGTTACCGCATCCGGGGGGATTAAAAAAGA
AGAAAAATGTAACAGTCCTCGATATTGGGGACGCATATTTTACAATCCCATTATATGAACCTTATAGACA
GTATACATGCTTTACTCTGCTAAGTCCCAATAATTTGGGACCCTGTGTTAGATATTATTGGAAAGTATTG
CCTCAAGGATGGAAGTTAAGCCCGTCGGTATATCAGTTTACAATGCAGAAAATATTAAAAGATTGGATAG
AGGAACACCCTATGATACAGTTTGGGATATATATGGATGATATTTATATAGGAAGTGATCTAGAAATAAC
AGAGCATAGGAAAATAGTAGAAGAATTAGCAAATTATATAGCACAGTTTGGCTTTATGGTGCCTGAAGAT
AAAAGGCAAGAAGGGTATCCAGCCAAGTGGCTAGGATTTGAGTTACATCCTGACAAATGGAGGTTTCAAA
AACATACATTAGCAGAGCTAAAAGAAGGACCGATCACATTAAATAAATTGCAAAAATTAGTAGGGGACTT
AGTCTGGAGACAATCATTAATAGGAAAAGGTATTCCAAATATATTAAAGTTAATGGAGGGAGATAGGGCA
CTCCAAAGTGAAAGGCAAATAGAAAAAGTTCATGTACAGGAGTGGGAAACATGTAAGAAAAAATTAGAAG
AAATGGAAGGAAGTTATTATAATGAAGAAAAGGACATTTATGGGCAAATAGATTGGGGAAATAAGGCAAT
TGAGTATATAGTGTTTCAGGAGAAGGGGAAACCTTTGTGGGTAAATGTGGTCCATAATATTAAAAACTTA
AGTCAACCACAACAAATTATTAAGGCAGCACAGAAGTTAACACAGGAAGTGATAATAAGACTAGGGAAAA
TACCATGGATTTTATTACCAGGAAAAGAAGAAGATTGGATTTTGGAATTGCAAATAGGGAATATCACATG
GATGCCTTCATTTTGGTCATGCTACAGGGGATCAGTAAGGTGGAAAAGGAGAAATGTAGTAACGGAAATA
GTAGAAGGACCAACATATTATACCGATGGAGGGAAGAAAAATGGGGTTGGAAATTTAGGCTACATTGCCT
CAACAGGGGAAAAATACAGGATACATGAGGAAGGGACCAATCAGCAATTAGAACTAAGGGCAATAGAAGA
GGCATGTAAACAGGGACCAAGCAAAATGAATATAGTAACTGACAGCAGATATGCATATGAATTTATGCTA
AGGAATTGGGATGAAGAAGTAATAAGAAACCCTATACAGGCCAGAATTATGAAAATAATTCATGGAAAAG
AAAAGGTGGGAGTACATTGGGTTCCCGGACATAAAGGAATTCCTCAAAATGAGGAGGTAGATAAATATAT
TGCAGAAATATTTTTAGCAAAAGAAGGAGAAGGGATTCTCCCAAAAAGGGAGGAAGATGCAGGGTATGAT
TTAATTTGTCCTCAGGAGGTACATATTCCGGCGGGACAGGTAAAAAAAATCCCCGTAGACTTAAGATTAA
ATTTACAAGAGAAGCAATGGGCCATGATAGGGACAAAGAGTAGCTTTGCCAGCAAGGGGGTATTTGTTCA
AGGAGGAATAATAGATTCAGGGTATCAGGGAACAATACAAGTAGTAATATACAATAGTAATGATGTGGAA
GTGGTGATTCCCCAGGGAAGGAAGTTTGCACAGTTAATTCTGATGCCCTTGATACATGAAGAATTAGAAT
CTTGGGGAGAAACAAGGAAAACAGAAAGGGGAAAACAAGGATTTGGATCTACAGGGGCTTATTGGATAGA
GAATATTCCTAAAGCAGAAGAGGATCATGATAAGTGGCATCAAGATGCAAATTCATTGCATTTAGAATTT
GGGATCCCTAGATCAGCTGCTGAAGATATAGTACAACAATGTGAGGTGTGTCAAGAAAAAAAGATGCCAA
GTACCATCAGAGGAGGGAATAAAAGAGGAATAGATCATTGGCAGGTAGATTATACACATCATGAAGATAA
AATATTATTAGTATGGGTAGAAACAAATTCAGGATTAATTTATGCAGAACGGGTAAAAGGGGAAACAGGC
CAAGAATTCAGAGTACACGTTATGAAATGGTATGCCATGTTTCATCCAAAATCATTGCAGTCTGATAATG
GACCTGCCTTTGTAGCAGAGCCAACGCAGCTCTTAATGAAATATCTAGGAATAGAGCACACTACAGGAGT
TCCATGGAACCCACAATCTCAAGCCTTAGTAGAAAGAACTCATCAAACGTTAAAACATACCCTGGAAAAA
TTTATCCCCATATTTGCAGCATTCGAATCTGCCCTTGCCGCGGCCCTAATAGCACTTAATATAAAAAGAA
AGGGTGGGCTAGGGACAAGCCCTATGGATATATTTATATTTAATAAGGAACAGCAAAGAACACAACAACA
ATATAATTTAAATCAGTCAAAAATTCGATTTTGTTATTACAGGATCAGAAAAAAAGGACATCCAGGCGAG
TGGCAAGGGCCAACACAGGTATTGTGGGAAGGGGAAGGTGCAATAGTAGTAAAAGATAAACCCACAGAAA
GATATTTGGTAGTAACCAACAAGGATGTCAGGTTCATCCCACCACCAAAAGAAATACAAAAAGAATAA

>MT993900.1 Small ruminant lentivirus isolate USMARC-200050064-r, complete genome
GGGAAACAGCAGGAGGGG
GCCACGTGTGGTGCCGTCCGCGCCCCCTATGTTGTAACAGAAGCACCACCGAAAATAGAAGCAAGAGTGG
GGACAACTTGGAAGGAATTATTAGTGGATACAGGAGCAGATAGAACTATAGTAAGAAAACATGATAGCAC
AGGAATACCAAAGGGAAGAATAAAGCTTCAAGGAATAGGAGGAATTATAGAAGGGGAAAAATGGGATCAA
GTACAACTACAATATAAAGAAAAAATAATAAAAGGAACAATAGTAGTACTGCCAACAAGTCCAGTAGAAG
TGTTAGGGAGAGACAATATGGGAAAATTGGGTATAGAATTAATTATGGCCAATTTAGAAGAAAAGAAAAT
TCCCATAACGCAGGTAAAATTGAAAGAGGGATGTAAGGGACCTCAGGTAGCACAGTGGCCTTTAACTCAA
GAAAAGTTGGAAGGGCTGAAAGAAATTATAGATAGATTAGAAAAAGAAGGAAAACTAGGAAGAGCACCAC
CTCATTGGACATGCAATACTCCAATATTTTGCATTAGGAAAAAATCAGGGAAATGGAGAATGTTAATAGA
TTTTAGGGAATTAAACAAGCAAACAGAGGATTTAGCAGAGGCTCAGCTAGGGTTACCGCATCCGGGGGA
TTGAAGAAGAAGAAAAATGTAACAATCCTCGATATTGGGGATGCGTATTTTACAATCCCATTATATGAAC
CTTATAGACAGTATACATGCTTTACTCTGCTAAGTCCCAATAATTTGGGACCATGTGTTAGATATTATTG
GAAAGTGTTGCCTCAAGGATGGAAGTTAAGCCCGTCGGTATATCAGTTTACAATGCAGAAAATATTAAAA
GATTGGATAGAGGAACACCCTATGGTACAGTTTGGGATATATATGGATGATATTTATATAGGAAGTGATC
TAGAAATAACAGAGCATAGGAAAATAGTAGAAGGATTAGCAAATTATATAGCACAGTTTGGCTTTATGCT
GCCTGAAGATAAAAGGCAAGAAGGGTATCCAGCCAAGTGGCTGGGATTTGAGTTACATCCTGACAAATGG
AGGTTTCAAAAACATACATTAGCAGAGCTAAAAGAAGGACCGATCACATTAAATAAATTGCAAAAATTAG
TAGGGGACTTAGTCTGGAGACAATCATTAATAGGAAAAGGTATTCCAAATATATTAAAGTTAATGGAGGG
AGATAGGGCACTCCAAAGTGAAAGGCAAATAGAAAAAGTTCATGTACAGGAGTGGGAAACATGTAAGAAA
AAATTAGAAGAAATGGAAGGAAGTTATTATAATGAAGAAAAGGACGTTTATGGGCAAATAGATTGGGGAA
ATAAGGCAATTGAGTATATAGTGTTTCAGGAGAAGGGGAAACCTTTGTGGGTAAATGTGGTCCATAATAT
TAAAAACTTAAGTCAACCACAACAAATTATTAAGGCAGCACAGAAGTTAACACAGGAAGTGATAATAAGA
CTAGGGAAAATACCATGGATTTTATTACCAGGAAAAGAAGAAGATTGGATTTTGGAATTGCAAATAGGGA
ATATCACATGGATGCCTTCGTTTTGGTCATGCTACAGGGGATCAGTAAGGTGGAAAAGGAGAAATGTAGT
AACGGAAATAGTAGAAGGACCAACATATTATACCGATGGAGGGAAGAAAAATGGGGTTGGAAATTTAGGA
TACATTGCCTCAACAGGGGAAAAATACAGGATACATGAGGAAGGGACCAATCAGCAGTTAGAACTAAGGG
CAATAGAAGAGGCATGTAAACAGGGACCAAGCAAAATGAATATAGTAACTGACAGCAGATATGCATATGA
ATTTATGCTAAGGAATTGGGATGAAGAAGTAATAAGAAACCCTATACAGGCCAGAATTATGAAAATAATT
CATGGAAAAGAAAAGGTGGGAGTACATTGGGTTCCAGGACATAAAGGAATTCCTCAAAATGAGGAGGTAG
ATAAATATATTGCAGAAATATTTCTAGCAAAAGAAGGAGAAGGGATTCTCCCAAAAAGGGAGGAAGATGC
AGGGTATGATTTAATTTGTCCTCAGGAGGTACATATTCCGGCGGGACAAGTAAGAAAAATCCCCGTAGAC
TTAAGATTAAATTTACAAGAGAAGCAATGGGCCATGATAGGGACAAAGAGTAGCTTTGCCAGCAAGGGGG
TATTTGTTCAAGGAGGAATAATAGATTCAGGGTATCAAGGAACAATACAAGTAGTAATATACAATAGTAA
TGATGTGGAAGTGGTAATTCCCCAGGGAAGGAAGTTTGCACAGTTAATTCTGATGCCCTTGATACATGAA
GAATTAGAATCTTGGGGAGAAACAAGGAAAACAGAAAGGGGAAAACAAGGATTTGGATCTACGGGGGCTT
ATTGGATAGAGAATATTCCTAAAGCAGAAGAGGATCATGATAAGTGGCATCAAGATGCAAATTCATTGCA
CTTAGACTTTGGGATCCCTAGATCAGCTGCTGAAGATATAGTACAACAATGTGAGGTGTGTCAAGAAAAT
AAGATGCCAAGTACCATCAGAGGAGGGAATAAAAGAGGAATAGATCATTGGCAGGTAGATTATACACATC
ATGAAGATAAAATATTATTAGTATGGGTAGAAACAAATTCAGGATTGATTTATGCAGAACGGGTAAAAGG
GGAAACAGGCCAAGAATTCAGAGTACACGTTATGAAATGGTATGCCATGTTTCATCCAAAATCATTGCAG
TCTGATAACGGACCTGCCTTTGTAGCAGAGCCAACGCAGCTCTTAATGAAATATCTAGGAATAGAGCACA
CTACAGGAGTTCCATGGAACCCACAATCTCAAGCCTTAGTAGAAAGAACTCATCAAACGTTAAAACATAC
CCTGGAAAAATTTATCCCCATATTTGCAGCATTCGAATCTGCCCTTGCCGCGGCCCTAATAGCACTTAAT
ATAAAAAGAAAGGGTGGGCTAGGGACAAGCCCTATGGATATATTTATATTTAATAAGGAACAGCAAAGAA
CACAACAACAATATAATTTAAATCAGTCAAAAATTCGATTTTGTTATTACAGGATCAGGAAAAAAGGACA
TCCAGGCGAGTGGCAAGGGCCAACACAGGTATTGTGGGAAGGGGAAGGTGCAATAGTAGTAAAAGATAAA
CCCACAGAAAGATATTTGGTAGTAACCAACAAGGATGTCAGGTTCATCCCACCACCAAAAGAAATACAAA
AAGAATAA

>MZ313872.1 Visna-maedi virus isolate XM-MDV30, complete genome
ATGCCATCATTGTGGA
AAAAGAGGACATATGCAAAAAGATTGCAGGAAAAAGAAAAGAGAGAACATGAAGCAGCAGGGAAACAGCA
GGAGGGGGCCACGTGTGGTGCCGTCCGCGCCCCCTATGCTGTAACAGAAGCACCACCAATGATAAGAATA
AAAGTAGGGACAAATTGGAAAGAGGTATTAGTAGACACAGGAGCAGACACAGGACTATAGTTAGAAAACATG
ATAATTCAGGGGTACCAAAGGGAAGAATAACATTACAGGGAATAGGGGGAATCATAGAAGGAGAAAAATG
GGATCAAGTGCCGTTGCAATATAAAAATAAAATTATAAAAGGTACAATTGTGGTACTACCAAGTAGTCCA
GTAGAAGTGTTAGGAAGAGATAATATGGGAAAAATAGGAATAGGTCTAGTCATGGCAACTTTAGAAGAAA
GAAAAATCCCCATTACAGAAGTAAGGTTAAAAGAGGGATGTAAAGGACCAAATATAGCACAATGGCCCTT
AACCCAAGAAAAGTTGGAGGGACTAAAAGAAATTGTAGATAGATTGGAGAAGGAAGGGAAACTAGGAAGG

GCACCACCACATTGGACGTGCAATACACCAATATTTTGCATTAAGAAAAAATCAGGGAAATGGAGAATGT
TAATAGATTTTAGGGAATTAAATAAACAAACAGAAGATTTAGCAGAAGCACAATTGGGGCTACCACATCC
AGGGGGATTACAAAAGAAAAAGCATGTAACAGTATTAGATATAGGGGATGCGTACTTCACTATTCCATTA
TATGAGCCATATCGGCAATATACGTGCTTTACATTGCTGAGTCCTAATAATCTAGGACCATGTGTCAGAT
ATTATTGGAAAGTGTTGCCTCAAGGATGGAAGTTGAGTCCATCGGTATATCAGTTTACAATGCAGAAGAT
ATTAAAAGATTGGATAGAGGCGCATCCTATGATACAGTTTGGAATATATATGGATGATATTTATATAGGA
AGTGATCTAGAGATAACAAAGCATAGGAAAATAGTGGAAGAATTAGCAAATTATATAGCACAATTTGGCT
TCATGTTGCCTGAAGATAAAAGGCAGGAAGGGTATCCAGCCAAGTGGCTAGGATTTGAGCTACACCCTGA
CAAATGGAGGTTTCAAAAACATACATTAGCAGAACTAAAAGAGGGAACAATCACATTAAATAAGTTGCAG
AAGTTAGTAGGGGATTTGGTCTGGCGGCAATCATTGATAGGAAAAAGTATTCCAAATATATTAAAGTTAA
TGGAGGGAGATAGGGCACTCCAAAGTGAAAGGCAAATAGAAAAAATTCATGTACAAGAATGGGAAACATG
TAGGAAAAAATTAGCAGAAATGGAAGGAAATTATTATGATGAAGAGAAAGACATTTATGGACAGATAGAT
TGGGGAAATAAAGCAATTGAGTATATAGTGTTTCAAGAGAAAGGGAAACCTTTATGGGTGAATGTAGTCC
ATAATATTAAAAACTTAAGTCAACCACAGCAAATTATTAAAGCAGCCCAGAAATTGACACAGGAAGTGAT
AATAAGAACAGGAAAAATACCATGGATATTATTACCAGGAAAAGAAGAGGATTGGATCTTAGAATTACAA
ATAGGGAATATAACGTGGATGCCATCATTTTGGTCATGTTATAGAGGAGCAGTGAGGTGGAAAAAGAGAA
ATATAGTAACAGAAGTAGTAGAAGGACCAACATACTATACAGATGGAGGGAAGAAAAATGGAATAGGAAA
TTTAGGCTACATTGCCTCGACAGGAGAAAAATATAGGATACATGAAGAAGGGACTAATCAGCAATTAGAA
CTACGGGCAATAGAGGAAGCATGTAAACGGGGACCAAGCAAAATGAATATAGTAACGGATAGCAAGTACG
CATATGAATTTATGCTAAGAAATTGGGATGAAGAGGTAGTAAAAAATCCTATACAGGCCAGAATTATGAA
AATAATTCATGGAAAGGAAAAAGTAGGAGTGCATTGGGTACCAGGACATAGAGGGATCCCTCAAAATGAG
GAAATAGATAAATATATTTCAGAAATATTCCTAGCAAGAGAAGGAGAAGGGATTCTCCCTAAAAGAGAAG
AAGATGCAGGATATGATTTAATTTGTCCTCAAGAGATACATATCCCGGCAGGCCAAGTAAGAAAAATTCC
CATAGATCTAAGGTTAAATTTAAAAAAGAATCAATGGACCATGATAGGAACAAAAAGCAGTTTCGCAAGC
AAGGGAGTATTTGTTCAGGGAGGAATAATAGACTCTGGGTATCAAGGAACAATACAGGTAGTAGTATACA
ATAGCAATGAAATAGAAGTAGTAATACCCCAAGGGAGGAAATTTGCGCAATTAATTCTAATGCCATTGAT
ACATGAAGAATTGGAACATTGGGGGAAAACGAGAAAAACAGAAAGGGGAAAGCAAGGATTTGGATCCACG
GGGGCATATTGGATAGAAAATATCCCTAAAGCAGAAGAGGACCATAATAAATGGCATCAGGATGCAAATT
CATTGCACTTAGACTTTGGGATTCCTAAATCAGCTGCTGAAGATATAGTACAACAATGTGAAGTGTGTCA
GGAAAATAAAATGCCAAGCACCCTCAGAGGAGGAAATAAAAGGGGAATAGACCATTGGCAAGTGGACTAC
ACACATTATGAAGATAAAATATTATTAGTATGGGTAGAAACAAATTCAGGATTAGTTTATGCAGAAAAGG
TAAAAGGAGAAACAGGCCAAGAATTCAGAATACAGGTTATGAAATGGTATGCTATGTTTAATCCAAAATC
ATTGCAGTCTGATAACGGACCTGCCTTTGTAGCGGAGCCAACCCAGCTCTTAATGAAATATCTAGGGATA
GAACACACTACGGGAGTTCCCTGGAACCCACAATCACAAGCTTTGGTTGAAAGAACCCATCAAACGTTAA
AACATACCATAGAAAAATTCATCCCCATGTTTGTAGCATTTGACTCTGCTCTTGCCGCCGCACTCATAGC
TCTAAATATAAAAAGAAAGGGTGGGCTAGGGACAAGCCCTATGGATATATTTATATTTAATAAAGAACAA
CAAAGAATACAGCAACAGTCTCAAGTAAATCAATCAAAAATTCGATTTTGTTATTACAGGATCAGGAAAA
AAGGACATCCAGGAGACTGGCAGGGGCCCACACAGGTATTGTGGGAAGGGGAAGGTGCAATAGTGGTAAA
AGATAAATTCACAGAAAGATATTTTGTGGTAACTAACAAGGATGTCAGGTTCATCCCACCACCAAAAGAA
ATACAAAAGGAATAA

>MT993904.1 Small ruminant lentivirus isolate USMARC-200023230-1, complete genome
GGGAAACAGCAGGAGGGGGCCACGTGT
GGTGCCGTCCGCGCCCCCTATGTTGTAACAGAAGCACCACCGAAAATAAAAGCAAGAGTGGGGACAACTT
GGAAGGAATTATTAGTGGATACAGGAGCAGATAGAACTATAGTAAGAAAACATGATAGCACAGGAATACC
AAAGGGAAGAATAAAGCTTCAGGGAATAGGAGGAATTATAGAAGGGGAAAAATGGGATCAAGTACACATA
CAATATAAAGAAAAAATAATAAAAGGAACAATAGTAGTACTGCCCACAAGTCCAGTAGAAGTGTTAGGGA
GAGACAATATGGGAAAATTGGGTATAGAATTAATTATGGCTAATTTAGAGGAAAAGAAAATTCCCATAAC
ACAGGTAAAATTGAAAGAGGGATGTAAGGGACCTCAGGTAGCACAGTGGCCTTTAACTCAAGAAAAGTTG
GAAGGGCTGAAAGAAATTATAGATAGATTAGAAAAAGAAGGAAAACTAGGAAGAGCACCACCTCACTGGA
CATGCAATACTCCAATATTTTGCATTAGGAAAAAATCAGGGAAATGGAGAATGTTAATAGATTTTAGGGA
ATTAAACAAGCAAACAGAGGATTTAGCAGAGGCTCAGCTAGGGTTACCGCATCCGGGGGGATTGAAGAAG
AAGAAAAATGTAACAGTCCTCGATATTGGGGATGCGTATTTTACAATCCCATTATATGAACCTTATAGAC
AGTATACATGCTTTACTCTGCTAAGTCCCAATAATTTGGGACCATGTGTTAGATATTATTGGAAAGTGTT
GCCTCAAGGATGGAAGTTAAGCCCGTCGGTATATCAGTTTACAATGCAGAAAGTATTAAAAGATTGGATA
GAGGAACACCCTATGATACAGTTTGGGATATATATGGATGATATTTATATAGGAAGTGATCTAGAAATAA
CAGAGCATAGGAAAATAGTAGAAGGATTAGCAAATTATATAGCACAGTTTGGCTTTATGGTGCCTGAAGA
TAAAAGGCAAGAAGGGTATCCAGCCAAGTGGCTAGGATTTGAGTTACATCCTGACAAATGGAGGTTTCAA
AAACATACATTAGCAGAGCTAAAAGAAGGACCGATCACATTAAATAAATTGCAAAAATTAGTAGGGGACT
TAGTCTGGAGACAATCATTAATAGGAAAAAGTATTCCAAATATATTAAAGTTAATGGAGGGAGATAGGGC

ACTCCAAAGTGAAAGGCAAATAGAAAAAGTTCATGTACAGGAGTGGGAAATATGTAAGAAAAAAATTAGAA
GAAATGGAAGGAAGTTATTATAATGAAGAAAAGGACATTTATGGGCAAATAGATTGGGGAAATAAGGCAA
TTGAGTATATAGTGTTTCAGGAGAAGGGGAAACCTTTGTGGGTAAATGTGGTCCATAATATTAAAAACTT
AAGTCAACCACAACAAATTATTAAGGCAGCACAGAAGTTAACACAGGAAGTGATAATAAGACTAGGGAAA
ATACCATGGATTTTGTTACCAGGAAAAGAAGAAGATTGGATGTTGGAATTGCAAATAGGGAATATCACAT
GGATGCCTTCATTTTGGTCATGCTACAGGGGATCAGTAAGGTGGAAAAGGAGAAATGTAGTAACGGAAAT
AGTAGAAGGACCAACATATTATACCGATGGAGGGAAGAAAAATGGGGTTGGAAATTTAGGCTACATTGCC
TCAACAGGGGAAAAATACAGGAGACATGAGGAAGGGACCAATCAGCAGTTAGAACTAAGGGCAATAGAAG
AGGCATGTAAACAGGGACCAAGCAAAATGAATATAGTAACTGACAGCAGATATGCATATGAATTTATGCT
AAGGAATTGGGATGAAGAAGTAATAAGAAACCCTATACAGGCCAGAATTATGAAAATAATTCATGGAAAA
GAAAAGGTGGGAGTACATTGGGTTCCAGGACATAAAGGAATCCCTCAAAATGAGGAGGTAGATAAATATA
TTGCAGAAATATTTCTAGCAAAAGAAGGAGAAGGGATTCTCCCAAAAAGGGAGGAAGATGCAGGGTATGA
TTTAATTTGTCCTCAGGAGGTACATATTCCGGCGGGACAAGTAAGAAAAATCCCCGTAGACTTAAGATTA
AATTTACAAAAGAAGCAATGGGCCATGATAGGGACAAAGAGTAGCTTTGCCAGCAAGGGGGTATTTGTTC
AAGGGGGAATAATAGATTCAGGGTATCAGGGAACAATACAAGTAATAATATACAATAGTAATGATGTGGA
AGTGGTAATTCCCCAGGGAAGGAAGTTTGCACAGTTAATTCTGATGCCCTTGATACATGAAGAATTAGAA
TATTGGGGAGAAACAAGGAAAACAGAAAGGGGAAAACAAGGATTTGGATCTACGGGGGCTTATTGGATAG
AGAATATTCCTAAAGCAGAAGAGGATCATGATAAGTGGCATCAAGATGCAAATTCATTGCACTTGGAATT
TGGGATCCCTAGATCAGCTGCTGAAGATATAGTACAACAATGTGAGGTGTGTCAAGAAAATAAGATGCCA
AGTACCATCAGAGGAGGCAATAAAAGAGGAATAGATCATTGGCAGGTAGATTATACACATCATAAAGATA
AAATATTATTAGTATGGGTAGAAACAAATTCAGGATTGATTTATGCAGAACGGGTAAAAGGGGAAACAGG
CCAAGAATTCAGAGTACACGTTATGAAATGGTATGCCATGTTTCATCCAAAATCATTGCAGTCTGATAAC
GGACCTGCCTTTGTAGCAGAGCCAACGCAGCTCTTAATGAAATATCTAGGAATAGAGCACACTACAGGAG
TTCCATGGAACCCACAATCTCAAGCCTTAGTAGAAAGAACTCATCAAACGTTAAAACATACCCTGGAAAA
ATTTATCCCCATATTTGCAGCATTCGAATCTGCCCTTGCCGCGGCCCTAATAGCACTTAATATAAAAAGA
AAGGGTGGGCTAGGGACAAGCCCTATGGATATATTTATATTTAATAAGGAACAGCAAAGAACACAACAAC
AATATAAGTTAAATCAGTCAAAAATTCGATTTTGTTATTACAGGATCAGAAAAAAAGGACATCCAGGCGA
GTGGCAAGGGCCAACACAGGTATTGTGGGAAGGGGAAGGTGCAATAGTAGTAAAAGATAAACCCACAGAA
AGATATTTGGTAGTAACCAACAAGGATGTCAGGTTCATCCCACCACCAAAAGAAATACAAAAAGAATAA

>MT993909.1 Small ruminant lentivirus isolate USMARC-200312013-r, complete genome
GGGAAACAGCAGGAGGGGGCCACGTGTGG
TGCCGTCCGCGCCCCCTATGTTGTAACAGATGCACCACCGAGAATAGAAGCAAAAGTAGGGACAACTTGG
AAAGAATTATTAGTGGATACAGGAGCAGATAGAACTATAGTAAGAAAACATGATAGCACAGGAATACCAA
AGGGAAGAATAAAGCTTCAAGGAATAGGAGGAATTATAGAAGGGGAAAAATGGGATCAAGTACAAATACA
ATATAAAGAGAAAATAATAAAAGGAACAATAGTAGTACTGCCAACAAGTCCAGTAGAAGTGTTAGGGAGA
GACAATATGGGAAAATTGGGTATAGAATTAATTATGGCCAATTTAGAAGAAAAGAAAATTCCCATAACGC
AGGTAAAATTGAAAGAGGGATGTAAGGGACCTCAGGTAGCACAGTGGCCTTTAACTCAAGAAAAGTTGGA
AGGGCTGAAAGAAATTATAGATAGATTAGAAAAAGAAGGAAAACTAGGAAGAGCACCACCTCATTGGACA
TGCAATACTCCAATATTTTGCATTAGGAAAAAATCAGGGAAATGGAGAATGTTAATAGATTTTAGGGAAT
TAAACAAGCAAACAGAGGATTTAGCAGAGGCTCAGCTAGGGTTACCGCATCCGGGGGGATTGAAGAAGAA
GAAAAATGTAACAATCCTCGATATTGGGGACGCGTATTTTACAATCCCATTATATGAACCTTATAGACAG
TATACATGCTTTACTCTGCTAAGTCCCAATAATTTGGGACCATGTGTTAGATATTATTGGAAAGTGTTGC
CTCAAGGATGGAAGTTAAGCCCGTCGGTATATCAGTTTACAATGCAGAAAATATTAAAAGATTGGATAGA
GGAACACCCTATGATACAGTTTGGGATATATATGGATGATATTTATATAGGAAGTGATCTAGAAATAACA
GAGCATAGGGAAATAGTAGAAGGATTAGCAAATTATATAGCACAATTTGGCTTCATGTTGCCTGAAGATA
AAAGGCAAGAAGGGTATCCAGCCAAGTGGCTAGGATTTGAGTTACATCCTAACAAATGGAGGTTTCAAAA
ACATACGTTAGCAGAGCTAAAAGAAGGACCGATCACATTAAATAAATTGCAAAAATTAGTAGGGGACTTA
GTCTGGAGACAATCATTAATAGGAAAAGGTATTCCAAATATATTAAAGTTAATGGAGGGAGATAGGGCAC
TCCAAAGTGAAAGGCAAATAGAAAAAGTTCATGTAAAGGAGTGGGAAACATGTAAGAAAAAATTAGAAGA
AATGGAAGGAAGTTATTATAATGAAGAAAAGGACATTTATGGGCAAATAGATTGGGGAAATAAGGCAATT
GAGTATATAGTGTTTCAGGAGAAGGGGAAACCTTTGTGGGTAAATGTGGTCCATAATATTAAAAACTTAA
GTCAACCACAGCAAATTATTAAGGCAGCACAGAAGTTAACACAGGAAGTGATAATAAGACTAGGGAAAAT
ACCATGGATTTTATTACCAGGAAAAGAAGAAGATTGGATTTTGGAATTGCAAATAGGGAATATCACATGG
ATGCCTTCATTTTGGTCATGCTACAGGGGATCAGTAAGGTGGAAAAGGAGAAATGTAGTAACGGAAATAG
TAGAAGGACCAACATATTATACCGATGGAGGGAAGAAAAATGGGGTTGGAAATTTAGGCTACATTGCCTC
AACAGGGGAAAAATACAGGATACATGAGGAAGGGACCAATCAACAATTGGAACTAAGGGCAATAGAAGAG
GCATGTAAACAGGGACCAAGCAAAATGAATATAGTAACAGATAGCAGATACGCATATGAATTTATGCTAA
GAAATTGGGATGAAGAAGTAATAAGAAACCCTATACAGGCCAGAATTATGAAAATAATTCATGGAAAGGA
AAAGGTGGGAGTACATTGGGTTCCAGGACATAAAGGAATTCCTCAAAATGAGGAGGTAGATAAATATATT

GCAGAAATATTTCTAGCAAAAGAAGGAGAAGGGATTCTCCCAAAAAGGGAGGAAGATGCAGGGTATGATT
TAATTTGTCCTCAGGAGGTACATATTCCGGCGGGACAAGTAAGAAAAATCCCCGTAGACTTAAGATTAAA
TTTACAAGAGAAGCAATGGGCCATGATAGGGACAAAGAGTAGCTTTGCCAGCAAGGGGGTATTTGTTCAA
GGAGGAATAATAGATTCAGGGTATCAGGGAACAATACAAGTAGTAATATACAATAGTAATGATGTGGAAG
TGGTAATTCCCCAGGGAAGGAAGTTTGCACAGTTAATTCTAATGCCCTTGATACATGAAAAATTAGAATC
TTGGGGAGAAACAAGGAAAACAGAAAGGGGAAAACAAGGATTTGGATCTACGGGGGCTTATTGGATAGAG
AATATTCCTAAAGCAGAAGAGGATCATGATAAGTGGCATCAAGATGCAAATTCATTGCACTTAGAATTTG
GGATCCCTAGATCAGCTGCTGAAGATATAGTACAACAATGTGAGGTGTGTCAAGAAAATAAGATGCCAAG
TACCATCAGAGGAGGGAATAAAAGAGGAATAGATCATTGGCAGGTAGATTATACACATCATGAAGATAAA
ATATTATTAGTATGGGTAGAAACAAATTCAGGATTGATTTACGCAGAACGGGTAAAAGGGGAAACAGGCC
AAGAATTCAGAGTACACGTTATGAAATGGTATGCCATGTTTCATCCAAAATCATTGCAATCTGATAACGG
ACCTGCCTTTGTAGCAGAGCCAACGCAGCTCTTAATGAAATATCTAGGAATAGAGCACACTACAGGAGTT
CCATGGAACCCACAATCTCAAGCCTTAGTAGAAAGAACTCATCAAACGTTAAAACATGCCCTAGAAAAAT
TTATCCCCATATTTGCAGCATTCGAATCTGCCCTTGCCGCGGCCCTAATAGCACTTAATATAAAAAGAAA
GGGTGGGCTAGGGACAAGCCCTATGGATATATTTATATTTAATAAGGAACAGCAAAGAACACAACAACAA
TATAATTTAAATCAGTCAAAAATTCGATTTTGTTATTACAGGATCAGGAAAAAAGGACATCCAGGCGAGT
GGCAAGGGCCAACACAGGTATTGTGGGAAGGGGAAGGTGCAATAGTAGTAAAAGATAAACCCACAGAAAG
ATATTTGGTAGTAACCAACAAGGATGTCAGGTTCATCCCACCACCAAAAGAAATACAAAAAGAATAA

>MT993898.1 Small ruminant lentivirus isolate USMARC-200303332-1, complete genome
GGGAAACAGCAGGAGGGGGCCACGTGTGGTGCCGTCCGCGCCCCCTATG
TTGTAACAGAAGCACCACCGAAAATAGAAGCAAGAGTGGGGACAACTTGGAAGGAATTATTAGTGGATAC
AGGAGCAGATAGAACTATAGTAAGAAAACATGATAGCACAGGAATACCAAAGGGAAGAATAAAGCTTCAA
GGAATAGGAGGAATTATAGAAGGGGAAAAATGGGATCAAGTACAAATACAATATAAAGAAAAAATAATAA
AAGGAACAATAGTAGTACTGCCAACAAGTCCAGTAGAAGTGTTAGGGAGAGACAATATGGGAAAATTGGG
TATAGAATTAATTATGGCCAATTTAGAAGAAAAGAAAATTCCCATGACGCAGGTAAAATTGAAAGAAGGA
TGTAAGGGACCTCAGGTAGCACAGTGGCCTTTAACTCAAGAAAAGTTGGAAGGGCTGAAAGAAATTATAG
ATAGATTAGAAAAAGAAGGAAAACTAGGAAGAGCACCACCTCATTGGACATGCAATACTCCAATATTTTG
CATTAGGAAAAAATCAGGGAAATGGAGAATGTTAATAGATTTTAGGGAATTAAACAAGCAAACAGAGGAT
TTAGCAGAGGCTCAGCTAGGGTTACCGCATCCGGGGGGATTAAAAAAGAAGAAAAATGTAACAGTCCTCG
ATATTGGGGACGCGTATTTTACAATCCCATTATATGAACCTTATAGACAGTATACATGCTTTACTCTGCT
AAGTCCCAATAATTTGGGACCATGTGTTAGATATTATTGGAAAGTGTTGCCTCAAGGATGGAAGTTAAGC
CCGTCGGTATATCAGTTTACAATGCAGAAAATATTAAAAGATTGGATAGAGGAACACCCTATGATACAGT
TTGGGATATATATGGATGATATTTATATAGGAAGTGATCTAGAAATAACAGAGCATAGGAAAATAGTAGA
AGGATTAGCAAATTATATAGCACAGTTTGGCTTTATGGTGCCTGAAGATAAAAGGCAAGAAGGGTATCCA
GCCAAGTGGCTAGGATTTGAGTTACATCCTGACAAATGGAGGTTTCAAAAACATACATTAGCAGAGCTAA
AAGAAGGACCGATCACATTAAATAAATTGCAAAAATTAGTAGGGGACTTAGTCTGGAGACAATCATTAAT
AGGAAAAGGTATTCCAAATATATTAAAGTTAATGGAGGGAGATAGGGCACTCCAAAGTGAAAGGCAAATA
GAAAAAGTTCATGTACAGGAGTGGGAAACATGTAAGAAAAAATTAGAAGAAATGGAAGGAAGTTATTATA
ATGAAGAAAAGGACGTTTATGGGCAAATAGATTGGGGAAATAAGGCAATTGAGTATATAGTGTTTCAGGA
GAAGGGGAAACCTTTGTGGGTAAATGTGGTCCATAATATTAAAAACTTAAGTCAACCACAACAAATTATT
AAGGCGGCACAGAAGTTAACACAGGAAGTGATAATAAGACTAGGGAAAATACCATGGATTTTATTACCAG
GAAAAGAAGAAGATTGGATTTTGGAAGTGCAAATAGGGAATATCACATGGATGCCTTCATTTTGGTCATG
CTACAGGGGATCAGTAAGGTGGAAAAGGAGAAATGTAGTAACGGAAATAGTAGAAGGACCAACATATTAT
ACCGATGGAGGGAAGAAAAATGGGGTTGGAAATTTAGGCTACATTGCCTCAACAGGGGAAAAATACAGGA
TACATGAGGAAGGGACCAATCAGCAGTTAGAACTAAGGGCAATAGAAGAGGCATGTAAACAGGGACCAAG
CAAAATGAATATAGTAACTGACAGCAGATATGCCTATGAATTTATGCTAAGGAATTGGGATGAAGAAGTA
ATAAGAAACCCTATACAGGCCAGAATTATGAAAATAATTCATGGAAAAGAAAAGGTGGGAGTACATTGGG
TTCCAGGACATAAAGGAATTCCTCAAAATGAGGAGGTAGATAGATATATTGCAGAAATATTTTTAGCAAA
AGAAGGAGAAGGGATTCTCCCAAAAAGGGAGGAAGATGCAGGGTATGATTTAATTTGTCCTCAGGAGGTA
CATATTCCGGCGGGACAAGTAAAAAAAATCCCCGTAGACTTAAGATTAAATTTACAAGAGAAGCAATGGG
CCATGATAGGGACAAAGAGTAGCTTTGCCAGCAAGGGGGTATTTGTTCAAGGAGGAATAATAGATTCAGG
GTATCAGGGAACAATACAAGTAGTAATATACAATAGTAATGATGTGGAAGTGGTGATTCCCAAGGGAAGG
AAGTTTGCACAGTTAATTCTGATGCCCTTGATACATGAAGAATTAGAATCTTGGGGAGAAACAAGGAAAA
CAGAAAGGGGAAAACAAGGATTTGGATCTACGGGGGCTTATTGGATAGAGAATATTCCTAAAGCAGAAGA
GGATCATGATAAGTGGCATCAAGATGCAAATTCATTGCATTTAGAATTTGGGATCCCTAGATCAGCTGCT
GAAGATATAGTACAACAATGTGAGGTGTGTCAAGAAAAAAAGATGCCAAGTACCATCAGAGGAGGGAATA
AAAGAGGAATAGATCATTGGCAGGTAGATTATACACATCATGAAGATAAAATATTATTAGTATGGGTAGA
AACAAATTCAGGATTAATTTATGCAGAACGGGTAAAAGGGGAAACAGGCCAAGAATTCAGAGTACACGTT
ATGAAATGGTATGCCCTGTTTCATCCAAAATCATTGCAGTCTGATAACGGACCTGCCTTTGTAGCAGAGC

CAACGCAGCTCTTAATGAAATATCTAGGAATAGAGCACACTACAGGAGTTCCATGGAACCCACAATCTCA
AGCCTTAGTAGAAAGAACTCATCAAACGTTAAAACATACCCTGGAAAAATTTATCCCCATATTTGCAGCA
TTCGAATCTGCCCTTGCCGCGGCCCTAATAGCACTTAATATAAAAGAAAGGGTGGGCTAGGGACAAGCC
CTATGGATATATTTATATTTAATAAGGAACAGCAAAGAACGCAACAACAATATAATTTAAATCAGTCAAA
AATTCGATTTTGTTATTACAGGATCAGAAAAAAAGGACATCCAGGCGAGTGGCAAGGGCCAACAAAGGTA
TTGTGGGAAGGGGAAGGTGCAATAGTAGTAAAAGATAAATCCACAGAAAGATATTTGGTAGTAACCAACA
AGGATGTCAGGTTCATCCCACCACCAAAAGAAATACAAAAAGAATAA

>MT993901.1 Small ruminant lentivirus isolate USMARC-200323455-1, complete genome
GGGAAACAGCAGGAGGGGGCCACGTG
TGGTGCCGTCCGCGCCCCCTATGTTGTAACAGAAGCACCACCGAAAATAGAAGCAAGAGTGGGGACAACT
TGGAAGGAATTATTAGTGGATACAGGAGCAGATAGAACTATAGTAAGAAAACATGATAGCACAGGAATAC
CAAAGGGAAGAATAAAGCTTCAAGGAATAGGAGGAATTATAGAAGGGGAAAAATGGGATCAAGTACAACT
ACAATATAAAGAAAAAATAATAAAAGGAACAATAGTAGTACTGCCAACAAGTCCAGTAGAAGTGTTAGGG
AGAGACAATATGGGAAAATTGGGTATAGAATTAATTATGGCCAATTTAGAAGAAAAGAAAATTCCCATAA
CGCAGGTAAAATTGAAAGAGGGATGTAAGGGACCTCAGGTAGCACAGTGGCCTTTAACTCAAGAAAAGTT
GGAAGGGCTGAAAGAAATTATAGATAGATTAGAAAAAGAAGGAAAACTAGGAAGAGCACCACCTCATTGG
ACATGCAATACTCCAATATTTTGCATTAGGAAAAAATCAGGGAAATGGAGAATGTTAATAGATTTTAGGG
AATTAAACAAGCAAACAGAGGATTTAGCAGAGGCTCAGCTAGGGTTACCGCATCCGGGGGGATTGAAGAA
GAAGAAAAATGTAACAGTCCTCGATATTGGGGACGCGTATTTTACAATCCCATTATATGAACCTTATAGA
CAGTATACATGCTTTACTCTGCTGAGTCCCAATAATTTGGGACCATGTGTTAGATATTATTGGAAAGTGT
TGCCTCAAGGATGGAAGTTAAGCCCGTCGGTATATCAGTTTACAATGCAGAAAATATTAAAAGATTGGAT
AGAGGAACACCCTATGATACAGTTTGGGATATATATGGATGATATTTATATAGGAAGTGATCTAGAAATA
ACAGAGCATAGGAAAATAGTAGAAGGATTAGCAAATTATATCGCACAGTTTGGCTTTATGGTGCCTGAAG
ATAAAAGGCAAGAAGGATATCCAGCCAAGTGGCTAGGATTTGAGTTACATCCTGACAAATGGAGGTTTCA
AAAACATACATTAGCAGAGCTAAAAGAAGGACCGATCACATTAAATAAATTGCAAAAATTAGTAGGGGAC
TTAGTCTGGAGACAATCATTAATAGGAAAAGGTATTCCAAATATATTAAAGTTAATGGAGGGAGATAGGG
CACTCCAAAGTGAAAGGCAAATAGAAAAAGTTCATGTACAGGAGTGGGAAACATGTAAGAAAAAATTAGA
AGAAATGGAAGGAAGTTATTATAATGAAGAAAAGGACATTTATGGGCAAATAGATTGGGGAAATAAGGCA
ATTGAGTATATAGTGTTTCAGGAGAAGGGGAAACCTTTGTGGGTAAATGTGGTCCATAATATTAAAAACT
TAAGTCAACCACAACAAATTATTAAGGCAGCACAGAAGTTAACACAGGAAGTGATAATAAGACTAGGGAA
AATACCATGGATTTTATTACCAGGAAAAGAAGAAGATTGGATTTTGGAATTGCAAATAGGGAATATCACA
TGGATGCCTTCGTTTTGGTCATGCTACAGGGGATCAGTAAGGTGGAAAAGGAGAAATGTAGTAACAGAAA
TAGTAGAAGGACCAACATATTATACCGATGGAGGGAAGAAAAATGGGGTTGGAAATTTAGGATACATTGC
CTCAACAGGGGAAAAATACAGGATACATGAGGAAGGGACCAATCAGCAGTTAGAACTAAGGGCAATAGAA
GAGGCATGTAAACAGGGACCAAGCAAAATGAATATAGTAACTGACAGCAGATATGCATATGAATTTATGC
TAAGGAATTGGGATGAAGAAGTAATAAGAAACCCTATACAGGCCAGAATTATGAAAATAATTCATGGAAA
AGAAAAGGTGGGAGTACATTGGGTTCCAGGACATAAAGGAATTCCTCAAAATGAGGAGGTAGATAAATAT
ATTGCAGAAATATTTCTAGCAAAGGAAGGAGAAGGGATTCTCCCAAAAAGGGAGGAAGATGCTGGGTATG
ATTTAATTTGTCCTCAGGAGGTACATATTCCGGCGGGACAAGTAAGAAAAATCCCCGTAGACTTAAGATT
AAATTTACAAGAGAAGCAATGGGCCATGATAGGAACAAAGAGTAGCTTTGCCAGCAAGGGGGTATTTGTT
CAAGGAGGAATAATAGATTCAGGGTATCAAGGAACAATACAAGTAGTAATATACAATAGTAATGACGTGG
AAGTGGTGATTCCCCAGGGGAGGAAGTTTGCACAGTTAATTCTGATGCCCTTGATACATGAAGAATTAGA
ATCTTGGGGAGAAACAAGGAAAACAGAAAGGGGAAAACAAGGATTTGGATCTACGGGGGCTTATTGGATA
GAGAATATTCCTAAAGCAGAAGAGGATCATGATAAGTGGCATCAAGATGCAAATTCATTGCACTTAGAAT
TTGGGATCCCTAGATCAGCTGCTGAAGATATAGTACAACAATGTGAGGTATGTCAAGAAAATAAGATGCC
AAGTACCATCAGAGGAGGAAATAAAAGAGGAATAGATCATTGGCAGGTAGATTATACACATCATGAAGAT
AAAATATTATTAGTATGGGTAGAAACAAATTCAGGATTGATTTATGCAGAACGGGTAAAAGGGGAAACAG
GCCAAGAATTCAGAGTACACGTTATGAAATGGTATGCCATGTTTCATCCAAAATCATTGCAGTCTGATAA
CGGACCTGCCTTTGTAGCAGAGCCAACGGAGCTCTTAATGAAATATCTAGGAATAGAGCACACTACAGGA
GTTCCATGGAACCCACAATCTCAAGCCTTAGTAGAAAGAACTCATCAAACGTTAAAACATACCCTGGAAA
AATTTATCCCCATATTTGCAGCATTCGAATCTGCCCTTGCCGCGGCCCTAATAGCACTTAATATAAAAAG
AAAGGGTGGGCTAGGGACAAGCCCTATGGATATATTTATATTTAATAAGGAACAGCAAAGAACACAACAA
CAATATAATTTAAATCAGTCAAAAATTCGATTTTGTTATTACAGGATCAGGAAAAAAGGACATCCAGGCG
AGTGGCAAGGGCCAACACAGGTATTGTGGGAAGGGGAAGGTGCAATAGTAGTAAAAGATAGACCCACAGA
AAGATATTTGGTAGTAACCAACAAGGATGTCAGGTTCATCCCACCACCAAAAGAAATACAAAAAGAATAA

>AY101611.1 Visna virus strain 85/34 from USA Gag polyprotein (gag), pol polyprotein (pol), virion infectivity protein (vif), and transcriptional activator (tat) mRNAs, complete cds; and envelope glycoprotein (env) mRNA, partial cds
TTGTGGAAAACCGGGACATTTAGCGAGGCAGTGCCGGCAAGGGATAATATGCCATCATTGTGGAAAGA
GGGGGCATATGCAAAAAGATTGCCGGCAAAAGAAGAACAAGGATATGAAGCAGCAGGGAAACAGCAGGAG
GGGGCCACGTGTGGTGCCGTCCGCGCCCCCTATGTTGTAACAGAAGCACCACCTAAAATAGAAGCAAGAA
TAGGGACGACTTGGAAGGAATTATTAGTGGATACCGGAGCAGATAGAACTATAGTAAGAAAACATGATAA
TACAGGAATACCCAAAGGAAGAATAAAGCTTCAAGGAATAGGAGGGATTATAGAAGGGGAAAAATGGGAT
CAAGTAGAAATACAATATAAAGGAAAATCAATAAGGGGAACATTAGTGGTACTACCAAGTAGTCCAGTAG
AAGTGTTGGGGAGGGATAATATGGGAGAATTGGGCATAGGATTAATTATGGCCAATTTAGAAGAAAAGAA
AATCCCTGTAACGCAGGTAAAATTAAAAGAGGGCTGCAAAGGGCCTCATATAGCTCAGTGGCCGTTAACT
CGAGAAAAATTGGAAGGATTAAAAGAGATTATAGATAGATTAGAGAAGGAAGGGAAACTAGGAAGGGCAC
CACCACATTGGACGTGCAATACCCCAATATTTTGCATTAAGAAAAAATCAGGGAAATGGAGAATGTTAAT
AGATTTTAGGGAATTAAATAAACAAACAGAAGATTTAGCGGAAGCACAGTTAGGGCTACCACATCCAGGG
GGATTACAGAAGAAAAAGCATGTAACAGTATTAGATATAGGGGATGCGTACTTCACTATTCCATTGTATG
AGCCATATCGGCAATATACGTGCTTTACATTACTAAGTCCAAATAACCTAGGGCCATGTGTCAGATATTA
TTGGAGAGTATTGCCGCAGGGATGGAAATTAAGTCCCTCGGTATATCAGTTTACAATGCAGAAAATATTA
AAAGATTGGATAGAGGTGCATCCTATGATACAGTTTGGAATATATATGGATGATATCTACATAGGAAGTG
ACCTAGAGATAGCAGAGCATAGGAAAATAGTAGAAGAATTGGCAAATTACATAGCACAATTTGGCTTCAT
GTTGCCTGAAGATAAAAGGCAAGAGGGGTACCCGGCCAAGTGGCTAGGATTTGAGCTACACCCTGACAAA
TGGAAGTTTCAAAAACATACATTAGCAGACCTGAAAGAAGGGACAATCACCTTGAATAAATTGCAAAAAT
TAGTAGGGGATTTAGTCTGGCGGCAATCATTGATAGGAAAAAGTATTCCAAATATATTAAAGTTAATGGA
GGGAGATAGGGCACTTCAAAGTGAAAGACAAATAGAAAAAATTCATGTGCAGGAGTGGGAGACATGTAAG
AGAAAATTGGCAGAAATGGAAGGAAATTATTATGATGAAGAAAAGGACATCTATGGACAGATAGATTGGG
GAAATAAAGCAATTGAATATATAGTGTTTCAGGAGAAAGGGAAACCTTTATGGGTGAATGTAGTCCATAA
CATTAAAAACTTAAGTCAACCACAACAAATTATTAAAGCAGCACAGAAACTAACACAGGAAGTGATAGTA
AGAACAGGAAAAATACCATGGATACTGCTACCAGGAAAGGAAGAGGATTGGATTTTAGAAGTGCAAATAG
GGAATATAACGTGGATGCCTTCATTTTGGTCATGCTTTAGAGGATCAGTAAGATGGAAAAGGAGAAATGT
AGTAACAGAAGTAGTAGAAGGACCAACATATTATACAGATGGAGGGAAGAAAAATGGGATTGGAAATCTA
GGCTACATAGCTTCAACAGGAGAAAAATATAGGATACATGAGGAAGGAACTAATCAACAATTAGAACTAA
GAGCAATAGAAGAAGCATGTAAACAGGGACCAAGTAAAATGAATATAGTAACAGATAGCAGATACGCCTA
TGAATTTATGCTAAGAAATTGGGATGAAGAAGTAATAAAAAATCCTATACAGGCCAGAATCATGAAAATA
ATTCATGAAAAGGAAAAGGTAGGAGTGCATTGGGTACCAGGACATAGAGGGATCCCTCAAAATGAGGAAA
TAGATCAATATATTTCAGAAATATTCTTAGCAAAAGAAGGAGAAGGGATTCTCCCTAAAAGAGAGGAAGA
TGCAGGGTATGATTTAATTTGTCCGCAGGAGATACATATCCCGGCAGGCCAAGTAAGAAAAATTCCCATA
GATCTGAGGTTGAATTTAAAAAAGAATCAATGGGCCATGATAGGAACAAAAAGCAGTTTTGCAAGCAAGG
GAGTATTTGTTCAGGGAGGAATAATAGACTCTGGATATCAAGGAACAATACAGGTAGTAGTATACAATAG
CAATGAAATGGAAGTAATAATACCACAAGGGAGGAAATTTGCGCAATTAATTATAATGCCCTTGATACAT
GAAGAATTGGAACCTTGGGGAGAAACGAGAAAAACAGAAAGAGGAAAGCAAGGATTTGGATCCACGGGAG
CGTATTGGATAGAAAATATCCCTAAAGCGGAAGAGGATCATAATAAGTGGCATCAGGATGCAAATTCATT
GCACTTAGAATTTGGGATTCCTAGATCAGCGGCTGAGGATATAGTACAACAATGTGAAGTGTGTCAAGAG
AATAAAATGCCAAGTACCCTCAGAGGAGGAAATAAAAGGGGAATAGACCATTGGCAAGTGGACTACACAC
ATTATGAAGATAAAATATTGTTAGTATGGGTAGAAACAAATTCAGGATTAATTTATGCAGAAAAGGTAAA
AGGAGAAACAGGCCAAGAATTTAGAATACAGGTTATGAAATGGTATGCCATGTTTAATCCAAAATCATTG
CAGTCTGATAATGGACCTGCCTTTGTAGCAGAGCCAACGCAGCTCTTAATGAAATATCTAGGGATAGAAC
ATACGACAGGAGTTCCATGGAACCCCCAATCTCAAGCCTTAGTTGAAAGGACCCATCAAACGTTGAAACA
TACCCTAGAGAAATTCATCCCCATGTTTGTAGCATTTGACTCTGCTCTTGCAGCCGCACTCATAACTCTA
AATATAAAAAGAAAGGGTGGGCTAGGGACAAGCCCTATGGATATATTTATATTTAATAAAGAGCAACAAA
GAATACAGCAACAGTCTCACATGAATGAATCAAAAATTCGATTTTGTTATTACAGGGTCAGAAAAAAAGG
ACATCCAGGAGACTGGCAGGGGCCCACACAGGTATTGTGGGAAGGGGAAGGTGCAATAGTGGTAAAAGAT
AAACCCACAGAAAGATATTTTGTAGTAACTAACAAGGATGTCAGGTTCATCCCACCACCAAAAGAAATAC
AAAAAGAATAA

>MT993905.1 Small ruminant lentivirus isolate USMARC-201373037-1, complete genome
GGGAAACAGCAGGAGGGGGCCACGTGTGGTGCCGTCCGCGCCCCCTATGTTGTA
ACAGAAGCACCACCAAAAATAAAAGCAAGAGTGGGGACAACTTGGAAGGAATTATTAGTGGATACAGGAG
CAGATAGAACGATAGTAAGAAAACATGATAGCACAGGAATACCAAAGGGAAGAATAAAGCTTCAGGGGAT
AGGAGGAATTATAGAAGGGGAAAAATGGGATCAAGTACAAATACAATATAACGAAAAAATAATAAAAGGA
ACAATAGTAGTACTGCCCACAAGTCCAGTAGAAGTGTTAGGGAGAGACAATATGGGAAAATTGGGTATAG

AATTAATTATGGCCAATTTAGAGGAAAAGAAAATTCCCATAACACAGGTAAAATTGAAAGAGGGATGTAA
GGGACCTCGGGTAGCACAGTGGCCTTTAACTCAAGAAAAAATTGGAAGGACTGAAAGAAATTATAGATAGA
TTAGAAAAAGAAGGAAAACTAGGAAGAGCACCACCTCACTGGACATGCAATACTCCAATATTTTGCATTA
GGAAAAAATCAGGGAAATGGAGAATGTTAATAGATTTTAGGGAATTAAACAAGCAAACAGAGGATTTAGC
AGAGGCTCAGCTAGGGTTACCGCATCCGGGGGGATTGAAGAAGAAGAAAAATGTAACAGTCCTCGATATT
GGGGATGCGTATTTTACAATCCCATTATATGAACCTTATAGACAGTATACATGCTTTACTCTGCTAAGTC
CCAATAATTTGGGACCATGTGTTAGATATTATTGGAAAGTGTTGCCTCAAGGATGGAAGTTAAGCCCGTC
GGTATATCAGTTTACAATGCAGAAAGTATTAAAAGATTGGATAGAGGAACACCCTATGATACAGTTTGGG
ATATATATGGATGATATTTATATAGGAAGTGATCTAGAAATAACAGAGCATAGGAAAATAGTAGAAGGAT
TAGCAAATTATATAGCACAGTTTGGCTTTATGCTGCCTGAAGATAAAAGGCAAGAAGGGTATCCAGCCAA
GTGGCTAGGATTTGAGTTACATCCTGACAAATGGAGGTTTCAAAAACATACATTAGCAGAGCTAAAAGAA
GGACCGATCACATTAAATAAATTGCAAAAATTAGTAGGGGATTTAGTCTGGAGACAATCATTAATAGGAA
AAAGTATTCCAAATATATTAAAGTTAATGGAGGGAGATAGGGCACTCCAAAGTGAAAGGCAAATAGAAAA
AGTTCATGTACAGGAGTGGGAAACATGTAAGAAAAAATTAGAAGAAATGGAAGGAAGTTATTATAATGAA
GAAAAGGACATTTATGGGCAAATAGATTGGGGAAATAAGGCAATTGAATATATAGTGTTTCAGGAGAAGG
GGAAACCTTTGTGGGTAAATGTGGCCCATAATATTAAAAACTTAAGTCAACCACAACAAATTATTAAGGC
AGCACAGAAGTTAACACAGGAAGTGATAATAAGACTAGGGAAAATACCATGGATTTTGTTACCAGGGAAA
GAAGAAGATTGGATGTTGGAATTGCAAATAGGGAATATCACATGGATGCCTTCATTTTGGTCATGTTACA
GGGGATCAGTAAGGTGGAAAAGGAGGAATGTAGTAACAGAAATAGTAGACGGACCAACATATTATACCGA
TGGAGGGAAGAAAAATGGGGTTGGAAATTTAGGCTACATTGCCTCAACAGGGGAAAAATACAGGAGACAT
GAGGAAGGGACCAATCAGCAGTTAGAACTAAGGGCAATAGAAGAGGCATGTAAACAGGGACCAAGCAAGA
TGAATATAGTAACTGACAGCAGATATGCATATGAATTTATGCTAAGGAATTGGGATGAAGAAGTAATAAG
AAACCCTATACAGGCAAGAATTATGAAAATAATTCATGGAAAAGAAAAGGTGGGAGTACATTGGGTTCCA
GGACATAAAGGAATCCCTCAAAATGAGGAGATAGATAAATATATTGCAGAGATATTCCTAGCAAAAGAAG
GAGAAGGGATTCTCCCAAAAAGGGAGGAAGATGCAGGGTATGATTTAATTTGTCCTCAGGAGGTACATAT
TCCGGCGGGACAGACAAGAAAAAATCCCCGTAGACTTAAGATTAAATTTACAAGAGAAGCAATGGGCCATG
ATAGGGACAAAGAGTAGCTTTGCCAGCAAGGGGGTATTTGTTCAAGGGGGAATAATAGATTCAGGGTATC
AGGGAACAATACAAGTAGTAATATACAATAGTAATGATGTGGAAGTGGTAATTCCTCAGGGAAGGAAGTT
TGCACAGTTAATTCTGATGCCCTTGATACATGAAGAATTAGAAGATTGGGGAGAAATAAGGAAAACAGAA
AGGGGAAAACAAGGATTTGGATCTACGGGGGCTTATTGGATAGAGAATATTCCTAAAGCAGAAGAGGATC
ATGATAAGTGGCATCAAGATGCAAATTCATTGCACTTGGAATTTGGGATCCCTAGATCAGCTGCTGAAGA
TATAGTACAACAATGTGAGGTGTGTCAAGAAAATAAGATGCCAAGTACCAGCAGAGGAGGCAATAAAAGA
GGAATAGATCATTGGCAGGTAGATTATACACATCATAAAGATAAAATATTATTAGTATGGGTAGAAACAA
ATTCAGGATTGATTTATGCAGAACGGGTAAAAGGGGAAACAGGCCAGGAATTCAGAGTACACGTTATGAA
ATGGTATGCCATGTTTCATCCAAAATCATTGCAGTCTGATAACGGACCTGCCTTTGTAGCAGAGCCAACG
CAGCTCTTAATGAAATATCTAGGAATAGAGCACACTACAGGAGTTCCATGGAACCCACAATCTCAAGCCT
TAGTAGAAAGAACTCATCAAACGTTAAAACATACCCTGGAAAAATTTATGCCCATATTCGCAGCATTCGA
ATCTGCCATTGCAGCGGCCCTAATAGCACTTAATATAAAAAGAAAGGGTGGGCTAGGGACAAGCCCTATG
GATATATTTATATTTAATAAGGAACAGCAAAGAGCACAACAACAATATAAGTTAAATCAGTCAAAAATTC
AATTTTGTTATTACAGGATCAGAAAAAAAGGACATCCAGGCGAGTGGCAGGGGCCAACGCAGGTATTGTG
GGAAGGGGAAGGTGCAATAGTAGTAAAAGATAAACCCACAGAAAGATATTTTGTAGTAACCAACAAGGAT
GTCAGGTTCATCCCACCACCAAAAGAAATACAAAAAGAATAA

>MG554409.1 Small ruminant lentivirus isolate SRLV009, complete genome
CAGCAAG
AGGGGGCCACGTGTGGTGCCGTCCGCGCCTCCCATGTTGTAACAGAAGCACCACCAATGATAGATATAAA
AGTAGGGACAAAATGGAAGAAAGTACTAGTAGATACAGGGGCAGATAGAACAATTGTCAGATACCATGAT
AATTCGGGAATACCAAAAGGAAGAATAAAACTACAAGGAATAGGAGGAATTATAGAAGGAGAAAAATGGG
AGAAAGTAAAAATAGAGTATAAAGGACAGGAGGTATGGGGATCAATTGTGGTGCTGCCTAGCAGCCCGGT
AGAGGTATTAGGAAGGGATAATATGAGAGACTTAGGGATTAGTTTGGTAATGGCCAATTTAGAAGAAAAG
AAAATTCCTATCACAAAAGTAAAACTAAAAGAAGGCTGTAAGGGTCCTCATGTCCCACAATGGCCGCTAA
CAGAAGAAAAATTAAAAGGACTAACAGAAATAGTAGAGAAATTAGTAGAAGAGGGGAAATTAGGAAAGGC
ACCTCCRCATTGGACATGCAACACTCCTATCTTTGCTATAAAAAAGAGATCAGGGAAATGGAGAATGTTA
ATAGACTTTAGAGAATTGAACAAACAAACAGAAGATTTAACGGAGGCACAATTAGGATTACCACACCCAG
GGGGGTTGCAGAAAAGGAAGAATGTAACTATATTGGATATAGGAGATGCATATTTTACTATACCATTATA
TGAGCCATATCGGGAATATACATGTTTTACTCTATTAAGTCCAAATAATCTAGGACCATGTAGAAGATTC
TATTGGAAAGTCCTCCCACAGGGTTGGAAATTGAGTCCTTCTGTATATCAATTTACAATGCAAGAGATAC
TGAGGGAATGGATAAAAGAACATCCGATGATACAATTTGGGATATATATGGATGATATATATATAGGGAG
TGATTTAGAAATAGGACAGCATAGAAGTATAGTAGAAGAATTGGCTAGCTACATTGCACAATATGGATTT
ATGCTGCCGGAAGAAAAGAGGCAAGAGGGATATCCGGCTAAATGGCTTGGATTCGAACTGCACCCAGAAA
AATGGAAATTTCAGAAGCATACATTGCCAGAAATTAAAGAGGGACCGATAACTCTAAATAAATTGCAGAA

ATTAGTAGGAGATTTAGTGTGGCGACAGTCTTTGATAGGAAAAAGCATACCAAATATATTGAAATTAATG
GAGGGAGATAGAGCACTCCAAAGTGAAAGACAAATAGAAGGAATTCATGTGCAGGAATGGGAGGCATGTA
AGAGAAAATTAGCAGAAATGGAGGGAAGTTATTATGATGAAGACAAGGATGTCTATGGACAGATAGATTG
GGGAAATAAAGCTATAGAGTATATAGTTTTCCAGGAAAAAGGGAAACCATTATGGGTAAATGTAATACAT
AGTATTAAGAACTTAAGTCAGGCACAGCAAATTATTAAAGCAGCTCAAAAGCTTACACAAGAAGTGATAA
TAAGGACAGGGAAGATACCATGGATACTGTTACCAGGAAAGGAAGAAGATTGGATATTAGAACTGCAAAC
AGGGAATATAACATGGATGCCATCATTTTGGTCGTGTTACAGAGGGTCAGTACGCTGGAAAAGGAGAAAC
ATAGTAACAGAAGTAGTAGCAGGACCAACATACTATACTGATGGGGGAAAGAAAAATAATGTAGGAAGCC
TAGGCTATATAGCCTCAACAGGAGAAAGGTATCGAAAACATGAACAGGGAACAAATCAACAGTTGGAGTT
AAGAGCAATAGAGGAAGCATGTAAACATGGGCCTAGTAAAATGAACATAGTAACAGATAGCAGGTATGCA
TTTGAGTTCATGCTTAGGAATTGGGACGAGGAGGTCATACAGAATCCTATACAAGCAAGAATTATGGAAA
TAGTTCATAGAAAAGAAAAAGTAGGAGTACATTGGGTGCCTGGACATAAAGGGATTCCTCAGAATGAAGA
AATTGACAGATATATTTCAGAAATATTTTTAGCAAAGGAGGGAGAGGGAATCCTTCCCAAAAGAAAAGAG
GATGCTGGGTATGACTTAGTATGTCCACAAGAAGTGAGTATCCCAGCAGGACAAGTGAGAAAGATACCAA
TTGATCTTCGATTAAATTTAAAAGAAAATCAATGGGCTATGATAGGAACAAAGAGCAGCTTTGCAAATAA
AGGAGTATTTGTGCAGGGAGGAATAATAGATTCAGGATATCAGGGACAAATACAAGTAGTGATCTATAAC
AGCAATGAAAAAGAGGTAATCATACCGCAAGGAAGAAAATTTGCACAATTGATTCTAATGCCTTTAGTGC
ATGAAGAATTGGAAGCCTGGGGAGAGACAAGGAGAACGCAAAGAGGAAAACAAGGATTTGGGTCCACAGG
GGCATATTGGATAGAAAATATTCCACTAGCAGAAGAAGATCATAATAAATGGCATCAAGATGCTCAGTCA
CTACACTTGGAATTTGGAATTCCAAGAACGGCTGCGGAAGATATAATACAACAATGTGACATATGTAAAG
AAAACAAAATGCCTAGCACAAACAGAGGGACTAATAAAAGAGGAATAAACCATTGGCAGGTAGATTATAC
TCATTTTGAAGATAAAATAATATTGGTATGGGTAGAAACCAATTCGGGGCTAATTTATGCAGAAAGAGTA
AAAGGAGAAACAGGACAAGAATTTCGGATGTATGCCATAAAATGGTATAGCTTGTTTGCCCCAGAATCAT
TGCAATCTGATAATGGACCTGCATTTGTGGCAGAGCCTACGCAACTGCTAATGAAATATTTAGGGGTAGA
ACATAATACAGGGATACCGTGGAACCCGCAGTCGCAAGCTTTAGTAGAAAGAGCTCATCAAACATTAAAA
CATACCTTGGAAAAACTGCTCCCTATGTTTACAGCATTTGAATCCGCTTTAGCAGCAGCACTTATAGCTC
TAAATATAAAAAGAAAGGGTGGCCTAGGGACAAGCCCTATGGATATATTTATCTTTAATAAAGAACAGCA
AAGAATACAACAACAATCTAATATAAATCAGTCAAAAATTCGATTTTGTTATTACAGGGTCAGGAAAAGA
GGACATCCAGGGGATTGGCAAGGACCAGCACAGGTACTTTGGGAAGGGGAAGGTGCTATAGTAGTAAAAG
ATAAAGCAACGGAAAGATATTTAGTAATAGCTAATAAGGATGTTAAGTTCATACCACCCCCAAAAGAAAT
ACAGGAAAAATAA

>MH374291.1 Small ruminant lentivirus isolate SRLV_VdA, complete
genome
CCTCAAGAGGGGGCAACGTG
TGGTGCCGTCCGCACCCCCTATGCTGTAACAGACGCACCACCAAACATAAAAGTACAAGTAGGAACATGT
TGGAAAGAATTATTGATAGATACAGGGGCAGATAGAACAATAGTAAAATACCATGATAGTACAGGCATAC
CTAAAGGGAGGATAAAGTTACAGGGAATAGGAGGAATCATAGAAGGAGAAAAATGGGACAAAGTAAAAAT
GAAGTATAAAGGAAGGGAAATAGAAGGAACAATAGTAGTATTGATTAGTAGTCCAGTAGAAGTGTTAGGA
AGGGATAACATGGGAAAGTTAGGAATCGGGATAATAATGGCCAATTTAGAAGAAGGAAAAATTCCTATAA
CAGAAGTAAGGTTGAAGGAAGGATGTAAAGGACCCCATATACCACAATGGCCATTAACACAAGAAAAATT
GGAAGGACTAAGAGAAATAGTAGAAAGATTAGAAAAAGAAGGAAAGGTAGCGAAAGCTCCTCCAAAGTGG
CCGTGGAATACCCCGGTATTCTGCATAAAAAAGAAATCAGGAAAGTGGAGAATGCTAATAGATTTCAGAG
AATTGAACAAGCAGACAGAAGATCTAGCAGAGGCACAGTTAGGACTACCGCATCCCGGAGGGCTACGAAG
GAAAAAGAATATCACAGTATTGGATATAGGGGATGCGTATTTTACAATTCCATTGTATGGGCCATATAGA
AAGTATACATGTTTTACATTGTTAAGTCCCAATAATTTGGGACCATGTGTAAGGTATTATTGGAGAGTAC
TGCCTCAGGGATGGAAATTAAGTCCATCGGTGTATCAATTCACAATGCAAAAAATATTAGGAGATTGGAT
AAAAGCTCACCCTGAGATACAATTTGGAATTTATATGGATGATATATATATAGGAAGTGATTTAGACATA
AAAGAACATAGGGAGAAAGTAGAGGAATTGGCACAGTATATAGCGCAATATGGATTCATGTTGCCAGAAG
AGAAAAGGCAAGAAGGGTATCCAGCAAAATGGCTAGGATTTGAGTTGCATCCCGAGAAATGGAAATTTCA
GAAACATACCCTTCCGGAGCTTAAAGAAGGAACAATAACTTTAAATAAGTTACAAAAATTGGTAGGAGAT
TTAGTATGGCGACAGTCATTAATAGGGAAAAGTATACCAGAAATATTGAAATTAATGGAAGGGGATAGAG
CATTACAGAGTGAGAGAAGAATAGAAAGGAAACATGTACTAGAATGGGAAGGATGTAGAAAAAAGTTAGA
AGAAATGGAAGGAAATTATTATAATGAGGAGAAAGATGTTTATGGACAAATAGACTGGGGGAGTAAAGCA
ATAGAATACATAGTGTTTCAGGAGAAAGGGAAACCGTTATGGGTAAATGTGGTGCACAATATAAAGAATC
TAAGTCAAGCACAACAAATTATTAAAGCAGCACAAAAATTAACGCAAGAAGTAATAGTTAGAACAGGAAA
AATACCATGGATTTTATTGCCAGGAAAAGAGGAGGATTGGATCTTAGAATTACAAATAGGCAACATAACA
TGGATGCCCTCATTTTGGTCGTGTTATAGAGGATCAGTAAGGTGGAAGAAAAGAAATATAGTAGAAGAAG
TAGTCCCAGGACCAACATATTATACCGATGGAGGAAAGAAAAATGGAGCAGGGAGCTTAGGGTATATAGC
CTCTACAGGACAAAAATATAGAGCCCATGAGGAAGGGACAAATCAGCAATTAGAATTAAGAGCAATAGAA
GAAGCATGTAAACAGGGACCAGCAGAAATGAATATAGTAACAGATAGTAGATACGCATATGAGTTTATGC

TGAGAAATTGGGATGAAGAGGTAATAAAAAATCCTATACAGGCAAGGATCATGCAATTAGTGCATCAGAA
AAAGAGAATAGGAATACACTGGGTACCAGGACACAAAGGTATCCCTCAAAATGAAGAAATAGATAGGTAC
ATATCAGAAATATTTTTAGCAGTAGAAGGAACAGGGATCTGTACAAAAAGAAAGGAAGATGCGGGGTATG
ACTTGATATGTCCACAGGAAGTAAGCATACCCCCAGGACAAGTAAAAAGAATCCCTATAGATTTAAAGGT
AAATCTGAAAGAAGATCAATGGGCAATGATAGGGACAAAAAGTAGTTTTGCCAGCAAAGGAGTCTTTGTC
CAAGGAGGGATAATAGACTCAGGGTATCAAGGAACAATACAGGTAATAGTGTATAATAGTAATGATAGGG
AGGTAGTCATAACTCAGGGAAGAAAATTTGCACAGTTAATCCTCATGCCGTTGCAGCATGAAGAATTGGA
ACCATGGGGAAAAAGGAGAAAAACAGAAAGGGGGGAACAAGGATTTGGGTCTACGGGAATGTATTGGATA
GAGAACATTCCTGCAGCAGAAGAGGAACATGAAAAGTGGCACCAAGACGCGATGTCACTGCAATTAGAAT
TTGGCATCCCTAGGACAGCAGCAGAAGATATAATACAACAATGTGAAGTCTGCCAAGAGAATAAATTGCC
AAGTACGCTAAGAGGAGGAAATAGAAGGGGGGTAGATCATTGGCAAGTAGATTATACTCATTATGAGAAC
AAAATCATATTAGTATGGGTAGAAACAAATTCAGGATTAATATATGCAGAAAAAGTGAAAGGAGAAACAG
GAGAAGAATTCAGAACACAAGTATTAAAATGGTATGCTTTATTTAAACCAGCATCAGTGCAGTCTGATAA
TGGACCTGCATTTGTAGCAGACTCAACACAATTGTTAATGAAATACTTAGGGATTCAACATACAACAGGC
ATCCCATGGAATCCACAATCACAAGCATTAGTGGAAAGAACACATCAAACATTAAAACAAATGTTGAAAA
AACTAGAGGGAAACTTTGTAGCATTTGAATCTGCCCTAGCAGCCGCCCTAATAGCACTTAATATAAAAAG
AAAGGGTGGGCTAGGGACAAGCCCTATGGATATATTTATATTTAATAAAGAACAACAGAGATTATTAGTA
CAAGATAAAGTAAATAAAGAAAAAATTCGATTTTGTTATTACAGGATCAGAAAAAAAGGACACCCAGGCG
AGTGGCTGGGGCCAACACAGGTACTCTGGGAAGGGGAAGGAGCAATTGTAGTTAAGAATAAAGAGCAAGA
AAGATATTTGGTGATAGCATATAAAGATGTTAAGTTTATACCGGCACCAAAAGAATTGCAAAAAGAGTAA

>MW248464.1 Visna-maedi virus strain NM1111, complete genome
ATGCCATCATTGTGGAAAAAGGGGGCACATGCAGA
AAGACTGTCGGCAAAAGAAACAAAAAGATATAAAGCAGCAGGGAAACAGCAGGAGGGGGCCACGTGTGGT
GCCGTCCGCACCCCCTATGTTATAACAGACGCACCACCTAGAATAAAAGTAAAAGTCGGAACAACTTGGA
AAGAATTATTAGTGGACACGGGAGCAGATAGAACCATAGTGAGAAAACATGATAGTTTAGGGATACCAAA
GGGGAGAATAAAGCTTCAAGGAATAGGGGGAATTATAGAAGGAGAAAAATGGGATCAAGTACCAATACAA
TATAGAGAGAAGGTAATAGAGGGAACAATAGTAGTACTACCTAGCAGTCCGGTAGAAGTAATAGGGAGAG
ATAATATGGGGGAGTTAGGGATAAGCTTAATTATGGCAAACCTAGAAGAAAAGAAAATTCCTATTACAGA
AGTAAAATTAAAAGAGGGATGTAAAGGACCTCATATAGCCCAGTGGCCACTAACGCAAGAAAAATTAGAG
GGATTAAAAGAAATAGTAGATAGGCTGGAAAAAGAAGGAAAGTTGGGGAGAGCTCCACCACATTGGACAT
GTAATACCCCTATATTTTGTATTAAGAAAAAGTCAGGAAAATGGAGAATGTTAATAGATTTTAGAGAATT
AAATAAACAAACTGAAGATTTGGCAGAAGCACAACTAGGATTGCCACATCCAGGTGGGTTACAAAGGAAG
AAGCATGTAACGGTATTGGATATAGGGGATGCATATTTCACTATTCCATTATATGAGCCATATCAAAAAT
ATACATGCTTTACTCTATTAAGCCCTAATAATTTAGGGCCATGTGAAAGGTACTATTGGAAAGTATTGCC
GCAAGGATGGAAATTGAGTCCCTCAGTATATCAATTTACAATGCAGAAAATATTAAGGGAATGGATAAAG
GAACATCCTATGATACAATTTGGAATATATATGGATGATATTTACATAGGAAGTGATCTGTCAATAGAGG
AACATAGAGGTATTGTAGAGGAGTTAGCCAGCTTCATAGCAGTATATGGATTTATGTTGCCAGAAGAGAA
AAGGCAAGAAGGATACCCTGCCAAGTGGCTGGGGTTTGAATTACATCCAAATAAGTGGAGATTCCAAAAA
CATACCTTACCAGAACTTAGGGAAGGACCTATCACTCTAAATAAATTACAAAAATTAGTCGGGGATCTGG
TGTGGAGGCAATCCTTAATAGGAAAGAGTATTCCAAATATATTAAAATTAATGGAAGGAGATAGAGCACT
ACAAAGTGAAAGGCAGATAGAACAAATTCATATACAGGAATGGGACAAATGTAAGAAGAGATTAGAAGAG
ATGGAAGGAAATTATTATGATGAAAGTAGGGATATTTATGGACAAATAGACTGGGGAAATAAAGCAATAG
AGTATATAGTCTTCCAAGAGAAAGGAAAGCCGTTATGGGTCAATGTGATACATAGCATAAAAAATTTGAG
TCTAACACAACAAGTTATCAAAGCAGCGCAGAAAGTAACCCAAGAAGTAATAGTTAGAACAGGGAAAATA
CCATGGATACTATTACCAGGAAAAGAAGAAGATTGGATACTAGAGCTACAGGCAGGGAACATAACGTGGA
TGCCCTCATTTTGGTCATGCTATAGAGGATCGGTGAGATGGAAACAGAGAAATATAGTGACAGCAGTAGT
AGAGGGGCCAACGTATTATACGGATGGAGGAAAGAAAAATGGAATAGGAAGCCTGGGATATATTGCATCC
ACCGGAGAGAAATATAGAAAACATGAGCAAGGCACTAATCAGCAATTAGAATTGAGAGCAATAGAAGAGG
CGTGTAAACACGGGCCAGCAAAAATGAATATAGTAACGGATAGCAGATATGCATTTGAATTTATAAACAG
GAATTGGGATGAGGAAGTAATAAAAAATCCAATACAAACAAGAATTATGAAATTAATTCATGAAAAAGAA
AAAGTAGGAATACATTGGGTACCTGGACATAAAGGAATCCCTCAAAATGAAGAAATAGATAAATATATCT
CCGAAGTATTTCTCGCAAAACAAGGGGAAGGAATTATCTCAAAAAGGGCAGAAGATGCGGGGTATGATCT
AATCTGTCCGCTGGATGTAAGCATACCACCTGGAGAAGTGAGAAAGATTCCAATTGACTTAAGAATAAAC
TTAAAAAAGAATCAATGGGCTATGGTAGCAACAAAAAGTAGTTTTGCAAGTAAGGGAGTTTTTGTACAAG
GAGGCATAATAGATTCGGGGTATCAGGGGACAATACAGGTAGTAATATTTAACAGTAATAAGGTAGAAGT
AATAATACCGCAAGGGAGAAAGTTTGCACAATTAATCCTTATGCCTTTAATACATGAAGAACTAGAACCA
TGGGGAAAAAAAAAAAAAAAAGGAAAGGGGAAATCAGGGTTTTGGCTCAACAGGGGCATATTGGATAGAAA
ATATCCCAAAAGCAGAAGAAGAGCACGATAAATGGCATCAAGATGCACTTACACTACAGTTAGAACATGG
AATTCCTAGAAGTGCAGCAGAAGATATAGTAAGACAATGTGAGGTCTGTCAAGAAAGTAAAATGCCAAGT
ACTCTCAGAGGAGGAAATAAGAGAGGGATAGATCATTGGCAGGTAGACTATACTCATTATGAGGATAAAA

TTATATTAGTATGGGTAGAAACAAATTCAGGATTAATGTATGCAGAAAAGGTAAAAGGAGAAACAGGGCA
AGAATTTAGGATACAGGCAATGAGATGGTTTGCGATGTTTCACCCCCGGTCTTTGCAATCAGATAATGGA
CCTGCCTTTGTGGCGGAACCTACGCAGCTCTTAATGAAGTATTTGGGGGTAGACCATACAACAGGGGTAC
CATGGAATCCTCAATCGCAGGCATTAGTAGAAAGAGCACATCAAACACTAAAACATACGTTAGAAAAATT
TGTCCCCACGTTTGTGGCGTTTGAATCTGCCCTTGCCGCAGCCCTCATAGCACTAAATATAAAAAAAAAG
GGTGGGCTAGGGACAAGCCCTATGGATATATTTATATTTAATAAAGAACAGCAAAGAATACAACAACAAC
ATAATTTAAATCAACAAAAAATTCGATTTTGCTATTACAGGGTCAGAAAAAGAGGACACCCAAGCGAGTG
GCAAGGACCAGAACAGGTACTTTGGGAAGGGGAAGGAGCCATAGTAGTTAGGGATAAATCATTAGACAAG
TATTTAGTAATACCCTACAAGGATGTTAAATTTATACCGCAGCCAAAAGAAATACAAAAAGTATAA

>MG554406.1 Small ruminant lentivirus isolate SRLV005, complete genome
CAGCAAGAGGGGGCCACGTGTGGTGCCGTCC
GCACCCCCTATGCTGTAACAGACGCACCACCAAAAGTAAAAGTACAAGTAGGGACATGCTGGAAAGAATT
ATTAATAGATACAGGGGCTGATAGAACAATAGTAAAATACCATGATAGTACGGGCGTACCTGAAGGGAGA
ATAAAGTTACAGGGAATAGGAGGAATCATAGAAGGGGAAAAATGGAAACAAGTAAAGATTAAGTATAGAG
GAGAGGAAATTGAGGGAACAATAGTAGTATTAGCTAGTAGCCCAGTAGAAGTGTTAGGAAGGGATAATAT
GGGGAAGTTAGGAATAGGGATCATAATGGCAAATTTAGAAGAGGGGAAAATTCCAGTGACAGAAGTAAAA
TTGAAGGAAGGATGCAAAGGACCTCATATACCGCAATGGCCATTAACACAGGAGAAATTGGAGGGATTAA
GAGAAATAGTAGAGAGATTAGAAAAGGAAGGAAAAGTAGCGCAGGCTCCTCCACATTGGCCATGGAATAC
CCCTATATTTTGTATTAAGAAAAAGTCAGGAAAATGGAGGATGTTAATAGATTTTAGGGAGTTAAATAAA
CAGACAGAAGATCTAGCAGAGGCACAATTGGGATTACCACATCCCGGAGGACTGCGAAGGAAAAAGAATA
TCACTGTATTAGATATAGGGGATGCATATTTCACAATTCCATTATATGAGCCATATAGACAGTATACATG
TTTTACATTGTTAAGTCCTAATAATATGGGACCATGTAAGAGATATTATTGGAAAGTATTGCCTCAGGGA
TGGAAATTAAGTCCATCTGTGTATCAATTTACAATGCAAAAAATATTATGGGATTGGATAAAAGCTCACC
CTGAGGTACAATTTGGAATCTATATGGACGATATCTATATAGGAAGTGATCTAGAAATCAAAGAGCATAG
AGAAAAGGTAGAAGAATTAGCACAGTATATAGCACAATATGGGTTTATGTTGCCAGAAGATAAAAGACAA
GAAGGGTATCCGGCCAAGTGGCTGGGATTTGAGTTGCACCCGGAGAAATGGAAGTTTCAGAAGCACACCC
TCCCGGAGCTTAAAGAAGGAACCATAACTTTAAATAAGTTACAGAAATTGGTAGGAGATTTAGTATGGCG
ACAGTCATTAATAGGAAAAAGTATACCAGAAATATTGAAATTAATGGAAGGGGATAGAGAATTACAAAGT
GAGAGAAAAATAGAGAGAAAACATGTAGTAGAATGGGAGGAATGTAGAAAAAAGTTAGAGGAAATGGAAG
GAAATTACTATAATGAGGAAAAAGATGTTTATGGGCAAATAGACTGGGGAAATAAAGCAATAGAATACAT
AGTGTTTCAGGAGAAAGGGAAACCTTTATGGGTGAATGTGATGCACAATATAAAGAATTTAAGTCAGGCA
CAACAAATTATTAAAGCAGCACAGAAGTTAACACAAGAAGTAATAATTAGAACAGGAAAAATACCATGGA
TTTTGTTACCAGGAAAAGAAGAGGATTGGATCTTAGAGCTTCAAATAGGAAACATAACCTGGATGCCCTC
ATTTTGGTCATGTTATCGAGGGTCAGTAAGATGGAAGAGAAGAAATGTAGTAGAAGAAGTAGTCCCAGGG
CCAACATATTATACAGATGGAGGAAAGAAAAATGGAATGGGAAGTTTAGGATATATAGCGTCTACAGGAC
AAAAATTTAGAGCCCATGAGGAAGGGACAAATCAGCAATTAGAATTAAGAGCAATAGAAGAAGCATGTAA
ACAGGGACCAGCAGAAATGAATATAGTAACAGATAGTAGGTACGCATATGAATTTATGTTGAGGAATTGG
GATGAGGAGGTAATAAAGAATCCCATACAGGCAAGAATTATGCAGTTGGTACATCAGAAAGTCAAGATAG
GAATACACTGGGTGCCAGGACATAAAGGGATCCCTCAAAATGAGGAAATAGATAGATATATATCAGAAGT
ATTTTTGGCAGTACAAGGAACAGGGATATGTCAAAAAAGAAAGGAAGATGCAGGGTATGACTTGATATGC
CCACAGGAAGTAAGCATACCCCCTGGACAGGTGAAGAGAATCCCTATAGATTTAAAAGTAAATCTGAAAG
AAGACCAATGGGCAATGATAGGAACAAAAAGTAGTTTCGCCAGTAAGGGTGTATTTGTACAAGGAGGAAT
AATAGATTCAGGATATCAAGGGACAATACAGGTGATAGTGTACAACAGTAATGATAAGGAGGTAGTAATA
ACTCAGGGAAGAAAATTTGCACAATTAATTCTCATGCCATTGCAGCATGAGGAATTAGAACCATGGGGAA
AAATAAGAAAGACAGAAAGGGGAGAAGCAGGATTTGGGTCCACGGGAATGTATTGGATAGAAAACATTCC
TGCGGCAGAAGAGGAACATGAGAAATGGCATCAGGACGCGATGTCACTGCAATTAGAATTTGGCATTCCT
AGAACAGCCGCAGAAGATATAATACAACAATGTGAAGTCTGCCAAGAGAATAAAATGCCAAGTACTATGA
GGGGAAGTAATAAAAGGGGGGTAGATCATTGGCAAGTAGATTATACTCATTATGAGAATAAAATCATATT
AGTATGGGTAGAGACAAATTCAGGCTTAATATATGCAGAGAGAGTAAAAGGAGAAACAGGAGAAGAGTTC
AGAACACAAGCGTTGAAATGGTATGCCTTGTTTAAGCCCACATCAGTGCAGTCTGATAATGGACCAGCAT
TTGTTGCAGAGGCAACACAATTACTGATGAAGTACTTAGGGATTCAACATACAACAGGCATCCCATGGAA
TCCCCAATCACAAGCATTGGTGGAAAGGACACATCAAACATTAAAGCAAATGTTGCAAAAATTAGAGGGA
AACTTTGTAGCATTTGAATCTGCCCTAGCAGCTACCCTAATAGCTCTTAATATAAAAAGAAAGGGTGGGC
TAGGGGCAAGCCCTATGGATATATTTATATTTAATAAAGAACAACAGAGATTATTATTACAAAAGCATGA
AAATAAAGAAAAAAATTCGATTTTGTTATTACAGGATCAGAAAGAAAGGACACCCAGGCGAGTGGCTGGGG
CCAACACAGGTACTTTGGGAAGGGGAAGGAGCTATTGTAGTTAAAAATAAGGAACAAGGCAGATATTTAG
TGATAGCATATAAGGATGTCAGGTTTATACCCGCGCCAAAAGAATTGGGAAAAGCGTAA

>MH374284.1 Small ruminant lentivirus isolate SRLV025, complete genome
CATCAAGAGGGGGCAACGTGTGGTGCCGTCCGCACCCCCTATGCTGTAACAAACGCACCACCAAC

AGCAGAAGTACAAGTAGGGACATGTTGGAAAAAATTATTAATAGATACAGGGGCTGATAGAACAATAGTG
AAATACCATGATAGTACAGGCATACCTAAAGGGAGGATAATGTTACAGGGAATAGGAGGAATCATAGAAG
GAGAAAAATGGGACAAAGTAAAAATTAGGTATAAAGGAAAAGAAATAGAGGGAACAATAGTAGTATTGGC
GAGTAGCCCAGTAGAGGTATTAGGAAGAGACAACATGGGAAAACTAGGAATCGGGATAATAATGGCCAAT
TTGGAAGAAGGAAAAATTCCTGTGACTGAAGTAAGGTTAAAAGAAGGATGTAAAGGACCTCATATACCAC
AATGGCCATTAACACAGGAAAAACTAGAAGGGTTAAGAGAAATAGTAGAGAGATTAGAGAAAGAAGGAAA
AGTAGCGAAAGCTCCTCCGCATTGGACGTGTAATACCCCAATATTCTGTATTAAGAAAAAGTCAGGAAAA
TGGAGGATGCTAATAGATTTTAGGGAATTGAATAAGCAGACAGAGGATCTAGCAGAAGCACAATTAGGAT
TACCGCATCCAGGAGGACTACAAAAGAAAAAGAATATCACTGTATTAGATATAGGGGATGCATATTTTAC
AATTCCATTGTATGAGCCATATAGAAAGTATACATGTTTCACATTGTTAAGTCCCAATAATTTGGGACCA
TGTGAGAGATATTATTGGAAAGTAYTGCCTCAGGGATGGAAATTAAGTCCATCTGTGTATCAATTTACAA
TGCAAAAAATATTGTGGGATTGGAAAAAGGCACACCCTGAGGTACAATTTGGAATCTATATGGATGATAT
CTATATAGGAAGTGATCTAGAGAGAAAAGAGCATAGGCAGAAAGTAGAAGAATTGGCCCAGTATCTAGCG
CAATATGGGTTCATGTTGCCAGAAGATAAAAGGCAAGAAGGGTATCCGGCCAAGTGGCTAGGATTTGAGT
TGCACCCCGAAAAATGGAAATTTCAGAAGCATACCCTCCCAGAGCTTAAAGAAGGCATAATAACTTTAAA
TAAGTTACAAAAATTAGTAGGAGATTTAGTATGGCGACAGTCATTGATAGGAAAAAGTATACCAGAAATA
TTGAAGTTAATGGAGGGGAATAGAGAGTTACAAAGTGAAAGGAAAATAGAGAAAAAGCATGTACTGGAAT
GGGAGGAATGCAGAAAGAAATTAGAAGAAATGGAAGGAAATTATTATGATGAGGAAAAAGATGTATATGG
ACAAATAGACTGGGGAAATAAAGCAATAGAATATATAGTGTTTCAGGAGAAGGGGAAACCATTGTGGGTG
AATGTAGTACACAATGTAAAGAATTTGAGTCAAGCACAACAGATTATTAAAGCAGCGCAGAAATTAACAC
AAGAAGTAATAGTTAGAACAGGAAAAATACCATGGATTTTGTTACCAGGAAAAGAAGAGGATTGGATCTT
AGAGCTACAGATAGGAAACATAACATGGATGCCCTCATTTTGGTCATGCTATAGAGGATCAGTAAGATGG
AAGAAAAGAAATGTAGTAGAAGAAGTAGTCGCAGGACCGACATATTATACAGATGGAGGAAAGAAAAATG
GAACAGGGAGCTTAGGATATATAGCTTCTACAGGAGAAAAATATAGAGCCCATGAGGAAGGGACAAATCA
GCAATTAGAATTAAGAGCAATAGAAGAAGCATGTAAACAGGGACCAGCAGAAATGAATATAGTAACAGAT
AGTAGGTATGCATATGAATTCATGTTGAGAAATTGGGATGAGGAGGTCATAAAGAATCCCATACAGGCAA
GAATTATGAAATTAGTGCATGAGAAAAAGAAAATAGGAATACACTGGGTACCAGGACACAAAGGGATTCC
TCAAAATGAAGAAATAGATAGGTATATATCAGAAATATTTTTAGCAAAGCAAGGGACAGGAATCTGTCCT
AAAAGGAAGGAAGACGCAGGGTATGATTTGATATGCCCACAAGAAGTGAGCATAGCTCCAGGGCAAGTAA
AGAAAATCCCTATAGATCTAAAAATAAATCTAAGAGAGGATCAGTGGGCAATGATAGGGACAAAAAGTAG
TTTCGCAAGCAAAGGCGTATTTATCCAAGGAGGGATAATAGATTCAGGATATCAGGGAACAATACAGGTA
ATAGTATACAATAGTACGGATAAGGAAGTGGTAATACCTCAGGGAAGAAAATTTGCACAAATAATTCTCA
TGCCATTAGTGCATGAAGAATTAGAACCCTGGGGAAAAATGAGAAGAACAGAAAGAGGGGAAAAAGGATT
TGGGTCCACAGGAATGTATTGGATAGAAAATATCCCTGTAGCAGAGGAAGAACATGAGAAGTGGCATCAA
GATGCTATGTCACTGCATCTAGAGTTTGGAATTCCCAGAAGTGCAGCAGAAGATATAATACAACAATGTG
AAGTCTGCCAAGAGAATAAATTGCCGAGTACTATAAGAGGAAGTAACAGAAGGGGGGTAGATCATTGGCA
RGTAGATTATACTCATTATGAGAATAAAATCATATTAGTATGGGTAGAAACAAATTCAGGCTTAATATAT
GCAGAAAGGGTAAAAGGAGAAACAGGAGAAGAATTCAGAACACAGGCATTAAAATGGTACGCCTTGTTTA
AGCCCACATCAGTGCAGTCGGATAATGGGCCAGCATTTGTTGCAGAGGCAACGCAGTTGTTAATGAAGTA
TTTGGGGATTCAACATACAACAGGCATCCCATGGAACCCCCAATCACAAGCATTGGTAGAAAGGGCACAT
CAAACATTAAAACAAATGTTGCAAAAATTAGAGGGAAACTTTGTAGCGTTTGAATCTGCCATAGCAGCCG
CCCTAATATCTCTTAATATAAAAAGAAAGGGTGGGCTAGGGGCAAGCCCTATGGATATATTTATATTTAA
TAAAGAACAACAGAGATTATTAGTACAAAATCATATAAATAAAGAAAAAATTCGATTTTGTTATTACAGG
ATCAGAAAAAAAGGACACCCAGGCGAGTGGCTGGGGCCAACACAGGTACTCTGGGAAGGGGAAGGAGCAA
TTGTAGTTAAAAATAAGGAACAAGACAGATATTTAGTAATAGCCTATAAGGATGTCAAGTTTATACCAGC
CCCGAAAGAATTGCAAAAAGAATAA

>MH374283.1 Small ruminant lentivirus isolate SRLV024, complete genome
CATCAAGAGG
GGGCCACGTGTGGTGCCGTCCGCACCCCCTATGCTGTAACAAACGCACCACCAACAGTAAAAGTACAAGT
AGGGACATGCTGGAAAGAATTATTAATAGATACAGGGGCTGATAGAACAATAGTAAAATACCATGATAGT
ACAGGCATACCTAAAGGGAGAATAATGTTACAGGGAATAGGAGGAATCATAGAGGGAGAAAAATGGGACC
AAGTAAAAATCAAGTATAAAGGAAGAGAAGTAGAAGGAACAATAGTAGTATTGGCGAGTAGCCCAGTAGA
AGTATTAGGTAGAGACAACATGGGGAAGTTAGGAATAGGGATAATAATGGCCAATCTAGAAGAAGGGAAG
ATTCCGGTAACGGAAGTGAAATTAAAAGAAGGATGCAAAGGACCCCATATACCACAATGGCCATTAACGC
AGGAAAATTTGGAAGGACTAAAAGAAATAGTGGAAAGATTAGAAAAAGAAGGAAAAGTAGCGAAAGCTCC
TCCACATTGGACGTGGAATACCCCCATATTCTGTATTAAGAAGAAATCAGGAAAATGGAGAATGTTAATA
GATTTCAGAGAATTGAATAAGCAGACAGAGGATCTAGCGGAGGCACAATTAGGACTACCACACCCAGGAG
GATTACAAAAGAAAAAGAACATCACTGTATTAGATATAGGGGATGCATATTTTACAATTCCATTGTATGA
GCCATATAGAAAATATACATGTTTCACATTGCTAAGTCCTAATAATTTGGGACCATGTGTTAGATATTAT
TGGAAAGTATTGCCTCAGGGATGGAAATTAAGTCCATCAGTATACCAATTCACAATGCAAAAAATATTGT

GGGATTGGAGAAAGGCACACCCTGAGGTGCAATTTGGGATCTATATGGATGATATCTATATAGGAAGTGA
TTTAGAAATAAAAGAACATAGGGAAAAAGTAGAAGAATTAGCACAGTACATAGCGCAATATGGATTCATG
TTGCCAGAAGATAAAAGGCAAGAAGGGTATCCAGCCAAGTGGCTAGGATTTGAGTTGCACCCCGAGAAAT
GGAAATTTCAAAAACATACCCTCCCTGAGCTTAAAGAAGGAATAATAACTTTAAACAAGTTACAAAAATT
AGTAGGGGATTTGGTATGGCGACAGTCATTGATAGGAAAAAGTATACCTGAAATATTGAGGTTAATGGAA
GGGAACAGAGAGTTACAAAGTGAAAGGAAAATAGAAAGGAAACATGTACTGGAATGGGAAGAATGTAGAA
AAAAGTTAGAGAAAATGGAAGGGAGCTACTATAATGAGGAAAAGGATATCTATGGACAAATAGCCTGGGG
AAGTAAAGCAATAGAATATATAGTGTTCCAAGAGAAAGGGAAACCTTTGTGGGTAAATGTGGTACACAAT
ATAAAAAATTTGAGTCAAGCACAACAAATGATCAAAGCAGCACAGAAATTGACACAAGAGGTAATAGTTA
GAACAGGAAAAATACCATGGATTTTGTTACCAGGAAAAGAAGAGGATTGGATTTTAGAGCTACAAATAGG
AAACATAACATGGATGCCTTCATTTTGGTCATGTTATAGAGGATCAGTAAGGTGGAAGAGAAGAAATGTA
GTGGAAGAAGTAGTCCCAGGACCAACATATTATACGGATGGAGGAAAGAAAAATGGGAAAGGAAGTTTGG
GATATATAGCTTCTACGGGAGAAAAATATAGAGCCCATGAAGAGGGAACAAACCAGCAATTAGAATTAAG
AGCAATAGAAGAGGCATGTAAGCAGGGACCACCAGAAATGAATATAGTAACAGATAGTAGGTATGCGTAT
GAATTTATGTTGAGAAATTGGGATGAAGAGGTAATAAGAAATCCCATACAAGCAAGAATTATGCAATTGG
TGCATAAGAAAAAGAAAATAGGAATACACTGGGTGCCAGGACATAAAGGGATCCCTCAGAATGAAGAAAT
AGATAAATATATATCTGAAATATTTTTAGCAATACAAGGGACAGGAATCTGTCCAAAAAGGAAGGAGGAC
GCAGGGTATGACTTAATATGCCCACAGGAGGTAAGCATATCTCCTGGGCAGGTAAAGAAAATCCCTATAG
ACCTAAGAATAAATCTGAAAGAGGATCAATGGGCAATGATAGGGACAAAAAGTAGTTTCGCAAGCAAAGG
AGTATTTATCCAAGGAGGGATAATAGATTCAGGATATCAGGGAACAATACAGGTAGTAGTATACAATAGT
ACTGATAGGGAAGTAGTGATACCGCAAGGAAGAAAATTTGCACAAATAATACTTATGCCATTGATGCATG
AAGAGCTAGAACCATGGGGAAAAATGAGGAAAACAGAAAGAGGGGAACAAGGCTTTGGGTCCACAGGGAT
GTATTGGATAGAGAATATCCCCGTGGCAGAGGAGGAGCATGGGAAGTGGCATCAAGACGCGGTATCGCTA
CAATTAGACTTTGGCATCCCTAGAACGGCAGCAGAAGATATAATACAACAATGTGAAGTGTGCCAAGCAA
ATAAACTGCCGAGTACGCTAAGGGGAAGTAACAGAAGGGGGGTAGATCATTGGCAAGTAGATTATACTCA
TTATGAAAATAAAATTATATTAGTGTGGGTAGAAACCAATTCAGGCTTAATATATGCAGAAAGAGTAAAA
GGAGAAACAGGAGAAGAATTTAGGACACAGGTGTTAAAATGGTACGCCTTGTTTAGGCCAACGTCAGTGC
AGTCAGATAATGGACCAGCATTTGTTGCAGAACCAACACAGTTATTAATGAAATACTTGGGAATTCAGCA
TACAACAGGCATCCCATGGAACCCCCAATCGCAAGCGTTGGTAGAGAGGACCCACCAAACCTTAAAAAAT
ACGCTACAGAAATTAGAGGGAAATTTTGTAGCATTTGAATCTGCAGTAGCAGCCGCCCTAATAACGCTTA
ATATAAAAAGAAAGGGTGGGCTAGGGGCAAGCCCTATGGATATATTTATATTTAATAAAGAACAACAGAG
ATTATTATTACAAAATCAAGAAAATCAAGAAAAAATTCGATTTTGTTATTACAGGATCAGGAAAAAAGGA
CACCCAGGTGAGTGGCTGGGGCCAACAAAGGTACTCTGGGAAGGGGAAGGAGCTATTGTAGTCAAAAATA
AAGAACAAGATAGATACCTGGTGGTGGCCTATAAGGATGTCAAGTTCATACCGGCACCAAAAGACTTGCA
GAAAAGTTAA

>MH374285.1 Small ruminant lentivirus isolate SRLV026, complete genome
CA
TCAAGAGGGGGCCACGTGTGGTGCCGTCCGCACCCCCTATGCTGTAACAAGCGCACCACCAACAGTAAAA
GTACAAGTAGGGACATGCTGGAAAGAATTATTAATAGATACAGGGGCTGATAGAACAATAGTAAAATACC
ATGATAGTACAGGCATACCTAAAGGGAGAATAATGTTACAGGGAATAGGAGGAATCATAGAGGGAGAAAA
ATGGGACAAAGTAAAAATCAAGTATAAAGGAAGAGAAGTAGAAGGAACAATAGTAGTATTGGCGAGTAGC
CCAGTAGAAGTATTAGGTAGAGACAACATGGGGAAGTTAGGAATAGGGATAATAATGGCCAATCTAGAAG
AAGGGAAGATTCCGGTAACGGAAGTGAAATTAAAAGAAGGATGCAAAGGACCCCATATACCACAATGGCC
ATTAACGCAGGAAAAATTGGAAGGACTAAAAGAAATAGTGGAAAGATTAGAAAAAGAAGGAAAAGTAGCG
AAAGCTCCTCCACATTGGACGTGGAATACCCCCATATTCTGTATTAAGAAGAAATCAGGAAAATGGAGAA
TGTTGATAGATTTCAGAGAATTGAATAAGCAGACAGAGGATCTAGCGGAGGCACAATTAGGACTACCACA
CCCAGGAGGATTACAAAAGAAAAAGAACATCACTGTATTAGATATAGGGGATGCATATTTTACAATTCCA
TTGTATGAGCCATATAGAAAATATACATGTTTCACATTGCTAAGTCCTAATAATTTGGGACCATGTGTTA
GATATTATTGGAAAGTATTGCCTCAGGGATGGAAATTAAGTCCATCAGTATACCAATTCACAATGCAAAA
AATATTGTGGGATTGGATAAAGGCACACCCTGAGGTGCAATTTGGGATCTATATGGATGATATCTATATA
GGAAGTGATCTAGAAATAAAAGAACATAGGGAAAAAGTAGAAGAATTAGCACAGTACATAGCGCAATATG
GATTCATGTTGCCAGAAGATAAAAGGCAAGAAGGGTATCCAGCCAAGTGGCTAGGATTTGAGTTGCACCC
CGAGAAATGGAAATTTCAAAAACATACCCTCCCTGAGCTTAAAGAGGGAATAATAACTTTAAACAAGTTA
CAAAAATTAGTAGGAGATTTGGTATGGCGACAGTCATTGATAGGAAAAAGTATACCTGAAATATTGAGGT
TAATGGAAGGGAACAGAGAGTTACAAAGTGAAAGGAAAATAGAAAGGAAACATGTACTGGAATGGGAAGA
ATGTAGGAAAAAGTTAGAGAAAATGGAAGGGAACTACTATAATGAGGAAAAGGATATCTATGGACAAATA
GCCTGGGGAAGTAAAGCAATAGAATATATAGTGTTCCAAGAGAAAGGGAAACCTTTGTGGGTAAATGTGG
TACACAATATAAAAAATTTGAGTCAAGCACAACAAATGATCAAAGCAGCACAGAAATTGACACAAGAGGT
AATAGTTAGAACAGGAAAAATACCATGGATTTTGTTACCAGGAAAAGAAGAGGATTGGATTTTAGAGCTA
CAAATAGGAAACATAACATGGATGCCTTCATTTTGGTCATGTTATAGAGGATCAGTAAGGTGGAAGAGAA

GAAATGTAGTAGAAGAAGTAGTCCCAGGACCAACATATTATACGGATGGAGGAAAGAAAAATGGGAAAGG
AAGTTTGGGATATATAGCTTCTACAGGAGAAAAATATAGAGCCCATGAAGAGGGAACAAACCAGCAATTA
GAATTAAGAGCAATAGAAGAGGCATGTAAGCAGGGACCACCAGAAATGAATATAGTAACAGATAGTAGGT
ATGCGTATGAATTTATGTTGAGAAATTGGGATGAAGAGGTAATAAGAAATCCCATACAAGCAAGAATTAT
GCAATTGGTGCATAAGAAAAAGAAAATAGGAATACACTGGGTGCCAGGACATAAAGGGATCCCTCAAAAT
GAAGAAATAGATAAATATATATCTGAAATATTTTTAGCAATACAAGGGACAGGAATCTGTCCAAAAAGGA
AGGAGGACGCAGGGTATGACTTAATATGCCCACAGGAGGTAAGCATATCTCCTGGGCAGGTAAAGAAAAT
CCCTATAGACCTAAGAGTAAATCTGAAAGAGGATCAATGGGCAATGATAGGGACAAAAAGTAGTTTCGCA
AGCAAAGGAGTATTTATCCAAGGAGGGATAATAGATTCAGGATATCAGGGAACAATACAGGTAGTAGTAT
ACAATAGTACTGATAGGGAAGTAGTGATACCGCAAGGAAGAAAATTTGCACAAATAATACTTATGCCATT
GATGCATGAAGAACTAGAACCATGGGGAAAAATGAGAAAAACAGAAAGAGGGGAACAAGGCTTTGGGTCC
ACAGGGATGTATTGGATAGAGAATATCCCCGTGGCAGAGGAGGAGCATGGGAAGTGGCATCAAGACGCGG
TATCGCTACAATTAGACTTTGGCATCCCTAGAACGGCAGCAGAAGATATAATACAACAATGTGAAGTGTG
CCAAGCAAATAAACTGCCGAGTACGCTAAGGGGAAGTAACAGAAGGGGGGTAGATCATTGGCAAGTAGAT
TATACTCATTATGAAAATAAAATTATATTAGTGTGGGTAGAAACCAATTCAGGCTTAATATATGCAGAAA
GAGTAAAAGGAGAAACAGGAGAAGAATTTAGGACACAGGTGTTAAAATGGTACGCCTTGTTTAGGCCAAC
GTCAGTACAGTCAGATAATGGACCAGCATTTGTTGCAGAACCAACACAGTTATTAATGAAATACTTGGGA
ATTCAGCATACAACAGGCATCCCATGGAACCCCCAATCGCAAGCGTTGGTAGAGAGGACCCACCAAACCT
TAAAAAATACGCTACAGAAATTAGAGGGAAATTTTGTAGCATTTGAATCTGCAGTAGCAGCCGCCCTAAT
AACGCTTAATATAAAAAGAAAGGGTGGGCTAGGGGCAAGCCCTATGGATATATTTATATTTAATAAAGAA
CAACAGAGATTATTATTACAAAATCAAGAAAATCAAGAAAAAATTCGATTTTGTTATTACAGGATCAGGA
AAAAAGGACACCCAGGTGAGTGGCTGGGGCCAACAAAGGTACTCTGGGAAGGGGAAGGAGCTATTGTAGT
CAAAAATAAAGAACAAGATAGATACCTGGTGGTGGCCTATAAGGATGTCAAGTTCATACCGGCACCAAAA
GACTTGCAGAAAAGTTAA

>MH374286.1 Small ruminant lentivirus isolate SRLV032, complete genome
CATCAAGA
GGGGGCCACGTGTGGTGCCGTCCGCACCCCCTATGCTGTAACAAGCGCACCACCAACAGTAAAAGTACAA
GTAGGGACATGCTGGAAAGAATTATTAATAGATACAGGGGCTGATAGAACAATAGTAAAATACCATGATA
GTACAGGCATACCTAAAGGGAGAATAATGTTACAGGGAATAGGAGGAATCATAGAGGGAGAAAAATGGGA
CAAAGTAAAAATCAAGTATAAAGGAAGAGAAGTAGAAGGAACAATAGTAGTATTGGCGAGTAGCCCAGTA
GAAGTATTAGGTAGAGACAACATGGGGAAGTTAGGAATAGGGATAATAATGGCCAATCTAGAAGAAGGGA
AGATTCCGGTAACGGAAGTGAAATTAAAAGAAGGATGCAAAGGACCCCATATACCACAATGGCCATTAAC
GCAGGAAAAATTGGAAGGACTAAAAGAAATAGTGGAAAGATTAGAAAAAGAAGGAAAAGTAGCGAAAGCT
CCTCCACATTGGACGTGGAATACCCCCATATTCTGTATTAAGAAGAAATCAGGAAAATGGAGAATGTTGA
TAGATTTCAGAGAATTGAATAAGCAGACAGAGGATCTAGCGGAGGCACAATTAGGACTACCACACCCAGG
AGGATTACAAAAGAAAAAGAACATCACTGTATTAGATATAGGGGATGCATATTTTACAATTCCATTGTAT
GAGCCATATAGAAAATATACATGTTTCACATTGCTAAGTCCTAATAATTTGGGACCATGTGTTAGATATT
ATTGGAAAGTATTGCCTCAGGGATGGAAATTAAGTCCATCAGTATACCAATTCACAATGCAAAAAATATT
GTGGGATTGGAGAAAGGCACACCCTGAGGTGCAATTTGGGATCTATATGGATGATATCTATATAGGAAGT
GATCTAGAAATAAAAGAACATAGGGAAAAAGTAGAAGAATTAGCACAGTACATAGCGCAATATGGATTCA
TGTTGCCAGAAGATAAAAGGCAAGAAGGGTATCCAGCCAAGTGGCTAGGATTTGAGTTGCACCCCGAGAA
ATGGAAATTTCAAAAACATACCCTCCCTGAGCTTAAAGAGGGAATAATAACTTTAAACAAGTTACAAAAA
TTAGTAGGAGATTTGGTATGGCGACAGTCATTGATAGGAAAAAGTATACCTGAAATATTGAGGTTAATGG
AAGGGAACAGAGAGTTACAAAGTGAAAGGAAAATAGAAAGGAAACATGTACTGGAATGGGAAGAATGTAG
GAAAAAGTTAGAGAAAATGGAAGGGAACTACTATAATGAGGAAAAGGATATCTATGGACAAATAGCCTGG
GGAAGTAAAGCAATAGAATATATAGTGTTCCAAGAGAAAGGGAAACCTTTGTGGGTAAATGTGGTACACA
ATATAAAAAATTTGAGTCAGGCACAACAAATGATCAAAGCAGCACAGAAATTGACACAAGAGGTAATAGT
TAGAACAGGAAAAATACCATGGATTTTGTTACCAGGAAAAGAAGAGGATTGGATTTTAGAGCTACAAATA
GGAAACATAACATGGATGCCTTCATTTTGGTCATGTTATAGAGGATCAGTAAGGTGGAAGAGAAGAAATG
TAGTAGAAGAAGTAGTCCCAGGACCAACATATTATACGGATGGAGGAAAGAAAAATGGGAAAGGAAGTTT
GGGATATATAGCTTCTACAGGAGAAAAATATAGAGCCCATGAAGAGGGAACAAACCAGCAATTAGAATTA
AGAGCAATAGAAGAGGCATGTAAGCAGGGACCACCAGAAATGAATATAGTAACAGATAGTAGGTATGCGT
ATGAATTTATGTTGAGAAATTGGGATGAAGAGGTAATAAGAAATCCCATACAAGCAAGAATTATGCAATT
GGTGCATAAGAAAAAGAAAATAGGAATACACTGGGTGCCAGGACATAAAGGGATCCCTCAAAATGAAGAA
ATAGATAAATATATATCTGAAATATTTTTAGCAATACAAGGGACAGGAATCTGTCCAAAAAGGAAGGAGG
ACGCAGGGTATGACTTAATATGCCCACAGGAGGTAAGCATATCTCCTGGGCAGGTAAAGAAAATCCCTAT
AGACCTAAGAGTAAATCTGAAAGAGGATCAATGGGCAATGATAGGGACAAAAAGTAGTTTCGCAAGCAAA
GGAGTATTTATCCAAGGAGGGATAATAGATTCAGGATATCAGGGAACAATACAGGTAGTAGTATACAATA
GTACTGATAGGGAAGTAGTGATACCGCAAGGAAGAAAATTTGCACAAATAATACTTATGCCATTGATGCA
TGAAGAACTAGAACCATGGGGAAAAATGAGAAAAACAGAAAGAGGGGAACAAGGCTTTGGGTCCACAGGG

ATGTATTGGATAGAGAATATCCCCGTGGCAGAGGAGGAGCATGGGAAGTGGCATCAAGACGCGGTATCGC
TACAATTAGACTTTGGCATCCCTAGAACGGCAGCAGAAGATATAATACAACAATGTGAAGTGTGCCAAGC
AAATAAACTGCCGAGTACGCTAAGGGGAAGTAACAGAAGGGGGGTAGATCATTGGCAAGTAGATTATACT
CATTATGAAAATAAAATTATATTAGTGTGGGTAGAAACCAATTCAGGCTTAATATATGCAGAAAGAGTAA
AAGGAGAAACAGGAGAAGAATTTAGGACACAGGTGTTAAAATGGTACGCCTTGTTTAGGCCAACGTCAGT
ACAGTCAGATAATGGACCAGCATTTGTTGCAGAACCAACACAGTTATTAATGAAATACTTGGGAATTCAG
CATACAACAGGCATCCCATGGAACCCCCAATCGCAAGCGTTGGTAGAGAGGACCCACCAAACCTTAAAAA
ATACGCTACAGAAATTAGAGGGAAATTTTGTAGCATTTGAATCTGCAGTAGCAGCCGCCCTAATAACGCT
TAATATAAAAAGAAAGGGTGGGCTAGGGGCAAGCCCTATGGATATATTTATATTTAATAAAGAACAACAG
AGATTATTATTACAAAATCAAGAAAATCAAGAAAAAAATTCGATTTTGTTATTACAGGATCAGGAAAAAAG
GACACCCAGGTGAGTGGCTGGGGCCAACAAAGGTACTCTGGGAAGGGGAAGGAGCTATTGTAGTCAAAAA
TAAAGAACAAGATAGATACCTGGTGGTGGCCTATAAGGATGTCAAGTTCATACCGGCACCAAAAGACTTG
CAGAAAAGTTAA

>MG554405.1 Small ruminant lentivirus isolate SRLV004, complete genome
CAGCAGGAGGGGGCCACGTGTGGTGCCGTCC
GCACCCCCTATGCTGTAACAGACGCACCACCAAAAGTAAAAGTTCAAGTAGGGACAAGTTGGAAGGAATT
ATTAATAGATACAGGGGCTGATAGAACAATAGTAAAATACCATGATAGTACAGGCATACCTGAAGGGAGA
ATAAAATTACAGGGAATAGGAGGAATCATAGAGGGAGAAAAATGGAAACAAGTAAAAATTAAGTATAGAG
GAAAAGAAATCGAAGGGACAATAGTAGTGTTGGCGAGTAGCCCAGTAGAAGTGTTAGGCAGGGATAATAT
GGGAAAGTTAGGAATAGGGATAATAATGGCAAATTTAGAAGAAGGGAAAATTCCCGTGACAGAAGTAAAA
TTGAAAGAAGGATGTAAAGGACCTCATATACCACAATGGCCATTAACACAGGAAAAATTGGAGGGATTAA
AAGAAATAGTAGAGAGACTAGAAAAAGAAGGAAAAGTAGAAAAAGCTCCTCCACATTGGCCGTGGAATAC
CCCTATATTCTGTATTAGGAAAAAATCAGGGAAATGGAGGATGTTAATAGATTTTAGGGAATTAAATAAA
CAGACAGAAGACTTAGCAGAGGCTCAATTAGGACTACCGCATCCCGGAGGATTACAAAAGAGAAAGAATA
TCACAATATTAGATATAGGGGATGCATATTTTACAATTCCATTGTATGAGCCATATAGAAGGTATACATG
TTTCACATTGTTAAGTCCTAATAATTTGGGACCATGTGTGAGGTATTATTGGAAAGTACTACCTCAGGGA
TGGAAATTAAGTCCATCTGTATACCAGTTCACAATGCAAAAAATATTGTGGGACTGGATAAAGGCTCATC
CTGAGATACAATTTGGAATCTATATGGATGATATCTATATAGGAAGTGATCTAGAGATCAAAGAGCATAG
AGAAAAAGTAGAAGAATTAGCACAGTATCTAGCGCAATATGGATTCATGTTGCCAGAGGATAAAAGGCAA
GAAGGGTATCCAGCCAAGTGGCTAGGATTTGAGTTGCATCCGGAGAAGTGGAAATTTCAGAAACATACCC
TCCCGGAACTCAAAGAAGGAACAATAACTTTAAATAAGTTACAAAAATTAGTAGGAGACTTAGTATGGCG
ACAGTCATTAATAGGGAAAAGTATACCAGAAATATTGAAATTAATGGAAGGAGATAGAGAATTGCAGAGT
GAGAGAAAAATAGAAAGAAAACATGTATTAGAATGGGAAGCATGTAGGAAAAAGTTAAAAGAAATGGAAG
GAAGCTACTATAATGAGGAAAAAGATGTTTATGGGCAAATAGACTGGGGAAATAAAGCAATAGAATACAT
AGTGTTTCAAGAGAAAGGGAAACCTCTATGGGTGAATGTGATGCACAATGTAAAGAATTTAAGTCAGGCA
CAACAAATTATTAAAGCAGCACAGAAATTAACACAGGAAGTAATAGTTAGGACAGGAAAAATACCATGGA
TTTTGTTACCAGGAAAAGAGGAGGATTGGATTCTAGAACTACAAATGGGAAACATAACATGGATGCCCTC
ATTTTGGTCATGTTATCGGGGGTCAGTAAGGTGGAAGAAAAGAAATGTAGTGGAAGAAGTAGTCCCAGGA
CCAACATATTATACAGACGGAGGAAAGAAAAATGGCGTAGGAAGCTTAGGATATATAGCATCTACAGGAG
AAAAATACAGAGCCCATGAAGAAGGGACAAATCAGCAATTGGAGTTAAGAGCAATAGAGGAAGCATGTAA
ACAGGGACCAGCAGAAATGAATATAGTAACAGATAGTAGGTACGCATATGAATTTATGTTGAGAAATTGG
GATGAGGAAGTCATAAAGAATCCCATACAGGCAAGAATTATGCAATTGGTGCATAAGAAAAATAAGATAG
GAATACACTGGGTGCCAGGACATAAAGGGATCCCTCAAAATGAAGAAATAGATAGATATATATCAGAAGT
ATTTTTAGCAGTACAAGGGGCAGGAATAGAACAAAAAAGAAAGGAAGATGCAGGGTATGATTTGATATGC
CCACAGGAAGTAAGCATACCTCCAGGACAGGTGGAGAGAGTCCCTATAGATTTAAAGGTAAATCTGAAGG
AAAATCAATGGGCAATGATAGGGACAAAAAGTAGTTTCGCCAGCAAAGGAGTCTTTATCCAAGGAGGGAT
AATAGATTCCGGATATCAAGGAACAATACAGGTAATAGTATATAATAGTACTGATAAGGAGGTAGTAATA
CCGCAGGGAAGAAAATTTGCACAATTAATTCTCATGCCATTACAGCATGAGGAATTGGAACCATGGGGTA
ACATAAGAAAAACAGAAAGGGGAGAAAGAGGATTTGGATCCACAGGAATGTATTGGATAGAAAACATTCC
TGCGGCAGAAGAAGAGCATCAAAAGTGGCATCAAGACGCGATGTCACTGCACCTAGAATTTGGCATTCCT
AGAACGGCAGCAGAAGATATAATACAACAATGTGAAGTCTGCCAAGAGAATAAATTGCCAAGTACGATAA
GGGGAGGAAACAGAAGAGGGGTAGATCATTGGCAAGTAGATTATACTCATTATGAGAATAAAATTATATT
GGTCTGGGTAGAAACAAATTCAGGGTTAATATATGCAGAAAGAGTGAGAGGAGAAACAGGGGAAGAATTC
AGAACGCAGGTATTAAGATGGTACGCCTTGTTTAGACCAGCCTCAGTACAGTCCGATAATGGACCTGCAT
TTGTAGCAGACGCAACACAATTGCTAATGAAATACTTGGGGATTCAACATACAACAGGCATCCCATGGAA
TCCGCAATCACAAGCATTAGTGGAACGAACACATCAAACGTTAAAACAAATGTTGAAAAAATTGGAGGGT
AACTTTGTAGCATTTGAATCTGCCCTAGCAGCTGCCCTAATAGCGCTTAATATAAAAAGAAAGGGTGGGC
TAGGGACAAGCCCTATGGATATATTTATATTTAATAAAGAACAACAGAGATTATTATTACAAAATCAAGT
AAATAAAGAAAAAATTCGATTTTGTTATTACAGGATCAGGAAAAAAGGACAATCAGGTGAGTGGCTGGGG
CCAACACAGGTACTCTGGGAAGGGGACGGAGCCATTGTAGTTAAAAATAAAGAACAAGATAGGTATTTAG

TGATAGCATATAAAGATGTCAAGTTCATACCGGCCCCTAAAGAATTGCAAAAAGCATAA

>AY445885.1 Small ruminant lentivirus gag protein (gag), pol protein (pol), protein 1 (ORF 1), protein 2 (ORF 2), and env (env) genes, complete cds
ATGCCATCATTGTGG
AAAAAGGGGACATATGCAGAAGGACTGTGGGCAAAAAAGAGGAGAGAAGAGAACACAACAGCAGGGAAAC
AGCAGGAGGGGGCCACGTGTGGTGCCGTCCGCGCCTCCTATGCTTTAACAGAAGCACCACCTCTAGTAGA
CATAAGAATAGGGACGAAGTGGAAGAAAGTATTAGTGGATACAGGAGCAGATAGAACTATAGTAAGATAT
CATGATAATACAGGACTACCAAAGGGTAGAATAAAGTTACAAGGAATAGGGGGAATTATAGAAGGAGAAA
AATGGGAAAAAGTACCGATACACTATAAAGAAAAGGTAATAGAAGGAACACTAGTAGTACTGCCTAGCAG
TCCTGTAGAAGTGCTGGGGAGGGACAATATGGAAATGTTAGGAATTAAGCTAATTATGGCAAATTTAGAA
GAAAAGAAGATTCCTATAACAAAAGTAACCTTAAAGGAGGGATATAAAGGACCACATGTGGCACAATGGC
CCCTGACACAAGAGAAGTTAGAGGGATTAAAGGAAATTGTGGAGAGACTGGAAAAGGAGGGGAAATTAGG
TAGGGCACCCCCACATTGGACATGTAATACCCCCATATTTTGTATCAAGAAAAAGTCAGGGAAATGGAGA
ATGCTAATAGATTTTAGGGAATTAAATAAACAAACAGAGGATTTAGCAGAAGCACAATTAGGATTACCGC
ACCCAGGGGGATTGAAGAAGAGAAAAAATGTAACAGTATTAGATATAGGGGATGCATATTTTACTATCCC
CTTGTATGAACCATACAGACAATATACATGCTTTACTTTGTTGAGTCCTAACAATTTGGGACCCTGTGTA
AGGTATTATTGGAAAGTATTACCACAAGGGTGGAAATTAAGTCCTTCAGTATATCAATTTACAATGCAAG
AAATCCTATGGGAGTGGATAAAAGAACATCCACTGATACAATTTGGAATTTATATGGATGGCATTTATAT
AGGAAGTGACCTAGAGATAGGAGAACATAGAAAAATAGTGAACAATCTAGCAGGGTATATCGCACAATAT
GGGTTTATGTTACCAGAAGAGAAAAGACAGGAAGGATATCCTGCCCATTGGTTAGGATTTGAGCTACACC
CGGATAAATGGAAATTTCAAAAACATACATTGCCGGAATTGACGGAAGGGCCTATTGCTTTAAATAAGTT
ACAGAAATTAGTAGGGGATTTAGTTTGGAGGCAATCATTAATAGGCAAGAGTATTCCAAGTATATTAAAA
TTAATGGAAGGAGATAGAGCATTACAAAGTGAGAGGCAAATAGAAAAGAATCACATACAGGAATGGGAGG
AATGTAGGAAAAAATTAGCAAAAATGGAAGGAAGTTATTATGATGAAGAGAAGGATATTTATGGACAGAT
AGACTGGGGTTGTAAAGCAGTTGAATATATAGTATTCCAGGAGAAAGGAAAACCCTTGTGGGTAAATGTG
GTTCATAGTATTAAGAATTTGAGTCAGGCACAACAAATTATTAAAGCGGCGCAGAAACTTACCCAAGAAG
TGATAATAAGAACAGGAAAAATACCTTGGATAATGTTACCAGGAAAAGAAGAAGACTGGATATTAGAATT
GCAAATAGGGAATATAACATGGATGCCTTCATTTTGGTCGTGCTATAGAGGATCAGTAAGATGGAAGAAA
AGAAATGTGGTGACGGAGGTAGTGGAAGGGCCAACCTTCTATACAGATGGGGGGAAGAAGAATGGGATAG
GAAGCTTAGGGTATGTGGCCTCTACAGGAGCCAAGTACAGACAACATGAGTCAGGGACAAATCAGCAATT
AGAATTAAGGGCAATAGAAGAAGCATGTAAACAGGGACCCGCAAAAATGAATATAGTAACAGATAGCAGA
TATGCATATGAATTTATGTTAAGAAATTGGGATGAAGAAGTCATAAAAAATCCCATACAGGCACGGATTA
TGAAAATAGTTCATGATAAAGACAAAATAGGAGTACATTGGGTGCCAGGACATAAAGGGATCCCTCAAAA
TGAAGAAGTGGACAAATATATTTCAGAAATATTCTTGGCAAAGGAAGGGGAAGGAATTCTAGGAAAAAGA
AAAGAAGATGCAGGATATGATTTAATTTGCCCACAGAGAGTAGATATCCCACCAGGACAGGTAAGAAAAA
TACCCGTAGATCTTAGATTGAATTTGAAGGAGAAGCAATGGGCCATGATAACCACAAAAAGTAGTTTTGC
AAGCAAGGGAGTGTTTGTTCAAGGAGGAATAATAGACTCAGGATATCAAGGAACAATACAAGTAGTAGTA
TATAACAGTAATGACGTAGCAGTAATAATACCCCAGGGGAGAAAGTTTGCACAATTAATCCTCATGCCGT
TAATACATGAGGAATTGGAACCATGGGGAAAAACAAGGGAAACAGAGAGAGGAGAACAAGGATTTGGGTC
CACGGGGGCATATTGGATTGAGAATATCCCACTGGCAGAAGAAGAGCACAACAAATGGCATCAAGATGCT
ACGTCATTGCATTTGGACTTTGGAATTCCTAAATCCGCCGCGGAGGATATAATACAGCAATGTGAAAGCT
GTCAAGAAAATAAAATGCCAAGTGCCATTAGAGGAGGGAATAAAAGGGGAATAGATCATTGGCAAGTGGA
TTATACTCATTATGAGGATAAAATCATATTAACATGGATAGAAACAAATTCAGGATTGATCTATGCCGAA
AGGGTAAAAGGGGAAACAGGGCAAGAATTTAGAATCCAAGTTATGAAATGGTACTCTATGTTTGATCCAA
AATCATTGCAGTCGGATAATGGACCTGCGTTTATTGCAGAACCTACACAGCTCTTGATGAAATATTTGGG
AATAGAACACACAACGGGAATCCCGTGGAATCCCCAATCACAGGCCTTAGTGGAAAGGGCACATCAAACG
TTAAAACACACTTTGGAAAAATTTGTTCCCATGTTTGTTGCATTTGAATCTGCTCTTGCTGCGGCACTAA
TAGCTCTAAATATAAAAAGAAAGGGTGGGCTAGGGACAAGCCCTATGGACATATTTATATTTAATAAAGA
ACAACAAAGAATACAGCAACAAAGTATCATGAATAAATCAAAAATTCGATTTTGTTATTACAGAATCAGA
AAGAGAGGACATCCAGGCGACTGGCTTGGACCAACTCAGGTACTCTGGGAAGGGGACGGAGCTATAGTTG
TGAAGGATAAAGCTATAGAGAAGTACATAGTAATAGCAAAAAAGGATGTTAAATTTATACCAGAGCCCAA
AGAAAAAGGAAAAGAAAAAGAGTAG

>MG554404.1 Small ruminant lentivirus isolate SRLV003, complete genome
CAGCAGGAGGGGGCCACGTGTGGTGCCGTCCGCACCCCCTATGCTGTAACAGACGCACCAC
TAAAAGTGAAAGTTCAGGTAGGGACAAGTTGGAAGGAATTATTGATAGATACAGGGGCTGATAGAACAAT
AGTAAAGCATCATGATAGTACGGGCATACCTGAAGGGAGAATAAAGTTACAGGGAATAGGAGGAATCATA
GAGGGAGAAAAATGGAAACAAGTAAAAATTAAGTATAGAGGAAAAGAAATCGAAGGGACAATAGTAGTAT
TGGCGAGTAGCCCAGTAGAAGTGTTAGGAAGGGATAATATGGGAAAGTTAGGAATAGGGATAATAATGGC

AAATTTAGAAGAAGGAAAAATACCTGTGACAGAAGTAAAATTAAAGGAAGGATGCAAAGGACCTCATATA
CCACAATGGCCATTAACACAGGAGAAATTGGAAGGATTAAAAGAAATAGTAGAGAGATTAGAAAAAGAAG
GAAAAGTAGAAAAAGCTCCTCCACATTGGACGTGTAATACTCCTATATTCTGTATTAAGAAAAAGTCAGG
GAAATGGAGGATGTTAATAGATTTTAGGGAATTGAATAAACAAACAGAAGATTTAGCAGAGGCTCAATTA
GGACTACCGCATCCCGGAGGATTACAAAAGAGAAAGAATATCACAATATTAGACATAGGGGATGCATATT
TTACAATTCCATTGTATGAGCCATATAGAAAGTATACATGTTTCACATTGTTAAGTCCTAATAATTTGGG
ACCGTGTGTGAGGTATTATTGGAAAGTACTGCCTCAGGGATGGAAGTTAAGTCCATCTGTGTACCAATTC
ACAATGCAAAAGATATTGTGGGATTGGATAAAAGCTCATCCTGAGGTACAATTTGGAATTTATATGGATG
ATATCTATATAGGAAGTGATCTAGAGCTTAAAGAGCATAGAAAGAGGGTAGAAGAATTAGCACAATATAT
AGCGCAATATGGATTCATGTTGCCAGAAGATAAGAGGCAGGAAGGGTATCCGGCCAAGTGGCTAGGATTT
GAATTACACCCTGACAAATGGAAATTTCAGAAGCATACCCTCCCTGAACTCAAAGAGGGAACAATAACTT
TAAATAAGTTACAGAAATTGGTAGGAGATTTAGTATGGCGACAGTCATTAATAGGAAAAAGCATACCAGA
AATATTGAAATTAATGGAAGGAGATAGAGAATTACAAAGTGAGAGGAAAATAGAGAGAAAACATGTATTA
GAATGGGAAGCATGTAGGAAAAAGTTAGAGGAAATGGAAGGAAATTACTATAATAAGGAAAAAGATGTTT
ATGGACAAATAGACTGGGGAAATAAAGCAATAGAATACATAGTGTTTCAGGAGAAAGGGAAACCTCTATG
GGTGAATGTGATGCACAATGTAAAGAATTTAAGTCAGGCACAACAAATTATTAAAGCAGCACAGAAATTA
ACACAGGAAGTAATAGTTAGGACAGGAAAAATACCATGGATTTTGTTACCAGGAAAAGAGGAGGATTGGA
TTTTAGAACTACAAATGGGAAACATAACATGGATGCCCTCATTTTGGTCATGTTATCGAGGGTCAGTAAG
GTGGAAGAAAAGAAATGTAATAGAAGAAGTGGTCCCAGGACCAACATATTATACAGACGGAGGAAAGAAA
AATGGGATAGGAAGCTTAGGATATATAGCTTCTACAGGAGAAAAATACAGAGCCCATGAGGAAGGGACAA
ATCAGCAATTGGAGTTAAGAGCAATAGAGGAAGCATGTAAACAGGGACCAGCAGAAATGAATATAGTAAC
AGATAGTAGGTACGCATATGAATTTATGTTAAGAAATTGGGATGAGGAAGTAATAAAGAATCCCATACAG
GCAAGAATTATGCAATTGGTGCATAGGAAAAGGAAAATAGGAATACACTGGGTGCCAGGACATAAAGGGA
TCCCTCAAAATGAAGAAATAGATAGATATATATCAGAAGTATTTTTAGCAGTACAAGGAACAGGGATAAG
TCCAAAAAGAAAAGAAGATGCAGGATATGACTTGATATGTCCGCAGGAAGTAAGCATATTACCAGGGCAC
GTAAAGAGAATCCCTATAGATTTAAAGTTAAATCTGAGAAAAAATCAATGGGCAATGATAGGAACAAAAA
GTAGTTTCGCCAGCAAGGGTGTATTTATCCAAGGAGGGATAATAGATTCAGGATATCAAGGAACAATACA
GGTAATAGTGTACAACAGTACAAATAAAGAAGTAGTGATACCGCAAGGAAGAAAATTCGCACAAGTAATT
CTCATGCCTCTGGTACATGAGGAATTAGAACCATGGGGAAAAACAAGGAAAACAGAAAGAGGGGAACAAG
GATTTGGCTCCACAGGAATGTATTGGATAGAAAACATTCCTGCGGCAGAAGAAGAACATGGAAAGTGGCA
TCAAGATGCTACATCACTGCAGGTAGAATTCGGTATCCCTAAGCAGGCAGCGGAAGATATAGTACAACAA
TGTGATGTCTGCCAAGAGAATAAATTGCCAAGTACTATAAGGGGAAGTAATAAAAGAGGAGTAGATCATT
GGCAGGTAGATTATACCCATTATGAAAATAAAATTATATTAGTATGGGTAGAGACAAATTCAGGTTTAAT
ATATGCAGAAAGAGTGAAAGGAGAAACAGGAGAAGAGTTCAGAACACAAGTGTTAAAGTGGTATGCTTTG
TTTAAGCCAACATCAGTGCAGTCCGATAATGGACCAGCATTTGTTGCAGAGGCAACACAACTGTTAATGC
AATACTTAGGGATTCAACATACAACAGGTATTCCGTGGAATCCTCAATCACAAGCATTAGTGGAAAGGAC
TCACCAAACATTGAAAACCATGTTGCACAAATTAGAGGGAAATTTTGTAGCATTTGAATCCGCATTAGCA
GCCGCCCTAATAGCACTTAATATAAAAAGAAAGGGTGGCCTAGGGGCAAGCCCTATGGATATATTTATAT
TTAATAAAGAACAACAGAGATTATTATTACAAAATCAAGTAAATAAAGAAAAAATTCGATTTTGTTATTA
CAGGATCAGGAAAAAAGGACAATCAGGTGAGTGGCTGGGGCCAACACAGGTACTCTGGGAAGGGGAAGGA
GCCATTGTAGTTAAAAATAAAGAACAAGACAGGTATTTAGTGATAGCATATAAGGATGTCAAGTTCATAC
CGGCCCCTAAAGAATTGCAAAAAGCATAA

>MG554408.1 Small ruminant lentivirus isolate SRLV007, complete genome
CA
GCAGGAGGGGGCCACGTGTGGTGCCGTCCGCACCCCCTATGCTGTAACAGACGCACCACCCTTAGCAGAA
GTAAAAGTAGGGACATGTTGGAAAGAATTATTAATAGACACAGGGGCTGATAGAACAATAGTAAAGCATC
ATGATAGTACAGGCATACCAAGAGGAAGAATAAAATTACAGGGAATAGGAGGAATTATAGAAGGAGAAAA
ATGGGATCGTGTAAAAATTAGGTATAAAGGAAGGGAGGTCGAAGGAACAATAGTGGTGTTGGCGAGTAGC
CCAGTAGAAGTATTAGGTAGGGACAACATGGGAGAGTTAGGAATAGGAATAATAATGGCCAATTTAGAAG
AAGGAAAAATTCCTATAACTGAAGTAAAATTGAAAGAAGGATGTAAAGGACCTCATATACCACAATGGCC
ATTAACACAGGAGAAATTGGAAGGATTAAGAGAAATAGTAGAGAGATTAGAGAAAGAAGGAAAAGTAGCG
AAAGCTCCTCCACATTGGACGTGCAATACTCCTGTATTCTGTATTYTGAAGAAGTCAGGAAAGTGGAGGA
TGTTAATAGATTTTAGAGAATTGAACAAACAGACAGAAGATCTAGCAGAGGCACAATTAGGACTACCACA
CCCAGGGGGACTACAAAAGAGAAAGAATATCACAATATTAGACATAGGGGATGCATATTTCACAATCCCA
TTATATGAGCCATACAGAAAGTATACATGTTTTACATTGTTAAGTCCCAATAATTTGGGACCATGTGTTA
GATATTATTGGAAAGTACTACCTCAGGGATGGAAATTAAGTCCATCTGTATACCAGTTCACAATGCAAAA
AATATTGTGGGACTGGATAAAGGCTCACCCTGAGATACAATTTGGAATCTATATGGATGATATCTATATA
GGAAGTGATCTAGAGATCAAAGAACATAGAGAAAAAGTAGAAGAATTAGCACAGTATCTAGCGCAATATG
GATTCATGTTGCCAGAGGATAAAAGGCAAGAAGGGTATCCGGCCAAGTGGCTAGGATTTGAGTTGCATCC
GGAGAAGTGGAAATTTCAGAAACATACCCTCCCGGAACTCAAAGAGGGAACAATAACTTTAAATAAGTTA

CAAAAATTGGTAGGAGACTTAGTATGGCGACAGTCATTAATAGGGAAGAGTATACCAGAAATATTGAAAT
TAATGGAAGGAGATAGAGAATTGCAGAGTGAGAGAAAAATAGAAAGAAAACATGTATTAGAATGGGAAGA
ATGTAGGAAAAAATTAAAAGAAATGGAAGGAAGCTACTATAATGAGGAAAAAGATGTGTATGGACAAATA
GACTGGGGAAATAAAGCGATAGAATATATAGTATTTCAGGAAAAAGGGAAACCTCTATGGGTGAATGTGA
TGCACAATATAAAAAACTTAAGTCAGGCACAACAAATTATTAAAGCAGCGCAGAAATTGACGCAAGAAGT
AATAGTTAGAACAGGGAAGATACCATGGATTCTCTTGCCAGGAAAAGAAGAGGATTGGATTCTAGAACTA
CAAATAGGGAACATAACATGGATGCCATCGTTTTGGTCATGTTATAGAGGATCAGTAAGGTGGAAGAAAA
GAAATGTAGTGGAAGAAGTAGTCCCAGGACCAACATATTACACAGATGGAGGAAAGAAAAATGGAACAGG
GAGCTTGGGATATATAGCATCTACAGGAGAAAAATTTAGAGCCCATGAGGAAGGGACAAATCAGCAATTA
GAACTAAGAGCAATAGAAGAAGCATGTAAACGGGGACCACCAGAAATGAATATAGTAACAGATAGCAGGT
ATGCTTATGAATTTATGTTGAGAAATTGGGATGAGGAAGTCATAAAGAATCCCATACAGGCAAGAATTAT
GCAATTGGTGCATAAGAAAAATAAAATAGGAATACATTGGGTACCAGGACACAAAGGGATTCCTCAAAAT
GAAGAAATAGATACATATATATCAGAAATATTTTTAGCAGTACAAGGGGCAGGAATAGAACAAAAAAGAA
AGGAGGATGCAGGGTATGATTTGATATGCCCACAGGAAGTAAGCATACCCCCAGGACAGGTGGAGAGAGT
CCCTATAGATTTAAAGGTAAATCTAAAGGAAAATCAATGGGCAATGATAGGAACAAAAAGTAGTTTTGCC
AGCAAAGGAGTCTTTATCCAAGGAGGGATAATAGATTCGGGATATCAAGGAACAATACAGGTAATAGTAT
ATAATAGTACTGATAAGGAGGTAGTAATACCGCAGGGAAGAAAATTTGCACAATTAATTCTCATGCCATT
ACAGCATGAGGAATTGGAACCATGGGGAAAAATAAGAAAAACAGAAAGGGGAGAAAGAGGATTTGGATCC
ACAGGAATGTATTGGATAGAAAACATTCCTGCAGCAGAAGAGGAGCATCAAAAGTGGCATCAAGACGCGA
TGTCACTGCACCTAGAATTTGGTATTCCTAGAACGGCAGCAGAAGATATAATACAACAATGTGAAGTCTG
CCAAGAGAATAAATTGCCAAGTACTCTAAGAGGAGGAAATAAAAGGGGAGTAGATCATTGGCAAGTGGAT
TATACTCATTATGAGAATAAAATTATATTGGTCTGGGTAGAAACAAATTCAGGGTTAATATATGCAGAAA
GAGTGAGAGGAGAAACAGGGGAAGAATTCAGAACGCAGGTATTAAGATGGTACGCCTTGTTTAAACCAGC
CTCAGTACAGTCCGATAATGGACCTGCATTTGTAGCAGACGCAACACAATTGCTAATGAAATACTTAGGG
ATTCAACATACAACAGGCATTCCATGGAATCCTCAATCACAAGCCTTGGTGGAAAGGACACATCAAACAT
TAAAACAAATGTTGCAAAAATTAGAAGGAAACTTTGTAGCATTTGAATCTGCCCTAGCAGCTGCCCTAAT
AGCGCTTAATATAAAAAGAAAGGGTGGGCTAGGGACAAGCCCTATGGATATATTTATATTTAATAAAGAA
CAACAGAGATTATTATTACAAGATCATGTAAATAAAGAAAAAATTCGATTTTGTTATTACAGGATCAGGA
AAAAAGGACACCCAGGTGAGTGGCTGGGGCCAACAAAGGTGCTCTGGGAAGGGGAAGGTGCCATTGTAGT
CAAAAATAAGGAACAAGACAGATATCTGGTGATAGCATATAAGGATGTCAAGTTTATACCGGCACCGAAA
GAATTGCAAAAAGAGTAG

>MG554403.1 Small ruminant lentivirus isolate SRLV002, complete genome
CTTCAAGAGGGGGCAACGTGTGGTGCCGTCCGCACCCCCTATGCTGTAACAGACGCACCACCTATAGTA
GAAGTGCAAGTAGGGACATGTTGGAAAAAATTATTAATAGATACAGGGGCTGATAGGACAATAGTAAAAC
ATCATGATAGTACGGGCATACCTAAAGGGAGAATAAAGTTACAGGGCATAGGAGGAATCATAGAGGGAGA
AAAATGGGAACAAGTAAAAATGAAGTACAGTGGGAAAGAAATAAAAGGAACAATAGTAGTATTGGCTAGT
AGCCCAGTAGAGGTGTTAGGGAGAGATAATATGGGAGAGTTAGGAATAGGGATAATAATGGCTAATTTAG
AAGAAGAAAAAATTCCTGTAACAGAAGTAAAGTTGAAGGAAGGATGTAAAGGACCTCATATACCACAATG
GCCATTAACACAAGAAAAATTGGAAGGATTAAGAGAAATAGTGGATAGATTAGAAAAGGAAGGGAAAGTA
GCGAGAGCACCTCCGCATTGGACGTGTAATACCCCAGTATTCTGCGTACTAAAGAAATCAGGAAAATGGA
GGATGCTAATAGATTTTAGAGAATTGAATAAACAAACAGAAGATCTAGCAGAAGCACAATTGGGACTGCC
GCATCCCGGAGGACTACAAAAGAGAAAGAATGTGACTATACTAGATATAGGGGATGCATATTTCACAATT
CCATTGTATGAGCCATATAGAAAGTATACATGTTTCACACTGTTAAGTCCTAATAATTTGGGACCATGTG
TTAGATATTATTGGAGAGTATTGCCTCAGGGATGGAAATTAAGTCCATCGGTGTATCAATTCACAATGCA
AAAAATATTATGGGATTGGATAAAAGCCCACCCTGAGGTACAATTTGGGATCTATATGGATGATATCTAT
ATCGGAAGTGATCTAGACATAAAAGAACATAGGGAAATAGTAGAGGAATTGGCCCAGTACATAGCGCAAT
ATGGATTCATGTTGCCAGAAGAAAAAAGGCAAGAAGGGTATCCAGCAAAGTGGCTAGGATTTGAGTTGCA
TCCTGAAAAATGGAAATTTCAGAGACATACCCTTCCTGAGCTTAAAGAAGGAAACATAACGTTAAACAAG
TTACAAAAATTGGTAGGAGATTTAGTATGGAGACAGACATTAATAGGAAAAAGTATACCAGAAATATTGA
AATTAATGGAAGGGGATAGAGCATTACAGAGTGAGAGGAGAATAGAAAGGAAACATGTACTAGAATGGGA
AGAATGTCGGAAAAAGTTAGAAGAAATGGCGGGAAATTACTATAATAAGGAAAAAGATGTGTATGGACAA
ATAGACTGGGGAAATAAAGCAATAGAATACATAGTGTTTCAGGAGAAAGGGAAACCTTTATGGGTAAACG
TAGTACACAATATAAAGAACTTAAGCCAAGCTCAACAAATTATCAAAGCAGCACAAAAATTAACGCAGGA
GGTGATAGTGAGAACAGGAAAAATACCATGGATCTTATTGCCAGGGAAAGAAGAGGATTGGAGGTTAGAG
CTACAAATAGGAAATATAACATGGATGCCCTCATTTTGGTCATGTTACAGAGGATCGGTAAGGTGGAAGA
GAAGAAACGTAGTAGAAGAAGTAGTCCCAGGACCAACATATTATACAGATGGAGGAAAGAAAAATGGGAA
AGGAAGCTTAGGATATATAGCCTCTACAGGAGAGAGGTACAGAGCCCATGAGGAAGGGACAAATCAGCAA
TTGGAATTAAGAGCAATAGAAGAAGCATGTAAACAGGGACCAGCAGAAATGAATATAGTCACAGACAGTA
GGTATGCTTATGAATTTATGTTGAGAAATTGGGATGAAGAAGTAGTAAAGAATCCCATACAGGCAAGAAT
TATGCAGTTGATACATAAGAAAAGGAAAGTAGGAATACATTGGGTGCCAGGACACAAAGGTATCCCGCAA

AATGAAGAGATAGATCAATATATATCAGAGATATTTTTAGCAATACAAGGAACAGGGATCTGTCCGAAAA
GAAGGGAAGATGCAGGGTATGACTTGATATGTCCACAGGAAATAAGCATACCGCCAGGACAGGCAGAAAG
AGTCCCTATAGATTTAAGACTAAATCTGAAAGAAAATCAATGGGCAATGATAGGGACAAAAAGTAGTGTC
GCGAGCAAAGGTGTATTCATACTAGGAGGAATAATAGACTCAGGGTATCAAGGAACAGTACAAGTAATAG
TATATAATGGTAGTGAGAAGGAGGTAGTCATACCTCAAGGAAGAAAATTTGCACAATTAATTCTCATGCC
ATTACAGCATGAGGAGTTAGAACCATGGGGCAAAACGAGAAAAACAGAAAGAGGAGAAAAAGGGTTTGGA
TCCACAGGAATGTATTGGATAGAAAACATTCCTGCGGCAGAGGAGGAACACGGAAAATGGCACCAAGATG
CAATGTCACTGCAGGTAGAATTTGGCATCCCTAGGACAGCAGCAGAGGATATAATACAACAATGTGAAGT
CTGCCAAGAAAATAAGGTGCCAAGTACACTCAGGGGAGGTAACAAAAGGGGAGTAGATCATTGGCAAGTA
GATTATACTCATTATGAGAATAAAGTTATATTAGTATGGATAGAAACAAACTCAGGATTAATATATGCAG
AGAAAGTAAAAGGAGAATCAGGAGAGGAATTCAGAACACAAGTAATAAAATGGTATGCCTTATTTAAGCC
AGCCTCAGTGCAGTCAGATAATGGACCTGCGTTTACAGCAGAAGCAACACAATTGTTAATGAAATATTTG
GGAATTCAACATACAACAGGCATTCCATGGAACCCCCAATCACAAGCATTGGTGGAAAGGGCCCATCAAA
CTTTGAAACAAACGCTACAAAAGTTAGAAGGAAATTTTAAAGCATTTGAATCTGCCCTAGCAGCCGCCCT
AATAGCAATTAATATAAAAAGAAAGGGTGGGCTAGGGGCAAGCCCTATGGATATATTTATATTTAATAAA
GAACAACAGAGATTATTATTACAAAATCAGGAAAATAAAGAAAAAATTCGATTTTGTTATTACAGGATAA
GAAAAAAAGGACACCCAGGTGAGTGGCTGGGGCCAACAAAGGTACTCTGGGAAGGGGAAGGTGCCATTGT
AGTTAAAAATAAGGAGCAAGACAGATATCTGGTGATAGCCTATAAGGATGTCAAGTTTATACCGGCACCA
AAAGAATTGCAAAAGGGGTA

>MG554407.1 Small ruminant lentivirus isolate SRLV006, complete genome
CATCAAGAGGGGGCCACGTGTGGTGCCGTCCGCACCCCCTATGCTGTAATGGACG
CACCACCCCTCGCAAGAATACAAGTAGGGACATGCTGGAAGGAATTGTTAATAGATACAGGGGCTGATAG
AACAATAGTAAAGCATCATGATAGTACAGGCATACCTAAAGGAAGAATAAAGCTACAGGGAATAGGAGGA
ATCATAGAGGGGGAAAAATGGGATCAAGTAAAAATTAGGTATAAAGGAAGGGAAATAAAAGGAACAATAG
TAGTATTGGCTAGTAGCCCGGTAGAAGTATTAGGCAGGGATAACATGGGAAAGTTAGGAATAGGGATAAT
AATGGCCAATTTAGAAGAAGGGAAAATTCCTGTGACGGAAGTAAAATTAAAGGAAGGATGTCCAGGACCC
CATATACCCCAATGGCCATTAACAAAGGAAAAACTTGAAGGATTGAGAGAAATAGTGGAAAGATTGGAAA
AAGAAGGAAAAGTAGCGAAGGCGCCTCCACATTGGACATGGAATACCCCCACATTCTGCACAAGAAAGAA
ATCAGGGAAATGGAGAATGTTAATAGATTTTAGAGAATTGAACAAACAGACAGAAGATCTAGCAGAGGCT
CAATTGGGACTGCCGCATCCAGGAGGATTGCAAAAGAAAAAGAATGTCACTGTATTAGATATAGGGGATG
CATATTTTACAATTCCTTTATATGAGCCATATAGAAAGTATACATGTTTCACATTGTTAAGTCCCAATAA
TGTGGGACCATGTGTGAGATATTATTGGAAAGTACTGCCTCAGGGATGGAAATTAAGTCCATCTGTGTAT
CAATTTACAATGCAAAAAATATTATGGGATTGGATAAAAGCCCACCCTGAGGTACAATTTGGGATCTATA
TGGATGATATTTATATAGGAAGTGATCTAGAGATAAAAGAACATAGGAAGAGAGTGGACGGATTAGCCCA
GTATATAGAGCAATATGGGTTCATGTTGCCAGCAGAAAAAAGACAAGAAGGGTATCCAGCAAAATGGCTA
GGATTTGAGTTGCACCCTGATAAATGGAAGTTTCAGAAACACAGCCTCCCGGAGCTTAAAGAAGGAACAA
TAACCTTAAATAAGTTACAAAAATTAGTAGGAGATTTAGTATGGCGACAGTCATTGATAGGAAAAAGTAT
ACCAGAAATATTGAAATTAATGGAAGGGGATAGAGAATTGCAGAGTGAGAGAAAAATAGAGAAAAAACAT
GTAGAAGAATGGGAACGGTGTAGGAAAAAATTAGAAGAAATGGAAGGAAATTATTACAATGAGGAAAAAG
ATGTTTATGGACAAATAGACTGGGGAAATAAAGCAATAGAATATATAGTTTTCCAGGAGAAAGGGAAACC
TTTGTGGGTAAATGTGGTGCACAATATAAAGAATTTAAGTCAAGCGCAGCAAATTATCAGAGCAGCACAA
AAATTAACACAAGAAGTAATAGTTAGAACAGGAAAACTGCCATGGATTTTATTACCAGGCAAGGAGGAAG
ATTGGATTTTAGAACTACAAATAGGGAACATAACATGGATGCCCTCATTTTGGTCATGTTATCGAGGGTC
AGTAAGGTGGAAGAGAAGAAATGTAGTCGAGGAAGTGGTTCCAGGGCCAACATATTATACAGATGGAGGA
AAGAAAAATGGGAAAGGAAGTTTAGGATATATAGCTTCTACAGGAGAAAGGTATAGGGCCCATGAGGAAG
GGACCAATCAGCAATTAGAATTAAGAGCAATAGAGGAAGCATGTAAACAGGGACCAGCAAAAATGAATAT
AGTAACAGATAGCAGGTATGCTTATGAATTTATGTTAAGAAATTGGGATGAAGAAGTCATAAAGAATCCC
ATACAGGCAAGAATTATGCAATTGGTACATAAGAAAGAGAAAGTAGGAATACACTGGGTACCAGGACATA
AAGGGATTCCTCAAAATGAAGAGATAGACAGATATATAGCAGAAATATTTTTAGCAGTACAAGGAACAGG
AATCTCCTCAAAAAGAGAGGAAGATGCAGGGTATGACTTGATATGCCCACAAGAAGTATGCATATCGCCA
GGACAGGTAACGAGAATACCTATAGATTTAAAAGTAAATCTGAAGGAGAATCAGTGGGCAATGATAGGGA
CAAAAAGTAGCTTCGCCAGCAAAGGAGTATTCATACAAGGAGGGATAATAGACTCGGGATATCAAGGAAC
AATACAGGTGGTAGTCTACAATAGTACAGATAAGGAGGTAATAATACCTCAAGGAAGAAAATTTGCACAA
ATAATTCTCATGCCATTAATACATGAAGAATTGGAACCATGGGGAAAAATGAGAAAAACAAAAAGGGGAG
AAAAAGGATTTGGGTCCACGGGAATGTATTGGATAGAAAATATTCCCGCAGCTGAAGAGGAGCATGCAAA
ATGGCATCAGGACGCGATGTCTCTGCAGTTAGATTTTGGCATTCCTAGAACAGCTGCAGAGGATATAATA
CAACAATGTGAAGTCTGCCAGGAAAATAAATTACCAAGTACTCTCAGAGGAGGAAATAGAAGGGGAGTAG
ATCATTGGCAAGTAGATTATACTCATTATGATAACAAAAGCATATTAGTATGGGTAGAAACAAATTCAGG
CTTGATATATGCAGAAAGAGTGAAAGGAGAAACAGGAGAAGAATTTAGAACACAGGTGTTAAAGTGGTAC
GCCTTATTCAAGCCCACCTCAGTGCAGTCGGATAATGGGCCAGCGTTCGTTGCAGACGCAACACAGTTGT

TAATGAAATATTTAGGGATTCAACACACAACAGGCATCCCATGGAATCCTCAGTCACAAGCTTTGGTAGA
GAGGACTCATCAAACCCTGAAGCACACTTTACAAAAACTGGAAGGAAATTTTGTAGCATTTGAATCTGCC
CTAGCAGCAGCCCTAATAACACTTAATATAAAAAGAAAGGGTGGCCTAGGGGCAAGCCCTATGGATATAT
TTATATTTAATAAAGAACAACAGAGATTATTATTTCAAAATCAAAATAAAGAAAAAATTCGATTTTGCTA
TTACAGGGTCAGGAAAAAAGGACACCCAGGAGAGTGGCTTGGCCCAACACAGGTATTGTGGACGGGGAA
GGAGCCATTGTAGTTAAAGATAAGGAACAGGACAGATATCTAGTAGTAGCATATAAGGATGTCAAGTTCA
TACCACCACCCAAAGAATTGCAAAAAACATAA

>KT898826.1 Small ruminant lentivirus isolate Jord1, complete genome
CTGCAAACAAAAAA
GAAGGGAACCACAAGGCGGACTACGGCAGGGAAACTTCAGCAGGGGGCCACGTGTGGTGCCGTCCGCACC
CCCTATGCAGTAACATCTGCACCACCCATGATACAGGTTCGGATCGGGACTCAAGAGAAAAAAATTTTAG
TCGATACAGGGGCCGATCGAACCATAGTAAAATGGCATGATGGAACAGGGACACCAAAGGGAAGAATAAT
GCTACAAGGCATAGGAGGAATAATAGAAGGGGAAAAGTGGGGACACGTCCCTATCACTTATAACGAAGAA
AAAATATTAGGGACCCTTGTGGTGCTGCAAAGCAGCCCGGTAGAAGTTTTAGGACGAGATAATATGGGAA
AGTTAGGGATAAAATTAGTTATGGCAAACTTAGAGGAAAAGAAAATTCCAATTACAAAAGTAAAGTTGAA
GGAGGGATGCAAAGGACCTCATATTCCTCAGTGGCCACTAACATTAGAAAAATTACAGGGGCTAGAAGAA
ATAATTAACAGACTGTTAGAAGAAGGAAAATTAGGAGAAGCGCCACCAGAATGGACATGTAATACCCCAA
TATTTTGTATAAAAAAGAAGTCGGGGAAGTGGAGAATGTTGATAGACTTCAGGGAATTAAATAAACAAAC
AGAAGATCTAACAGAAGCACAGCTGGGGCTACCGCACCCAGGGGGGTTACAAAAGAAAAAGAATGTAACA
ATATTAGATATAGGGGATGCATATTTTACAATTCCTTTGTATGAACCCTACCGGCAGTATACATGCTTTA
CTTTGTTAAGTCCAAATAATTTAGGACCATGTAAAAGATATTATTGGAAAGTACTGCCGCAAGGATGGAA
GCTGAGCCCCTCAGTGTATCAATTTACAATGCAAAAAATATTAAAGGACTGGATAACCGAGCATAAAGAA
ATACAATTTGGAATTTATATGGATGACATTTATATTGGAAGTGATTTACCAATAGAACAACATAGAGGGA
TAGTCCAACAACTGGCAAAGTATATAGAAAAATTTGGATTCTTGTTGCCCGAGGACAAAAGACAAGAAGG
ATATCCAGCAAAGTGGCTGGGATTTGAACTCCATCCGGAGAAATGGAAATTTCAAAAACATACTCTTCCA
GAATTACAAGAGGGAAATATTACTCTAAATAAATTACAAAAATTAGTGGGAGACTTAGTCTGGAGACAGT
CTCTAATAGGAAAAAGTATCCCTAAAATATTAAAATTAATGGAGGGAGATAGAGCACTACAAAGCGAGAG
ACAAGTTACTTGGGAGCATGTAAAGGAGTGGGGAGAATGTAAAAAGAAGCTACAACAAATGGAAGGGAAT
TATTATAATGCAGAGAAGGATATTTATGGACAACTAGATTGGGGAGAAAAAGCAATAGAATATATAGTCT
TTCAAGAGAGGGGAAAGCCAATGTGGGTGAATGTAGTCCATCAAATAAAAAACCTGAGTCTACCACAGCA
AGTAATTAAAGCAGCCCAAAAACTTACTCAAGAAGTCATTATTAGAACAGGAAAAATCCCATGGATTCTA
CTTCCAGGAAAAGAGGAAGATTGGATACTAGAAATGCAATCAGGAGGGATAAGTTGGCTACCCTCTTTTT
GGTCATGCTATAAAGGATCTGTGAGATGGAAAAGGAGAAATGTCATAGCAACAGTAGTAGCAGGGCCAAC
ATATTATACGGATGGGGGAAAGAAGAATGGACAAGGGAGTCTCGGATATATAGCCTCCACGGGAGAAAAA
TTTAGAAAACATGAACAAGGGACAAATCAACAATTAGAATTAAGGGCAATAGAAGAAGCGTGTAACCATG
GACCCCAAAGTATGAATATAGTAACAGATAGCAGATATGCCTACGAATTCTTATTAAGAAATTGGGATGA
AGAAGTAATAAAAAACCCTATACAAGCAAGAATAATGAAGAAGGTCCATGAAAAAGACTTGGTAGGAATA
CATTGGGTACCAGGGCACAGAGGGATTCCTCAAAATGAAGAAGTAGATAGATATATATCAGAAATTTTCT
TAGCAAAAGAAGGAACAGGAATTCTACCAAAAAGAGAAGAAGATGCAGGGTATGATCTTATATGTCCAAA
GGAAATTAGTATACCACCAGGGCAAGTACAAAAAGTGCCTATCGATTTAAAAATAAATTTAAAAAGAAAT
CAATGGGCCATGATAGGAACAAAAAGTAGTATGGCAGCTAAGGGAGTATTTACACAAGGAGGAATCATAG
ATTCAGGATATCAGGGACAGATACAGGTGATAGTGTACAACAGTAATGCAGTAGAAGTAGTAATTCCGGA
AGGAAGAAAATTTGCACAATTAATTATAATGCCATTAGTGCATGAAGAATTAGAACCATGGGGGGTAACA
AGAAAAACAGAAAGAGGAACACAAGGATTTGGATCTACTGGGGCATATTGGATAGAACAAATTCCACAAG
CAGAAGAAGAACATGCAAAATGGCATCAAGATGCAAATTCGTTGCACTTAACATTTGGGATACCAAAGTC
AGCTGCAGAGGACATTGTGCAACAATGTGACATATGTCAGGAAGGAAAACTTCCATCACCTGTGCGGGGA
GGAAACAAAAGGGGAATAGATCATTGGCAAGTAGATTATACCCATTATGAAAATCATATAGTCTTAGTGT
GGGTAGAAACAAATTCAGGCTTAATACACGCAGAAAGAGTAAAAGGAGAATCAGGGCAAGAATTTAGAAT
ACAAGTTTTAAAATGGTATGCGTTATACAATCCCGCCTCATTGCAGTCCGACAATGGACCAGCATTCGTG
GCAGAAGCAACCCAGTTACTAATGCAGTATCTTGGGGTACAGCATACAACAGGGATACCCTGGAATCCGC
AGTCACAAGCTTTAGTAGAAAGAACCCATCAAACATTAAAACACACCTTAAATAAGCTCAAAGCTCAGTT
TGCCTCAATAGATTCAGCACTAGCCGCAAGCTTAATCACTCTTAACATAAAAAGAAAGGGTGGGCTAGGG
ACAAGCCCTATGGACATATTTATTTATAATAAAGAACAAGAAAGATTACAATTACAGTTTAATAAAAATA
CAGAAAAAATTCAATTTTGTTATTACAGAAGCAGAAAAAGAGGACATCCAGGAGAGTGGATTGGTCCTAC
TCAGGTACTTTGGAGGGGGGAAGGAGCAATAGTAGTAAAGGATAAAGAGACTGAAAGGTATTTGGTAATC
CCTAATAAAGATGCAAAATTTGTTCCCCCGCCTAAAGAACTGTAA

>MG554402.1 Small ruminant lentivirus isolate SRLV001, complete genome
C
GGGAGACGGGGGATACGTGTGGTGCCGTCCGCTCCCCCTATGGAATAACTACAGCACCACCAAAGGTTCA

GGTCCGCATAGGTTCCCAATGGAGGAACTTATTATTTGATACAGGAGCGGACCGAACTATAGTAAGATGG
CATGATGGATCAGGAATTCCAGCAGGAAGAATAAAATTACAGGGAATAGGAGGAATAGTAGAAGGGGAAA
AATGGACTAATGTCTTGTTAGAATATAAAGAAAAAGTAATAAAAGGAACCATAGTAGTGTTACCTCAAAG
TCCAGTAGAAGTATTAGGAAGAGATAATATGGAGAAGTTTGATATAAAAATAATTATGGCAAATTTGGAA
GATAAAAAGATTCCTATTACGAAAGTAGCATTAAAAGAAGGATGTACAGGACCACATATCCCGCAATGGC
CTTTAACAGAAGAAAAGTTGAAAGGACTAACAGAAATAATAGATAAATTATTAGAAGAAGGAAAATTAGG
AAAGGCACCTCCACATTGGACATGTAATACACCAATATTTTGTATTAAGAAAAAGTCAGGAAAATGGAGA
ATGTTAATAGACTTTAGAGAATTAAATAAGCAAACAGAAGATTTAACAGAGGCACAATTAGGACTCCCGC
ATCCAGGAGGACTACAAAAGAAGAAACATGTCACAGTATTAGACATAGGGGATGCATATTTTACCATACC
ACTTTATGAGCCGTATCAACAGTATACATGTTTTACTCTGTTAAGCCCTAATAATTTGGGTCCGTGCAAA
AGGTATTATTGGAAAGTGCTGCCACAGGGATGGAAATTAAGTCCTTCTGTATATCAGTTCACCATGCAAA
AAATCTTAGAAGATTGGATACAGCAACACCCAGAGATTCAATTTGGTATATATATGGATGATATTTATAT
AGGAAGTGACTTAGAAATCAAAAGACATAGAGGAATAGTGGAAGAATTAGCAAACTACATTGCCCAATAT
GGATTCACACTCCCAGAAGATAAAAGGCAAGAAGGGCATCCAGCAAAATGGCTAGGATTTGAACTGCACC
CTCAAACATGGAAATTCCAGAAACATACATTACCAGAATTGAAAATAGGGACAATTACTTTAAATGAACT
ACAAAAATTAGTAGGGGAATTAGTGTGGAGGCAATCCATAATAGGGAAAAGTATACCCAACATCTTGAAA
TTAATGGAAGGAGATAGGGCATTGCAAAGTGAAAGAAGAATTGAGGAAACACATATAAAGGAATGGGAAG
AATGCAGGAAGAAGTTAGCAGAAGCAGAAGGGCATTATTTAGACACAGAAAAGGATGTCTATGGACAAAT
AGCATGGGGCGACAAAGCAATAGAATATATAGTCTATCAGGAAAAAGGAAAACCATTATGGGTAAACGTA
GTGCATAGCATAAAGAATTTAAGTATCCCACAACAAATCATTAAAGCAGCACAAAAGTTAACACAGGAAG
TAATTATAAGAACAGGAAAAATACCATGGATATTATTACCAGGAAAAGAGGAGGATTGGAGACTAGAATT
GCAATTAGGAAATATAACCTGGATGCCAAAATTTTGGTCATGCTACAGAGGACAGACTAGATGGAGGAAA
AGAAATATAACAGAAGAAGTGGTAGAAGGGCCCACATATTACACAGACGGAGGGAAAAAGAATAAGATAG
GAAGCATGGGATTTATAACCTCCAGAGGGGAGAAGGTTAGGAGACATGAAGATGGAACAAACCAGCAATT
GGAATTGAGGGCAATTGAAGAAGCTCTAAAGCATGGGCCATCCGTAATGAACATAGTAACAGATAGCAGA
TATGCATTTGAATTCTTATTGCGAAATTGGGATGAAGAAACCATTAAGAATCCAATTCAAGCAAGAATTA
TGGAAATAGCGCATAAAAAAGAAAAATTAGGAGTACATTGGGTACCAGGACATAAAGGGATCCCTCAAAA
CGAAGAAATAGATAAATATATATCAGAAATATTTCTAGCAAAAGAAGGAGAAGGAATTCTCCCGAAAAGA
GAGGAGGATGCCGGATATGATTTAATATGTCCACAAGAAACTAGTGTTGGGCCAGGGCAAGTAAGAAAAA
TCCCAATAGATTTAAAACTAAATTTAAAGGAATCACAGTGGGCCATGATTGCCACAAAAAGCAGCATGGC
GGCCAAAGGAGTATTCACACAAGGGGGAATCATAGATTCAGGATATCAAGGACAAATACAGGTAATAGTG
TATAATAGTAATAATATAGAAGTTGTCATACCTAAAGGAAGAAAATTTGCACAGTTAATATTAATGAATA
AAATACATGAAGAGTTGGAACCTTGGGGAATAACCAGGAAAACAGAAAGAGGAAAAGAAGGATTTGGATC
TACAGGAATGTATTGGATAGAAAACATCCCAATAGCAGAAGAAGAACATGCAAAATGGCATCAAGATGCC
CAGTCCTTACATTTAGAATTTGAAATTCCCAGGACAGCAGCAGAAGACATAGTTAACCAATGTGACATAT
GCCAACAAGAAAAGACACCCTCTGTCATAAGAGGAGGGAATAAGAGAGGGGTAGATCATTGGCAAGTAGA
TTATACCCATTATGAGAATAATATACTATTAGTTTGGGTAGAAACAAATTCAGGGTTAATATATGCAGAA
AAAGTGAAAGGAGAATCAGGACAAGAATTTAGAATAAGAACAATGCAATGGTATGCACTATTTGAGCCAG
AATCGCTACAGTCTGACAATGGACCTGCCTTTGTAGCTGAGCCCACACAGTTATTAATGAAATACCTAGG
AATTAAACATACAACAGGGATACCTTGGAACCCACAGTCTCAAGCCTTAGTAGAGCGAACTCATCAAACT
TTAAAACAAACGCTAAACAAGTTTAAAAACAACTTTGTAGCATTAGAATCAGCAATAGCAGCAGCCCTAG
TAGCAATAAATATAAAAAGAAAGGGTGGGCTAGGGACAAGCCCTATGGACATTTTTATATATAATAAAGA
ACAAAAAAGACTAAATAATAAATATAATAAAAATTCTGAAAAAATGCAATTTTGCTATTACAGAACACGA
AGGAGAGGACATCCAGGAAACTGGACAGGGCCAACCCAGGTACTGTGGAAAGGAGAAGGAGCCATAGTAG
TAAAAGATAACAGCTCAGAAAAATATTTAGTAATACCTAACAAAGATGCAAAATTTATCCCGCCGCCGAC
AAAGGGAGAAAAATAA

>MH936674.1 Small ruminant lentivirus strain BRMG CNPC gag polyprotein (gag), pol polyprotein (pol), vif protein (vif), and vpr-like protein (vpr-like) genes, complete cds; and env polyprotein (env) gene, partial cds

TTGTGGGAAAAGAGGGCATATGCAGAAAGATTGC
AGAGGAAAGAAGGATGCAAAAGGAAGGCAGCAGGGAAACGAGAGGAGGAGGCCACGTGTGGTGCCGTCCG
CTCCTCCTATGGAATAAATAATGCACCACTCATAGTTCAGGTCCGCATAGGGTCCCAAAAGAGGGACTTG
TTATTTGACACCGGGGCGGATCGGACTATAGTAAAATGGCATGATGGCTCGGGAACCCCAGCAGGAAGAA
TAAAATTACAAGGAATAGGGGGTCTAGTAGAAGGAGAAAAATGGAATAATGTAGAATTGGAATATAAAGG
AGAAATAAAAAAGGGAACAATAGTAGTGTTACCACAAAGTCCAGTAGAAGTACTAGGACGAGATAATATG
GCCAGATTTGACATAAAAATAGTAATGGCAAATTTAGAAGAAAAGAAAATCCCAATCACAAAAGTAAAAT
TGAAAGAGGGATGTACAGGACCGCATGTCCCACAATGGCCGTTAACAGAAGAAAAATTAAGAGGACTGAC
AGAAATAATAGATAAATTAGTGGAAGAAGGAAAACTAGGAAAAGCACCTCCACATTGGACATGTAATACT
CCAATATTTTGCATAAAAAAGAAGTCAGGAAAGTGGAGGATGTTAATAGATTTCAGAGAATTGAATAAAC

AAACAGAGGATTTAACAGAGGCACAGTTAGGACTTCCACATCCTGGGGGCCTACAAAAGAAAAAACATGT
TACAATATTAGACATAGGAGATGCATATTTCACTATACCCTTGTATGAACCCTATAGAGAGTACACATGT
TTTACTCTATTAAGTCCTAATAATTTGGGACCATGTAAAAGATATTATTGGAAAGTGCTGCCACAAGGGT
GGAAGCTGAGTCCTTCTGTATATCAATTTACTATGCAAGAAATTTTAGAGGATTGGATACAGCAACATCC
AGAAATTCAATTTGGAATATATATGGATGATATATACATAGGAAGTGATTTAGAAATTAAGAAGCATAGA
GAGATAGTGGAGAACTTAGCAAATTACATTGCCCTCTATGGATTTACCCTACCAGAAGAAAAGAGACAAG
AGGGGTATCCAGCAAAATGGCTAGGATTTGAATTACATCCCCAGACTTGGAAATTTCAAAAGCATACATT
GCCGGAATTAACAGTAGGGACGATTACATTAAATAAATTACAAAAATTAGTAGGAGAATTAGTATGGAGG
CAATCCATAATTGGGAAAAGCATCCCTAATATTTTAAAATTAATGGAAGGAGATAGGGCATTACAAAGTG
AAAGAAAAATTGAGGAAATACATGTAAAAGAATGGGAAGCATGTAGAAAGAAATTAGAAGAAATGGAAGG
AAATTATTATAATGAAGACAAGGATGTCTATGGACAATTGGCTTGGGGAGACAAAGCTATAGAATATATA
GTGTTTCAAGAAAAAGGAAAACCATTATGGGTAAATGTAGTTCATAATATAAAGAACTTAAGCATACCGC
AACAAATTATTAAGGGAGCGCAAAAGCTAACCCAAGAAGTAATCATTAGGACAGGGAAAATACCATGGAT
ATTACTGCCAGGGAAAGAAGAAGATTGGAGGCTGGAATTGCAATCAGGGAATATCACATGGATGCCAAAA
TTTTGGTCCTGCTACAGAGGACAGACTAGGTGGAAAAGAAGAAATATACTAGAAGAAGTAGTAGCAGGCC
CTACATATTATACAGATGGAGGGAAGAAGAATAAAGTCGGAAATTTGGGGTTCATAGCATCAACAGGAGA
AAAATTTAGAATACATGAAGAGGGTACAAATCAGCAACTTGAATTGAGAGCAATTGAAGAAGCTCTAAAA
CAAGGACCTCAAACAATGAATATAGTAACAGATAGTAGATATGCATTTGAATTTATGTTAAGAAATTGGG
ATGAAGAAGTAATAAAAAACCCAATTCAAGCAAGAATTATGGAAATCGCCCATAAGAAAGAAAAAATAGG
AGTGCATTGGGTGCCAGGACACAAAGGAATTCCCCAAAATGAGGAAATAGACAAATATATCTCAGAGATA
TTTCTTGCAAAAGAAGGAGAAGGGATTCTCCCAAAAAGAGAAGAAGATGCAGGGTATGATTTAATCTGCC
CTGAAGAGGTTGTCCTGGAGCCAGGCCAGGTAAAGTGCATTCCCATAGATTTGAGAATAAATTTGAAGGA
ATCACAATGGGCTATGATTGCTACAAAGAGCAGCATGGCTGTCAAAGGAGTATTTACACAAGGGGGAATC
ATAGATTCAGGATATCAGGGACAAATACAAGTAATAGTGTACAACAGCAATAAAGTAGAAGTGGTCATAC
CCCAAGGGAGGAAATTTGCACAATTAATATTAATGAATAAAATACATGAAGAATTAGAACCTTGGGGAGA
AAATAGGAAAACAGAAAGAGGGGAACAAGGATTTGGGTCCACAGGAATGTATTGGATAGAAAATATTCCC
CTAGCAGAAGAAGATCACACAAAATGGCATCAAGATGCTAGATCATTACACTTAGAATTTGAAATTCCCA
GAACAGCAGCAGAAGACATAGTAAATCAATGTGAAGTATGTCAAGAAGAAAAAACACCCTCCTTGATTAG
AGGCGGAAACAAAAGGGGGGTAGATCATTGGCAAGTGGATTATACCCATTATGAAAATATAATACTATTA
GTATGGGTAGAAACAAATTCAGGATTAATATATGCAGAGAAAGTGAAAGGAGAATCAGGACAAGAATTCA
GAACAAAAGTAATGCAATGGTATGCTCTATTTAGTCCAGAATCATTGCAATCAGACAATGGACCTGCATT
TGTAGCTGAGCCTACACAGCTACTAATGAAATACTTAGGAATCCAGCATACAACAGGGATACCTTGGAAT
CCCCAATCTCAAGCTATAGTGGAAAGAGCGCATCAATTACTAAAAAGTGCTCTAAAGAAATTTCAGCCAC
AATTTGTCGCTGTGGAATCAGCGATAGCCGCAGCCCTAGTCGCCATAAATATAAAAAGAAAGGGTGGGCT
AGGGACAAGCCCTATGGATATTTTTGTATATAATAAAGAACAGAAAAGATTAAGTAATAAATATAATAAA
AATTCTGAAAAAATTCAATTTTGCTATTACAGAGTAAGAAAAAGAGGACATCCAGGAGAGTGGAAAGGTC
CAACTCAGGTACTGTGGAAAGGGGAAGGAGCCATTGTAGTAAAAGACATAGAAAGTGAAAAGTATCTAGT
AGTACCTTACAAAGATGCAAAATTCATCCCGCCACCAACAAAAGAAAAGGAATAG

>JF502416.1 Caprine arthritis encephalitis virus isolate Fonni, complete genome
TTGCGGAAGGCGGGGGCATATTCAGAAAGATTGCAGAGGCAAAAGACAA
GGACAGCCGCAGGGAAACAAGGGGAGGGGGCCACGTGTGCTGCCGTCCGCACCCCCTATGGAATAACTGC
AGCACCCCCCATGATAGAAGTAAAAGTGGGAGGTCTAGAAAAGACCTTACTACTAGATACAGGAGCGGAT
AAAACAATAATAAAAAAGCACAATAATAAGGGTATACCAAATGGAAGAATAAAATTACAGGGAATAGGAG
GATTAGTGGAAGGAGAAAAATGGAAAAATGTAGAAATAGAATATAAAGGTTTAAAGGTAAAAGGAGAAAT
AGTAGTCATGACCCAAACTCCCATAGAAGTATTAGGAAGGGATAACATGGAGAAATTAGGGATAGGGATA
GTAATGGCAAATCTGGAGGAAGATAAAATACCCACCATAAAGGTAAAACTGAAAGAAGGATGTAAAGGAC
CACATGTACAGCAGTGGCCATTGACAGCAGAAAAATTACAAGGATTGACAGAAATTGTAGAAAAATTACT
AGGGGAAGGAAAAATAGGAGAAGCTCCACCTCATTGGACTTGGAATACCCCAATATTCTGTATAAAGAAG
AAATCAGGAAAATGGAGAATGTTGATAGATTTTAGAGAATTAAATAAGCAAACAGAAGATCTAACAGAGG
CACAATTAGGATTACCGCATCCAGGAGGATTGCAGAAAAAGAAAAACGTAACAGTTCTGGATATTGGAGA
TGCATATTTCACAATTCCATTATATGAACCATATAGAAAATATACATGTTTTACTCTGTTAAGCCCAAAT
AACTTAGGACCATGTAAGAGATATTATTGGAAAGTATTGCCCCAGGGATGGAAATTAAGTCCTTCTGTAT
ATCAGTTTACCATGCAGAGGATATTAAAAGATTGGATAAAAGATCATTCAGAAATACAATTTGGAATATA
TATGGATGATATATATATATAGGAAGTGATCTAGATAGAAAAAGCCATAGAGACATAGTAGAGAATTTGGCG
AAACACATAGCACAGTATGGATTTATGCTACCAGAAGATAAAAGACAAGAAGGGCATCCAGCAAAGTGGC
TAGGATTTGAGCTACACCCAGACACATGGAAATTTCAAAAACATGATTTGGCAGAACTTACGGAAGGGAA
AATAACGCTAAACAAATTGCAAAAATTAGTAGGGGATTTAGTCTGGAGACAATCATTGATAGGAAAGAGT
ATCCCAAAAATATTAAAATTAATGGAAGGAGATAGAGATTTACAAAGTACCCGAGAAGTCACAGCAGAGC
ATACACAAGAATGGGAAGCTTGTAGAAGGAAATTAAAAGAGATGGCAGGACACTATTATGATGAGGAGAA

AGATGTGTATGGACAGCTGACCTGGGGAAATAAAGCAATAGAATATATAGTGTTTCAAGAGAAAGGAAGA
CCGCTATGGGTAAATGTAGTACATCAGATAAAAAATCTAAGTCTACCACAACAAATTATTAAAGCAGTGC
AAAAGCTAACACAAGAAGTAATAATAAGAACGGGAAAAGTACCTTGGATAATGCTCCCAGGAAAGGAAGA
AGATTGGATATTAGAATTGCAAATGGGAAATATTACTTGGATGCCCGCATTCTGGTCATGTTATAGAGGA
GCGCCAAGATGGAGAAGAAGGAACATAGTAGAAGAGGTAGTAGAAGGACCAACATATTATACGGATGGAG
GAAAGAAAAAATGGACAGGGAAGCTTTGGATATATTTCATCCACCGGGGAAAAATTCAGAAAACATGAAAA
GGGCACAAATCAACAATTGGAACTGAGAGCCATAGAAGAAGTGTGTAAACGGGGACCAGAAAAAATTAAAT
ATCGTAACAGACAGCAGATATGCCTTTGAATTCATGCTAAGAGATTGGGATGAACAGGTCATAAAGAACC
CAATTCAGGCAAGAATAATGGAAATAGTACATAAAAAAGAAAAAATAGGAGTACACTGGGTGCCTGGACA
TAAGGGAATCCCTCAGAATGAGGAAATAGATAAATACATTTCAGAAGTATTCCTAGCAAAAGAGGGAGAA
GGAATACAACAAAAAAGACCAGAGGATGCAGGATATGACCTAATATGTCCTCAGGATGTAAGTATTGGAG
CAGGGGAGGTTAAGAAGATAGCAATAGACTTAAAATTAAATTTAAAAAAGGAACAATGGGCAATGATAGG
AACAAAAAGCAGCTTTGCAGCAAAAGGAGTATTCACACAAGGAGGAATCATAGATTCAGGATATCAAGGA
CAAATACAAGTAATTATATTCAACAGCAATAAATTTGAAGTAGTTATACCAAAAGGGAGAAAATTTGCAC
AATTAATACTCATGCCTCTGATACATGAAAACTTAGAGTCTTGGGGAAAGGATAGAAAAACAGAAAGAGG
GAAAAAAGGATTCGGATCAACAGGAGCCTTCTGGATAGAAAAAATCCCTGAAGCAGAGGAGGATCACTAT
AAATGGCACCAAGATGCAAAATCATTACACTGGGAATTTGGAATACCTATGTCTGCGGCAGAAGACATAG
TACAACAGTGTGAAGAATGTCAGCAAGAAAAACCAGCCTCGACTGTAAGAGGGGGAAATAAAAGGGGAAT
AAACCATTGGCAAGTCGATTATACCCACTATGAAGATATAATAATATTAGTATGGGTGGAAACTAATTCA
GGATTAATGTATGCAGAAAAAGTAAAAGATGAATCAGGGAAAGAATTTAGAATTCAGGCAATAAAATGGT
ATGCCCTGTTTAACCCAGAATCCGTGCAATCTGATAATGGGCCAGCATTTGTGGCAGAACCAACCCAACT
GTTAATGAAATACCTAGGAGTGCAGCATACAACAGGAATCCCATGGAATCCCCAATCGCAGTCAATAGTG
GAAAGGAGTCATAGAAGCTTTAAAAATACATTGAAAAAATTACAAAGTCAGTTCACAGCCATAGAATCGG
CAATAGCAGCAACCTTAATCACACTTAACATAAAAAGAAAGGGTGGGCTAGGGACAAGCCCTATGGAAAT
ATTTATATATAATAAAGAACAACAAAGAATAGATAAAAATAATCACTTAAAAAATCAAAACAAAAAATTT
TGTTATTACAGAGTGAGAAAAAGAGGCCACCCAGGACCGTGGGAAGGGCCCACAGAAGTGCTGTGGCAAG
GAGAAGGTGCCATAGTAGTAAAAGATAAGTATTCAGAAAGGTATCTAGTAATAGCACATAAAGATGCAAA
ATTTATTCCGCCGCCAACAGAGACGAAGAGAAAGGATCATAGGGCCACAATTACCTCTTAG

>MH374288.1 Small ruminant lentivirus isolate SRLV042, complete genome
CGGGAGACGGGGGA
TACGTGTGGTGCCGTCCGCTCCCCCTATGGAATAACTACAGCACCACCAAAGGTTCAGGTCCGCATAGGT
TCCCAATGGAGGAACTTATTATTTGACACAGGAGCGGATCGAACTATAATAAGAAGGCATGATGGATCAG
GGAATCCAGCTGGAAGAATAAAGCTACAAGGAATAGGAGGAATAGTAGAAGGAGAGAAATGGGATAATGT
AGAATTAGAATATAAAGGGGAAATAAGAAGAGGGACTATAGTGGTATTACCTCAAAGTCCAGTGGAAGTA
TTAGGAAGAGATAACATGGAAAGGTTTAACATAAAAATAATTATGGCTAATTTGGAAGATAAAAAGATTC
CTATTACAAAAGTAGCATTAAAAGAAGGATGTACAGGGCCACATAGTCCACAATGGCCTTTAACAGAAGA
AAAGTTGAAAGGATTAAAAGAAATAATAGATAAATTATTAGAAGAAGGAAAATTAGGAAAGGCACCGCCA
CATTGGACATGTAATACGCCAATATTTTGTATTAAGAAAAAGTCAGGAAAGTGGAGGATGTTAATAGACT
TTAGAGAATTAAATAAGCAAACAGAAGATTTAACAGAAGCACAATTAGGACTCCCGCATCCAGGAGGACT
TCAAAAGAAAAAACATGTCACAGTATTAGACATAGGGGATGCGTATTTTACTATACCACTTTATGAGCCA
TATCAACAGTATACATGTTTTACTTTGTTAAGCCCTAATAATTTGGGCCCATGCAAAAGGTATTATTGGA
AAGTGCTGCCACAGGGATGGAAATTAAGTCCTTCTGTATATCAATTCACCATGCAAAAAATATTAGAAGA
TTGGATACAGCAACACCCAGAGATTCAATTTGGCATATATATGGATGATATTTATATAGGCAGCGACTTA
GAAATTAAAAAACATAGAGGGATAGTAGAAGAATTAGCAAACTACATTGCCCAATATGGATTCACACTCC
CAGAGGAGAAAAGACAAGAGGGATATCCCGCAAAATGGTTAGGATTTGAACTGCACCCACAAACATGGAA
ATTTCAAAAACATACATTACCAAAATTAGAAATAGGGACAATTACTTTAAATAAACTGCAGAAATTAGTA
GGAGAACTAGTGTGGAGGCAATCTATAATAGGAAAAAGTATTCCCAACATCTTGAAATTGATGGAAGGAG
ATAGGGCATTGCAAAGTGAAAGAAGAATTGAGGAAACACATATACAAGAATGGGAAGAATGCAGGAAAAA
ATTAGCAGAAGCAGAAGGACATTATTTAGACACAGAAAAAGATATCTATGGACAAATAGCATGGGGCAAC
AAAGCAATAGAATATATAGTCTATCAAGAAAAAGGAAAACCATTATGGGTAAATGTAGTACATAGCATAA
AGAATTTAAGTATCCCACAACAGATCATTAAAGCAGCACAGAAATTAACACAGGAAGTAATTATAAGAAC
AGGAAAAATACCATGGATATTATTACCAGGAAAAGAGGAGGATTGGAGATTAGAATTGCAATTAGGAAAT
ATAACCTGGATGCCAAAATTTTGGTCATGCTACAGAGGACAAACTAGATGGAGAAAAAGAAATATAACAG
AAGAAGTGGTAGAAGGGCCTACATATTACACAGATGGGGGGAAAAAGAATAAGACAGGAAGCATGGGATT
TATAACTTCCATGGGGGAGAAGGTGAGGAAACATGAAAACGGAACAAATCAGCAACTTGAATTGAGGGCA
ATTGAAGAAGCTCTGAAGCATGGGCCACCTGTAATGAACATAGTAACAGATAGCAGATATGCATTTGAAT
TCTTATTGCGAAATTGGGATGAAGAAACTATTAGAAATCCAATTCAAGCAAGAATTATGGAAATAGCGCA
TAGAAAAGAAAAAATAGGGGTACATTGGGTACCAGGACATAAAGGGATCCCTCAAAATGAAGAAGTAGAT
AAATACATAGCAGAAATATTTCTAGCAAAAGAAGGAGAAGGGATTCTCCCGAAAAGAAGAGAGGATGCAG
GATATGATTTAATATGTCCACAAGAAGTTAGTGTCGGGCCAGGACAAGTAAGAAAAATCTCAATAGATCT

AAAACTAAATTTAAAAGAATCACAGTGGGCCATGATTGCTACAAAAAGCAGCATGGCGGCCAAAGGAGTA
TTTACACAGGGGGGAATCATAGATTCAGGATATCAAGGACAGATACAGGTAATAGTGTATAATAGTAATA
ATGTAGAAGTTGTTATACCTAGAGGAAGAAAATTTGCACAGTTAATACTAATGAATAAAATACATGAAGA
ATTGGAACCTTGGGGAATAACAAGAAAAACAGAAAGGGGAAAAGAAGGATTTGGATCTACAGGAATGTAT
TGGATAGAAAACATCCCAATAGCGGAAGAAGAACATGCAAAATGGCATCAAGATGCCCAGTCCTTACACT
TAGAATTTGAAATTCCCAGAACAGCAGCAGAAGACATAGTTAACCAATGTGACATATGTCAACAAGAAAA
GACACCCTCTATCATAAGAGGAGGGAATAAGAGAGGGGTAGATCATTGGCAAGTGGATTATACCCATTAT
GAAAATAATATATTATTAGTATGGGTAGAAACAAATTCAGGCTTAATATATGCAGAAAGAGTAAAAGGAG
AATCAGGACAAGAATTTAGAATAAAAACAATGCAATGGTATGCACTGTTTAAGCCAGAGTCACTACAGTC
TGACAATGGACCTGCCTTTGTAGCTGAGCCCACACAATTATTAATGCAATACCTAGGAATTAAACATACC
ACAGGGATACCTTGGAATCCACAGTCTCAAGCTTTAGTAGAGCGAACTCATCAAACTTTAAAACAAACAT
TAAACAAGTTTAAAAACAACTTTGTAGCATTAGAATCAGCAATAGCAGCCGCCCTAGTAGCAATAAATAT
AAAAAGAAAGGGTGGGCTAGGGACAAGCCCTATGGACATTTTTATATATAATAAAGAACAGAAAAGATTA
AATAATCAATACAATAAAAATTCTGAAAAAATGCAATTTTGCTATTACAGAACAAGAAAGAAAGGACATC
CAGGAGAGTGGACAGGGCCGACACAGGTACTGTGGAAAGGAGAAGGGGCCATAGTAGTAAAAGACAAAGG
CTCAGAGAAGTATTTGGTAATACCTAACAAAGATGCAAAATTCATCCCGGAGCCGACAAAGGGAGAAAAA
TAA

>MG554411.1 Small ruminant lentivirus isolate SRLV014, complete genome
CGGGAAGCGG
GGGATACGTGTGGTGCCGTCCGCTCCCCTTATGGAATAACTTCAGCACCACCTATGGTTCAGGTCCGCAT
AGGTTCCCAGTGGAGGAACTTATTATTTGATACCGGGGCGGACCGAACTATAGTAAAATGGCATGATGGC
TCGGGAACCCCAGCCGGAAGAATAAAATTACAAGGAATAGGTGGGATAGTGGAAGGAGAAAAATGGAAAA
ATGTAGAATTGGAATATAAAGGAGAAAAAAGAAGAGGAACAATAGTAGTATTACCACAAAGTCCAGTAGA
AGTACTAGGACGAGATAACATGACTCTATTTGAAGTAAAAATCATAATGGCAAATTTGGAAGAAAAGAAA
ATTCCTATTACAAAAGTAAAATTGAAAGAGGGATGTACAGGTCCTCATGTCCCGCAATGGCCCTTAACAG
AAGAAAAGTTAAAGGGTTTAACAGAAATAATAGATAAATTAGTAGAAGAAGGAAAACTAGGAAAAGCACC
TCCACATTGGACATGTAATACTCCGATCTTTTGTATAAGAAAGAAATCAGGAAAGTGGAGAATGCTAATA
GATTTCAGAGAATTGAACAAACAAACAGAAGATTTAACAGAGGCACAATTGGGACTCCCACATCCTGGGG
GATTGCAGAAGAAGAAAAATGTTACAATATTAGACATAGGAGATGCATATTTTACTATACCCTTGTATGG
ACCATATCAAGAATATACATGTTTTACTCTATTAAGTCATAATAATCTGGGACCATGTAAAAGATATTAT
TGGAAAGTCTTACCGCAAGGGTGGAAATTGAGTCCTTCTGTATATCAATTTACCATGCAAAAAATATTAG
AAGATTGGATACAGCAACATCCTGAGATTCAATTTGGAATATATATGGATGATATTTACATAGGAAGTGA
TTTAGAAATAAAAAAGCATAGAGAAATAGTAGAGGAACTAGCAAATTATATTGCCCAGTATGGATTTACC
CTGCCAGAAGAAAAAAGACAAGAGGGATATCCAGCAAAATGGCTAGGATTTGAATTACATCCTCAGACAT
GGAAATTTCAGAAACATACTTTACCTGAGTTAAAAGAAGGGATAATTACATTAAACAAATTGCAAAAATT
AGTAGGAGAATTAGTATGGAGGCAATCCATAATTGGAAAGAGCATTCCCAATATCTTAAAATTAATGGAA
GGGGATAGAGCATTACAAAGTGAAAGAAGAATTGGAGCCATACATGTGAAAGAATGGGAAGCATGTAGAA
AAAAGTTAGAAGAAATGGAAGGAAATTACTATGATGCAGAAAAAGATATCTATGGACAGCTAGCTTGGGG
AGACAAAGCTATAGAGTATATAGTGTATCAAGAGAAAGGGAAACCATTATGGGTAAATGTGGTTCACAAT
GTGAAGAACTTAAGCATCCCACAACAAATTATTAAAGCTGCGCAAAAGTTAACACAAGAAGTAATTATTA
GAACAGGAAAAATACCATGGATATTATTGCCAGGGAGAGAAGAAGATTGGAGATTAGAATTGCAATTAGG
AAACATCACATGGATGCCAAAATTTTGGTCATGTTATAGAGGGCAAACTAGATGGCAAAAGAGAAATATA
ATAGAAAGTGTAGTAGAGGGACCTACCTACTATACAGATGGAGGAAAAAAGAATAAAATAGGAAGCTTAG
GGTTCATAGCCTCAACAGGGGAAAAATTTAGAAAGCATGAAGAGGGCACAAATCAGCAACTTGAACTAAG
GGCTATTGAAGAAGCTCTAAAACATGGTCCTCCAACAATGAATATAGTAACAGATAGTAGATATGCATTT
GAATTCATGTTAAGAAATTGGGATGAAGAAGTAATTAAAAATCCAATTCAAGCAAGAATTATGAGTATAG
CCCATAAAAAGAACATGATAGGAGTGCATTGGGTGCCTGGACATAAAGGAATCTCACAAAACGAAGAAAT
AGATAGATATATCTCAGAGATATTCTTAGCAAAAGAAGGGGAAGGAATCCTTCCAAAAAGAGAGGAAGAT
GCAGGATATGACTTGGTATGTCCAAAAGAGGTCACTATAGGACCAGGCCAAGTAGAAAAAATCCCCATAG
ATCTAAGAATAAATTTGAAGGAATCGCAATGGGCTATGATTGCTACAAAGAGCAGCATGGCTGCCAAAGG
AGTGTTTACACAAGGAGGTATCATAGATTCAGGATATCAGGGACAAATACAAGTAATAGTGTACAATAGC
AATAATGCAGCAGTAGTCATACCTCAAGGAAGAAAGTTTGCACAGTTAATCTTAATGGATAAAAAACATG
AAGAATTAGAACCTTGGGGAACAAGCAGAAAAACAGAAAGAGGGGAAAAAGGATTTGGATCCACAGGGCT
GTATTGGATAGAGAATATTCCTCTGGCAGAAGAGGAACACTCAAAATGGCATCAAGATGCTCGATCATTA
CACCTAGAATTTGAAATCCCCAGAACAGCAGCAGAAGACATAGTAAATCAATGTGAGGTATGTAGGGAAG
AAAAATCACCGTCTTTAATCAGAGGGGGAAACAAAAGGGGAGTAGATCATTGGCAAGTAGATTATACCCA
TTATGAAACCATAATACTATTAGTATGGGTAGAGACAAATTCAGGGCTAATATATGCAGAAAAAGTAAAA
GGAGAATCAGGACAGGAATTCAGAATAAAAGTGATGCAATGGTATGCCTTATTTAGTCCAGAGTCCCTGC
AGTCAGATAATGGACCTGCCTTTACAGCAGAAGCCACGCAGCTGTTAATGAAATACTTAGGGATAAAACA
TACAACAGGCATACCTTGGAATCCACAGTCTCAAGCAATAGTAGAAAGAGCACATCAACTATTGAAAAGT

GCATTGAAGAAATTTCAGCCGCAATTTGTAGCTGTGGAATCAGCTCTAGCAGCCGCCCTAGTCGCAATAA
ATATAAAAAGAAAGGGTGGGCTAGGGACAAGCCCTATGGATATTTTTGTATATAATAAAGAACAGAAAAG
AATAAGTAATAAATATAATAAAAATTTTGAAAAAATTCAATTCTGTTATTACAGAGTAAGAAAAAGAGGA
CTTCCAGGAGAGTGGAAAGGACCAACCCAGGTACTGTGGAAAGGGGAAGGTGCCATTGTCGTAAAAGATA
CAGAGAGTGAAAAGTATTTAGTAATACCTTACAAAGATGCAAAATTCATCCCGCCGCCAACAAAAGAAAA
GGAATAA

>KT749878.1 Caprine arthritis encephalitis virus isolate Shandong, complete genome
ATGTCA
CAATTGTGGAAAAAGAGGACATATGCAAAAAGATTGCAGGGGAAAGAAAGAAATGGGAAGGCAGCAGGGA
AACGGGAGGAGGGGGATACGTGTGGTGCCGTCCGCTCCTCCTATGGAATAACTTCAGCACCACCAATGGT
TCGGGTCCGCATAGGTTCCCAGCAGAGGGACTTATTATTTGATACCGGGGCGGACCGAACTATAGTAAAA
TGGCATGATGGATCGGGAAACCCAGCTGGAAGAATAAAACTGCAAGGAATAGGGGGGATAGTAGAAGGGG
AAAAATGGAATAATGTAATATTGGAATATAAAGGAGAAACAAGAAAGGGAACAATAGTAGTGTTACCACA
AAGTCCAGTAGAAGTATTAGGACGAGATAACATGGCCCGATTTGGCGTAGAGATACTCATGGCAAATTTA
GAAGAAAAGAGAATCCCAATTACAAAAGTAAAATTGAAAGAGGGATGTACGGGTCCTCATGTCCCACAAT
GGCCATTAACAGAGGAAAAATTAAAAGGCCTAACAGAAATCATAGACAAATTAGTGGAAGAAGGAAAACT
AGGGAAGGCACCCCCACATTGGACATGCAATACTCCAATATTTTGTATAAAAAAGAAATCAGGAAAGTGG
AGAATGTTAATAGATTTCAGAGAATTGAACAAACAGACAGAAGATTTAACAGAAGCGCAGTTAGGACTAC
CACATCCGGGAGGACTACAAAAGAAAAAACATGTTACAATATTAGACATAGGAGATGCATATTTTACTAT
ACCCCTATATGAACCCTATAGAGAGTACACATGTTTTACTTTATTAAGTCCTAATAATCTAGGACCATGT
AAAAGATACTATTGGAAAGTGCTGCCACAAGGTTGGAAACTGAGTCCATCTGTATATCAGTTTACCATGC
AGGAAATTTTAGAGGATTGGATACAACAGCATCCTGAAATTCAATTTGGAATATACATGGATGATATTTA
CATAGGAAGTGATTTAGAAATAAAAAGGCACAGAGAAATAGTGAAAGAATTAGTCAATTATATTGCCCAA
TATGGATTCACCCTACCAGAAGAAAAGAGACAAGAGGGATATCCTGTCAAATGGCTAGGATTTGAACTAC
ACCCTCAGACCTGGAAATTTCAGAAGCATACCTTACCTGAATTAACAAAAGGGATCATTACATTAAATAA
ATTGCAAAAATTAGTAGGGGAGTTAGTATGGAGACAATCCATAATTGGAAAAAGCATTCCTAACATTCTG
AAATTAATGGAAGGGGATAGAGAATTACAAAGTGAAAGAAAAATTGAAGAAGTACATGTGAAAGAATGGG
AAGCATGTAGGAAAAAATTAGAAGAAATGGAAGGAAATTATTATAATGAAGAAAAAGATGTCTATGGACA
ATTGGCTTGGGGAGACAAAGCTATAGAATATATAGTGTATCAGGAAAAAGGGAAACCGTTATGGGTAAAT
GTGGTTCACAATATAAAAAACCTGAGCATCCCGCAACAGATTATTAAAGCAGCACAAAAACTAACACAAG
AAGTAATTATTAGGACAGGGAAAATACCATGGGTATTACTGCCAGGGAAAGAAGAAGATTGGAGACTAGA
ATTGCAATTAGGGAACATCACATGGATGCCAAAATTTTGGTCCTGTTATCGAGGACATACAAGATGGAGG
AAAAGAAACATAATAGAAGAAGTAGTAGCAGGACCTACATATTATACAGATGGAGGAAAAAAGAATAAAA
TGGGAAGCTTAGGGTTCATAGCATCAACAGGGGAAAAATTTAGAAAGCATGAAGAAGGCACAAATCAGCA
ATTAGAATTAAGAGCCATAGAAGAAGCTCTAAAACAAGGGCCTCCAACAATGAATTTAGTAACAGATAGT
AGATATGCATTTGAATTTTTATTAAGAAACTGGGATGAAGAAGTAATAAGAAATCCAATTCAAACAAGAA
TTATGGAACTTGCCCACAAGAAAGAAAAGATAGGAGTGCATTGGGTGCCAGGACACAAAGGAATTCCTCA
AAATGAAGAAATAGATAGATATATCTCAGAAATATTCTTGGCAAAAGAAGGAGAAGGAATTCTCCCAAAA
AGAGAAGAAGATGCAGGATATGATTTAATATGTCCAGAAGAAGTTACCATAGAACCAGGACAAGTGAAAT
GCATTCCCATAGATTTAAGAATAAATTTAAAGAAATCACAATGGGCTATGATTGCTACAAAAAGCAGCAT
GGCTGCCAAAGGCGTATTCACACAAGGAGGAATCATAGACTCAGGATATCAGGGACAAATACAGGTAATA
ATGTATAATAGCAATAAAATAGCAGTAGTCATACCCCAAGGGAGAAAGTTTGCACAATTAATATTAATGG
AGAAAATGCATGAAGAATTGGAACCCTGGGGGAAAAGCAGAAAAACAGAGAGGGGAGAAAAAGGATTTGG
GTCCACAGGAATGTATTGGATAGAAAATATTCCTTTGGCAGAAGAAGACCACACAAAATGGCATCAAGAT
GCCAGATCATTGCATCTAGAATTTGAAATTCCAAGAACAGCAGCAGAAGACATAGTAAACCAATGTGAAA
TATGTCAAAAAGAAAGGACACCTGCCGTGATCAGAGGAGGAAACAAAAGAGGGGTAGATCACTGGCAAGT
AGATTATACCCATTATGAAAATATCATACTATTAGTATGGGTAGAAACAAATTCAGGATTAATATATGCA
GAGAAAGTAAAAGGAGAATCAGGACAAGAATTCAGAATAAAAGTGATGCAATGGTATGCATTATTTGGTC
CAGAGTCATTGCAGTCAGACAATGGACCTGCATTTACAGCAGAGCCCACACAGCTGTTGATGCAATACTT
AGGAATAAAACACACAACCGGGATACCTTGGAATCCACAGTCTCAGGCTATAGTAGAAAGGGCACATCAA
CTATTAAAAAGTGCTTTAAAGAAATTTCAGCCACAATTTGTCGCTGTAGAGTCAGCAATAGCAGCAGCCC
TAGTCGCCATAAATATAAAAAGAAAGGGTGGGCTAGGGACAAGCCCTATGGATATTTTTATATATAATAA
AGAACAGAAAAGAATAAGTAATAAATATAATAAAAATTCTCAAAAAATTCAATTCTGTTATTACAGAATA
AGGAAAAGAGGACATCCAGGAGAGTGGAAAGGACCAACTCAGGTACTGTGGAAAGGGGAAGGAGCAATTG
TGGTAAAGGATACAGAAAGGCAAAAGTATTTAGTAATACCTTACAAAGATGCGAAATTCATCCCGCCACC
AACAAAGGAGAAGGAATAG

>MG554412.1 Small ruminant lentivirus isolate SRLV016, complete genome
CGGGAAGCGGGGGATACGTGTGGTGCCGTCCGCTCCCCCTATGGAATAACTTCAGCACCA

CCTATGGTTCAGGTCCGCATAGGTTCCCAGTGGAGGAACTTATTATTTGATACAGGGGCGGACCGAACTA
TAATAAAATGGCATGATGGATCGGGAACCCCAGCCGGAAGAATAAAATTACAAGGAATAGGTGGGATAGT
TGAAGGAGAAAAATGGAAAGATGTAGAATTGGAATATAAAGGAGAAAAAAGAAGAGGAACAATAGTAGTG
CTACCACAAAGTCCAGTGGAAGTACTAGGACGAGATAACATGACCCGATTTGACGTAAAAATAATCATGG
CAAACTTAGAAGAAAAGAAAATTCCTATTACAAAAGTAAAATTAAAAGAGGGATGTACAGGCCCTCATGT
CCCGCAATGGCCCTTAACAGAAGAAAAGTTAAAGGGGCTAACAGAAATAGTAGATAAATTAGTGGAAGAA
GGAAAACTAGGAAAAGCACCTCCACATTGGACGTGTAATACTCCAATCTTTGCCATAAAAAAGAAATCAG
GAAAATGGAGAATGCTAATAGATTTTAGAGAATTGAATAAACAGACAGAAGATTTAACAGAGGCACAATT
GGGGCTCCCACATCCGGGAGGATTGCAGAGGAAGAAAAATGTTACAATATTGGACATAGGRGATGCATAT
TTTACTATACCCTTGTATGAACCATATCGAGAATACACCTGTTTTACATTATTAAGTCCTAACAATTTAG
GACCATGTAAAAGATATTATTGGAAGGTACTGCCACAAGGGTGGAAACTGAGTCCTTCTGTATATCAGTT
CACCATGCAAAAAATATTAGAAGATTGGATACAGCAACATCCTGAGATTCAATTTGGAATATATATGGAT
GATATTTACATAGGAAGTGATTTGGAAATAAAAAAGCATAGAGAAATAGTAGAAGAATTAGCAAATTATA
TTGCCCAATATGGATTTACCCTGCCAGAAGAAAAAAGACAAGAGGGATATCCAGCAAAGTGGCTAGGATT
TGAATTACACCCACAGACATGGAAATTTCAGAAACATACTTTACCCGAGTTGAGAGAAGGAATAATTACA
TTAAACAAATTGCAGAAATTAGTGGGAGAGTTAGTATGGAGACAGTCCATAATTGGAAAGAGCATTCCCA
ACATCTTAAAATTGATGGAAGGGGATAGAGCATTACAAAGTGAAAGAAAAATTGAAGCCATACATGTGAA
AGAATGGGAGGCATGTAGAAAAAAGTTAGAAGAAATGGAAGGAAATTACTATGATGAAGAAAGAGATATC
TATGGACAGCTAGCTTGGGGTGACAAAGCTATAGAATACATAGTATATCAAGAAAAAGGGAAACCCTTAT
GGGTAAATGTGGTTCACAATGTAAAGAATCTAAGCATCCCGCAGCAAATTATTAAAGCCGCACAAAAGCT
AACGCAAGAAGTAATTATTAGAACAGGAAAAATACCATGGCTATTATTACCAGGGAAAGAAGAAGATTGG
AGATTAGAATTGCAATTAGGGAACATCACATGGATGCCAAAATTTTGGTCATGCTATAGAGGGCAAACTA
GATGGAAAAAGAGAAATATAATAGAAAGTGTAGTAGAGGGACCTACCTACTATACCGACGGAGGAAAAAA
GAATAAAATGGGAAGCTTAGGGTTCATAGCCTCAACAGGGGAAAAATTTAGAAAGCATGAAGAAGGCACA
AACCAGCAACTTGAACTAAGGGCTATTGAAGAAGCTCTAAAGCAAGGGCCTCAGACTATGAATATAGTAA
CAGATAGTAGATATGCATATGAATTCATGTTAAGAAATTGGGATGAAGAAGTAATTAAAAATCCAATTCA
AGCAAGAATTATGAGTATAGCCCATAAAAAGAACAGGATAGGAGTACATTGGGTACCCGGACATAAAGGA
ATCCCACAAAATGAAGAAATAGATAGATATATCTCAGAGATATTCTTAGCAAAAGAAGGAGAAGGAATCC
TTCCAAAAAGAGAGGAAGATGCTGGATATGACTTAATATGTCCAAAAGAAGTCAGTATAGGGCCAGGACA
GGTGGAAAAAATCCCCATAGATCTAAGACTAAATTTAAAGGAATCACAATGGGCTATGGTTGCTACAAAA
AGCAGCATGGCTGCCAAAGGAGTGTTTACACAAGGGGGAATCATAGATTCAGGCTATCAGGGACAAATAC
AAGTAATAGTATACAACAGCAATAATGTAGCAGTGGTCATACCCCAAGGAAGAAAGTTTGCACAATTAAT
ATTAATGGATAAAAAACATGAAGAATTAGAACCTTGGGGAACAAGCAGAAAAACAGAAAGAGGGGAAAAA
GGATTTGGATCCACAGGGCTGTATTGGATAGAAAATATTCCCTTGGCAGAAGAAGAACACTCAAAATGGC
ATCAAGATGCTCGATCACTGCACCTAGAATTTGAAATCCCCAGAACTGCAGCAGAAGACATAGTAAATCA
ATGTGAAGTATGTAGGGAAGAAAAATCACCGTCGTTAATCAGAGGGGGCAACAAAAGGGGAGTAGATCAT
TGGCAAGTAGATTATACCCATTATGAAAACATAATACTATTAGTATGGGTAGAAACAAATTCAGGGCTAA
TATATGCAGAAAAAGTAAAAGGAGAATCAGGACAAGAATTCAGGATAAAAGTGATGCAATGGTATGCATT
ATTTAGTCCAGAGTCACTGCAGTCAGATAATGGACCCGCATTTACCGCTGAGGCTACGCAGCTGTTGATG
AAATACTTAGGGATAACACATACAACAGGCATACCTTGGAATCCACAGTCTCAAGCAATAGTAGAAAGAG
CACATCAACTCTTGAAAAGTGCATTGAAAAAATTTCAGCCTCAATTTGTAGCTGTAGAATCAGCTCTAGC
AGCCGCCCTAGTCGCAATAAATATAAAAAGAAAGGGTGGGCTAGGGACAAGCCCTATGGATATTTTTGTA
TATAATAAAGAACAGAAAAGATTAAATAATAAATATAATAAAAATTTTGAAAAAATTCAATTCTGCTATT
ACAGAGTAAGAAAAAGAGGACTTCCAGGAGAGTGGAAAGGACCAACTCAGGTACTGTGGAAAGGGGAAGG
AGCCATTGTGGTAAAGGATACAGAGAGTGAAAAGTATTTAGTAATACCTTACAAAGATGCAAAATTCATC
CCGCCGCCAACAAAAGAAAAGGAATAA

>KT214469.1 Caprine arthritis encephalitis virus isolate Sichuan, complete genome
ATGTCACAATTGTGGAAAAAGAGGACATATGCAAAAAGA
TTGCAGGGGAAAGAAAGAAATGGGAAGGCAGCAGGGAAACGGGAGGAGGGGGATACGTGTGGTGCCGTCC
GCTCCTCCTATGGAATAACTTCAGCACCACCAATGGTTCGGGTCCGCATAGGTTCCCAGCAGAGGGACTT
ATTATTTGATACCGGGGCGGACCGAACTATAGTAAAATGGCATGATGGATCGGGAACCCCAGCTGGAAGA
ATAAAACTGCAAGGAATAGGGGGGATAGTAGAAGGGGAAAAATGGAATAATGTAATATTGGAATATAAAG
GAGAAACAAGAAAGGGAACAATAGTAGTGTTACCACAAAGTCCAGTAGAAGTATTAGGACGAGATAACAT
GGCCCGATTTGGCGTAGAGATACTCATGGCAAATTTAGAAGAAAAGAGAATCCCAATTACAAAAGTAAAA
TTGAAAGAGGGATGTACGGGTCCTCATGTCCCACAATGGCCATTAACAGAAGAAAAATTAAAAGGCCTAA
CAGAAATCATAGACAAATTAGTGGAAGAAGGAAAACTAGGGAAGGCACCCCCACATTGGACATGCAATAC
TCCAATATTTTGTATAAAAAAGAAATCAGGAAAGTGGAGAATGTTAATAGATTTCAGAGAATTGAACAAA
CAGACAGAAGATTTAACAGAAGCGCAGTTAGGACTACCACATCCGGGAGGACTACAAAGAAAAAACATG
TTACAATATTAGACATAGGAGATGCATATTTTACTATACCCCTATATGAACCCTATAGAGAGTACACATG

TTTTACTTTATTAAGTCCTAATAATCTAGGACCATGTAAAAGATACTATTGGAAAGTGCTGCCACAAGGT
TGGAAACTGAGTCCATCTGTATATCAGTTTACCATGCAGGAAATTTTAGAGGATTGGATACAACAGCATC
CTGAAATTCAATTTGGAATATACATGGATGATATTTACATAGGAAGTGATTTAGAAATAAAAAGGCACAG
AGAAATAGTGAAAGAATTAGTCAATTATATTGCCCAATATGGATTCACCCTACCAGAAGAAAAGAGACAA
GAGGGATATCCTGTCAAATGGCTAGGATTTGAACTACACCCTCAGACCTGGAAATTTCAGAAGCATACCT
TACCTGAATTAACAAAAGGGATCATTACATTAAATAAATTGCAAAAATTAGTAGGGGAGTTAGTATGGAG
ACAATCCATAATTGGAAAAAGCATTCCTAACATTCTGAAATTAATGGAAGGGGATAGAGAATTACAAAGT
GAAAGAAAAAATTGAAGAAGTACATGTGAAAGAATGGGAAGCATGTAGGAAAAAATTAGAAGAAATGGAAG
GAAATTATTATAATGCAGAAAAAGATGTCTATGGACAATTGGCTTGGGGAGACAAAGCTATAGAATATAT
AGTGTATCAGGAAAAAGGGAAACCGTTATGGGTAAATGTGGTTCACAATATAAAAAACCTAAGCATCCCG
CAACAGATTATTAAAGCAGCACAAAAACTAACACAAGAAGTAATTATTAGGACAGGGAAAATACCATGGG
TATTACTGCCAGGGAAAGAAGAAGATTGGAGACTAGAATTGCAATTAGGGAACATCACATGGATGCCAAA
ATTTTGGTCCTGTTATCGAGGACATACAAGATGGAGGAAAAGAAACATAATAGAAGAAGTAGTAGCAGGA
CCTACATATTATACAGATGGAGGAAAAAAGAATAAAATGGGAAGCTTAGGGTTCATAGCATCAACAGGGG
AAAAATTTAGAAAGCATGAAGAAGGCACAAATCAGCAATTAGAATTAAGAGCCATAGAAGAAGCTCTAAA
ACAAGGGCCTCCAACAATGAATTTAGTAACAGATAGTAGATATGCATTTGAATTTTTATTAAGAAACTGG
GATGAAGAAGTAATAAGAAATCCAATTCAAACAAGAATTATGGAAATTGCCCACAAGAAAGATAGAATAG
GAGTGCATTGGGTGCCAGGACACAAAGGAATTCCTCAAAATGAAGAAATAGATAGATATATCTCAGAAAT
ATTCTTGGCAAAAGAAGGAGAAGGAATTCTCCCAAAAAGAGAAGAAGATGCAGGATATGATTTAATATGT
CCAGAAGAAGTTACCATAGAACCAGGACAAGTGAAATGCATTCCCATAGATTTAAGAATAAATTTAAAGA
AATCACAATGGGCTATGATTGCTACAAAAAGCAGCATGGCTGCCAAAGGCGTATTCACACAAGGAGGAAT
CATAGACTCAGGATATCAGGGACAAATACAGGTAATAATGTATAATAGCAATAAAATAGCAGTAGTCATA
CCCCAAGGGAGAAAGTTTGCACAATTAATATTAATGGAGAAAATGCATGAAGAATTGGAACCCTGGGGGA
AAAGCAGAAAAACAGAGAGGGGAGAAAAAGGATTTGGGTCCACAGGAATGTATTGGATAGAAAATATTCC
TTTGGCAGAAGAAGACCACACAAAATGGCATCAAGATGCCAGATCATTGCATCTAGAATTTGAAATTCCA
AGAACAGCAGCAGAAGACATAGTAAACCAATGTGAAATATGTCAAAAAGAAAGGACACCTGCCGTGATCA
GAGGAGGAAACAAAAGAGGGGTAGATCACTGGCAAGTAGATTATACCCATTATGAAAATATCATACTATT
AGTATGGGTAGAAACAAATTCAGGATTAATATATGCAGAGAAAGTAAAAGGAGAATCAGGACAAGAATTC
AGAATAAAAGTGATGCAATGGTATGCATTATTTGGTCCAGAGTCATTGCAGTCAGACAATGGACCTGCAT
TTACAGCAGAGCCCACACAGCTGTTGATGCAATACTTAGGAATAAAACACACAACCGGGATACCTTGGAA
TCCACAGTCTCAGGCTATAGTAGAAAGGGCACATCAACTATTAAAAAGTGCTTTAAAGAAATTTCAGCCA
CAATTTGTCGCTGTAGAGTCAGCAATAGCAGCAGCCCTAGTCGCCATAAATATAAAAAGAAAGGGTGGGC
TAGGGACAAGCCCTATGGATATTTTTATATATAATAAAGAACAGAAAAGAATAAGTAATAAATATAATAA
AAATTCTCAAAAAATTCAATTCTGTTATTACAGAATAAGGAAAAGAGGACATCCAGGAGAGTGGAAAGGA
CCAACTCAGGTACTGTGGAAAGGGGAAGGAGCAATTGTGGTAAAGGATACAGAAAGGCAAAAGTATTTAG
TAATACCTTACAAAGATGCGAAATTCATCCCGCCACCAACAAAGGAGAAGGAATAG

>KT749881.1 Caprine arthritis encephalitis virus isolate gs-35V,
complete genome
ATGTCA
CAATTGTGGAAAAAGAGGACATATGCAAAAAGATTGCAGGGGAAAGAAAGAAATGGGAAGGCAGCAGGGA
AACGGGAGGAGGGGGATACGTGTGGTGCCGTCCGCTCCTCCTATGGAATAACTTCAGCACCACCAATGGT
TCGGGTCCGCATAGGTTCCCAGCAGAGGGACTTATTATTTGATACCGGGGCGGACCGAACTATAGTAAAA
TGGCATGATGGATCGGGAAACCCAGCTGGAAGAATAAAACTGCAAGGAATAGGGGGGATAGTAGAAGGGG
AAAAATGGAATAATGTAATATTGGAATATAAAGGAGAAACAAGAAAGGGAACAATAGTAGTGTTACCACA
AAGTCCAGTAGAAGTATTAGGACGAGATAACATGGCCCGATTTGGCGTAGAGATACTCATGGCAAATTTA
GAAGAAAAGAGAATCCCAATTACAAAAGTAAAATTGAAAGAGGGATGTACGGGTCCTCATGTCCCACAAT
GGCCATTAACAGAGGAAAAATTAAAAGGCCTAACAGAAATCATAGACAAATTAGTGGAAGAAGGAAAACT
AGGGAAGGCACCCCCACATTGGACATGCAATACTCCAATATTTTGTATAAAAAAGAAATCAGGAAAGTGG
AGAATGTTAATAGATTTCAGAGAATTGAACAAACAGACAGAAGATTTAACAGAAGCGCAGTTAGGACTAC
CACATCCGGGAGGACTACAAAAGAAAAAACATGTTACAATATTAGACATAGGAGATGCATATTTTACTAT
ACCCCTATATGAACCCTATAGAGAGTACACATGTTTTACTTTATTAAGTCCTAATAATCTAGGACCATGT
AAAAGATACTATTGGAAAGTGCTGCCACAAGGTTGGAAACTGAGTCCATCTGTATATCAGTTTACCATGC
AGGAAATTTTAGAGGATTGGATACAACAGCATCCTGAAATTCAATTTGGAATATACATGGATGATATTTA
CATAGGAAGTGATTTAGAAATAAAAAGGCACAGAGAAATAGTGAAAGAATTAGTCAATTATATTGCCCAA
TATGGATTCACCCTACCAGAAGAAAAGAGACAAGAGGGATATCCTGTCAAATGGCTAGGATTTGAACTAC
ACCCTCAGACCTGGAAATTTCAGAAGCATACCTTACCTGAATTAACAAAAGGGATCATTACATTAAATAA
ATTGCAAAAATTAGTAGGGGAGTTAGTATGGAGACAATCCATAATTGGAAAAAGCATTCCTAACATTCTG
AAATTAATGGAAGGGGATAGAGAATTACAAAGTGAAAGAAAAAATTGAAGAAGTACATGTGAAAGAATGGG
AAGCATGTAGGAAAAAATTAGAAGAAATGGAAGGAAATTATTATAATGCAGAAAAAGATGTCTATGGACA
ATTGGCTTGGGGAGACAAAGCTATAGAATATATAGTGTATCAGGAAAAAGGGAAACCGTTATGGGTAAAT

GTGGTTCACAATATAAAAAACCTGAGCATCCCGCAACAGATTATTAAAGCAGCACAAAAACTAACACAAG
AAGTAATTATTAGGACAGGGAAAATACCATGGGTATTACTGCCAGGGAAAGAAGAAGATTGGAGACTAGA
ATTGCAATTAGGGAACATCACATGGATGCCAAAATTTTGGTCCTGTTATCGAGGACATACAAGATGGAGG
AAAAGAAACATAATAGAAGAAGTAGTAGCAGGACCTACATATTATACAGATGGAGGAAAAAAGAATAAAA
TGGGAAGCTTAGGGTTCATAGCATCAACAGGGGAAAAATTTAGAAAGCATGAAGAAGGCACAAATCAGCA
ATTAGAATTAAGAGCCATAGAAGAAGCTCTAAAACAAGGGCCTCCTACAATGAATTTAGTAACAGATAGT
AGATATGCATTTGAATTTTTATTAAGAAACTGGGATGAAGAAGTAATAAGAAATCCAATTCAAACAAGAA
TTATGGAACTTGCCCACAAGAAAGAAAAGATAGGAGTGCATTGGGTGCCAGGACACAAAGGAATTCCTCA
AAATGAAGAAATAGATAGATATATCTCAGAAATATTCTTGGCAAAAGAAGGAGAAGGAATTCTCCCAAAA
AGAGAAGAAGATGCAGGATATGATTTAATATGTCCAGAAGAAGTTACCATAGAACCAGGACAAGTGAAAT
GCATTCCCATAGATTTAAGAATAAATTTAAAGAAATCACAATGGGCTATGATTGCTACAAAAAGCAGCAT
GGCTGCCAAAGGCGTATTCACACAAGGAGGAATCATAGACTCAGGATATCAGGGACAAATACAGGTAATA
ATGTATAATAGCAATAAAATAGCAGTAGTCATACCCCAAGGGAGAAAGTTTGCACAATTAATATTAATGG
AGAAAATGCATGAAGAATTGGAACCCTGGGGGAAAAGCAGAAAAACAGAGAGGGGAGAAAAAGGATTTGG
GTCCACAGGAATGTATTGGATAGAAAATATTCCTTTGGCAGAAGAAGACCACACAAAATGGCATCAAGAT
GCCAGATCATTGCATCTAGAATTTGAAATTCCAAGAACAGCAGCAGAAGACATAGTAAACCAATGTGAAA
TATGTCAAAAAGAAAGGACACCTGCCGTGATCAGAGGAGGAAACAAAAGAGGGGTAGATCACTGGCAAGT
AGATTATACCCATTATGAAAATATCATACTATTAGTATGGGTAGAAACAAATTCAGGATTAATATATGCA
GAGAAAGTAAAAGGAGAATCAGGACAAGAATTCAGAATAAAAGTGATGCAATGGTATGCATTATTTGGTC
CAGAGTCATTGCAGTCAGACAATGGACCTGCATTTACAGCAGAGCCCACACAGCTGTTGATGCAATACTT
AGGAATAAAACACACAACCGGGATACCTTGGAATCCACAGTCTCAGGCTATAGTAGAAAGGGCACATCAA
CTATTAAAAAGTGCTTTAAAGAAATTTCAGCCACAATTTGTCGCTGTAGAGTCAGCAATAGCAGCAGCCC
TAGTCGCCATAAATATAAAAAGAAAGGGTGGGCTAGGGACAAGCCCTATGGATATTTTTATATATAATAA
AGAACAGAAAAGAATAAGTAATAAATATAATAAAAATTCTCAAAAAATTCAATTCTGTTATTACAGAATA
AGGAAAAGAGGACATCCAGGAGAGTGGAAAGGACCAACTCAGGTACTGTGGAAAGGGGAAGGAGCAATTG
TGGTAAAGGATACAGAAAGGCAAAAGTATTTAGTAATACCTTACAAAGATGCGAAATTCATCCCGCCACC
AGCAAAGGAGAAGGAATAG

>MG554410.1 Small ruminant lentivirus isolate SRLV010, complete genome
CGGGAA
GCGGGGGATACGTGTGGTGCCGTCCGCTCCCCCTATGGAATAACTTCAGCACCACCGATGGTTCAGGTCC
GCATAGGTTCCCAGCAGAGGAACTTATTATTTGATACCGGGGCGGACCGAACTATAATAAAATGGCATGA
TGGCTCAGGAATCCCAGCCGGAAGAATAAAATTACAAGGAATAGGAGGGGTGGTGGAAGGAGAAAAGTGG
AAGGATGTACAATTGGAGTATAAAGGAGAAATAAGAAAGGGAACAATAGTAGTGCTGCCACAAAGCCCAG
TRGAAGTATTAGGGAGAGAYAACATGGCCAAATTTGGAATAGAAATAATTATGGCAAATTTAGAAGAAAA
GAAAATTCCTATTACAAAAGTAAAGTTGAAAGAGGGATGTAMAGGTCCTCATGTCCCGCAATGGCCCTTG
ACAGAAGAAAAGTTACAAGGTCTAACAGAAATAATAGATAAGTTAGTGGAGGAAGGAAAATTAGGAAAGG
CACCCCCACATTGGACATGTAATACTCCAATATTTTGTATAAAAAAGAAGTCAGGAAAATGGAGAATGTT
AATAGATTTCAGAGAATTGAATAAACAGACAGAAGATTTAACAGAAGCACAATTAGGACTCCCGCATCCT
GGGGGATTACAAAGGAAGAAACATGTTACAATATTAGACATAGGAGATGCGTATTTTACTATACCCTTGT
ACGAACCATATCGAGAATATACATGCTTTACTCTATTAAGTCCTAATAATCTAGGACCATGTAAAAGATA
TTATTGGAAAGTCCTACCACAAGGGTGGAAATTGAGTCCTTCAGTATATCAATTTACTATGCAACAGATA
TTAGAGGATTGGATACAACAGCATCCTGAAATTCAATTTGGAATATATATGGATGATATTTACATAGGAA
GTGATTTGGAAATAAAGAAGCATAGAGAAAGAGTAGAGGAATTAGCAAATTATATTGCCCAGTATGGATT
TACCCTGCCAGAAGAAAAGAGACAAGAGGGATATCCAGCAAAGTGGCTAGGATTTGAATTACACCCTCAG
ACCTGGAAATTCCAGAAACATACTTTACCTGAATTAAGAGAAGGGATAATTACATTAAACAAATTGCAAA
AATTAGTAGGAGAATTAGTATGGAGGCAATCCATAATTGGAAAAAGTATTCCCAACATCTTAAAATTGAT
GGAAGGAGATAGAGCATTACAAAGTGAAAGAAGAATTGAAGCMATACATGTGAAAGAATGGGAGGCATGT
AGAAAAAGATTAGAAGAAATGGAAGGAAATTATTATAATGCAGAAAAGGATCTCTATGGACAGCTAGCWT
GGGGTGACAAAGCAATAGAGTATATAGTGTATCAAGAAAAAGGGAAACCATTATGGGTAAATGTGGTGCA
CAATGTAAAGAACTTAAGCATCCCACAGCAAATTATTAAAGCCGCACAAAAGCTAACACAAGAAGTAATA
ATTAGAACAGGAAAAATACCATGGATTCTATTACCAGGGAAAGAAGAAGATTGGAGATTAGAATTGCAAT
TAGGAAACATCACGTGGATGCCAAAATTTTGGTCATGTTATAGAGGACAAACTAGATGGCAAAAGAGAAA
TATAATAGAAAGTGTAGTAGAAGGACCTACCTACTATACAGATGGGGGAAAAAAGAATAAAATAGGAAGC
TTAGGGTTCATAACCTCAACAGGGGAGAAATTTAGAAAGCATGAAGAAGGCACAAATCAGCAACTGGAAC
TAAGAGCCATTGAAGAAGCTCTAAAGCAAGGCCCTCAGACTATGAATATAGTAACAGATAGTAGATATGC
ATTTGAATTTATGTTAAGAAATTGGGATGAAGAAGTAATTAAAAATCCAATTCAAGCAAGAATAATGAGG
ATAGCCCATAAAAAGAACATGATAGGAGTGCATTGGGTGCCCGGACATAAAGGAATCCCACAAAATGAAG
AAATAGACACATATATCTCAGAAATATTCCTTGCAAAAACAGGAGAAGGAATTCTCCCAAAAAGAGAAGA
AGATGCAGGATATGATTTAATATGTCCAAAAGAGATAACTATAGGGCCAGGACAAGTGGAAAAAATCCCC
ATAGACTTAAGAATAAATTTAAAGGAGTCACAATGGGCTATGATTGCTTCAAAAAGCAGCATGGCCGCTA

AGGGAGTATTCACACAAGGGGGTATCATAGATTCAGGCTATCAGGGACAAATACAAGTAATAGTGTACAA
TAGCAATAATGTAGCAGTGGTCATACCTCAAGGAAGAAAGTTTGCACAGTTAATATTAATGGATAAAAAG
CATGAAGAATTAGAACCCTGGGGGATTAGCAGAAAAACAGAAAGAGGAGAAAAAGGATTTGGGTCCACAG
GGCTGTATTGGATAGAAAACATTCCTCTAGCAGAAGAGGATCACTCAAAGTGGCATCAAGATGCTCGCTC
ACTGCACCTGGAATTTGAAATTCCCAGAACTGCAGCAGAAGATATAGTAAATCAATGTGAAGTATGCAAA
GAAGAAAAAGCACCTTCCCTTGTTAGAGGAGGAAACAAAAGAGGAGTAGATCACTGGCAAGTAGATTATA
CCCACTATGAAAATAATATACTATTAGTATGGGTGGAAACAAATTCAGGGCTAATATTTGCAGAAAAAGT
AAAAGGAGAATCAGGACAAGAATTCAGAACAAAGGTGATGCAATGGTATGCATTATTTGGTCCAGAATCA
TTGCAGTCAGATAATGGACCTGCATTTACAGCTGAAGCCACACAATTGTTAATGACATATTTAGGAATAA
AACACACAACAGGGATACCATGGAATCCACAATCTCAAGCAATAGTAGAAAGAGCACATCAACACTTGAA
AAGTACTTTAAAGAAGTTTCAGCCACAATTTGTAGCTATAGAATCAGCAATAGCAGCAGCCCTAGTCGCC
ACAAACATAAAAAGAAAGGGTGGGCTAGGGACAAGCCCTATGGATATTTTTATATATAATAAAGAACAGA
AAAGAGTAAATAATAAATATAATAAAAATTTTGAAAGAATTCAATTCTGTTATTACAGAGTAAGAAAAAG
AGGACATCCAGGAGAGTGGAAGGGACCAACTCAGGTACTGTGGAAAGGGGAAGGAGCCATTGTGGTAAAA
GATACAGAAAGCGACAAGTACTTAGTAATACCTTACAAAGATGCAAAATTCATCCCGCCGCCAACAAAAG
AAAAGGAATAA

>KT749880.1 Caprine arthritis encephalitis virus isolate guizhou,
complete genome
ATGTCA
CAATTGTGGAAAAAGAGGACATATGCAAAAAGATTGCAGGGGAAAGAAAGAAATGGGAAGGCAGCAGGGA
AACGGGAGGAGGGGGATACGTGTGGTGCCGTCCGCTCCTCCTATGGAATAACTTCAGCACCACCAATGGT
TCGGGTCCGCATAGGTTCCCAGCAGAGGGACTTATTATTTGATACCGGGGCGGACCGAACTATAGTAAAA
TGGCATGATGGATCGGGAAACCCAGCTGGAAGAATAAAACTGCAAGGAATAGGGGGGATAGTAGAAGGGG
AAAAATGGAATAATGTAATATTGGAATATAAAGGAGAAACAAGAAAGGGAACAATAGTAGTGTTACCACA
AAGTCCAGTAGAAGTATTAGGACGAGATAACATGGCCCGATTTGGCGTAGAGATACTCATGGCAAATTTA
GAAGAAAAGAGAATCCCAATTACAAAAGTAAAATTGAAAGAGGGATGTACGGGTCCTCATGTCCCACAAT
GGCCATTAACAGAGGAAAAATTAAAAGGCCTAACAGAAATCATAGACAAATTAGTGGAAGAAGGAAAACT
AGGGAAGGCACCCCCACATTGGACATGCAATACTCCAATATTTTGTATAAAAAAGAAATCAGGAAAGTGG
AGAATGTTAATAGATTTCAGAGAATTGAACAAACAGACAGAAGATTTAACAGAAGCGCAGTTAGGACTAC
CACATCCGGGAGGACTACAAAAGAAAAAACATGTTACAATATTAGACATAGGAGATGCATATTTTACTAT
ACCCCTATATGAACCCTATAGAGAGTACACATGTTTTACTTTATTAAGTCCTAATAATCTAGGACCATGT
AAAAGATACTATTGGAAAGTGCTGCCACAAGGTTGGAAACTGAGTCCATCTGTATATCAGTTTACCATGC
AGGAAATTTTAGAGGATTGGATACAACAGCATCCTGAAATTCAATTTGGAATATACATGGATGATATTTA
CATAGGAAGTGATTTAGAAATAAAAAGGCACAGAGAAATAGTGAAAGAATTAGTCAATTATATTGCCCAA
TATGGATTCACCCTACCAGAAGAAAAGAGACAAGAGGGATATCCTGTCAAATGGCTAGGATTTGAACTAC
ACCCTCAGACCTGGAAATTTCAGAAGCATACCTTACCTGAATTAACAAAAGGGATCATTACATTAAATAA
ATTGCAAAAATTAGTAGGGGAGTTAGTATGGAGACAATCCATAATTGGAAAAAGCATTCCTAACATTCTG
AAATTAATGGAAGGGGATAAAGAATTACAAAGTGAAAGAAAAATTGAAGAAGTACATGTGAAAGAATGGG
AAGCATGTAGGAAAAAATTAGAAGAAATGGAAGGAAATTATTATAATGCAGAAAAAGATGTCTATGGACA
ATTGGCTTGGGGAGACAAAGCTATAGAATATATAGTGTATCAGGAAAAAGGGAAACCGTTATGGGTAAAT
GTGGTTCACAATATAAAAAACCTGAGCATCCCGCAACAGATTATTAAAGCAGCACAAAAACTAACACAAG
AAGTAATTATTAGGACAGGGAAAATACCATGGGTATTACTGCCAGGGAAAGAAGAAGATTGGAGACTAGA
ATTGCAATTAGGGAACATCACATGGATGCCAAAATTTTGGTCCTGTTATCGAGGACATACAAGATGGAGG
AAAAGAAACATAATAGAAGAAGTAGTAGCAGGACCTACATATTATACAGATGGAGGAAAAAAGAATAAAA
TGGGAAGCTTAGGGTTCATAGCATCAACAGGGGAAAAATTTAGAAAGCATGAAGAAGGCACAAATCAGCA
ATTAGAATTAAGAGCCATAGAAGAAGCTCTAAAACAAGGGCCTCCAACAATGAATTTAGTAACAGATAGT
AGATATGCATTTGAATTTTTATTAAGAAACTGGGATGAAGAAGTAATAAGAAATCCAATTCAAACAAGAA
TTATGGAACTTGCCCACAAGAAAGAAAAGATAGGAGTGCATTGGGTGCCAGGACACAAAGGAATTCCTCA
AAATGAAGAAATAGATAGATATATCTCAGAAATATTCTTGGCAAAAGAAGGAGAAGGAATTCTCCCAAAA
AGAGAAGAAGATGCAGGATATGATTTAATATGTCCAGAAGAAGTTACCATAGAACCAGGACAAGTGAAAT
GCATTCCCATAGATTTAAGAATAAATTTAAAGAAATCACAATGGGCTATGATTGCTACAAGAAGCAGCAT
GGCTGCCAAAGGCGTGTTCACACAAGGAGGAATCATAGACTCAGGATATCAGGGACAAATACAGGTAATA
ATGTATAATAGCAATAAAATAGCAGTAGTCATACCCCAAGGGAGAAAGTTTGCACAATTAATATTAATGG
AGAAAATGCATGAAGAATTGGAACCCTGGGGGAAAAGCAGAAAAACAGAGAGGGGAGAAAAAGGATTTGG
GTCCACAGGAATGTATTGGATAGAAAATATTCCTTTGGCAGAAGAAGACCACACAAAATGGCATCAAGAT
GCCAGATCATTGCATCTAGAATTTGAAATTCCAAGAACAGCAGCAGAAGACATAGTAAACCAATGTGAAA
TATGTCAAGAAGAAAGGACACCTGCCGTGATCAGAGGAGGAAACAAAAGAGGGGTAGATCACTGGCAAGT
AGATTATACCCATTATGAAAATATCATACTATTAGTATGGGTAGAAACAAATTCAGGATTAATATATGCA
GAGAAAGTAAAAGGAGAATCAGGACAAGAATTCAGAATAAAAGTGATGCAATGGTATGCATTATTTGGTC
CAGAGTCATTGCAGTCAGACAATGGACCTGCATTTACAGCAGAGCCCACACAGCTGTTGATGCAATACTT

AGGAATAAAACACACAACCGGGATACCTTGGAATCCACAGTCTCAGGCTATAGTAGAAAGGGCACATCAA
CTATTAAAAAGTGCTTTAAAGAAATTTCAGCCACAATTTGTCGCTGTAGAGTCAGCAATAGCAGCAGCCC
TAGTCGCCATAAATATAAAAAGAAAGGGTGGGCTAGGGACAAGCCCTATGGATATTTTAATATATAATAA
AGAACAGAAAAGAATAAGTAATAAATATAATAAAAATTCTCAAAAAATTCAATTCTGTTATTACAGAATA
AGGAAAAGAGGACATCCAGGAGAGTGGAAAGGACCAACTCAGGTACTGTGGAAAGGGGAAGGAGCAATTG
TGGTAAAGGATACAGAAAGGCAAAAGTATTTAGTAATACCTTACAAAGATGCGAAATTCATCCCGCCACC
AACAAAGGAGAAGGAATAG

>KT749879.1 Caprine arthritis encephalitis virus isolate Shaanxi, complete genome
ATGTCA
CAATTGTGGAAAAAGAGGACATATGCAAAAAGATTGCAGGGGAAAGAAAGAAATGGGAAGGCAGCAGGGA
AACGGGAGGAGGGGGATACGTGTGGTGCCGTCCGCTCCTCCTATGGAATAACTTCAGCACCACCAATGGT
TCGGGTCCGCATAGGTTCCCAGCAGAGGGACTTATTATTTGATACCGGGGCGGACCGAACTATAGTAAAA
TGGCATGATGGATCGGGAACCCCAGCTGGAAGAATAAAACTGCAAGGAATAGGGGGGATAGTAGAAGGGG
AAAAATGGAATAATGTAATATTGGAATATAAAGGAGAAACAAGAAAGGGAACAATAGTAGTGTTACCACA
AAGTCCAGTAGAAGTATTAGGACGAGATAACATGGCCCGATTTGGCGTAGAGATACTCATGGCAAATTTA
GAAGAAAAGAGAATCCCAATTACAAAAGTAAAATTGAAAGAGGGATGTACGGGTCCTCATGTCCCACAAT
GGCCATTAACAGAGGAAAAATTAAAAGGCCTAACAGAAATCATAGACAAATTAGTGGAAGAAGGAAAACT
AGGGAAGGCACCCCCACATTGGACATGCAATACTCCAATATTTTGTATAAAAAAGAAATCAGGAAAGTGG
AGAATATTAATAGATTTCAGAGAATTGAACAAACAGACAGAAGATTTAACAGAAGCGCAGTTAGGACTAC
CACATCCGGGAGGACTACAAAAGAAAAAACATGTTACAATATTAGACATAGGAGATGCATATTTTACTAT
ACCCCTATATGAACCCTATAGAGAGTACACATGTTTTACTTTATTAAGTCCTAATAATCTAGGACCATGT
AAAAGATACTATTGGAAAGTGCTGCCACAAGGTTGGAAACTGAGTCCATCTGTATATCAGTTTACCATGC
AGGAAATTTTAGAGGATTGGATACAACAGCATCCTGAAATTCAATTTGGAATATACATGGATGATATTTA
CATAGGAAGTGATTTAGAAATAAAAAGGCACAGAGAAATAGTGAAAGAATTAGTCAATTATATTGCCCAA
TATGGATTCACCCTACCAGAAGAAAAGAGACAAGAGGGATATCCTGTCAAATGGCTAGGATTTGAACTAC
ACCCTCAGACCTGGAAATTTCAGAAGCATACCTTACCTGAATTAACAAAAGGGATCATTACACTAAATAA
ATTGCAAAAATTAGTAGGGGAGTTAGTATGGAGACAATCCATAATTGGAAAAAGCATTCCTAACATTCTG
AAATTAATGGAAGGGGATAGAGAATTACAAAGTGAAAGAAAAATTGAAGAAGTACATGTGAAAGAATGGG
AAGCATGTAGGAAAAAATTAGAAGAAATGGAAGGAAATTATTATAATGCAGAAAAAGATGTCTATGGACA
ATTGGCTTGGGGAGACAAAGCTATAGAATATATAGTGTATCAGGAAAAAGGGAAACCGTTATGGGTAAAT
GTGGTTCACAATATAAAAAACCTGAGCATCCCGCAACAGATTATTAAAGCAGCACAAAAACTAACACAAG
AAGTAATTATTAGGACAGGGAAAATACCATGGGTATTACTGCCAGGGAAAGAAGAAGATTGGAGACTAGA
ATTGCAATTAGGGAACATCACATGGATGCCAAAATTTTGGTCCTGTTATCGAGGACATACAAGATGGAGG
AAAAGAAACATAATAGAAGAAGTAGTAGCAGGACCTACATATTATACAGATGGAGGAAAAAAGAATAAAA
TGGGAAGCTTAGGGTTCATAGCATCAACAGGGGAAAAATTTAGAAAGCATGAAGAAGGCACAAATCAGCA
ATTAGAATTAAGAGCCATAGAAGAAGCTCTAAAACAAGGGCCTCCAACAATGAATTTAGTAACAGATAGT
AGATATGCATTTGAATTTTTATTAAGAAACTGGGATGAAGAAGTAATAAGAAATCCAATTCAAACAAGAA
TTATGGAACTTGCCCACAAGAAAGAAAAGATAGGAGTGCATTGGGTGCCAGGACACAAAGGAATTCCTCA
AAATGAAGAAATAGATAGATATATCTCAGAAATATTCTTGGCAAAAGAAGGAGAAGGAATTCTCCCAAAA
AGAGAAGAAGATGCAGGATATGATTTAATATGTCCAGAAGAAGTTACCATAGAACCAGGACAAGTGAAAT
GCATTCCCATAGATTTAAGAATAAATTTAAAGAAATCACAATGGGCTATGATTGCTACAAAAAGCAGCAT
GGCTGCCAAAGGCGTATTCACACAAGGAGGAATCATAGACTCAGGATATCAGGGACAAATACAGGTAATA
ATGTATAATAGCAATAAAATAGCAGTAGTCATACCCCAAGGGAGAAAGTTTGCACAATTAATATTAATGG
AGAAAATGCATGAAGAATTGGAACCCTGGGGGAAAAGCAGAAAAACAGAGAGGGGAGAAAAAGGATTTGG
GTCCACAGGAATGTATTGGATAGAAAATATTCCTTTGGCAGAAGAAGACCACACAAAATGGCATCAAGAT
GCCAGATCATTGCATCTAGAATTTGAAATTCCAAGAACAGCAGCAGAAGACATAGTAAACCAATGTGAAA
TATGTCAAAAAGAAAGGACACCTGCCGTGATCAGAGGAGGAAACAAAAGAGGGGTAGATCACTGGCAAGT
AGATTATACCCATTATGAAAATATCATACTATTAGTATGGGTAGAAACAAATTCAGGATTAATATATGCA
GAGAAAGTAAAAGGAGAATCAGGACAAGAATTCAGAATAAAAGTGATGCAATGGTATGCATTATTTGGTC
CAGAGTCATTGCAGTCAGACAATGGACCTGCATTTACAGCAGAGCCCACACAGCTGTTGATGCAATACTT
AGGAATAAAACACACAACCGGGATACCTTGGAATCCACAGTCTCAGGCTATAGTAGAAAGGGCACATCAA
CTATTAAAAAGTGCTTTAAAGGAATTTCAGCCACAATTTGTCGCTGTAGAGTCAGCAATAGCAGCAGCCC
TAGTCGCCATAAATATAAAAAGAAAGGGTGGGCTAGGGACAAGCCCTATGGATATTTTTATATATAATAA
AGAACAGAAAAGAATAAGTAATAAATATAATAAAAATTCTCAAAAAATTCAATTCTGTTATTACAGAATA
AGGAAAAGAGGACATCCAGGAGAGTGGAAAGGACCAACTCAGGTACTGTGGAAAGGGGAAGGAGCAATTG
TGGTAAAGGATACAGAAAGGCAAAAGTATTTAGTAATACCTTACAAAGATGCGAAATTCATCCCGCCACC
AGCAAAGGAGAAGGAATAG

>GU120138.1 Caprine arthritis encephalitis virus strain Shanxi, complete genome
ATGTCACAAT
TGTGGAAAAAGAGGACATATGCAAAAAGATTGCAGGGGARAGAAAGAAATGGGAAGGCAGCAGGGAAACG
GGAGGAGGGGGATACGTGTGGTGCCGTCCGCTCCTCCTATGGAATAACTTCAGCACCACCAATGGTTCGG
GTCCGCATAGGTTCCCAGCAGAGGGACTTATTATTTGATACCGGGGCGGACCGAACTATAGTAAAATGGC
ATGATGGATCGGGAAACCCAGCTGGAAGAATAAAACTGCAAGGAATAGGGGGGATAGTAGAAGGGGAAAA
ATGGAATAATGTAATATTGGAATATAAAGGAGAAACAAGAAAGGGAACAATAGTAGTGTTACCACAAAGT
CCAGTAGAAGTATTAGGACGAGATAACATGGCCCGATTTGGCGTAGAGATACTCATGGCAAATTTAGAAG
AAAAGAGAATCCCAATTACAAAAGTAAAATTGAAAGAGGGATGTACGGGTCCTCATGTCCCACAATGGCC
ATTAACAGAGGAAAAATTAAAAGGCCTAACAGAAATCATAGACAAATTAGTGGAAGAAGGAAAACTAGGG
AAGGCACCCCCACATTGGACATGCAATACTCCAATATTTTGTATAAAAAAGAAATCAGGAAAGTGGAGAA
TGTTAATAGATTTCAGAGAATTGAACAAACAGACAGAAGATTTAACAGAAGCGCAGTTAGGACTACCACA
TCCGGGAGGACTACAAAAGAAAAAACATGTTACAATATTAGACATAGGAGATGCATATTTTACTATACCC
CTATATGAACCCTATAGAGAGTACACATGTTTTACTTTATTAAGTCCTAATAATCTAGGACCATGTAAAA
GATACTATTGGAAGGTGCTGCCACAAGGTTGGAAACTGAGTCCATCTGTATATCAGTTTACCATGCAGGA
AATTTTAGAGGATTGGATACAACAGCATCCTGAAATTCAATTTGGAATATACATGGATGATATTTACATA
GGAAGTGATTTAGAAATAAAAAGGCACAGAGAAATAGTGAAAGAATTAGTCAATTATATTGCCCAATATG
GATTCACCCTACCAGAAGAAAAGAGACAAGAGGGATATCCTGTCAAATGGCTAGGATTTGAACTACACCC
TCAGACCTGGAAATTTCAGAAGCATACCTTACCTGAATTAACAAAAGGGATCATTACATTAAATAAATTG
CAAAAATTAGTAGGGGAGTTAGTATGGAGACAATCCATAATTGGAAAAAGCATTCCTAACATTCTGAAAT
TAATGGAAGGGGATAGAGAATTACAAAGTGAAAGAAAAATTGAAGAAGTACATGTGAAAGAATGGGAAGC
ATGTAGGAAAAAATTAGAAGAAATGGAAGGAAATTATTATAATGCAGAAAAAGATGTCTATGGACAATTG
GCTTGGGGAGACAAAGCTATAGAATATATAGTGTATCAGGAAAAAGGGAAACCGTTATGGGTAAATGTGG
TTCACAATATAAAAAACCTGAGCATCCCGCAACAGATTATTAAGGCAGCGCAAAAGTTAACACAAGAAGT
CATCATTAGGACAGGAAAAATACCATGGATATTACTGCCAGGGAAAGAAGAAGATTGGAGACTAGAATTA
CAATTAGGGAACATCACATGGATGCCAAAATTTTGGTCCGGTTATCGAGGACATACAAGATGGAGAAAGA
GAAACATAATAGAAGAAGTAGTAGCAGGACCTACATATTATACAGATGGAGGAAAAAAGAATAAAATGGG
AAGCTTAGGGTTCATAGCATCAACAGGGGAAAAATTTAGAAAGCATGAAGAAGGCACAAATCAGCAATTA
GAATTAAGAGCCATAGAAGAAGCTCTAAAACAAGGGCCTCCAACAATGAATTTAGTAACAGATAGTAGAT
ATGCATTTGAATTTTTATTAAGAAACTGGGATGAAGAAGTAATAAGAAATCCAATTCAAACAAGAATTAT
GGAACTTGCCCACAAGAAAGAAAAGATAGGAGTGCATTGGGTGCCAGGACACAAAGGAATTCCTCAAAAT
GAAGAAATAGATAGATATATCTCAGAAATATTCTTGGCAAAAGAAGGAGAAGGAATTCTCCCAAAAAGAG
AAGAAGATGCAGGATATGATTTAATATGTCCAGAAGAAGTCACCATAGAACCAGGACAAGTGAAATGCAC
TCCCATAGATTTAAGAATAAATTTAAAGAAATCACAATGGGCTATGATTGCTACAAAAAGCAGCATGGCT
GCCAAAGGCGTATTCACACAAGGAGGAATCATAGACTCAGGATATCAGGGACAAATACAGGTAATAATGT
ATAATAGCAATAAAATAGCAGTAGTCATACCCCAAGGGAGAAAGTTTGCACAATTAATATTAATGGAGAA
AATGCATGAAGAATTGGAACCCTGGGGGAGAAGCAGAAAAACAGAGAGGGGAGAAAAAGGATTTGGGTCC
ACAGGAATGTATTGGATAGAAAATATTCCTTTGGCAGAAGAAGACCACACAAAATGGCATCAAGATGCCA
GATCATTGCATCTAGAATTTGAAATTCCAAGAACAGCAGCAGAAGACATAGTAAACCAATGTGAAATATG
TCAAGAAGAAAGGACACCTGCCGTGATCAGAGGAGGAAACAAAAGAGGGGTAGATCACTGGCAAGTAGAT
TATACCCATTATGAAAATATCATACTATTAGTATGGGTAGAAACAAATTCAGGATTAATATATGCAGAGA
AAGTAAAAGGAGAATCAGGACAAGAATTCAGAATAAAAGTGATGCAATGGTATGCATTATTTGGTCCAGA
GTCATTGCAGTCAGACAATGGACCTGCATTTACAGCAGAGCCCACACAGCTGTTGATGCAATACTTAGGA
ATAAAACACACAACCGGGATACCTTGGAATCCACAGTCTCAGGCTATAGTAGAAAGGGCACATCAACTAT
TAAAAAGTGCTTTAAAGAAATTTCAGCCACAATTTGTCGCTGTAGAGTCAGCAATAGCAGCAGCCCTAGT
CGCTATAAATATAAAAAGAAAGGGTGGGCTAGGGACAAGCCCTATGGATATTTTTATATATAATAAAGAA
CAGAAAAGAATAAGTAATAAATATAATAAAAATTCTCAAAAAATTCAATTCTGTTATTACAGAATAAGGA
AAAGAGGACATCCAGGAGAGTGGAAAGGACCAACTCAGGTACTGTGGAAAGGGGAAGGAGCAATTGTGGT
AAAGGATACAGAAAGGCAAAAGTATTTAGTAATACCTTGCAAAGATGCGAAATTCATCCCGCCACCAGCA
AAGGAGAAGGAATAG

>MG554413.1 Small ruminant lentivirus isolate SRLV017, complete genome
CGAGAAGAGGAGGCCACGTGTGGTGCCGTCCGCTCCTCCTATGGAA
TAAATGCTGCACCACTCATAGTTCAGGTCCGCATAGGTTCCCAAAAGAGGAACTTATTATTTGACACCGG
GGCGGACCGGACTATAGTAAAATGGCATGATGGCTCGGGGAACCCAGCCGGAAGAATAAAACTACAAGGA
ATAGGGGGCCTAGTAGAAGGAGAAAAATGGACTAATGTAGAATTGGAATATAAAGGAGAAATAAAAAAGG
GAACAATAGTAGTATTACCACAAAGTCCAGTAGAAGTACTAGGCAGAGATAACATGGGGAAGTTTGGTAT
AACCATAATAATGGCAAATTTAGAAGAGAAAAGAATCCCAATTACAAAAGTAAAATTGAAAGAGGGGTGT
ACAGGCCCTCATGTCCCGCAATGGCCCTTAACAGAAGAAAAGTTAAAGGGTCTAACAGAAATAGTAGACA
AACTAGTAGAAGAAGGAAAATTAGGAAAGGCACCCCCACATTGGACATGTAATACTCCCATATTTGCAAT

AAAAAAGAAATCAGGAAAATGGAGAATGCTAATAGATTTTAGAGAATTAAACAAACAAACAGAAGATTTA
ACAGAGGCACAATTGGGGCTCCCACATCCTGGAGGATTGCGGAAGAAGAAAAATGTTACAATATTAGACA
TAGGAGATGCATATTTCACTATACCCTTGTATGAACCATATCGAGAATATACATGTTTTACTCTATTAAG
TCCTAACAATTTAGGACCATGTAGAAGATATTATTGGAAAGTACTGCCACAAGGATGGAAGCTAAGTCCT
TCTGTATATCAATTTACTATGCAGAAAATATTAGAGGAGTGGATACAAAGGCATCCTGAAATTCAATTTG
GAATATATATGGATGATATTTATATAGGAAGTGATTTAGAAATTAAAAAACATAGAGAAATAGTGGAAGA
ACTAGCGAATTATATTGCCCAATATGGATTTACCCTGCCAGAAGAAAAAAGACAAGAGGGATATCCAGCA
AAGTGGCTAGGATTTGAATTACACCCTCAGACCTGGAAATTCCAGAAACACACTTTACCTGAATTAAGAG
AAGGAATAATTACATTAAATAAATTGCAAAAATTAGTAGGAGAATTAGTATGGAGGCAGTCCATAATTGG
AAAGAGCATTCCCAATATTTTAAAATTAATGGAAGGGGATAGAGCATTACAAAGTGAAAGAAGAATTGAA
GCCATACATGTGAAAGAATGGGAGGCATGTAGAAAAAAGTTAGAAGAAATGGAAGGAAATTACTATGATG
AAGAAAAAGATATCTATGGACAGCTAGCTTGGGGTAACAAAGCTATAGAATATATAGTATATCAGGAAAA
AGGGAAACCCTTATGGGTAAATGTGGTTCACAATGTGAAGAACCTAAGCATCCCACAGCAAATTATTAAG
GCCGCACAAAAGCTAACACAGGAAGTAATTATTAGAACAGGAAAAATACCATGGATATTATTACCAGGGA
AAGAAGAAGATTGGAGATTAGAATTGCAATTAGGAAATATAACGTGGATGCCAAAATTTTGGTCATGTTA
TAGGGGACAAACTAGATGGAAAACAAGAAATGTAATGGAAGAGGTAGTAACAGGACCTACCTATTACACT
GATGGCGGGAAAAAGAATAAAACAGGAAGCTTAGGGTTCATAGCCTCAACAGGAGAAAAATTTAGAAAAC
ATGAAGAAGGCACAAATCAGCAACTTGAGCTAAGAGCCATTGAAGAAGCTCTAAAGCAAGGGCCAAGCAC
AATGAATATAGTAACAGATAGCAGATATGCATTTGAATTTTTATTAAGAAATTGGGATGAAGAAGTAATA
AAAAATCCAATTCAAGCAAGGATTATGGCACTAGCCCATAAAAAAGATAAGATAGGGGTACATTGGGTAC
CAGGACATAAAGGAATCCCCCAAAATGAAGAAATAGATACCTATATCTCGGAGATATTCCTTGCAAAAGA
AGGAAATGGAATTCTCCCAAAAAGAGAGGAAGATGCTGGGTATGACTTAATATGTCCAAAAGAAGTCAGT
ATAGGGCCAGGACAGGTAGAAAAAATCCCCATAGATCTAAGACTAAACTTAAAGGAAACACAATGGGCTA
TGATTGCCACAAAAAGCAGCATGGCTGCCAAAGGAGTGTTTACACAAGGGGGAATCATAGATTCAGGATA
TCAGGGACAAATACAAGTAATAGTATACAACAGCAATAATGTAGCAGTGGTCATACCCCAAGGAAGAAAG
TTTGCACAATTAATATTAATGGATAAAAAACATGAAGAATTAGAACCTTGGGGAACAAGCAGAAAAACAG
AAAGAGGGGAAAAAGGATTTGGATCCACAGGGCTGTATTGGATAGAAAATATTCCTCTGGCAGAAGAGGA
TCACTCAAAATGGCATCAAGATGCTCGATCACTGCACCTAGAATTTGAAATCCCCAGAACAGCAGCAGAA
GACATAGTAAATCAATGTGAAGTATGTAGGGAAGAAAAGTCACCATCTTTAATCAGAGGGGGCAATAAAA
GGGGAGTAGATCATTGGCAAGTAGATTATACCCATTATGAAAACATAATACTATTAGTATGGGTAGAAAC
AAATTCAGGGCTAATATATGCAGAAAAGGTAAAAGGAGAATCAGGACAAGAATTCAGAATAAAAGTGATG
CAATGGTATGCATTATTTGGTCCAGAATCACTGCAGTCGGACAATGGACCTGCATTTGCAGCTGAAGCCA
CGCAGCTGTTAATGAAATACTTAGGGATAAAGCACACAACAGGCATACCTTGGAATCCGCAATCTCAAGC
AATAGTAGAAAGAGCACATCAACTATTAAAAAATGCTCTAAAGAAGTTTCAGCCACAATTTGTTGCTATA
GAATCAGCAATAGCAGCCGCCCTAGTCGCAATTAATATAAAAAGAAAGGGTGGGCTAGGGACAAGCCCTA
TGGATATTTTTGTATATAATAAAGAACAGAAAAGAATAAGTAATAAATATAATAAAAATTCTGAAAAAAT
TCAATTTTGTTATTACAGAGTAAGGAAAAGAGGACTTCCAGGAGAGTGGAAGGGACCGACTCAGGTACTG
TGGAAAGGGGAAGGAGCCATTGTCATAAAAGATACAGAGAGTGAAAAGTATTTAGTAATACCTTACAAAG
ATGCAAAATTCATTCCGCCGCCAACAAAAGAAAAGGAATAA

>FJ195346.1 Caprine arthritis encephalitis virus Ov496, complete genome
CTG
TGGAAAAAGAGGGCATATGCAAAAGGACTGCAGGGGGAAGAAAACAGGGATGCAGTCGGGAAATGGAAGG
AGGGGGCTACGTGTGGTGCCGTCCGCTCCCCCTATGGAATAACTACAGCACCACCAATGGTTCAGGTCCG
CATAGGTTCCAAATGGAGGAACTTATTATTTGACACAGGAGCGGACCGAACTATAATTAGATGGCATGAT
GGATCAGGGATTCCAGCGGGAAGAATAAAGCTACAAGGAATAGGAGGAATAGTAGAAGGAGAAAAATGGG
ATAATGTAAAAATAGAATACAAAGGAGAAACAAGAAAGGGACCAATAGTGGTCCTACCGCAAAGTCCAGT
AGAAGTACTAGGACGAGATAACATGGAAAAATTCGGCATAGAAATAATAATGGCAAATTTAGAGGATAAG
AAAATCCCAATTACTCAGGTACACTTGAAGGAAGGGTGTATGGGACCACATGTGCCACAATGGCCGTTGA
CGGAGGAAAAATTAAAAGGCCTAACAGAAATAATAGACAAATTATTAGAGGAAGGAAAACTAGGAAAGGC
ACCCCCACATTGGACATGGAATACGCCAATATTTTGCATAAAAAAGAAATCAGGAAAATGGAGAATGTTA
ATAGATTTTAGAGAATTAAATAAACAAACAGAAAATTTAACAGAGGCACAATTAGGACTTCCGCATCCGG
GAGGATTGCAAAAGAAAAAGCATGTTACAGTATTAGATATAGGAGATGCCTACTTTACAATCCCGCTATA
TGAACCATATCAAAAATATACATGTTTTACTTTATTAAGCCCTAATAATTTGGGACCATGTAAAAGGTAT
TATTGGAAGGTCCTTCCACAGGGATGGAAATTAAGTCCATCTGTGTATCAGTTTACCATGCAGAAGATAT
TAGAGGATTGGATACAACAACATCCAGACATTCAATTTGGTATATATATGGATGACATTTATATAGGAAG
TGATTTGGAAATTAAAAAGCATAGAAAAATAGTAAAAGAATTAGCAAACTATATTGCACAATATGGATTT
ACACTCCCGGAAGATAAAAGGCAGGAAGGCTATCCAGCAAAATGGCTGGGATTTGAACTACATCCGCAAA
CCTGGAAATTCCAGAAGCATACATTACCAGAATTAAGAATAGGGACAATTACTTTAAATAAATTGCAAAA
ATTAGTAGGAGAATTAGTGTGGAGACAATCCATAATTGGGAAAAGTATTCCTAATATTTTGAAATTAATG

GAAGGGGATAGAGCATTGCAAAGTGAAAGAAGAATAGAAGAAATACATGTAAAAGAATGGGAAGAATGCA
GGAAGAAATTAGCAGAAGCAGAAGGACATTACTTAGATCCAGAGAAAGATATCTATGGACAAATAGCCTG
GGGCAACAAAGCTATAGAATATATAGTATATCAAGAAAAAGAAAAACCATTGTGGGTAAATGTGGTTCAT
GACATAAAGAACCTGAGCATTCCCCAACAAGTTATTAAAGCAGCACAAAAATTAACACAAGAAGTAGTAA
TAAGAACAGGAAAAATACCTTGGATATTACTGCCAGGGAAAGAAGAAGATTGGAGATTAGAATTGCAATT
AGGAAATATAACGTGGATGCCAAAATTTTGGTCATGCTATAGAGGGCAAACTAGATGGAGAAGAAGAAAC
ATAACGGAAGAAATAGTAGAAGGGCCCACATATTACACAGATGGGGGGAAGAAGAATAAAGTAGGAAGTT
TAGGATTCATAACCTCAGCAGGGGAGAAAGTAAGACAGCATGAAGAAGGGACAAATCAGCAGCTTGAACT
GAGGGCAATTGAAGAAGCGCTAAAGCATGGACCAACCACTATGAATATAGTAACAGATAGCAGATATGCC
TTTGAATTTTTATTGAGAGACTGGGATGAAGAAACAATTAGGAATCCAATTCAAGCAAGAATTATGGAAA
TAGCACATAAGAAAAATAGGATAGGGATACATTGGGTACCAGGACATAAAGGAATCCCTCAGAATGAAGA
AGTAGATAGGTACATATCAGAAGTATTCTTAGCAAGAGAAGGGGAAGGAATTCTCCCAAAAAGAGAAGAA
GATGCAGGTTATGATCTACTCTGTCCAGAAGAAGTGATCATTGGGGCAGGGCAAGTCAAGGCAATCCCCA
TAGACTTAAGAATAAATTTAAAGGAGACTCAATGGGCCATGATTGCTACAAAAAGCAGCATGGCTGCAAA
AGGAGTATTCACACAAGGGGGAATTATAGATTCAGGATATCAGGGACAGATACAGGTAATAATTTATAAT
AGCAATAAAGTAGAAGTAGTAATACCTAGAGGAAGAAAATTTGCGCAATTAATATTAATGGAAAAAATAC
ATGAAGAGTTAGAACCTTGGGGAATAACAAGGAAGACAGAAAGAGGAACAAAGGGATTTGGATCAACAGG
AATGTATTGGATAGAGAATATCCCTATAGCAGAAGAAGAACACGCAAAATGGCACCAAGATGCTCAATCC
CTACATTTAGAATTTAACATACCAAGAACAGCAGCAGAAGACATAGTAAGCCAATGTGAGACCTGTCAAC
AGGAAAAAGCACCCTCTATTATAAGAGGAAGTAATAAAAGAGGGATAGATCACTGGCAAGTAGATTATAC
CCACTATGAGAACCATATACTATTAGTATGGGTGGAAACAAACTCAGGGCTAATATATGCAGAAAAAGTA
AAAGGAGAATCAGGACAAGAATTTAGAATAAAAGTAATGCAATGGTTTGCACTGTTTAGTCCAGAATCAT
TACAGTCCGACAACGGACCGGCTTTTGTAGCTGAGCCCACACAGCTGCTAATGAAATATTTAGGGATACA
GCATATGACAGGAATACCATGGAATCCACAATCACAAGCTTTAGTAGAAAGAGCCCATCAAACATTAAAA
CGTACAATAAACAAATTTAAAGATAGCTTCATAGCATTAGAATCAGCAATAGCAGCAGCCCTAGTAGCAA
TAAATATAAAAAGAAAGGGTGGGCTAGGGACAAGCCCTATGGACATTTTCATTTATAACAAAGAACAAAA
AAGAGTAACTAATAAATACAATAAAAATTCTGAAAAAATGCAATTTTGCTATTACAGAACAAGAAAAAGA
GGACACCCAGGAGACTGGGAAGGCCCGACCCAGGTATTGTGGAAAGGGGAAGGAGCCATAGTAATAAAAG
ACAAAAACTCAGAAAAATATTTAGTAGTACCTAACAAAGATGCAAAATTCATTCCGCCGCCAACAAAAGA
AAAGGGATAA

>HM210570.1 Small ruminant lentivirus strain FESC-752, complete genome
CTGTGGAAAAAGAGGGCATATGCAAAAAGATTGCAGAAGCAAGAGAGACATGAGAGGAAAAC
AGCAGGGAAACATGAGGAGGGGGATACGTGTGGTGCCGTCCGCTCCTCCTATGGAATAACTTCTGCACCA
CCAATGGTTCGGGTCCGCATAGGTTCCCAGCAGAGGGACTTATTATTTGATACCGGGGCGGACCGAACTA
TAGTAAAATGGCATGATGGCTCGGGAAACCCAGCCGGAAGAATAAAATTACAAGGAATAGGAGGGATAGT
GGAAGGAGAAAAATGGAATAATGTAGAATTGGAATATAAAGGAGAAACAAGAAAAGGAACAATAGTAGTG
TTACCACAAAGTCCAATAGAAGTATTAGGACGAGATAATATGGCCCGATTTGAAATAAAAATAATAATGG
CAAATTTAGAGGAAAAGAAAATCCCAATTACCAAAGTGAAATTGAAAGAGGGATGTAAGGGTCCACATGT
CCCTCAATGGCCGTTAACAGAAGAAAAATTGAAAGGACTAACAGAAATAGTAGATAAATTAGTGGCAGAA
GGAAAGCTAGGAAAGGCACCCCCGGATTGGACATGTAACACGCCAGTCTTCGCCATACGCAAGAAATCAG
GAAAGTGGAGAATGTTAATAGATTTCAGAGAATTGAACAAACAGACAGAAGATTTAACAGAAGCACAATT
AGGACTCCCTCATCCGGGAGGGTTAAAACAGAAAAAACATGTTACAATATTAGACATCGGGGATGCATAT
TTCACTATACCCTTATATGAACCCTATCGAGAGTATACTTGCTTTACTTTATTAAGTCCTAATAATTTAG
GACCATGTAAGAGATACTATTGGAAAGTGTTACCACAAGGCTGGAAACTGAGCCCATCTGTATATCAATT
TACCATGCAAAAGATTTTAGAGGATTGGATACAGCAACATCCTGAAATTCAATTTGGAATATACATGGAT
GATATTTATATAGGGAGTGATTTAGACATAAAAAGGCATAGGGAAATAGTGGAGGAATTAGCAAATTATA
TTGCCCAATACGGATTCACTCTGCCAGAAGAAAAAAGACAAGAAGGGTATCCAGCAAAATGGTTAGGATA
CGAACTACACCCGCAGACCTGGAAATTTCAAAAGCATACATTGCCTGAATTAAAAGAGGGGACAATTACC
TTAAATAAATTGCAAAAGTTAGTAGGAGAGTTAGTATGGAGACAGTCCATAATTGGAAAAAGTATCCCTA
ACATTCTAAAATTGATGGAAGGAGATAGGGCATTGCAAAGTGAAAGGAAAATTGAAGAGAAACATGTACA
AGAATGGGAAACATGTAGAAAAAGATTAGAAGAAATGGAAGGAAATTATTATGATGCAGAAAAAGATATC
TATGGACAATTGGCTTGGGGAGACAAAGCCATAGAATATATAGTGTATCAGGAAAAGGGAAAACCATTAT
GGGTAAATGTAGTTCATAATATAAAGAACTTAAGTATACCACAACAGATCATTAAAGCAGCACAAAAGTT
AACTCAAGAAGTAATCATTAGAACAGGAAAAATACCATGGATATTGCTGCCAGGGAAAGAAGAAGACTGG
CGACTGGAATTGCAATTAGGAAACATCACGTGGATGCCAAAATTTTGGTCCTGTTATAGAGGCCACACCA
GATGGAGAAAAAGAAACATAGTAGAAGAAGTAGTAGCAGGCCCTACATATTATACAGATGGAGGAAAAAA
GAATAAAACAGGAAATTTAGGGTTCATAGCATCAACAGGGGAAAAATTTAGAAGGCATGAAGAGGGCACA
AATCAGCAACTTGAACTAAGAGCCATTGAAGAAGCCCTAAAACAAGGACCTCAAAGGATGAATATAGTAA
CAGATAGCAGATATGCATTTGAATTTATGTTAAGAAATTGGGATGAAGAAGTGATTAAAAACCCAATTCA
AGCAAGAATTATGGAAATCGCCCACAAGAAAGACATGATAGGAGTGCATTGGGTGCCAGGACACAAAGGA

ATCCCACAAAATGAAGAAGTAGACCAATATATCTCAGAGATATTTCTTGCAAAAGAAGGAGAAGGAATTC
TCCCAAAAGGAGAGGAAGATGCCGGATATGATTTGATATGTCCAGAAGAGGTCACTATAGGACCAGGCCA
AGTGGCGAGGATCCCCATAGATTTAAGAATAAATTTAAAAGAGTCACAGTGGGCTATGATTGCTACAAAA
AGCAGCATGGCTGCCAAAGGAGTGTTCACACAAGGGGGCATCATAGATTCAGGATATCAGGGACAAATAC
AAGTAATAGTGTACAACAGCAATAAAGTAGAAGTGGTCATACCCCGAGGGAGAAAATTTGCACAACTAAT
TTTGATGAATAAGATACATGAAGAATTAGAACCCTGGGGGAAAAACAGAAAAACAGAAAGGGGAAAAGAA
GGATTTGGGTCCACAGGAATGTATTGGATAGAAAACATTCCTCTAGCAGAAGAAGAGCACACAAAATGGC
ATCAAGATGCGAACTCGTTGCATCTAGAATTTGAAATTCCTAGAACAGCAGCAGAAGACATAGTAAACCA
GTGTGAAATATGTCAAACAGAAAAAGCACCGTCCGTGATCAGAGGCGGCAACAAAAGAGGAGTAGATCAT
TGGCAGGTAGATTATACCCATTATGAAAATATCATAATCCTGGTATGGGTAGAAACAAATTCAGGGCTAA
TATATGCAGAGAAAGTAAAAGGAGAATCAGGGCAAGAATTCAGAACAAAAGTAATGCAATGGTATGCTCT
ATTTGGTCCAGAATCATTGCAGTCAGACAATGGACCTGCATTCACAGCAGAAGCCACACAACTATTAATG
CAATACTTAGGGATAAAACATACAACAGGGATACCTTGGAATCCACAGTCTCAAGCTATAGTGGAAAGAG
CACATCAACTCTTAAAAAGCAACCTCAGGAAATTTGAACCACAATTTGTTGCTGTAGAGTCAGCAATAGC
AGCAGCCCTAGTCGCCATAAATATAAAAAGAAAGGGTGGGCTAGGGACAAGCCCTATGGATATTTTTATA
TATAATAAAGAACAGAAAAGAATAAGTAATAAATATAATAAAAATTCTCAAAAAATGCAATTTTGTTATT
ACAGAGTAAGAAAGAGAGGACATCCAGGAGAGTGGAGAGGTCCAACTCAGGTACTGTGGAAAGGGGAAGG
AGCCATTGTAGTAAAGGATATAGAAAGTGAAAAGTATCTAGTGATACCTTACAAAGATGCAAAATTCATT
CCGCCCCCAACAAAGGAGAAGGAATAA

>MH374289.1 Small ruminant lentivirus isolate SRLV_Taccone, complete genome
CGGGGGACGGGGGATACGTGTGGTGCCGTCCGCTCCCC
CTATGGAATAACAGAAGCACCACCAATGGTTCGGGTCCGCATAGGTTCCCAGCAGAGGGACTTATTATTC
GATACTGGGGCGGACCGAACTATAGTAAAATGGCATGATGGCTCGGGAAACCCAGCCGGAAGAATAAAAC
TACAAGGAATAGGGGGCCTAGTAGAAGGAGAAAAATGGAACAATGTGGAATTGGAATATAAAGGAGAAAC
AAGAAAGGGAACAATAGTAGTGTTACCTCAAAGCCCAGTAGAAGTATTAGGAAGGGATAACATGGCCCGC
TTTGGTATAACAATAATAATGGCAAATTTAGAGGAGAAAAGAATCCCCATTACCAAAGTAAAGTTAAAAG
AGGGATGTAAAGGTCCTCATGTTCCGCAATGGCCGCTAACAGAAGAAAAATTAAAAGGACTAACAGAAAT
AGTAGATAAATTAGTGGAAGAAGGAAAACTAGGAAAAGCACCTCCACATTGGACATGTAACACGCCTATA
TTTTGCATAGCAAAGAAATCAGGGAAATGGAGAATGTTAATAGACTTCAGAGAATTAAATAAACAAACAG
AAGATTTAACAGAAGCACAACTAGGACTCCCACATCCAGGGGGTCTAAAAAGAAAGAAGCATGTTACAAT
ATTAGACATAGGAGATGCATATTTTACTATACCTTTATATGAACCCTATCGAAAATACACATGTTTTACT
CTATTAAGTCCGAATAATCTGGGACCATGTAAAAGATATTATTGGAAAGTTCTGCCACAAGGATGGAAAC
TGAGTCCTTCTGTATATCAATTTACCATGCAAAAGATTTTAGAGGACTGGATAAAGCACCATCCCGAAAT
TCAATTTGGAATATATATGGATGATATTTACATAGGAAGTGATTTAGAAATTAAAAAGCATAGAGAAATA
GTGGAAGAATTGGCCAGTTATATTGCCCAGTATGGATTTACCCTACCAGAAGAGAAAAGGCAAGAGGGGT
ATCCAGCAAAATGGCTAGGATTTGAACTACATCCTCAGACCTGGAAATTTCAGAAACATACATTGCCGGA
ATTAAAAGAGGGGGTAATTACATTAAACAAATTGCAAAAATTAGTAGGAGAATTAGTATGGAGACAATCC
ATAATTGGAAAAAGTATCCCTAACATTCTAAAATTGATGGAAGGGGATAGAGCATTGCAAAGTGAAAGAA
TAATTGAAGAGATACATGTAAAAGAATGGGAAGCTTGTAGAGAGAAATTAAAAGAAATGGAAGGAAATTA
CTATGATCCAGAAAAGGATGTTTATGGACAGCTAGCCTGGGGTGATAAGGCTATAGAGTATATAGTGTAT
CAGGAAAAAGGGAAACCATTATGGGTAAATGTAATGCACAATATAAAGAATCTAAGCATCCCACAACAAA
TTATTAAAGCCACACAAAAGTTAACTCAAGAAGTAATTATTAGGACAGGGAAAATACCATGGATATTATT
GCCCGGGAAAGAAGAAGATTGGAGATTAGAATTGCAATTAGGAAACATCACATGGATGCCAAAATTTTGG
TCATGTTATCGCGGGCAAACCAGGTGGAGAAAAAGAAATATAACAGAAGAAGTAGTAGAAGGACCTACAT
ATTATACAGATGGAGGAAAAAAGAATAAAACAGGAAATTTAGGGTTCATAGCATCAACAGGAGAAAAATT
TAGAAAGCATGAAGAGGGCACAAATCAACAGCTTGAATTAAGAGCCATTGAAGAAGCTCTAAAGCAAGGC
CCGCAGATAATGAATATAGTGACAGATAGCAGATATGCATTTGAATTTTTGCTAAGAAATTGGGATGAAG
AAGTAATTAAAAATCCAATTCAAGCAAGAATTATGGAGATAGCACACAAAAAAGACAAAGTAGGAGTGCA
TTGGGTGCCAGGACATAAAGGAATTCCTCAAAATGAGGAAATAGACCAATATATCTCAGACATATTTTTA
GCAAAAGAAGGAGAAGGAATTCTCCCAAAGAGAGAGGAGGATGCAGGATATGATTTGATTTGTCCAAAAG
AAGTCACTATAGGGCCAGGCCAAGTCGAAAAAATTGGCATAGATTTAAAATTAAATCTAAAAGAAACACA
ATGGGCTATGATTGCCTCAAAGAGCAGCATAGCTGCCAAAGGAGTATTCACACAAGGAGGAATCATAGAT
TCAGGATATCAGGGACAAATACAGGTAATAGTGTACAACAGCAATAAAGTAGCAGTAAGTATACCCCAAG
GGAGAAAATTTGCACAGTTAATCTTAATGGATAAGATACATGAGGAGTTAGAACCTTGGGGGACCAACAG
AAAAACAGAAAGAGGAGAACAAGGATTTGGATCCACAGGGCTATATTGGATAGAAAATATTCCTCTGGCA
GAAGAGGACCACGCAAAATGGCATCAGGATGCTCGATCATTGCATCTAGAATTTGAAATTCCCAGAACCG
CAGCAGAAGACATTGTAAAGCAATGTGAAATATGTAGAGAAGAAAAAACACCCTCTGTGATTAGAGGAGG
AAACAAAAGAGGAGTAGATCATTGGCAAGTAGATTATACCCATTACGAAAATAATATACTACTAGTATGG
GTAGAAACAAATTCAGGATTAATATATGCAGAAAAAGTGAAAGGAGAATCAGGACAAGAATTCAGAACAA

AAGTAATGCAATGGTATGCACTGTTTGGCCCAGAATCATTACAATCAGACAATGGACCAGCGTTCACGGC
TGAGCCCACACAGCTGTTAATGAAATACTTAGGAATAAAGCACACAACAGGCATACCGTGGAATCCACAA
TCTCAAGCAATGGTAGAAAGAGCCCATCAGCACTTAAAAGGGGCGTTAAGGAAATTTCAACCGCAATTTG
TCTCTATAGAATCAGCAATAGCAGCAGCCCTAGTCGCCATAAATATAAAAAGAAAGGGTGGGCTAGGGAC
AAGCCCTATGGATATTTTTGTATATAATAAAGAACAGAAAAGATTAAATAATAAATATAATAAAAATTTT
GAAAAAATTCAATTCTGTTATTACAGAGTAAGAAAAAGAGGACATCCAGGAGAGTGGAAAGGACCAACTC
AGGTACTGTGGAGAGGAGAGGGAGCCATTGTAGTAAAAGATACAGAAAGTGAAAGGTATCTAGTAATCCC
ATACAAGGATGCAAAATTCATCCCGCCGCCAACAAAGGAACAAGAATAA

>MH374290.1 Small ruminant lentivirus isolate SRLV_To1.89, complete genome
TGTGGTGCCGTCCGCTCCCCCTATGGATTAACAGATGCACCACTCATAGTTCAGGTCCGC
ATAGGTTCCCAGATGAGGGACTTATTATTTGACACCGGGGCGGACCGAACTATAATTAAATGGCATGATG
GCTCGGGAAACCCAGCCGGAAGAATAAAATTACAAGGAATAGGTGGGATAGTAGAAGGAGAAAAATGGAA
TGAGGTAGAATTGGAATATAAAGGAGAAAGAAGGAAAGGAACAATAGTAGTGCTACCGCAAAGTCCAATA
GAAGTACTAGGACGAGATAACATGGCCCGATTTGGGATACAGATAATAATGGCAAATTTAGAAGAAAAAA
GGATCCCAATTACAAAGGTAAAATTAAAAGAGGGATGTACAGGTCCTCATGTACCTCAATGGCCGTTAAC
AGAAGAAAAATTAAAAGGCCTGACAGAAATAATAGATAAATTGGTGGAAGAAGGAAAACTAGGAAAGGCA
CCCCCACATTGGACATGTAATACTCCAATATTTTGCATAAAAAAGAAATCAGGCAAATGGAGAATGTTAA
TAGATTTTAGAGAATTAAACAAACAAACAGAAGATTTAACAGAAGCACAATTAGGACTGCCACACCCGGG
AGGATTGCAAAAGAAAAAACATGTCACAATATTAGACATAGGAGATGCATATTTTACAATACCATTGTAT
GGACCCTACAGAGAATACACCTGTTTTACTCTATTAAGCCCTAATAATTTAGGACCATGTAAAAGATATT
ATTGGAAAGTGCTGCCGCAAGGGTGGAAATTAAGTCCTTCTGTATATCAATTCACCATGCAAGAGATCTT
AGAGGATTGGATACAGCAACATCCGGAAATTCAATTCGGGATATATATGGATGATATTTATATAGGGAGT
GATTTAGAAATTAAACAGCATAGAGAAAGAGTGGAAGAATTAGCCAATTATATTGCCCAGTATGGATTCA
CCCTGCCAGAAGAGAAAAGGCAAGAGGGATATCCAGCAAAGTGGCTAGGATTTGAATTACACCCCCAGAC
ATGGAAATTTCAAAAACATACATTACCGGCATTAACAGAAGGAACCATCTCCTTAAACAAATTACAAAAA
TTAGTAGGAGAATTAGTATGGCGACAATCCATAATTGGCAAAAGTATCCCCAATATCTTGAAATTATTGG
AAGGAGATAGGGCATTACAAAGTGAAAGAAAAATTGAAGAGATACATGTAAAAGAATGGGAGGCATGCAG
AAAAAGATTAGAAGAAATGCAAGGAAATTACTATAATAAAGAAAAAGACATTTATGGACAGCTAGCCTGG
GGTGACAAAGCAATAGAATATATAGTGTATCAGGAGAAAGGGAAACCATTATGGGTGAATGTGGTTCACA
ATATAAAGAATCTAAGCATACCGCAACAGATTATTAAAGCCACGCAAAAGTTAACCCAAGAAGTAATTAT
TAGAACAGGAAAAATACCATGGGTATTGTTGCCAGGGAAAGAAGAAGATTGGAGATTAGAATTGCAATTA
GGAAACATAACATGGATGCCAACATTTTGGTCGTGTTATAGAGGACACACCAGATGGAGAAAGAGAAACA
TAACAGAAGAAGCAGTAGAAGGCCCTACATATTATACAGATGGGGGAAGAAAGAATAAAACAGGAAGCTT
AGGATTTATAGCATCAACAGGAGAAAAATTTAGAAAACATGAAGAAGGTACAAATCAGCAACTTGAACTA
AGAGCAATTGAAGAAGCTCTAAAACAAGGTCCTCCAACAATGAATATAGTAACAGATAGCAGATATGCAT
TTGAATTTTTATTAAGGGATTGGGATGAAGAAGTAATTAAAAATCCAATTCAGGCAAGGATTATGGAAAT
AGCCCACAAAAAGGACAGGATAGGAGTACATTGGGTGCCAGGACATAAAGGAATCCCCCAAAATGAAGAA
ATAGACAAGTATATCTCGGAAATATTCCTTGCAAAAGAAGGAGAAGGAATTCTCCCAAAAAGAGAAGAGG
ATGCGGGATATGATTTAATATGCCCAGAAGAGGTCACTATAAGACCAGGAAAAGTCAAAACAATCCCCAT
AGATTTAAGATTAAATCTAAAAGAGTCACAATGGGCTATGATTGCTGCAAAAAGCAGCATGGCTGCTAAA
GGAGTGTTCATACACGGGGGAATCATAGACTCAGGATATCAGGGACAAATACAGGTAATAGTGTACAACA
GCAATGATGTAGCAGTGGTTATACCACAAGGGAGAAAGTTTGCACAATTAATCTTAATGGATAAAATACA
TGAAGACTTAGAACCGTGGGGGACAAGCAGAAAAACAGAAAGGGGGGAAAAAGGATTTGGATCCACAGGA
ATGTTTTGGATAGAAAATATTCCTCTAGCAGAAGAAGAACACGCGAAGTGGCATCAAGATGCCAGATCAC
TGCATCTAGACTTTGAAATTCCAAGAACAGCCGCGGAAGACATAGTAAACCAATGTGAAATATGCAAAGA
AGTAAAGACACCTTCCAATATTAGAGGCGGAAACAAAAGAGGGGTAGATCATTGGCAAGTAGATTATACC
CATTATGATAATAACATACTATTAGTATGGGTAGAAACAAATTCAGGATTAATATTTGCAGAAAAAGTAA
AAGGAGAATCAGGGCAGGAATTCAGAACAAAGGTAATGCAGTGGTATGCACTGTTCAGTCCAGAGTCATT
GCAGTCAGACAATGGACCTGCATTCGCAGCTGAGCCCACACAGCTGTTAATGAAATATTTAGGAATAACA
CATACAACAGGAATACCTTGGAATCCGCAATCTCAAGCTATAGTGGAAAGAGCGCATCAACTCTTGAAGA
GCGCTTTAAAGAAATTTCAGCCACAGTTTGCAACATTAGAATCTGCAATAGCGGCAGCCCTAGTAGCCAT
AAATATAAAAAGAAAGGGTGGGCTAGGGACGAGCCCTATGGATATTTTTGTATATAATAAAGAACAGAAA
AGATTAACTAATAAATATAACAAAAATTTTGAAAAAATTCAATTCTGCTATTACAGAGGAAGAAAAAGAG
GACATCCAGGAGAGTGGAAGGGACCGACTCAGGTACTGTGGAAAGGGGAAGGAGCCATAGTAGTAAAAGA
TACAGAGAGTGAAAAGTATTTAGTAATCCCCTACAAAGATGCAAAGTTCATTCCGCCGCCAACAAAGGAA
AAGGGATAA

>JF502417.1 Caprine arthritis encephalitis virus isolate Volterra, complete genome

CTGYGGGAAGCGCGGGCATATTCAAAGAGACTGCAGAAACAAAAAGCA
AGGAATGCAGCAGGGAAACATAAGGAGGGGGCCACGTGTGCTGCCGTCCGCTCCCCCTATGGAATAACTG
CAGCACCACCGATGATAAAAATAAATATAGGAGGTCATGAAAAGAATCTATTATTAGACACAGGAGCAGA
TAGGACAATAATAAGARAACATAATAGCACAGGCACCCCAAATGGAAGAATAATATTACAGGGAATAGGA
GGCTTAGTAGARGGAGAAAAATGGAAAAATGTAGACATAGAATATAAGGGATTAAAAATARAGGGAGAAG
TAGTAGTAATGATAACCACGCCTGTAGAAGTGTTAGGTAGGGATAACATGGAAAAGTTAGGGATAGGCAT
CATAATGGCCCATCTAGAGGAAGATAAAATACCTACAGTTAAAGTAAAATTGAAGGAAGGATGTACAGGA
CCCCATGTGCAACAATGGCCTTTAACAGCAGAAAAGTTACAGGGACTAACTGAAATTGTGAATAGATTAT
TAGAAGAAGGGAAGATAGGAGAAGCCCCTCCACATTGGACATGGAATACTCCCATATTCTGTATAAAGAA
AAAATCAGGAAAATGGAGAATGCTAATAGACTTTAGAGAATTAAACAAGCAAACAGAAGACTTGACTGAG
GCACAGTTAGGATTACCACACCCAGGAGGGCTACAGAAAAAGAAAAATGTAACAGTCTTAGATATTGGTG
ATGCATATTTCACGATACCTCTGTATGAACCATATAGAAAATATACATGTTTTACTTTGTTAAGCCCCAA
TAACTTGGGACCCTGCCGAAGGTATTATTGGAAAGTATTGCCTCAAGGATGGAAACTAAGCCCTTCTGTA
TATCAATTTACAATGCAAAAGATATTAACAAATTGGAGAAATGAACACCCAGAAATACAATTTGGAATAT
ATATGGATGATATATACTTAGGGAGTGACCTTGWAATAAAAGATCATAGAAGAATAGTAGAAGACCTAGC
AATGCAAATAGCAAAATATGGGTTTATGTTACCGGAAGACAAAAGACAAGAAGGATATCCAGCGAACTGG
CTAGGATTTGAGTTACATCCAAATACATGGAAATTTCAAAAACATAAACTACCGGAACTTCAAAAAGGCG
CCATAACCTTAAATAAATTGCAAAAATTAGTAGGAGATTTAGTGTGGAGACAATCATTAATAGGAAAAGG
CATACCTAATATATTAAAATTAATGGAAGGGGATCGAGATTTACAAAGTCATAGAGAGATCACAGAGGTC
CATATCCAGGAATGGGAAGAATGTAGGAAGAAATTACAACAAATGGAAGGGAGTTATTATCAAGAAGAAA
AAGATATATATGGACAATTAACATGGGGAAATAAAGTAATAGAATATATAGTGTTTCAAGAAAAAGGGAA
ACCATTATGGGTAAATGTAGTACATCAGATTAAGAACTTAAGCTTAGCTCAGCAAATTATAAAAGCAGCA
CAAAAATTAACTCAAGAAGTAATAATTAGGACAGGGAAAGTTCCCTGGGTAATGCTCCCAGGAAAAGAGG
AAGATTGGATCCTAGAATTGCAAACAGGAAATATTACATGGATGCCACCCTTTTGGTCATGTTATAGGGG
GTCACCCAGATGGAAAAGAAGGAACATAACAGAAGAGGTAGTAGAAGGGCCGACTTATTATACAGATGGG
GGAAAGAAAAATGGGATAGGAAGTTTTGGGTATATCTCATCAACAGGGGAGAAATTTAGAAAGCATGAAG
ATGGGACGAATCAACAACTAGAACTGAGAGCAATAGAAGAGGCATGCAAGAATGGGCCAGAAAAATTAAA
TATAGTAACAGATAGCAGATATGCATTTGAATTTATGAAAAGAAATTGGGATGAAGAAGTTATAAAGAAT
CCCATCCAAGCAAGAATAATGAAACTGTTACATGAGAAAAAAGCAGTAGGGATACATTGGGTTCCAGGAC
ATAAAGGGATTCCGCAAAATGAAGAGATAGATAAATATATATCAGAAGTATTCTTAGCAAAAGAAGGTGA
AGGTATAATGCCAAAAAGAAAGGAGGATGCAGGATATGATTTGATCTGTCCCCAGGAAATTAGTATTGAG
GCAGGTAAAGTAAAAAAAGTACCTGTAGATCTTAGATTAAATCTCAAGGAAAATCAATGGGCAATGATAG
GGACAAAAAGTAGTATGGCTGCTAAAGGAGTATTTACACAAGGGGGAATAATTGATTCAGGATATCAAGG
ACAGATACAAGTAATTGTATTTAATAGTAATAATTATGAAGTAGTAATACCTAAAGGGAGAAAATTTGCA
CAACTAGTAATCATGCCCTTAATACATGAAGAATTAGAATCATGGGGAAAAGAAAGAAGGACTGAGAGAG
GAAGCAAAGGATTTGRATCAACAGGAGCATATTRGATAGAAAGGATCCCGGAGGCAGAAGAAGATCACTA
TAAGTGGCATCAAGATGCAAGATCTTTACAATGGGAATTCGGAATACCTAGAATGGCAGCAGAAGAAATT
GTGCAACAATGTGAAGAATGTCAAAAAGAGAAACCCGCGGTTACCATAAGAGGGGGAAATAAAAGAGGAA
TAAATCATTGGCAAGTAGATTACACTCATTATGAAAATAACATAATATTAGTATRGGTAGAAACCAATTC
AGGGCTAATTTATGCAGAAAAAGTAAAAGATGAATCAGGAAAAGAATTTAGAATACAAGTAATGAAATGG
TATGCGCTATTTCAGCCAGAATCAGTGCAGTCAGACAATGGACCAGCCTTTGTAGCTGAACCTACACAGC
TATTAATGAAATATTTAGGAGTAAAACATACAACTGGAATCCCATGGAATCCCCAATCACAGTCAATTGT
AGAAAGAACCCATAAAAGTTTTAAAGATACTTTGAAAAAATTARAAAGTCAATTCGCAGCAATAGAATCA
GCAATAGCTGCTACCTTGGTCGCACTTAACATAAAAAGAAAGGGTGGGCTAGGGACAAGTCCTATAGATA
TATTTGTATATAATAAAGAACAAATAAGAATAGAAACAAATAATCAACAAAAATTTAAAAATAAAAAATT
TTGTTACTACAGGATAAGAAAAAAAGGCCACCCAGGACCTTGGGAAGGGCCAACAGAAGTACTGTGGCAA
GGAGAAGGGGCCATAGTRGTAAAAGATAAGTATTCAGAAAAATATATAGTAATAGCATATAAGGATGCAA
AATTTATTCCACCACCGCAGGGGACGGAAAGAACGGAACATAGGACCGCAATTGCCTCTTAG

>AY900630.1 Caprine arthritis-encephalitis virus strain gansu, complete genome

ATGCGAAATTCATCCC
GCCACCAGCAAAGGAGAAGGAATAGGGAACAGGGACCAGAATTACCATTAGCATTGTGGAAACATATAGC
AGAAAGCATCAATAAAGAGAGCTCATGGTACATAACAGTAAGACTACAACAGATGATGTGGAACAAAAGG
GGAAATAAATTACAATATAAAAATGAGGATAGGGAATATGAAAATTGGGAAATCACATCATGGGGATGGA
AAATGAAACTAAGGAGAGTAAAACAATGGATAAAAGAAGGAGGGAGAGGAACTCCATGGCAGTACAAAGT
AGCAGGGACATGGAAAAATATAGGAGTGTGGTTCCTACAAGCAGGAGATTACAAAAAGGTAGACAGGCAC
TTCTGGTGGGCATGGCGGATAATGTTGTGTTCATGCAGGAAAGAAAGATTTGACATAAGAGAATTCCTGA
GAGGAAGGCATAGATGGGATTTGTGCAAATCCTGTGCTCAAGGAGAAGTAGTAAAGCATACTAGAACAAA

AAGTCTGGAAAGACTAGTATTGCTGCAGATGGTAGAACAGCATGTGTTTCAAGTATTGCCACTGTGGAGA
GCCAGGAGAGGTAATACCATAGATTTCCCATGGTGCAGGGACACAACGGGATACACGCATGCGTGGTCTG
TCCAGGAGTGCTGGTTGATGGAATATCTCTTAGAAGATGA

>MG554414.1 Small ruminant lentivirus isolate SRLV020, complete genome
ATGGCGAGGCAAGTCTCCGGGGGGAAAAGGGAT
TATCCTGAGCTCGACAAATGTATCAAGCAAGCATGCAAGATAAAAGTTCGAGTCAGAGGGGAGCACTTGA
CAGAAGGAAATTGTTTGTGGTGCCTTAAAACATTAGATTATATGTTTGAGGAACACAAAGAGGAGCCTTG
GACAAAAGTAAAATTTAGAAACATATGGCAGAGGGTAAAAAATTTAACTCCTGAGGAGAGTAATAAAAAA
GACTATATGTCTTTGCAGGCCACATTAGCGGGGCTAATGTGTAGCCAAATGGGGATGAAACCTGAAACAT
TACAGGATGCAATAGCAACAATAAATATGAGAGATGGGGTATTAGAGCAAGAAGAGAAAAAAGAAGAAAA
GAGAGAAAAAGAAGAGAGTGTCTTCCCAATAGTAGTGCAAGCAGCAGGAGGAAGAAGCTGGAAGGCAGTA
GACTCAGTAATGTTCCAGCAACTACAAACAGTAGCAATGCAACATGGCATCGTGTCTGAGGAATTTGAGA
GGCAGTTAGCATATTATGCTACTACCTGGACAAGTAAAGATATATTAGAAGTATTAGCCATGATGCCTGG
AAATAGAGCTCAAAAAGAGTTAATTCAAGGGAAATTAAATGAGGAAGCAGAGAGGTGGAGAAGGAACAAT
CCACCACCTCAAGCAGGAGGAGGATTAACAGTGGATCAAATTATGGGGGTGGGACAAACAAATCAGGCCG
CAGCACAAGCCAACATGGATCAGGCAAGACAAATATGCCTACAATGGGTAATATCAGCATTAAGGGGGGT
AAGGCATATGGCTCACAAACCAGGAAATCCAATGTTAATAAAGCAAAAAACAAATGAGTCATATGAAGAT
TTTGCAGGAAGACTGCTAGAAGCAATAGATGCAGAACCAGTTACCCAGCCTATAAAAGAATATCTAAAGC
TAACATTATCGTATACAAATGCATCCTCAGACTGTCAAAAGCAAATGGATAGAGTACTAGGACAGAGAGT
ACAACAAGCTACGGTAGAAGAAAAATTGCAGGCATGCAGAGATGTGGGATCAGAAGGATTCAGAATGCAA
TTGTTAGCACAAGCGTTGAGGCCAGAAAGAGGGAAAAGGAATGGGCCATCACAAAGGTGCTATAATTGTG
GAAAACCAGGACACGTAGCAAAGGAATGCAGACAGGGCATCATCTGTCATAATTGTGGGAAAAGAGGACA
TGTGCAAAAAGATTGTAGAAGAAAGAAAGGAGAAAGACAGCAGGGAAACGGACGACGGGGGATACGTGTG
GTGCCGTCCGCTCCCCCTATGGAATAA

>DQ844925.1 Small ruminant lentivirus isolate NMV1 pol polyprotein
(pol) gene, partial cds
GAGCAAGTACAATTGCAATATAAAGATAAAATAAGCAGAGGAACCATAGTGGTGTTAGCTACAAGCCCGG
TAGAAGTATTAGGAAGAGACAATATGAGCGAACTAGGAATAGGATTAATTATGGCAAATTTAGAAGAAAA
GAAAATTCCCATTACAGAAGTAAGATTAAAAGAGGGATGTAAGGGACCCCACATAGCGCAATGGCCGTTG
ACGCAAGAAAAATTAGAGGGATTAAAAGAGATAGTAGACAGATTAGAGAAGGAAGGGAAAGTAGGAAGAG
CGCCCCCACACTGGACTTGTAATACCCCTATATTTTGTATTAAGAAGAAATCAGGAAAATGGAGAATGTT
AATAGATTTTAGGGAATTAAATAAGCAAACAGAAGATTTAGCAGAAGCACAGTTAGGGTTACCGCATCCA
GGGGGATTAAAGAAAAAGAGACATGTAACAATATTAGATATAGGAGATGCATATTTTACAATACCATTAT
GTGAGTCATATAGACAATATACATGTTTTACCATGTTAAGTCCAAATAATTTAGGACCATGTGTAAGATA
TTATTGGAAGGTGTTACCACAAGGATGGAAGTTAAGTCCTTCAGTATATCAATTTACAATGCAAAAAATA
TTAAGAGGATGGATAGAAGAACATCCTATGATA

>MN784744.1 Small ruminant lentivirus isolate WV.14.5_Sheep.BEL pol
protein (pol) genes, partial cds
GGGAAACAGCAGGAGGGGGCC
ACGTGTGGTGCCGTCCGCACCCCCTATGTTGTAACAGAAGCACCACCAAACATAGAAATAAAAGTAGGAA
AATTATGGAAAAGAATGTTAGTAGACACAGGGGCAGATAGAACCATAGTAAGATATCATGATAATTCGGG
GGTACCAAAAGGAAGGATAAAATTACAAGGTATAGGTGGAATTATAGAAGGGGAAAAATGGGAGCAAGTT
AACATCCAATATAAAGAAAAACTAATAAAGGGAACACTAGTAGTGCTGCCTAGCAGCCCAGTAGAAGTAT
TAGGACGAGATAATATGAGGGAGCTAGGAATAAGCTTAATAATGGCAAATTTAGAAGAAAAGAAAATTCC
CATTACTAAAGTAAAATTAAAAGAAGGATGTCACGGACCACATATAATGCAATGGCCTTTAACACAAGAA
AAATTAGAAGGGTTAAAAGAAATAGTAGATAAATTGGAAAAGGAAGGGAAACTAGGTAGAGCTCCTCCGC
ATTGGACATGTAATACCCCTATATTTTGTATTAAGAAGAAATCAGGGAAATGGAGGATGTTAATAGATTT
TAGAGAATTAAATAAACAAACAGAAGATTTAACAGAGGCTCAATTAGGATTACCGCATCCTGGCGGGTTG
CAAAGAAAGAAACATGTAACAATATTAGATATCGGGGATGCGTATTTCACCATACCGTTATATGAGCCAT
ATAGGCAATATACATGTTTCACTATGTTAAGTCCAAATAATTTAGGGCCATGTACAAGATATTATTGGAA
GGTGTTACCACAAGGTTGGAAGTTAAGTCCTTCAGTGTATCAATTTACAATGCAGGAAATATTAAGGGAA
TGGATAAAGGCGCACCCTCAAGTACAGTTTGGAATATATATGGATGATATCTATATTGGAAGTGATT

>MN784752.1 Small ruminant lentivirus isolate OV.13.1_Sheep.BEL;
nonfunctional pol protein (pol) gene, partial sequence
GGGAAACAACAGGAGGGGGCC
ACGTGTGGTGCCGTCCGCACCCCCTATGCTGTAACAGAAGCACCACCCAAAATAGAAATAAAAGTAGGAA
GAAATTGGAAAAAAGTGTTAGTAGATACAGGGGCAGATAGGACTATAATAAAGTATCATGATAATTCGGG

AATACCAAAAGGAAGAATACAATTGCAGGGCATAGGAGGAATTATAGAAGGGGAAAAATGGGATAACGTA
GAAATACAGTATAAGGATAAGATAATAAAAGGAGCAATTGTAGTGCTGCCTAGCAGCCCAGTAGAAAGTA
TTAGGAAGGGATAATATGGGAGAATTGGGAATAAGATTAATTATGGCAAATTTAGAAGAAAAGAAAATTC
CCATTACTAAAGTGAAATTAAAAGAGGGATGTAAAGGACCTCATATAGCGCAATGGCCGTTGACGCGGGA
AAAGCTAGAAGGATTACAAGAAATAGTAGATAGATTAGAAAAGGAAGGAAAACTAGGGAGGGCACCTCCG
CATTGGACATGTAACACCCCTATATTCTGTATTAAGAAAAAAATCGGGGAAATGGAGAATGTTAATAGATT
TTAGAGAATTAAATAAACAGACAGAGGACCTAGCAGAGGCACAATTGGGGACTGCCGCATCCAGGAGGAT
TACAGAAGAAGAAGCATGTAACAGTATTAGATATAGGGGATGCATATTTTACAATACCATTGTATGAGCC
TTATAGGCAATATACATGCTTCACTATGTTAAGTCCAAATAATTTAGGGCCTTGTGTAAGGTATTATTGG
AAGGTATTGCCACAAGGATGGAAATTAAGTCCTTCAGTATATCAATTTACAATGCAGGAAATATTGAGAG
GGTGGATAAGGCAACACCCTATGATACAATTTGGAATATATATGGATGATATTTACATAGGAAGTGATT

>KJ641393.1 Small ruminant lentivirus strain 2-20 pol protein gene, partial cds
ATGCCACCATTGTGGAAAAAGAGGACATATGCAGAAGGATTGCAGGAAAA
AGAAAAGAGATATGAAGCAGCAGGGAAACAGCAGGAGGGGGCCACGTGTGGTGCCGTCCGCGCCCCCTAT
GCTGTAACAGAAGCACCACCGAGAATAGAAGCAAGAGTAGGGACAACTTGGAAGGACTTATTAGTGGATA
CAGGAGCAGACAGAACCATAGTAAGAAAACACGATAATTCAGGGATACCAAGGGGAAGAATAAAATTACA
AGGAATAGGAGGGATTATAGAAGGGGAAAGATGGGATCAAGTACAAATACAATATAAAGAAAAAACAATA
AGAGGAACAATAGTGGTACTACCGGCAAGTCCAGTAGAAGTGTTAGGAAGAGATAATATGGGAAAGTTGG
GCATAGGATTAATTATGGCAAATTTAGAAGAAAAGAAAATTCCCATAACGCGGGTAAAATTGAAGGAGGG
ATGTAAGGGACCCCATGTAGCACAGTGGCCTTTGACTCAAGAAAAATTGGAAGGATTAAAGGAAATAATA
GACAGATTAGAAAAAGAGGGAAAGCTAGGCAGAGCACCACCGCATTGGACATGTAATACCCCTATCTTCT
GTATTAAGAAAAAATCGGGGAAATGGAGAATGTTAATAGATTTTAGGGAATTAGATAAGCAAACAGAAGA
TTTGGCGGAAGCACAGCTAGGGCTACCGCATCCAGGGGGATTAAAAGGAAAGAAAAATGTAACGATCCTC
GATATTGGGGATGCGTATTTTACAATACCATTATATGAGCCTTATAGACAATATACATGTTTTACTTTGC
TAAGTCCAAATAATTTGGGACCATGTGTTAGATACTATTGGAAGGTGCTGCCGCAGGGATGGAAGTTAAG
TCCGTCAGTGTATCAGTTTACAATGCAGAAAATATTAGAAAATTGGATAGAGGGACATCCTATGATACAG
TTTGGGATATATATGGATGATATTTATATAGGAAGTGATCTAGAGATAAAGGAGCATAGAAAGATAGTAG
ATGACTTAGCAAGTTATATAGCACAATTTGGCTTCATGTTGCCTGAAGATAAAAGACAGGAAGGGTATCC
AGCCAAGTGGCTAGGATTTGAACTACACCC

>MN784755.1 Small ruminant lentivirus isolate LX.2.1_Sheep.BEL nonfunctional pol protein (pol) gene, partial sequence
GGGAAACAACAGGAGGGGGCCACGTGT
GGTGCCGTCCGCGCCTCCTATGTTGTAACAGAAGCACCACCAAACATAGCACTAAAAGTAGGGACAAATT
GGAAAAAGGTATTAGTAGATACAGGGGCAGATCGGACCATAGTAAGAAATCATGATAATTCGGGAGTACC
TAAGGGGAGGATAAAATTACAAGGTATAGGAGGAATTATAGAAGGGGAAAAATGGGATAATGTAGCCCTA
CAATATAAAGAAAAATTGATAAGGGGATCAATAGTAGTGCTGCCTAGCAGCCCAGTAGAAAGTATTAGGA
AAGGGATAACATGGGAGAAATTGGGTATAGGATTGATTATGGCAAATTTAGAAGAAAAAAAGAATTCCTA
TTACTAAGGTAAGATTAAAAGAAGGCTGTAAGGGACCTCATATAGCACAATGGCCTTTAACACAGGAGAA
ATTAGAGGGACTACAAGGAATAGTGGACAGATTAGAGAAGGAAGGAAAATTAGGAAGAGCGCCCCCACAT
TGGACATGTAACACTCCTATATTTTGTATTAAGAAAAAATCGGGGAAATGGAGAATGTTAATAGATTTCA
GAGAATTAAATAAACAAACAGAGGATCTAGCGGAAGCACAATTAGGGCTGCCGCATCCGGGGGGATTACA
GAAAAAGAAGCATGTCACGATATTAGATATAGGGGATGCATATTTTACAATACCTTTATATGAGCCTTAT
AGACAATACACATGTTTCACTATGTTGAGTCCCAATAATTTGGGTCCTTGTGTAAGGTATTATTGGAAAG
TGTTACCACAAGGATGGAAATTAAGTCCTTCAGTGTATCAATTCACGATGCAAGAAATATTAAGAGATTG
GATAAAAGCACACCCCATGATACAATTTGGGATATATATGGATGACATCTATATAGGAAGTGATT

>MN784756.1 Small ruminant lentivirus isolate LK.6.3_Sheep.BEL nonfunctional pol protein (pol) gene, partial sequence
GGGAAACAACAGGAGGGGGCC
ACGTGTGGTGCCGTCCGCTCCCCCTATGTTGTAACAGAAGCACCACCAAAGGTAGAGATAAAGGTAGGGA
CAAATTGGAAAAGATTACTAGTGGACACGGGAGCAGATAGAACTATAGTAAGATGTCATGATAATTCGGG
AATACCAAAAGGGAGGATAAAATTACAGGGTATAGGCGGAATTATAGAAGGGGAAAAATGGGATAAAGTA
ACAATACAATATAGAGACAAAATAATAAGGGGAACAATAGTAGTGTTGCCTAGCAGTCCAGTAGAAGTAT
TAGGAAGGGATAACATGGGAGAATTAGGAATAGGTTTAATTATGGCCAATCTAGAAGAAAAGAAAATCCC
CATTACAAAGGTAAAGTTAAAAGAAGGATGCAATGGACCACACATAGCGCAGTGGCCTTTAACTCAAGAA
AAACTGGAGGGACTAAAAGAAATAGTGGATAGGTTAGAGAAGGAAGGGAAATTAGGTAGGGCACCTCCAC
ATTGGACATGTAATACTCCTATATTTTGCATTAAGAAGAAATCAGGGAAATGGAGGATGTTAATAGATTT
TAGGGAATTAAATAAGCAAACAGAAGAGCTAGCAGAGGCTCAATTAGGACTGCCGCATCCCGGGAGGATT

GCAGAAAAAGAAGCATGTAACAATATTAGATATAGGGGATGCATATTTTACAATACCTTTTGTATGAGCC
ATATAGACAATATACATGCTTCACTATGTTGAGTCCCAATAATTTAGGTCCTTGTGTAAGATATTATTGG
AAGGTGTTACCACAAGGATGGAAGTTAAGTCCTTCAGTGTATCAGTTCACGATGCAAGAAATATTAAGAG
ATTGGATAAAGGCACACCCCCTGGTACAATTTGGGATATATATGGATGATATTTACATAGGAAGTGATT

>AY454175.1 Small ruminant lentivirus isolate SNCR5560 nonfunctional pol protein gene, partial sequence
ATGCCATCACTGTGGAAAGAGAGGGCATATGCAAAAGGAC
TGTAGGCAAAAACAAAAGGGACAGAAGGGACAGCAGCAGGGAAACAGCCGGAGGGGGCCACGTGTGGTGC
CGTCCGCGCCTCCTATGCTGTAACAGAAGCACCACCTATAGTAAAAATACAAATGGGGACAATTTGGAAA
AAAGTATTAGTGGATACAGGGGCCGATAGGACAATAGTAAGATATCATGATAATTCGGGAACGCCAAAAG
GGAGAATAAAATTACAAGGAATAGGGGGAATAATAGAAGGAGAAAAGTGGGACAATGTAATAATACAGTA
TAAGGAGAAGATAGTAAGGGGAACAATAGTGGTGCTGCCTAGCAGCCCAGTAGAAGTATTAGGGAGAGAT
AATATGGGAAAGCTAGGAATAGGTCTAGTTATGGCAAATTTGGAAGAAAGGAAAATTCCGATCACGGAAG
TAAAGTTAAAGGAGGGATGTAAAGGACCACATATAGCCCAGTGGCCTCTAACACAAGACAAGTTAGAAGG
ATTAAAAGAAATAGTGGATAGATTAGAGAAAGAAGGAAAGTTAGGAAGAGCACCACCGCATTGGACTTGT
AATACACCTATATTCTGTATAAAGAAAAAGTCAGGGAAATGGCGAATGTTAATAGATTTTAGGGAATTAA
ATAAACAGACAGAAGATTTAGCAGAAGCACAATTAGGCTTGCCGCATCCAGGAGGATTACAGAAAAAGAA
AAATGTAACAGTATTAGATATAGGAGATGCATACTTTACCATACCATTATATGAGCCATATAGACAATAT
ACTTGTTTTACTTTACTAAGTCCCAATAATCTGGGACCGTGTGTAAGATATTATTGGAAAGTATTACCAC
AAGGATGGAAATTAAGTCCCTCAGTGTATCAATTTACAATGCAGAAAATATTAAGGGACTGGATAAAAGA
ACATCCTATGATACAATTTGGAATTTATATGGATGATATTTACATAGGGAGTGATTTAGACATAGGAGAG
CATAGGAAAACAGTAGACGAGTTAGCAAGTTATATGGCACAATATGGCTTCATGTTACCGGAAGATAAAA
AGCAAGAAGGATATCCGGCTAAATGGCTT

>MN784758.1 Small ruminant lentivirus isolate LK.3.3_Sheep.BEL nonfunctional pol protein (pol) gene, partial sequence
GGGAAACAACAGGAGGGGGCCACG
TGTGGTGCCGTCCGCTCCCCCTATGTTGTAACTGAAGCACCACCGAAAATAAGAGCGAAAATAGGAATAA
ATTGGAAAGAATTATTAGTGGATACAGGAGCAGATAGAACCATAGTAAGAAATCATGATAATTCGGGAAT
ACCGAAAGGGAAAATAAAATTACAGGGTATAGGCGGAATTATAGAAGGGGAAAAATGGGATAAAGTAACA
ATACAATATAGAGATAAAATAATAAGGGGAACAATAGTAGTGTTGATAGCAGCCCGGTAGAAGTATTAGG
AAGGGATAATATGGGAGAATTAGGAATAGGATTAATTATGGCAAATTTAGAAGAAAAGAAGATACCTATT
ACCCAAGTGAGATTGAAAGAAGGATGAAGGGCCCATATACCGCAGTGGCCTCTAACTCAAGAAAAATTAG
AAGGACTAAAAGAAATAGTGGATAGGTTAGAAAAGGAAGGAAAATTAGGTAGGGCACCTCCACATTGGAC
ATGTAATACTCCTATATTTTGCATTAAGAAGAAATCAGGGAAATGGAGGATGTTAATAGATTTTAGGGAA
TTAAATAAGCAAACAGAAGACCTAGCAGAGGCTCAATTGGGACTGCCGCATCCCGGAGGATTGCAGAAAA
AGAAGCATGTAACAGTATTAGATATAGGGGATGCATATTTTACAATACCTTTGTATGAGCCATATAGCAA
TATACATGCTTCACTATGTTGAGTCCAAATAATTTAGGACCATGTACAAGATATTATTGGAAAGTATTGC
CGCAAGGATGGAAGTTAAGTCCTTCAGTGTATCAATTTACAATAAGAAATATTAAGAGATTGGATAAAGG
CCCCCCATGATACAATTTGGGATATATATGGATGATATCTATATAGGGAGTGATT

>MN784754.1 Small ruminant lentivirus isolate OV.1.7_Sheep.BEL nonfunctional pol protein (pol) genes, partial sequence
GGGAAACAACAGGAGGG
GGCCACGTGTGGTGCCGTCCGCGCCTCCTATGCTGTAACAGAAGCACCACCAGAGATAGGAATAAAAGTG
GGAACAAAGTGGAAAAGGGTATTAGTAGATACAGGAGCAGATAGGACGATAGTAAGATATCATGATAATT
CGGGAATACCGAAAGGGAGAATAAAGTTGCAGGGTATAGGGGGAATTATAGAAGGGGAAAAATGGGATAA
TGTAACCCTACAATATAAAGAGAAAATAATAAAAGGGGCAATAGTAGTGCTGCCTAGCAGTCCAGTAGAA
GTATTAGGAAGGGATAATATGGGAGAATTAGGTATAGGATTAATTATGGCAAATTTAGAAGAGAAGAAGA
TCCCTATTACAAAAGTAAAATTAAAAGAAGGCTGTAAAGGACCTCATGTAACACAATGGCCTATAAACGC
AAGAAAAGTGGGAGGGAATTAAAAGAGATAGTGGACAGACTAGAGAAAGAAGGAAAATTAGGAAGAGCGC
CCCCACACTGGACATGTAATACTCCTATCTTCTGCATTAAGAAAAATTTCGGGGAAATGGAGAATGTTAA
TAGATTTTAGAGAATTAAATAAACAAACAGAGGATCTGGCGGAAGCGCAATTAGGGTTACCGCATCCGGG
GGGATTACAAAAAAAGAAGCATTGTTACTATACTAGATATAGGGGATGCATATTTTACAATACCTTTGTA
TAAGCCGTATAGACAATATACATGCTTCACTATGTTGAGTCCCAATAATTTAGGGCCTTGTGTAAGGTAT
TATTGGAAGGTGTTACCACAAGGATGGAAATTAAGTCCGTCAGTGTATCAATTTACAATGCAAGAAATAT
TAAGAGATTGGATAAAAGCACACCCCTTGATACAATTTGGAATATATATGGATGATATTTATATAGGAAG
TGATT

>MN784750.1 Small ruminant lentivirus isolate VB.1.2_Sheep.BEL gag protein (gag) gene, partial cds; and nonfunctional pol protein (pol) gene, partial sequence
GGGAAACAGCAGGAGGGGGCC
ACGTGTGGTGCCGTCCGCACCCCCTATGTTGTAACAAACGCACCACCAAATGTAGAAATAAGAGTAGGGA
AAAATTGGAAAAGAATGTTAGTAGATACAGGGGCAGATAGGACTATAGTAAGGTATTCCATGATAATACG
GGGATACCAAAGGGAAGAATAAAATTGCAGGGTATAGGAGGAATTATAGAAGGGGAAAGATGGGAAAATG
TAGAAATACAGTATAAAGGAAAGAGAATGTGGGGAACAATTGTGGTGCTGCCTAGCAGCCCAGTAGAAGT
ATTAGGAAAGAGATAATATGGGGGAATTAGGAATAACATTAGTTATGGCAAATTTAGAAGAGAAGAAAAT
CCCTATTACTAAAGTAAAACTGAAAGAAGGGTGTAAGGGACCTCATATAGCGCAATGGCCGTTAACACAA
GAGAAAATTAGAGGGATTGCAAGAAATAGTAGATAGATTAGAGAAAGAAGGAAAAGTAGGAAGAGCTCCT
CCACATTGGACTTGTAACACCCCTATATTTTGTATTAAGAAAAAATCAGGAAAATGGAGGATGTTAATAG
ATTTTAGAGAATTAAATAAACAAACAGAAGATTTAGCAGAGGCACAATTGGGATTGCCACATCCAGGGGG
GTTACAGAAAAAGAAGAATGTAACAATATTGGATATAGGGGATGCATATTTTACATACCTTTTGTTATGA
ACCGTATAGACAATATACATGTTTTACCATGTTAAGTCCAAATAACCTAGGACCTTGTACAAGGTATTAT
TGGAAGGTATTACCACAAGGATGGAAATTAAGCCCCTCAGTATATCAATTTACAATGCAAGATATATTAA
GGGATTGGATAAAAGAGCACCCTAGGATACAATTTGGGATATATATGGATGACATCTACATAGGAAGTGA
TC

>KJ641396.1 Small ruminant lentivirus strain 20-2 pol protein genes, partial cds
ATGCCATCATTGTGGAAAAAGAGGGCATATGCAAAAAGATT
GCCGGCAAAAGAAGAAGCAAGATATAAAGCAGCAGGGAAACAGCAGGAGGGGGCCACGTGTGGTGCCGTC
CGCGCCCCCTATGTTGTAACGGACGCACCACCAAGGATAAAAGTAAAAATAGGAACAAAGTGGAAAGAAG
TACTAGTAGACACAGGAGCAGATAGGACTATAGTTAAAAATCATGATAATTCCGGGATACCAGGGGGAAG
AATAAAATTACAGGGAATCGGGGGGATCATAGAAGGAGAAAAGTGGGATCAAGTGCCACTACAATATAAA
GAAAAAATTATACTCGGCACAATAGTGGTACTACCAAGTAGTCCAGTAGAAGTGTTGGGGAGGGATAATA
TGAGAAAAGTAGGAATAGGGCTAGTTATGGCTAATTTAGAAGAAAGAAAAATCCCCATTACAGAAGTAAA
ATTAAAAGAGGGATGTAAAGGACCCCATATAGCTCAGTGGCCTTTAACTCAAGAAAAATTGGAAGGATTA
AAAGAAATTGTAGAAAGATTAGAGAAAGAAGGGAAACTAGGAAGAGCACCACCACATTGGACATGCAATA
CACCAATATTTTGCATTAAGAAAAAATCAGGGAAATGGAGAATGTTAATAGATTTTAGGGAATTAAACAA
GCAAACGGAAGACTTGGCGGAGGCACAGTTAGGACTACCGCATCCCGGGGGATTGAAGAAGAAGAAAAAT
GTAACAGTCCTTGATATTGGGGATGCGTATTTCACAATACCATTATATGAACCTTATAGACAATATACAT
GCTTTACTCTGCTAAGTCCAAATAATTTGGGACCATGTGTTAGATATTATTGGAAGGTGTTGCCGCAAGG
ATGGAAGTTAAGTCCATCGGTATATCAGTTTACAATGCAGAAGATATTAAAAGATTGGATAGAGGCACAT
CCTAGGATACAGTTTGGGATATATATGGATGATATCTACATAGGAAGTGATCTAGAGAGAACAGAACATA
GAGAAATAGTGGAGGAATTAGCAAATTACATAGCACAATTTGGCTTCATGTTGCCTGAAGACAAAAGGCA
GGAGGGGTACCCGGCAAAATGGCTAGGATTTGAACTACACCC

>JX660694.1 Small ruminant lentivirus isolate 4007 nonfunctional pol protein (pol) gene, partial sequence
CAGAAGCACCACCTAGAATAAAAGTAAAAATAGGGACAAATTGGAAAGAAG
TACTTGTGGACACGGGGGCTGATAGAACTATAGTAAGAAGACATGATAATACAGGGATACCGCGAGGAAG
AATAAAATTACAGGGAATAGGAGGAATTATAGAAGGGGAAAAATGGGATCAAGTGCAAATACAATATAGA
GAAGAAATTATAAAAGGAACAATTGTGGTGCTACCAAGTAGCCCAGTAGAGGTATTAGGAAGAGATAACA
TGGGGAAATTGGGGATAGGGTTAGTTATGGCAAATTTAGAAGAAAGAAAAATCCCCATCACGGAAGTAAG
ATTAAAAGAGGGATGTAAAGGACCTCATATAGCACAATGGCCCTTAACCCAAGAAAAGTTGGAAGGGTTA
AAAGAAATTGTAGATAGATTAGAGAAGGAAGGAAAATTAGGAAGAGCACCACCACATTGGACATGTAATA
CCCCAATATTTTGCATCAAGAAAAAATCAGGAAAATGGAGAATGCTGATAGACTTTAGAGAATTAAACAA
ACAGACAGAAGATTTGGCAGAGGCACAATTAGGGTTACCGCATCCTGGGGGGCTAAAGAAAAAGAAACAT
GTAACAATTCTTGATATTGGGGATGCATATTTTACAATACCATTGTATGAGCCTTATAGGCCATATACAT
GCTTTACTCTGCTAAGTCCAAATAATTTGGGGCCATGTGTAAGGTATTATTGGAAAGTGTTGCCTCAAGG
ATGGAAGTTAAGCCCATCAGTGTATCAATTTACAATGCAGAGAATATTAAAAGATTGGATAAAAGAACAT
CCTATGATACAGTTTGGAATATATATGGATGATATTTACATAGGAAGTGACCTAGAGATCAGAGAACATA
GACAAATAGTAGATGAACTAGCAGGTTATATAGCACAATTTGGGTTTATGCTGCCAGAAGATAAAAGACA
GGAAGGATATCCGGCCAAGTGGCTAG

>AY454208.1 Small ruminant lentivirus isolate SNCR5692 nonfunctional pol protein gene, partial sequence
ATGCCATCATTGTGGAAAAAGAGGGCATATGCAAAAGGAC
TGTCGGCAAAAGAGAAACAATAACAAGACTCAGCCGGGAAACACCGGGAGGGGGCCACGTGTGGTGCCGT

CCGCGCCCCCTATGTTATAACAGAAGCACCACCTAGGGTAAAAGTAAAAATAGGGACAGTTTGGAAAGAA
GTACTAATAGATACGGGGGCAGATAGGACTATAGTTAGACATCATGATAATTCAGGGATACCCACGGGAA
GAATAAAATTACAAGGAATAGGGGGAATCATAGAAGGAGAAAAATGGGATCAAGTACTATTGCAATATAA
AGGAGAAAGTATAAAAGGAACACTTGTGGTATTGCCGACGAGTCCTGTAGAAGTATTAGGGAGAGATAAT
ATGAACAAATTAGGAATAGGGTTAATTATGGCAAATTTAGAAGAAAGGAAAATCCCCGTCACAGAAGTAA
GGTTAAAAGAGGGATGTAAAGGACCAAGTATAGCACAATGGCCCTTAACCCAAGAGAAGTTAGAAGGGCT
AAAAGAAATTATAGATAGGTTGGAGAAAGAAGGAAAATTGGGGAGAGCGCCGCCACATTGGACATGTAAT
ACACCTATATTTTGTATTAAGAAAAAATCAGGGAAATGGAGAATGTTGATAGATTTCAGGGAATTAAACA
AGCAAACAGAAGATTTAGCAGAGGCACAGCTGGGACTACCACATCCCGGAGGATTGAAAAAGAAGAAGAA
TGTCACAATCCTCGATATTGGGGATGCGTATTTTACAATACCATTGTATGAACCTTATAGACAGTATACA
TGCTTTACTCTGCTAAGTCCAAATAATTTGGGACCATGTGTGAGATATTATTGGAAAGTGTTGCCTCAGG
GGTGGAAACTAAGTCCGTCAGTATATCAGTTTACAATGCAAAAAATCTTAAGGGATTGGATAAAAGAACA
TCCTATGATACAGTTTGGGATATATATGGATGACATTTATATAGGAAGTGATTTAGAGATAACAGCACAT
AGAAAAATAGTGGAGGAATTAGCAGAATACATAGCGAAATTTGGCTTCATGTTGCCTGAAGATAAAAGGC
AGGAAGGGTACCCAGCCAAATGGCTG

>MN784751.1 Small ruminant lentivirus isolate OV.20.1_Sheep.BEL
nonfunctional pol protein (pol) genes, partial sequence
GGGAAACAGCAGGAGGGG
GCCACGTGTGGTGCCGTCCGCACCCCCTATGTTGTAACAGAAGCACCACCAAATGTAGAAATAAGAGTAG
GAAAGGATTGGAAAAGAATGTTAATAGATACAGGGGCAGATAGGACTATAGTAAGGTATCATGATAATAC
GGGGATACCAAAGGGAAGAATAAAACTGCAGGGAATAGGGGGAATTATAGAAGGGGAACGATGGGAAAAC
GTAGTAATACAGTATAAAGAAAAGATAATAAAGGGAACCATAGTGGTGCTGCCTAGCAGCCCAGTAGAAG
TATTGGGAAGAGATAATATGGGGGAATTAGGAATAACATTAGTTATGGCAAATTTAGAAGAAAAGAAAAT
CCCTATTACTAAAGTAAAACTGAAAGAAGGGATGTAAGGGACCTCATATAGCACAATGGCCTTTTAACAC
AAGAAAAATTGGAGGGATTACAAGAAATAGTGGATAGATTAGAAAAAGAAGGAAAAGTAGGGAGAGCTCC
TCCACATTGGACATGTAACACTCCTATATTTTGTATTAAGAAAAAATCAGGAAAATGGAGGATGTTAATA
GACTTTAGAGAATTAAATAAACAAACAGAAGATTTGGCGGAAGCACAGTTGGGATTACCGCATCCGGGAG
GATTGCAGAAAAAGAAGCATGTAACAATCTTAGATATAGGGGATGCATATTTTACAATACCATTGTATGA
GCCGTATAGGCAATACACATGCTTTACCATGTTGAGTCCAAATAACCTAGGACCTTGTACAAGGTATTAT
TGGAAGGTACTGCCACAGGGATGGAAATTAAGTCCCTCAGTATATCAATTTACAATGCAGGAGATATTAA
GAGATTGGATAAAGGCGCACCCTATGATACAGTTTGGGATATATATGGATGATATTTACATAGGAAGTGA
GT

>AY454244.1 Small ruminant lentivirus isolate SNCR5525 pol protein
(pol) gene, partial cds
CTCAAAATGAAGAAGTGGATAAATATATTTCAGAAGTATTTTTGGCAAAGGAAGGGGAAGGAATTCTAGG
AAAGAGAAAAGAAGATGCAGGATATGATTTAATTTGCCCACAGGAAGTAAATATTCCACCGAACCGGGTG
AGAAAAATACCCATAGATCTTAAGTTGAATTTGAAAGAAAATCAATGGGCCATGATAACAACGAAAAGTA
GTTTTGCGAGTAAGGGAGTGTTTGTGCAAGGCGGAATAATAGATTCAGGATATCAAGGAACAATACAAGT
AGTGATATATAATAGTAATGACGTAGAAGTAATAATACCCCAGGGGAGAAAGTTTGCACAGTTAATTCTC
ATGCCGTTAATACATGAAGAATTGGAGCCATGGGAAAAACAAGAGAGACAGAGAGAGGAAAACAAGGAT
TTGGGTCCACGGGGGCCTATTGGATTGAGAATCTCCCATTAGCAGAAGAAGAGCATAGCAAATGGCATCA
AGATGCTATGTCATTGCATTTAGACTTTGGAATTCCTAGATCCGCCGCGGAAGATATAATACAACAATGT
GAGAGCTGTCAAGAAAATAAACTGCCAAGCGCCATTAGAGGAGGAAATAAAAGGGGAGTAGATCATTGGC
AAGTAGATTATACTCACTATGAGGATAAAATAATATTAACATGGATAGAAACAAATTCAGGATTGATTTA
TGCAGAAAGGGTAAAAGGAGAAACAGGGCAAGAATTTAGAATTCAAGTCATGAAATGGTACTCTACGTTT
GCTCCACAATCATTGCAGTCAGATAATGGACCTGCATTTATTGCAGAACCTACACAGCTCTTAATGAGAT
ATTTGGGAGTAGAGCATACAACGGGAATTCCGTGGAATCCCCAATCACAGGCCTTAGTAGAGAGAGCACA
TCAAACGTTAAAACATACTTTAGAAAAATTTGTTCCCATGTTTGTTGCATTTGAATCTGCTCTTGCTGCG
GCCCTAATAGCTCTAAATATAAAAAGAAAGGGTGGGCTAGGGACAAGCCCTATGGATATATTTATATTTA
ATAAAGAACAACAAAGAATACAGCAACAAAGTATAATGAATAAATCAAAAATTCGATTTTGTTATTACAG
AATCAGAAAG

>AY454231.1 Small ruminant lentivirus isolate SNCR6247 nonfunctional
pol protein gene, partial sequence
ATGTCATCATTGTGGAAAAAGGGGACATATGCAAAAGGAC
TGTAGACAAAAAAGAAATGGTGATATGAAACAGCAGGGAAACAACAGGAGGGGGCCACGTGTGGTGCCGT
CCGCGCCCCCTATGCTGTAACCGAAGCACCACCAAAGATAAAAATTAAAGTAGGAGGAACTTGGAAAGAA
GTATTAATAGATACGGGAGCGGATAGGACTATAGTAAAATATCATGATAATTCGGGGGTACCAAAAGGAA
GAATAAAATTACAGGGGATAGGAGGAATTATAGAAGGGGAAAAATGGGAGAGAGTGAGGATACAGTATAA

AGATAAAATAACATGGGGAACAATAGTGGTGCTGCCTAGCAGCCCAGTAGAAGTTCTAGGGAGAGATAAT
ATGAGGGAATTAGGAATAGGGCTAATTATGGCAAATTTAGAAGAGAAGAAAATTCCTATTACTAAAGTAA
AACTAAAAGAGGGTTGTAAGGGACCTCATATAGCGCAATGGCCGTTGACGCAAGAGAAACTAGAGGGATT
AAAAGAAATAGTTGATAGATTGGAAAAAGAAGGAAAATTAGGAAGGGCGCCACCGCATTGGACCTGTAAT
ACCCCTATATTTTGTATCAAGAAAAAATCAGGGAAATGGAGAATGTTGATAGATTTTAGAGAATTAAATA
AGCAAACAGAAGACTTGGCGGAGGCGCAGCTAGGATTACCACACCCGGGGGGATTGCAAAGAAAGAAACA
CATAACAATACTGGATATAGGGGATGCATATTTTACAATACCATTGTATGAGCCGTATAGGCAATATACC
TGCTTCACTCTACTAAGCCCAAATAATTTGGGACCATGTATAAGGTATTATTGGAAAGTTTTGCCGCAAG
GGTGGAAATTAAGTCCCTCGGTATATCAGTTTACCATGCAGGAAATATTAAGAGATTGGATAAAAAAGCA
TCCTGTGATACAATTTGGGATCTATATGGATGACATTTATATAGGAAGTGATTTAGAGATAGGAGAACAC
AGAAAAATAGTGGAAGAGCTTGCCAGTTACATTGCTCAATATGGATTTATGCTACCTGAAGAAAAGAGAC
AAGAAGGGTATCCGGGGGAAGGG

>MN784757.1 Small ruminant lentivirus isolate LK.5.4_Sheep.BEL
nonfunctional pol protein (pol) gene, partial sequence
GGGAAACAGCAGGAGGGGGCC
ACGTGTGGTGCCGTCCGCGCCCCCTATGTTGTAACAGAAGCACCACCCAAGTAGCAATAATAGTGGGAAA
AGTTTGGAAGCAAATGCTAGTGGATACGGGAGCAGACAGAACGATAATAAAAAGCCATGATAATACGGGA
ATACCTAAGGGGAGAATAAAACTGCAGGAATTGGGGGAATTATAGAAGGGGAAAGATGGGACAATGTAAC
CTTGCAGTATAAAGGAAAAGTAATACAGGGTACAATAGTAGTGTTGCCTTCAAGTCCGGTTGAAGTGCTA
GGAAGAGATAACATGAGCAAACTAGGAATAGGATTAATTATGGCCAATTTAGAAGAAAAGAAAATTCCAA
TCACAGAAGTGAAATTAAAAGAGGGATGTATGGGGCCTCATGTGGCACAGTGGCCTTTAACACAGGAGAA
ATTAGAAGGATTAAAAGAAATTGTAGACAGATTAGAAAAAGAAGGGAAAGTAGGAAGAGCACCTCCACAC
TGGACATGTAATACTCCTATATTTTGCATTAAGAAAAATCAGGAAAATGGAGAATGCTCATAGATTTTAG
AGAATTAAACAAACAAACAGAGGATTTAGCAGAGGCACAATTAGGGTTACCGCATCCAGGAGGGTTGCAA
AAGAAGAAACATGTGACAATATTAGATATAGGGGATGCATACTTTACAATACCATTGTATGAGCCTTATA
GGCAATATACATGCTTTACAATGTTGAGTCCAAATAATTTAGGGCCATGTGTAAGATATTATTGGAAGGT
CTTGCCACAGGGGTGGAAATTAAGCCCCTCGGTATATCAGTTTACCATGCAGAAAATATTAAGAGATTGG
ATAGAAGAACATCCTATGATACAATTTGGAATCTATATGGATGATATTTATATAGGCAGTGATC

>AM419951.2 Small ruminant lentivirus proviral partial pol gene for
pol protein, strain B_S604, sub strain 1
CTCAAAATGAAGAAATAGATAAGTACATATCAGAAATATTTTTGGCAAAAGAAGGAAATGGAATTTTGGG
GAAAAGAGAAGAAGATGCGGGATATGATTTAATATGYCCRCAAGAGACAGTTATCCCCGCCGGACARGTA
AAAAAGATTGCCATAGATCTGAGATTAAATTTGAAAAGAAACCAATGGGCCATGATAGGRACAAAAAGTA
GTTTTGCRAATARGGGWGTRTTTGTACAAGGAGGAATAATAGATTCAGGATATCAGGGGACAATACAGGT
AGTAGTATATAATAGTAATAATATAGAAGTAGTAATACCCCAAGGGAGAAAGTTTGCACAATTGATTTTA
ATGCCATTGATGCATGAAGAATTGGAGCCTTGGGGAGAAACAAGGAAATCAGAAAGAGGAGAACAGGGGT
TTGGATCGACAGGAGCGTTCTGGATAGAGAATATCCCAAGAGCAGAGGAAGATCATGACAGATGGCATCA
GGATGCATTGTCTTTGCATCTAGACTATGGRATCCCCAGAGCAGCTGCGGARGATATAGTGCAACAATGT
GAGGTGTGTCAAGAGAATAAAATGCCTAGCACACAGAGGGGTGGAAATAAAAGGGGAGTARACCATTGGC
AAGTAGATTATACCCATTATGAAGATAAAATCATACTAGTATGGGTAGAAACAAATTCAGGGTTAATATA
TGCAGAAAAAGTAAAGGGAGAAACAGGACAGGAGTTTAGAATACAAGTCATGAAATGGTATGCCATGTTT
TCTCCGAGTTCATTACAATCAGATAATGGACCAGCTTTCGTAGCAGAACCTACGCAGCTCTTAATGAAGT
ATTTRRAAATAGAGCACACTACGGGAGTGCCATGGAACCCGCAATCACAAGCCTTAGTGGAAAGGGCACA
TCAGACATTAAAGCATACATTAGAAAAATTCATCCCCATGTTYGTYGCATTCGAATCAGCTCTTGCTGCT
GCACTCATAGCTCTAAATATAAAAAGAAAGGGTGGGCTAGGGGCAAGCCCTATGGATATATTTATATTTA
ATAAGGAACAGAAAAGAATACAGCAACAATTTAAAACAAATCCGTCAAAAATTCGGTTTTGTTATTACAG
GATCAGAAAA

>AY454191.1 Small ruminant lentivirus isolate SNCR5637 nonfunctional
pol protein gene, partial sequence
ATGCCATCATTGTGGAAAAAAGGGACATATGCAAAGGGAT
TGTAGAAAGAAGAAAAATGAGGAACGGCAGCAGGGAAACAGCAGGAGGGGGCCACGTGTGGTGCCGTCCG
CGCCCCCTATGTTGTAACAGAAGCACCACCTAGGATAAAAGTAAAAATAGGAACAGTTTGGAAAGAAGTA
TTAATAGATACAGGGGCAGATAGGACGATAGTTAGACATCATGATAATTCAGGGATACCTAAGGGAAGAA
TAAAATTACAAGGAATAGGGGGAATCATAGAAGGAGAAAAATGGGATCAAGTGTTGTTGCAATATAAAGA
AGAAATTATAACAGGGACACTCGTAGTACTACCAACGAGTCCTGTAGAGGTATTAGGGAGAGATAATATG
GGGAAATTAGGAATAGGACTAATTATGGCAAATCTAGAAGAAAGAAAAATACCCATCACAGAGGTAAGGT
TAAAAGAGGGATGTAAAGGACCCAATATAGCACAATGGCCCTTGACTCAGGAAAAGTTAGAAGGGCTAAA
AGAAATTATAGATAGGTTGGAGAAAGAAGGAAAATTGGGGAGAGCGCCGCCACATTGGACATGCAATACA

CCAATATTTTGTATTAAGAAGAAATCAGGGAAATGGAGAATGTTGATAGATTTTAGGGAATTAAACAAGC
AAACAGAAGACTTAGCGGAGGCACAGCTTGGACTACCCCATCCCGGAGGATTGAAAAAGAAAAAGAATGT
CACAATCCTCGATATTGGGGATGCGTATTTTACAATACCATTGTATGAACCTTATAGACAGTATACATGT
TTTACTCTGCTAAGTCCAAATAATTTGGGACCATGTGTGAGGTATTATTGGAAGGTGTTGCCTCAGGGGT
GGAAGTTAAGTCCATCAGTATATCAGTTTACAATGCAAAAAATATTAAGGGATTGGATAGAGGAACATCC
TATGATACAGTTTGGGATATATATGGATGATATTTATATAGGAAGTGATCTAGAAATAACAAAACATAGA
CAAATAGTGAATGAATTAGCAGATTACATAGCACAATTTGGCTTCATGTTGCCTGAAGATAAAAGACAGG
AAGGGTATCCAGCCAAATGGCTA

>AY454232.1 Small ruminant lentivirus isolate SNCR6249 nonfunctional pol protein gene, partial sequence
ATGTCATCATTGTGGAAAAAGGGGACATATGCAAAAGGAC
TGTAGACAAAAAAGAAATGGTGATATGAAACAGCAGGGAAACAACAGGAGGGGGCCACGTGTGGTGCCGT
CCGCGCCCCCTATGCTGTAAGCGAAGCACCACCAAAGATAAAAATTAAAGTAGGAGGAACTTGGAAAGAA
GTATTAATAGATACGGGAGCGGATAGGACTATAGTAAAATATCATGATAATTCGGGGGTACCAAAAGGAA
GAATAAAATTACAGGGGATAGGAGGAATTATAGAAGGGGAAAAATGGGAAAAGGTGAGGATACAGTATAA
AGATAAAACAACATGGGGAACAATAGTGGTGCTGCCTAGCAGCCCAGTAGAAGTTCTAGGGAGAGATAAT
ATGAGGGAATTAGGAATAGGGCTAATTATGGCAAATTTAGAAGAGAAGAAAATTCCTATTACTAAAGTAA
AACTAAAAGAGGGTTATAAGGGACCTCATATAGCGCAATGGCCGTTGACGCAAGAGAAACTAGAGGGATT
AAAAGAAATAGTTGATAGATTGGAAAAAGAAGGAAAATTAGGAAGGGCGCCACCGCATTGGACCTGTAAT
ACCCCTATATTTTGTATCAAGAAAAAAATCAGGGAAATGGAGAATGTTGATAGATTTTAGAGAATTAAATA
AGCAAACAGAAGACTTGGCGGAGGCGCAGCTAGGATTACCACACCCGGGGGGATTGCAGAGAAAGAAACA
CATAACAATACTGGATATAGGGGATGCATATTTTACAATACCATTGTATGAGCCGTATAGGCAATATACC
TGCTTCACTCTACTAAGTCCAAATAATTTGGGACCATGTATAAGGTATTATTGGAAAGTTTTGCCGCAAG
GGTGGAAATTAAGTCCCTCGGTATATCAGTTTACCATGCAGGAAATATTAAGAGATTGGATAAAAGAGCA
TCCTATGATACAATTTGGGATCTATATGGATGACATTTATATAGGAAGTGATTTAGACATAGGAGAACGC
AGAAAAATAGTGGAAGAGCTTGCCAGTTACATTGGTCAATATGGATTTATGCTACCTGGAGTAAAGAGAC
AAGAAGGGTATCCGGGGAGGGG

>AY454176.1 Small ruminant lentivirus isolate SNCR5561 pol protein genes, partial cds
ATGCCATCATTGTGGAAAAAGGGGACATATGCAAAAGGAT
TGCAGAAAGAAGAAAAAAGAAGTAGAGCAGCAGGGAAACAGAAGGAGGGGGCCACGTGTGGTGCCGTCCG
CGCCCCCTATGTTGTAACAGAAGCACCACCTAGGATAAAAGTAAAAATAGGAACCGTATGGAAAGAAGTA
CTAGTAGATACAGGGGCAGATAGGACTATAGTGAGGCATCATGATAATTCAGGGATACCCAAGGGAAGAA
TAAAATTACAAGGAATAGGGGGAATTATAGAAGGAGAAAAATGGGATAGAGTATTAATACAATATAAAGA
AGAAATTATAAAAGGGACACTCGTGGTATTGCCGACGAGTCCTGTAGAGGTATTAGGAAGAGATAACATG
GGGAAATTAGGAATAGGGCTAGTTATGGCAAATCTAGAAGAAAGAAAAATTCCCATCACAGAGGTAAAGT
TAAAAGAAGGGTGTAAAGGACCACATATAGCCCAATGGCCTTTGACCCAAGAAAAGTTAGAAGGGTTAAA
AGAAATTATAGATAGGTTGGAGAAAGAAGGAAAATTGGGGAGAGCGCCGCCACATTGGACATGCAATACA
CCAATATTTTGTATTAAGAAAAAAATCAGGGAAATGGAGAATGTTGATAGATTTCAGAGAATTAAACAAGC
AAACAGAAGACTTGGCGGAGGCACAGCTTGGACTACCACATCCCGGAGGATTGAAAAAGAAAAAGAATGT
CACAATCCTCGATATTGGGGATGCGTATTTTACAATACCATTGTATGAACCTTATAGACAATATACATGT
TTTACTCTGCTAAGTCCAAATAATTTGGGACCATGTGTGAGATATTATTGGAAAGTGTTGCCTCAGGGGT
GGAAGTTAAGTCCATCAGTATATCAGTTTACAATGCAAAGAATATTAAGGGATTGGATAGAGGAACATCC
TATGATACAGTTTGGAATATATATGGATGATATTTATATAGGAAGTGATTTAGAAATAACAAAACATAGA
CAGATAGTGGATGAATTAGCAGATTACATAGCACAATTTGGCTTTATGTTACCTGAAGATAAAAGACAGG
AAGGGTATCCGGCCAAATGGCTT

>AF479638.1 Ovine lentivirus strain P1OLV, complete genome
AT
GGGTGAATTAGGATTTAGATTAGTTATGGCAAATTTAGAAGAAAAGAAAATTCCCGTAACAGAAGTAAAA
TTAAAGGAAGGATGTAAAGGACCTCATATAGCGCAATGGCCATTGACTCAAGAAAAAATTAGAAGGATTGC
AAGAAATAGTAGACAGATTAGAAAAAGAAGGGAAAGTAGGAAGAGCACCGCCTCATTGGACATGTAACAC
TCCAATATTTTGTATTAAGAAAAAAATCAGGGAAATGGAGGATGTTAATAGATTTCAGAGAATTAAATAAG
CAAACAGAAGATTTGGCGGAAGCTCAGTTAGGACTCCCGCATCCTGGAGGACTTCAGAAAAAGAAACATG
TAACAATATTAGATATAAGTGATGCATATTTTACAATTCCTTTGTTTGAACCCTATAGAAAGTATACATG
TTTTACTATGCTAAGCCCAAATAATTTAGGACCCTGCACCAGATATTATTGGAAGGTGCTACCTCAGGGG
TGGAAGTTAAGTCCCGCAGTATATCAGTTTACAATGCAAAGTATATTAAGAAGTTGGATAGCAAAACATC
CTTTAATACAATTTGGGATATATATGGATGACATCTATATAGGAAGTGATATGGACATAGAAAAACATAG

AGGGGTGGTGGAGGAATTGGCAGCGTATATTGCCCAATATGGGTTTATGCTGCCTGAAGAAAAGAGACAA
GAAGGATACCCAGCAACATGGCTTGGATTTGAATTACATCCAGATAAATGGAGATTTCAGAAACATACTT
TGCCAGACTTAAAAGAAGGGACGATAACATTAAATAAATTACAAAAAGTAGTAGGAGACTTAGTCTGGAG
ACAATCCTTAATAGGGAAAAGTATACCAAATATATTGAAGTTAATGGAAGGGGATAGAGCGCTGCAGAGT
GAGAGGAAAATACAACAAGTACATGTTCAGGAATGGGAAAGATGTAAGAGAAAACTAGAAGAAATGGAAG
GAAAGTATTATGATGAAGGAAAAGATGTGTATGGACAAATAGATTGGGGGGATAGAGCTGTAGAATATGT
AGTGTTCCAGGAAAAGGGAAAGCCTTTATGGGTCAATGTAGTACATAGTATAAAAAATTTAAGTCTAGCA
CAGCAAATTATCAAAGCAGCGCAGAAGATGACACAAGAAGTAATAGTCAGAACAGGGAAGATACCGTGGA
TACTGTTACCAGGGAAAGAAGAGGATTGGATACTGGAATTACAAGCGGGAAATATAACCTGGATGCCATC
ATTTTGGTCGTGTTACAGGGGGTCAGTAAGATGGAGGAAGAGAAATATAGTAGCAGAGGTAGTAGCGGGA
CCAACATATTATACTGATGGAGGAAAGAAAAATGGAATAGGAAGCTTAGGCTATATAGCATCAACAGGGG
AGAAATACAGAAAACGTGAACAGGGAACAAATCAACAGTTAGAATTAAGGGCAATAGAGGAAGCGTGTAA
GCAAGGGCCAAAAGTAATGAATGTAGTAACAGATAGCAGATATGCATTTGAATTTATGTTACGGGACGGA
GATGAAGAAGTTATCAAAAATCCAATACAAGCCAGAATTATGAAATTGATACATAACAAGGATAAGATAG
GAATACATTGGGTGCCTGGACACAAAGGAATTCCACAAAATGAAGAAATTGATAAATATATTTCAGAAAT
ATTTTTAGCAAAGGAAGGAGAAGGGATTCTCCCCAAAAGGAGAGAAGACGCGGGATATGACTTAATATGT
CCACAAGAAGTGAGCATTCCAGCAGGACAAGTAAAAAAGATACCAATTGATCTAAGGTTAAACCTAAAGG
AGGATCAATGGGCCCTGATAGGGACCAAAAGTAGTCTTGCAAGTAAGGGAGTATTTGTACAAGGAGGGAT
CATAGATTCAGGGTATCAAGGACAGGTACAGGTAGTAATTTATAACAGTAATGATAGGGAAGTAGTTATA
CCACAGGGGAGAAAATTTGCACAATTAATACTCATGCCTTTGGTGCATGAAGAGTTAGGAACGTGGGGAA
AAACAAGGAAGACAGAAAGAGGGAAAGAAGGATTTGGATCAACAGGAGCATATTGGGTCGAAAATATTCC
AATAGCAGAAGAGGATCATCACAGATGGCATCAAGATGCTATGTCATTACAATTAAGCTTTGGAATACCC
AGAGCTGCAGCTGAGGATATAGTACAGCAATGTGAAGTATGTCAAGAAAGTAAAATGTCGAGTACTATCA
GAGGAGGTAACAAAAGAGGGATAGATCATTGGCAGGTAGACTATACTCATTATGAAGACAAAATAATACT
AGTATGGATAGAAACAAATTCAGGGTTAATATATGCAGAAAGAGTAAAAGGGGAAACGGGACAAGAATTT
AGGATGCAAGTAATGAAATGGTATGCTCTGTTTGCCCCAAGTTCATTGCAGTCTGATAATGGACCTGCAT
TTGTGGCAGAACCAACACAACTGTTAATGAAATATTTAGGGATAGAACACCGTACAGGAATACCATGGAA
CCCTCAATCACAAGCATTAGTAGAGAGAGCCCATCAAACATTCAAGTATACATTAGAAAAATTGCTCCCT
ATGTTTGCAGCATTTGAATCTGCAGTTGCGGCTACCCTAATAGCTCTAAATATAAAAAGAAAGGGTGGGC
TAGGGACAAGCCCTATGGATATATTTATCTTCAATAAAGAACAGCAAAGAATACAAAAACAACAGCAAAT
AAATCAGTCGAAAAATCGATTTTGTTATTACAGGATCAGAAAAAGAGGACACCCAGGTGACTGGCAGGGA
CCAACACAGGTACTGTGGGAAGGGGAAGGAGCAATAGTAGTCAAAGATAAAACTACAGAGAAGTATTTAG
TAATAGCTAACAAAGATGCAAAATTCATCCCACCGCCTAAAGAAATACAAAAAGAATAA

>MZ484405.1 Small ruminant lentivirus isolate BEL-LX160316, complete genome
ATGCTTATAGA
TTTTAGAGAATTAAACAGGCAGACAGAGGACTTGGCAGAGGCACAATTGGGGTTACCGCATCCAGGAGGG
TTACAGAAGAAGAAACATGTGACAATATTAGATATAGGGGATGCGTATTTTACAATACCATTATATGAGC
CTTATAGACAATATACATGCTTTACAATGTTGAGCCCGAATAATTTAGGGCCATGTGTAAGATATTATTG
GAAGGTGTTGCCACAGGGGTGGAAATTAAGCCCCTCGGTATATCAGTTTACCATGCAAAAGATATTAAGA
GATTGGATAGAAGAACACCCTATGATACAATTTGGAATTTATATGGATGATATTTATATAGGAAGTGACC
TGGAAATAACAAAGCATAGGCAAATAGTAGAAGAATTAGCAGGGTATATAGAGCAATATGGATTTATGTT
ACCAGAAGAAAAGAGACAGGAAGGATATCCTGCCAACTGGTTAGGATTTGAATTACACCCGGATAAATGG
AAATTTCAAAAGCATATGTTACCAGAACTAAGAGAGGGAAAAATCACTTTAAACAAATTGCAAAAATTAG
TGGGAGATTTAGTTTGGAGGCAATCCTTAATAGGAAAAGGGATACCAAATATATTAAAGTTAATGGAAGG
AGACAGAGCATTACAAAGCGAAAGGAAAATAGAAAGAATACATGTTGAAGAATGGGAAAGGTGTAAGAAA
AAACTGGAAGAAATGGAAGGGAATTATTATGATGCAGAAAAAGATATCTATGGGCAAATAGATTGGGGAA
ATAAAGCCATAGAATATATAGTATTCCAAGAAAAGGGGAAACCATTATGGGTGAATGTGGTACATAGCAT
AAAAAACTTAAGTCAGGCACAACAGATTATTAAAGCAGCGCAAAAACTTACGCAGGAAGTAATAATCAGG
GTAGGAAAAATACCTTGGATATTGTTGCCCGGGAAGGAAGAGGACTGGATATTAGAATTACAAGTAGGAA
ACATAACGTGGATGCCATCATTTTGGTCATGCTATAGAGGATCAGTAAGATGGAAGAAAAGAAATGTAGT
GACAGAAGTAGTGGAAGGTCCAACATATTACACCGATGGAGGCAAGAAAAATGGCATAGGAAGTTTAGGA
TATATTGCATCCACAGGGGAAAAATTTAGAAAACATGAAGAAGGGACAAATCAGCAATTAGAGTTAAGAG
CAATCGAGGAGGCCTGTAAGCAAGGACCAGAGAGGATGAATATAGTCACAGATAGTAGATATGCATTTGA
ATTTTTGCAGAGAAATTGGGATGAAGAAGTAATAAAAAATCCAATTCAGGCGAGGATTATGAAAATGGTG
CATAAAAAAGAAAAAATAGGAGTACATTGGGTGCCGGGGCATAAAGGGATCCCCCAAAATGAAGAAATAG
ATAAATTTATCTCGGAAGTATTTTTAGCAAAAGAAGGAGCTGGAATATTACCAAAGAGAGCAGAAGATGC
GGGGTATGATTTAATATGCCCACAAGAAGTGAGTATCCCAGCAGGACAAGTGCGAAAGATTCCAATTGAT
TTAAAAGTAAATCTAAAAAAGGAGCAATGGGCATTAATAGGCACAAAAAGTAGTTTTGCAAGTAAGGGAG
TATTTGTGCAAGGAGGCATAATAGATTCTGGGTATCAGGGGACAATACAGGTAGTAATATACAATAGCAA

TGACAAAGAAGTTATTATCCCCCAGGGAAGAAAATTTGCACAATTAATCCTCATGCCTTTAACACATGAG
GAATTAGAACCATGGGGAGAACAGAGGAGAACAGAAAGGGGGAATCAGGGGTTTGGATCAACAGGAGCAT
ATTGGATTGAGAATATACCCTTGGCAGAGGAGGAGCATAGTAAATGGCATCAAGATGCGATATCGTTGCA
TCTCGACTTTAGAATACCGAGATCGGCTGCTGAGGATATAGTGCAGCAGTGTGAGATATGTCAAGAAAAT
AAGATGCCTAGCACACTGAGAGGAGGCAACAAGAGGGGAATAGATCATTGGCAAGTGGATTACACTCATT
TTGAAGATAAGATATTATTGGTATGGGTAGAAACAAATTCAGGGTTAATATATGCAGAAAAAGTAAAAGG
AGAGACAGGACAAGAATTTAGAACTCTAACTATGAAATGGTATGCTCTGTTTGCCCCAAAATCATTGCAG
TCTGATAATGGACCAGCATTTGTAGCAGAACCAACACAAATGCTAATGAAATATTTAGGGATAACACATA
CAACAGGTATCCCCTGGAACCCACAATCACAAGCACTAGTGGAGAGAGCCCATCAGACATTAAAAAATGT
AGTAGAAAAATTCGGGCCTATGTTTGTGGCATTTGAATCTGCACTAGCAGCTGCTCTAATAGCTCTAAAT
ATAAAAAGAAAGGGTGGGCTAGGGACAAGCCCTATGGATATATTCATATTTAATAAGGAACAGCAAAGAA
TACAACAACAGTCTAAACTAAATATAGAAAAAATTCGATTTTGTTATTACAGAATCAGAAAAAGAGGACA
CCCAGGTGATTGGCTGGGACCGTCACAGGTACTTTGGGAAGGGGAAGGAGCAGTGGTAGTAAAGGACAAA
ACCTTAGAAAAGTATTTAGTAATAGCCAATAAAGATGTCAAGTTTATCCCACCGCCAAAAGAAATATCAA
AAACACAAGACTAG

>MH374287.1 Small ruminant lentivirus isolate SRLV038, complete genome
ATGGGGAAATTAG
GTATAGGATTAGTAATGGCTAATTTAGAAGAAACAAAGATTCCTATTACGGAAGTTAAGTTGAAAGAGGG
CTGTAAGGGGCCTCATATCCCGCAATGGCCGTTAACACAAGAAAAATTGGAAGGACTGAAAGAAATAGTA
GATAGGTTAGAGAAAGAAGGAAAGTTAGGGAGAGCGCCACCACACTGGACATGTAATACTCCTATATTCT
GTATTAAGAAGAAATCAGGAAAATGGAGAATGTTAATAGATTTTAGAGAATTGAATAGACAAACAGAGGA
GTTAGCAGAAGCGCAATTGGGTCTACCCCACCCTGGGGGATTACAAAGAAAGAGGAATGTCACTATATTA
GACATAGGGGATGCATATTTTACAATCCCCCTATATGAACCCTATAGACAATATACATGTTTTACATTGT
TGAGCCCCAATAATCTGGGACCCTGTGTAAGGTATTATTGGAAAGTATTACCACAAGGTTGGAAATTAAG
TCCAGCGGTGTATCAATTTACAATGCAAAAGATTTTAAAAAACTGGATAGAAGCTCATCCAAACATACAG
TTTGGTATATACATGGATGATATCTATATAGGCAGTGATATGGATATTAGAGAACATAGGGCCATAGTGA
ACAATTTAGCAGAGTATATAGCCCACTATGGGTTTATGCTACCAGAGGACAAAAGACAAGAGGGCTATCC
AGCAAAATGGTTAGGATTTGAATTGCATCCAGATAAATGGAGGTTTCAGAAGCATATATTGCCAGAAGTA
ATTGAAGGAAAGATAACGTTAAATAAGTTGCAAAAGTTAGTAGGAGATTTGGTGTGGAGGCAGACTCTAA
TAGGAAAAAGTATACCAAATATATTGAAATTAATGGAAGGGGATAGAGATTTACGAAGCGAAAGGAAGAT
AGAGGAGATACATGTAAAAGAATGGGAACAGTGCAGGAAAAAATTAGCGGAAATGGAAGGGACTTATTAT
GATGAAGAAAAAGATGTTTATGGACAAATAGATTGGGGAAATAAAGCAATAGAATATATAGTATTCCAGG
AAAAGGGAAAACCTTTATGGGTGAATGTGGTACATAATATTAAAAATTTGAGTCAACAGCAACAAATTAT
TAAGGCAGCACAAAAGCTCACACAAGAAGTGATAATTAGAATAGGAAAAATTCCATGGATACTATTGCCA
GGGAGAGAAGAGGATTGGATATTAGAACTGCAAGCTGGTAATATAACGTGGATGCCATCATTTTGGTCAT
GTTATCGGGGATCAGTAAGATGGAAGAGAAGAAATGTGGTAACAGAAGTAGTGGAAGGACCAACATATTA
CACGGATGGAGGGAAGAAAAACAAGGTAGGAAGTTTGGGATATATTTCCTCAACAGGGGAAAAATATAGA
AAACATGAGGAAGGGACAAATCAGCACTTAGAATTAAGAGCTATAGAAGAAGCATGTAAACAAGGACCCG
CATCTATGAATATAGTAACAGACAGCCGGTATGCATATGAATTTATGATAAGGAATTGGGATGAAGAAGT
AATAAAAAACCCAATACAGGCAAGAATTATGAAATTGATACATGAAAAGGAGAAAATAGGCATACATTGG
GTTCCAGGGCACAAAGGAATACCGCAAAATGAAGAAATAGATAAGTATATATCAGAAATATTTTTAGCAA
GAGAAGGAGAAGGGATTCTCCCAAAAAGAAGGGAAGATGCAGGGTATGATTTAATATGCCCCGAGGAAGT
ATGTATCGCAGCAGGACAAGTAAAGAGAATACCGATTGACTTAAGATTAAATTTGAAAAAGAACCAATGG
GCTATGATAGGGACAAAAAGCAGTTTTGCAAGCAAAGGGGTATTCGTACAAGGAGGAATAGTAGATTCAG
GATATCAGGGAACTATACAAGTAGTAATTTATAATAGTAATAATAGGGAAGTGGTAATACCACGAGGAAG
AAAATTTGCTCAGATAATTCTCATGCCATTAATACATGAAGAATTAGAACCTTGGGGCCAAGGAAGAAAA
ACAGAGAGAGGAGAAAAAGGATTTGGATCAACAGGAGCATATTGGATAGAGAATATACCTAGGGCAGAAG
AGGAACATGAGAAATGGCATCAGAGTGCTGAAACATTGCAGCTTGATTTTGGAATTCCTAGGACAGCAGC
AGAAGATATAGTGCAGCAATGCGAAGTATGTCAGGAAAATAAAATGCCTAGTAATATAAGAGGGGGAAAT
AAGAGAGGGGTAGACCATTGGCAAGTGGACTATACCCATTATGAGGATAAAATAATATTGGTATGGATAG
AAACAAATTCAGGATTAATATATGCGGAAAGAGTAAAAGGAGAGTCAGGGGCAGAATTTAGAACACAAGT
AATAAAATGGTATAGTATATTTAGACCAAAATCGCTACAATCAGATAATGGACCTTCTTTCGTAGCAGAG
CCCACTCAATTATTAATGGCATATCTAGGGGTCGAGCATCATACAGGAATACCTTGGAACCCCCAATCAC
AAGCAATAATAGAACGTACCCACCAGACGTTAAAGCATACTCTACAAAAGTTTGAAAACAATTTTGTATC
ATTTGACTCTGCATTGGCAGCGGCGCTTATAGCACTTAATATAAAAAGAAAGGGTGGGCTAGGGACAAGC
CCTATGGATATATTCATATTTAATAAAGAACAGCAAAGAATACAACAACAAAGTATAAAAAATCAATCAA
AAATTCGGTTTTGTTATTACAGGGTCAGAAAAAGAGGACACCCGAGTGAGTGGTTTGGACCTACTCAGGT
ATTATGGGAAGGGGAAGGAGCGATAGTAATTAAAGATAAAGAATCAGAAAAATATCTAGTGATAGCCTTT
AAAGATGTCAAATTCATACCACCGCCGAAAAATATATAA
