# Supplementary figures and images for: Amplicon sequencing with Oxford nanopore technologies as a diagnostic alternative for small ruminant lentiviruses in sheep
Source: Sci Rep. 2026 Jan 25;16:6212. doi: 10.1038/s41598-026-36989-y (PMC12905341; doi:10.1038/s41598-026-36989-y)

Consensus Tree

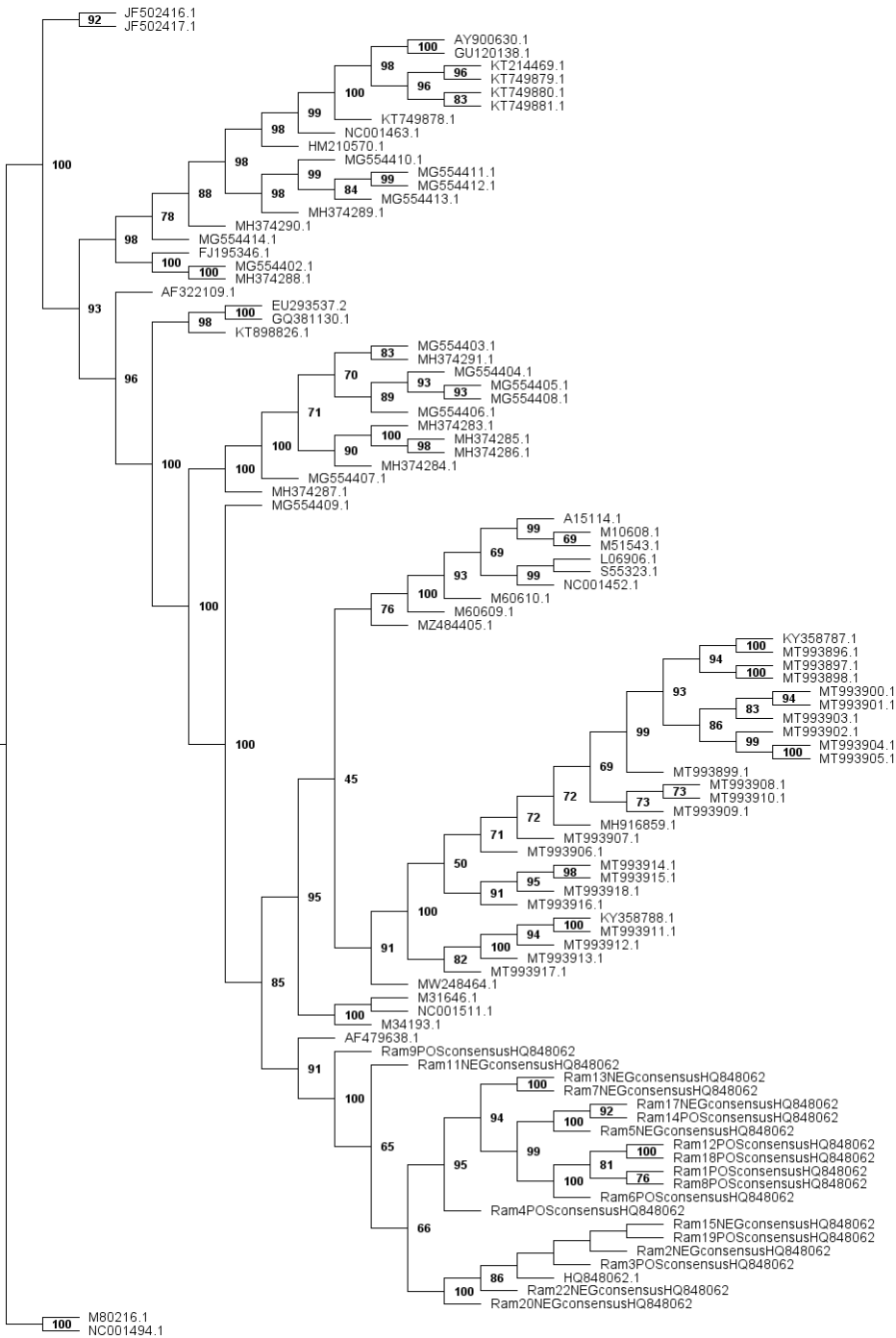

## ML Tree

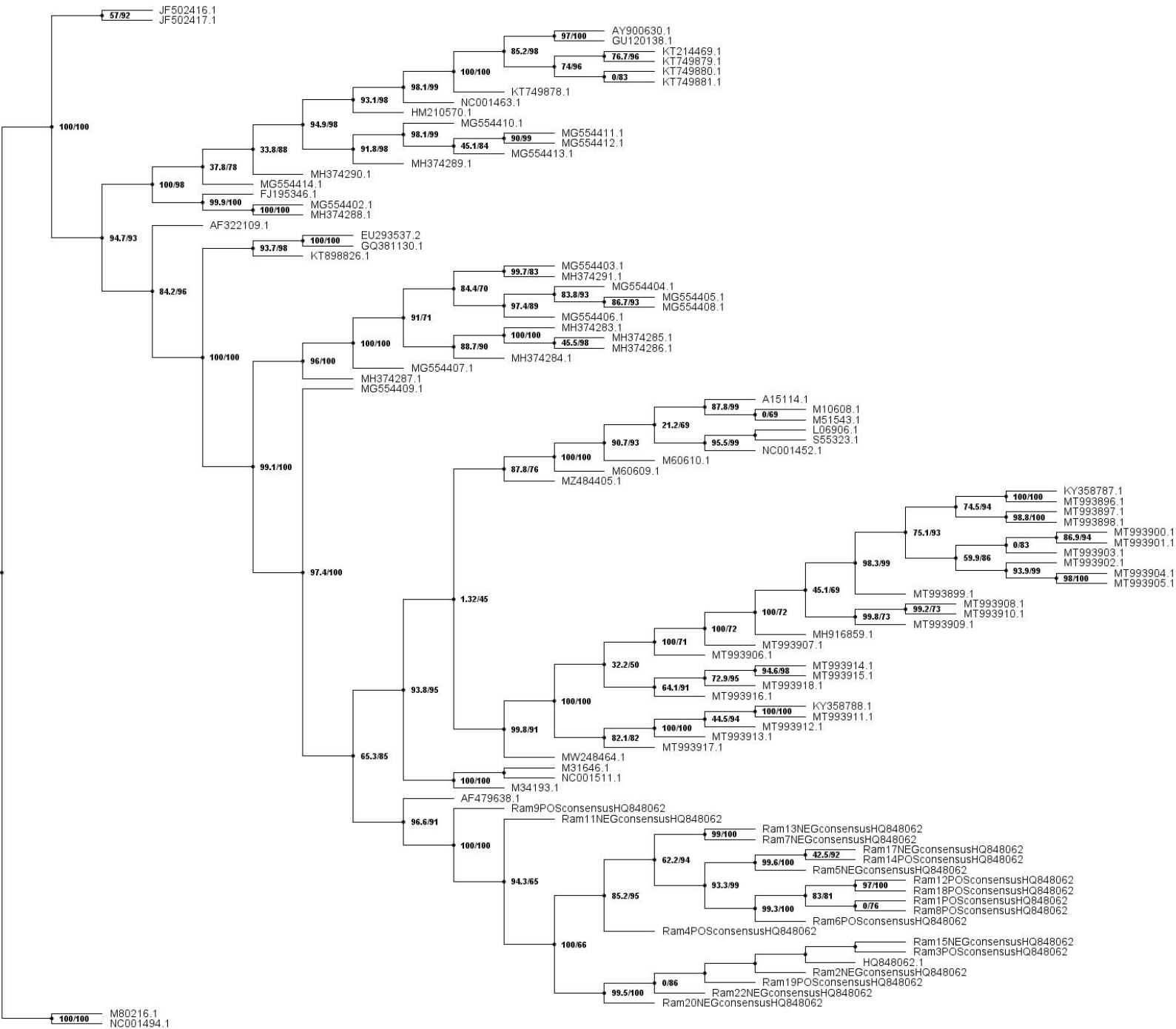

Supplement: Supplementary file 8 — Supplementary Material 8 [file 41598_2026_36989_MOESM8_ESM.pdf]
